# The state of food systems worldwide in the countdown to 2030

**DOI:** 10.1038/s43016-023-00885-9

**Published:** 2023-12-19

**Authors:** Kate R. Schneider, Jessica Fanzo, Lawrence Haddad, Mario Herrero, Jose Rosero Moncayo, Anna Herforth, Roseline Remans, Alejandro Guarin, Danielle Resnick, Namukolo Covic, Christophe Béné, Andrea Cattaneo, Nancy Aburto, Ramya Ambikapathi, Destan Aytekin, Simon Barquera, Jane Battersby, Ty Beal, Paulina Bizzoto Molina, Carlo Cafiero, Christine Campeau, Patrick Caron, Piero Conforti, Kerstin Damerau, Michael Di Girolamo, Fabrice DeClerck, Deviana Dewi, Ismahane Elouafi, Carola Fabi, Pat Foley, Tyler J. Frazier, Jessica Gephart, Christopher Golden, Carlos Gonzalez Fischer, Sheryl Hendriks, Maddalena Honorati, Jikun Huang, Gina Kennedy, Amos Laar, Rattan Lal, Preetmoninder Lidder, Brent Loken, Quinn Marshall, Yuta J. Masuda, Rebecca McLaren, Lais Miachon, Hernán Muñoz, Stella Nordhagen, Naina Qayyum, Michaela Saisana, Diana Suhardiman, U. Rashid Sumaila, Maximo Torero Cullen, Francesco N. Tubiello, Jose-Luis Vivero-Pol, Patrick Webb, Keith Wiebe

**Affiliations:** 1 School of Advanced International Studies, Johns Hopkins University, Washington, DC USA; 2 Columbia Climate School, Columbia University, New York, NY USA; 3 Global Alliance for Improved Nutrition, Geneva, Switzerland; 4 College of Agriculture and Life Sciences, Cornell University, Ithaca, NY USA; 5 Cornell Atkinson Center for Sustainability, Cornell University, Ithaca, NY USA; 6 Food and Agriculture Organization of the United Nations, Rome, Italy; 7 Harvard T.H. Chan School of Public Health, Boston, MA USA; 8 Glocolearning, Genk, Belgium; 9 Alliance of Bioversity and CIAT, Cali, Colombia; 10 International Institute for Environment and Development, London, UK; 11 International Food Policy Research Institute, Washington, DC USA; 12 International Livestock Research Institute, Addis Ababa, Ethiopia; 13 CGIAR, Montpellier, France; 14 Wageningen Economic Research Group, Wageningen University, Den Haag, the Netherlands; 15 Bloomberg School of Public Health, Johns Hopkins University, Baltimore, MD USA; 16 Research Center of Nutrition and Health, National Institute of Public Health, Cuernavaca, México; 17 University of Cape Town, Rondebosch, South Africa; 18 Global Alliance for Improved Nutrition, Washington, DC USA; 19 Regenerative Collaborations, Nijmegen, the Netherlands; 20 CARE, Geneva, Switzerland; 21 University of Montpellier, Montpellier, France; 22 Cirad, Montpellier, France; 23 ART-DEV, Montpellier, France; 24 EAT Forum, Montpellier, France; 25 Regional Bureau for Latin America and the Caribbean, World Food Programme, Panama City, Panama; 26 Oak Ridge National Laboratory, Oak Ridge, TN USA; 27 American University, Washington, DC USA; 28 Natural Resources Institute, University of Greenwich, Kent, UK; 29 World Bank, Washington, DC USA; 30 School of Advanced Agricultural Sciences, Peking University, Beijing, China; 31 School of Public Health, University of Ghana, Accra, Ghana; 32 Ohio State University, Columbus, OH USA; 33 WWF, Washington, DC USA; 34 Paul G. Allen Family Foundation, Seattle, WA USA; 35 University of Rome La Sapienza, Rome, Italy; 36 Friedman School of Nutrition Science and Policy, Tufts University, Boston, MA USA; 37 Joint Research Centre, European Commission, Ispra, Italy; 38 Royal Netherlands Institute of Southeast Asian and Caribbean Studies/KITLV, Leiden, the Netherlands; 39 Leiden University, Leiden, the Netherlands; 40 School of Public Policy and Global Affairs, University of British Columbia, Vancouver, British Columbia Canada; 41 Cameroon Country Office, World Food Programme, Yaoundé, Cameroon

**Keywords:** Agriculture, Environmental impact, Malnutrition, Economics, Sustainability

## Abstract

This Analysis presents a recently developed food system indicator framework and holistic monitoring architecture to track food system transformation towards global development, health and sustainability goals. Five themes are considered: (1) diets, nutrition and health; (2) environment, natural resources and production; (3) livelihoods, poverty and equity; (4) governance; and (5) resilience. Each theme is divided into three to five indicator domains, and indicators were selected to reflect each domain through a consultative process. In total, 50 indicators were selected, with at least one indicator available for every domain. Harmonized data of these 50 indicators provide a baseline assessment of the world’s food systems. We show that every country can claim positive outcomes in some parts of food systems, but none are among the highest ranked across all domains. Furthermore, some indicators are independent of national income, and each highlights a specific aspiration for healthy, sustainable and just food systems. The Food Systems Countdown Initiative will track food systems annually to 2030, amending the framework as new indicators or better data emerge.

## Abstract (web summary)

Holistic indicator frameworks are needed to track food systems transformation. This Analysis shows the application of a framework recently developed by the Food Systems Countdown Initiative to all UN member states, revealing current status, data gaps and priority actions.

## Main

Food systems fundamentally shape lives, well-being and human and planetary health, and they are central to tackling some of the most pressing global challenges of our time^1^. The United Nations (UN) held its first-ever Food Systems Summit (UNFSS) in 2021, which demonstrated the interconnectedness of food systems with the Sustainable Development Goals (SDGs) and provided a space for countries to develop national pathways towards food system transformation. Food systems also featured prominently at the 26th and 27th UN Climate Change Conference^2^ and in the Kunming-Montreal Global Biodiversity Framework targets^3^. This context offers growing momentum to influence public policy, private sector and civil society actions to transform food systems from their current unsustainable and inequitable trajectories to a healthier, more equitable, sustainable and resilient future^4–6^. Rapidly progressing towards the 2030 expiration of the SDGs and amid mounting social, political, health and ecological challenges, transforming food systems to support healthy diets in sustainable, resilient, just and equitable ways is more urgent than ever^1,7,8^. Yet while the contributions of food systems to global goals are recognized and the clear need for monitoring has been articulated^9^, decision-makers across sectors lack a means to assess their food systems, guide action or evaluate progress. Furthermore, without monitoring, bright spots and success stories go unrecognized when they can offer important lessons for other places.

In 2021, the Food Systems Countdown to 2030 Initiative (FSCI) emerged from the UNFSS as an interdisciplinary collaboration of dozens of scientists with the ambition to fill this monitoring gap. The authors first published the conceptual foundation in which they described the goal of food system transformation as “a future where all people have access to healthy diets, produced in sustainable, resilient ways that restore nature and deliver just and equitable livelihoods”^1^. They developed a monitoring architecture comprising five thematic areas, each with three to five indicator domains^1^. Building on the architecture, this paper presents the indicator selection process and the resulting indicator framework and global food systems baseline. To select indicators that capture all elements of the architecture, we surveyed additional scientific experts and conducted consultations with hundreds of policy stakeholders in a multi-stage indicator selection process. The process was restricted to existing indicators—or feasible modifications thereof—and aimed to align with other indicator frameworks, such as the SDGs, where sensible.

The consultative process selected 50 indicators and identified several data gaps, of which many are expected to be fillable in the near term (before 2030). We applied the 50-indicator framework to provide a harmonized baseline dataset as an initial descriptive analysis of the world’s food systems, the starting point to track change and an essential first step in a global food systems research agenda. For the next seven years (2023–2030), the FSCI will publish annual updates, incorporate new indicators to fill the remaining gaps and carry out further analyses. Specifically, in the next two years, publications will concentrate on understanding country-level performance and the dynamic interactions across indicators, domains and themes.

The fundamental contributions of this paper are (1) an application of the recently developed global architecture to monitor food systems^1^, (2) the selection of a set of indicators legitimated through consultative process, (3) the identification of the most critical data and information gaps for global food systems monitoring and (4) a harmonized baseline dataset to track food systems and their changes. These contributions are relevant to the government officials responsible for developing food system transformation pathways coming out of the UNFSS, who have expressed clear demand for guidance on indicators^10–13^. African countries are working to adapt the Comprehensive African Agricultural Development Programme to incorporate a broader food systems perspective, also requiring additional indicators for the Biennial Review process^14,15^. The intent is not to create another set of indicators that countries have to track but rather to offer a menu that can be useful for the food system transformation goals that countries are establishing, providing a mechanism for accountability to stated commitments where existing suites of indicators (for example, SDGs) are insufficient for food systems (Supplementary Fig. 1.1 contains the theory of transformation)^16^. Basing the framework on feasibility, existing indicators and available data lends further practicality and usefulness to leaders acting now. At the global level, the framework enables policymakers, advisors, private sector actors and civil society actors to monitor food systems worldwide.

## Results

### Indicator selection

**Fig. 1:**
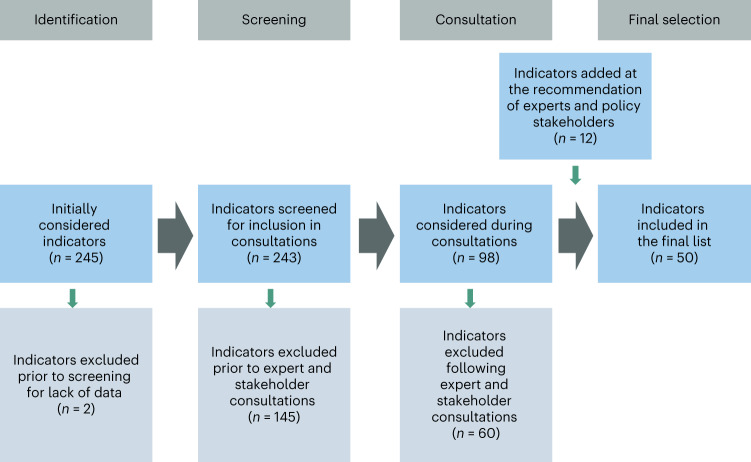
Multi-stage indicator selection process. The process of indicator selection and the number of indicators included and excluded at each stage. The excluded indicators are listed in Supplementary Appendix 2.

We employed a multi-stage, multi-stakeholder process to select the suite of indicators for food systems monitoring. In the first stage, we developed a long list of all possible indicators. This list was screened for feasibility, coverage and transparency (defined in Extended Data Table 2). The result was a shorter list of candidate indicators to be evaluated against the criteria of relevance, high quality, interpretability and usefulness (the operational definitions are provided in Extended Data Table 2). In stage two, a survey was fielded to all authors and additional experts to quantitatively score the indicators against the criteria and identify any alternative indicators or data sources and indicator gaps. Qualitative consultations were held with over 500 policy stakeholders across the world focused on gathering input on usefulness and gaps. In the final stage, we examined the indicator scores, additional suggestions to address gaps, and gaps that could not be filled to identify the list of 50 indicators presented in this baseline. Figure 1 presents the flow of indicators through the selection process, and full reports of the survey results and policy consultations are provided in Supplementary Appendix 3.

Table 1 presents the indicators and their global distributions, while Extended Data Table 1 contains the definitions, sources, rationale for inclusion, coverage and notable limitations. Many indicators have long time series available, while those without are expected to be collected or computed globally going forward and therefore are applicable for monitoring. Given our objective to work with existing data, there are limitations to these indicators, with several serving as imperfect proxies given data availability to be replaced as improved data and indicators are available (further details are given in Extended Data Table 1).

**Table 1:** Indicator list and global baseline^a^ distributions

Domain		Indicator		Unit	Worst ranking^b^	Best ranking^b^	Distribution^c^	Median	Weighted mean	Weighted s.d.	Weighted by
**Diets, nutrition and health**											
Food environments	1	Cost of a healthy diet		Current PPP US$ per person per day	Jamaica, Japan, Grenada, Suriname, South Korea	United Kingdom, Democratic Republic of the Congo, Belize, Ireland, Senegal		3.4	3.3	0.6	Population
	2	Availability of fruits and vegetables	Fruits	Grams per capita per day	Burkina Faso, the Gambia, Chad, Zambia, Togo	Dominica, Dominican Republic, Papua New Guinea, Sao Tome and Principe, Ghana		201.3	223.8	145.8	(Unweighted)
			Vegetables	Grams per capita per day	Chad, Democratic Republic of Congo, Comoros, Solomon Islands, Ethiopia	China, Albania, Guyana, Croatia, Tunisia		210.0	246.8	186.5	(Unweighted)
	3	Retail value (total sales) of ultra-processed foods		Current (nominal) US$ per capita per year	Norway, Finland, Ireland, Japan, Denmark	Mozambique, Uganda, Yemen, Burundi, Somalia		163.7	204.0	293.1	Population
	4	Percentage of population using safely managed drinking water services (SDG 6.1.1)		Percentage of population	Chad, Central African Republic, Sierra Leone, Rwanda, Ethiopia	Belgium, Canada, Cyprus, Germany, Spain		85.7	66.3	30.9	Population
	5	Prevalence of undernourishment (SDG 2.1.1)		Percentage of population	Central African Republic, Madagascar, Haiti, North Korea, Yemen	Australia, Austria, Azerbaijan, Belgium, Bosnia and Herzegovina		5.6	9.4	8.9	Population
Food security	6	Percentage of population experiencing moderate or severe food insecurity (SDG 2.1.2)		Percentage of population	Congo, Sierra Leone, South Sudan, Haiti, Central African Republic	Switzerland, Kazakhstan, Luxembourg, Austria, Germany		26.5	29.5	23.0	Population
	7	Percentage of population who cannot afford a healthy diet		Percentage of population	Burundi, Madagascar, Liberia, Malawi, Nigeria	United Arab Emirates, Azerbaijan, Switzerland, Iceland, Finland		21.4	42.3	33.9	Population
Diet quality	8	MDD-W: minimum dietary diversity for women		Percentage of population, women 15–49	Tanzania, Burkina Faso, Sierra Leone, India, Benin	Viet Nam, Kazakhstan, Bolivia, Tajikistan, China		71.7	65.7	20.3	Population
	9	MDD (IYCF): minimum dietary diversity for infants and young children		Percentage of population, 6–23 months	Guinea-Bissau, Liberia, Kiribati, Ethiopia, Congo	Serbia, Peru, Sri Lanka, Costa Rica, El Salvador		34.4	31.8	15.9	Population
	10	All-5: consumption of all five food groups		Percentage of adult population (≥15 yr)	Burkina Faso, Sierra Leone, Tanzania, Ghana, Cambodia	Tajikistan, Indonesia, Sri Lanka, Mexico, China		30.5	39.0	13.7	Population
	11 12	Zero fruit or vegetable consumption	Adults	Percentage of adult population (≥15 yr)	India, Tanzania, Nigeria, Benin, Sierra Leone	Israel, Tajikistan, Bolivia, Viet Nam, Chile		8.4	10.8	7.9	Population
			Children 6–23 months	Percentage of population, 6–23 months	Ethiopia, Guinea-Bissau, Sudan, Yemen, Guinea	Serbia, Belarus, Uruguay, Peru, Burundi		31.5	39.1	15.8	Population
	13	NCD-Protect		Score (points out of 9)	Sierra Leone, Nigeria, Gabon, Burkina Faso, Jordan	Mexico, Bolivia, Indonesia, China, Viet Nam		3.5	3.8	0.7	Population
	14	NCD-Risk		Score (points out of 9)	Kazakhstan, Chile, United States, South Africa, Philippines	Sierra Leone, Tanzania, Burkina Faso, Lebanon, Benin		2.0	2.1	0.7	Population
	15	Sugar-sweetened soft drink consumption		Percentage of adult population (≥15 yr)	South Africa, Nicaragua, Israel, Chile, Jordan	Sri Lanka, Indonesia, Benin, Bangladesh, China		24.1	18.9	10.6	Population
**Environment, natural resources and production**											
Greenhouse gas emissions	16	Food systems greenhouse gas emissions		ktCO_2_e (AR5)	China, India, Brazil, United States, Indonesia	Monaco, San Marino, Liechtenstein, Nauru, Tuvalu		18,626.2	82,463.9	226,713.0	(Unweighted)
	17	Greenhouse gas emissions intensity, by product group^d^	Cereals (excluding rice)^e^	kg CO_2_e per kg product	Suriname, Mauritius, Fiji, Guyana, Cabo Verde	Antigua and Barbuda, Djibouti, Nauru, Saint Vincent and the Grenadines, Micronesia		0.2	0.2	0.1	Area harvested
			Beef	kg CO_2_e per kg product	Timor-Leste, Madagascar, Niger, Lesotho, Mali	Brunei Darussalam, Mauritius, Lebanon, Jordan, Israel		37.3	30.3	28.2	Animals slaughtered
			Cow’s milk	kg CO_2_e per kg product	Papua New Guinea, Vanuatu, Côte d’Ivoire, Lao People’s Democratic Republic, Cambodia	Israel, Saudi Arabia, Cyprus, Jordan, Kuwait		1.4	1.0	1.0	Producing animals
			Rice	kg CO_2_e per kg product	Jamaica, Algeria, Mauritius, South Africa, Hungary	Panama, Nicaragua, Togo, El Salvador, Benin		1.6	1.1	0.6	Area harvested
Production	18	Food product yield, by food group^d^	Cereals^e^	t ha^−1^	Cabo Verde, Namibia, Sudan, Somalia, Niger	Saint Vincent and the Grenadines, United Arab Emirates, Oman, Kuwait, Mauritius		3.3	4.1	2.1	Area harvested
			Fruit^e^	t ha^−1^	Mongolia, Estonia, Tonga, Mauritania, Micronesia	Netherlands, Honduras, Costa Rica, Belgium, Kuwait		10.3	13.7	5.1	Area harvested
			Beef	kg per animal	Bangladesh, Georgia, Yemen, Nepal, Rwanda	Iran, Japan, Singapore, Malaysia, Canada		188.4	231.5	95.1	Animals slaughtered
			Cow’s milk	kg per animal	Papua New Guinea, Côte d’Ivoire, Burkina Faso, Ghana, Mali	Israel, Saudi Arabia, USA, Estonia, Denmark		1,537.4	2,676.6	2,713.3	Producing animals
			Vegetables^e^	t ha^−1^	Samoa, Maldives, Brunei Darussalam, Timor-Leste, Guinea	Kuwait, Iceland, Bahrain, Netherlands, Guyana		13.9	19.7	9.1	Area harvested
Land	19	Cropland expansion (per cent change)		Percentage	Lesotho, Colombia, Oman, Israel, Qatar	Samoa, Lao People’s Democratic Republic, Uruguay, Bhutan, Barbados		0.0	0.1	1.2	Cropland^f^
Water	20	Agriculture water withdrawal as percentage of total renewable water resources		Percentage of total renewable	Kuwait, United Arab Emirates, Saudi Arabia, Libya, Qatar	Cyprus, Maldives, Saint Vincent and the Grenadines, Papua New Guinea, Iceland		1.9	16.9	52.6	Cropland
Biosphere integrity	21	Functional integrity: percentage of agricultural land with minimum level of natural habitat		Percentage of agricultural land	Republic of Moldova, Bangladesh, Ukraine, Kiribati, Nauru	Andorra, Dominica, Grenada, Iceland, Palau		93.4	88.3	13.9	Agricultural land^g^
	22	Fishery health index progress score		Index	Djibouti, Mozambique, Eritrea, Viet Nam, Myanmar	Latvia, Peru, Norway, Iceland, United States		22.3	21.4	12.8	Population
Pollution	23	Total pesticides per unit of cropland		kg active ingredient per ha	Saint Lucia, Maldives, Oman, Israel, Ecuador	Congo, Comoros, Mali, Niger, Tanzania		1.4	1.8	1.9	Cropland
	24	Sustainable nitrogen management index		Index	Serbia, Romania, Argentina, Paraguay, Ukraine	Iceland, Botswana, Brunei Darussalam, Bahrain, Comoros		0.9	0.7	0.2	Cropland
**Livelihoods, poverty and equity**											
Poverty and income	25	Share of agriculture in GDP		Percentage of GDP	Sierra Leone, Liberia, Niger, Mali, Ethiopia	San Marino, Singapore, Liechtenstein, Luxembourg, Qatar		7.9	4.4	5.2	GDP
Employment	26	Unemployment, rural		Percentage of working-age population	South Africa, Lesotho, Eswatini, Djibouti, Botswana	Qatar, Niger, Cambodia, Rwanda, Solomon Islands		4.9	5.7	4.1	Population
	27	Underemployment rate, rural		Percentage of working-age population	Ethiopia, Honduras, Nicaragua, Nigeria, Belize	Egypt, Jordan, Timor-Leste, Senegal, Sierra Leone		4.4	7.3	8.2	Population
Social protection	28	Social protection coverage		Percentage of population	Bhutan, Uganda, Tonga, Mali, Solomon Islands	India, Mongolia, Chile, Hungary, Slovakia		40.8	55.8	28.0	Population
	29	Social protection adequacy		Percentage of welfare of beneficiary households	Papua New Guinea, Sudan, South Sudan, Sierra Leone, Azerbaijan	Congo, Poland, Serbia, Romania, Belarus		23.3	21.0	15.1	Population
Rights	30	Percentage of children 5–17 engaged in child labour		Percentage of children 5–17 (sex specific is percentage of children 5–17 of each sex)	Ethiopia, Burkina Faso, Cameroon, Togo, Chad	Turkmenistan, Trinidad and Tobago, Sri Lanka, Philippines, Barbados		9.0	9.4	9.6	Population
	31	Female share of landholdings		Percentage of landholdings by sex of operator	Lao People’s Democratic Republic, Bangladesh, Mali, Fiji, Egypt	Cabo Verde, Lithuania, Latvia, Eswatini, Moldova		18.7	16.8	8.3	Land area
**Governance**											
Shared vision and strategic planning	32	Civil society participation index		Index	North Korea, Eritrea, Turkmenistan, Cuba, Syrian Arab Republic	Denmark, United States, Germany, Norway, Finland		0.7	0.6	0.2	Population
	33	Percentage of urban population living in cities signed on to the MUFPP^h^		Percentage of urban population	Afghanistan, Andorra, Armenia, Antigua and Barbuda, Azerbaijan	Latvia, Mongolia, Argentina, Peru, Congo		0.0	7.2	10.4	Urban population
	34	Degree of legal recognition of the right to food (1, explicit protection or directive principle of state policy; 2, other implicit or national codification of international obligations or relevant provisions; 3, none)		Categorical				2.0	1.9	0.6	(Unweighted)
	35	Presence of a national food system transformation pathway (0, no; 1, yes)		Binary				1.0	0.6	0.5	(Unweighted)
Effective implementation	36	Government effectiveness index		Index	South Sudan, Yemen, Somalia, Haiti, Libya	Singapore, Switzerland, Finland, Norway, Denmark		−0.1	0.1	0.8	Population
	37	International Health Regulations State Party Assessment report (IHR SPAR), food safety capacity		Score	Central African Republic, Côte d’Ivoire, Poland, Afghanistan, Bolivia	United Arab Emirates, Australia, Austria, Belgium, Bahrain		80.0	69.4	21.6	Population
	38	Presence of health-related food taxes^h^		Binary				0.0	0.3	0.5	Population
	39	V-Dem Accountability Index		Index	Eritrea, North Korea, Syrian Arab Republic, Turkmenistan, Saudi Arabia	Denmark, Sweden, Norway, Costa Rica, Estonia		0.7	0.3	0.9	Population
Accountability	40	Open Budget Index score		Index	Comoros, Equatorial Guinea, Venezuela, Yemen, Sudan	Georgia, South Africa, New Zealand, Sweden, Mexico		46.0	43.1	21.3	Population
	41	Guarantees for public access to information (SDG 16.10.2)		Binary				1.0	0.7	0.5	Population
**Resilience**											
Exposure to shocks	42	Ratio of total damages of all disasters to GDP		Ratio	Dominica, Saint Vincent and the Grenadines, Bahamas, Tonga, Antigua and Barbuda	Afghanistan, Angola, Albania, United Arab Emirates, Argentina		0.0	0.3	0.8	GDP
	43	Dietary sourcing flexibility index		Index	Comoros, Seychelles, Kiribati, Cambodia, Eswatini	Netherlands, Belgium, Italy, Portugal, Switzerland		0.7	0.7	0.1	Population
Resilience capacities	44	Mobile cellular subscriptions (per 100 people)		Number per 100 people	South Sudan, Micronesia, North Korea, Marshall Islands, Liberia	Antigua and Barbuda, Seychelles, United Arab Emirates, Montenegro, Thailand		108.8	105.5	35.0	(Unweighted)
	45	Social capital index		Index	Lebanon, Zimbabwe, Brazil, Central African Republic, Nicaragua	Norway, Finland, New Zealand, Switzerland, Netherlands		0.4	0.5	0.2	Population
Agrodiversity and food diversity	46	Proportion of agricultural land with minimum level of species diversity (crop and pasture)^h^		Percentage of agricultural land	Albania, Andorra, United Arab Emirates, Armenia, Antigua and Barbuda	Grenada, Saint Vincent and the Grenadines, Jamaica, Ukraine, Haiti		14.1	22.5	23.6	Agricultural land^i^
	47	Number of (a) plant and (b) animal genetic resources for food and agriculture secured in either medium- or long-term conservation facilities (SDG 2.5.1)	Plants	Thousands	Malta, Honduras, Mauritania, Suriname, Guinea	United Kingdom, United States, India, Australia, Japan		7.0	161.4	174.5	Land area
			Animals	Number	Argentina, Azerbaijan, Burundi, Benin, Burkina Faso	Spain, India, Portugal, Republic of Korea, Norway		0.0	4.4	8.8	Land area
Resilience responses/ strategies	48	Coping strategies index		Percentage of population	Zimbabwe, Afghanistan, Yemen, Central African Republic, Syrian Arab Republic	Iraq, Tanzania, El Salvador, Burkina Faso, Nicaragua		39.0	38.5	12.7	Population
Long-term outcomes	49	Food price volatility^h^		Unitless	Côte d’Ivoire, Austria, Guinea-Bissau, Equatorial Guinea, San Marino	Kiribati, Democratic Republic of the Congo, Micronesia, Eswatini, Djibouti		0.7	0.7	0.3	(Unweighted)
	50	Food supply variability		kcal per capita per day	Benin, Republic of Moldova, Trinidad and Tobago, Viet Nam, Sweden	Lesotho, Venezuela, Central African Republic, Montenegro, Zimbabwe		27.0	29.9	17.2	(Unweighted)

Source: Our calculations based on the data sources listed in Extended Data Table 1 (the year is the latest data point per country per indicator). Each indicator includes a maximum of all UN member states as of August 2022; the country list differs per indicator given data availability (Supplementary Figs. 1.3–1.11).

^a^Baseline data comprise the latest available data point per country–indicator. The latest data point per country–indicator differs given data availability and is reported in Supplementary Data 1; 92.5% of data points are from 2017–2022, 6.5% are from 2010–2016 and only 1% are from 2000–2009.

^b^The best and worst rankings are the top and bottom countries in an ordered list for each variable. Where higher is more desirable, the highest value is ranked 1. Where lower is more desirable, the lowest value is ranked 1. Ranking does not incorporate any weighting. Binary and categorical indicators are not ranked. Countries with no data for an indicator are not ranked on that indicator. The top and bottom countries reflect outliers by definition and should not be generalized as exemplars without further contextualization. Under the environment domain, some outliers have very little agricultural production. We also note the politicized nature of consumer food price indices on which the food price volatility indicator is based.

^c^Frequency histograms displayed.

^d^Additional products are included in Supplementary Appendix 1 and in the baseline dataset (Supplementary Data 2).

^e^The product mix varies across countries.

^f^The cropland variable used for weighted means comes from the FAOSTAT database and adheres to the FAO cropland classification as described in Extended Data Table 1.

^g^Weighted by agricultural land in 2015 in concordance with the only available year of data for this indicator.

^h^Indicates FSCI value-added to existing data.

^i^Weighted by agricultural land in 2010 in concordance with the only available year of data for this indicator.

PPP, purchasing power parity; NCD, non-communicable disease; CO_2_e, CO_2_-equivalent emissions.

#### Diets, nutrition and health

Supporting human health is one of the three fundamental goals of food systems. The three indicator domains in this theme are food environments (the interface between individuals and the food system), food security and diet quality. One important aspect of food environments is the availability of different kinds of foods, reflected by the availability of fruits and vegetables and per capita sales of ultra-processed^17^ foods. Access to sufficient, safe, nutritious food and clean water is a core piece of food systems monitoring. Access to food is in part determined by the cost of a healthy diet—that is, the cost of purchasing the least expensive locally available foods to meet requirements for energy and food-based dietary guidelines. The affordability of that diet (cost relative to income) is one of three food security indicators alongside the prevalence of undernourishment and the percentage of the population experiencing moderate or severe food insecurity. Access to clean water is essential for avoiding food-borne and water-borne illnesses. No adequate available indicators exist for food safety, a priority data gap. Diet quality indicators capture what individuals actually eat, and they reflect diversity, adequacy and moderation. Indicators include minimum dietary diversity for women and children, consumption of the five food groups typically recommended for daily consumption in food-based dietary guidelines (fruits; vegetables; pulses, nuts or seeds; animal-source foods; and starchy staples), dietary factors that either protect against or increase risk for non-communicable diseases, and unhealthy dietary practices over the life cycle, aligned with international guidance^18–20^.

#### Environment, food production and natural resources

Food systems are a major contributor to environmental degradation, but they can also protect and restore environmental outcomes if managed appropriately. The six domains of environmental indicators address the multiple environmental impacts of food systems: greenhouse gas emissions, land, biosphere integrity, water, pollution (conceptually including nutrient runoff, chemical exposure and solid waste) and agricultural production, which interacts with all other domains.

Indicators of greenhouse gas emissions include total emissions (from production through consumption and waste disposal) and emissions intensities (emissions per unit of primary product) of major foods. Land use change is measured by cropland expansion and water use, expressed by how much agricultural water withdrawals place pressure on renewable freshwater resources. Overuse of pesticides and sustainable nitrogen management capture pollution; additional indicators of solid waste and chemical pollution attributable to food systems are wanting. Functional integrity—the capacity for biodiversity to support sustainable food production and other ecosystem services—and the integrity of fishery stocks capture biosphere integrity. Yields interact with all other domains; increases are directly tied to the observed declining trends in emissions intensities.

#### Livelihoods, poverty and equity

Poverty is most prevalent in rural areas where people earn substantial income shares from agriculture (including marginalized groups such as Indigenous Peoples and female-headed households)^21–23^. Food systems provide employment for 1.23 billion people and (including household members) support over 3.83 billion livelihoods, in all stages of the value chain across rural and urban areas^24^. Four indicator domains capture their well-being: income and poverty, employment, social protection and rights. Compared with other themes, the available data are more limited due in large part to lack of disaggregation to distinguish food system livelihoods from others.

Lacking a rural poverty indicator with sufficient coverage, the share of gross domestic product (GDP) from agriculture provides a proxy for a country’s overall level of development^25^. Declining GDP from agriculture and fewer people working in agriculture are hallmarks of the structural transformation process that is integral to poverty reduction and rural transformation^25^. Unemployment and underemployment capture employment, though not ‘decent’ work^26^. Though lacking sectoral disaggregation, the rural rates proxy the status of agricultural and farm-related labour markets^27^. Social protection systems increase access to food quantity and quality, reduce producers’ risk and incentivize productive investment^28,29^. Social protection programmes may be particularly impactful in breaking the cycle of poverty for small-scale food producers and informal workers who face chronic food insecurity and vulnerability to shocks^29^. Finally, among the many rights and issues of justice related to livelihoods, the indicators currently available capture women’s access to land and the specific human rights violation of child labour, of which an estimated 70% occurs in agriculture^30^.

#### Governance

Governance is foundational for inclusive food system transformation, encompassing not only the political commitment to adopt supportive policies but also promoting participatory processes and accountability to ensure that policies have legitimacy and reach the intended target group. Furthermore, governance involves strengthening capacities for implementation across sectors to ensure that aspirational goals are technically feasible. Three indicator domains collectively capture these dimensions of governance: shared vision and strategic planning, effective implementation and accountability. There are few indicators of governance specific to food systems, but broad indices of the governance landscape may have substantial impacts on food system choices and outcomes. Further research is especially needed in this area to develop more direct indicators of food system governance.

Indicators of shared vision and strategic planning include one broad indicator beyond food systems and three others reflecting intentionality by governments to pursue food systems objectives. The Civil Society Participation Index captures whether civil society organizations (for example, non-governmental organizations, unions and social movements) have opportunities to convey their views to policymakers. Food-system-specific indicators are the presence of a legal recognition of the right to food, the existence of a food system transformation pathway and the share of the urban population living in cities that have signed on to the Milan Urban Food Policy Pact (MUFPP). The MUFPP is an innovative policy mechanism that has rapidly become the leading international tool for urban food policy governance (37 recommended actions and specific indicators) as well as a platform for cooperation, organizing and political influence^31^.

Effective implementation is also measured by a combination of indicators that are contextual (broader than the food system but establish the governance regime within which food system actors can operate) and specific to food systems. The government effectiveness index reflects the quality of public services, civil service, policy formulation, implementation and credibility. Public tracking of investments for food systems requires transparency over budgets and guarantees for public information access, reflected in the Open Budget Index score and guarantees for public access to information, as well as the overall Accountability Index, which encompasses the existence of mechanisms to keep officials responsive to the public (for example, checks and balances, elections and press freedoms). Specific to food systems, available data can monitor two policy tools for achieving healthy food systems: health-related food taxes and food safety capacity (the number of specific mechanisms in place to detect and respond to food-borne disease and contamination).

#### Resilience

We define food system resilience as “the ability of different individual and institutional food system actors to maintain, protect, or quickly recover the key functions of that system despite the impacts of disturbances”^1^. The COVID-19 pandemic and the conflict in Ukraine both demonstrated the imperative to better understand and strengthen the resilience of local and global food systems to numerous shocks and stressors—not just climate change. Assessing resilience requires a combination of indicators related to two domains: (1) the contextual elements of resilience (the level of exposure of the system to adverse events and the capacities of that system to anticipate, absorb or adapt to those events) and (2) the short- and longer-term outcomes of resilience—generally measured through individual and system well-being, ideally considered at multiple scales^32^.

A range of indicators is necessary to capture these different components of resilience and to better understand how to establish more efficient, inclusive and sustainable food systems in the face of increasingly complex and intertwined shocks. The indicators of resilience therefore cover five domains: exposure to shocks, resilience capacities, agrodiversity and food diversity, short-term resilience responses and long-term outcomes.

Exposure to shocks depends on the intensity, nature and frequency of shocks and stressors and can be proxied by the cumulative costs of those events relative to GDP. Resilience capacities are the different elements that can be used to buffer and respond to adverse events. Those capacities take many forms. In food systems, the diversity and redundancy of food sources, national infrastructure (proxied by mobile phone coverage) and social capital are some of the key elements that constitute resilience capacities. Also critical to food system resilience is the level of biodiversity on which food production relies, captured by the number of plant and animal genetic resources conserved for use. Understanding how actors react and respond in the short term to the impact of shocks is also a foundational element of resilience analysis. This element can be measured using the coping strategies index, while longer-term outcomes of food system resilience can be captured by the ability of the system to maintain low price volatility and low food supply variability.

This resulting indicator framework partially overlaps with the SDGs, underscoring both the relevance of the overall development agenda for food system transformation and, conversely, the inadequacy (incompleteness) of the SDG framework for food systems monitoring^16^. Of the 240 SDG indicators, 81 are related to food systems and food system transformation. Only 11 are specific to food systems, and of those, only 5 meet the criteria for inclusion in the FSCI. The SDG indicators included are 2.1.1 (undernourishment), 2.1.2 (food insecurity), 6.1.1 (safe drinking water), 16.10.2 (access to information) and 2.5.1 (conserved genetic resources). Three SDG indicators are expected to be added as soon as data become available, including 2.4.1 (sustainable agriculture) and 12.3.1 (food loss and waste indices). Similarly, 5.a.1 (women’s agricultural land ownership) will replace the current data source (which will no longer be updated), and 5.b.1 (mobile phone ownership) will replace the current indicator of phones per 100,000 people, as soon as there is sufficient country coverage. Supplementary Table 4.1 documents which SDG indicators are relevant to food systems, the subset of those that are included in the FSCI framework and an explanation for why the others are not included.

### Data gaps

Notable data gaps emerged through the indicator selection process. Several gaps cut across multiple themes such as the true cost of food, a cost that includes the externalities currently unaccounted for in the market price such as diet-related disease, pollution and natural resource degradation^33^. Similarly lacking are data on food loss and waste at the country level, and we await country-level data of sufficient quality for SDG 12.3.1 (food waste and food loss indices).

In the realm of diets, nutrition and health, food safety is an area lacking indicators (and data), though food safety capacity is captured in governance. Under environment, natural resources and production, a gap regarding sustainable agriculture will be filled with SDG 2.4.1 (agricultural area under sustainable management practices) when data for the recently developed indicator are available^34^. Other gaps include food production and supply indicators inclusive of aquatic and wild foods. Furthermore, environmental indicators predominantly relate to production and largely exclude loss and waste as well as pollution related to processes further down the value chain (for example, solid waste and material pollution from packaging^35^). Many gaps exist with respect to livelihoods, including the economic value of food systems, the magnitude and composition of populations working in food systems and their vulnerabilities, and productivity in the sector (for example, value-added as a share of GDP and per worker). In addition, indicators of livelihoods that can capture the welfare of food system workers beyond agriculture—especially measures of decent work, gender equity and violations of human rights in food systems—are needed. With respect to food system governance, data gaps include policy coherence (alignment across policy areas) for food system transformation and budgetary allocations to food systems. These gaps require substantial country-level data inputs to fill, but new machine learning methods may provide opportunities to develop estimates that can be added to the indicator suite in the near term. Additional indicators of governance and resilience specific to food systems are also lacking.

**Fig. 2:**
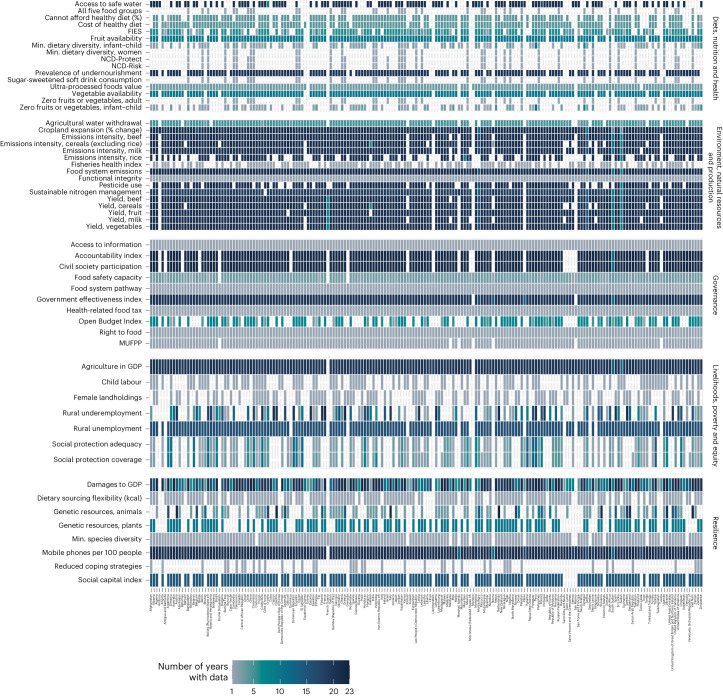
Data coverage, number of years per country–indicator, 2000–2021. Heat map illustrating the density of data points per country–indicator pairing, with the darkest cells illustrating more years of data between 2000 and 2021. Indicators with no data at all for that country are shown in white. The figure illustrates greater availability of data for food security and agricultural indicators and lesser availability for indicators of diet quality, livelihoods and resilience. Heat maps showing the indicator–country time series by region are available in Supplementary Figs. 1.3–1.11. The maximum country coverage is all UN member states, but coverage differs per indicator depending on data availability. Differences in indicator coverage largely drive the observed differences across countries. Specifically, the indicators with the most heterogeneous coverage are the six indicators of diet quality sourced from the Global Diet Quality Project (currently available for only 41 mostly low- and lower-middle-income countries); the livelihood indicators of employment, social protection, child labour and landholdings; and the resilience indicators of genetic resources and coping strategies (available for countries with a high prevalence of food insecurity). Looking across countries within each indicator, countries with the indicator typically have time series of similar durations. Yield and emissions intensity for additional products are provided in Supplementary Appendix 1 and the baseline dataset. FIES, Food Insecurity Experience Scale.

Gaps also pertain to the country and time series coverage of indicators. Figure 2 presents a data coverage heat map from 2000 forward showing that the indicators with the greatest country coverage and the longest time series are those associated with agricultural development such as yields and the share of agriculture in GDP. For other indicators—adult diet quality, biodiversity, and agrodiversity and food diversity—the country and year coverage remain sparse. Country coverage of diet quality indicators is expected to increase rapidly, but there are no adult diet quality data for any countries in Oceania, and there are data for only one country in the Caribbean—a priority gap, given the high burden of diet-related disease in these regions^18,36,37^. Environmental indicators have the greatest coverage, partly because so many derive from FAOSTAT indicators with a long history of collection^38^. Governance indicators also have good country coverage, but one third of the indicators in this theme are newly developed (right to food, presence of a food system pathway and urban population signed on to the MUFPP). Livelihood and resilience indicators have poorer geographic coverage across most regions, especially Oceania and northern Africa and western Asia.

By region (Supplementary Figs. 1.3–1.11), Oceania has the greatest scarcity in data overall, with very few diet quality indicators collected in that region and only for children in 4 of the 14 countries. Dietary data are collected in fewer countries of North America and Europe, northern Africa and western Asia, and Latin America and the Caribbean than in other regions. Countries with the fewest indicator and year observations are small island nations (for example, Caribbean and Pacific islands), very small high-income countries (HICs) (for example, Brunei, Monaco and Singapore), several countries in the Middle East (such as Saudi Arabia and Qatar) and countries (recently) experiencing conflict (for example, Eritrea and Syria).

### Global baseline

**Fig. 3:**
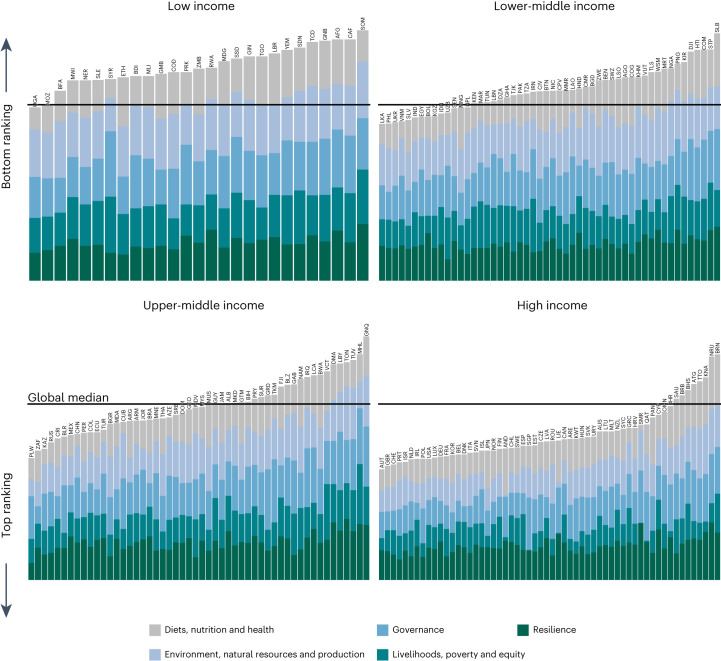
Average country ranking per theme, by country income level. The mean ranking of indicators within each theme shows that no country is performing in alignment with desirable outcomes for all themes. The bottom ranking indicates scores farthest from the minimum or maximum value observed across all countries, depending on whether lower or higher values are aligned to the desirable outcome. Countries are ranked per indicator relative to all other countries, and the average rank for all indicators within a theme is shown per country. Countries are grouped by income level and presented in order of increasing income from left to right and top to bottom. The horizontal black lines indicate the global median rank pooling all indicators. The results are from our calculations based on the data sources listed in Extended Data Table 1 and from the latest data point per country–indicator pair, of which the majority come from 2017–2021. Supplementary Data 1 contains the specific year for each country–indicator data point. Binary and categorical indicators are not ranked and are therefore excluded from the governance theme average. Country ranking per indicator is averaged at the theme level. Not all countries have data for every indicator. Missing data do not bias the total ranking visualized, but they do result in implicit weighting of the thematic mean rank by the present indicators. Country abbreviations shown as ISO alpha-3 country codes.

Table 1 presents the selected indicators and their global distributions in the most recent year for which data are available (the definitions, data sources, rationale for inclusion, key limitations and desirable direction of change are provided in Extended Data Table 1). In Table 1 (and Fig. 3), the best ranking and worst ranking reflect the ranking of all countries per indicator relative to the desirable direction of change, where the best (first ranking) is the highest value for indicators where higher is more desirable and the lowest for indicators where lower is more desirable. A lower rank indicates a better ranked position. The characterization is meant to be descriptive of the relative baseline starting points and is not intended as a performance assessment, which is a subsequent research agenda of the FSCI in the next two years. The baseline dataset includes the latest available data point per country–indicator, which differs given data availability. Most data (92.5%) are from 2017–2022, 6.5% are from 2010–2016 and only 1% are from 2000–2009. The specific year per country–indicator pair is reported in Supplementary Data 1, and the complete country-level dataset is in Supplementary Data 2. Several general patterns emerge from this global view, supported by descriptive analyses by region and income group for all indicators in Supplementary Figs. 1.1.1–1.5.17.

All food environment indicators suggest inequalities across countries: the availability of fruits and vegetables is generally a challenge in low- and middle-income countries, while HICs generally have widespread availability of ultra-processed foods. The cost of healthy diets is similar across most countries, but given wide differences in purchasing power, that cost is largely unaffordable across low- and middle-income countries.

Despite some improving trajectories, total food system emissions are increasing and remain high in HICs. Northern Africa and western and southern Asia remain at the greatest risk of exhausting available water resources. Only 88% of agricultural lands have the minimum of 10% functional integrity needed to support food production, meaning over one tenth of the world’s agricultural lands lack foundational ecosystem services such as crop pollination, pest regulation and soil protection, and other research suggests that the 10% threshold may be insufficient^39^.

The available data provide only a partial view of food-system-based livelihoods, but even the incomplete picture suggests deep inequalities. Important differences in unemployment and underemployment between rural and urban areas show that unemployment is prevalent in urban areas while underemployment is more prevalent in rural areas. Other evidence shows a larger gender gap in labour force participation in rural areas^40^. Even where there is adequate coverage of social protection programmes, the level of benefits provided may be insufficient to produce meaningful impacts, and informal and seasonal workers are often excluded^41–44^. Finally, access to land shows a stark gender disparity with no country approaching gender equality in landholdings.

The data show that indicators of overall governance track country income, while those more closely related to food systems show more heterogeneity across regions and income groups. For example, only 29 countries explicitly recognize the right to food, while the United States, Canada, the United Kingdom and Australia notably have no degree of legal recognition. In addition, health-related food taxes exist in 38 countries spread across all continents.

Looking across resilience indicators for a sub-group of countries (Supplementary Fig. 1.5.17), the data show that some countries (for example, the Philippines, Nicaragua and Indonesia) demonstrate relatively higher food price volatility or food supply variability than others (for example, the Netherlands, Thailand and India). These are countries facing higher exposure and/or lower resilience capacities (such as Nicaragua and Ecuador), showing that they also fare worse in their food system outcomes than those less exposed to shocks and/or characterized by higher social capital and dietary sourcing flexibility (such as Thailand and the Netherlands). However, this trend displays important variability, reflecting the specificity in how shocks propagate through a country’s food system, and calls for more in-depth analyses such as the future work planned to focus on interactions and dynamics of change across food systems.

Many aspects of food systems are associated with country income level^25^, raising questions of which indicators evade income trends and whether there are inflection points by income that might help countries set priorities. Figure 3 presents the country-level mean ranking per theme, grouped by country income level (for grouping by region, see Supplementary Figs. 1.12–1.20). The results illustrate that within every income group, there are some countries performing better than others. Even among the lowest-income countries, Uganda and Mozambique rank near the global median across all indicators, while on the other end of the spectrum, despite their high-income status, several countries rank worse on average than countries with many fewer resources. This analysis begins to suggest which countries might have useful lessons for others, especially those non-HICs outranking their income-group peers such as Uganda, Mozambique, Sri Lanka, the Philippines, Nigeria and Kazakhstan.

**Fig. 4:**
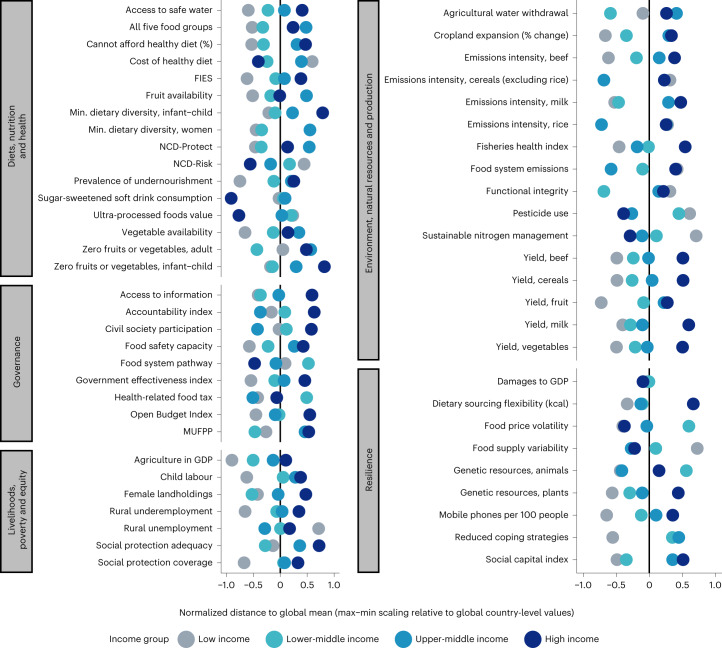
Average country ranking per theme, by country income level. Normalized difference between each income group mean value per indicator and the global mean for that indicator (represented by the black vertical lines). Differences are aligned to the desirable direction of change such that points to the left of the global mean indicate that the indicator mean level is less desirable than the global mean and points to the right indicate values more desirable than the global mean. The results are from our calculations based on the data sources listed in Extended Data Table 1 and from the latest data point per country–indicator pair, of which the majority come from 2017–2021. Supplementary Data 1 contains the specific year for each country–indicator data point. The normalized distance to the global mean (weighted means following the weights defined in Table 1) is calculated relative to the global mean and scaled to the minimum and maximum of the income group mean, per indicator (the global mean is centred at 0). The sign of the normalized distance has been reversed for all indicators where a lower value is more desirable, such that −1 can be interpreted as ‘worse than’ and 1 can be interpreted as ‘better than’ the global mean. The number of people who cannot afford a healthy diet and the degree of legal recognition of the right to food are not shown. The product mixes in aggregate categories of emissions intensities (cereals) and yields (cereals, citrus, fruit, pulses, roots and tubers, and vegetables) differ across countries. Yield and emissions intensity for additional products are included in Supplementary Appendix 1 and the baseline dataset.

Looking at each indicator by country groupings, Fig. 4 shows the distribution of country income groups relative to the global average. The figure is aligned to the desirable direction of change (defined in Extended Data Table 1) such that to the right of the global mean is better. Additional analyses present the values displayed and test for statistically significant differences by country income group and by region (Supplementary Tables 1.1 and 1.4) and provide weighted means and medians by region and income group (Supplementary Tables 1.2, 1.3, 1.5 and 1.6). These analyses show that only the presence of a national food system pathway is not statistically significantly different by region, while numerous variables do not differ by country income group, including the cost of a healthy diet, the availability of fruits and vegetables, the minimum dietary diversity for women, food system emissions, cereal yields, cropland change, agricultural water withdrawals, functional integrity, rural underemployment, women’s share of landholdings, the presence of a food system transformation pathway, the Open Budget Index and mobile subscriptions.

Beyond country income level, understanding each indicator’s relationship to GDP per capita is useful for hypothesis generation. Supplementary Figs. 1.21–1.26 show the relationship between each (continuous) indicator and GDP per capita. Several indicators exhibit less obvious relationships to GDP, including the cost of a healthy diet, pesticide use and sustainable nitrogen management, yields for vegetables and roots and tubers (potentially reflecting different crop mixes), female landholdings, food price volatility, food supply variability and mobile phone subscriptions. These findings underscore the potential for policymakers and other actors to influence more desirable outcomes on at least some indicators of food systems even in low-income countries, and to identify where income seems to be a necessary driver (though alone probably insufficient) of more desirable outcomes.

## Discussion

The indicator framework presented in this paper allows progress across global food systems to be meaningfully tracked, complementing the SDGs and other indicator frameworks with a curated set of existing indicators to monitor food systems, selected through a consultative process. It provides the foundation for future research to better understand how and where change comes about, and importantly how to identify where improvements in any one domain do not necessarily translate into improvements in others^45,46^. Looking across this baseline, the indicators included offer a trove of information that provides transparency and specificity to the important constructs but does not prescribe obvious or uniform actions. Three clear messages emerge. First, no country, region or income group exhibits desirable status across all indicators. Second, not all food system indicators are aligned to country income level; there are a diversity of food system trajectories. And third, there are some critical data gaps to monitor the world’s food systems that must be filled in the near term to guide action in service of food system transformation, meeting the SDGs and ensuring that food systems positively contribute to the many global goals linked to food systems.

The FSCI effort is intended to complement other global goal setting and monitoring efforts such as the SDGs, through the lens of food systems, which have been only partially captured in existing goals, indicators and monitoring efforts. We aim for synergies with these internationally recognized goals, but the very small overlap between the SDGs and the FSCI framework reflects the fact that food systems were not yet considered a mainstream framing approach when the SDGs were developed. As food systems become more widely understood from a systems perspective, the large set of FSCI indicators that are not in the SDGs provides some guidance as to indicators that could be considered for the next set of global goals.

The process of indicator selection identified key data gaps—the specific information that needs to be collected at scale to achieve the ambitious goal of tracking and informing food system transformation. The gaps span all themes—for example, livelihood indicators beyond agriculture, food loss and waste, and governance of food systems. Many ongoing initiatives are working to fill some gaps (Supplementary Table 4.2), with notable achievements already in bringing data together (for example, the Food Systems Dashboard^47^). The baseline dataset provides a starting point for tracking, and the framework of indicators can be used by policymakers and other food system actors to diagnose their food systems and formulate appropriate responses, including transformation plans, and monitor advances in their countries. The baseline description demonstrates that no country shows positive outcomes across all dimensions. In addition, given that some food system outcomes are independent of national income levels, dedicated monitoring and transformation agendas specific to food systems are needed. Ongoing expansion of the FAOSTAT database and the Global Diet Quality Project will also help fill these gaps^18,48^. Other advances are dramatically reducing costs and increasing the quality and granularity of new data collection (for example, the 50×2030 Initiative)^49–52^.

This indicator framework was developed with usefulness to countries and other food system decision-makers as a driving purpose, but country-level testing and adaptation is warranted. Following the UNFSS process, at the time of latest analysis by the Food and Agriculture Organization (FAO), 123 governments had developed national food system transformation pathways^12^. The five domains of the FSCI architecture map closely to these pathways and will allow them to be well monitored with the indicators selected and presented here (Supplementary Table D.3). There is utility in tracking national progress relative to goals as well as relative progress within a region, by income peer group or in the world overall. In addition to meeting the information needs at a country level, the indicator framework is useful in addressing the supranational and transboundary issues within food systems that require alignment, coordination and goals at higher jurisdictional levels. Decision-makers can use the framework as a starting point to consider what changes in indicators are achievable at different scales and can forge coalitions to drive change. Furthermore, different actors may find certain indicators more useful for guiding action than others. For example, donors may be more concerned with cross-country comparisons when deciding how to allocate resources. National policymakers may be more interested in understanding how their country is doing over time on indicators under more direct national influence or control.

This baseline sets the stage, but future work is needed to close data gaps, assess status relative to benchmarks aligned to transformation, understand how food systems evolve over time (including interactions across different indicators that affect the sustainability of food systems overall) and better understand and take action to support the needs of national and global data users. The FSCI will undertake this research and action agenda in the coming years alongside regularly updated assessments tracking progress from this baseline forward, including the addition of new indicators or the refinement of the current set of indicators as food systems science progresses. By doing so, the FSCI aims to facilitate and accelerate food system transformation to deliver a healthier, more equitable, sustainable and resilient future for all.

## Methods

This paper has used the term ‘food systems’ throughout, in line with the UNFSS language. However, the indicator framework presented takes an expanded concept of agri-food systems given that many indicators cannot distinguish between food and non-food components of production and value addition, although such non-food components greatly influence the environment, social outcomes and the food people ultimately eat. Hence, food systems as used here encompass activities and processes around non-food agricultural products (for example, forestry, fibres and biofuels) that are interconnected with food for human consumption^1^.

A rigorous set of prerequisite criteria were established that all indicators had to meet to be considered at all for this work, which included feasibility (having recent data and being planned to be updated within the next eight years), coverage (at least 70 countries across regions and income levels) and transparency (no modelled indicators with undisclosed or untraceable methodologies). A comprehensive multi-stage, multi-stakeholder process was then conducted to select the list of indicators analysed in this paper (described in further detail below). Using a quantitative survey, dozens of experts were asked to rate each candidate indicator on its relevance, the quality of the data and methods, and its interpretability for policy purposes. Indicators assessed to be relevant, high quality and interpretable were considered to be useful, and a usefulness criterion was applied to the suite of indicators selected to monitor each domain to ensure sufficient but not redundant information. Finally, crucial input on regional priorities and policy utility provided by policy stakeholders was incorporated. Several indicators come from common sources such as FAOSTAT, the Gallup World Poll and the World Bank, but data from many other academic and non-governmental organization sources are also included. This replicable protocol including the survey and consultation processes culminated in our final selection of the indicators presented in this paper. All data and replication code are publicly available.

### Data

The data used in this paper were sourced from many global, publicly available data sources. Extended Data Table 1 provides the data source, description, rationale for inclusion and coverage metadata for each indicator as well as any notable limitations and mitigation actions to address them. Supplementary Data 1 provides an Excel spreadsheet containing the complete metadata, a codebook, country and year coverage, and the year of the latest data point per country–indicator that comprises the baseline. Supplementary Data 2 contains the complete baseline dataset of the latest data point per country per indicator used in the baseline analysis presented herein.

### Indicator selection

We employed a multi-stage, multi-stakeholder process to select the list of indicators analysed in this paper. A preliminary set of criteria was previously published in Fanzo et al.^1^. In the first stage of indicator selection, we refined these criteria by deeming three attributes to be essential: feasibility, coverage and transparency. Next, we refined the four criteria established previously: relevant, high quality, interpretable and useful. Extended Data Table 2 details the requirements, criteria definitions and sub-criteria.

Working group members compiled a list of candidate indicators for each domain that met the prerequisite requirements for potential inclusion. Supplementary Appendix 2 contains the indicator catalogue of all candidates, indicator options excluded for failure to meet the prerequisites and all relevant information that was provided to assess the indicators. This list of candidate indicators was assessed against the first three criteria (relevance, quality and interpretability) using an online survey by all the collaborators and an additional group of over two dozen external experts who were volunteer respondents based on a list of experts generated by all the authors with additional research to reach relevant people unknown to the author group. Everyone assessed indicators in the domain(s) aligned with their expertise. The respondents were asked to choose their level of agreement (from 1 to 5) with the statement that the candidate indicator met each sub-criterion, the elements in the bulleted lists in Extended Data Table 2. All respondents were also asked to state their agreement that the indicator is important for tracking food system transformation and to share their interpretation of both importance and transformation in that context, providing complementary qualitative data. Finally, the external experts were also asked to suggest additional data sources for candidate indicators and to describe any observed gaps in the domains and indicators and how they recommend filling those gaps. For those who assessed governance indicators, an additional question asked what new indicators the respondent deemed necessary and asked for recommendations for their construction. Supplementary Appendix 3 contains the full report of the survey procedures and outcomes, including all the scoring results. Figure 1 summarizes the flow of indicators through the process.

In parallel, the FAO convened five regional policy stakeholder consultations in Latin America and the Caribbean, sub-Saharan Africa, North Africa and the Middle East, Asia and the Pacific, and Europe. Over 500 people participated, averaging 75–100 per region. The consultations included a short overview presentation and breakout discussions of each thematic area. The participants were asked to assess the local pertinence of the architecture and indicator framework and to solicit regional priorities, interests and needs. Supplementary Appendix 3 contains the reports for each regional consultation. The consultation asked experts and stakeholders to suggest alternative indicators and data sources and to identify gaps, which resulted in the addition of several indicators to the initial list of candidates.

To identify the final list of indicators, scores from the assessment of indicators against the six sub-criteria of relevance, quality and interpretability criteria were summed to the indicator level with equal weighting, providing a single score per indicator. Usefulness was assessed qualitatively at the level of indicator domains, with emphasis on meeting the needs illuminated by the policy stakeholder workshops. Twelve indicators were added and ultimately included in the final set after the survey and consultations because they address gaps that were widely identified. These indicators are safe drinking water, agri-food system emissions, yields, share of agriculture in GDP, underemployment, degree of legal recognition of the right to food, percentage of the urban population living in a signatory municipality to the MUFPP, food safety capacity, health-related food taxes, guarantees for public access to information, proportion of agricultural land with minimum species richness and the number of animal and plant genetic resources in conservation facilities. Some gaps identified in the consultations could not be filled and are instead described in the data gaps and research agenda discussion; in particular, the lack of food loss and waste data was a prominent theme of the consultations.

### Analysis methods

Analyses were carried out in Stata v.17 and R v.4.2.2. The data were compiled into a dataset where all years of available data per country and indicator were included. In two instances (EM-DAT and Varieties of Democracy indices), data prior to 1960 were excluded because no other datasets provided data before that year. Initially, all territories classified in the UN Global Administrative Units List dataset^53^ and present in any datasets were included (94 areas in total). After compiling the complete dataset with all indicators, we investigated whether there was sufficient coverage across all indicators for any territories or areas that are not UN member states to remain in the dataset. A criterion was applied that the area must have at least 80% of all indicators. In practice, all territories were dropped at a much lower threshold, none having more than the median number of variables present for member states (40, where certain indicators are represented in the dataset by more than one variable). In sum, the dataset contains all the available data from 1960 to 2021 for all UN member states, and one indicator (the presence of a food system transformation pathway) defined only in 2022.

The focus of this manuscript is a baseline dataset comprising the latest data point per country per year. Overall, 92.5% of all data points are from 2017–2022, 6.5% are from 2010–2016 and only 1% are from 2000–2009. A small number of observations (*N* = 24 across all indicators) were dropped from the dataset because the latest data point for that country–indicator pair came from prior to 2000. The only indicator where this dropped more than a few observations is female share of landholdings, which has 13 countries whose data point in that cross-sectional dataset is from the 1990s or before. A new data source will become available through the SDG process (SDG 5.a.1) for this indicator in future years.

The Supplementary Information includes analysis of the data from 2000 forward wherever time series are available. Countries are grouped into regions based on modified groupings of the M49 classification system of the UN Statistical Commission, using a combination of continental and sub-regional groupings. Supplementary Fig. 1.2 depicts the alignment of countries to the modified M49 regional grouping used in this paper. Countries are identified by income group using the World Bank country income classification^54^.

The rankings of indicators (Fig. 3) are calculated by ordering every continuous indicator numerically and assigning each country a rank order for every indicator. The rank is reversed for all indicators where a higher value is more desirable (per Extended Data Table 1), such that a ranking of 1 is assigned to the country with the most extreme (highest or lowest) value, whichever direction is desirable for that indicator. We calculated the average rank for all indicators per theme, with the limitation that doing so implicitly weights the thematic average rank for any countries without data for any indicators within the theme. This is an unavoidable limitation and allowed for country-level visualization of data with great variation in their range and units of analysis.

The distributions of the indicators by region and income group relative to the global weighted mean (Fig. 4 and Supplementary Tables 1.1 and 1.4) are presented as the normalized difference from the global weighted mean. The global weighted mean is subtracted from the region (income group) weighted mean and normalized using min–max scaling, which divides the demeaned observation by the total range across all regions (income groups) (that is, it divides by the maximum observed minus the minimum observed). Deviations of region and income group weighted means from the global weighted mean (Supplementary Tables 1.1 and 1.4) are calculated using weighted least squares regression with heteroskedasticity robust standard errors regressing region (income group) on the demeaned observation. Demeaned observations are calculated by subtracting the global weighted mean from each observation. The sign of the demeaned observation is reversed for all indicators where the desirable direction of change is lower. Regression coefficients are the regional (income group) deviation from the global average with the sign indicating whether the region is performing worse (negative sign) or better (positive sign) than the global average. The signed deviation is then translated into a percentage deviation by dividing by the global average to harmonize the presentation of indicators given the different units and scales of their level measurements.

Finally, we emphasize that this exercise was based on a framework of food systems, and therefore we would expect that certain features of a country’s food system would be related to other features of a country’s food system. To explore this, we calculated a Spearman rank correlation matrix (Supplementary Fig. 1.26). However, we caution the interpretation of correlation as redundancy; we do not intend to create a single index, in which case high levels of correlation among the variables entering the model would be problematic. Instead, we put forward this matrix for the purpose of hypothesis generation regarding the key interactions among indicators that merit further investigation, which will be the focus of our research agenda over the next two years.

### Reporting summary

Further information on research design is available in the Nature Portfolio Reporting Summary linked to this article.

### Supplementary information

Supplementary Information Supplementary Appendices 1–4.

Reporting Summary

Supplementary Data 1 Metadata and codebook.

Supplementary Data 2 Baseline dataset.

Supplementary Data 3 Time series dataset.

## Figures and Tables

**Extended Data Table 2:**
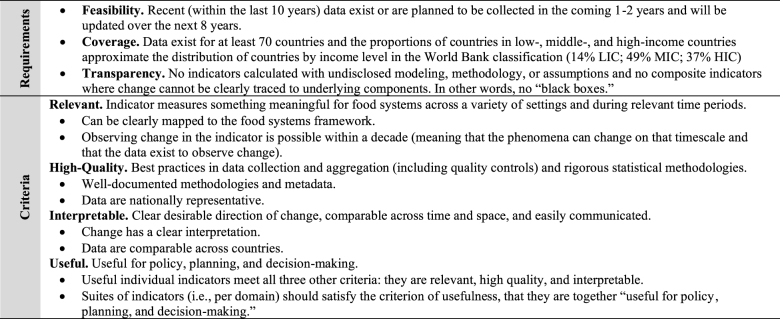
Requirements and criteria used to select indicators

Screening requirements used to eliminate candidate indicators from consideration prior to the consultative process and criteria used to evaluate indicators in the consultative process to select the final list. Criteria definitions describe the overall criteria. Indicators were assessed by the sub-criteria in the bulleted lists used to operationalize each criterion.

## Data Availability

The analysis in this paper relies on numerous datasets in the public domain unless otherwise noted (for which permission to include in our dataset was secured). The metadata contain the necessary links to access the underlying raw data. Static copies of the raw data downloaded and used at the time of this analysis are also available in the GitHub repository with replication code, analysis datasets and all analysis output, at https://github.com/KateSchneider-FoodPol/FSCI_2023Baseline_Replication. The use of any materials in the GitHub repository is subject to a CC BY-NC-SA 4.0 (non-commercial, share alike) licence. The datasets are archived on Harvard Dataverse under a CC BY-NC-SA 4.0 (non-commercial, share alike) licence, and any use or derivatives require attribution of the following: 10.7910/DVN/A1H2SH.

## Extended data

**Extended Data Table 1:** Indicator metadata

Domain	Indicator		Data Source	Description	Rationale for inclusion	Country coverage	Years covered	Desirable direction*	Notable limitations and mitigation
**Food environments**	**1**	Cost of a healthy diet	FAOSTAT	The per capita cost of the least expensive locally available foods to meet requirements for energy and food-based dietary guidelines, per capita, per day (2017 US$)^56,57^.	Food-based dietary guidelines are designed to achieve nutrient adequacy and provide protection of health. This indicator reflects the cost of purchasing a diet aligned to a diet that reflects average amounts in guidelines.	157	2017-2020	↓	Calculated with data from the International Comparison Project (ICP) so the food list is internationally comparable items, and therefore contains fewer local food items which may result in an upward bias to the estimated cost.
	**2**	Availability of fruits and vegetables	FAOSTAT	Amounts of fruits and vegetables available in a country’s food supply (sum of production, imports, and net stocks minus exports, food manufacturing, feed, seed, waste, and other uses) at the national level (expressed as grams per person per day) to align to the unit typically used for dietary recommendations.	Availability of fruits and vegetables is an essential precondition, yet not a guarantee, for their consumption. Consumption of abundant fruits and vegetables is universally recommended in global and national dietary guidance.	174	2010-2019	↑	Availability in the food supply does not equate to consumption, however, it is an appropriate indicator of the food environment.
		Fruits							
		Vegetables							
	**3**	Retail value of ultra-processed foods	Euromonitor	Total sales of ultra-processed foods in the calendar year per person (USD/person).	This indicator proxies the availability of UPFs, defined as foods made of mostly industrial ingredients and additives with minimal amounts of unprocessed foods. These additives are not naturally occurring in the food but are added in the processing phase in order to increase palatability and shelf life. Examples of UPFs include sweet and savory snacks, instant noodles, confectionery, meat substitutes, and soft drinks, among others. These data are not publicly available but have been acquired for use by this Initiative and no comparable public sector data exist to capture this important aspect of food environments.	187	2017-2019	↓	These data are proprietary, and no public source is available. When a publicly available indicator of comparable information value becomes available, this proprietary data will be replaced. This indicator is not present in the released baseline dataset due to lack of license permissions to replicate or disseminate the underlying data.
	**4**	% Population using safely managed drinking water services (SDG 6.1.1)	WHO/UNICEF Joint Monitoring Programme	Percentage of the population that obtains drinking water from an improved water source, defined as one located on premises, available when needed, and free from fecal and chemical contamination.	Access to clean water is essential for food and nutrition security, to avoid foodborne and waterborne illness.	117	2000-2020	↑	Country coverage limitations, collected in more LMICs than HICs.
**Food Security**	**5**	Prevalence of Undernourishment (SDG 2.1.1)	FAOSTAT	An estimate of the proportion of the population that lacks enough dietary energy for a healthy, active life.	An indicator used to monitor hunger at the global and regional level. The estimate is obtained with a model that compares the distribution of habitual food consumption levels with the dietary energy requirements for an average individual in the population.	161	2001-2020	↓	
	**6**	% Population experiencing moderate or severe food insecurity (SDG 2.1.2)	FAOSTAT	Prevalence of the population experiencing moderate or severe food insecurity as measured by the FIES.	The FIES is an experience-based food security scale used to produce a measure of access to food at different levels of severity that can be compared across contexts. It relies on data obtained by asking people, directly in surveys, about the occurrence of conditions and behaviors that are known to reflect constrained access to food.	120	2015-2020	↓	Country coverage limitations, collected in more LMICs than HICs.
	**7**	% Population who cannot afford a healthy diet	FAOSTAT	The share of the population whose food budget is below the cost of a healthy diet.^6,56^	The food budget is defined as 52% of household income, based on the average share of income that households in low-income countries spend on food. Where the minimum cost of a healthy diet (see definition above) exceeds this amount of income, it is considered unaffordable.	141	2017-2020	↓	In countries where food expenditures are lower than 52%, the % who cannot afford a healthy diet may be underestimated.
**Diet quality**	**8**	MDD-W: % adult women meeting minimum dietary diversity	Gallup World Poll	Percentage of women 15-49 years of age who consumed at least five out of ten defined food groups the previous day or night. It is associated with a higher probability of nutrient adequacy for 11 micronutrients.	It is a food group diversity indicator that reflects micronutrient adequacy, summarized across 11 micronutrients. The proportion of women aged 15-49 years who achieve this minimum of five food groups out of ten can be used as a proxy indicator for higher micronutrient adequacy.	41	2021	↑	Country coverage limitations. However, additional countries are collecting the data and coverage will expand in coming years.
	**9**	MDD (IYCF): % children 6-23 months meeting minimum dietary diversity	UNICEF	Percentage of children 6–23 months of age who consumed foods and beverages from at least five out of eight defined food groups during the previous day.^58^	WHO guiding principles for infant and young child feeding recommend that children aged 6–23 months be fed a variety of foods to ensure that nutrient needs are met. A diet lacking in diversity can increase the risk of micronutrient deficiencies, which may have a damaging effect on children’s physical and cognitive development.	100	2005-2020	↑	Country coverage limitations, data collection is limited to LMICs.
	**10**	All-5: % adult population consuming all 5 food groups	Gallup World Poll	Proportion of the population age 15 years and older consuming all five food groups typically recommended for daily consumption: fruits; vegetables; pulses, nuts, or seeds; animal-source foods; and starchy staples.^20^	This indicator reflects the proportion of the population consuming any non-zero amount of each food group, and therefore may reflect minimal adherence to dietary guidelines. These food groups are aligned with the food groups used in the “Cost of a healthy diet” indicator.	41	2021	↑	Country coverage limitations, especially low representation of HICs. However, additional countries are collecting the data and coverage will expand in coming years.
	**11**	Zero fruit or vegetable consumption	Gallup World Poll	Proportion of the population age 15 years and older who did not consume any vegetables or fruits in the previous day.^20^	Consumption of zero vegetables or fruits is an unhealthy practice, as these food groups are associated with reduced risk of NCDs. It is a general population diet quality indicator aligned with the infant and young child feeding indicator (see next).	41	2021	↓	Country coverage limitations, especially low representation of HICs. However, additional countries are collecting the data and coverage will expand in coming years.
		Adults							
		Children 6-23 months	UNICEF	Percentage of children 6–23 months of age who did not consume any vegetables or fruits during the previous day.^58^	Consumption of zero vegetables or fruits is an unhealthy practice, as these food groups are recommended for IYC, and are associated with reduced risk of NCDs.	99	2005-2020	↓	Country coverage limitations, data collection is limited to LMICs.
	**12**	NCD-Protect	Gallup World Poll	The NCD-Protect score is an indicator of dietary factors protective against NCDs, based on consumption during the previous day or night of nine food groups that are associated with meeting WHO recommendations on fruits, vegetables, whole grains, pulses, nuts and seeds, and fiber. The score ranges from zero to nine expressed as an average score for the population age 15 years and older.^20^	Dietary factors protective against NCDs include consumption of whole grains, pulses, nuts or seeds, at least 400g fruits and vegetables per day, and at least 25g of fiber per day. A higher NCD-Protect score indicates inclusion of more health-promoting foods in the diet, and correlates positively with meeting global dietary recommendations.	41	2021	↑	Country coverage limitations, especially low representation of HICs. However, additional countries are collecting the data and coverage will expand in coming years.
	**13**	NCD-Risk	Gallup World Poll	The NCD-Risk score is an indicator of dietary risk factors for NCDs, based on consumption during the previous day or night of eight food groups that are negatively associated with meeting WHO recommendations on free sugar, salt, total and saturated fat, and red and processed meat. The score ranges from zero to nine expressed as an average score for the population age 15 years and older.^20^	Dietary risk factors for NCDs include consumption of >10% of dietary energy from free sugar/day, >5g of salt/day, >30% of total fat/day, >10% of dietary energy from saturated fat/day, >350-500g red meat/week, and any processed meat. A higher NCD-Risk score indicates inclusion of more foods and drinks to limit in the diet, and correlates negatively with meeting global dietary recommendations. The NCD-Risk score is also a proxy for ultra-processed food intake; a higher NCD-Risk score indicates higher ultra-processed food consumption.	41	2021	↓	Country coverage limitations, especially low representation of HICs. However, additional countries are collecting the data and coverage will expand in coming years.
	**14**	Sugar-sweetened soft drink consumption	Gallup World Poll	Proportion of the population age 15 years and older who consumed a sugar-sweetened soft drink during the previous day or night. Sugar-sweetened soft drinks include soda, energy drinks, and sports drinks.	Sugar-sweetened soft drinks are a large source of free sugars; WHO recommends added sugars be limited to less than 10% of total energy. Excessive consumption may increase the risk of overweight and obesity and diet-related noncommunicable diseases.	41	2021	↓	Country coverage limitations, especially low representation of HICs. However, additional countries are collecting the data and coverage will expand in coming years.
**Greenhouse Gas Emissions**	**15**	Food systems greenhouse gas emissions	FAOSTAT	Production based greenhouse gas emissions (carbon dioxide, methane, nitrous oxide and F-gases) for food systems, expressed in kT CO2eq (AR5).	Food systems account for about 30% of total anthropogenic emissions. Reducing food systems emissions is crucial to reduce the impact of climate change and reach the targets of the Paris Agreement. And it is a sub-indicator of the FAO monitoring progress towards sustainable agriculture (SDG 2.4.1).	194	1990-2020	↓	Total greenhouse emissions are the basis of country targets; however, differences are driven in part by country size (in addition to productivity and efficiency). To normalize by country size (area or population) would increase the interpretability of a comparison, however doing so per capita implies attribution of the emissions to the people living there, even if the product is ultimate exported. Since our goal is to track progress of the food system, the total greenhouse gas emissions is most aligned with our objectives, however if improved options become available to address the issue of comparability and attribution of emissions to consumption that account for trade, we will replace this indicator in future publications.
	**16**	Greenhouse gas emissions intensity, by product group	FAOSTAT	Greenhouse gas emissions, CO2 equivalents (carbon dioxide, methane, and nitrous oxide) from the production of different crops and livestock and commodities within the farm gate.	Reducing the emissions intensity is a necessary - but not sufficient solution to reduce GHG emissions. Differences in emission intensities across countries reflect differences in environmental conditions, production systems and production efficiency. Changes in emission intensity over time helps to track improvements in efficiency, adoption of better practices and other changes in production systems. And it is a sub-indicator of the FAO monitoring progress towards sustainable agriculture (SDG 2.4.1). The most informative products for monitoring are shown in the main paper with additional products included in SD-A and dataset (SD-F).	176	1961-2020	↓	Product available are limited by data availability but include the products of greatest concern in terms of share of agricultural production emissions.
		Cereals							
		(excl. rice)							
		Beef				184		↓	
		Cow’s milk				179		↓	
		Rice				119		↓	
**Production**	**17**	Food product yield, by food group	FAOSTAT	Yield means the harvested production per ha for the area under cultivation, expressed in tonnes per hectare or kg per animal.	Yields measure the efficiency with which inputs are used to produce agricultural output. Improving production efficiency is considered a critical path to meet food and nutrition security needs of current and future generations. The yields data, subtracted from consumptive need can serve as an indicator of coherence between sustainable production and healthy consumption targets. The most informative products for monitoring are shown in the main paper with additional products included in SD-A and the dataset (SD-F).	184	1961-2020	↑	Crop mix varies somewhat in the cereals product group, however it is sufficiently standardized across the world for valid geographic comparison.
		Cereals							
		Fruits				186		↑	Crop mix varies by country and region and therefore geographic comparisons must be made with caution. Fruits are critical to healthy diets and increased productivity is needed to meet the nutritional needs of current and future populations. Meanwhile, comparison at the country level over time remains a valid monitoring indicator.
		Beef				179		↑	
		Cow’s milk				167		↑	
		Vegetables				187		↑	Crop mix varies by country and region and therefore geographic comparisons must be made with caution. Vegetables are critical to healthy diets and increased productivity is needed to meet the nutritional needs of current and future populations. Meanwhile, comparison at the country level over time remains a valid monitoring indicator.
**Land**	**18**	Cropland expansion	FAOSTAT	Average change in total cropland over previous 5 years in percentage terms relative to the total cropland (total cropland as of year 5 of each 5-year window, the year to which the change value is assigned). Computed for every year except the first five years in the time series (1961-1966). Cropland is defined according to the FAO land use classification, based on the World Census of Agriculture.	The increasing demand for food can be met by increasing yields or expanding agricultural lands. Land use change is a major driver of biodiversity loss and climate change. Even with improved production efficiency, the total impact of the food system can still increase. Halting or reducing cropland expansion is another crucial step towards reducing the impact of the food system.	173	1966-2020	↓	
**Water**	**19**	Agriculture water withdrawal as % of total renewable water resources	AQUASTAT	Water withdrawn for irrigation in a given year, expressed in percent of the total renewable water resources.	Water is often a limiting factor for agricultural production and increasing irrigation is one of the main proposed ways to increase food production. At the same time, water scarcity is already a serious issue in many regions of the world and the situation is expected to get worse under climate change and increasing demand. This is also one of the eight proxy sub-indicators currently proposed for monitoring SDG 2.4.1 ‘’sustainable and productive agriculture’’.	175	1967-2018	↓	
**Biosphere Integrity**	**20**	Functional integrity: % agricultural land with minimum level of natural habitat	DeClerck et al (2021)^59^	Measures the proportion of semi-natural or natural habitat per km^2^ of cropland or rangeland. The threshold is set at 10%, based on evidence that agroecosystem services are lost below 10%. At national level this indicator is articulated as the proportion of the country’s agricultural lands having above or below the integrity threshold.	There is great pressure to expand agricultural area to increase food production. At the same time, there is growing concern about the loss of biodiversity and ecosystems services provided by natural habitats in agriculture. For example, fruit, vegetable, and legume production depend on pollination services, safe food production is dependent on regulations of pest and diseases provided by natural predators.	194	2015	↑	Only one year of data has been released for this indicator but additional years are anticipated from the authors. The underlying data are publicly available, and the methodology can be replicated by the FSCI if needed.
	**21**	Fishery health index progress score	Minderoo Foundation	The product of stock data availability and stock sustainability.	Fisheries can provide a substantial contribution to people’s diets. Overfishing and environmental degradation have resulted in a drop of catching rates and raised questions about the future contribution that some fisheries could have in the future.	122	2021	↑	The indicator is recently developed but will be updated every three years.^60^
**Pollution**	**22**	Total pesticides per unit of cropland	FAOSTAT	The use of pesticides per area of cropland (which is the sum of arable land and land under permanent crops) at national level expressed as kg active ingredient per hectare.	Pesticide use in general, and use of hazardous pesticides in particular pollutes the biosphere at all levels, damaging flora and fauna and putting human health at risk. Reducing use and reverting current trends is a fundamental component of sustainable agricultural production and hence food systems. This is also one of the eight proxy sub-indicators currently proposed for monitoring SDG 2.4.1 “sustainable and productive agriculture”.	153	1990-2020	↓	
	**23**	Sustainable nitrogen management index	Zhang et al (2022)^61^	A one-dimensional ranking score that combines two efficiency measures in crop production: Nitrogen use efficiency and land use efficiency (crop yield), to provide a measure of the environmental efficiency of agricultural production.	Overuse of synthetic fertilizers pollutes the biosphere at all levels, with specific risk to aquifers and pollution downstream and into the oceans. Reducing use and reverting current trends is a fundamental component of sustainable agricultural production and hence food systems.	188	1961-2018	↑	Data are expected to be updated in future years by the authors. The underlying data are publicly available, and the methodology could be replicated by the FSCI to update, if need be.
**Poverty & Income**	**24**	Share of agriculture in GDP	FAOSTAT	The share of income derived from agriculture is a key parameter in agricultural transformation and the evolution of food systems. Historically, structural transformation has been characterized by a transition from a low-productivity agriculture-based economy that employs the majority of workers and generates the most output, to one dominated by industry and services and a smaller, more productive agriculture sector.	The indicator is a measure of the stage of structural transformation in the economy, closely related to the economic dimensions of food system transformation. It is correlated with economic development: countries with larger share of income coming from agriculture are poorer.	192	2001-2020	↓	This is a proxy indicator well understood in agricultural economics and development as an indicator of structural transformation but is not as easily interpretable outside of that disciplinary lens. It is used here as a proxy for rural poverty, which would be the ideal indicator. When a rural poverty indicator is available with sufficient country coverage, this indicator will be replaced. Meanwhile we present more explanation for the underlying rationale to ensure widespread interpretability.
**Employment**	**25**	Unemployment rate, rural	ILO	Share of people of employment age that are unemployed, disaggregated by total, urban, and rural.	The share of unemployed people in rural areas is an indicator of economic activity and livelihood opportunities for people in areas dominated by agriculture.	177	2005-2020	↓	People in rural areas are not exclusively engaged in food systems, and therefore rural unemployment captures the whole of the rural economy and not specifically the size of the food system labor market relative to the population. However, the transformation of agriculture is linked to the ability of the rural economy to provide non-farming jobs in the structural transformation process and therefore this indicator reflects the potential for people to move out of farming and into wage labor.
	**26**	Underemployment rate, rural	ILO	Time-related underemployment is defined as people who (during the reference period) are: willing and available to work additional hours and worked less than a relevant nationally determined threshold of working time.	The use of the time-based underemployment rate is recommended to be used in combination with the unemployment rate, as unemployment does not capture the quality of employment. The use of these two indicators together was recommended in the 19th International Conference of Labour Statisticians in 2013. Monitoring the relationship between unemployment and time-related underemployment in rural vs urban areas by region may be useful in tracking shifts in agricultural employment. Disaggregated analysis by sex (SD-A) provides additional understanding of the shifts taking place in rural agricultural employment.	104	1996-2021	↓	People in rural areas are not exclusively engaged in food systems, and therefore rural underemployment captures the rural economy more broadly and is not specific to food systems. As with unemployment, underemployment also indicates whether there is sufficient labor demand to absorb all the available workers at full capacity. Food system transformation requires the sustainable productivity in agriculture, which requires that excess labor in LMICs be able to move out of farming as their primary income and livelihood endeavor into higher productivity activities.
**Social Protection**	**27**	Social protection coverage	World Bank	The share of individuals in the total population from households where at least one member participates in a social protection and labor market program, including non-contributory social safety nets (e.g. cash transfers, school feeding), contributory social insurance (e.g. old-age pension, health insurance), and labor market programs (e.g. job training, unemployment insurance).	Social protection supports food systems through demand and supply channels for both labor and food items. For example, social protection supports healthier diets when implemented in the form of school feeding, contains nutrition sensitive programming, or provides income support for increased consumption of nutrient-dense foods, dietary diversity, and micronutrient intake. Cash transfers, especially when combined with skills development interventions and entrepreneurship support programs, may have positive impacts on supply of products from food systems, including through spurring entrepreneurship in non-farming businesses and increasing the smallholder farmer productivity by providing a safety net to take risks and try new technologies or invest in assets or capital improvements.	122	2000-2019	↑	Though not specific to food systems, social protection expands the options available especially the people with the fewest resources and safety nets, among whom are the world’s small scale food producers and urban residents in low-skill jobs whose work is often informal and tied to food systems such as through trade and informal vending.
	**28**	Social protection adequacy	World Bank	The total social protection benefit amount received by beneficiary households (direct and indirect beneficiaries) as a percentage of beneficiaries’ post-transfer, household wealth. This includes non-contributory social safety nets and contributory social insurance with monetary transfers but excludes safety nets without a monetary transfer and labor market programs.	The adequacy of social protection programs measures the likelihood that social protection intervention will have desired impacts on households’ consumption of healthy diets and on improving the productivity of food production and distribution.	117	2000-2019	↑	
**Rights**	**29**	% Children 5-17 engaged in child labor	UNICEF	Percent of children 5-17 years classified as engaged in child labor over the total population aged 5-17 years, disaggregated by sex. Criteria for child labor varies by age group:	ILO conventions 138 and 182 (which outlaw child labor and exploitation) have been almost universally ratified. It is a regularly monitored indicator with good global coverage.	99	2010-2021	↓	While the data used for this indicator are not disaggregated by economic activity, child labor is predominantly associated with agriculture and rural areas, which makes this indicator informative for food systems monitoring.
				1) Age 5 to 11 years: At least 1 hour of economic work or 21 hours of unpaid household services per week.					
				2) Age 12 to 14 years: At least 14 hours of economic work or 21 hours of unpaid household services per week.					
				3) Age 15 to 17 years: At least 43 hours of economic work per week.					
	**30**	Female share of landholdings	FAO Gender and Land Database	Distribution of land holdings by sex (female %).^62^	The stark inequality in female land ownership occurs in countries of all income levels and belies other types of gender-based discrimination with profound consequences for livelihoods and resource use within and beyond the food system.	112	1988-2016	↑	The data used here are not currently being updated and will eventually be replaced by SDG 5.a.1 on agricultural landholding by sex but which are not yet available for a sufficient number of countries.
**Shared Vision & Strategic Planning**	**31**	Civil society participation index	Varieties of Democracy	The core civil society index is designed to provide a measure of a robust civil society, understood as one that enjoys autonomy from the state and in which citizens freely and actively pursue their political and civic goals, however conceived.	Captures whether an enabling environment exists for citizens to articulate their preferences over the food system, ensuring that policy goals are broadly representative.	172	1960-2021	↑	
	**32**	% Urban population living in cities signed onto the Milan Urban Food Policy Pact^+^	Milan Urban Food Policy Pact / Oakridge National Laboratory (Landscan population product) / FSCI	Proportion of the total national urban population living in cities that signed on to the Milan Urban Food Policy Pact. There were 221 cities from 73 different countries in 2020.	Indicates intentionality by subnational governments to prioritize food policy in their urban planning.	187	2020	↑	
	**33**	Degree of legal recognition of the Right to Food^+^	FAOLEX / FSCI	Policies are classified based on the FAOLEX database five-group typology as:	Indicates a government’s recognition that guaranteeing food security to its citizens is one of its main responsibilities. As a human right, governments have duties to protect and fulfil this right, and the citizens have entitlements to be free from hunger and food insecurity.	194	2021	↓	
				1) Explicit protection of the right to food or directive of state policy.					
				2) Some other implicit recognition, codification of international statues, or other pertinent provisions.					
				3) None: countries with no policies catalogued in the FAOLEX database.					
	**34**	Presence of a food system transformation pathway (from the UNFSS)	FAO / FSCI	The country has developed a food system transformation pathway, as reported to the FAO UN Food Systems Summit Hub.	Specific strategy that addresses food systems from a systems perspective at the country level.	194	2022	↑	
**Effective Implementation**	**35**	Government effectiveness index	World Governance Indicators	Perceptions of the quality of public services, the quality of the civil service and the degree of its independence from political pressures, the quality of policy formulation and implementation, and the credibility of the government’s commitment to such policies.	A credible government with high levels of bureaucratic quality is more likely to effectively implement complex food systems policies.	192	1996-2020	↑	
	**36**	International Health Regulations State Party Assessment report (IHR SPAR), Food safety capacity	WHO Global Health Observatory	Mechanisms are established and functioning for detecting and responding to foodborne disease and food contamination.	Indicates whether a government can effectively manage food safety challenges.	191	2018-2020	↑	Though presented as a continuous indicator, the data take only discrete values from 0 to 100 in 20-unit intervals, reducing the ability to identify key variation across countries.
	**37**	Presence of health-related food taxes^+^	World Cancer Research Fund International NOURISHING / FSCI	Reflects the presence of any health-related tax at all in the country (defined as binary given the diversity of policy mechanisms and objectives catalogued). In some countries, however, the tax may not be a federal policy and may only apply to certain subnational areas such as municipalities.	Indicates a government’s willingness and ability to implement policy instruments aimed at promoting consumer health.	194	2021	↑	Binary construction of this indicator does not capture differences in the targeted items or the intensive margin if governments apply a tax to multiple foods. However, many of the taxes captured in the database are implemented at the subnational level, so a count-based construction would conflate multiple administrative units implementing taxes with multiple items for which a tax applies.
**Accountability**	**38**	V-Dem Accountability index	Varieties of Democracy	Combines horizontal (across government ministries), vertical (between voters and leaders), and diagonal (between civil society organizations, media, and government) accountability. It is measured by the following questions:	Governments that demonstrate higher levels of general accountability are more likely to allow for citizen/legislative oversight of food system commitments and spending.	172	1960-2021	↑	
				4) To what extent is the ideal of government accountability achieved?					
				5) Does the state’s population hold government accountable through elections?					
				6) Are there checks and balances between institutions?					
				7) Is there oversight by civil society organizations and media activity?					
	**39**	Open Budget Index Score	International Budget Partnership	A score based on an assessment of the public’s access to information on how the central government raises and spends public resources.	Governments that demonstrate higher levels of general accountability are more likely to allow for citizen/legislative oversight of food system commitments and spending.	120	2006-2021	↑	
	**40**	Guarantees for public access to information (SDG 16.10.2)	Sustainable Development Goals	The number of countries that adopt and implement constitutional, statutory, and/or policy guarantees for public access to information	Citizens are more likely to be able to hold their governments accountable for food system and other policies if they can access information about government activities	194	2021	↑	
**Exposure to Shocks**	**41**	Ratio of total damages of all disasters to GDP	EM-DAT	Total estimated damages (nominal 000’USD, meaning unadjusted for inflation) divided by GDP (nominal 000’USD) and multiplied by 100 for readability. A disaster is only capture in the database if it meets at least one of the following criteria: 10 or more dead, 100 or more affected/injured, call for external assistance, or declaration of emergency. Therefore the events captured are significant, and can be characterized as medium or extreme events.	An essential element of any resilience analysis is to assess and, if possible, document the intensity, nature, and frequency of the shock and stressors that a particular system is exposed to. The EM-DAT database has superior country and year coverage to other available options such as the SDG indicators related to economic losses due to disasters.	187	1960-2021	↓	This indicator is not specific to food systems, it would be preferable to have damages associated with the food system, or at least covering agricultural losses specifically. Furthermore, it is based on the EM-DAT economic losses indicator, which is missing for many disaster events recorded in the EM-DAT database and more likely to be missing for LMICs compared to HICs. Alternative data sources (such as the SDG data) have even greater coverage limitations, but this indicator will be replaced by an indicator of agricultural losses currently under development by FAO and which will be based on a combination of EM-DAT and FAOSTAT data.
**Resilience capacities**	**42**	Dietary sourcing flexibility index	FAO (soon to be available in FAOSTAT)	Measures the diversity of pathways through which food reaches consumers. Expresses how difficult it is to disrupt a country’s food supply. Considers three possible pathways a unit of food can reach a consumer:	Diversification (of portfolios, livelihoods, income, source of food, etc.) is an essential risk strategy well established in both theoretically and empirically in the general literature. In the present case the dietary sourcing flexibility index capture the possible diversification of sources of food supply.	151	2018	↑	This indicator was recently developed and has only been computed for one year so far. It will become a regular series in FAOSTAT to be updated regularly in the future.
				1) Food produced domestically;					
				2) Imported food;					
				3) Stocks carried over from the previous year.					
				Total sources for calories is the indicator used, disaggregation by source and for other nutrients and food groups are available.					
	**43**	Mobile cellular subscriptions	International Tele-communications Union / World Bank	Subscriptions to a public mobile telephone service using cellular technology, which provides access to the public switched telephone network. Includes post-paid and prepaid subscriptions.	The number of mobile cellular telephone subscriptions has been included in the computation of food system resilience as a proxy for countries’ infrastructure level as well as a direct indicator of response capacity.	193	1991-2020	↑	The data count number of phone subscriptions in the numerator divided by population, which could overestimate or double-count where one person holds more than one phone subscription. When data for SDG 5.b.1 become available for a sufficient number of countries, this indicator will be replaced.
	**44**	Social capital index	Legatum Institute / FSCI	A composite index based on a subset of indicators from the Social Capital pillar of the Legatum Prosperity Index, which assesses social cohesion and engagement, community and family networks, and political participation and institutional trust. The social capital index used here is a composite of the indicators of "Help from family & friends”; “Generalized interpersonal trust”; “Confidence in financial institutions”; and “Public trust in politicians or confidence in national government”, based on the relevance of these indicators to resilience. The index is calculated as the geometric mean of the four variables, each measured on a scale that ranges from 0 (low) to 100 (high).	Social capital is another extremely important element of resilience in general. The score on the Social Capital pillar of the Legatum Prosperity Index has been included in that regard.	165	2007-2021	↑	
**Agro- and Food Diversity**	**45**	Proportion of agricultural land with minimum level of species diversity (crop and pasture)^+^	Jones et al. (2021)^63^	The proportion of agricultural land with minimum species diversity is defined by the top global quartile of land with the highest species richness. The threshold number of species at which (and above) covers 25% of total global ag land (the 25% of land with the most diversity) is 24 species. Therefore, the indicator reflects the percentage of agricultural land per country with 24 or more species.	Diversification (of portfolios, livelihoods, income, source of food, etc.) is an essential risk strategy well established in both theoretically and empirically in the general literature. The proportion of agricultural land with minimum level of species diversity (crop and pasture) contributes to landscape and livelihood buffering capacity, with more functionality to cope with for example unexpected environmental changes or shocks and spreading risks to cope with for example market volatility. It is therefore a relevant indicator reflecting the diversity of the agricultural production component of the food system.	180	2010	↑	This indicator has only been computed for a single year, relying on the data and methodology developed by Jones et al. (2021)^63^, but will be carried out for additional years as the FSCI moves forward.
	**46**	Number of (a) plant and (b) animal genetic resources for food and agriculture secured in either medium- or long-term conservation facilities (SDG 2.5.1)	Sustainable Development Goals	The conservation of plant genetic resources for food and agriculture in medium- or long-term conservation facilities (ex situ in gene banks) represents the most trusted means of conserving genetic resources worldwide. Measured as:	Diversification (of portfolios, livelihoods, income, source of food, etc.) is an essential risk strategy well established in both theoretically and empirically in the general literature. The Number of plant and animal genetic resources for food and agriculture secured in conservation facilities is considered a buffering capacity, providing a back-up in times of crises and shocks and indicating the food and agricultural diversity ex-situ ‘stock’ of a country	97	2000-2020	↑	
		Plants		a) Plant genetic resources accessions stored ex situ (number).					
	**47**	Animals		b) Number of animal genetic resources represents the number of species with sufficient genetic material stored for reconstitution.		97	2000-2021	↑	
**Resilience Responses / Strategies**	**48**	Coping strategies index	WFP	The reduced Coping Strategies Index (rCSI) measures the frequency and severity of household behaviors when faced with shortages of food or financial resources to buy food. It is calculated using five standard food consumption-based strategies and severity weighting, a higher score indicates more frequent and/or extreme negative coping strategies. It measures the proportion of population using extreme coping strategies (with a rCSI >=19). It is the highest observed daily prevalence throughout the year.	Understanding and documenting the type of responses that actors adopt when they are faced with shocks or stressors is an essential part of resilience analysis. In theory the objective of a resilience building intervention is to reduce the propensity of actors to engage in detrimental responses and to increase their ability to engage in more ‘positive’, adaptive or transformative responses. At the present time the rCSI is only recorded in a limited number of, mostly low-income, countries.	114	2021	↓	
**Long-term Outcomes**	**49**	Food price volatility^+^	FAOSTAT / FSCI	Domestic food price volatility index measures the variation (volatility) in domestic food prices over time, measured as the relative variation in the domestic food price index, a standardized measure of the cost of a basket of goods. High values indicate a higher volatility (more variation) in food prices.	Different indicators can be used to assess the long-term outcomes of a system resilience. In the case of food systems, the ability of the system to maintain a low price-volatility in the face of shocks, is a direct way to assess the system resilience. The lower the volatility, the better.	42	2000-2021	↓	
	**50**	Food supply variability	FAOSTAT	This indicator uses the data on dietary energy supply from the Food Balance Sheet to measure annual fluctuations in the per capita food supply (kcal), represented as the standard deviation over the previous five years per capita food supply. Food supply variability results from a combination of instability and responses in production, trade, consumption, and storage, in addition to changes in government policies such as trade restrictions, taxes and subsidies, stockholding, and public distribution.	Along with food price volatility (see above), the ability of the system to maintain a low variability in the supply of food products in the face of shocks is a direct way to assess the system long-term resilience. A resilient food system would be able to keep the variability of food supply low despite being hit by shocks. Therefore, the lower the food supply variability, the better.	181	2000-2021	↓	
**Contextual variables**	GDP		World Bank	Sum of gross value added by all resident producers in the economy plus any product taxes and minus any subsidies not included in the value of the products. Data are in current U.S. dollars. Dollar figures for GDP are converted from domestic currencies using single year official exchange rates.	Provides the denominator for the exposure to shocks indicator and for analysis of indicator relationship to GDP. Weighting variable for weighted means as specified in Table 1.	192	1960-2021		
	Population		World Bank	Total population, counting all residents regardless of legal status or citizenship at midyear estimates.	Weighting variable for weighted means as specified in Table 1.	193	1960-2021		
	Urban population		Oak Ridge National Laboratory / Landscan	Total urban population based on GADM administrative unit boundaries.	Weighting variable for weighted means as specified in Table 1.	187	2020		
	Land area		FAOSTAT	Country area excluding area under inland waters and coastal waters.	Weighting variable for weighted means as specified in Table 1.	194	1961-2020		
	Cropland		FAOSTAT	Land used for cultivation of crops. Includes all arable land and permanent crops.	Weighting variable for weighted means as specified in Table 1.	193	1961-2020		
	Agricultural land		FAOSTAT	Land used for cultivation of crops and animal husbandry. Includes cropland and permanent meadows and pastures.	Weighting variable for weighted means as specified in Table 1.	193	1961-2020		

^+^Indicates substantial value add by FSCI to existing data.

*Desirable direction: ↑ denotes a higher value is more desirable, ↓ denotes a lower value is more desirable.

Important information pertaining to each indicator. Some information reports metadata from the original data source including its source, definition, and coverage. Other information includes the rationale for inclusion, desirable direction of change, and notable limitations as determined by the authors. Country and year coverage refers to coverage in the FSCI dataset, limited to UN member states and all available years as of February 2023 when the data were last pulled.

## Code availability

Replication code for this paper is available on GitHub at https://github.com/KateSchneider-FoodPol/FSCI_2023Baseline_Replication.
