# Supplementary material for: The state of food systems worldwide in the countdown to 2030
Source: Nat Food. 2023 Dec 19;4(12):1090–110. doi: 10.1038/s43016-023-00885-9 (PMC10730405; doi:10.1038/s43016-023-00885-9)
Supplement: Supplementary file 1 — Supplementary Appendices 1–4. [file 43016_2023_885_MOESM1_ESM.pdf]

nature food

Analysis https://doi.org/10.1038/s43016-023-00885-9

The state of food systems worldwide in the countdown to 2030

In the format provided by the authors and unedited

The state of food systems worldwide in the countdown to 2030

Supplementary Information

Supplementary Information – Appendix 1

1. Theory of transformation.
2. Countries by regional group (modified M49 grouping)
3. Regional visualizations of data coverage.
4. Supplementary analysis: regional average ranking by theme (available in .xlsx format in the "Results Table" file in the GitHub replication folder here (direct file link).
5. Supplementary analysis: regional and income group deviations from the global mean, weighted means, and medians (available in .xlsx format in the "Results Table" file in the GitHub replication folder here (direct file link).
6. Analysis and visualization of indicator relationship with GDP per capita, by theme.
7. Comprehensive visualizations for all indicators over time, and by region, income group, and other relevant disaggregation, where data are available.

Supplementary Information – Appendix 2

The list of candidate indicators that were ultimately excluded.

Supplementary Information – Appendix 3

Reports from the indicator selection process (survey report, reports from regional consultations).

Supplementary Information – Appendix 4

List of alignment to SDGs, related initiatives and how they relate to the FSCI work, and map of the FSCI architecture and indicators to the Food System Transformation Pathways developed by countries.

Supplementary Data (external files)

All datasets are archived on Harvard Dataverse (doi:10.7910/DVN/A1H2SH), under a CC-BY-NC-SA 4.0 license.

All source data and replication files and code are available in the following GitHub repository: https://github.com/KateSchneider-FoodPol/FSCI_2023Baseline_Replication. All replication files are licensed under a CC-BY-NC-SA 4.0 license.

- **Supplementary Data 1 (external file available here):** Metadata and codebook Excel workbook
- **Supplementary Data 2 (external files available here):** Baseline dataset (latest data point per country-indicator)
- **Supplementary Data 3 (external files available here):** Time series dataset

Supplementary Information, Appendix 1: Supplementary Analysis

Supplementary Tables……………………………………………………………………….…..4
Table 1.1. Percent deviation from global mean, by region and desirable direction of change‡
Table 1.2 Regional weighted mean values
Table 1.3 Regional median values
Table 1.4. Income group deviation from global weighted means, by desirable direction of change‡
Table 1.5 Income group weighted means‡
Table 1.6 Income group median values

Supplementary Figures…………………………………………………………………….....4
Figure 1.1: FSCI Theory of Transformation
Figure 1.2 Country alignment to modified M49 regions
Figure 1.3 Indicator coverage by country and extent of time series, Central Asia
Figure 1.4 Indicator coverage by country and extent of time series, Eastern Asia
Figure 1.5 Indicator coverage by country and extent of time series, Latin America & Caribbean
Figure 1.6 Indicator coverage by country and extent of time series, Northern Africa & Western Asia
Figure 1.7 Indicator coverage by country and extent of time series, Northern America & Europe
Figure 1.8 Indicator coverage by country and extent of time series, Oceania
Figure 1.9 Indicator coverage by country and extent of time series, South-Eastern Asia
Figure 1.10 Indicator coverage by country and extent of time series, South Asia
Figure 1.11 Indicator coverage by country and extent of time series, Sub-Saharan Africa
Figures 1.13 Country average ranking by theme and region: South-Eastern Asia
Figures 1.14 Country average ranking by theme and region: Oceania
Figures 1.15 Country average ranking by theme and region: Northern America & Europe
Figures 1.16 Country average ranking by theme and region: Northern Africa & Western Asia
Figures 1.17 Country average ranking by theme and region: Latin America & Caribbean
Figures 1.18 Country average ranking by theme and region: Eastern Asia
Figures 1.19 Country average ranking by theme and region: Central Asia
Figures 1.20 Country average ranking by theme and region: Sub-Saharan Africa
Figure 1.21 Relation to GDP per capita: Diets, nutrition, and health
Figure 1.22 Relation to GDP per capita: Environment, natural resources, and production
Figure 1.23 Relation to GDP per capita: Livelihoods, poverty, and equity
Figure 1.24 Relation to GDP per capita: Governance
Figure 1.25 Relation to GDP per capita: Resilience
Figure 1.26 Spearman rank correlation coefficient matrix, latest data point per country-indicator

Supplementary Figures for all Indicators, by Thematic Area………………..….49

Diets, Nutrition & Health

1.1.1 Distribution of the cost of the least-cost healthy diet ($PPP/capita/day) by region, 2017-2020
1.1.2 Distribution of the cost of the least-cost healthy diet ($PPP/capita/day) by country income groups, 2017-2020
1.1.3 Change in fruit and vegetable availability, 2010-2019, by region
1.1.4 Change in fruit and vegetable availability, 2010-2019, by income group
1.1.5 Retail value of Ultra-Processed Foods per capita, 2017-2019, by region
1.1.6 Retail value of Ultra-Processed Foods per capita, 2019
1.1.7 Percent population using safely managed drinking water services (SDG 6.1.1), 2000-2020, by region
1.1.8 Percent population using safely managed drinking water services (SDG 6.1.1), 2000-2020, by income group
1.1.9 Prevalence of Undernourishment, 2001-2020, by region
1.1.10 Prevalence of Undernourishment, 2001-2020, by income group
1.1.11 Percent population experiencing moderate or severe food insecurity, 2014-2020, by region
1.1.12 Percent population experiencing moderate or severe food insecurity, 2014-2020, by income group
1.1.13 Percent population who cannot afford a healthy diet, 2017-2020, by region
1.1.14 Percent population who cannot afford a healthy diet, 2017-2020, by income group
1.1.15 Percent adult women (15-49 y) meeting minimum dietary diversity, 2021
1.1.16 Percent of children 6-23 months meeting minimum dietary diversity, by sex
1.1.17 Percent of children 6-23 months meeting minimum dietary diversity, by urban/rural
1.1.18 Percent adult population (≥15 y) consuming all 5 food groups, 2021
1.1.19 Percent adult population (≥15 y) with zero fruits and vegetables consumption, 2021
1.1.20 Prevalence of zero fruits and vegetables consumption, children 6-23 months, by sex
1.1.21 Prevalence of zero fruits and vegetables consumption, children 6-23 months, by urban/rural
1.1.22 NCD-Protect, adults (≥15 y), 2021
1.1.23 NCD-Risk, adults (≥15 y), 2021
1.1.24 Prevalence of sugar-sweetened soft drink consumption, adults (≥15 y), 2021

Environment, Natural Resources, & Production

1.2.1 Total food system emissions, 2000-2020, by region
1.2.2 Total food system emissions, 2020
1.2.3 Emissions intensity (kg CO_2eq/kg product), Staple foods, 2000-2020, by region
1.2.4 Emissions intensity (kg CO_2eq/kg product), Meats, 2000-2020, by region
1.2.5 Emissions intensity (kg CO_2eq/kg product), Other animal-source foods, 2000-2020, by region
1.2.6 Yield, Staple foods, 2000-2020, by region
1.2.7 Yield, Meat, 2000-2020, by region
1.2.8 Yield, Other animal-source foods, 2000-2020, by region
1.2.9 Yield, Pulses & Nuts, 2000-2020, by region
1.2.10 Yield, Fruits & Vegetables, 2000-2020, by region
1.2.11 Cropland expansion, 2020 (% change over prior 5 years)
1.2.12 Agriculture water withdrawal as % of total renewable water resources, 2018
1.2.13 Agriculture water withdrawal as % of total renewable water resources, by region
1.2.14 Agriculture water withdrawal as % of total renewable water resources, by income group
1.2.15 Functional integrity: Percent agricultural land with minimum level of natural habitat, 2015
1.2.16 Fishery health index progress score, 2021
1.2.17 Total pesticides per unit of land (kg/ha), 2000-2019, by region
1.2.18 Total pesticides per unit of land (kg/ha), 2019

1.2.19 Sustainable nitrogen management index, 2000-2018, by region
1.2.20 Sustainable nitrogen management index, 2018

Livelihoods, Poverty, & Equity
1.3.1 Share of agriculture in GDP, 2019
1.3.2 Share of agriculture in GDP, 2000-2019, by region
1.3.3 Unemployment and Underemployment rates by sex, age, and urban/rural, 2020
1.3.4 Unemployment rate (total), 2010-2020, by region
1.3.5 Underemployment rate (total), 2010-2020, by region
1.3.6 Unemployment rate (total), 2010-2020, by income group
1.3.7 Underemployment rate (total), 2010-2020, by income group
1.3.8 Unemployment and underemployment (total), 2005-2020, by region
1.3.9 Unemployment and underemployment (total), 2005-2020, by income group
1.3.10 Social protection coverage and adequacy
1.3.11 Percent children 5-17 engaged in child labor, by sex, by region
1.3.12 Percent children 5-17 engaged in child labor, by sex, by income group
1.3.13 Distribution of landholdings by sex

Governance
1.4.1 Civil society participation index, 2021
1.4.2 Civil society participation index, 2000-2021, by region
1.4.3 Civil society participation index, 2000-2021, by income group
1.4.4 Percent urban population living in cities signed onto the Milan Urban Food Policy Pact, 2020
1.4.5 Percent urban population living in cities signed onto the Milan Urban Food Policy Pact, 2020, by country and region
1.4.6 Degree of legal recognition of the Right to Food, 2021
1.4.7 Presence of a food policy transformation pathway (from the UN Food Systems Summit processes), 2022
1.4.8 Government Effectiveness Index, 2000-2020, by region
1.4.9 Government Effectiveness Index, 2000-2020, by income group
1.4.10 International Health Regulations State Party Assessment report (IHR SPAR), Food safety capacity, 2020
1.4.11 Presence of health-related food taxes, 2021
1.4.12 V-Dem Accountability index, 2021
1.4.13 V-Dem Accountability index, 2000-2021, by region
1.4.14 V-Dem Accountability index, 2000-2021, by income group
1.4.15 Open Budget Index score, by income group
1.4.16 Guarantees for public access to information, by year of adoption

Resilience
1.5.1 Exposure to shocks: Ratio of total damages of all disasters to GDP, 2012-2021
1.5.2 Dietary sourcing flexibility index, by nutrient / food group and country income level, 2018
1.5.3 Dietary sourcing flexibility index, Calories, 2018
1.5.4 Mobile cellular subscriptions (per 100 people), 2000-2020, by region
1.5.5 Mobile cellular subscriptions (per 100 people), 2011-2020 average
1.5.6 Social capital index, 2007-2021, by region
1.5.7 Social capital index, 2007-2021, by income group
1.5.8 Social capital index, 2017-2021
1.5.9 Percentage of agricultural land with minimum level of species diversity (crop and pasture), 2010
1.5.10 Number of wild useful plants for food and agriculture secured in conservation facilities (SDG 5.2.a), 2020

1.5.11 Number of animal genetic resources for food and agriculture secured in conservation facilities (SDG 5.2.b), 2021
1.5.12 Prevalence of reduced coping strategies, 2021
1.5.13 Food price volatility, 2019-2021 (average)
1.5.14 Food supply variability, 2000-2020, by region
1.5.15 Food supply variability, 2000-2020, by income group
1.5.16 Food supply variability, 10-year average, (2011-2020)
1.5.17 Food System Resilience

Supplementary Information, Appendix 1: Supplementary Tables

Table 1.1. Percent deviation from global mean, by region and desirable direction of change‡

						% Deviation of regional weighted mean from global weighted mean, sign aligned to desirable direction of change										
**Domain**		**Indicator**		**Global weighted mean**	**Dir. of Δ**	**Latin America & Caribbean**	**Northern America & Europe**	**Oceania**	**Northern Africa & Western Asia**	**Central Asia**	**Eastern Asia**	**South-eastern Asia**	**Southern Asia**	**Sub-Saharan Africa**	**Joint signif. (F-test) p-val (sig)§**	
	Diets, Nutrition, & Health															
Food environments	**1**	Cost of a healthy diet		US PPP $3.3 per person/day	↓	1.2	4.9	21.1***	-3.1	10.3	1.3	-27.4***	6.2	-1.1	0.0000	***
	**2**	Availability of fruits and vegetables	Fruits	223.8 grams per capita/ day	↑	37.6*	7.9	20.4	4	-30.1	-36.3*	-22.3	-29.9	-24.3*	0.0017	**
			Vegetables	246.8 grams/ capita/ day	↑	-25.2*	32.6**	-37.4**	50.1**	133.2***	91.9	-9.6	-13.7	-50***	0.0000	***
	**3**	Retail value of ultra-processed foods		US PPP $204.0 per capita per year	↓	-19.6	-245.7***	-256.7*	26.9	41.4**	-10.8	52.1***	88.1***	79.8***	0.0000	***
	**4**	% Population using safely managed drinking water services (SDG 6.1.1)		66.3% population	↑	4.2	42.2***	42.9***	13.9*	5.3	41.4***	-16	-24.3	-69.3***	0.0000	***
Food security	**5**	Prevalence of Undernourishment (SDG 2.1.1)		9.4% population	↓	17.5	73.1***	25.5	11.3	61.9***	66.3***	37***	-62.9***	-116.7***	0.0000	***
	**6**	% Population experiencing moderate or severe food insecurity (SDG 2.1.2)		29.5% population	↓	-10.8	74.4***	56.5***	1.7	48.9*	84.3***	36.4	-21.2	-104.1***	0.0000	***
	**7**	% Population who cannot afford a healthy diet		42.3% population	↓	46.5***	95.6***	93.6***	2.9	48.9	74***	-27.5	-65.6***	-100.8***	0.0000	***
Diet quality	**8**	MDD-W: minimum dietary diversity for women		65.7% population, women 15-49	↑	24.9***	9.3***		14.3***	33.6***	31.3***	28.5***	-31.6***	-20.2**	0.0001	--
	**9**	MDD (IYCF): minimum dietary diversity for infants and young children		31.8% population, 6-23 months	↑	95.4***	122.9***	1.1	10.7	14.7	16.7***	66.5***	-40.2***	-27.4***	0.0000	***
	**10**	All-5: consumption of all 5 food groups		39.0% adult population (≥15 y)	↑	20.7	-1.8		6.9	-3.2	39***	27.4	-29.2***	-35.8***	0.0150	--
	**11**	Zero fruit or vegetable consumption	Adults	10.8% adult population (≥15 y)	↓	53.1***	49.3***		38.7***	71.1***	65.3***	59.6***	-84.7***	-26.1	0.0004	--
			Children 6-23 months	39.1% population, 6-23 months	↓	50***	81.7***	66.4***	-9.3	13.1	24.8***	45.1***	-38***	-8.9	0.0001	***
	**12**	NCD-Protect		3.8 points (out of 9)	↑	15.2	-2		-5.7	-2.6	20.4***	14.4*	-14***	-19.7***	0.0045	--

Domain		Indicator		Global weighted mean	Dir. of Δ	Latin America & Caribbean	Northern America & Europe	Oceania	Northern Africa & Western Asia	Central Asia	Eastern Asia	South-eastern Asia	Southern Asia	Sub-Saharan Africa	Joint signif. (F-test) p-val (sig)[§]	
						% Deviation of regional weighted mean from global weighted mean, sign aligned to desirable direction of change										
	**13**	NCD-Risk		2.1 points (out of 9)	↓	-29.1***	-46.6***		12.4	-68***	-12.1***	-40.9***	30.6***	17.1	0.0000	--
	**14**	Sugar-sweetened soft drink consumption		18.9% adult population (≥15 y)	↓	-91.4***	-76.9*		-31**	-77***	38.9***	-9.8	24.6**	-44*	0.0248	--
Environment, natural resources, and production																
Greenhouse gas emissions	**16**	Food systems greenhouse gas emissions		82,463.9 kt CO_2eq (AR5)	↓	3.6	6.4	72.6***	63.3***	62.5***	-436.5	-102	-215.6	40.9*	0.0051	***
Greenhouse gas emissions	**16**	Greenhouse gas emissions intensity, by product group[$]	Cereals (excl. rice)[†]	0.2 kg CO_2eq/ kg product	↓	10	8.2	-51.2***	-30.9**	15	-2.7***	4.5	-37.6***	17.3**	0.8091	***
Greenhouse gas emissions	**16**	Greenhouse gas emissions intensity, by product group[$]	Beef	30.3 kg CO_2eq/ kg product	↓	-34.4***	49.9***	30.2***	30.9	44.6***	49.6***	-75.6***	-108.8	-147*	0.0000	***
Greenhouse gas emissions	**16**	Greenhouse gas emissions intensity, by product group[$]	Cow's milk	1.0 kg CO_2eq/ kg product	↓	-4.5	38.1***	17.7**	-6	-18.7	13.5	-185.3	-35.1**	-288***	0.0000	***
Greenhouse gas emissions	**16**	Greenhouse gas emissions intensity, by product group[$]	Rice	1.1 kg CO_2eq/ kg product	↓	12.5	-75.9	-33.3	-6.7	-166.8	16.3***	-33.9*	20.4***	-46.8*	0.9187	***
Production	**17**	Food product yield, by food group[$]	Cereals[†]	4.1 tonnes/ha	↑	16.3*	32.5	-58.2***	-43.8**	-59***	53.2***	6.2	-19***	-59.8***	0.0001	***
Production	**17**	Food product yield, by food group[$]	Fruit[†]	13.7 tonnes/ha	↑	24.9**	-5.6	-2.5	1.8	-1.8	18.7***	3.1	2.3	-43.5***	0.0050	***
Production	**17**	Food product yield, by food group[$]	Beef	231.5 kg/animal	↑	18.2	37.3**	0.7	-17.7	-19.9***	-31.9***	-8.7	-44.2***	-35.9***	0.0000	***
Production	**17**	Food product yield, by food group[$]	Cow's milk	2676.6 kg/animal	↑	-6.9	184.1***	82.6***	-29.9	-15.8	14.3	-59.5***	-41.1***	-81.4***	0.0000	***
Production	**17**	Food product yield, by food group[$]	Vegetables[†]	19.7 tonnes/ha	↑	-5.2	45.9**	2.9	27.1	74.2**	29.9***	-40.5***	-21.7***	-71***	0.0000	***
Land	**18**	Cropland change, 5-year average (%)		0.1%	↓	178.3	101.5*	251.5***	-44.4	82.5*	136.4***	-552.7	11	-177.7	0.5710	***
Water	**19**	Agriculture water withdrawal as % of total renewable water resources		16.9% total renewable	↓	78.6***	80.3***	86.2***	-474.1*	-86.8	20.5***	49.5***	-139.8***	75***	0.0011	***
Biosphere integrity	**20**	Functional integrity: % agricultural land with minimum level of natural habitat		88.3% agricultural land	↑	7.2***	-3.4	9***	5.8	3.1***	-0.4	-7.1	-30.1*	2.6	0.0001	***
Biosphere integrity	**21**	Fishery health index progress score		21.4	↑	13.2	79.4**	27.9***	-36*		-42.7***	-35.9*	27.4***	-42.3*	0.0000	***
Pollution	**22**	Total pesticides per unit of cropland		1.8 kg active ingredient/ ha	↓	-195.9***	-10.7	-20.1*	29	58.5**	-30.9	46.7	76.1***	77.4***	0.0001	***
Pollution	**23**	Sustainable nitrogen management index		0.7	↑	-20	-12.5	16.1***	22.1	9.7**	-5.4**	-6.4	10.7*	24.3***	0.0003	***
Livelihoods, Poverty, & Equity																
Poverty and income	**24**	Share of agriculture in GDP		4.4% GDP	↓	-32.1**	69.1***	36.8	-19.2	-151.1	-30.2	-143.9***	-305***	-315.3**	0.0000	***
Employment	**25**	Unemployment, rural		5.7% working age population	↓	-11.7	-15.5	29.3*	-77.4***	21.6**	34.1***	63.4***	-20.1	4.4	0.0659	***

Domain		Indicator	Global weighted mean	Dir. of Δ	% Deviation of regional weighted mean from global weighted mean, sign aligned to desirable direction of change										
					Latin America & Caribbean	Northern America & Europe	Oceania	Northern Africa & Western Asia	Central Asia	Eastern Asia	South-eastern Asia	Southern Asia	Sub-Saharan Africa	Joint signif. (F-test) p-val (sig)§	
	**26**	Underemployment rate, rural	7.3% working age population	↓	-45.1***	60.2***	-16.9***	71.9***	90	59.9***	6.8	61.8***	-116*	0.0006	***
Social protection	**27**	Social protection coverage	55.8% population	↑	-14.1*	32***	-86.7***	-18.8	-34.2**	13***	-23.3**	38.4	-59.7***	0.0000	***
	**28**	Social protection adequacy	21.0% welfare of beneficiary households	↑	55.6**	79.9*	-81.9***	-0.3	32.5	75.3***	-46.1*	-60.7***	-20	0.0000	***
Rights	**29**	% Children 5-17 engaged in child labor	9.4% children 5-17	↓	39.4**	65.1***	-52.6***	54.4**	-13.8	52.5***	45.3*	43.4***	-134.9***	0.0000	***
	**30**	Female share of landholdings	16.8% landholdings by sex of operator	↑	-11.5	13.2	-99.4***	-3.4	-47.4***	-15.9***	9.7	-59.5**	-13.3	0.5166	--
Governance															
Shared vision and strategic planning	**31**	Civil society participation index	0.6	↑	7.9	37.6**	29.5***	-37.6***	-42.4***	-41.3***	14.5	5.8	18.4***	0.0000	***
	**32**	% Urban population living in cities signed onto the Milan Urban Food Policy Pact	7.2% urban population	↑	250.7**	66.9*	-76.4***	-65.5***	-58.4*	16	-70.9***	-91.3***	16.4	0.0143	***
	**33**	Degree of legal recognition of the Right to Food (1 = Explicit protection or directive principle of state policy 2= Other implicit or national codification of international obligations or relevant provisions 3 = None)	1.9	↓	4.5	-4.7	-33.1**	-9.3*	-4.7***	5.7	-4.7	34.3***	8.3*	0.0001	--
	**34**	Presence of a national food system transformation pathway (0 = No, 1 = yes)	0.6	↑	-18.2	-35.5**	34.6	-0.2	31.6	31.6	15	23.3	23.3*	0.0059	
Effective implemen-tation	**35**	Government effectiveness index	0.1	↑	-380.3***	637.4***	683.9	-549.3***	-438**	467.3***	51.5	-55	-756.9***	0.0000	***
	**36**	International Health Regulations State Party Assessment report (IHR SPAR), Food safety capacity	69.4	↑	22.3**	27.7***	18.8	4	-38.9	17.9***	-0.4	-17.6***	-35.3***	0.0000	***
	**37**	Presence of health-related food taxes	0.3	↑	1.1	-18.7	-87.3***	-47.6	-100***	-100***	4.2	137	-47.5	0.1449	--

Domain		Indicator		Global weighted mean	Dir. of Δ	% Deviation of regional weighted mean from global weighted mean, sign aligned to desirable direction of change										
						Latin America & Caribbean	Northern America & Europe	Oceania	Northern Africa & Western Asia	Central Asia	Eastern Asia	South-eastern Asia	Southern Asia	Sub-Saharan Africa	Joint signif. (F-test) p-val (sig)[§]	
Accountability	38	V-Dem Accountability index		0.3	↑	229.5***	337***	427.5***	-187.1***	-259.1***	-389.7**	31.5	59	62.5	0.0000	***
	39	Open Budget Index Score		43.1	↑	53**	55.8***	68.1***	-28.8	15.9	-41.8**	37.3**	-12.8***	-12.7	0.0000	***
	40	Guarantees for public access to information (SDG 16.10.2)		0.7	↑	9.3	43.4***	-35.6	-13.3	14.4	14.4	-29	25.3	-32**	0.0000	--
Resilience																
Exposure to shocks	41	Ratio of total damages of all disasters to GDP		0.3	↓	19.5	-43.4	27.3***	88.2***	99***	47.4***	56.4***	15.9	74.8***	0.4486	***
Resilience capacities	42	Dietary sourcing flexibility index		0.7	↑	-2.1	9.8	-3.9	9.9***	-8.2***	-1.2	-9.6	-1.6	-3.6*	0.0000	***
	43	Mobile cellular subscriptions (per 100 people)		105.5 per 100 people	↑	4.4	13.4***	-28.1**	3.1	20.2*	7.1	21.5*	-0.1	-17.4**	0.0000	***
	44	Social capital index		0.5	↑	-46.2***	16.3	28.9*	-17.9***	3	46.1***	-16.4*	-8.1*	-23.6***	0.0000	***
Agro- and food diversity	45	Proportion of agricultural land with minimum level of species diversity (crop and pasture)		22.5 % agricultural land	↑	-60.3***	-12.9	-33***	-59*	-71.2***	45.7	92.1*	96.9	40.1*	0.2297	--
	46	Number of (a) plant and (b) animal genetic resources for food and agriculture secured in either medium- or long-term conservation facilities (SDG 2.5.1)	Plants	161.4 (thousands)	↑	-35	55.9	41.9**	-88.4***	-74.9***	-63.8*	-93.1***	62.7	-92.1***	0.3821	***
	47		Animals	4.4	↑	-86.9***	30.2	-100***	-95.1***	-100***	-71.4	7.2	731.2***	-70.2***	0.0022	--
Resilience responses/ strategies	48	Coping strategies index		38.5% population	↓	-19.9			5.4				15	-2.4	0.9774	--
Long-term outcomes	49	Food price volatility		0.7	↓	-1.8	-1.9	19.1	-2.1	21.2***	4	-4	-0.6	-1.9	0.7014	***
	50	Food supply variability		29.9 kcal per capita/ day	↑	-3.2	-8.2	-22.4	6.8	28	-16.1	-12	-3.2	12.5	0.5984	--

*** p < 0.001 ** p < 0.01 * p < 0.05

[§] Reflects p-value of joint significance tests (F-test), '--' indicates insufficient observations in one or more regions to compute the F-test with cluster robust standard errors, required due to unequal variances by region.

‡ See **SI-A Table 1.2** for regional means and **SI-A Table 1.3** for regional medians.

† Product mix varies across countries.

[$] Additional products included in **SI-A Figures 1.2.3-1.2.10** and in the baseline dataset.

Sources: Author's calculations based on data sources listed in **Extended Data Table 1**.

Table 1.2 Regional weighted mean values

Domain		Indicator		Global weighted mean	Dir. of Δ	Latin America & Caribbean	Northern America & Europe	Oceania	Northern Africa & Western Asia	Central Asia	Eastern Asia	South-eastern Asia	Southern Asia	Sub-Saharan Africa
Diets, Nutrition, & Health														
Food environments	**1**	Cost of a healthy diet		US PPP $3.3 per person/day	↓	3.3	3.2	2.6	3.4	3.0	3.3	4.2	3.1	3.4
	**2**	Availability of fruits and vegetables	Fruits	223.8 grams per capita/ day	↑	308.1	241.5	269.5	232.9	156.6	142.6	174.1	157.0	169.5
			Vegetables	246.8 grams/ capita/ day	↑	184.8	327.6	154.8	370.7	575.9	473.9	223.4	213.3	123.6
	**3**	Retail value of ultra-processed foods		US PPP $204.0 per capita per year	↓	243.9	705.3	727.6	149.2	119.6	226.0	97.8	24.3	41.2
	**4**	% Population using safely managed drinking water services (SDG 6.1.1)		66.3% population	↑	69.1	94.3	94.7	75.6	69.8	93.8	55.7	50.2	20.3
Food security	**5**	Prevalence of Undernourishment (SDG 2.1.1)		9.4% population	↓	7.8	2.5	7.0	8.4	3.6	3.2	6.0	15.4	20.5
	**6**	% Population experiencing moderate or severe food insecurity (SDG 2.1.2)		29.5% population	↓	32.7	7.6	12.8	29.0	15.1	4.6	18.8	35.8	60.3
	**7**	% Population who cannot afford a healthy diet		42.3% population	↓	22.6	1.9	2.7	41.1	21.6	11.0	53.9	70.0	84.9
Diet quality	**8**	MDD-W: minimum dietary diversity for women		65.7% population, women 15-49	↑	82.1	71.8		75.1	87.7	86.2	84.4	44.9	52.4
	**9**	MDD (IYCF): minimum dietary diversity for infants and young children		31.8% population, 6-23 months	↑	62.1	70.8	32.1	35.2	36.4	37.1	52.9	19.0	23.1
	**10**	All-5: consumption of all 5 food groups		39.0% adult population (≥15 y)	↑	47.0	38.3		41.7	37.7	54.2	49.7	27.6	25.0
	**11**	Zero fruit or vegetable consumption	Adults	10.8% adult population (≥15 y)	↓	5.1	5.5		6.6	3.1	3.8	4.4	20.0	13.7
			Children 6-23 months	39.1% population, 6-23 months	↓	19.6	7.1	13.1	42.7	34.0	29.4	21.4	54.0	42.6
	**12**	NCD-Protect		3.8 points (out of 9)	↑	4.4	3.8		3.6	3.7	4.6	4.4	3.3	3.1
	**13**	NCD-Risk		2.1 points (out of 9)	↓	2.8	3.1		1.9	3.6	2.4	3.0	1.5	1.8
	**14**	Sugar-sweetened soft drink consumption		18.9% adult population (≥15 y)	↓	36.2	33.4		24.8	33.5	11.6	20.8	14.3	27.2

Domain		Indicator		Global weighted mean	Dir. of Δ	Latin America & Caribbean	Northern America & Europe	Oceania	Northern Africa & Western Asia	Central Asia	Eastern Asia	South-eastern Asia	Southern Asia	Sub-Saharan Africa
Environment, natural resources, and production														
Greenhouse gas emissions	**16**	Food systems greenhouse gas emissions		82463.9 kt CO_2eq (AR5)	↓	82584.4	80290.1	25641.1	33310.2	33971.7	445521.9	169650.5	263351.1	51790.6
	**16**	Greenhouse gas emissions intensity, by product group[$]	Cereals (excl. rice)[†]	0.2 kg CO_2eq/kg product	↓	0.2	0.2	0.3	0.2	0.2	0.2	0.2	0.2	0.1
			Beef	30.3 kg CO_2eq/kg product	↓	40.7	15.2	21.2	20.9	16.8	15.3	53.2	63.2	74.8
			Cow's milk	1.0 kg CO_2eq/kg product	↓	1.0	0.6	0.8	1.0	1.2	0.8	2.8	1.3	3.8
			Rice	1.1 kg CO_2eq/kg product	↓	1.0	1.9	1.5	1.2	2.9	0.9	1.5	0.9	1.6
Production	**17**	Food product yield, by food group[$]	Cereals[†]	4.1 tonnes/ha	↑	4.7	5.4	1.7	2.3	1.7	6.2	4.3	3.3	1.6
			Fruit[†]	13.7 tonnes/ha	↑	17.1	12.9	13.3	13.9	13.4	16.2	14.1	14.0	7.7
			Beef	231.5 kg/animal	↑	273.6	317.9	233.2	190.6	185.5	157.8	211.4	129.3	148.4
			Cow's milk	2676.6 kg/animal	↑	2491.2	7605.2	4887.0	1876.5	2254.9	3058.7	1083.3	1575.6	499.0
			Vegetables[†]	19.7 tonnes/ha	↑	18.7	28.7	20.3	25.0	34.3	25.6	11.7	15.4	5.7
Land	**18**	Cropland change, 5-year average (%)		0.1%	↓	-0.1	0.0	-0.2	0.2	0.0	-0.1	1.0	0.1	0.4
Water	**19**	Agriculture water withdrawal as % of total renewable water resources		16.9% total renewable	↓	3.6	3.3	2.3	96.9	31.5	13.4	8.5	40.5	4.2
Biosphere integrity	**20**	Functional integrity: % agricultural land with minimum level of natural habitat		88.3% agricultural land	↑	94.6	85.2	96.2	93.4	91.0	87.9	82.0	61.7	90.6
	**21**	Fishery health index progress score		21.4	↑	24.2	38.3	27.3	13.7	0.0	12.2	13.7	27.2	12.3
Pollution	**22**	Total pesticides per unit of cropland		1.8 kg active ingredient/ ha	↓	5.3	2.0	2.1	1.3	0.7	2.3	0.9	0.4	0.4
	**23**	Sustainable nitrogen management index		0.7	↑	0.6	0.6	0.8	0.9	0.8	0.7	0.7	0.8	0.9
Livelihoods, Poverty, & Equity														
Poverty and income	**24**	Share of agriculture in GDP		4.4% GDP	↓	5.8	1.4	2.8	5.2	11.0	5.7	10.7	17.7	18.2
Employment	**25**	Unemployment, rural		5.7% working age population	↓	6.4	6.6	4.0	10.1	4.5	3.8	2.1	6.9	5.5
	**26**	Underemployment rate, rural		7.3% working age population	↓	10.7	2.9	8.6	2.1		2.9	6.9	2.8	15.9
Social protection	**27**	Social protection coverage		55.8% population	↑	48.0	73.7	7.4	45.3	36.7	63.1	42.9	77.3	22.5

Domain		Indicator	Global weighted mean	Dir. of Δ	Latin America & Caribbean	Northern America & Europe	Oceania	Northern Africa & Western Asia	Central Asia	Eastern Asia	South-eastern Asia	Southern Asia	Sub-Saharan Africa
	**28**	Social protection adequacy	21.0% welfare of beneficiary households	↑	32.7	37.8	3.8	20.9	27.8	36.8	11.3	8.2	16.8
Rights	**29**	% Children 5-17 engaged in child labor	9.4% children 5-17	↓	5.7	3.3	14.3	4.3	10.6	4.4	5.1	5.3	22.0
	**30**	Female share of landholdings	16.8% landholdings by sex of operator	↑	18.4	22.6	3.6	19.8	12.4	17.7	22.0	10.4	18.1
Governance													
Shared vision and strategic planning	**31**	Civil society participation index	0.6	↑	0.7	0.8	0.8	0.4	0.3	0.4	0.7	0.6	0.7
	**32**	% Urban population living in cities signed onto the Milan Urban Food Policy Pact	7.2% urban population	↑	25.4	12.1	1.7	2.5	3.0	8.4	2.1	0.6	8.4
	**33**	Degree of legal recognition of the Right to Food (1 = Explicit protection or directive principle of state policy 2= Other implicit or national codification of international obligations or relevant provisions 3 = None)	1.9	↑	1.8	2.0	2.5	2.1	2.0	1.8	2.0	1.3	1.8
	**34**	Presence of a national food system transformation pathway (0 = No, 1 = yes)	0.6	↑	0.5	0.4	0.8	0.6	0.8	0.8	0.7	0.8	0.8
Effective implemen-tation	**35**	Government effectiveness index	0.1	↑	-0.4	0.9	1.0	-0.6	-0.4	0.7	0.2	0.1	-0.8
	**36**	International Health Regulations State Party Assessment report (IHR SPAR), Food safety capacity	69.4	↑	84.9	88.7	82.5	72.2	42.4	81.9	69.2	57.2	44.9
	**37**	Presence of health-related food taxes	0.3	↑	0.3	0.2	0.0	0.2	0.0	0.0	0.3	0.7	0.2
Accountability	**38**	V-Dem Accountability index	0.3	↑	0.9	1.2	1.5	-0.2	-0.4	-0.8	0.4	0.4	0.5
	**39**	Open Budget Index Score	43.1	↑	65.9	67.1	72.4	30.7	49.9	25.1	59.1	37.5	37.6

Domain		Indicator		Global weighted mean	Dir. of Δ	Latin America & Caribbean	Northern America & Europe	Oceania	Northern Africa & Western Asia	Central Asia	Eastern Asia	South-eastern Asia	Southern Asia	Sub-Saharan Africa
	**40**	Guarantees for public access to information (SDG 16.10.2)		0.7	↑	0.8	1.0	0.5	0.6	0.8	0.8	0.5	0.9	0.5
Resilience														
Exposure to shocks	**41**	Ratio of total damages of all disasters to GDP		0.3	↓	0.2	0.4	0.2	0.0	0.0	0.1	0.1	0.2	0.1
Resilience capacities	**42**	Dietary sourcing flexibility index		0.7	↑	0.7	0.7	0.6	0.7	0.6	0.7	0.6	0.7	0.7
	**43**	Mobile cellular subscriptions (per 100 people)		105.5 per 100 people	↑	110.6	120.2	76.3	109.3	127.3	113.5	128.7	105.9	87.6
	**44**	Social capital index		0.5	↑	0.3	0.6	0.6	0.4	0.5	0.7	0.4	0.4	0.4
Agro- and food diversity	**45**	Proportion of agricultural land with minimum level of species diversity (crop and pasture)		22.5 % agricultural land	↑	8.9	19.6	15.1	9.2	6.5	32.8	43.2	44.3	31.5
	**46**	Number of (a) plant and (b) animal genetic resources for food and agriculture secured in either medium- or long-term conservation facilities (SDG 2.5.1)	Plants	161.4 (thousands)	↑	105.0	251.6	229.1	18.7	40.5	58.4	11.1	262.7	12.8
	**47**		Animals	4.4	↑	0.6	5.7	0.0	0.2	0.0	1.3	4.7	36.5	1.3
Resilience responses/ strategies	**48**	Coping strategies index		38.5% population	↓	46.1			36.4				32.7	39.4
Long-term outcomes	**49**	Food price volatility		0.7	↓	0.8	0.8	0.6	0.8	0.6	0.7	0.8	0.7	0.8
	**50**	Food supply variability		29.9 kcal per capita/ day	↑	28.8	27.4	23.1	31.9	38.2	25.0	26.2	28.9	33.6

† Product mix varies across countries.
$ Additional products included in **SM Figures 1.2.3-1.2.10** and in the baseline dataset.
Sources: Author's calculations based on data sources listed in **Extended Data Table 1**.

Table 1.3 Regional median values

Domain	Indicator		Unit	Latin America & Caribbean	Northern America & Europe	Oceania	Northern Africa & Western Asia	Central Asia	Eastern Asia	South-eastern Asia	Southern Asia	Sub-Saharan Africa
Diets, nutrition, and health												
Food environments	Cost of a healthy diet		current PPP dollar/ person/ day	3.7	3.1	2.7	3.3	3.2	5.1	4.1	3.8	3.4
	Availability of fruits and vegetables	Fruits	grams/ capita/ day	264.7	221.9	189.6	233.6	135.1	143.0	189.0	129.0	120.4
		Vegetables	grams/ capita/ day	159.9	264.1	146.7	331.5	630.5	334.6	173.7	179.8	93.1
	Retail value of ultra-processed foods		current (nominal) US$/capita/ year	196.4	496.1	125.4	176.8	90.7	156.0	107.6	21.3	31.0
	% Population using safely managed drinking water services (SDG 6.1.1)		% population	66.8	97.6	46.2	86.9	70.1	82.5	53.1	36.2	21.1
Food security	% Population experiencing moderate or severe food insecurity (SDG 2.1.2)		% population	7.5	2.5	5.1	5.1	3.5	3.2	5.8	11.4	19.3
	% Population who cannot afford a healthy diet		% population	33.1	7.4	23.2	27.6	6.6	5.3	25.5	32.6	61.1
	PoU: Prevalence of Undernourishment (SDG 2.1.1)		% population	21.0	0.9	30.6	16.7	42.1	7.3	65.1	61.8	83.5
Diet quality	MDD-W: minimum dietary diversity for women		% population, women 15-49	83.7	71.8		75.6	87.5	86.2	79.9	58.8	50.9
	MDD (IYCF): minimum dietary diversity for infants and young children		% population, 6-23 months	59.3	67.8	33.2	35.6	49.0	45.0	47.0	28.0	22.8
	All-5: consumption of all 5 food groups		% adult population (≥15 y)	46.2	29.2		38.9	44.0	54.2	36.4	28.2	23.3
	Zero fruit or vegetable consumption	Adults	% adult population (≥15 y)	4.6	5.3		6.9	2.8	3.8	5.1	12.7	12.1
		Children 6-23 months	% population 6-23 months	18.3	10.1	29.6	32.1	21.2	37.4	27.9	49.8	42.7
	NCD-Protect		Score (points out of 9)	4.1	3.3		3.5	3.8	4.6	3.9	3.4	3.0
	NCD-Risk		Score (points out of 9)	2.7	2.9		2.0	3.4	2.4	2.8	1.5	1.5

Domain	Indicator		Unit	Latin America & Caribbean	Northern America & Europe	Oceania	Northern Africa & Western Asia	Central Asia	Eastern Asia	South-eastern Asia	Southern Asia	Sub-Saharan Africa
	Sugar-sweetened soft drink consumption		% adult population (≥15 y)	38.0	24.1		24.5	33.3	11.6	24.6	12.7	19.7
Environment, natural resources, and production												
Greenhouse gas emissions	Food systems greenhouse gas emissions		kt CO_2eq (AR5)	17,024.2	20,674.9	237.7	15,327.3	22,024.1	87,730.7	92,989.9	36,144.2	21,595.8
	Greenhouse gas emissions intensity, by product group	Cereals (excl. rice)	kg CO2eq/ kg product	0.3	0.2	0.2	0.3	0.2	0.2	0.2	0.1	0.2
		Beef	kg CO_2eq/ kg product	49.7	16.7	68.5	11.6	15.8	22.5	58.7	33.2	66.9
		Cow’s milk	kg CO_2eq/ kg product	1.7	0.6	3.5	0.8	1.3	0.9	3.2	1.6	4.2
		Rice	kg CO_2eq/ kg product	1.2	3.2	3.6	2.6	2.4	1.0	1.5	0.9	1.4
Production	Food product yield, by food group	Cereals	tonnes/ha	3.5	5.2	1.9	2.6	3.3	6.1	3.7	3.2	1.4
		Fruit	tonnes/ha	15.1	9.8	8.6	12.1	11.8	15.5	10.2	11.1	6.8
		Beef	kg/animal	193.8	271.8	157.2	200.5	180.8	150.0	199.9	116.6	145.4
		Cow’s milk	kg/animal	1,481.0	7,040.4	1,187.0	2,305.5	1,950.9	2,839.8	953.0	1,305.3	448.5
		Vegetables	tonnes/ha	13.3	24.9	11.9	25.4	29.1	25.6	8.8	12.8	7.1
Land	Cropland change, 5-year average (%))		%	0.0	0.0	0.0	0.3	0.1	-0.3	0.1	0.3	0.0
Water	Agriculture water withdrawal as % of total renewable water resources		% total renewable	1.2	0.4	0.6	65.9	33.7	12.5	4.4	21.4	1.0
Biosphere integrity	Functional integrity: % agricultural land with minimum level of natural habitat		% agricultural land	98.6	80.1	98.9	92.3	91.6	85.4	82.4	76.7	94.1
	Fishery health index progress score		index	23.0	32.5	23.0	18.6	0.0	13.6	9.3	24.4	12.6
Pollution	Total pesticides per unit of croplands		Kg active ingredient/ ha	4.8	2.1	2.0	2.0	0.5	6.2	0.9	0.4	0.1
	Sustainable nitrogen management index		index	0.8	0.8	0.9	1.0	0.7	0.8	0.7	0.8	0.9
Livelihoods, poverty, and equity												
Poverty and income	Share of agriculture in GDP		% GDP	6.7	2.1	17.6	6.1	13.5	7.4	13.7	17.2	18.5

Domain	Indicator	Unit	Latin America & Caribbean	Northern America & Europe	Oceania	Northern Africa & Western Asia	Central Asia	Eastern Asia	South-eastern Asia	Southern Asia	Sub-Saharan Africa
Employment	Unemployment, rural	% working age population	5.5	6.3	3.2	8.4	4.8	2.9	1.9	4.9	4.0
	Underemployment rate, rural	% working age population	8.9	3.3	7.1	4.1	0.0	1.8	2.2	3.0	6.5
Social protection	Social protection coverage	% population	52.0	71.5	13.4	54.6	44.2	78.5	35.3	31.3	20.7
	Social protection adequacy	% welfare of beneficiary households	27.7	38.4	21.6	14.0	30.3	30.0	17.7	12.6	13.6
Rights	% Children 5-17 engaged in child labor	% children 5-17	3.6	3.4	13.4	1.9	10.2	6.0	7.7	5.9	17.5
	Female share of landholdings	% landholdings by sex of operator	25.2	17.3	19.4	14.6	12.4	15.8	12.9	15.8	20.3
Governance											
Shared vision and strategic planning	Civil society participation index	index	0.7	0.8	0.7	0.5	0.3	0.6	0.5	0.7	0.7
	% Urban population living in cities signed onto the Milan Urban Food Policy Pact ⁺	% urban population	0.0	0.4	0.0	0.0	0.0	15.6	0.0	0.0	0.0
	Degree of legal recognition of the Right to Food ⁺	Categorical	2.0	2.0	3.0	2.0	2.0	2.0	2.0	1.0	2.0
	Presence of a national food system transformation pathway	Binary	0.5	0.0	1.0	1.0	1.0	1.0	1.0	1.0	1.0
Effective implementation	Government effectiveness index	index	-0.2	1.0	-0.1	-0.1	-0.5	0.6	0.2	-0.5	-0.8
	International Health Regulations State Party Assessment report (IHR SPAR), Food safety capacity	Score	80.0	80.0	80.0	80.0	40.0	80.0	70.0	60.0	40.0
	Presence of health-related food taxes ⁺	Binary	0.0	0.0	0.0	0.0	0.0	0.0	0.0	0.0	0.0
Accountability	V-Dem Accountability index	index	1.0	1.5	1.2	-0.2	-0.5	1.0	0.1	0.6	0.4
	Open Budget Index Score	index	50.0	65.0	64.5	42.0	62.0	60.5	49.5	38.0	31.0
	Guarantees for public access to	Binary	1.0	1.0	0.0	1.0	1.0	1.0	0.0	1.0	0.0

Domain	Indicator		Unit	Latin America & Caribbean	Northern America & Europe	Oceania	Northern Africa & Western Asia	Central Asia	Eastern Asia	South-eastern Asia	Southern Asia	Sub-Saharan Africa
	information (SDG 16.10.2)											
Resilience												
Exposure to shocks	Ratio of total damages of all disasters to GDP		Ratio	0.0	0.0	0.0	0.0	0.0	0.1	0.0	0.0	0.0
Resilience capacities	Dietary sourcing flexibility index		index	0.7	0.8	0.6	0.8	0.6	0.7	0.6	0.7	0.6
	Mobile cellular subscriptions (per 100 people)		Number per 100 people	108.8	120.2	72.7	103.9	129.4	133.1	135.1	107.0	87.5
	Social capital index		index	0.3	0.5	0.7	0.4	0.5	0.6	0.5	0.4	0.4
Agro- and Food Diversity	Proportion of agricultural land with minimum level of species diversity (crop and pasture) ⁺		% agricultural land	13.2	0.0	12.2	0.1	12.2	39.9	30.9	26.3	22.8
	Number of (a) plant and (b) animal genetic resources for food and agriculture secured in either medium- or long-term conservation facilities (SDG 2.5.1)	Plants	thousands	6.0	12.0	36.1	13.2	4.8	122.0	12.1	12.4	3.6
		Animals	number	0.0	1.5	0.0	0.0	0.0	0.0	0.5	19.0	0.0
Resilience responses/ strategies	Coping strategies index		% population	45.0			55.6				43.4	38.4
Long-term outcomes	Food price volatility ⁺		unitless	0.7	0.7	0.7	0.7	0.6	0.7	0.8	0.7	0.8
	Food supply variability		kcal/ capita/ day	27.0	26.0	23.5	27.0	31.0	18.0	27.5	31.0	28.5

⁺ Indicates FSCI value-added to existing data

Sources: Author's calculations based on data sources listed in **Extended Data Table 1**.

Table 1.4. Income group deviation from global weighted means, by desirable direction of change‡

Domain		Indicator		Global weighted mean	Dir. of Δ	% Deviation of income group weighted mean from global weighted mean, sign aligned to desirable direction of change: Low income	Lower middle income	Upper middle income	High income	Joint signif. (F-test) p-val (sig)§	
Diets, Nutrition, & Health											
Food environments	**1**	Cost of a healthy diet		US PPP $3.3 per person/day	↓	7.5	-3.1	5	-5.2	0.0000	
	**2**	Availability of fruits and vegetables	Fruits	223.8 grams per capita/ day	↑	-27.3*	-9.5	25.7*	-0.6	0.0017	
			Vegetables	246.8 grams/ capita/ day	↑	-48.4***	-9.5	26	11	0.0000	***
	**3**	Retail value of ultra-processed foods		US PPP $204.0 per capita per year	↓	88.1***	77.8***	11.1	-293***	0.0000	***
	**4**	% Population using safely managed drinking water services (SDG 6.1.1)		66.3% population	↑	-70***	-26.9*	9	47.8***	0.0000	***
Food security	**5**	Prevalence of Undernourishment (SDG 2.1.1)		9.4% population	↓	-211.3***	-33.4	58.6***	71.5***	0.0000	***
	**6**	% Population experiencing moderate or severe food insecurity (SDG 2.1.2)		29.5% population	↓	-123.1***	-16.9	15.4	76.5***	0.0000	***
	**7**	% Population who cannot afford a healthy diet		42.3% population	↓	-108.9***	-63.9***	63.8***	96.3***	0.0000	***
Diet quality	**8**	MDD-W: minimum dietary diversity for women++		65.7% population, women 15-49	↑	-22.3**	-17.2	27.4***		0.0001	***
	**9**	MDD (IYCF): minimum dietary diversity for infants and young children		31.8% population, 6-23 months	↑	-32.5***	-14.7	34.6*	119.7***	0.0000	
	**10**	All-5: consumption of all 5 food groups		39.0% adult population (≥15 y)	↑	-29.7**	-18.5	27.2*	13.5***	0.0150	--
	**11**	Zero fruit or vegetable consumption	Adults	10.8% adult population (≥15 y)	↓	5	-45.2	58.8***	50.3***	0.0004	***
			Children 6-23 months	39.1% population, 6-23 months	↓	-19.1	-15.4	30.9***	85.9***	0.0001	
	**12**	NCD-Protect		3.8 points (out of 9)	↑	-13.1*	-10*	15.3**	3.9***	0.0045	--
	**13**	NCD-Risk		2.1 points (out of 9)	↓	40.7***	15.8	-16.5***	-51.3***	0.0000	***
	**14**	Sugar-sweetened soft drink consumption		18.9% adult population (≥15 y)	↓	-2.7	6.6	10.2	-105***	0.0248	***
Environment, natural resources, and production											
Greenhouse gas emissions	**16**	Food systems greenhouse gas emissions		82,463.9 kt CO_2eq (AR5)	↓	27.1	-7.1	-38.2	25.6	0.0051	
	**16**	Greenhouse gas emissions intensity, by product group$	Cereals (excl. rice)†	0.2 kg CO_2eq/ kg product	↓	24.5**	-20.1	5.1	2.3	0.8091	
			Beef	30.3 kg CO_2eq/ kg product	↓	-213.1***	-62.2	0.1	45.7***	0.0000	***
			Cow's milk	1.0 kg CO_2eq/ kg product	↓	-354.2***	-46.3**	8.8	40.5***	0.0000	***
			Rice	1.1 kg CO_2eq/ kg product	↓	-40.7	-0.4	5.9	-22.8	0.9187	
Production	**17**	Food product yield, by food group$	Cereals†	4.1 tonnes/ha	↑	-645.6***	-199.9**	135.3	456.2	0.0001	***
			Fruit†	136.7 tonnes/ha	↑	-51.4***	-5.4	15.4***	5.2	0.0050	***

Domain		Indicator	Global weighted mean	Dir. of Δ	% Deviation of income group weighted mean from global weighted mean, sign aligned to desirable direction of change: Low income	Lower middle income	Upper middle income	High income	Joint signif. (F-test) p-val (sig)[§]	
		Beef	231.5 kg/animal	↑	-46.9***	-31.5***	-2.3	38.2***	0.0000	***
		Cow's milk	2676.6 kg/animal	↑	-83.9***	-43.9***	11.2	193.1***	0.0000	***
		Vegetables[†]	19.7 tonnes/ha	↑	-52.7***	-34.3**	25.7***	65.2***	0.0000	***
Land	18	Cropland change, 5-year average (%)	0.1%	↓	-284.4*	-148.2	125.8	142.9*	0.5710	
Water	19	Agriculture water withdrawal as % of total renewable water resources	16.9% total renewable	↓	-8.3	-49.3	34.2	21.5	0.0011	
Biosphere integrity	20	Functional integrity: % agricultural land with minimum level of natural habitat	88.3% agricultural land	↑	5.1**	-11.4	2.3	3.5	0.0001	**
	21	Fishery health index progress score	21.4	↑	-56.8***	-1.5	-22.9	67.6*	0.0000	***
Pollution	22	Total pesticides per unit of cropland	1.8 kg active ingredient/ ha	↓	89.5***	65.5***	-38.9	-57.1***	0.0001	***
	23	Sustainable nitrogen management index	0.7	↑	31.7***	4.7	-5	-13.2	0.0003	***
Livelihoods, Poverty, & Equity										
Poverty and income	24	Share of agriculture in GDP	4.4% GDP	↓	-486.5***	-278***	-54.5***	70.9***	0.0000	***
Employment	25	Unemployment, rural	5.7% working age population	↓	11.9	-0.1	-5	2.8	0.0659	
	26	Underemployment rate, rural	7.3% working age population	↓	-105.2	-10.7	5.5	55.8***	0.0006	**
Social protection	27	Social protection coverage	55.8% population	↑	-74.9***	7.1	9.3*	37.1**	0.0000	***
	28	Social protection adequacy	21.0% welfare of beneficiary households	↑	-23.5	-48.6***	62.8***	125.1**	0.0000	***
Rights	29	% Children 5-17 engaged in child labor	9.4% children 5-17	↓	-123.2*	10.4	55.5***	74.7***	0.0000	***
	30	Female share of landholdings	16.8% landholdings by sex of operator	↑	-28.7*	-38.4**	36.8	8	0.5166	
Governance										
Shared vision and strategic planning	31	Civil society participation index	0.6	↑	-1.6	8	-29.9*	40.9***	0.0000	***
	32	% Urban population living in cities signed onto the Milan Urban Food Policy Pact	7.2% urban population	↑	-38.7*	-68.9***	68	78.1**	0.0143	***
	33	Degree of legal recognition of the Right to Food (1 = Explicit protection or directive principle of state policy 2= Other implicit or national codification of international obligations or relevant provisions 3 = None)	1.9	↓	12*	8	1.6	-15.1***	0.0001	***
	34	Presence of a national food system transformation pathway (0 = No, 1 = yes)	0.6	↑	5.5	27.2**	-2.9	-22.7*	0.0059	
Effective implementation	35	Government effectiveness index	0.1	↑	-1010.6***	-186.2	138.8	856.3***	0.0000	***
	36	International Health Regulations State Party Assessment report (IHR SPAR), Food safety capacity	69.4	↑	-40.7***	-16***	18.3***	30***	0.0000	***
	37	Presence of health-related food taxes	0.3	↑	-44.8	53.1	-54.4	-6.7	0.1449	

Domain		Indicator		Global weighted mean	Dir. of Δ	% Deviation of income group weighted mean from global weighted mean, sign aligned to desirable direction of change: Low income	Lower middle income	Upper middle income	High income	Joint signif. (F-test) p-val (sig)§
Accountability	**38**	V-Dem Accountability index		0.3	↑	-105.9*	55	-236.7	406.8***	0.0000 ***
	**39**	Open Budget Index Score		43.1	↑	-41.2**	-1.7	-8.3	50.7***	0.0000 ***
	**40**	Guarantees for public access to information (SDG 16.10.2)		0.7	↑	-18.7	-16.7	-1	27.6***	0.0000 ***
Resilience										
Exposure to shocks	**41**	Ratio of total damages of all disasters to GDP		0.3	↓	30.2	36.4*	53.8***	-31.6	0.4486 ***
Resilience capacities	**42**	Dietary sourcing flexibility index		0.7	↑	-7**	-2.3	-2.7	13.8*	0.0000 **
	**43**	Mobile cellular subscriptions (per 100 people)		105.5 per 100 people	↑	-37.4***	-6.8	6.2	20.9***	0.0000 ***
	**44**	Social capital index		0.5	↑	-20.5***	-14.6**	15.2	21.9**	0.0000 ***
Agro- and food diversity	**45**	Proportion of agricultural land with minimum level of species diversity (crop and pasture)		22.5 % agricultural land	↑	61.5*	42.9	-2.5	-54.2**	0.2297
	**46**	Number of (a) plant and (b) animal genetic resources for food and agriculture secured in either medium- or long-term conservation facilities (SDG 2.5.1)	Plants	161.4 (thousands)	↑	-89.7***	-47.3	-17	70	0.3821 ***
	**47**		Animals	4.4	↑	-83.3***	106.2	-78.8***	27.9	0.0022 ***
Resilience responses/ strategies	**48**	Coping strategies index		38.5% population	↓	-7.2	4.5	5.8	0***	0.9774
Long-term outcomes	**49**	Food price volatility		0.7	↓	-6.5	9.5	-0.7	-6.1	0.7014
	**50**	Food supply variability		29.9 kcal per capita/ day	↑	12.4	0.3	-7	-6	0.5984

*** p < 0.001 ** p < 0.01 * p < 0.05

§ Reflects p-value of joint significance tests (two-sided F-test), '--' indicates insufficient observations in one or more income groups to compute the F-test with cluster robust standard errors, required due to unequal variances by income group.

‡ See **SI-A Table 1.5** for income group weighted means and **SI-A Table 1.6** for income group medians.

++ Note that MDD-W has not been validated in HICs.

† Product mix varies across countries.

$ Additional products are included in the **SI-A** and baseline dataset.

+ Indicates FSCI value-added to existing dat1.

Sources: Author's calculations based on data sources listed in **Extended Data Table 1**.

Table 1.5 Income group weighted means[‡]

Domain		Indicator		Unit	Low income	Lower middle income	Upper middle income	High income	Global
Diets, nutrition & health									
Food environments	**1**	Cost of a healthy diet		current PPP dollar/ person/ day	3.1	3.4	3.2	3.5	3.3
	**2**	Availability of fruits and vegetables	Fruits	grams/capita/day	162.8	202.6	281.5	222.5	223.8
			Vegetables	grams/capita/day	127.5	223.7	311.3	274.2	246.8
	**3**	Retail value of ultra-processed foods		current (nominal) US$/capita/year	24.4	45.2	181.4	801.6	204.0
	**4**	% Population using safely managed drinking water services (SDG 6.1.1)		% population	19.9	48.5	72.3	98.0	66.3
Food security	**5**	Prevalence of Undernourishment (SDG 2.1.1)		% population	29.4	12.6	3.9	2.7	9.4
	**6**	% Population experiencing moderate or severe food insecurity (SDG 2.1.2)		% population	65.9	34.6	25.0	7.0	29.5
	**7**	% Population who cannot afford a healthy diet		% population	88.3	69.3	15.3	1.6	42.3
Diet quality	**8**	MDD-W: minimum dietary diversity for women		% population, women 15-49	51.0	54.4	83.7		65.7
	**9**	MDD (IYCF): minimum dietary diversity for infants and young children		% population, 6-23 months	21.4	27.1	42.8	69.8	31.8
	**10**	All-5: consumption of all 5 food groups		% adult population (≥15 y)	27.4	31.8	49.6	44.2	39.0
	**11**	Zero fruit or vegetable consumption	Adults	% adult population (≥15 y)	10.3	15.7	4.5	5.4	10.8
			Children 6-23 months	% population 6-23 months	46.6	45.1	27.0	5.5	39.1
	**12**	NCD-Protect		Score (points out of 9)	3.3	3.5	4.4	4.0	3.8
	**13**	NCD-Risk		Score (points out of 9)	1.3	1.8	2.5	3.2	2.1
	**14**	Sugar-sweetened soft drink consumption		% adult population (≥15 y)	19.4	17.7	17.0	38.8	18.9
Environment, natural resources, & production									
Greenhouse gas emissions	**15**	Food systems greenhouse gas emissions		kt CO_2eq (AR5)	63,201.0	91,359.8	117,046.8	64,454.8	82,463.9
	**16**	Greenhouse gas emissions intensity, by product group[$]	Cereals (excl. rice)[†]	kg CO_2eq/kg product	0.1	0.2	0.2	0.2	0.2
			Beef	kg CO_2eq/kg product	94.8	49.1	30.3	16.4	30.3
			Cow's milk	kg CO_2eq/kg product	4.4	1.4	0.9	0.6	1.0
			Rice	kg CO_2eq/kg product	1.6	1.1	1.0	1.4	1.1
Production	**17**	Food product yield, by food group[$]	Cereals[†]	tonnes/ha	1.4	3.3	4.6	5.9	4.1
			Fruit[†]	tonnes/ha	6.6	12.9	15.8	14.4	13.7
			Beef	kg/animal	123.0	158.6	226.2	320.0	231.5
			Cow's milk	kg/animal	430.1	1,502.8	2,976.0	7,845.7	2,676.6

Domain		Indicator	Unit	Low income	Lower middle income	Upper middle income	High income	Global
		Vegetables†	tonnes/ha	9.3	12.9	24.8	32.5	19.7
Land	**18**	Cropland change, 5-year average (%)	%	0.6	0.4	0.0	-0.1	0.1
Water	**19**	Agriculture water withdrawal as % of total renewable water resources	% total renewable	18.3	25.2	11.1	13.3	16.9
Biosphere integrity	**20**	Functional integrity: % agricultural land with minimum level of natural habitat	% agricultural land	92.8	78.2	90.3	91.3	88.3
	**21**	Fishery health index progress score	index	9.2	21.0	16.5	35.8	21.4
Pollution	**22**	Total pesticides per unit of land	kg/ha	0.2	0.6	2.5	2.8	1.8
	**23**	Sustainable nitrogen management index	index	0.9	0.7	0.7	0.6	0.7
Livelihoods, poverty & equity								
Poverty and income	**24**	Share of agriculture in GDP	% GDP	25.6	16.5	6.8	1.3	4.4
Employment	**25**	Unemployment, rural	% working age population	5.0	5.7	6.0	5.6	5.7
	**26**	Underemployment rate, rural	% working age population	15.1	8.1	6.9	3.3	7.3
Social protection	**27**	Social protection coverage	% population	14.0	59.8	61.0	76.6	55.8
	**28**	Social protection adequacy	% welfare of beneficiary households	16.0	10.8	34.2	47.2	21.0
Rights	**29**	% Children 5-17 engaged in child labor	% children 5-17 (sex specific is % children 5-17 of each sex)	20.9	8.4	4.2	2.4	9.4
	**30**	Female share of landholdings	% landholdings by sex of operator	14.4	13.1	18.3	18.7	16.8
Governance								
Shared vision and strategic planning	**31**	Civil society participation index	index	0.6	0.7	0.4	0.9	0.6
	**32**	% Urban population living in cities signed onto the Milan Urban Food Policy Pact +	% urban population	4.4	2.2	12.2	12.9	7.2
	**33**	Degree of legal recognition of the Right to Food +	categorical	1.7	1.8	1.9	2.2	1.9
	**34**	Presence of a national food system transformation pathway	binary	0.6	0.8	0.6	0.5	0.6
Effective implementation	**35**	Government effectiveness index	index	-1.1	-0.1	0.3	1.2	0.1
	**36**	International Health Regulations State Party Assessment report (IHR SPAR), Food safety capacity	score	41.2	58.3	82.2	90.3	69.4
	**37**	Presence of health-related food taxes +	binary	0.2	0.5	0.1	0.3	0.3
Accountability	**38**	V-Dem Accountability index	index	0.0	0.4	-0.4	1.4	0.3
	**39**	Open Budget Index Score	index	25.3	42.3	39.5	64.9	43.1
	**40**	Guarantees for public access to information (SDG 16.10.2)	binary	0.6	0.6	0.7	0.9	0.7

Domain		Indicator		Unit	Low income	Lower middle income	Upper middle income	High income	Global
Resilience									
Exposure to shocks	**41**	Ratio of total damages of all disasters to GDP		ratio	0.2	0.2	0.1	0.4	0.3
Resilience capacities	**42**	Dietary sourcing flexibility index		index	0.6	0.7	0.7	0.8	0.7
	**43**	Mobile cellular subscriptions (per 100 people)		number per 100 people	66.5	98.8	112.5	128.1	105.5
	**44**	Social capital index		index	0.4	0.4	0.6	0.6	0.5
Agro- and Food Diversity	**45**	Proportion of agricultural land with minimum level of species diversity (crop and pasture) ⁺		% agricultural land	36.4	32.2	22.0	10.3	22.5
	**46**	Number of (a) plant and (b) animal genetic resources for food and agriculture secured in either medium- or long-term conservation facilities (SDG 2.5.1)	Plants	thousands	16.7	85.0	134.0	274.5	161.4
	**47**		Animals	number	0.7	9.1	0.9	5.6	4.4
Resilience responses/ strategies	**48**	Coping strategies index		% population	41.2	36.7	36.2		38.5
Long-term outcomes	**49**	Food price volatility ⁺		unitless	0.8	0.7	0.7	0.8	0.7
	**50**	Food supply variability		kcal/capita/day	33.5	29.9	27.7	28.0	29.9

‡ See **SM Table 1.4** for income group medians.
† Product mix varies across countries.
$ Additional products are included in the **SM-A** and baseline dataset.
⁺ Indicates FSCI value-added to existing dat1.
Sources: Author’s calculations based on data sources listed in **Extended Data Table 1**.

Table 1.6 Income group median values

Domain	Indicator		Unit	Low income	Lower middle income	Upper middle income	High income
Diets, nutrition & health							
Food environments	Cost of a healthy diet		current PPP dollar/person/day	3.2	3.6	3.6	3.1
	Availability of fruits and vegetables	Fruits	grams/capita/day	117.5	177.2	240.7	208.0
		Vegetables	grams/capita/day	93.2	149.2	217.4	245.1
	Retail value of ultra-processed foods		current (nominal) US$/capita/year	21.7	50.8	178.1	485.0
	% Population using safely managed drinking water services (SDG 6.1.1)		% population	19.0	46.1	76.8	99.4
Food security	% Population experiencing moderate or severe food insecurity (SDG 2.1.2)		% population	30.4	8.2	6.1	2.5
	% Population who cannot afford a healthy diet		% population	72.4	41.7	25.3	7.5
	PoU: Prevalence of Undernourishment (SDG 2.1.1)		% population	89.0	66.8	18.0	0.8
Diet quality	MDD-W: minimum dietary diversity for women		% population, women 15-49	44.9	69.3	74.1	
	MDD (IYCF): minimum dietary diversity for infants and young children		% population, 6-23 months	22.8	30.6	53.0	69.8
	All-5: consumption of all 5 food groups		% adult population (≥15 y)	24.7	28.2	29.7	42.0
	Zero fruit or vegetable consumption	Adults	% adult population (≥15 y)	11.3	9.6	6.8	3.9
		Children 6-23 months	% population 6-23 months	44.1	36.3	21.2	5.5
	NCD-Protect		Score (points out of 9)	3.1	3.6	3.5	4.0
	NCD-Risk		Score (points out of 9)	1.2	1.6	2.5	3.2
	Sugar-sweetened soft drink consumption		% adult population (≥15 y)	15.9	21.2	31.6	42.4
Environment, natural resources & production							
Greenhouse gas emissions	Food systems greenhouse gas emissions		kt CO_2eq (AR5)	35,806.0	23,912.9	13,618.9	17,965.5
	Greenhouse gas emissions intensity, by product group	Cereals (excl. rice)	kg CO_2eq/kg product	0.1	0.2	0.3	0.2
		Beef	kg CO_2eq/kg product	78.6	57.6	32.5	17.5
		Cow's milk	kg CO_2eq/kg product	4.1	2.5	1.4	0.6
		Rice	kg CO_2eq/kg product	1.3	1.4	1.7	2.2
Production	Food product yield, by food group	Cereals	tonnes/ha	1.2	2.0	3.4	5.9
		Fruit	tonnes/ha	6.6	10.5	11.5	12.0
		Beef	kg/animal	125.0	165.2	199.2	269.5
		Cow's milk	kg/animal	459.3	1,108.2	2,022.3	7,022.9

Domain	Indicator	Unit	Low income	Lower middle income	Upper middle income	High income
	Vegetables	tonnes/ha	8.1	12.0	15.3	25.2
Land	Cropland change, 5-year average (%)	%	0.0	0.1	0.0	0.0
Water	Agriculture water withdrawal as % of total renewable water resources	% total renewable	1.6	5.1	1.3	0.8
Biosphere integrity	Functional integrity: % agricultural land with minimum level of natural habitat	% agricultural land	93.2	91.6	95.1	91.5
	Fishery health index progress score	index	6.8	19.2	22.8	31.0
Pollution	Total pesticides per unit of cropland	kg/ha	0.1	0.4	2.2	2.7
	Sustainable nitrogen management index	index	0.9	0.8	0.8	0.9
Livelihoods, poverty & equity						
Poverty and income	Share of agriculture in GDP	% GDP	23.8	14.9	7.1	1.8
Employment	Unemployment, rural	% working age population	3.7	4.6	7.9	5.0
	Underemployment rate, rural	% working age population	6.7	3.3	5.0	3.9
Social protection	Social protection coverage	% population	7.8	33.6	55.2	80.9
	Social protection adequacy	% welfare of beneficiary households	9.7	18.7	28.4	34.2
Rights	% Children 5-17 engaged in child labor	% children 5-17 (sex specific is % children 5-17 of each sex)	18.9	11.5	3.4	2.2
	Female share of landholdings	% landholdings by sex of operator	16.5	18.7	25.0	18.3
Governance						
Shared vision and strategic planning	Civil society participation index	index	0.7	0.7	0.7	0.9
	% Urban population living in cities signed onto the Milan Urban Food Policy Pact +	% urban population	0.0	0.0	0.0	0.0
	Degree of legal recognition of the Right to Food +	Categorical	2.0	2.0	2.0	2.0
	Presence of a national food system transformation pathway	Binary	1.0	1.0	1.0	0.0
Effective implementation	Government effectiveness index	index	-1.2	-0.5	-0.1	1.1
	International Health Regulations State Party Assessment report (IHR SPAR), Food safety capacity	Score	40.0	60.0	80.0	80.0
	Presence of health-related food taxes +	Binary	0.0	0.0	0.0	0.0
Accountability	V-Dem Accountability index	index	0.1	0.4	0.8	1.5
	Open Budget Index Score	index	27.0	42.0	55.0	64.0
	Guarantees for public access to information (SDG 16.10.2)	Binary	1.0	1.0	1.0	1.0

Domain	Indicator		Unit	Low income	Lower middle income	Upper middle income	High income
Resilience							
Exposure to shocks	Ratio of total damages of all disasters to GDP		Ratio	0.0	0.0	0.0	0.0
Resilience capacities	Dietary sourcing flexibility index		index	0.6	0.7	0.7	0.8
	Mobile cellular subscriptions (per 100 people)		Number per 100 people	57.8	99.1	110.6	125.2
	Social capital index		index	0.4	0.4	0.4	0.6
Agro- and Food Diversity	Proportion of agricultural land with minimum level of species diversity (crop and pasture) +		% agricultural land	43.8	22.1	12.6	0.0
	Number of (a) plant and (b) animal genetic resources for food and agriculture secured in either medium- or long-term conservation facilities (SDG 2.5.1)	Plants	thousands	4.7	6.5	6.0	20.8
		Animals	number	0.0	0.0	0.0	1.0
Resilience responses/ strategies	Coping strategies index		% population	40.9	38.3	38.6	
Long-term outcomes	Food price volatility +		unitless	0.8	0.7	0.7	0.8
	Food supply variability		kcal/capita/day	28.5	26.5	26.0	26.0

+ Indicates FSCI value-added to existing data

Sources: Author's calculations based on data sources listed in **Extended Data Table 1**.

Supplementary Information, Appendix A: Supplementary Figures

Figure 1.1: FSCI Theory of Transformation

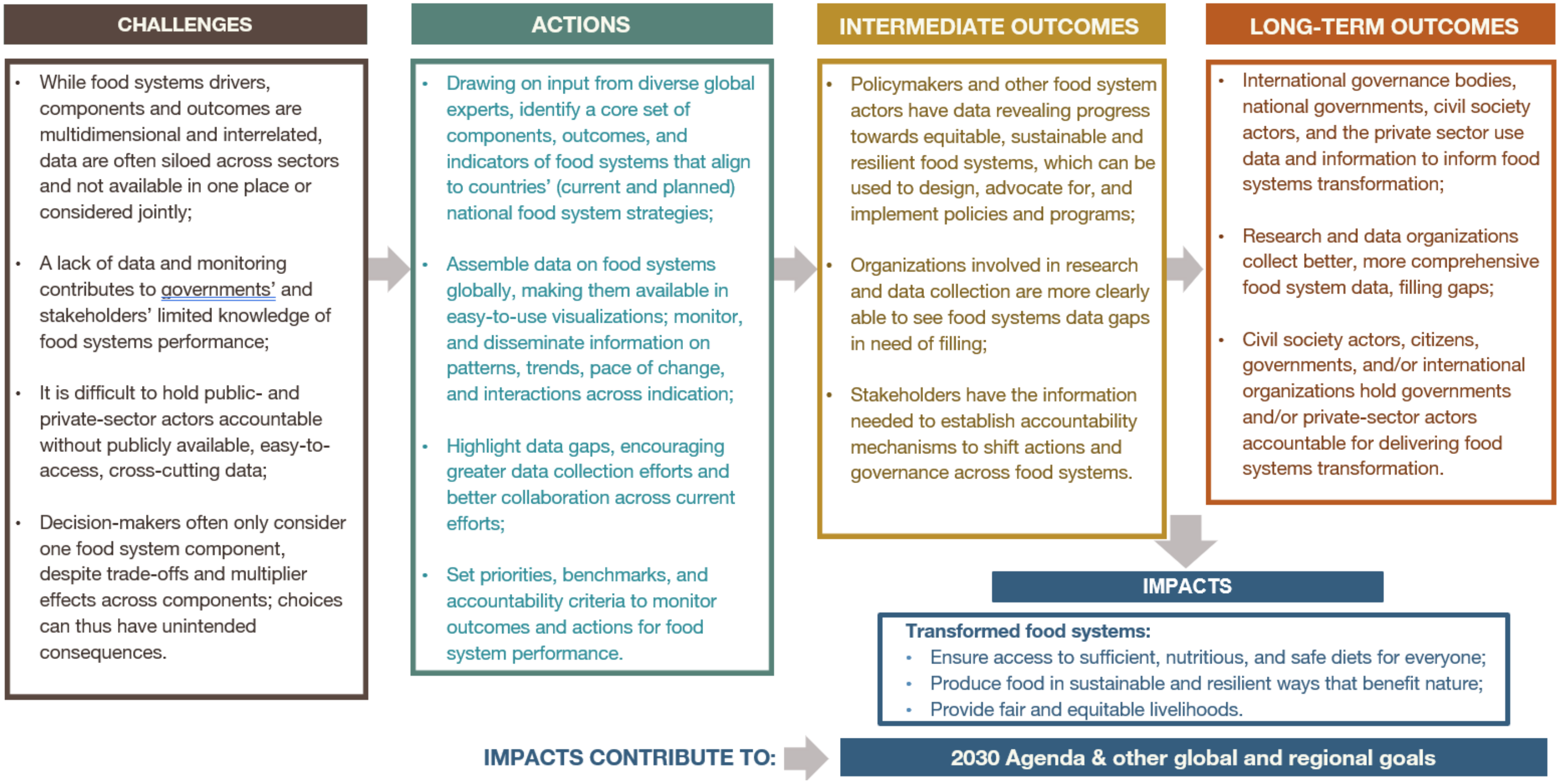

Figure 1.2 Country alignment to modified M49 regions

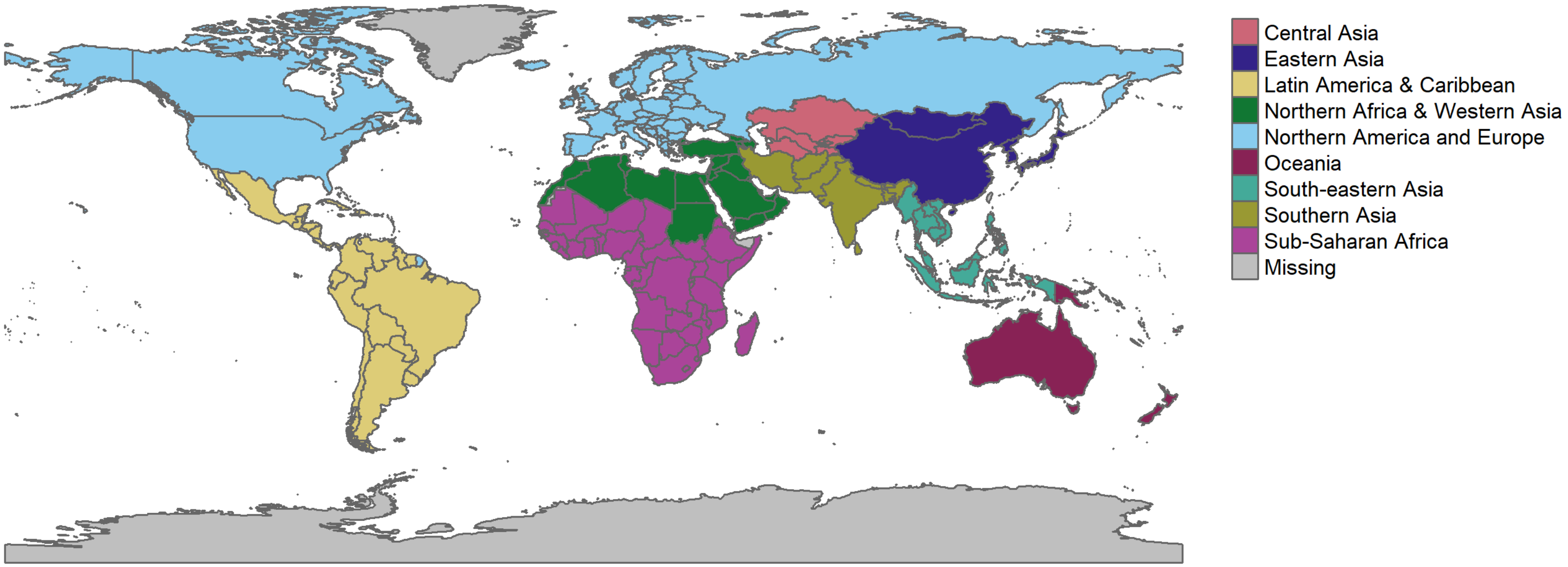

Basemap obtained using the R package ‘rnaturalearth’ which accesses Natural Earth v4.1.0 (March 2018) maps which are wholly in the public domain.

Figure 1.3 Indicator coverage by country and extent of time series

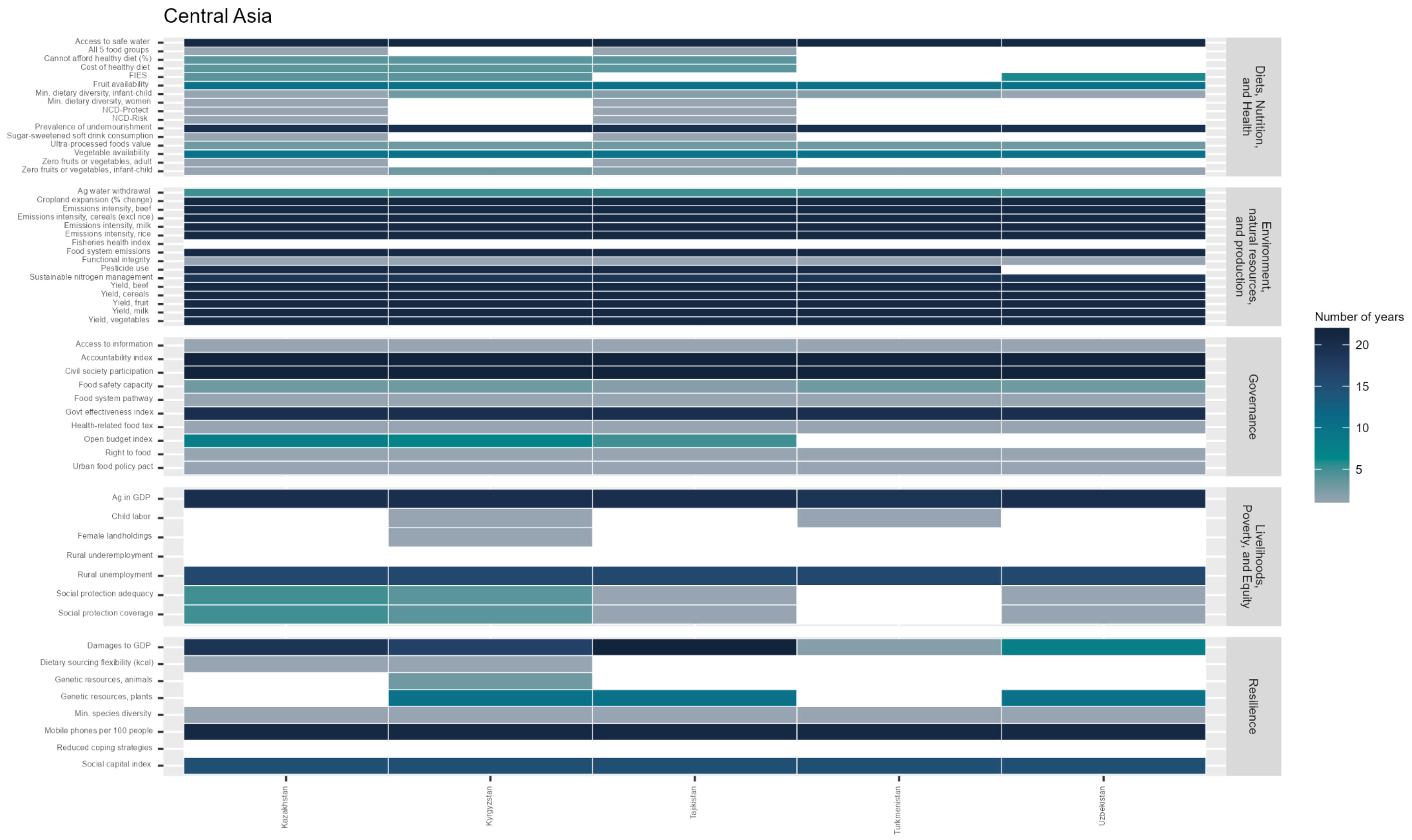

Figure 1.4 Indicator coverage by country and extent of time series

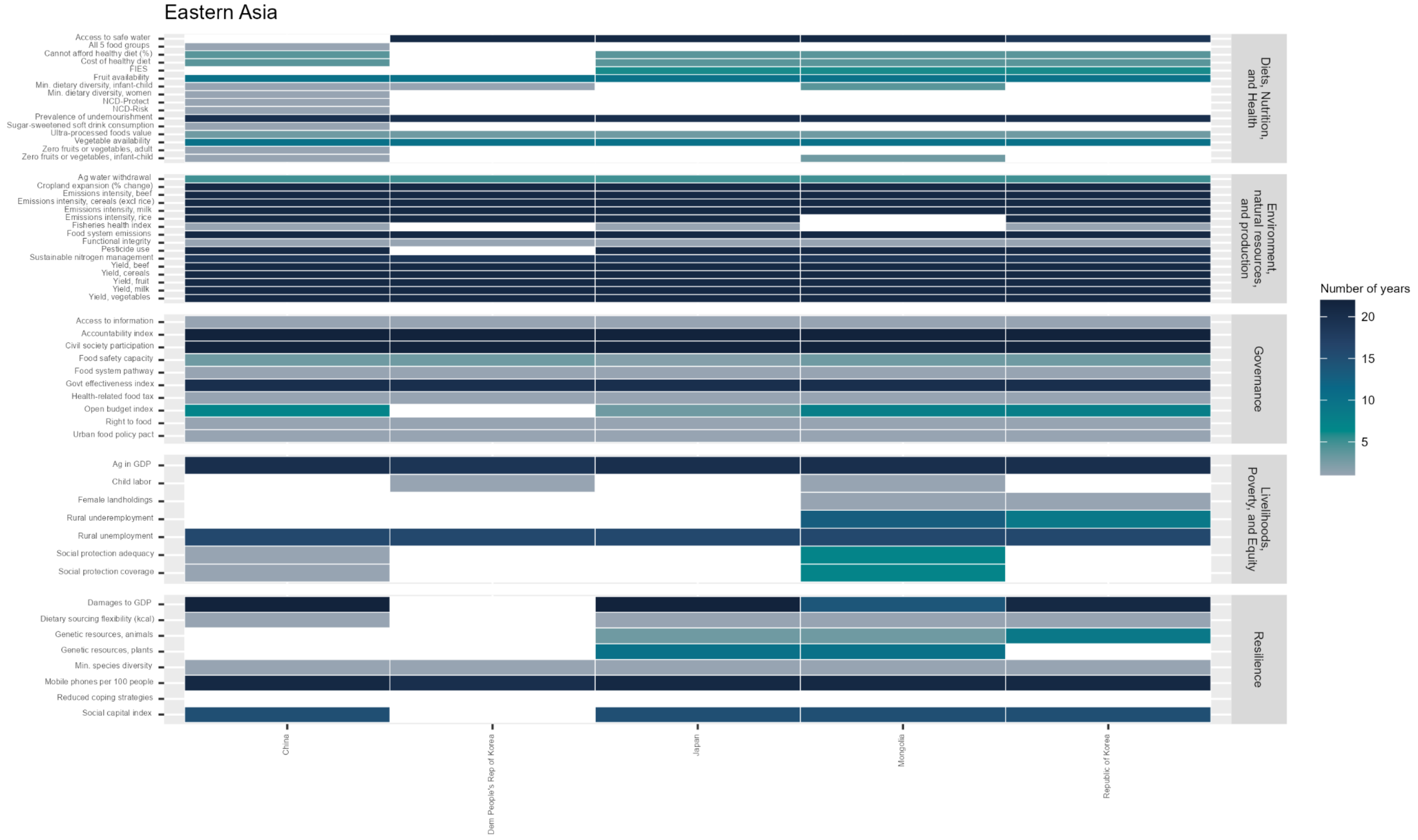

Figure 1.5 Indicator coverage by country and extent of time series

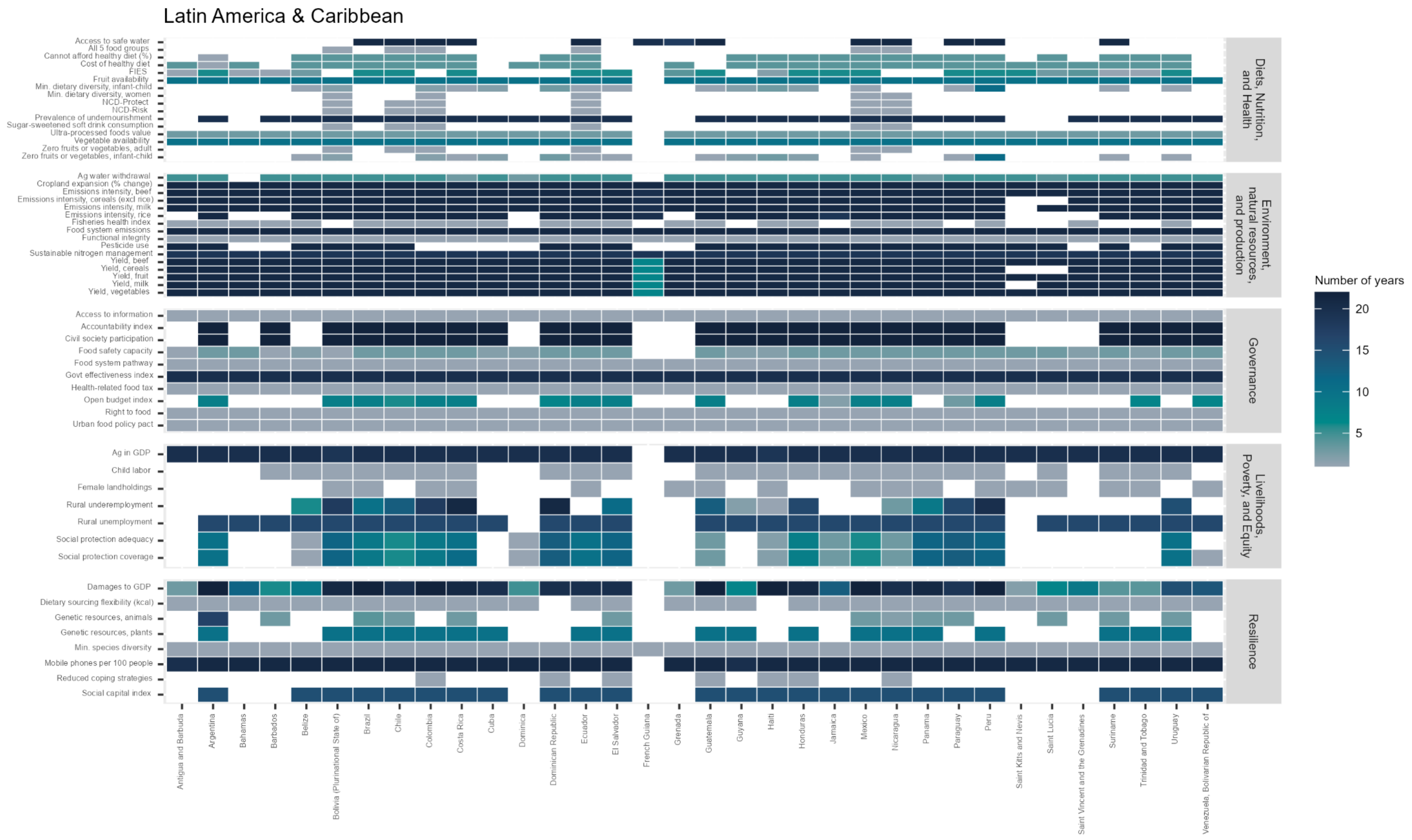

Figure 1.6 Indicator coverage by country and extent of time series

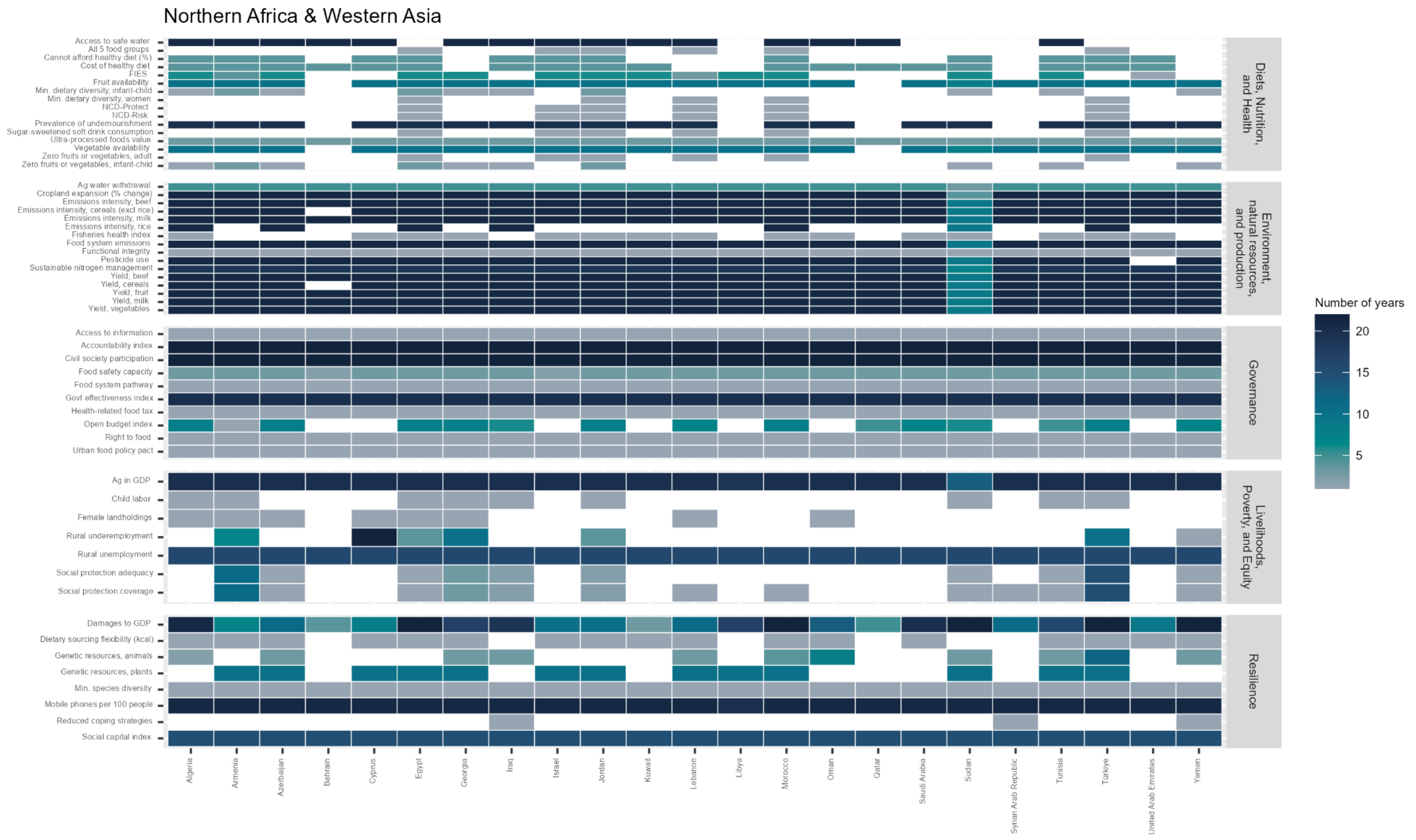

Figure 1.7 Indicator coverage by country and extent of time series

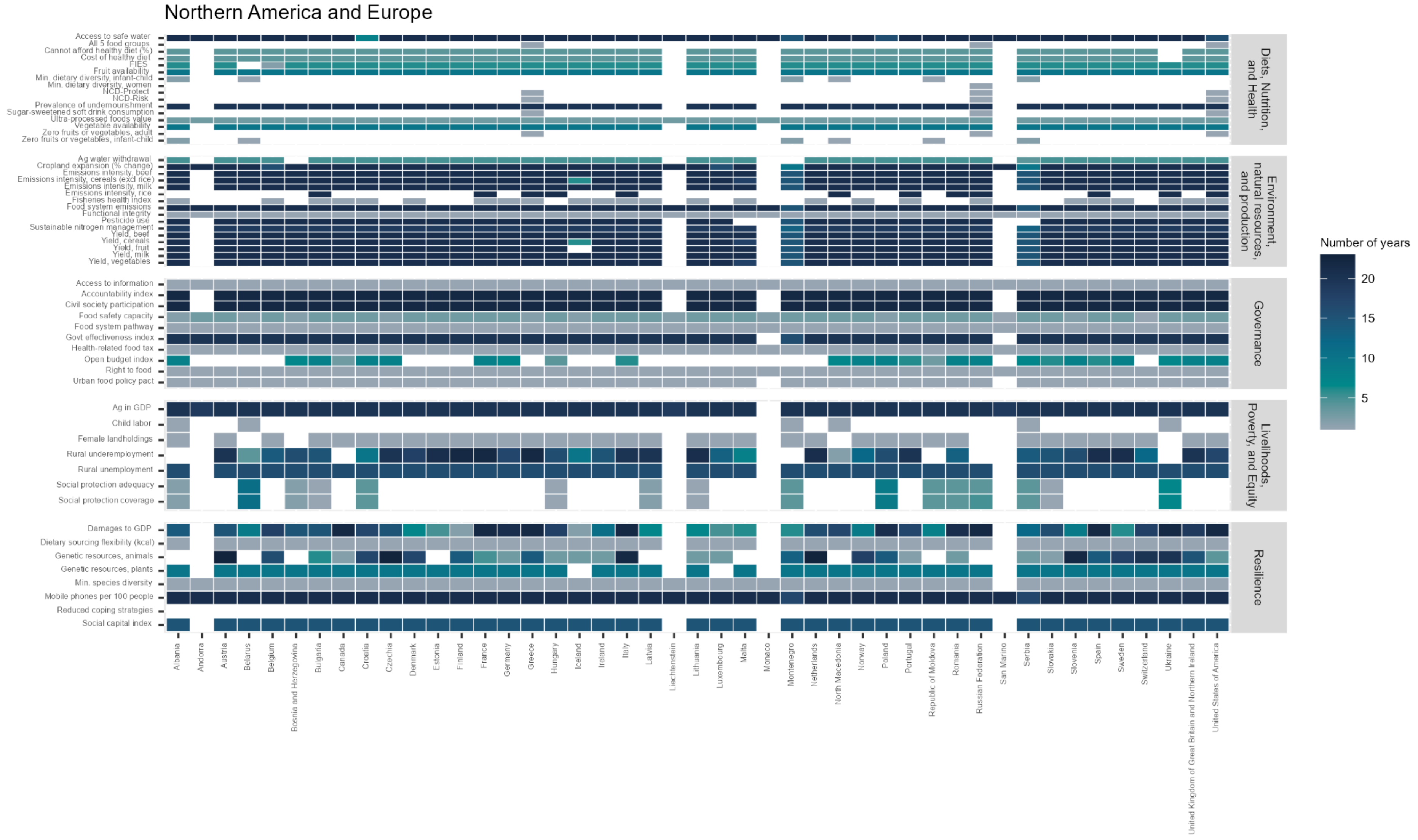

Figure 1.8 Indicator coverage by country and extent of time series

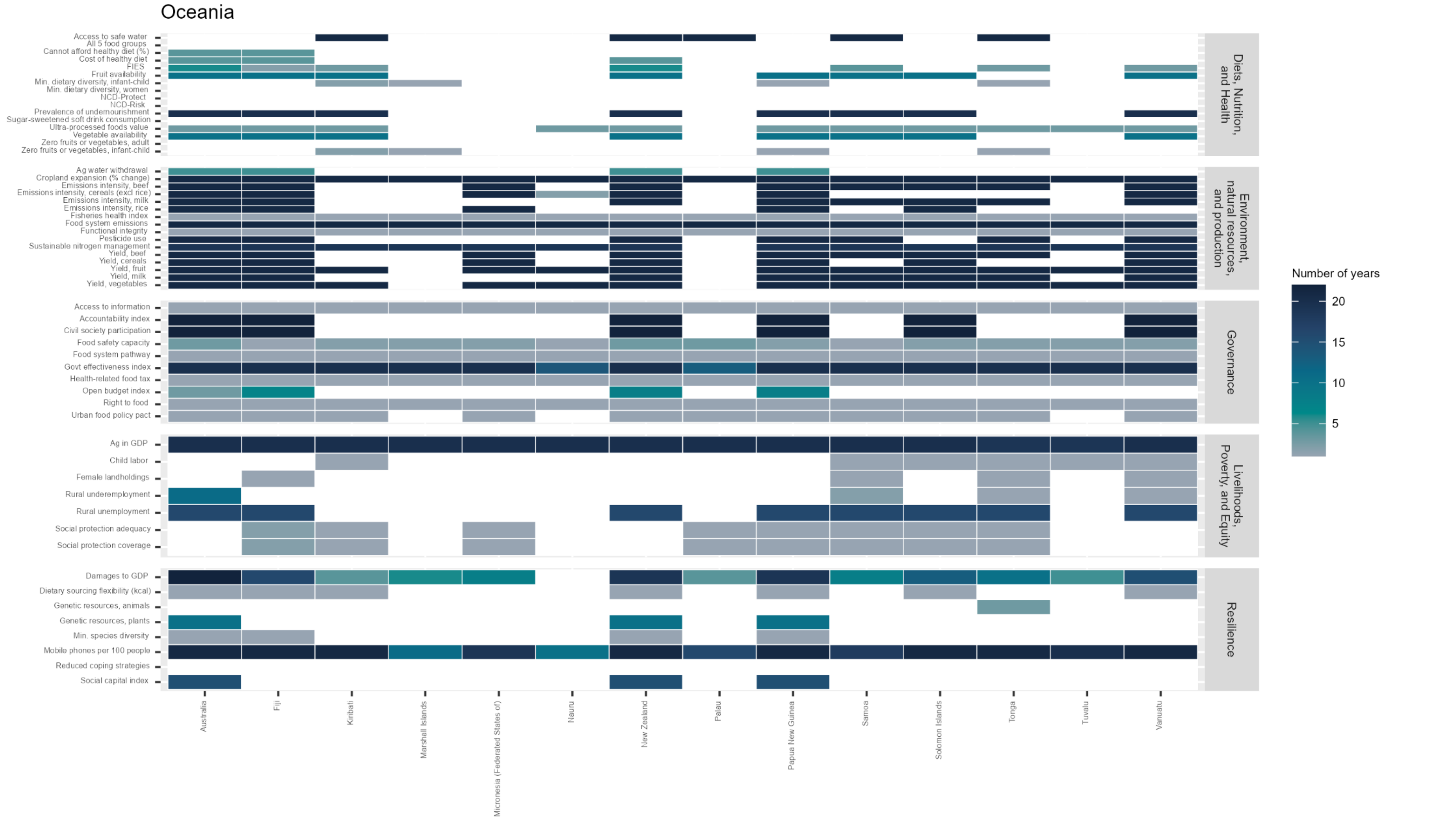

Figure 1.9 Indicator coverage by country and extent of time series

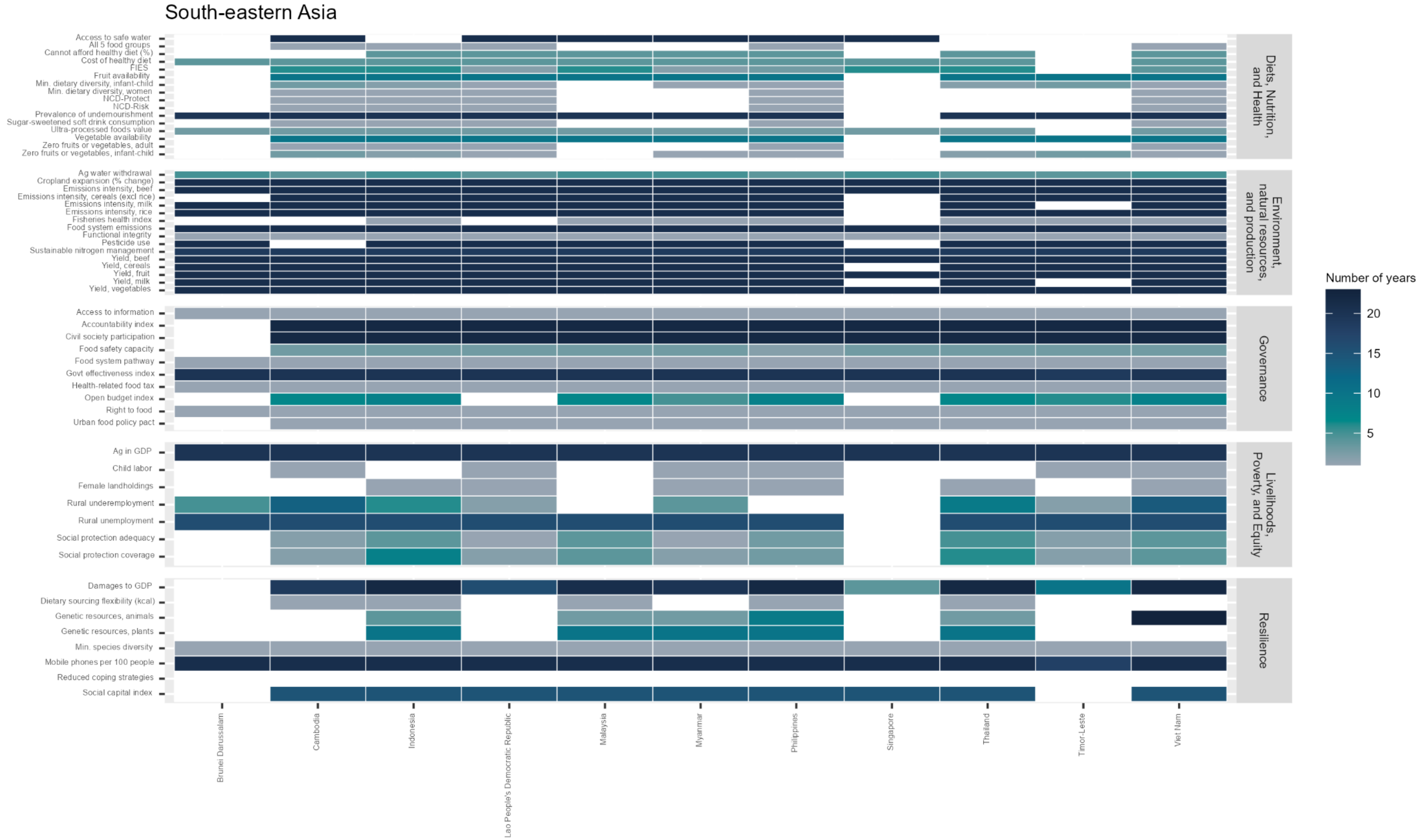

Figure 1.10 Indicator coverage by country and extent of time series

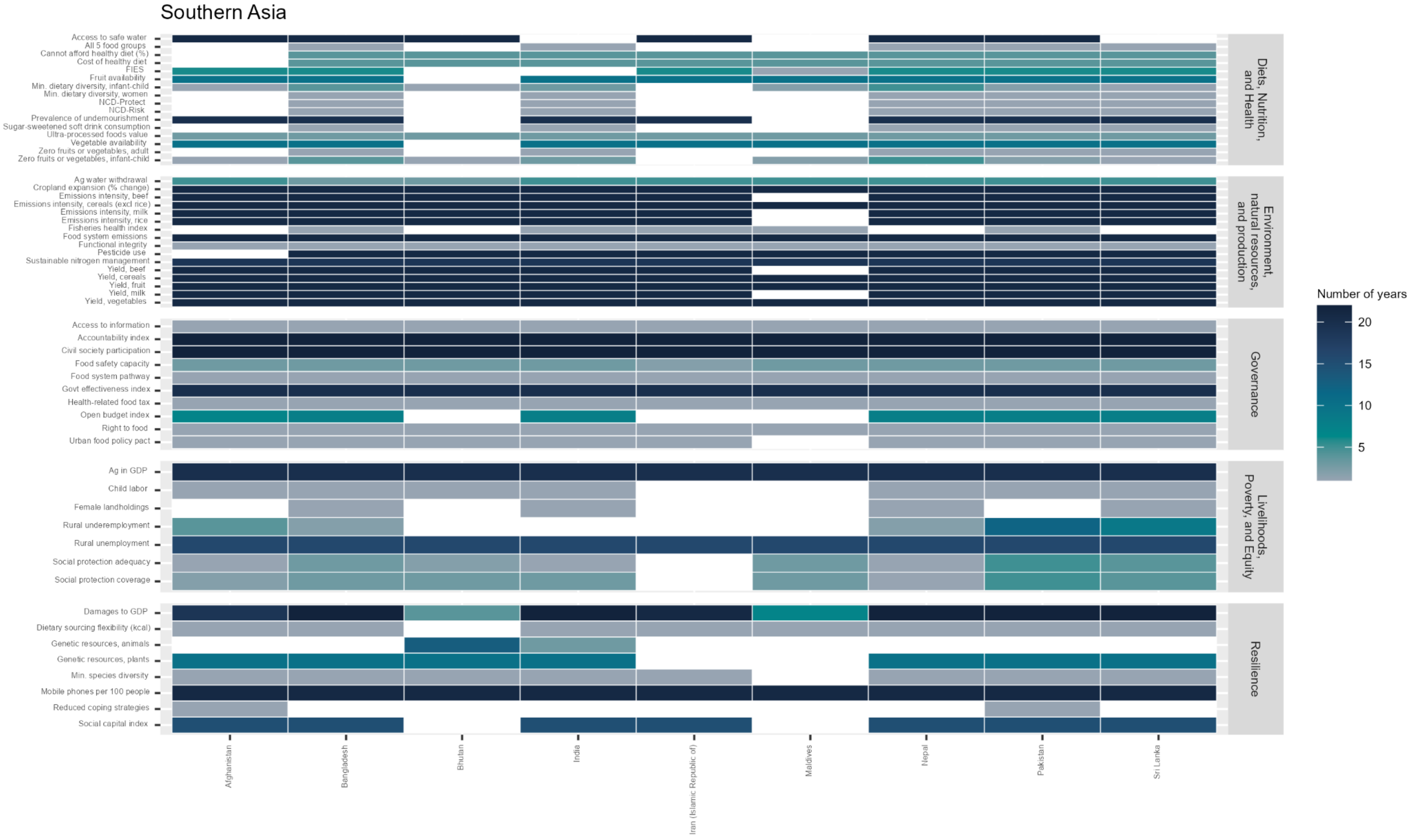

Figure 1.11 Indicator coverage by country and extent of time series

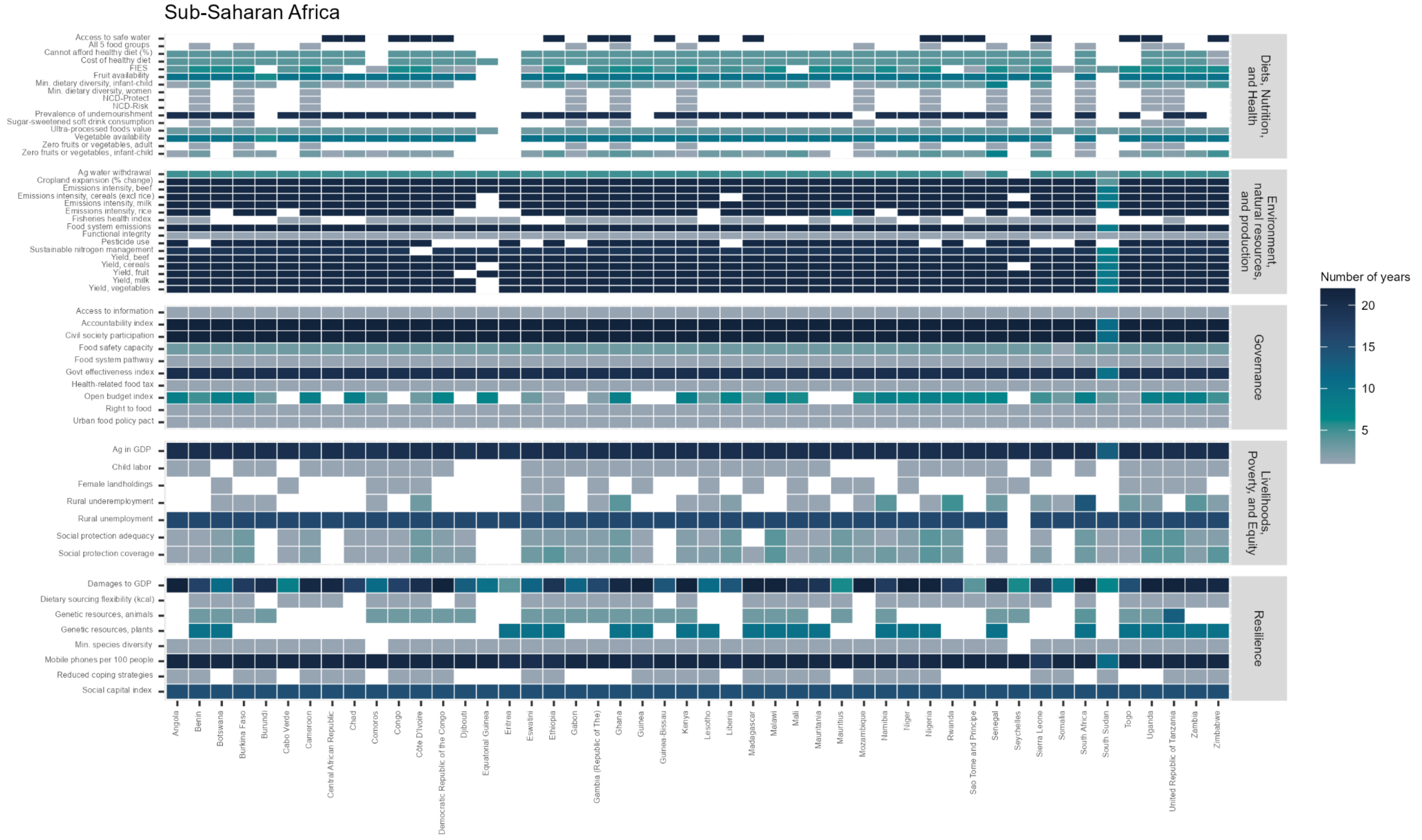

Figures 1.12 Country average ranking by theme and region: Southern Asia

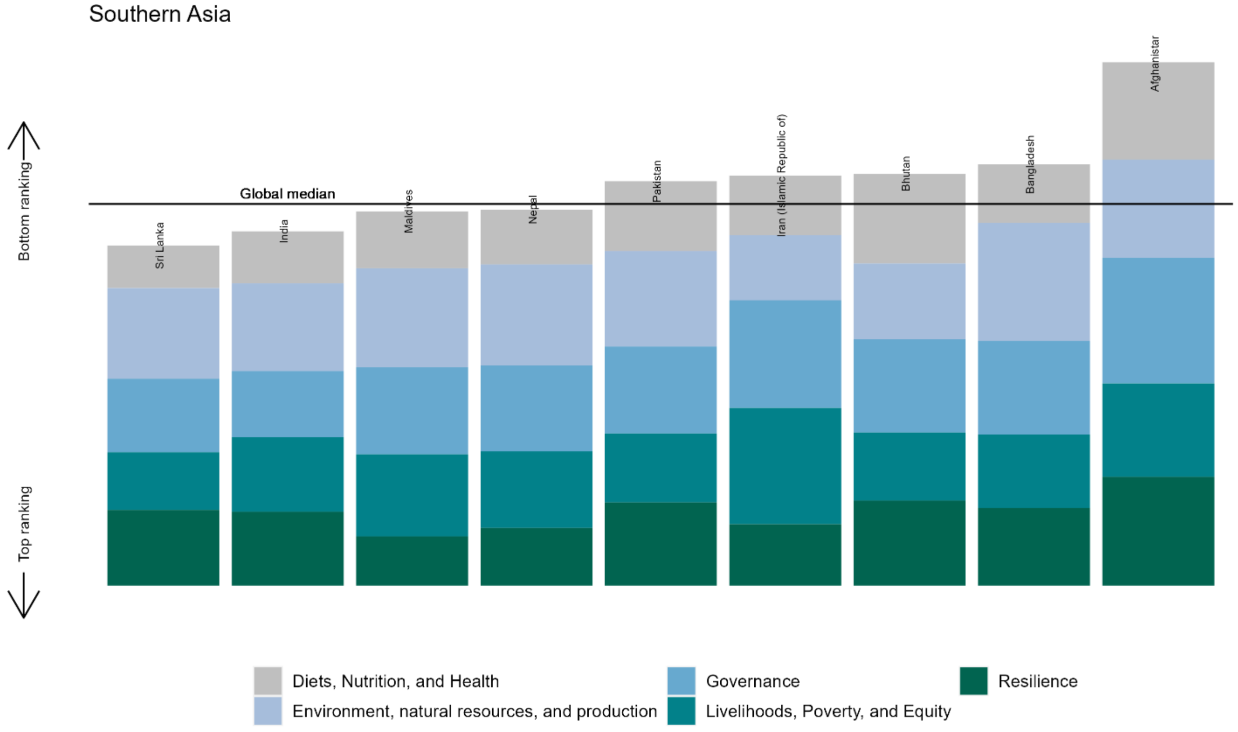

Figures 1.13 Country average ranking by theme and region: South-Eastern Asia

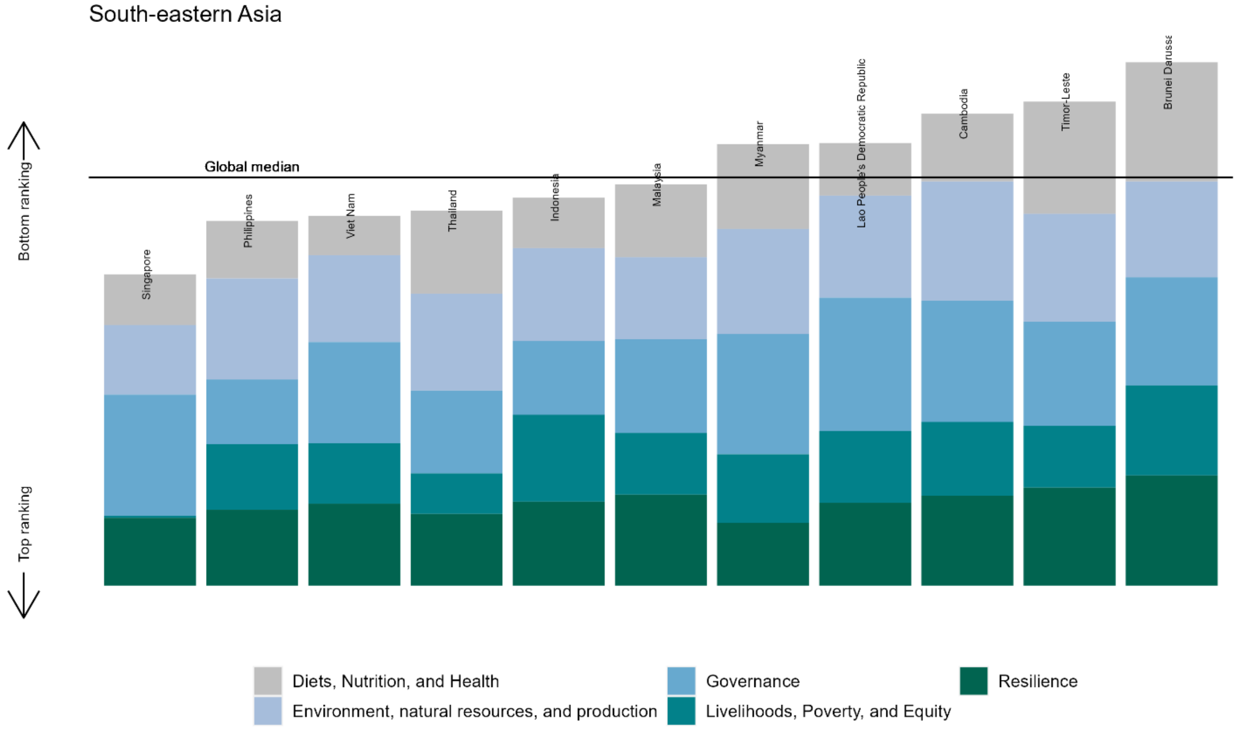

Figures 1.14 Country average ranking by theme and region: Oceania

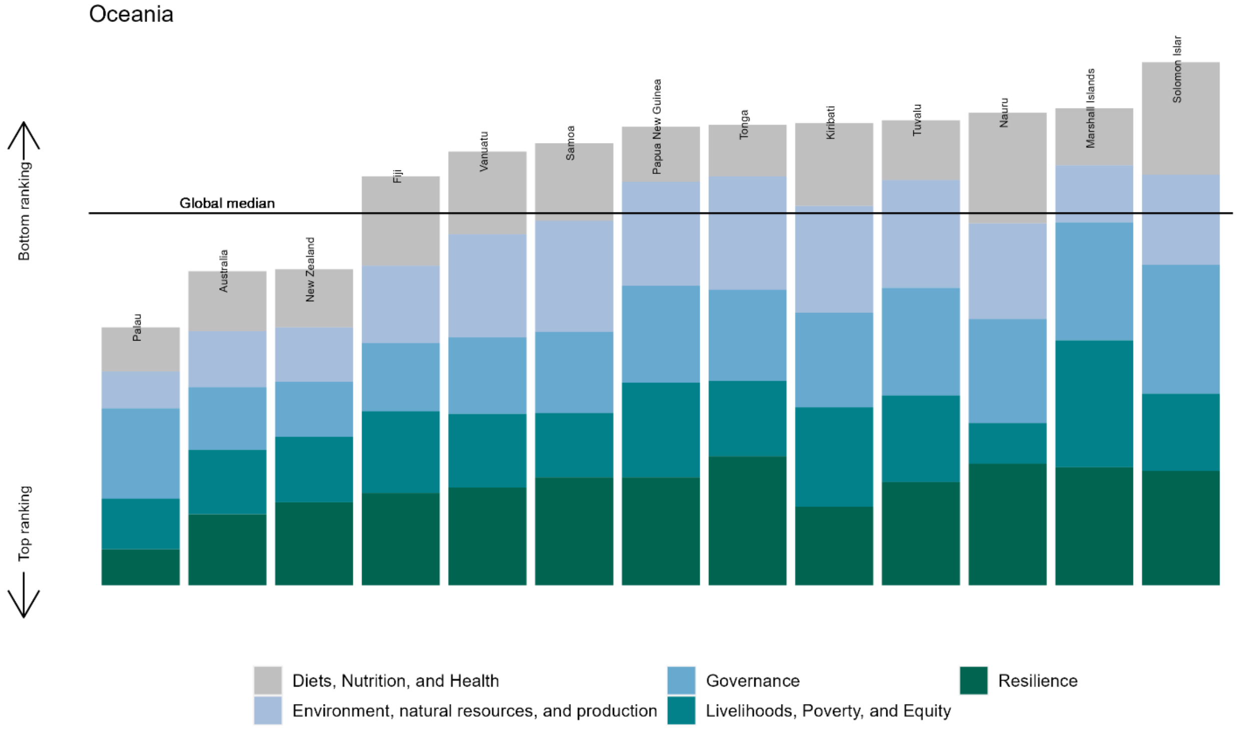

Figures 1.15 Country average ranking by theme and region: Northern America & Europe

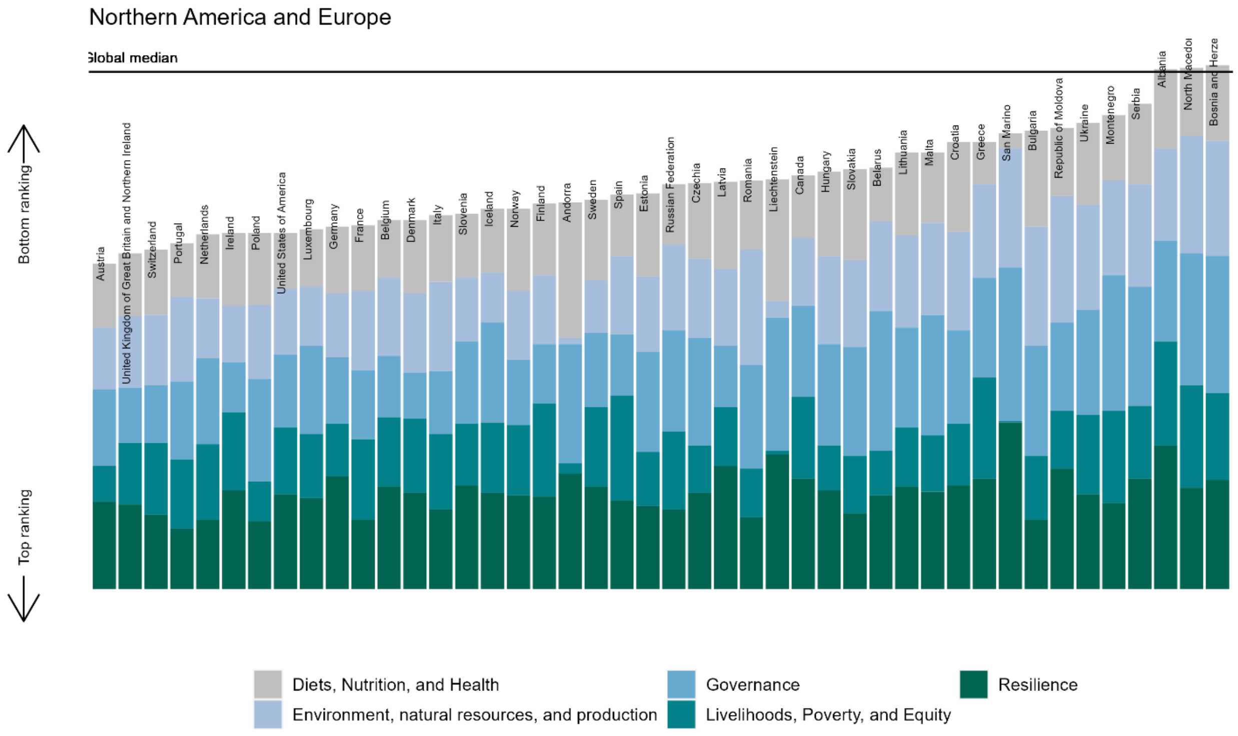

Figures 1.16 Country average ranking by theme and region: Northern Africa & Western Asia

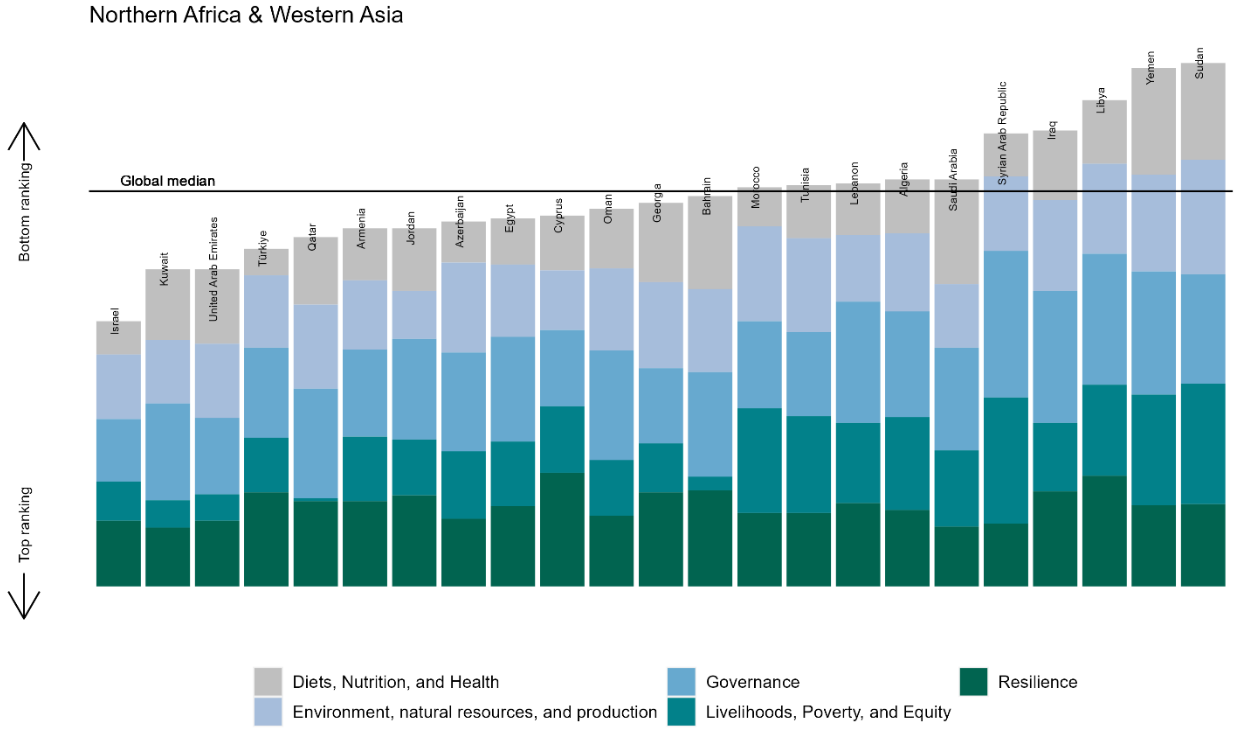

Figures 1.17 Country average ranking by theme and region: Latin America & Caribbean

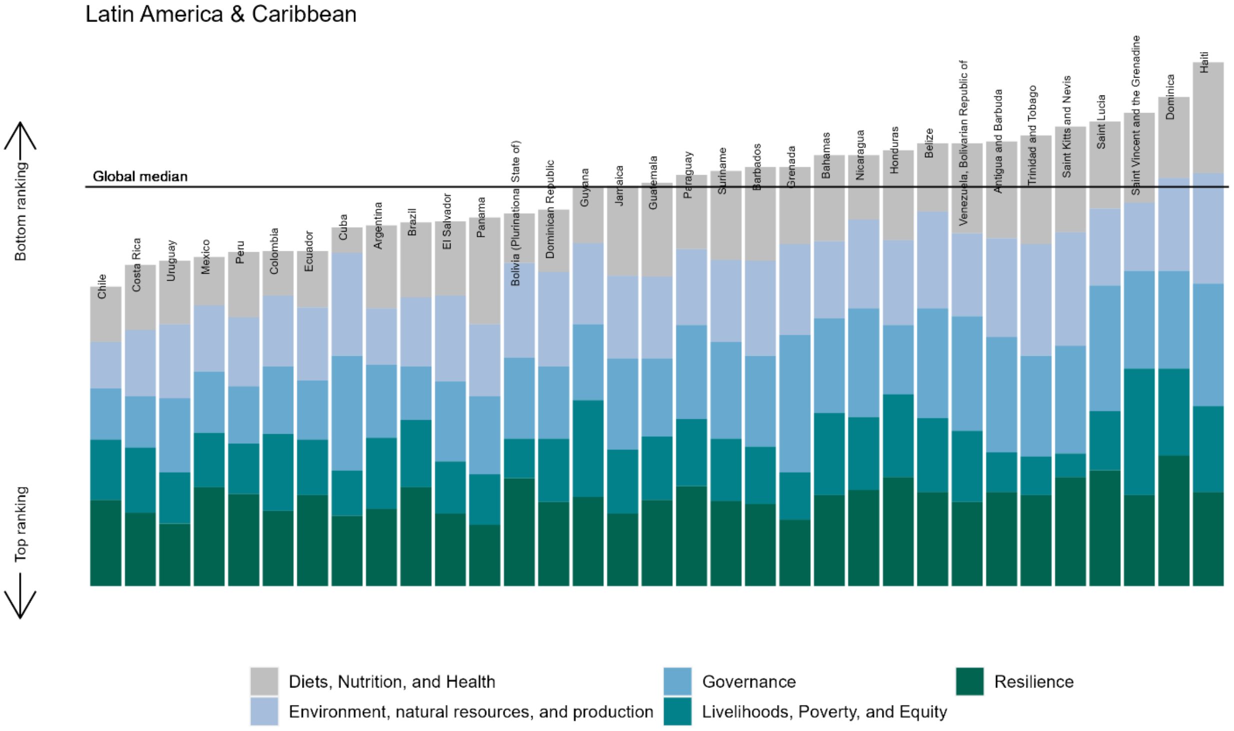

Figures 1.18 Country average ranking by theme and region: Eastern Asia

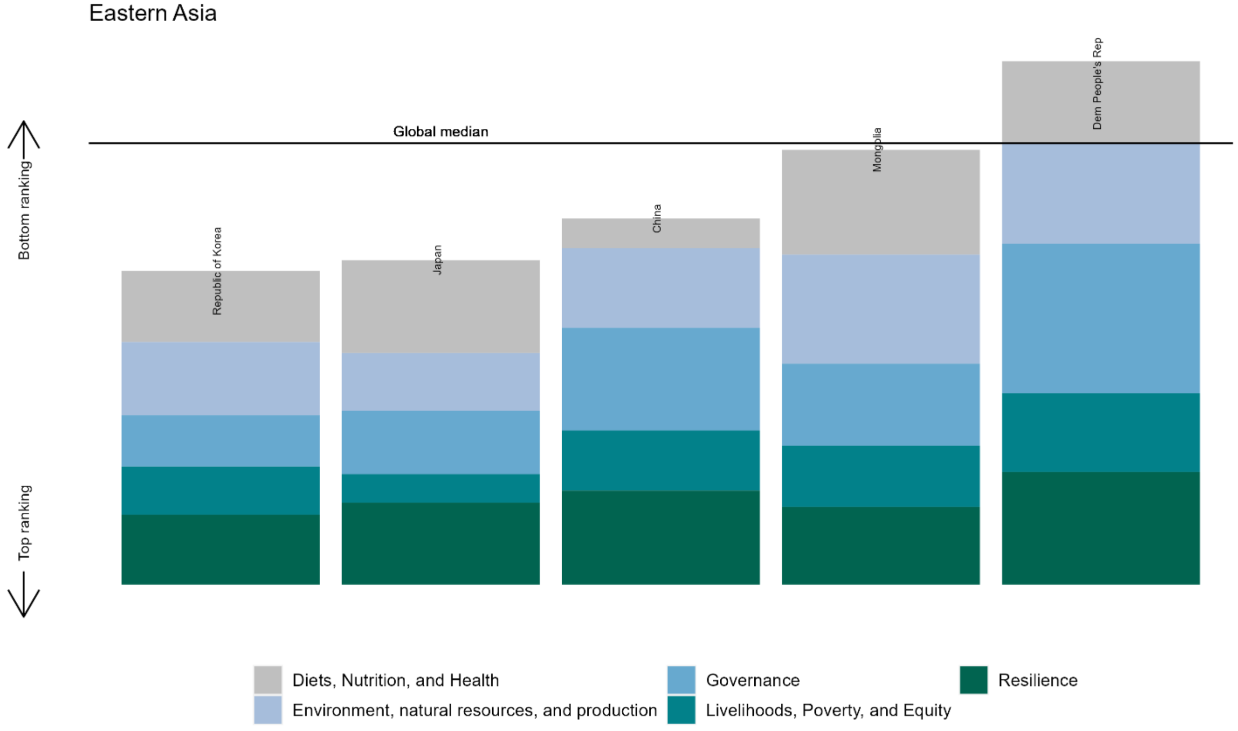

Figures 1.19 Country average ranking by theme and region: Central Asia

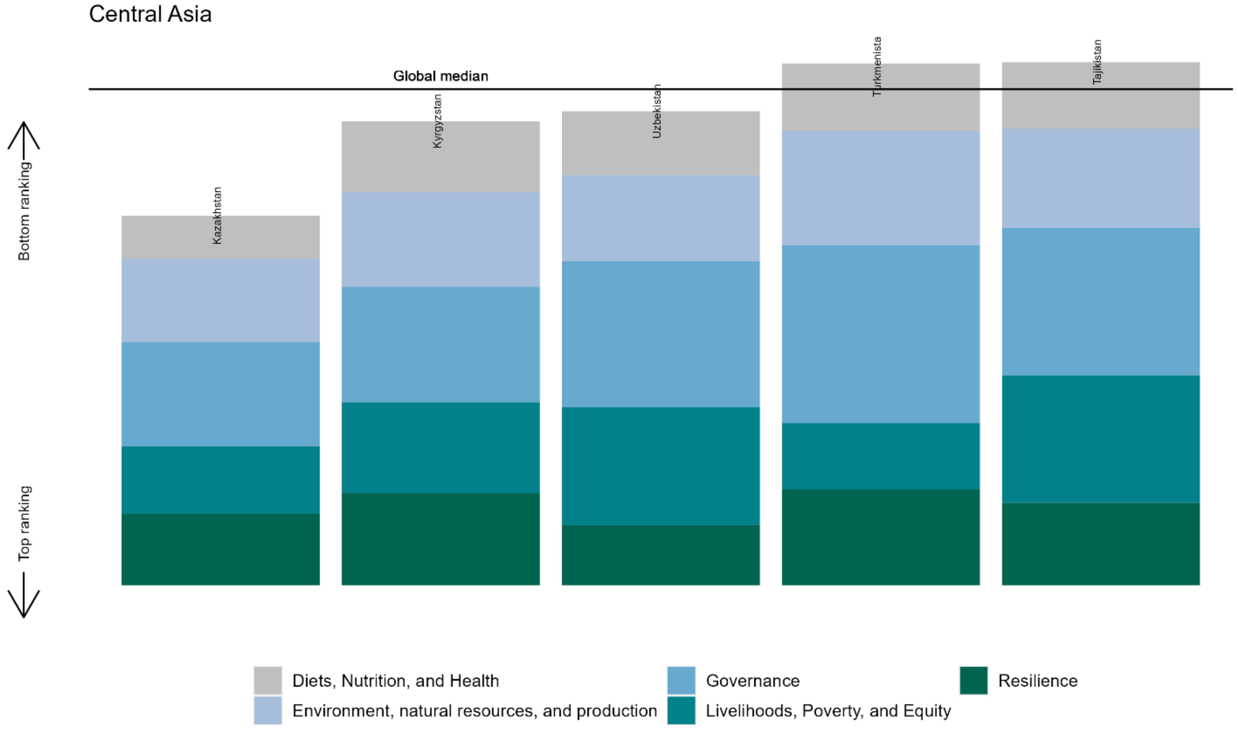

Figures 1.20 Country average ranking by theme and region: Sub-Saharan Africa

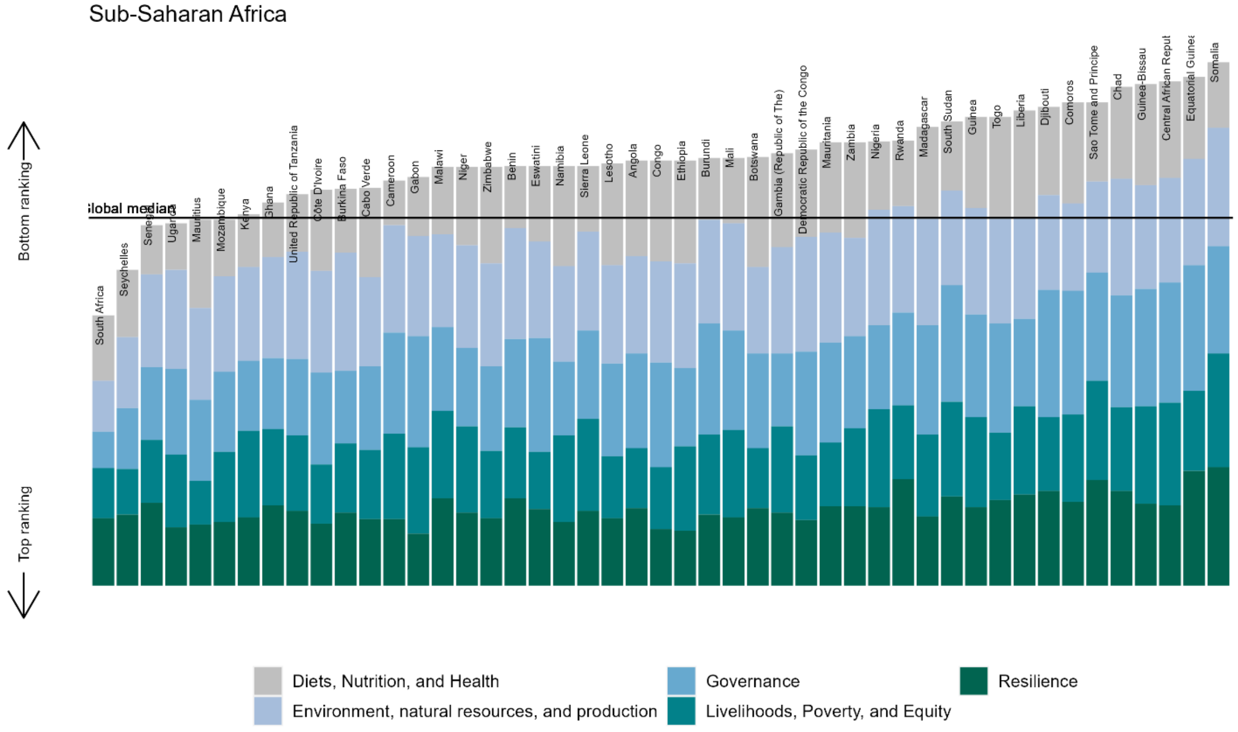

Figure 1.21 Relation to GDP per capita: Diets, nutrition, and health

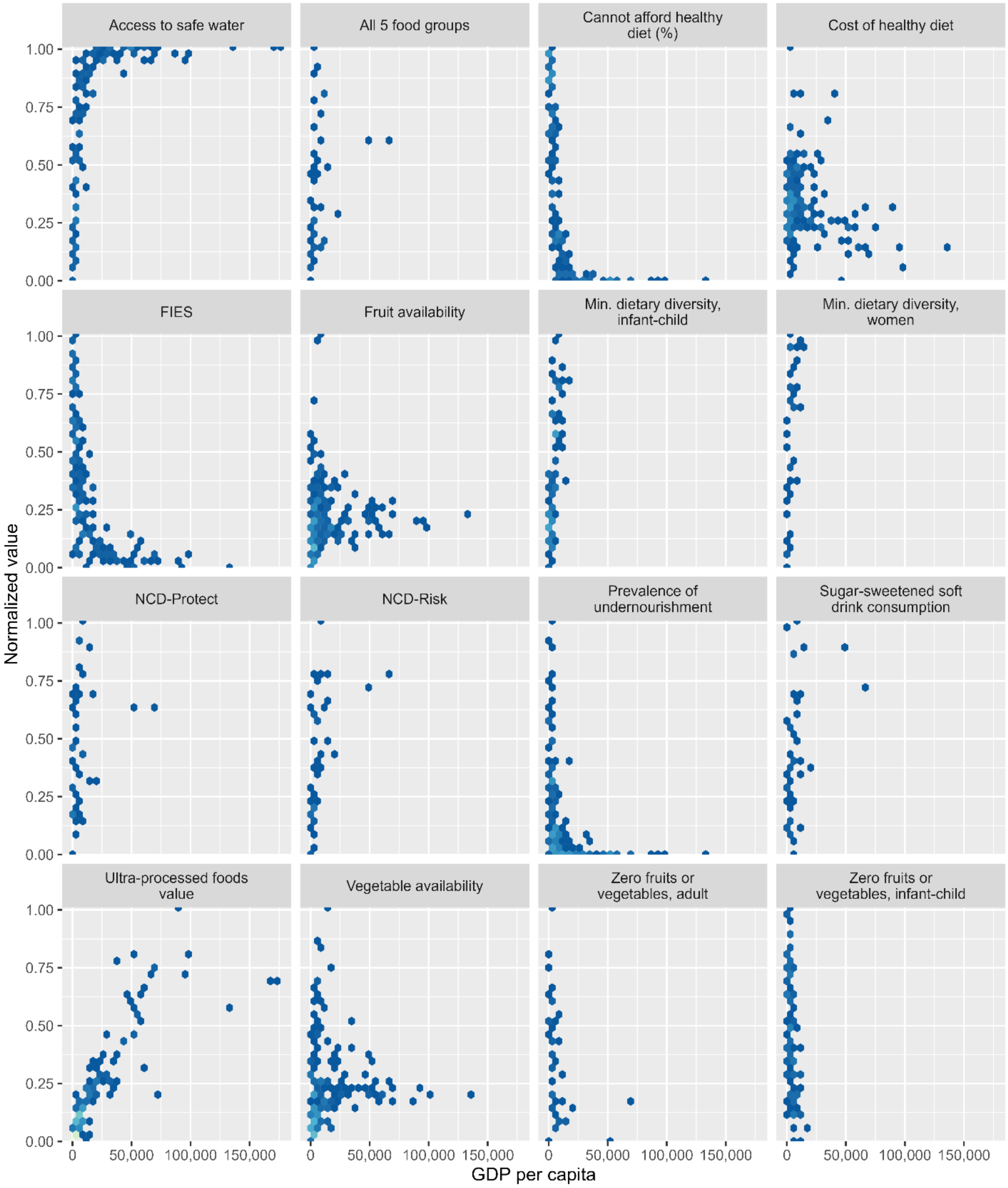

Sources: Author's calculations based on data sources listed in **Extended Data Table 1** in the latest year per country-indicator.
Notes: GDP per capita is in nominal terms and data come from 2021 for most countries, see **SM-E** for the specific year of data for GDP and each variable. Normalized values are calculated using max-min normalization relative to the global weighted mean.

Figure 1.22 Relation to GDP per capita: Environment, natural resources, and production

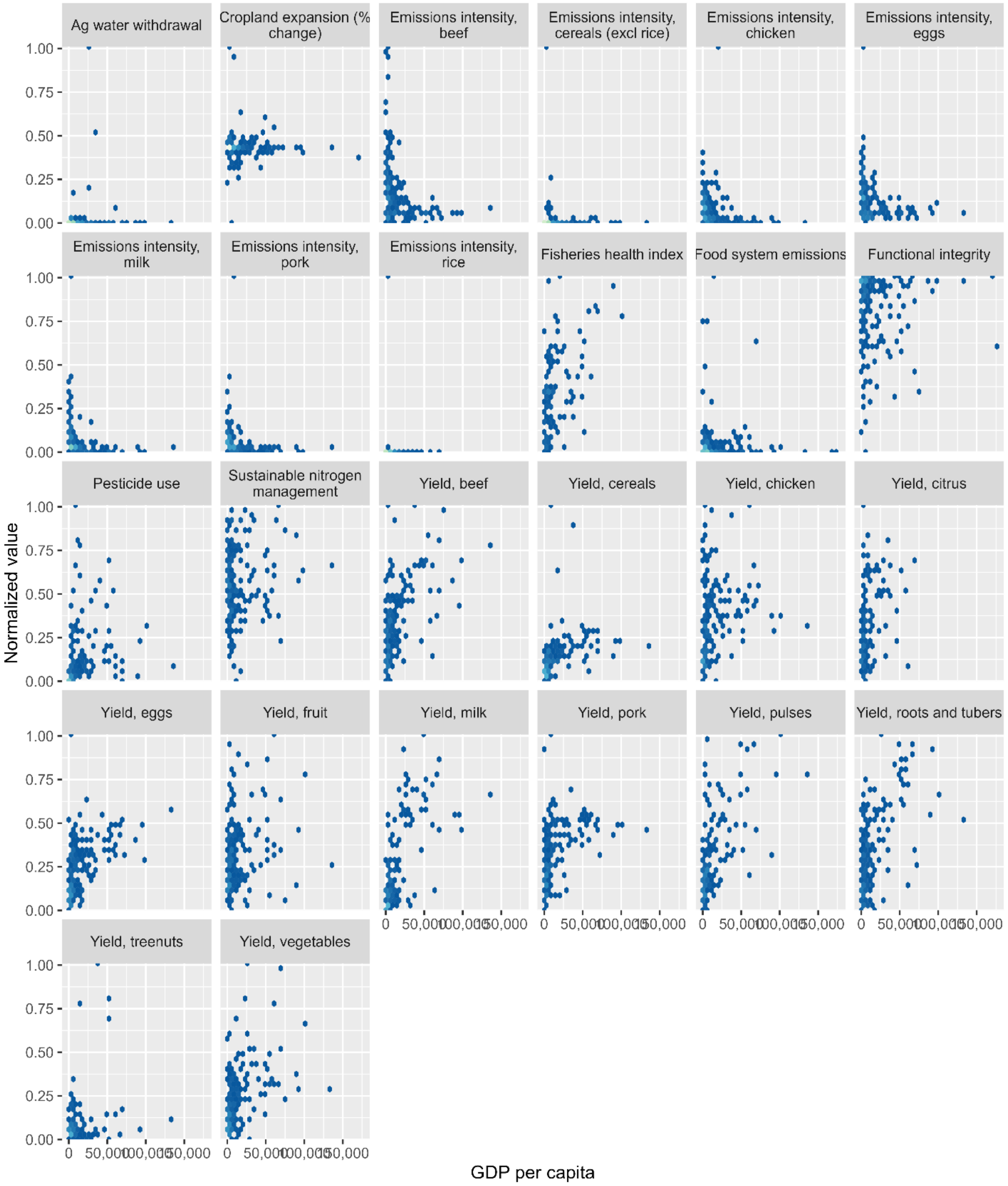

Sources: Author's calculations based on data sources listed in **Extended Data Table 1** in the latest year per country-indicator.
Notes: GDP per capita is in nominal terms and data come from 2021 for most countries, see **SM-E** for the specific year of data for GDP and each variable. Normalized values are calculated using max-min normalization relative to the global weighted mean.

Figure 1.23 Relation to GDP per capita: Livelihoods, poverty, and equity

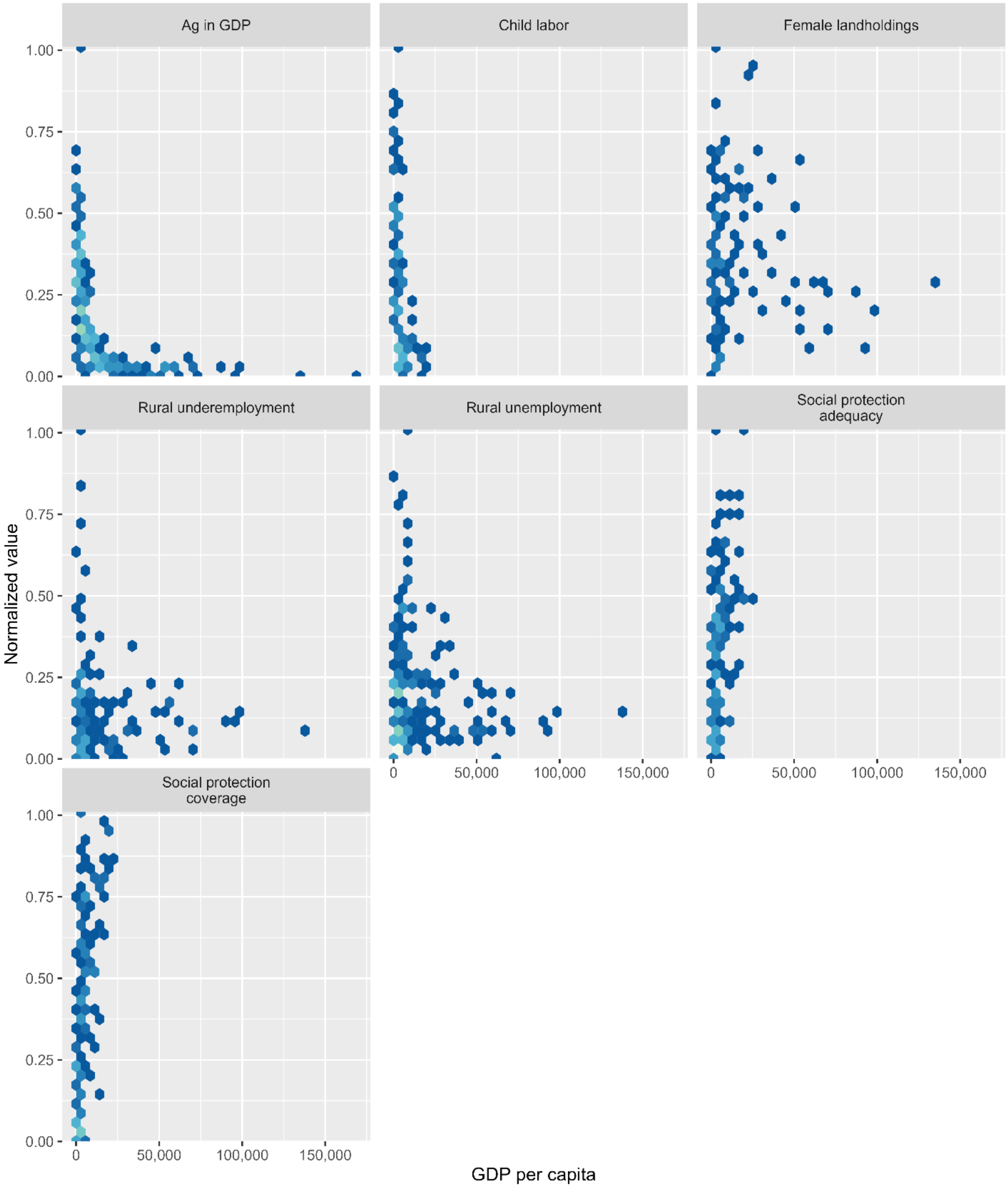

Sources: Author's calculations based on data sources listed in **Extended Data Table 1** in the latest year per country-indicator.
Notes: GDP per capita is in nominal terms and data come from 2021 for most countries, see **SM-E** for the specific year of data for GDP and each variable. Normalized values are calculated using max-min normalization relative to the global weighted mean.

Figure 1.24 Relation to GDP per capita: Governance

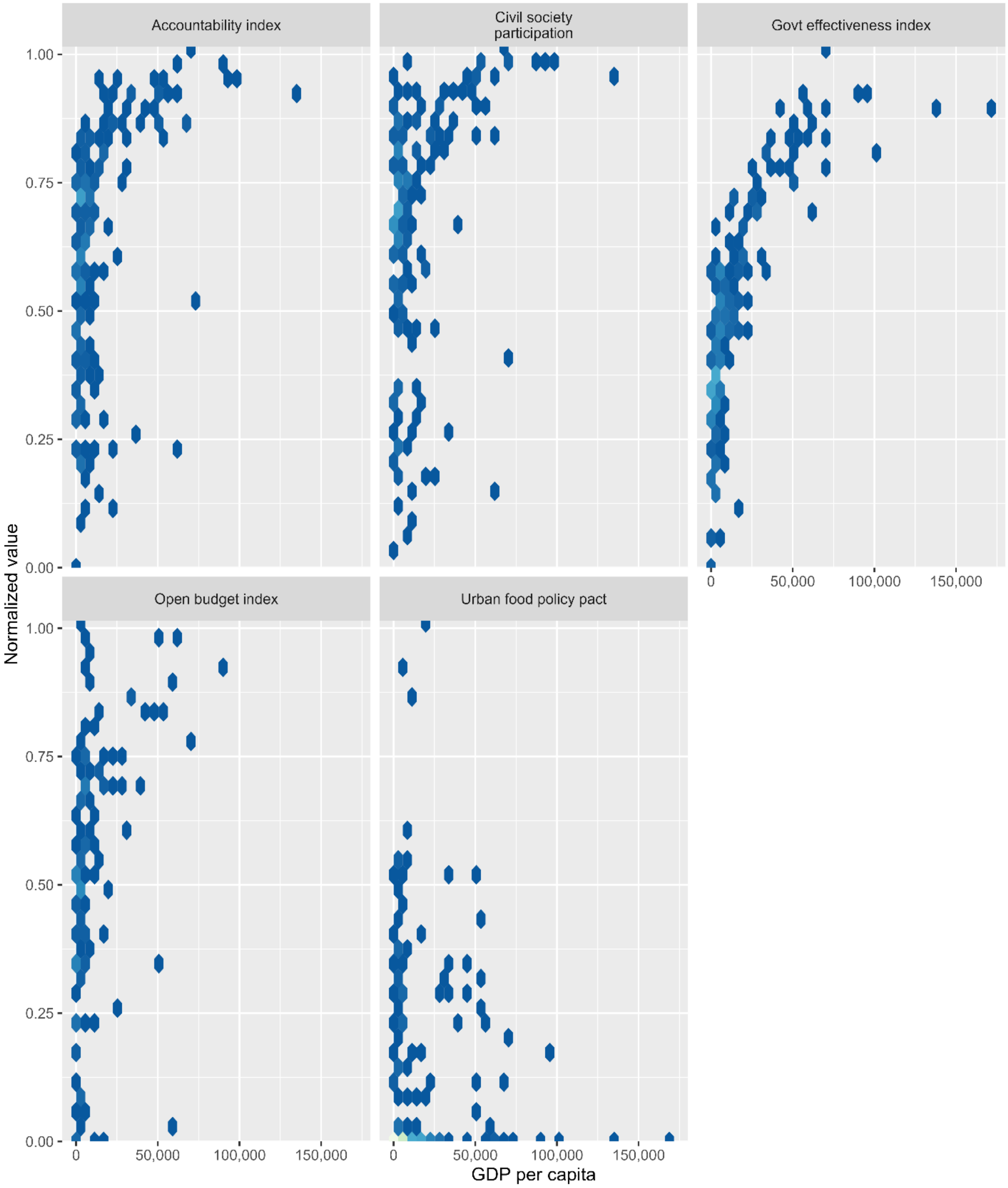

Sources: Author's calculations based on data sources listed in **Extended Data Table 1** in the latest year per country-indicator.
Notes: Only continuous indicators shown. GDP per capita is in nominal terms and data come from 2021 for most countries, see **SM-E** for the specific year of data for GDP and each variable. Normalized values are calculated using max-min normalization relative to the global weighted mean.

Figure 1.25 Relation to GDP per capita: Resilience

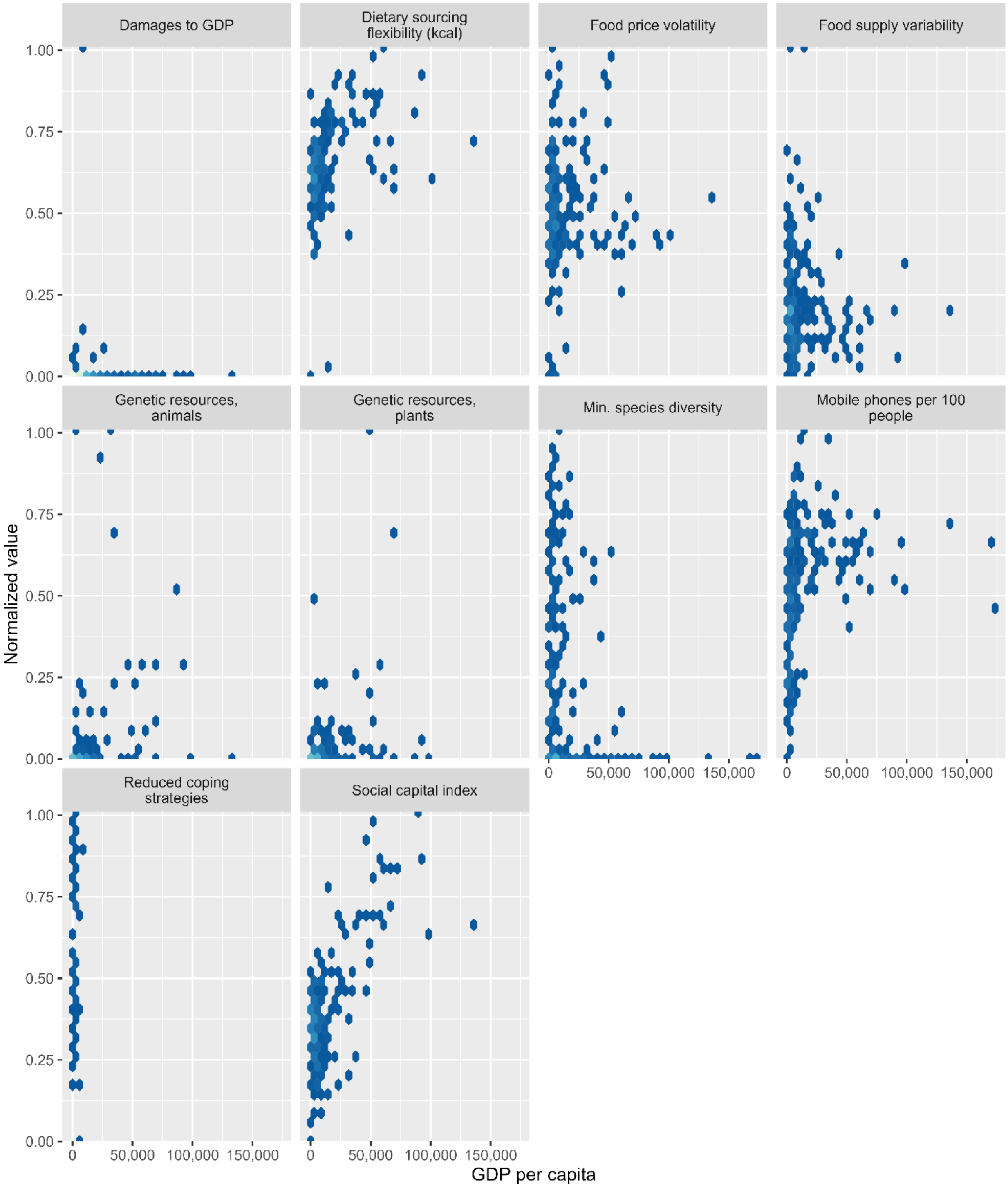

Sources: Author's calculations based on data sources listed in **Extended Data Table 1** in the latest year per country-indicator.
Notes: GDP per capita is in nominal terms and data come from 2021 for most countries, see **SM-E** for the specific year of data for GDP and each variable. Normalized values are calculated using max-min normalization relative to the global weighted mean.

Figure 1.26 Spearman rank correlation coefficient matrix, latest data point per country-indicator

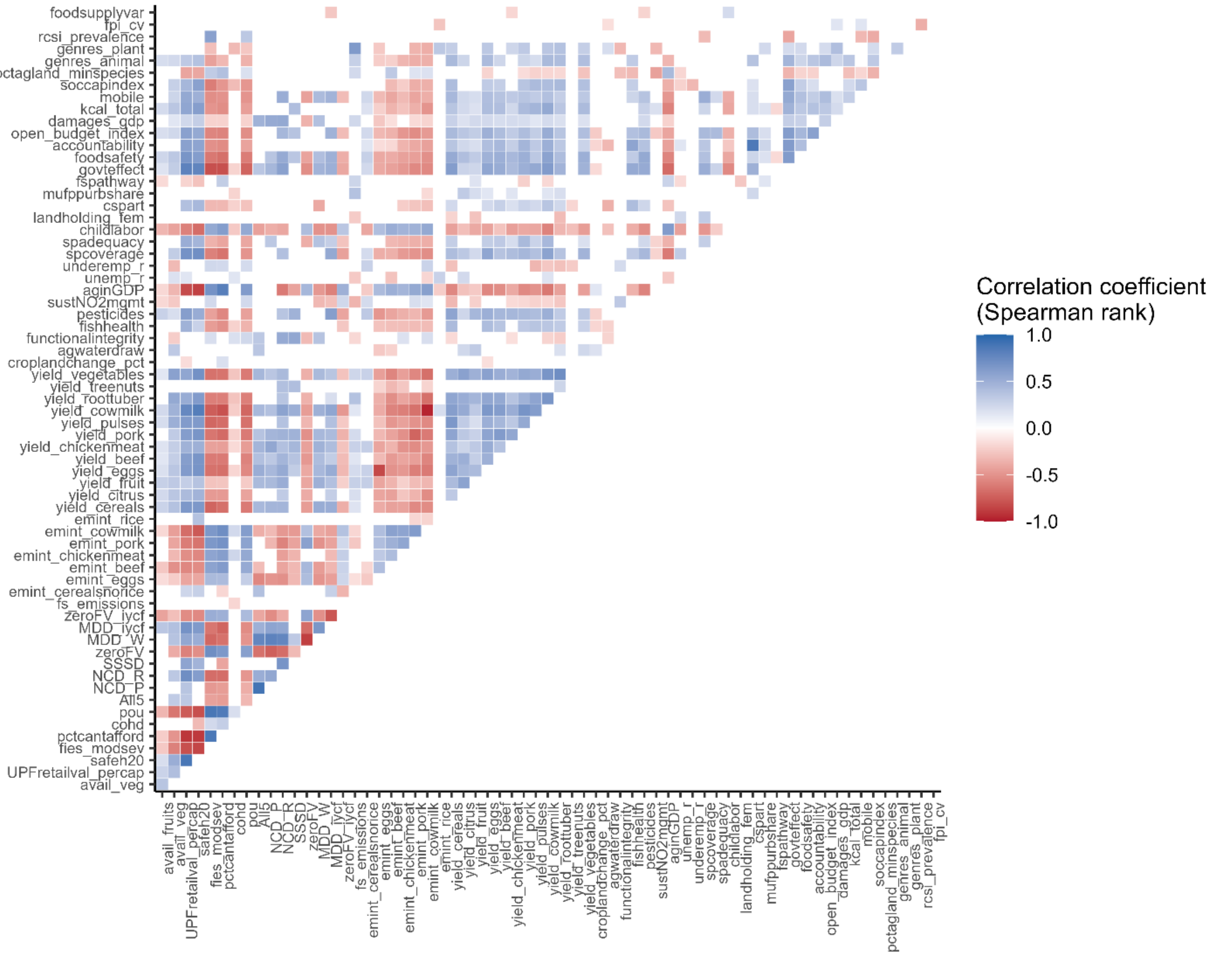

Supplementary Information, Appendix A: Supplementary Figures for all Indicators

Figures 1.1.1 to 1.5.16, visualization(s) for every indicator, by theme
All sources: Author's calculations based on data sources listed in **Extended Data Table 1**.

1.1.1 Distribution of the cost of the least-cost healthy diet ($PPP/capita/day) by region, 2017-2020

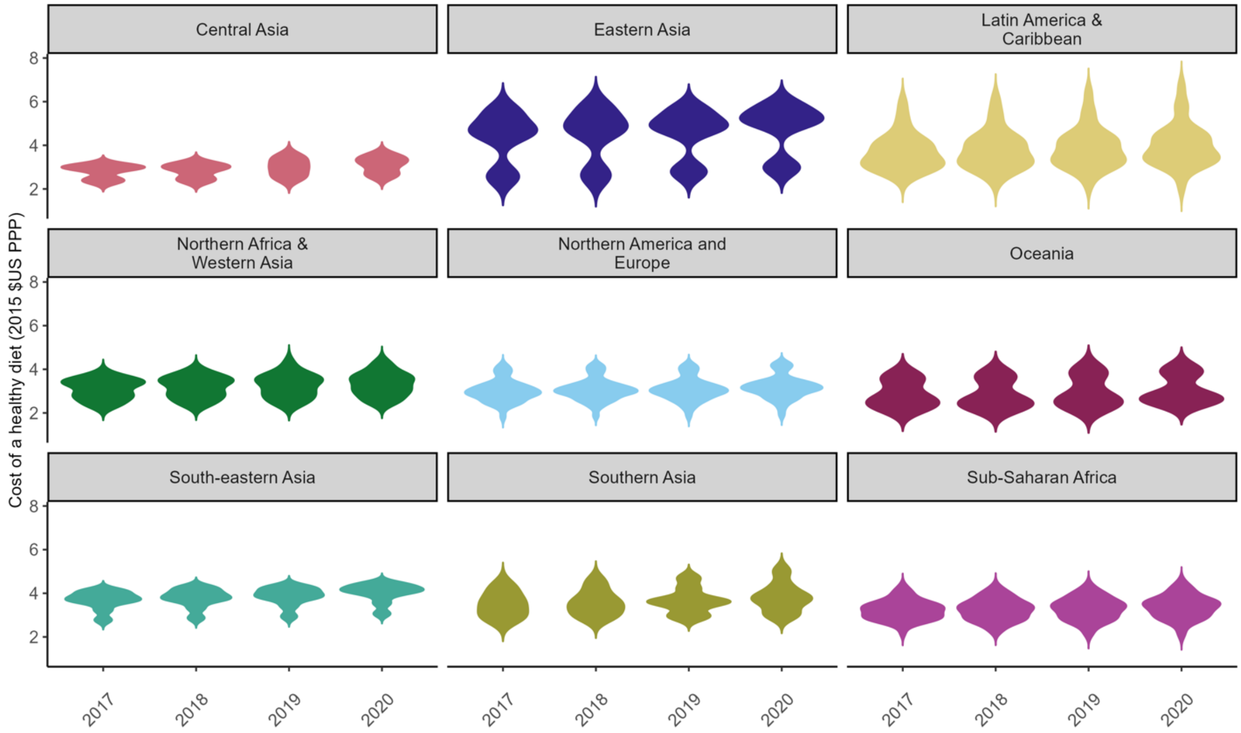

Violin plots reflect the distribution of the observations where the curved line and colored fill illustrate the probability distribution of the dat1. Sample size by year: 2017, N=157 countries; 2018-2020, N=155 countries. Sample size by region and year provided in the following table:

Region	2017	2018	2019	2020
Central Asia	3	3	3	3
Eastern Asia	4	4	4	4
Latin America & Caribbean	28	27	27	27
Northern Africa & Western Asia	18	18	18	18
Northern America and Europe	40	40	40	40
Oceania	3	3	3	3
South-eastern Asia	10	10	10	10
Southern Asia	8	8	8	8
Sub-Saharan Africa	43	42	42	42

1.1.2 Distribution of the cost of the least-cost healthy diet ($PPP/capita/day) by country income groups, 2017-2020

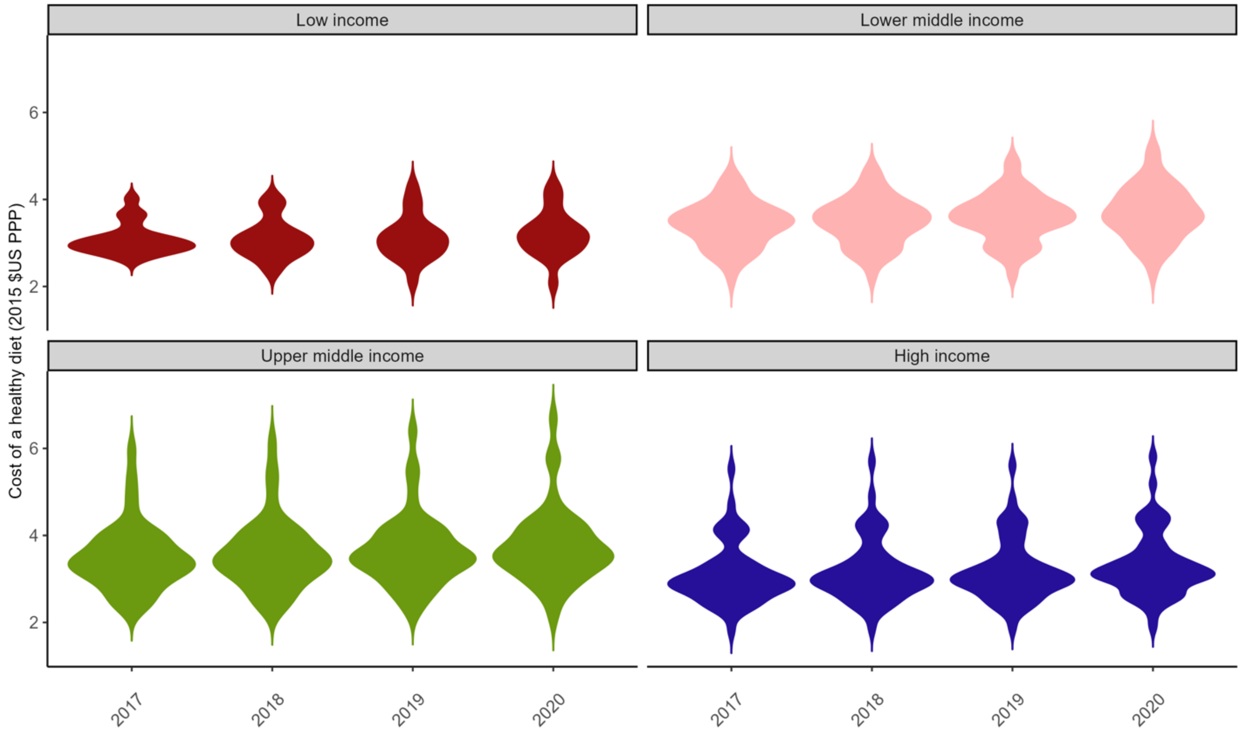

Sample size by year: 2017, N=157 countries; 2018-2020, N=155 countries. Sample size by income group and year provided in the following table:

**Income group**	**2017**	**2018**	**2019**	**2020**
Low income	20	20	20	20
Lower middle income	41	40	40	40
Upper middle income	43	42	42	42
High income	53	53	53	53

1.1.3 Change in fruit and vegetable availability, 2010-2019, by region

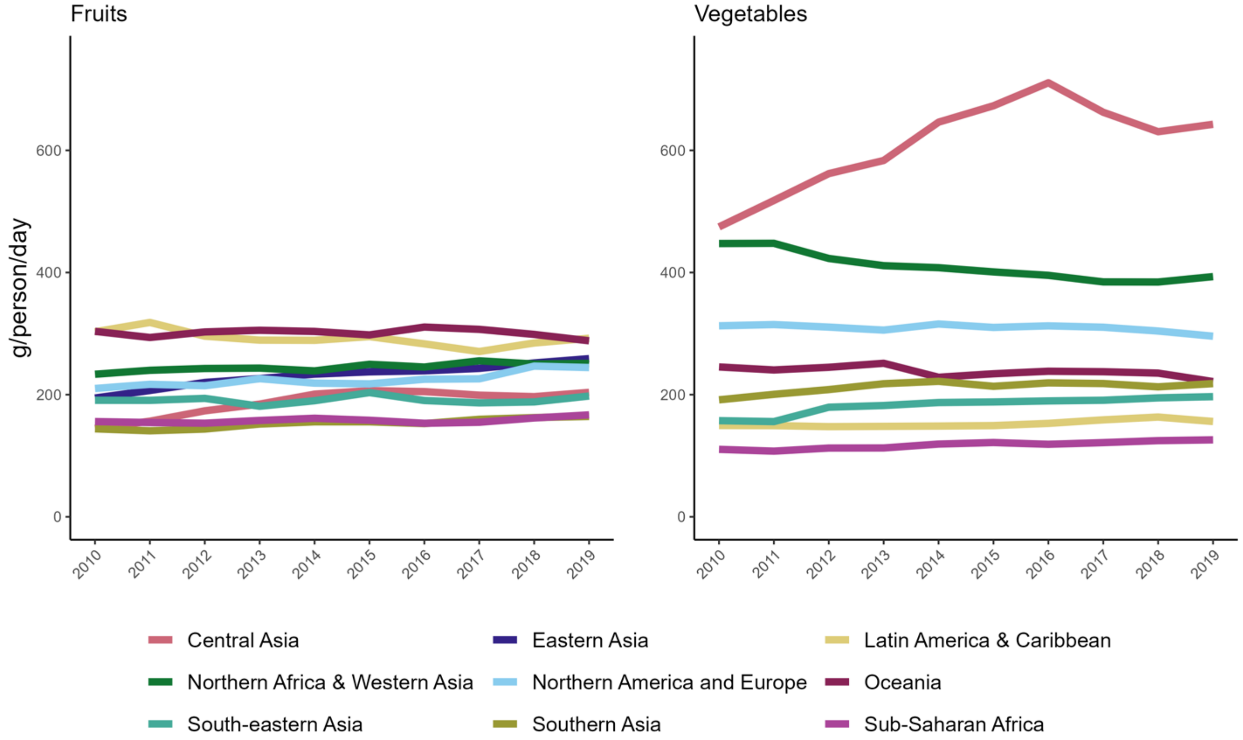

Unweighted regional means.

1.1.4 Change in fruit and vegetable availability, 2010-2019, by income group

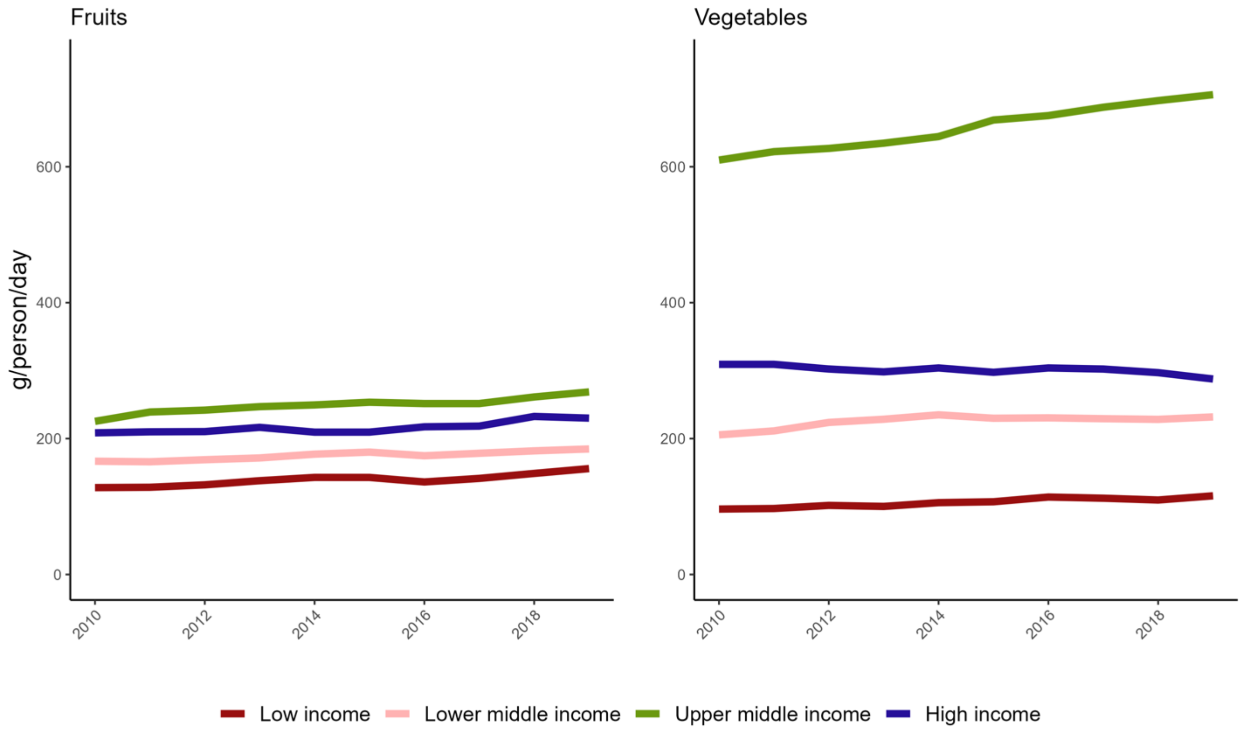

Unweighted income group means.

1.1.5 Retail value of Ultra-Processed Foods per capita, 2017-2019, by region

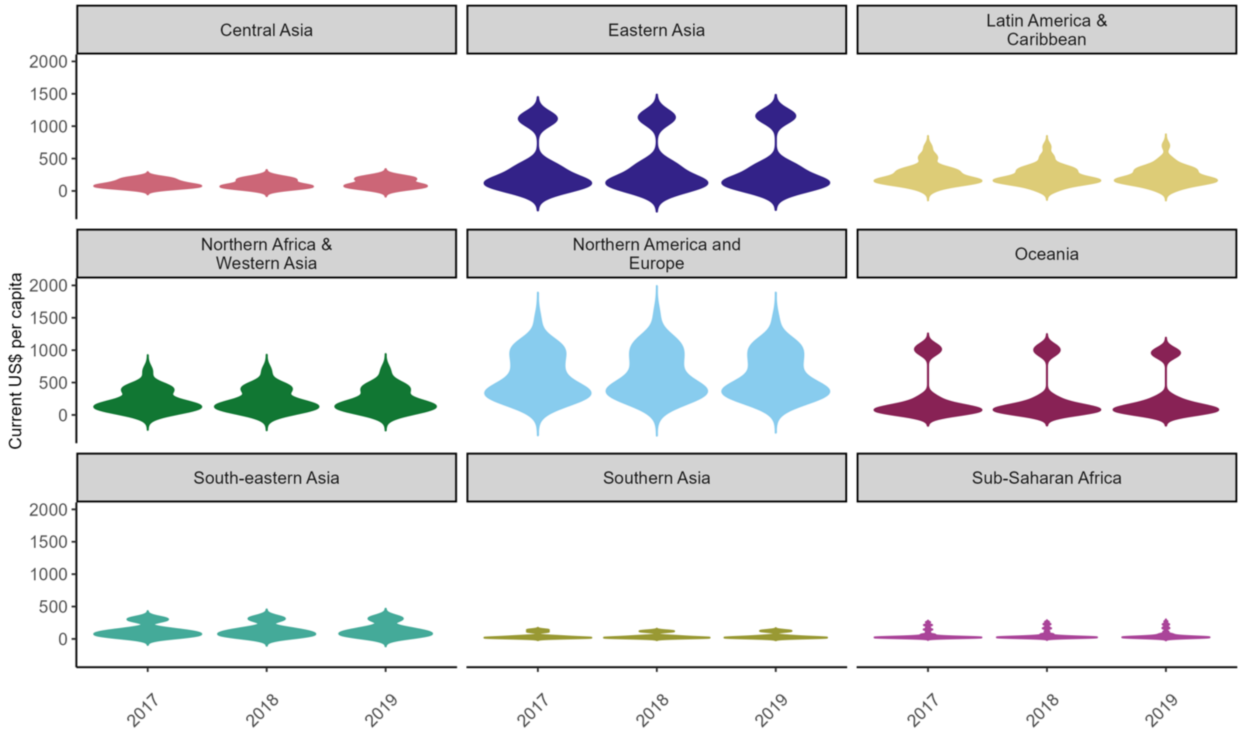

Sample size is 187 countries per year. Sample size by region and year provided in the following table:

Region	2017	2018	2019
Central Asia	5	5	5
Eastern Asia	5	5	5
Latin America & Caribbean	33	33	33
Northern Africa & Western Asia	23	23	23
Northern America and Europe	44	44	44
Oceania	11	11	11
South-eastern Asia	10	10	10
Southern Asia	9	9	9
Sub-Saharan Africa	47	47	47

1.1.6 Retail value of Ultra-Processed Foods per capita, 2019

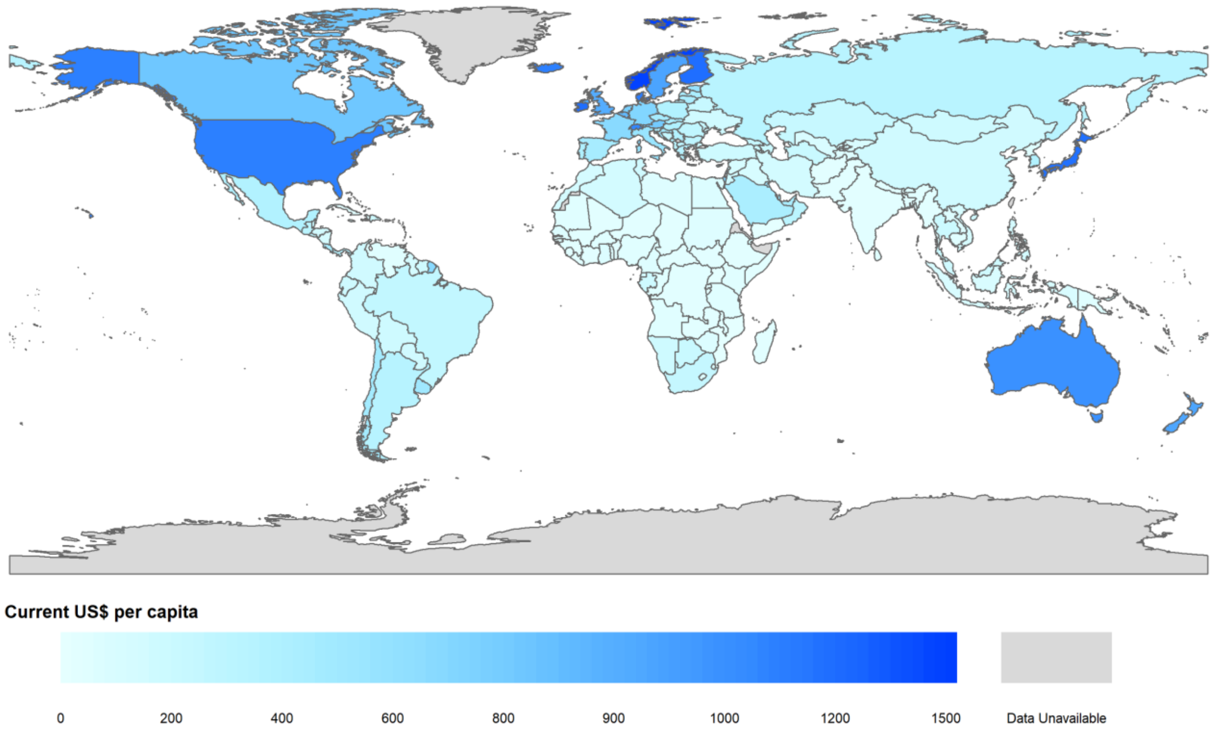

Basemap obtained using the R package ‘rnaturalearth’ which accesses Natural Earth v4.1.0 (March 2018) maps which are wholly in the public domain.

1.1.7 Percent population using safely managed drinking water services (SDG 6.1.1), 2000-2020, by region

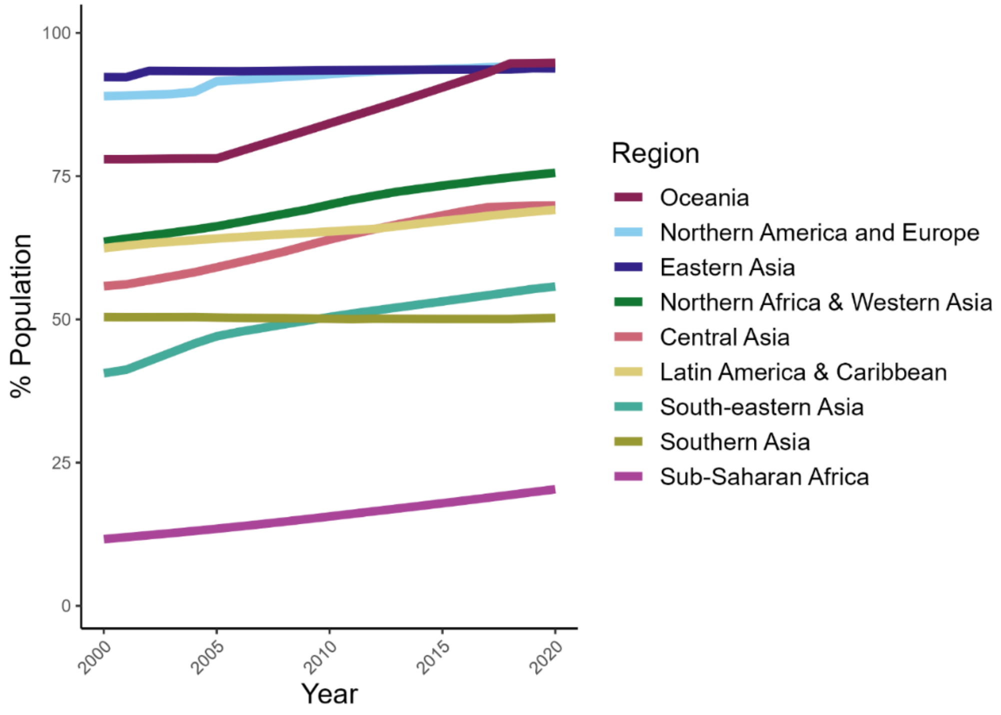

Population-weighted regional means.

1.1.8 Percent population using safely managed drinking water services (SDG 6.1.1), 2000-2020, by income group

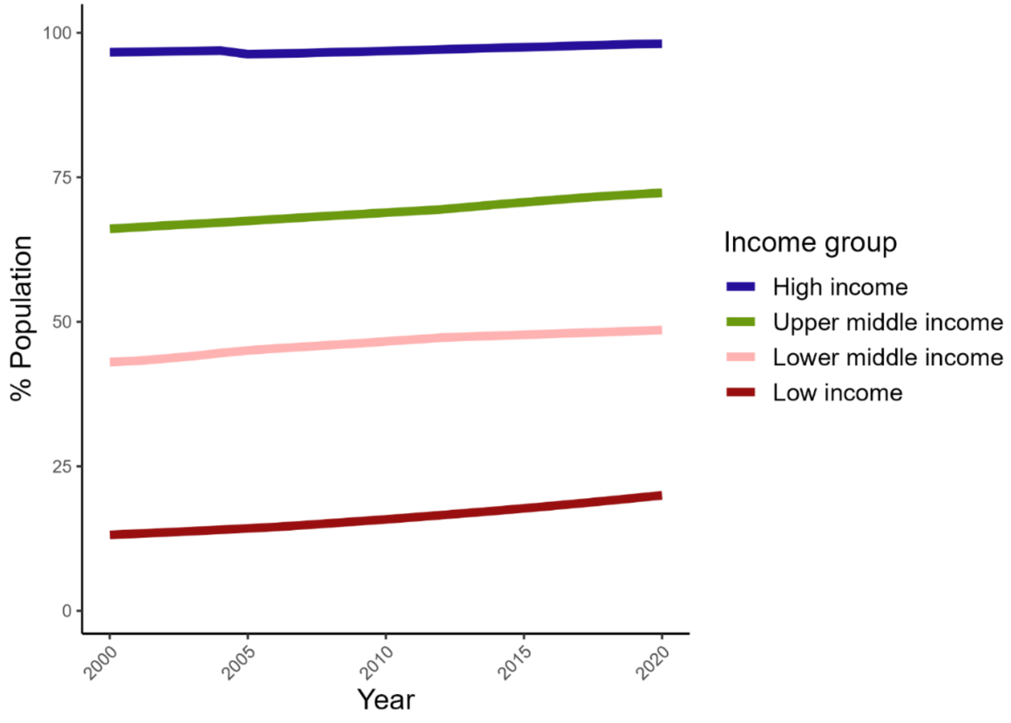

Population-weighted income group means.

1.1.9 Prevalence of Undernourishment, 2001-2020, by region

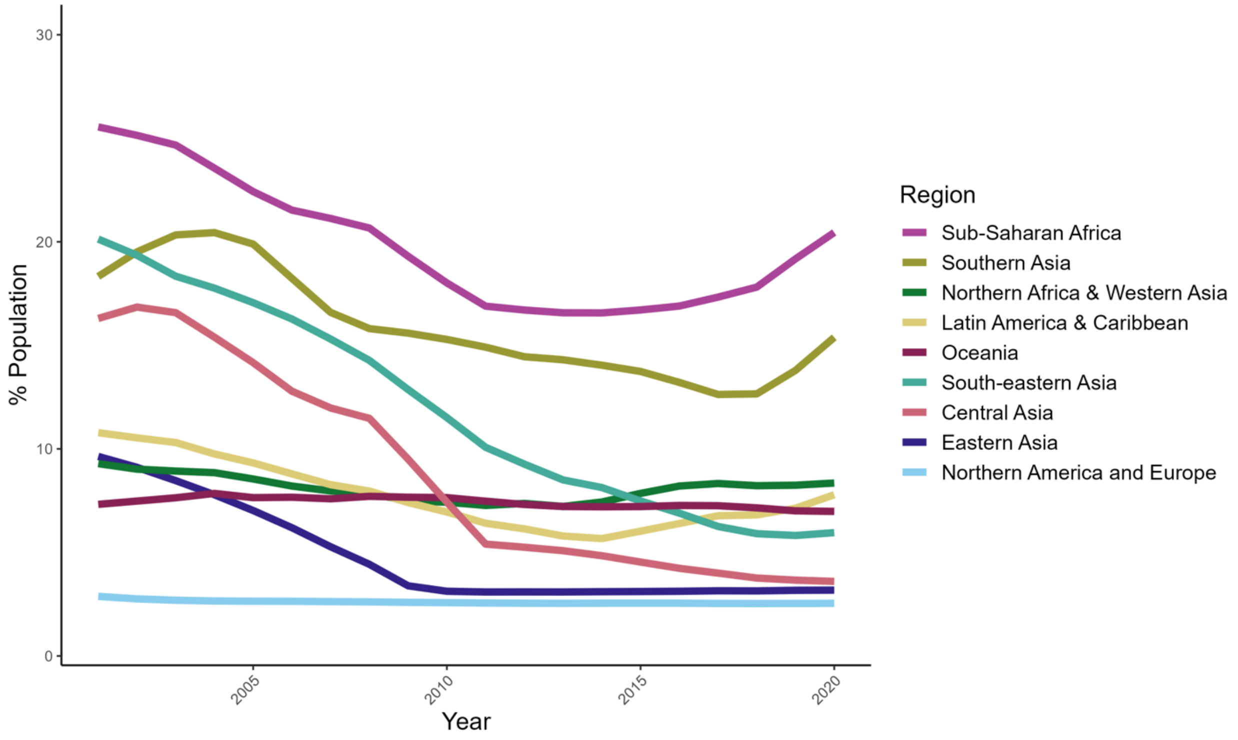

Annual value taken at the midpoint year of 3-year average. Population-weighted regional mean.

1.1.10 Prevalence of Undernourishment, 2001-2020, by income group

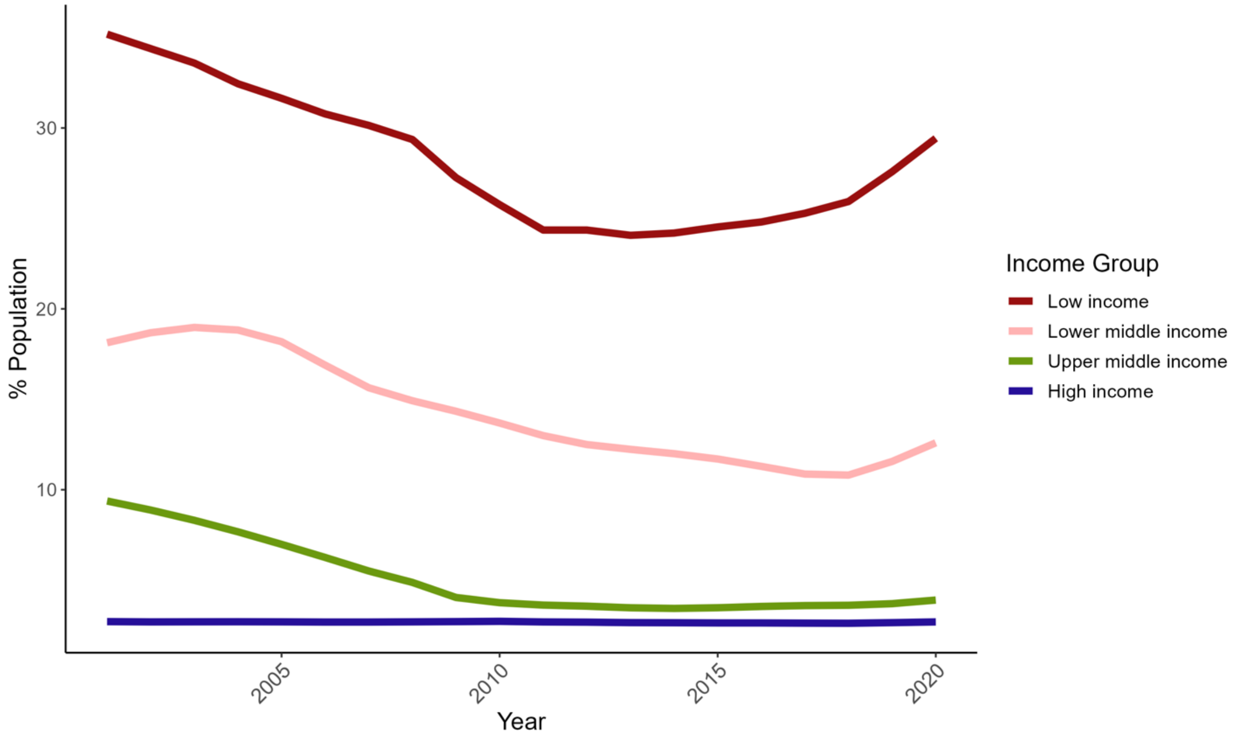

Annual value taken at the midpoint year of 3-year average. Population-weighted income group mean.

1.1.11 Percent population experiencing moderate or severe food insecurity, 2014-2020, by region

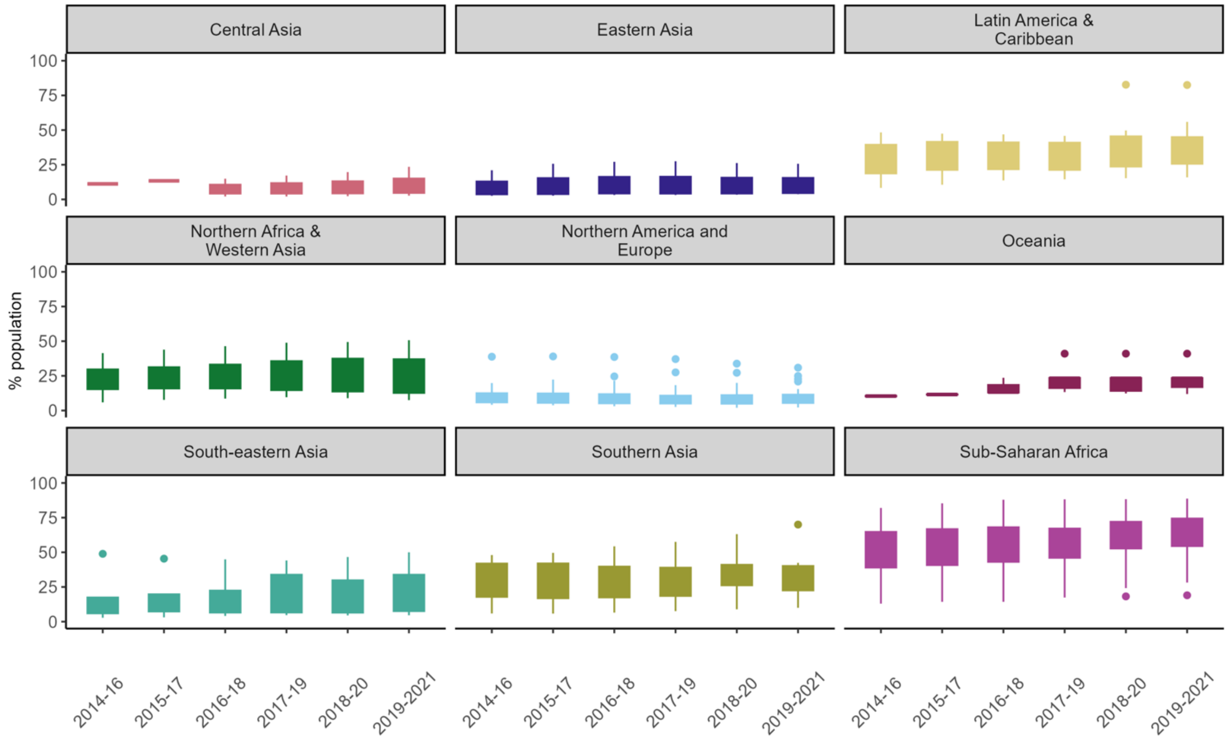

Annual value taken at the midpoint year of 3-year average. Boxplot illustrates a box of the 25th to 75th percentiles with whiskers (vertical lines) extend the length of 1.5 times the interquartile range. All outliers beyond 1.5 times the interquartile range are shown individually. Sample size (countries) by year and region provided in the following table:

**Region**	**2014-2016**	**2015-2017**	**2016-2018**	**2017-2019**	**2018-2020**	**2019-2021**
Central Asia	1	1	3	3	3	3
Eastern Asia	3	3	3	3	3	3
Latin America & Caribbean	15	15	17	17	18	23
Northern Africa & Western Asia	11	11	12	13	13	14
Northern America and Europe	39	39	39	39	40	40
Oceania	2	2	3	6	7	7
South-eastern Asia	5	5	6	7	9	9
Southern Asia	6	6	6	6	6	7
Sub-Saharan Africa	25	25	30	32	35	39

1.1.12 Percent population experiencing moderate or severe food insecurity, 2014-2020, by income group

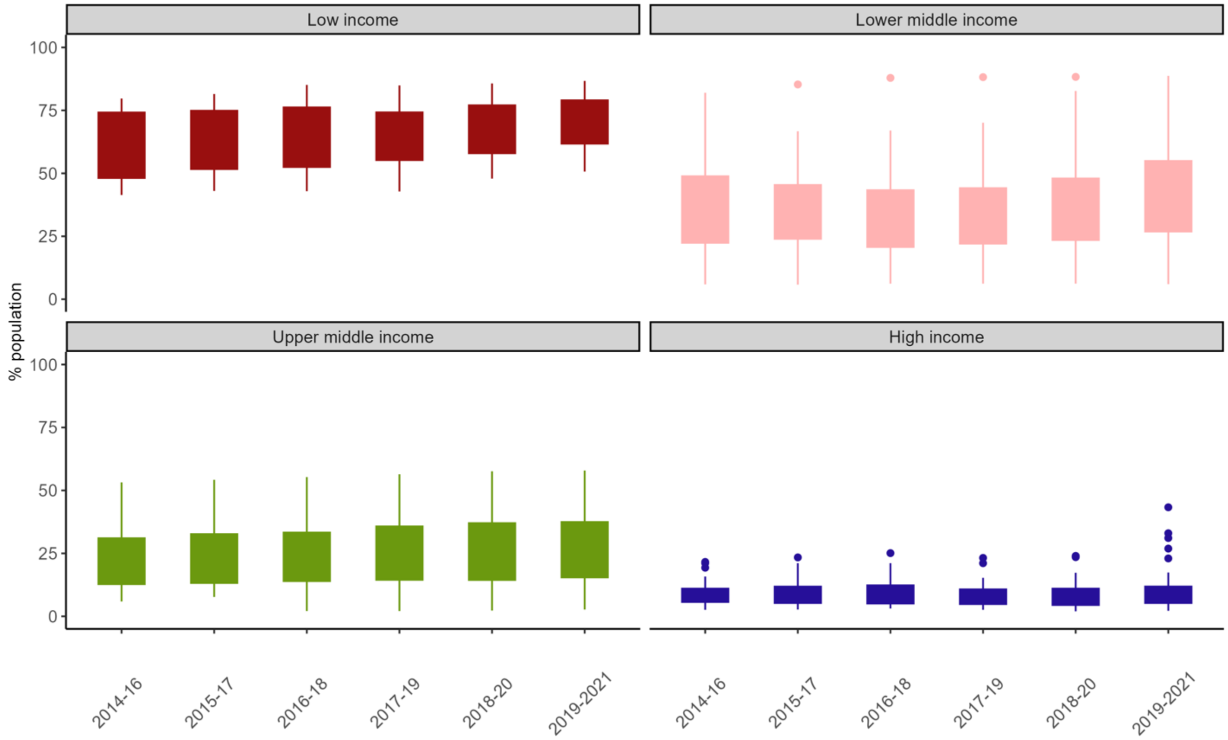

Annual value taken at the midpoint year of 3-year average. Boxplot illustrates a box of the 25th to 75th percentiles with whiskers (vertical lines) extend the length of 1.5 times the interquartile range. All outliers beyond 1.5 times the interquartile range are shown individually. Sample size (countries) by year and income group provided in the following table:

**Income group**	**2014-2016**	**2015-2017**	**2016-2018**	**2017-2019**	**2018-2020**	**2019-2021**
Low income	11	12	16	16	19	19
Lower middle income	28	27	31	37	40	44
Upper middle income	27	27	31	33	34	36
High income	41	41	41	40	41	46

1.1.13 Percent population who cannot afford a healthy diet, 2017-2020, by region

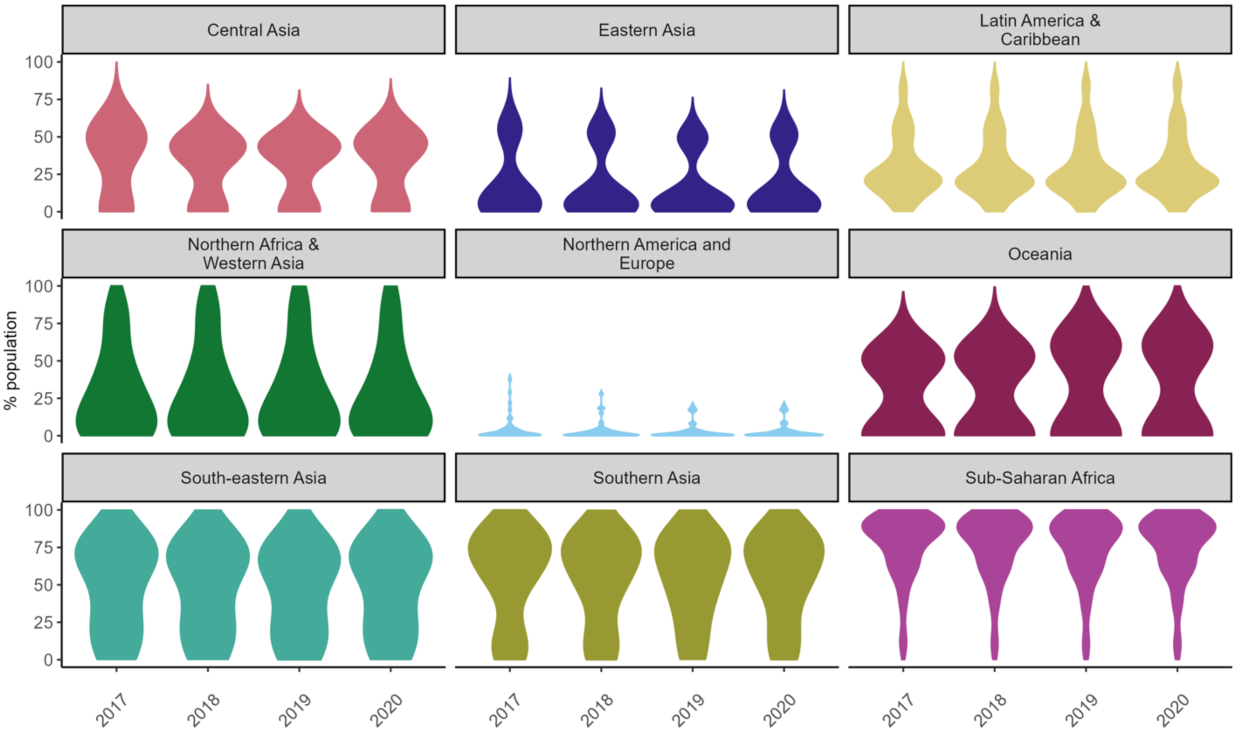

Sample size (countries) by year and region provided in the following table:

Region	2017	2018	2019	2020
Central Asia	3	3	3	3
Eastern Asia	4	4	4	4
Latin America & Caribbean	22	21	21	21
Northern Africa & Western Asia	13	13	13	13
Northern America and Europe	40	40	40	40
Oceania	2	2	2	2
South-eastern Asia	7	7	7	7
Southern Asia	8	8	8	8
Sub-Saharan Africa	42	41	41	41

1.1.14 Percent population who cannot afford a healthy diet, 2017-2020, by income group

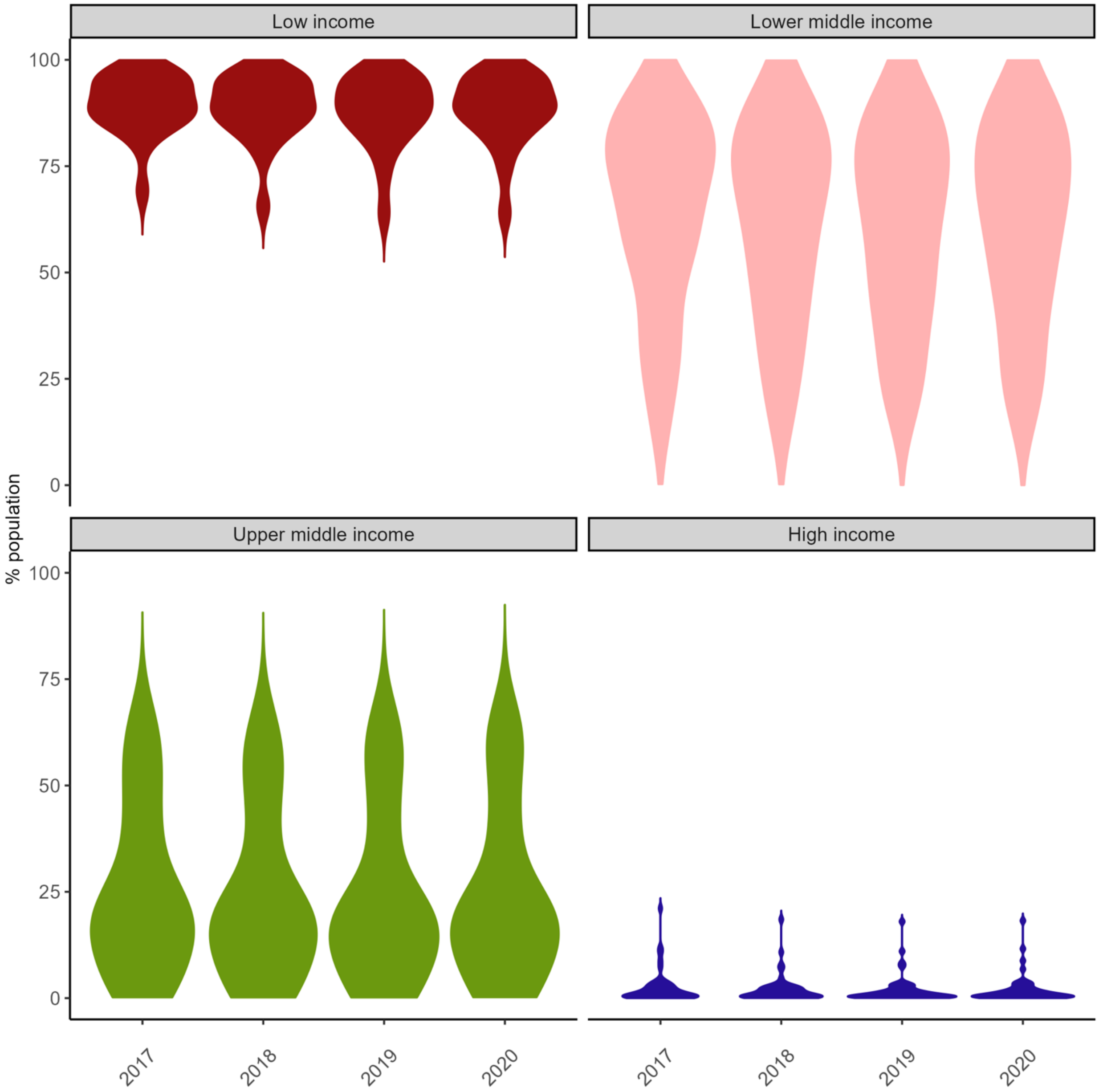

Sample size (countries) by year and income group provided in the following table:

Income group	2017	2018	2019	2020
Low income	20	20	20	20
Lower middle income	40	39	39	39
Upper middle income	39	38	38	38
High income	42	42	42	42

1.1.15 Percent adult women (15-49 y) meeting minimum dietary diversity, 2021

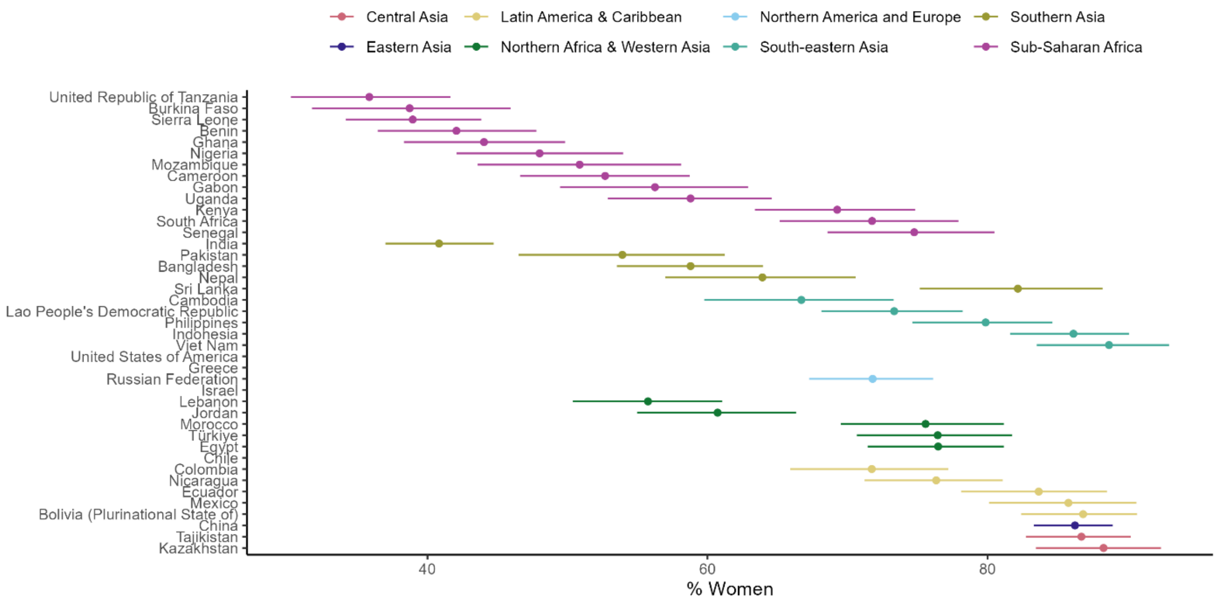

Legend bar shows the 95% confidence interval around the mean point estimate. Sample size is 41 countries.

1.1.16 Percent of children 6-23 months meeting minimum dietary diversity, by sex

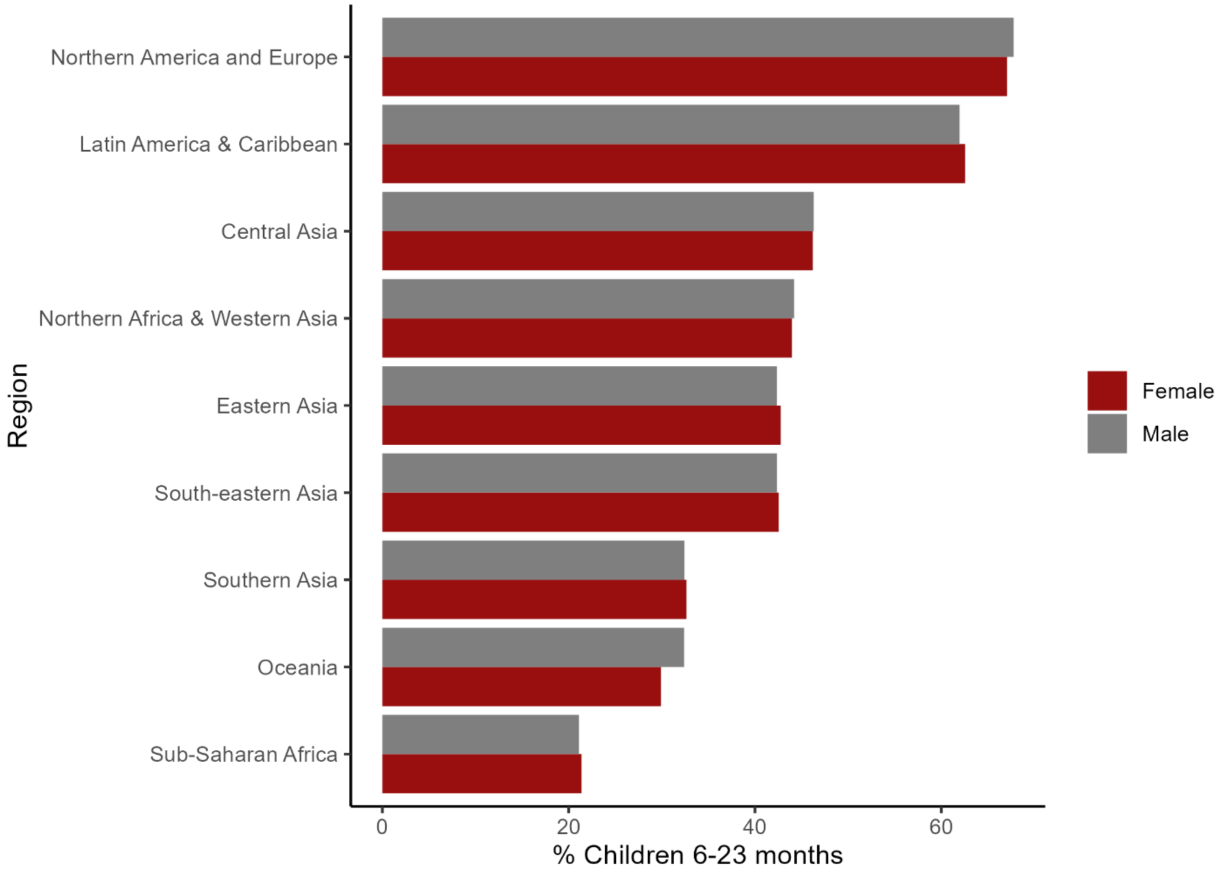

Data source year differs by country. See table A1.2.

1.1.17 Percent of children 6-23 months meeting minimum dietary diversity, by urban/rural

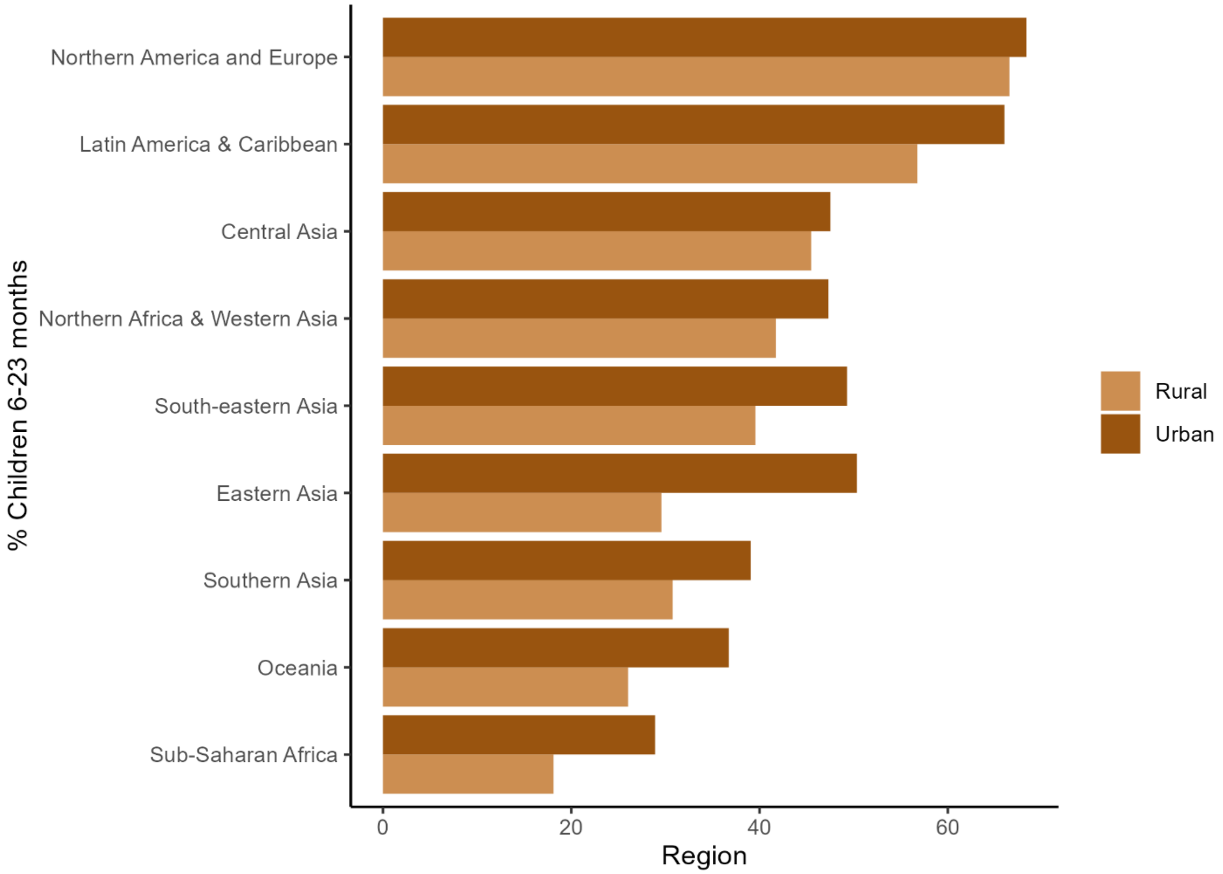

Data source year differs by country. See table A1.2.

1.1.18 Percent adult population (≥15 y) consuming all 5 food groups, 2021

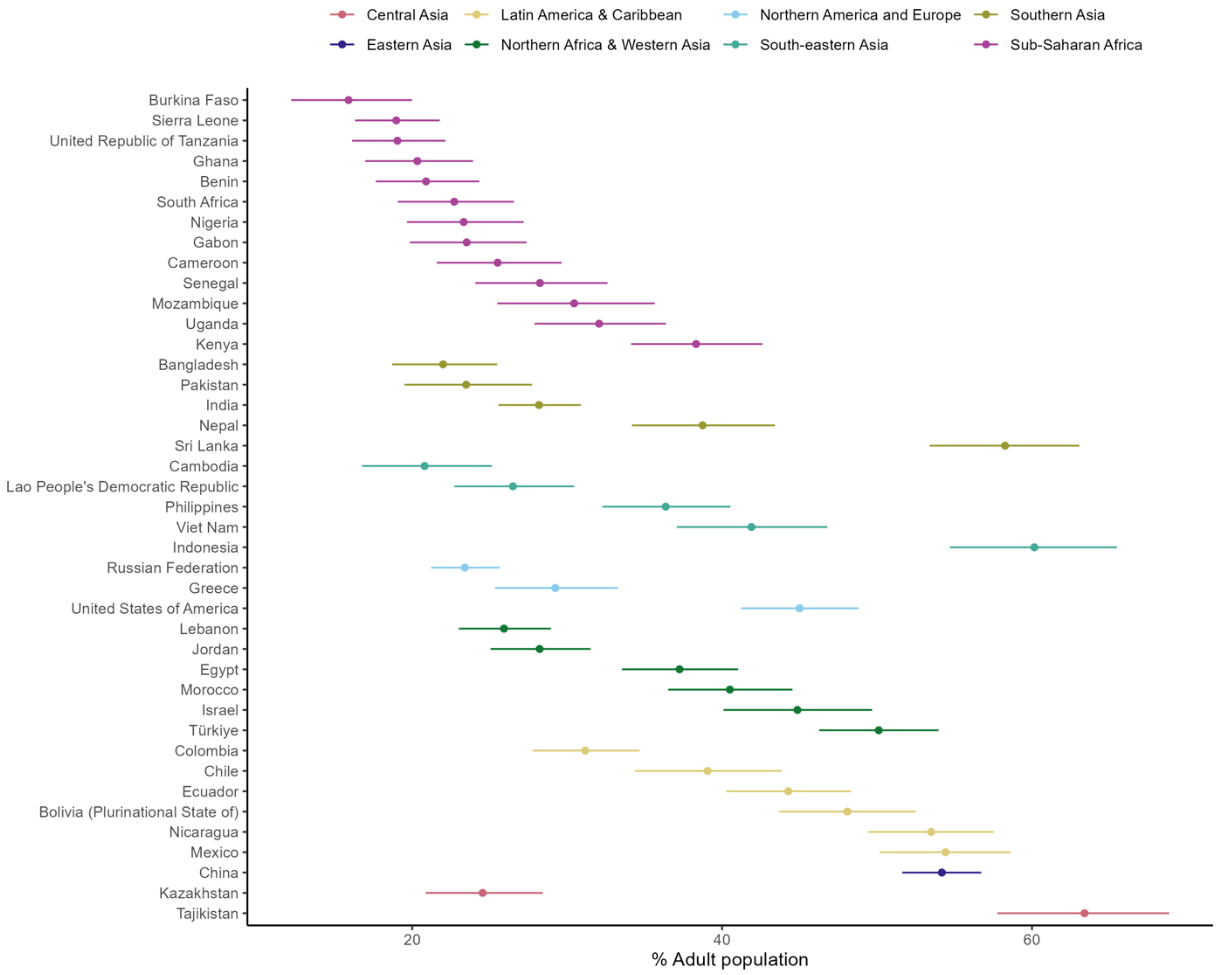

Legend bar shows the 95% confidence interval around the mean point estimate. Sample size is 41 countries.

1.1.19 Percent adult population (≥15 y) with zero fruits and vegetables consumption, 2021

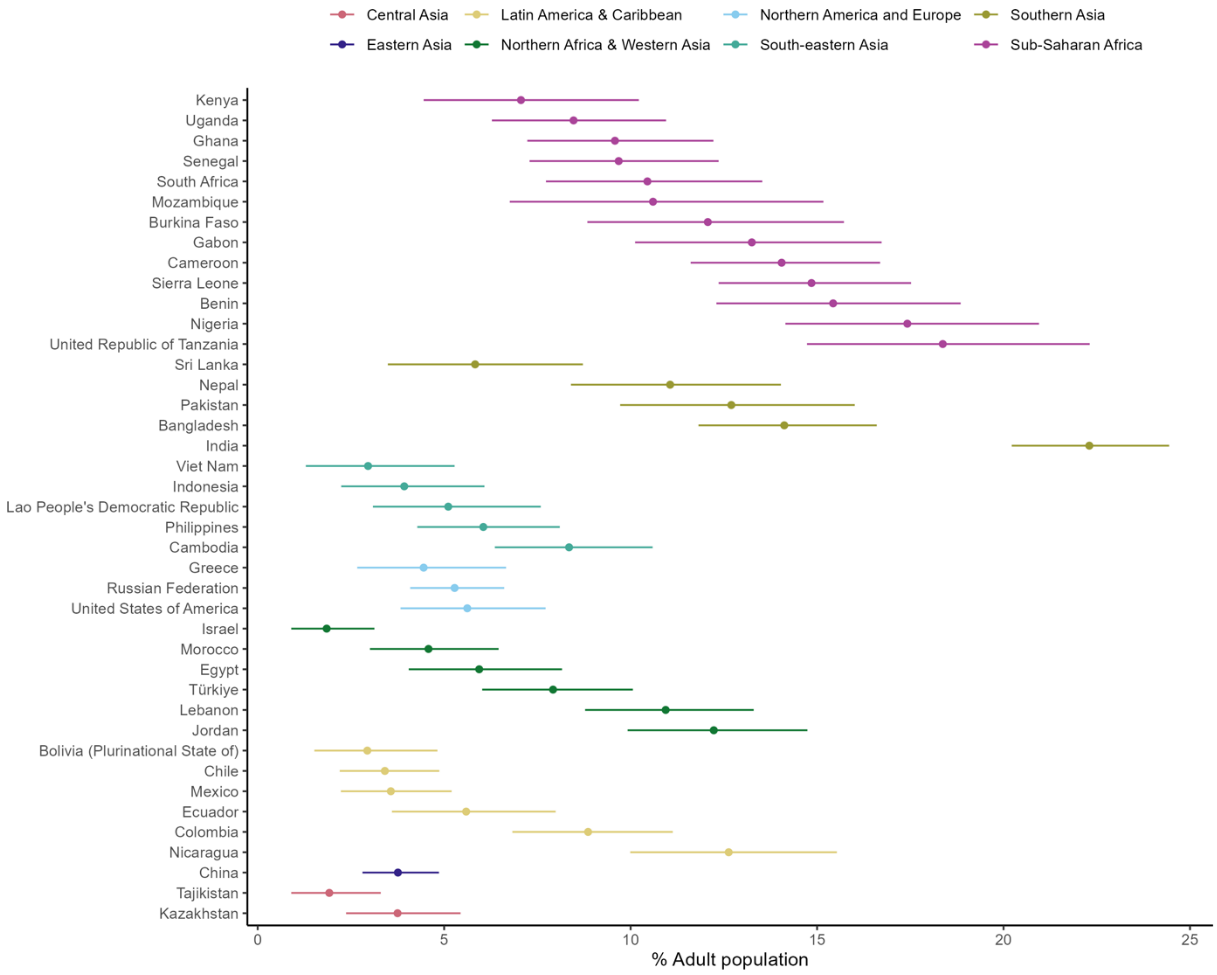

Legend bar shows the 95% confidence interval around the mean point estimate. Sample size is 41 countries.

1.1.20 Prevalence of zero fruits and vegetables consumption, children 6-23 months, by sex

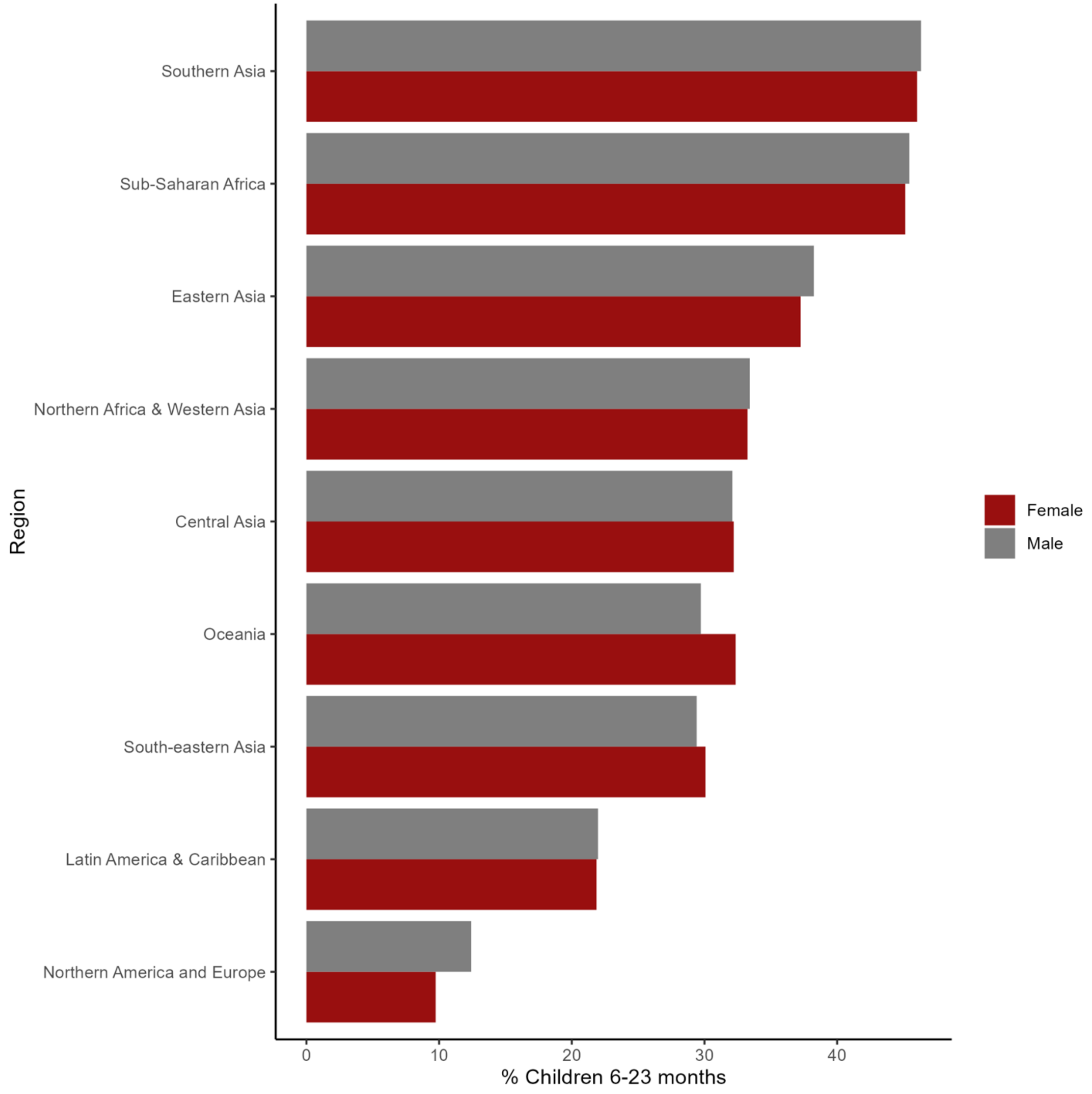

Data source year differs by country. See table A1.2.

1.1.21 Prevalence of zero fruits and vegetables consumption, children 6-23 months, by urban/rural

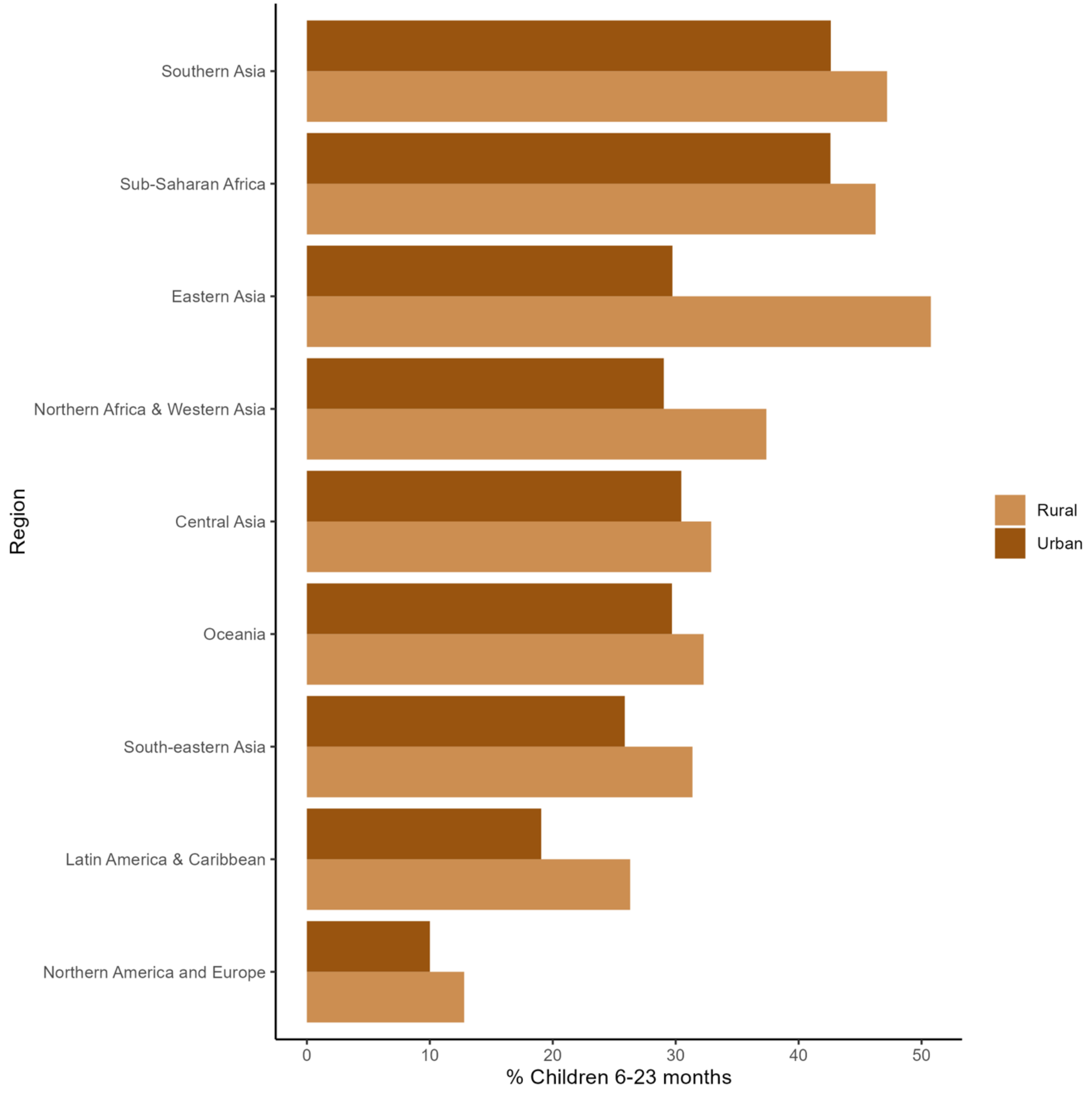

Data source year differs by country. See table A1.2.

1.1.22 NCD-Protect, adults (≥15 y), 2021

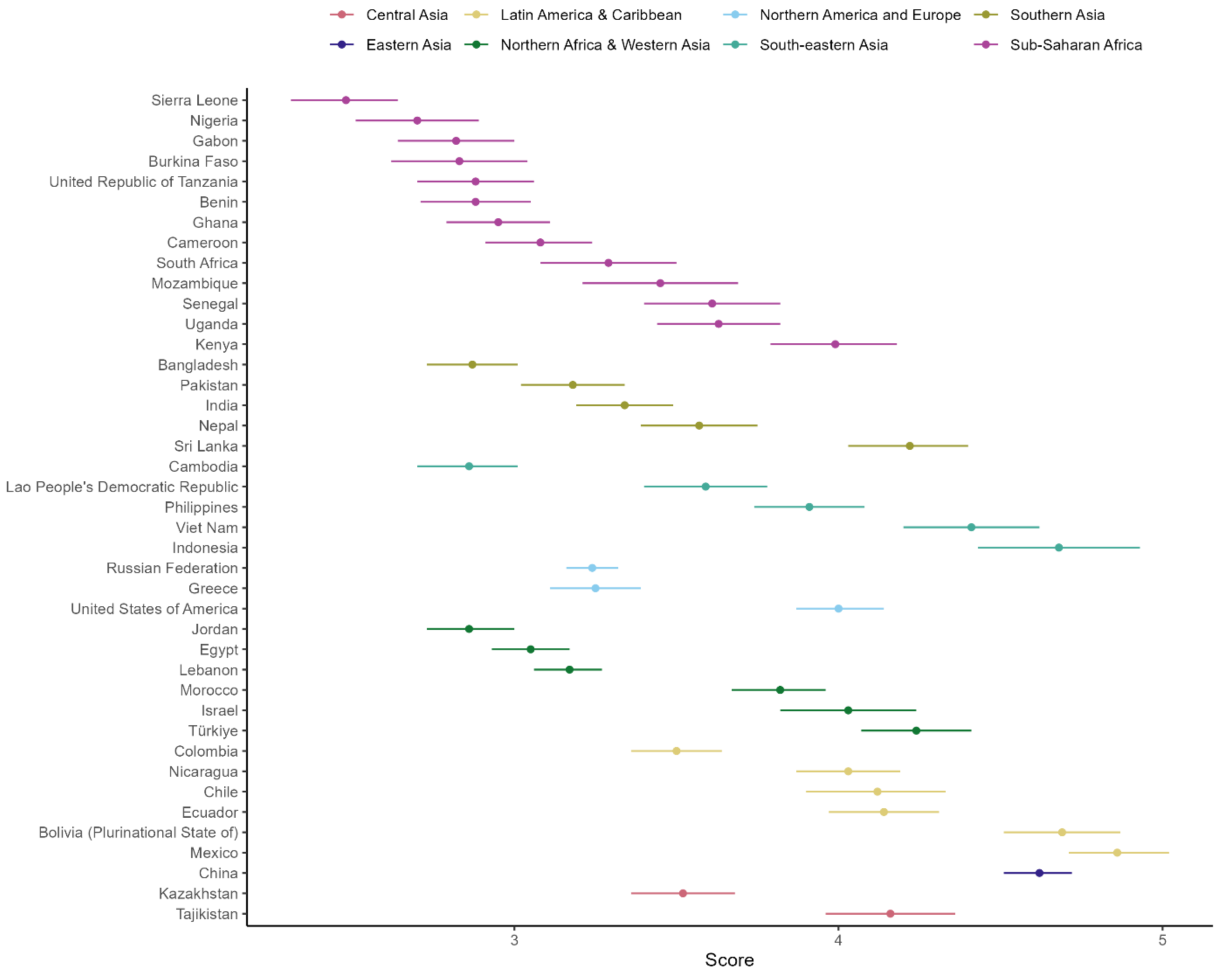

Legend bar shows the 95% confidence interval around the mean point estimate. Sample size is 41 countries.

1.1.23 NCD-Risk, adults (≥15 y), 2021

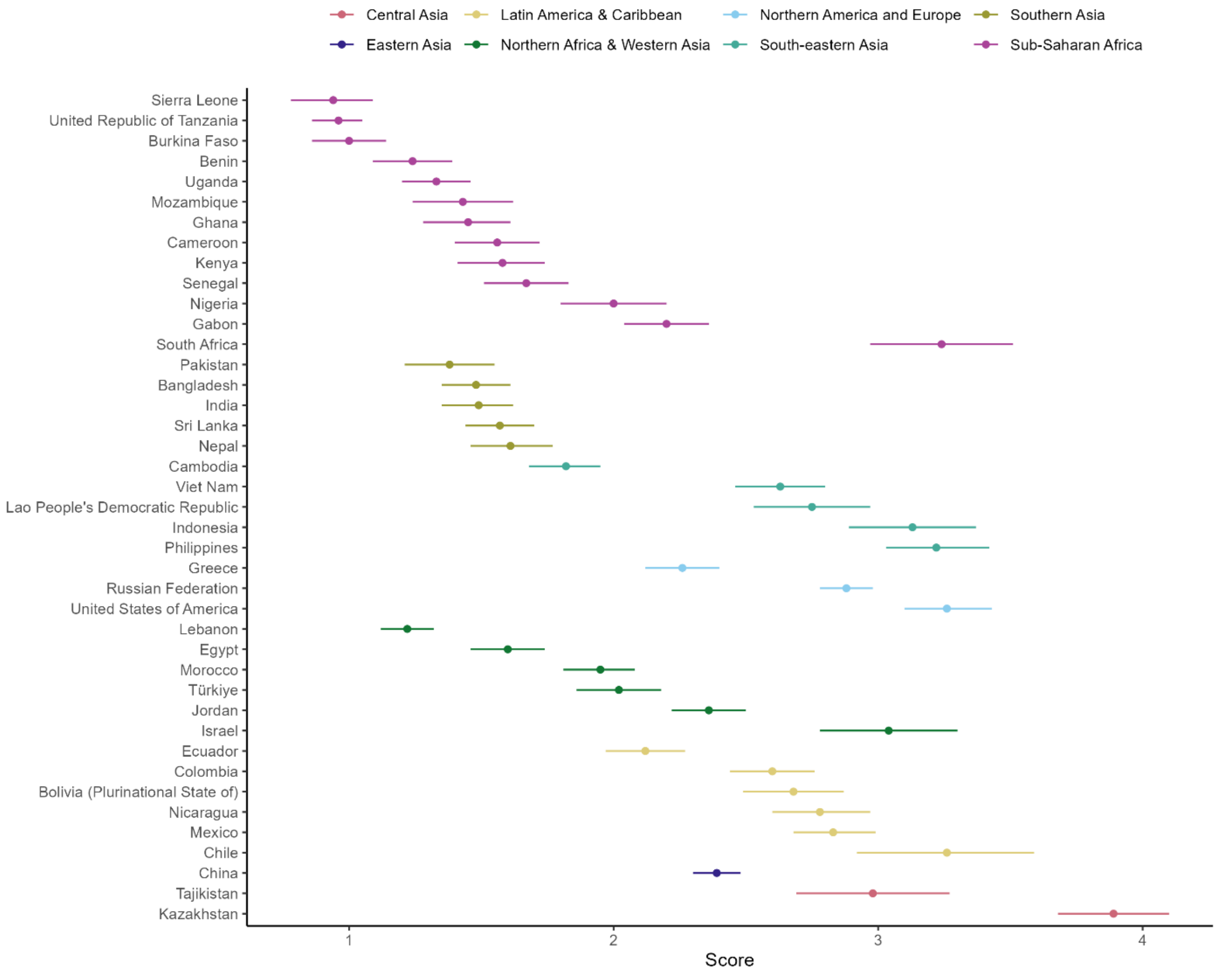

Legend bar shows the 95% confidence interval around the mean point estimate. Sample size is 41 countries.

1.1.24 Prevalence of sugar-sweetened soft drink consumption, adults (≥15 y), 2021

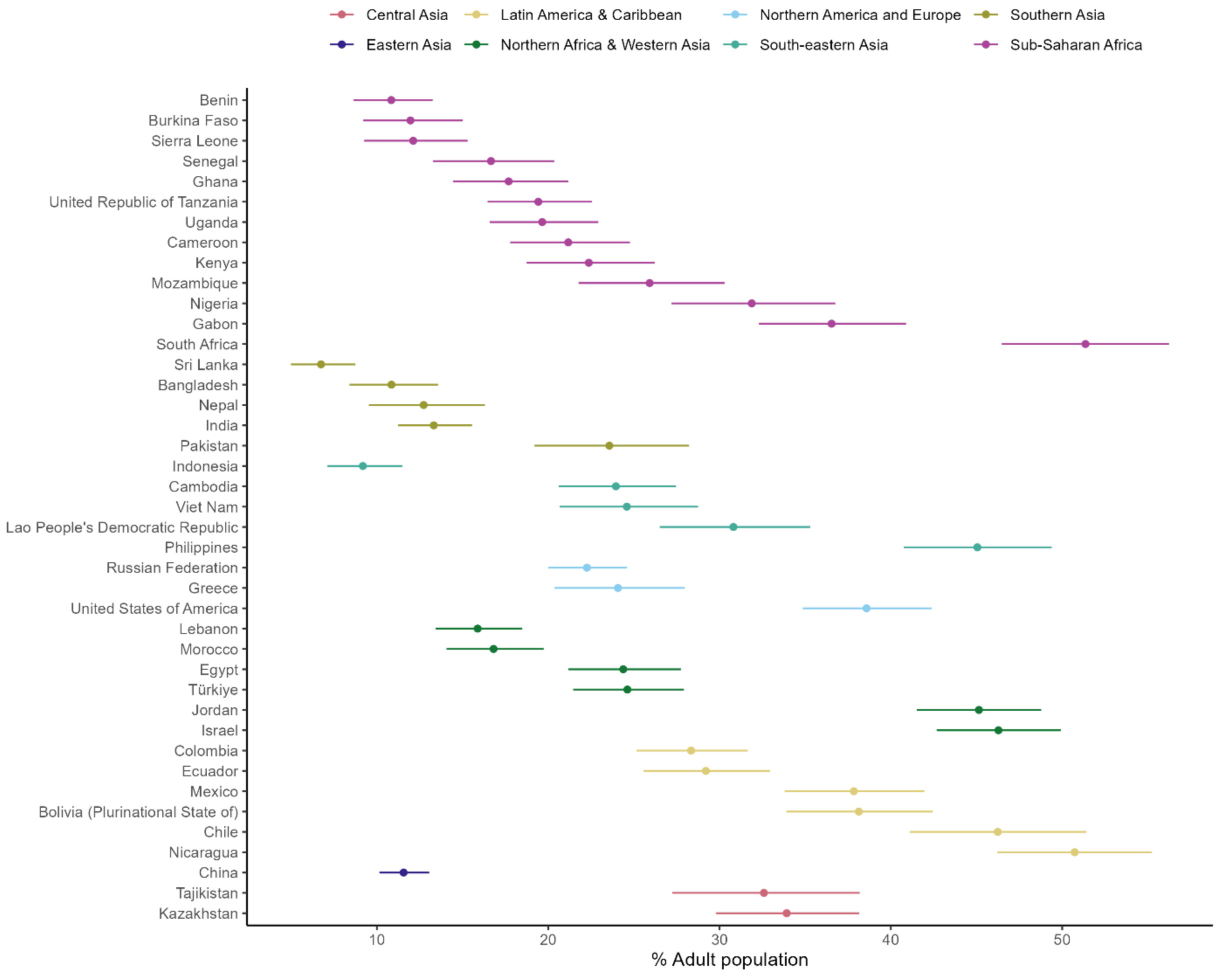

Legend bar shows the 95% confidence interval around the mean point estimate. Sample size is 41 countries.

1.2.1 Total food system emissions, 2000-2020, by region

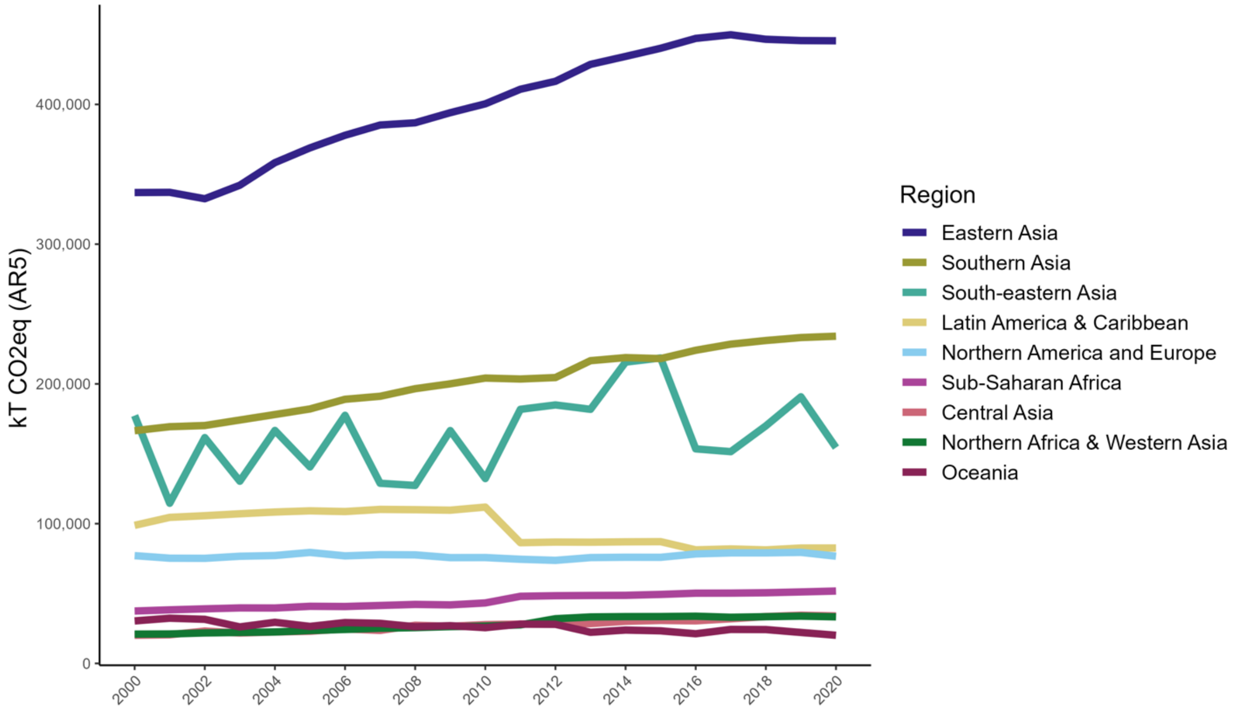

Unweighted mean by region.

1.2.2 Total food system emissions, 2020

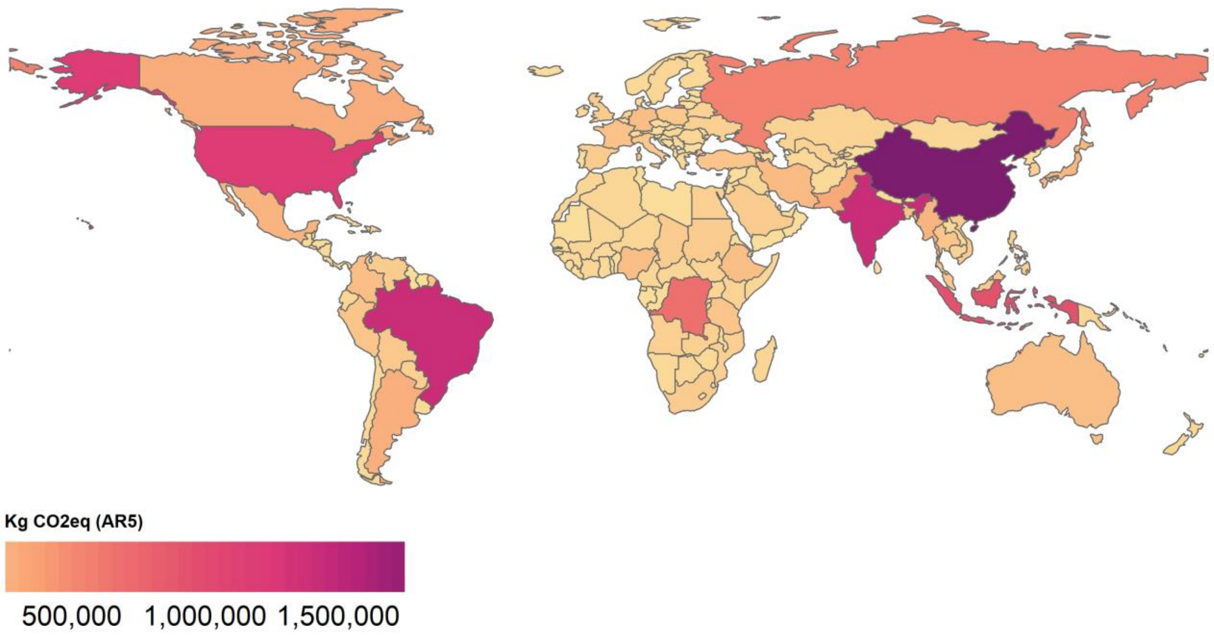

Basemap obtained using the R package ‘rnaturalearth’ which accesses Natural Earth v4.1.0 (March 2018) maps which are wholly in the public domain.

1.2.3 Emissions intensity (kg CO_2eq/kg product), Staple foods, 2000-2020, by region

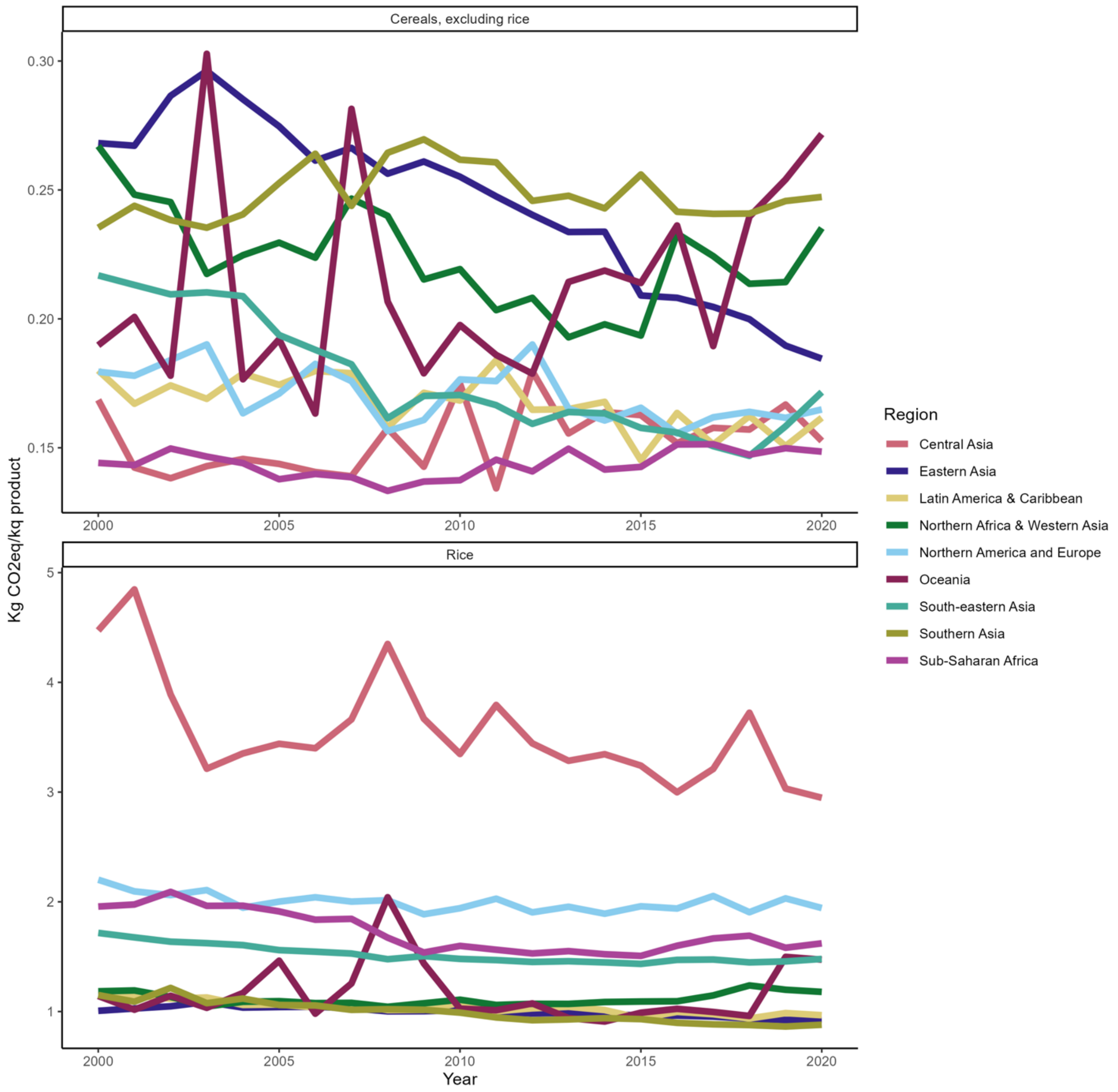

Production-weighted mean by region.

1.2.4 Emissions intensity (kg CO_2eq/kg product), Meats, 2000-2020, by region

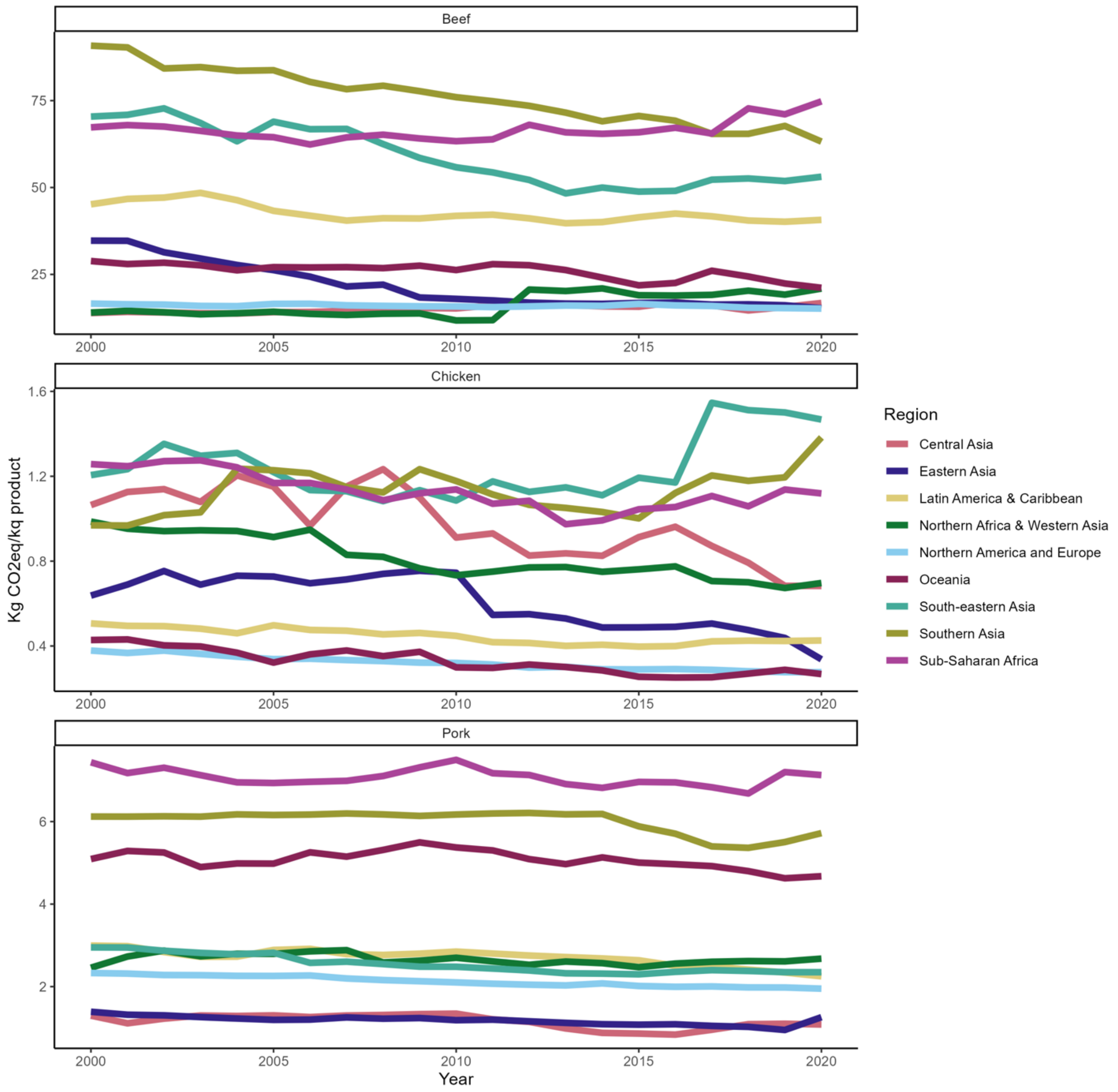

Production-weighted mean by region.

1.2.5 Emissions intensity (kg CO_2eq/kg product), Other animal-source foods, 2000-2020, by region

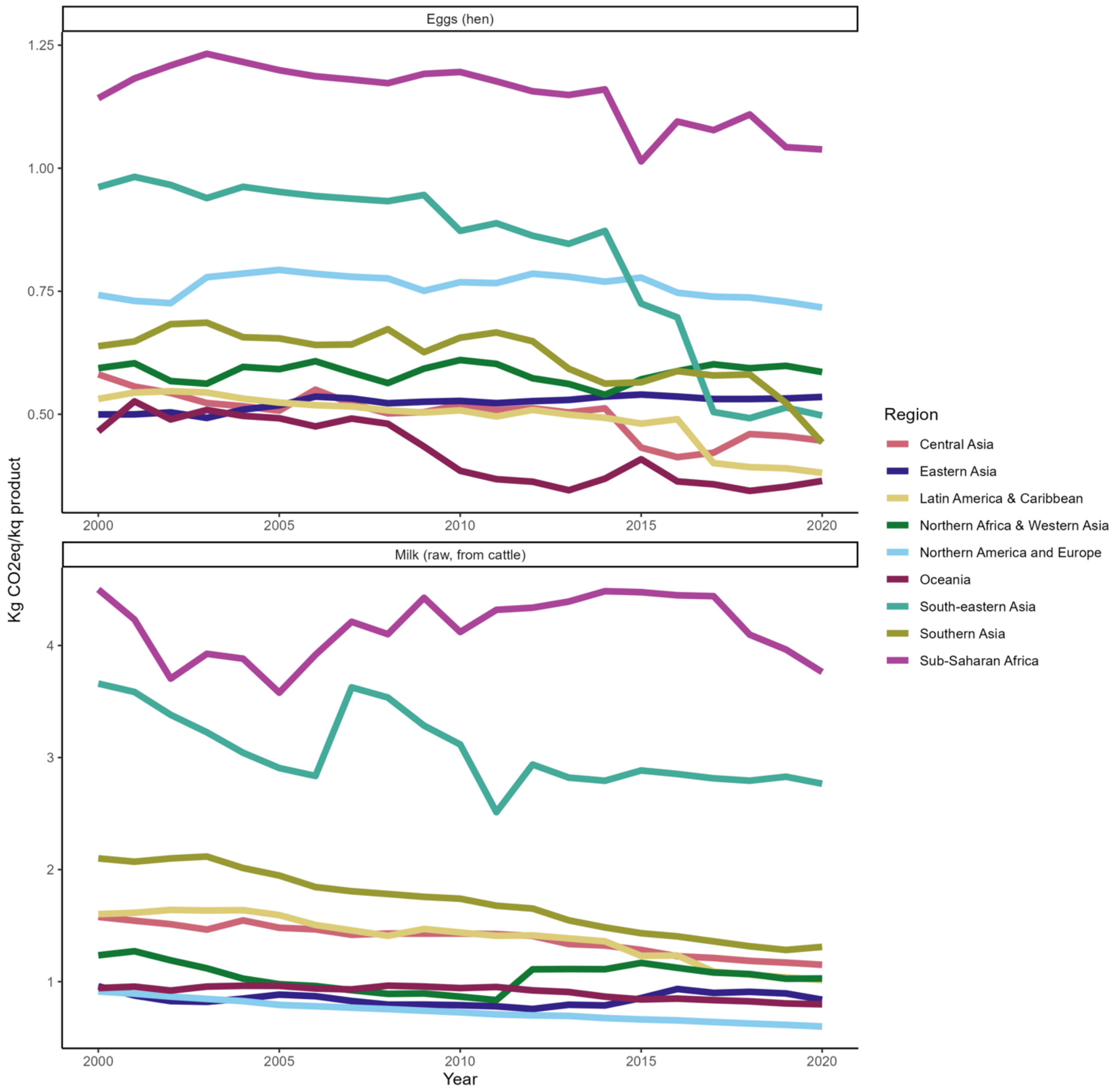

Production-weighted mean by region.

1.2.6 Yield, Staple foods, 2000-2020, by region

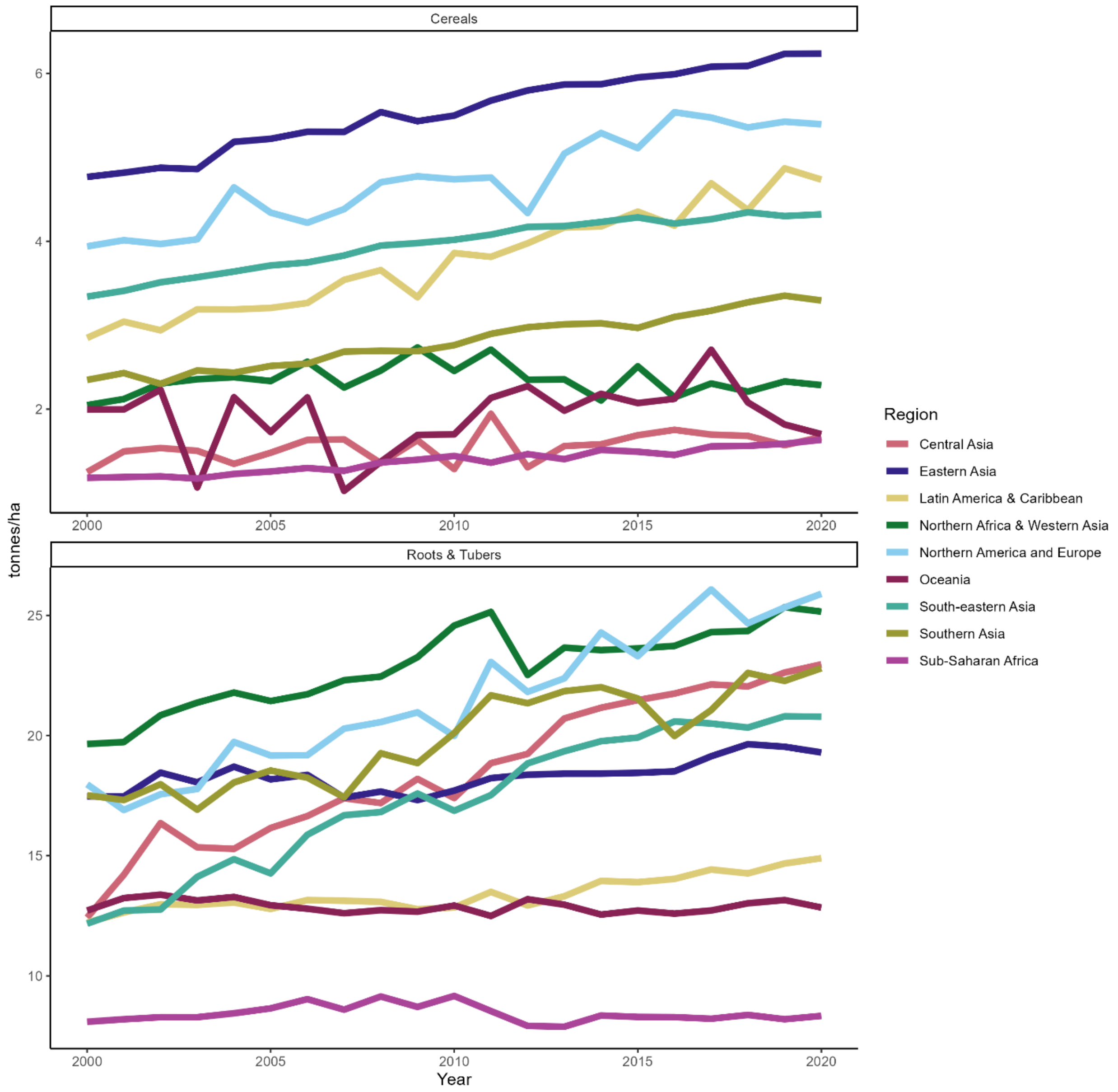

Area harvested-weighted mean by region.

1.2.7 Yield, Meat, 2000-2020, by region

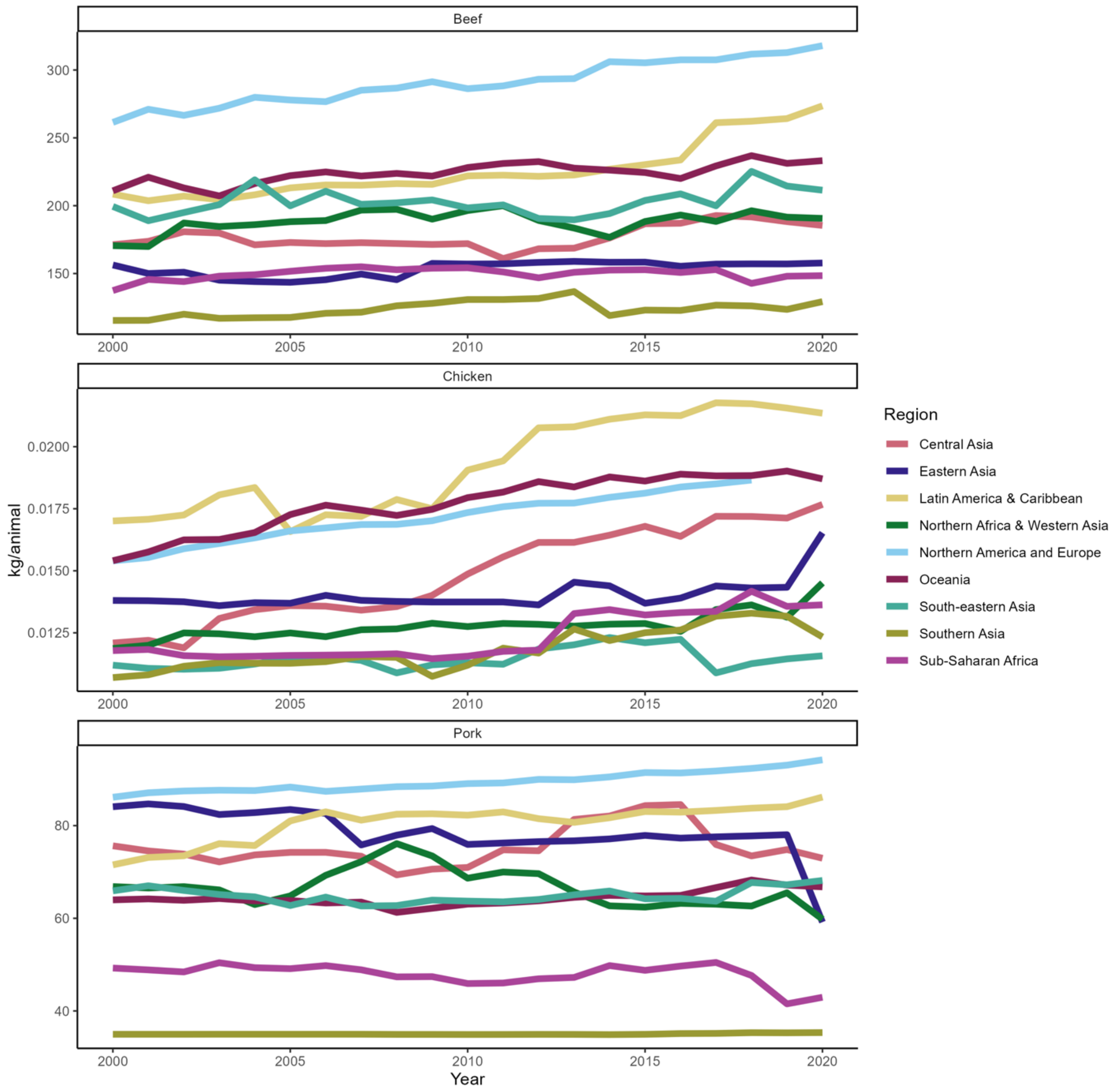

Producing animals-weighted mean by region.

1.2.8 Yield, Other animal-source foods, 2000-2020, by region

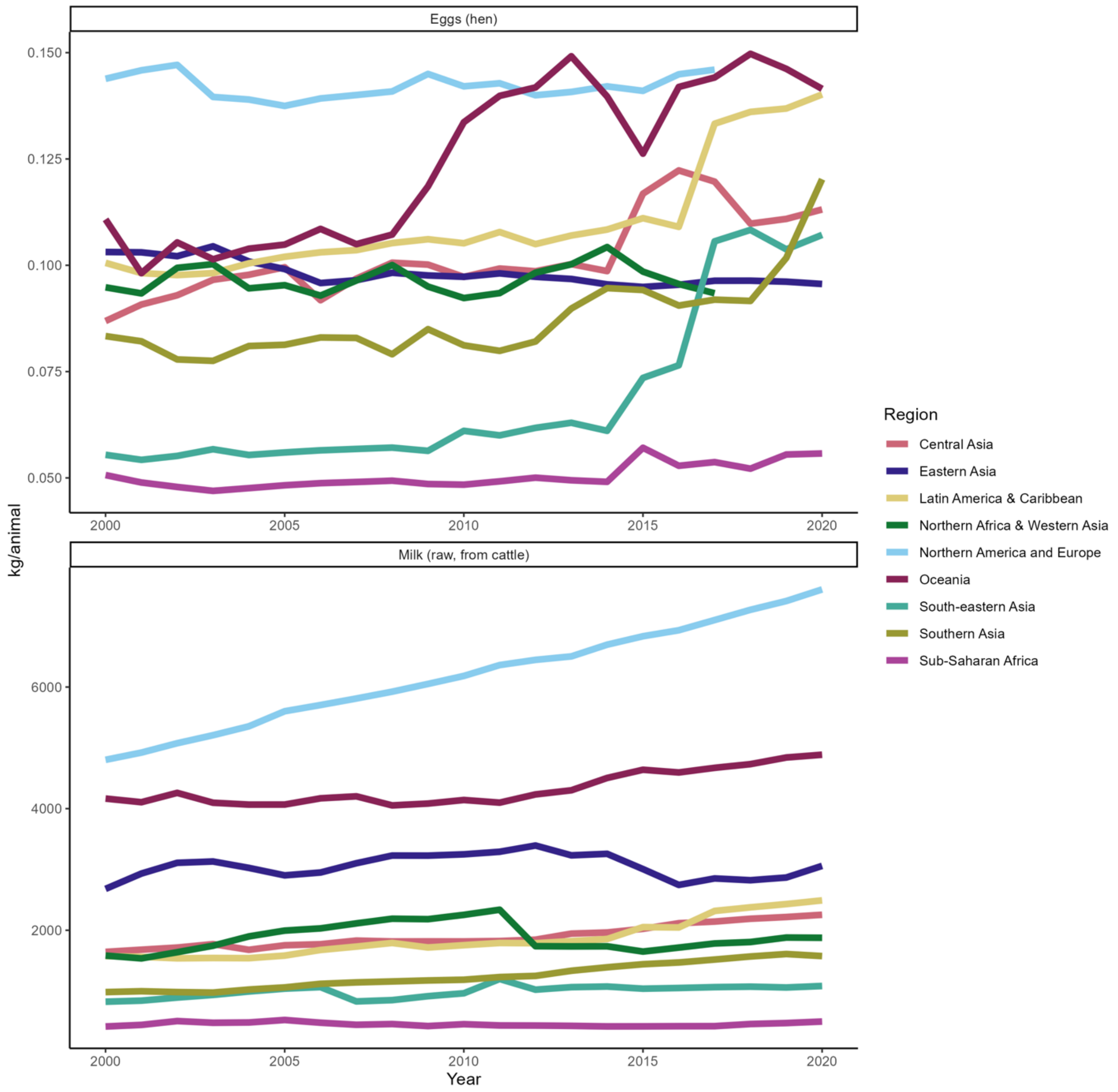

Producing animals-weighted mean by region.

1.2.9 Yield, Pulses & Nuts, 2000-2020, by region

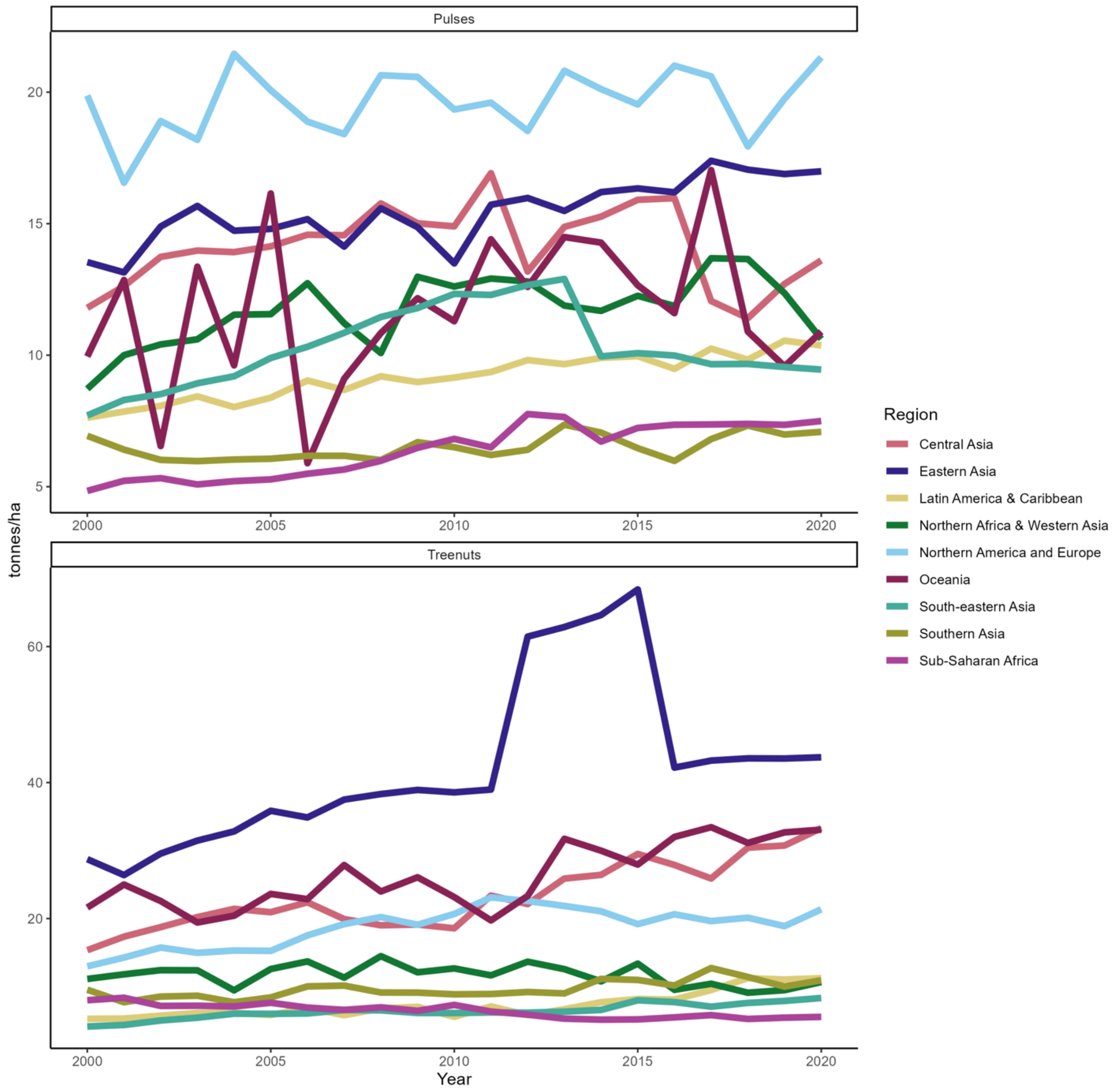

Area harvested-weighted mean by region.

1.2.10 Yield, Fruits & Vegetables, 2000-2020, by region

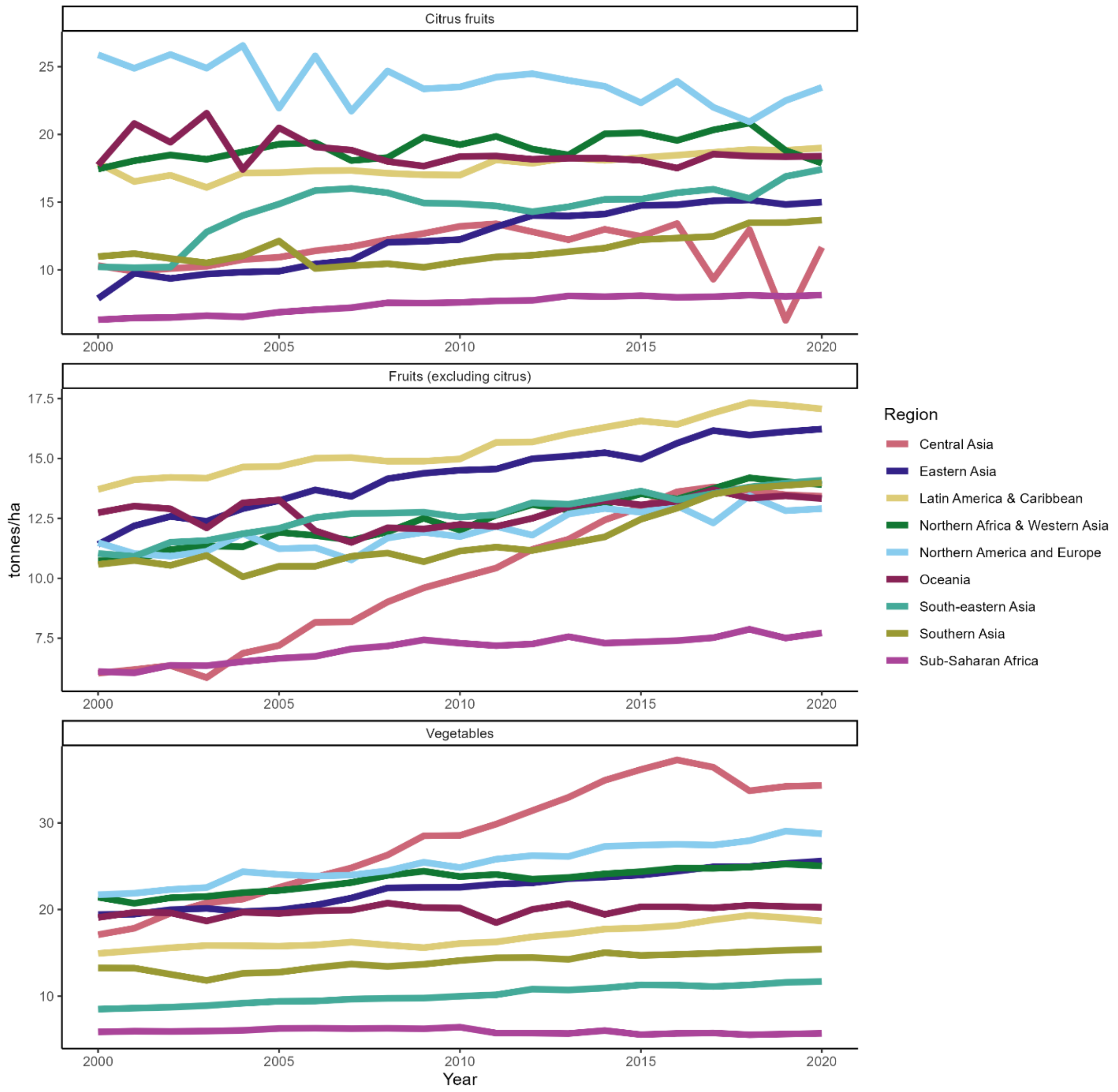

Area harvested-weighted mean by region.

1.2.11 Cropland expansion, 2020 (% change over prior 5 years)

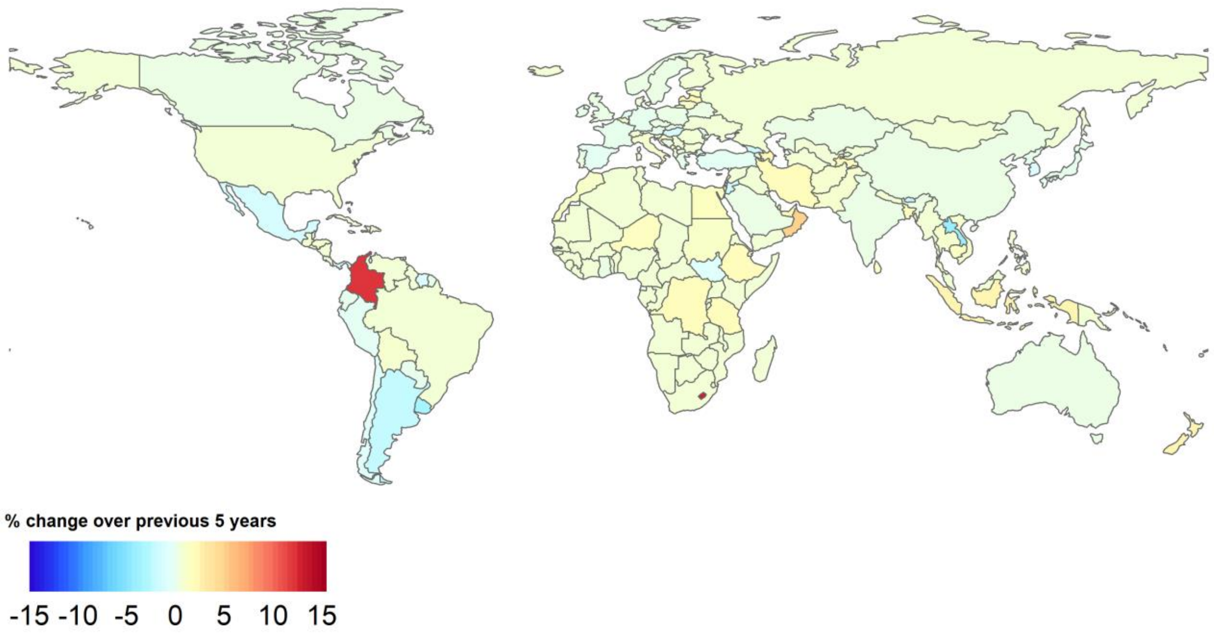

Basemap obtained using the R package ‘rnaturalearth’ which accesses Natural Earth v4.1.0 (March 2018) maps which are wholly in the public domain.

1.2.12 Agriculture water withdrawal as % of total renewable water resources, 2018

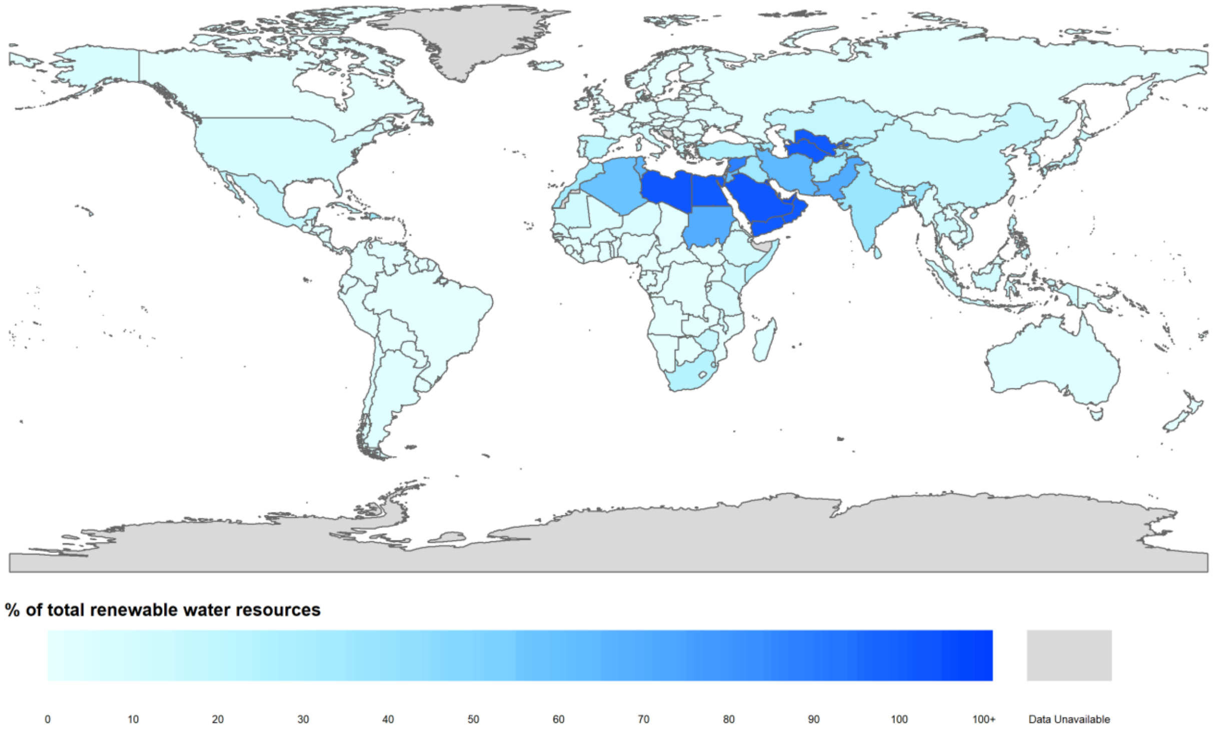

Basemap obtained using the R package ‘rnaturalearth’ which accesses Natural Earth v4.1.0 (March 2018) maps which are wholly in the public domain.

1.2.13 Agriculture water withdrawal as % of total renewable water resources, by region

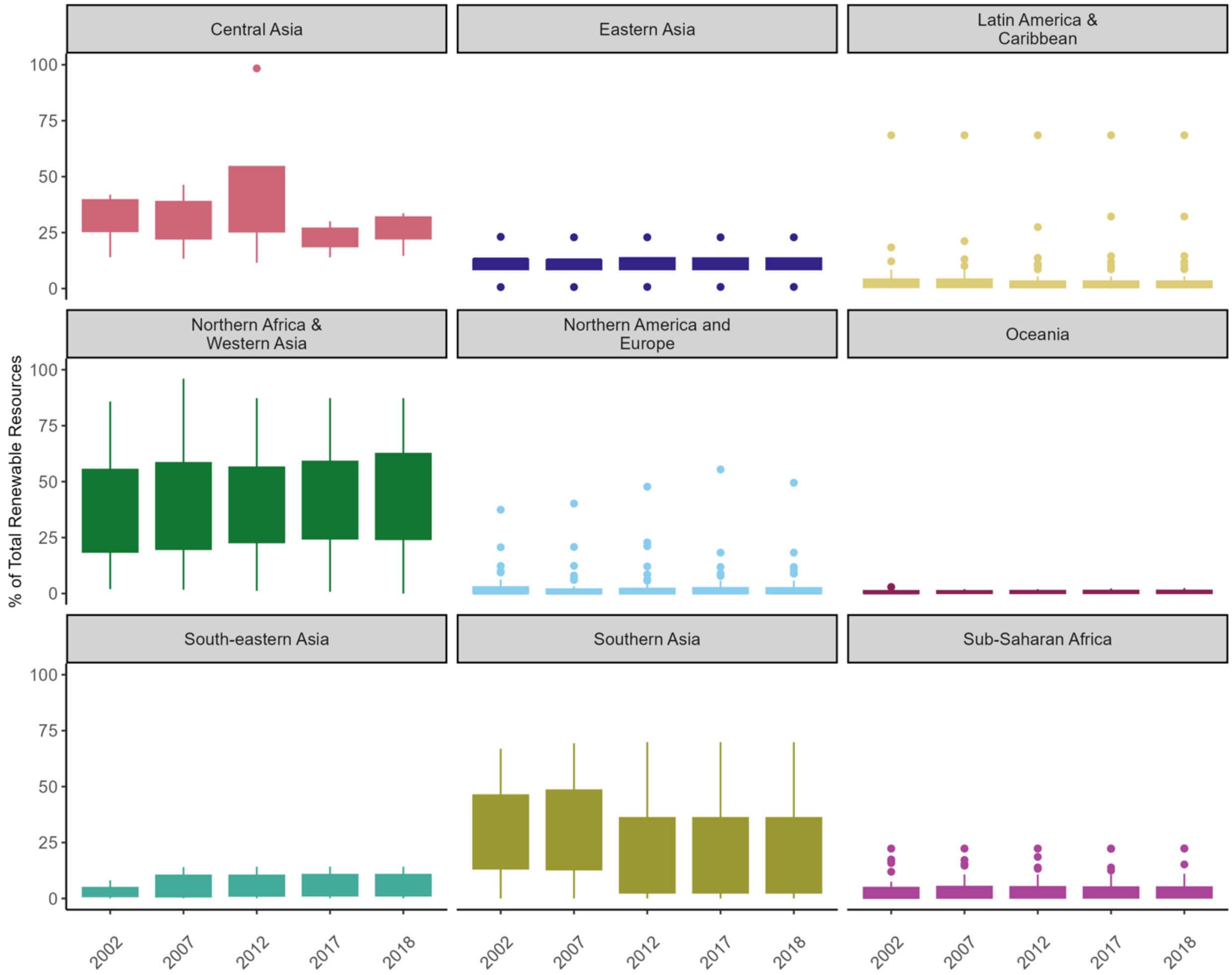

Boxplot illustrates a box of the 25th to 75th percentiles whiskers (vertical lines) extend the length of 1.5 times the interquartile range. All outliers beyond 1.5 times the interquartile range are shown individually. Sample size by region and year provided in the following table:

Region	2002	2007	2012	2017	2018
Central Asia	5	5	5	5	5
Eastern Asia	5	5	5	5	5
Latin America & Caribbean	28	29	32	32	32
Northern Africa & Western Asia	22	22	23	23	23
Northern America and Europe	38	39	39	39	39
Oceania	4	4	4	4	4

Region	2002	2007	2012	2017	2018
South-eastern Asia	6	11	11	11	11
Southern Asia	7	7	9	9	9
Sub-Saharan Africa	45	45	46	47	47

1.2.14 Agriculture water withdrawal as % of total renewable water resources, by income group

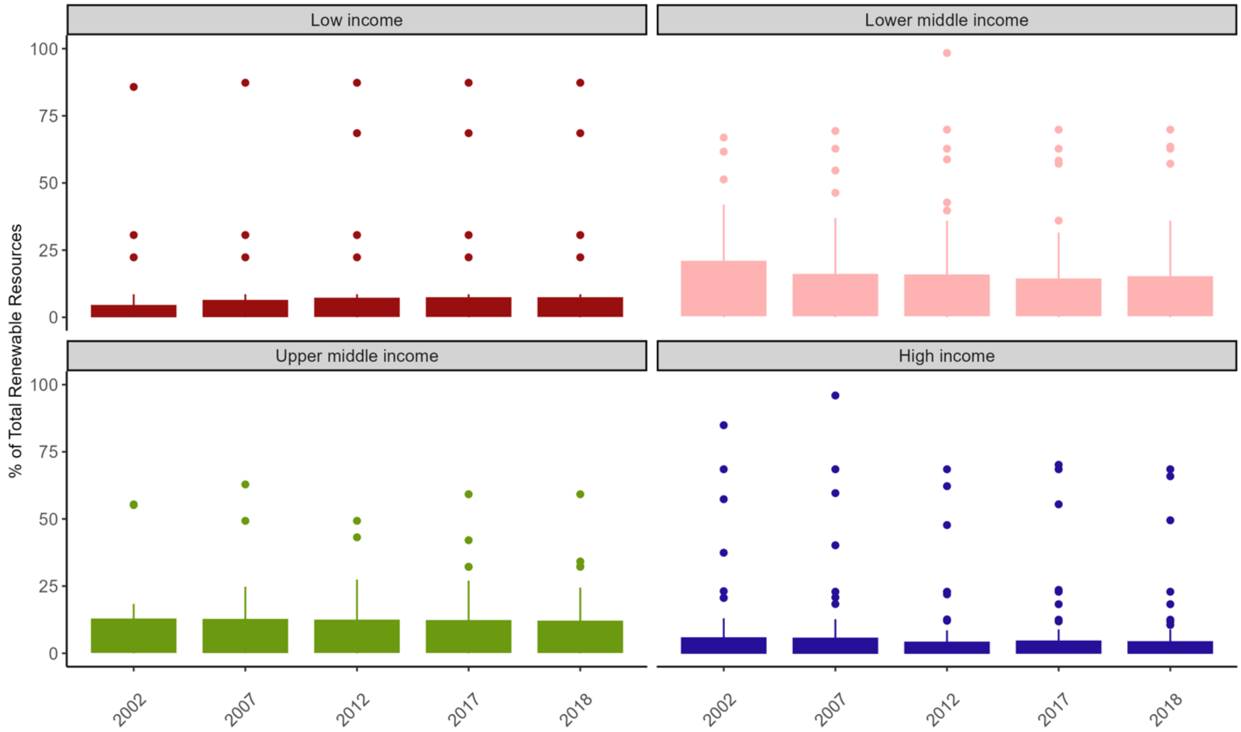

Boxplot illustrates a box of the 25th to 75th percentiles with whiskers (vertical lines) extend the length of 1.5 times the interquartile range. All outliers beyond 1.5 times the interquartile range are shown individually. Sample size by income group and year provided in the following table:

Income group	2002	2007	2012	2017	2018
Low income	26	26	28	28	28
Lower middle income	41	45	47	48	48
Upper middle income	41	44	46	46	46
High income	51	51	52	52	52

1.2.15 Functional integrity: Percent agricultural land with minimum level of natural habitat, 2015

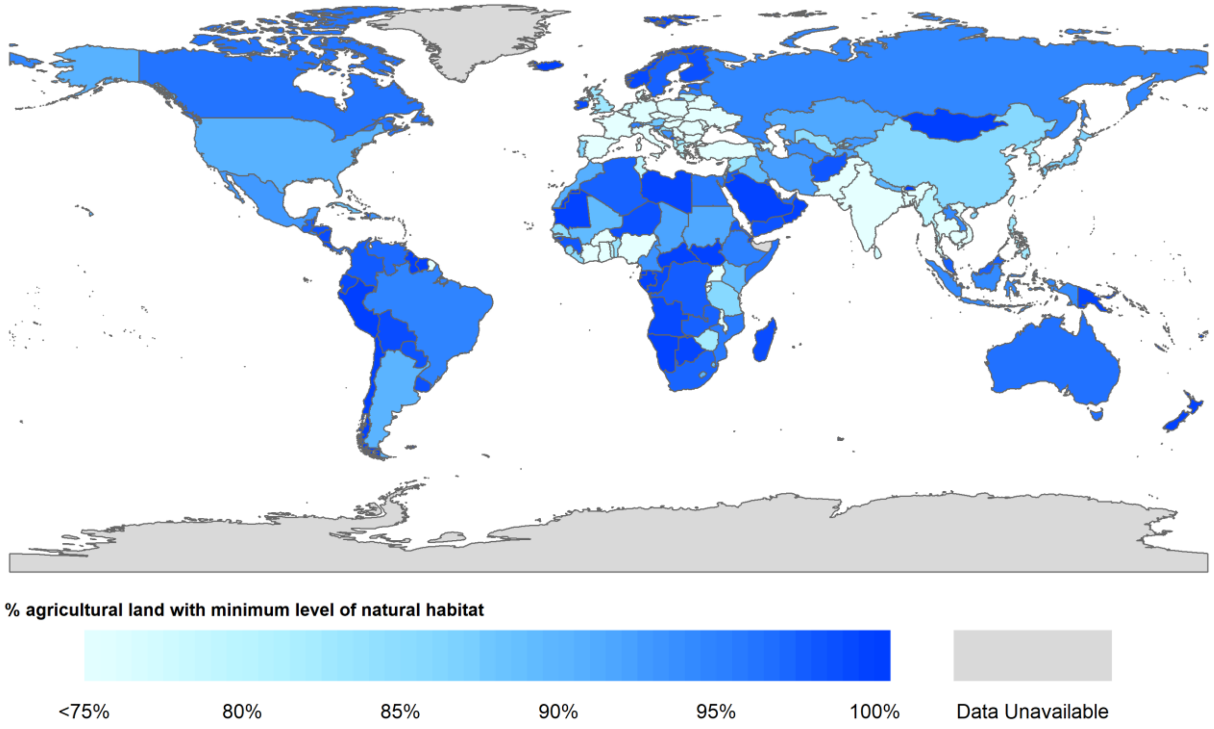

Basemap obtained using the R package 'rnaturalearth' which accesses Natural Earth v4.1.0 (March 2018) maps which are wholly in the public domain.

1.2.16 Fishery health index progress score, 2021

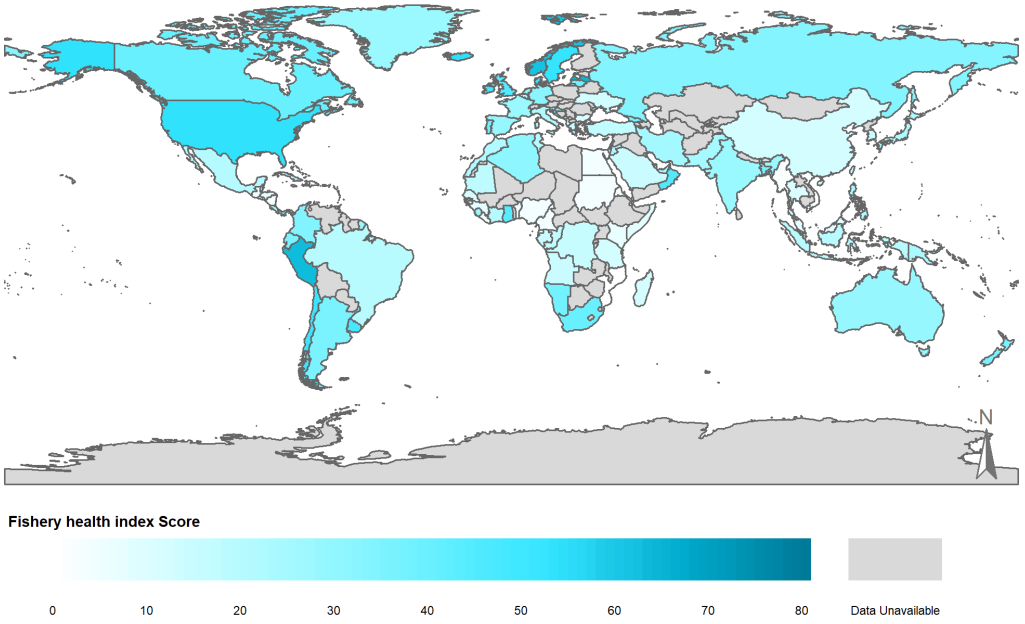

Basemap obtained using the R package 'rnaturalearth' which accesses Natural Earth v4.1.0 (March 2018) maps which are wholly in the public domain.

1.2.17 Total pesticides per unit of land (kg/ha), 2000-2019, by region

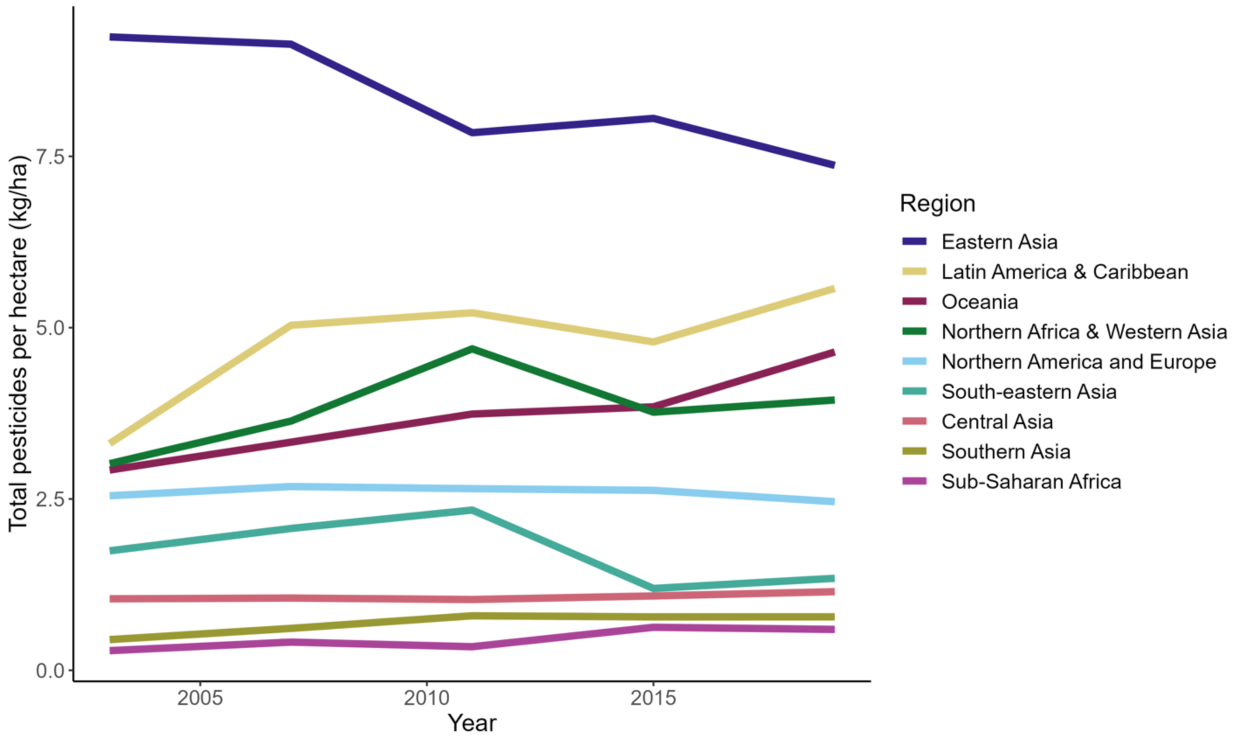

Cropland-weighted mean by region.

1.2.18 Total pesticides per unit of land (kg/ha), 2019

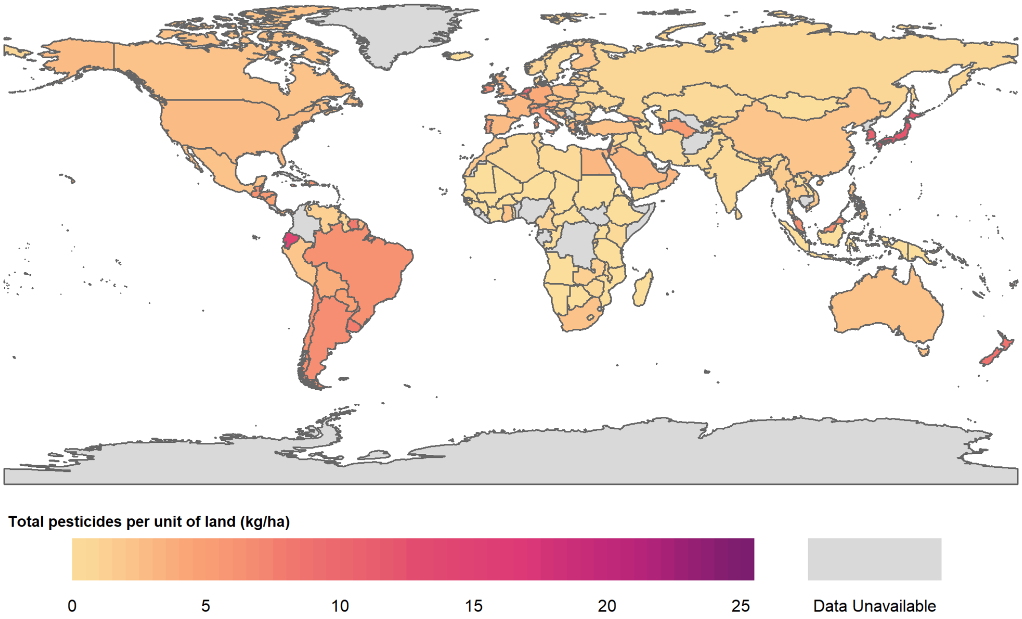

Basemap obtained using the R package ‘rnaturalearth’ which accesses Natural Earth v4.1.0 (March 2018) maps which are wholly in the public domain.

1.2.19 Sustainable nitrogen management index, 2000-2018, by region

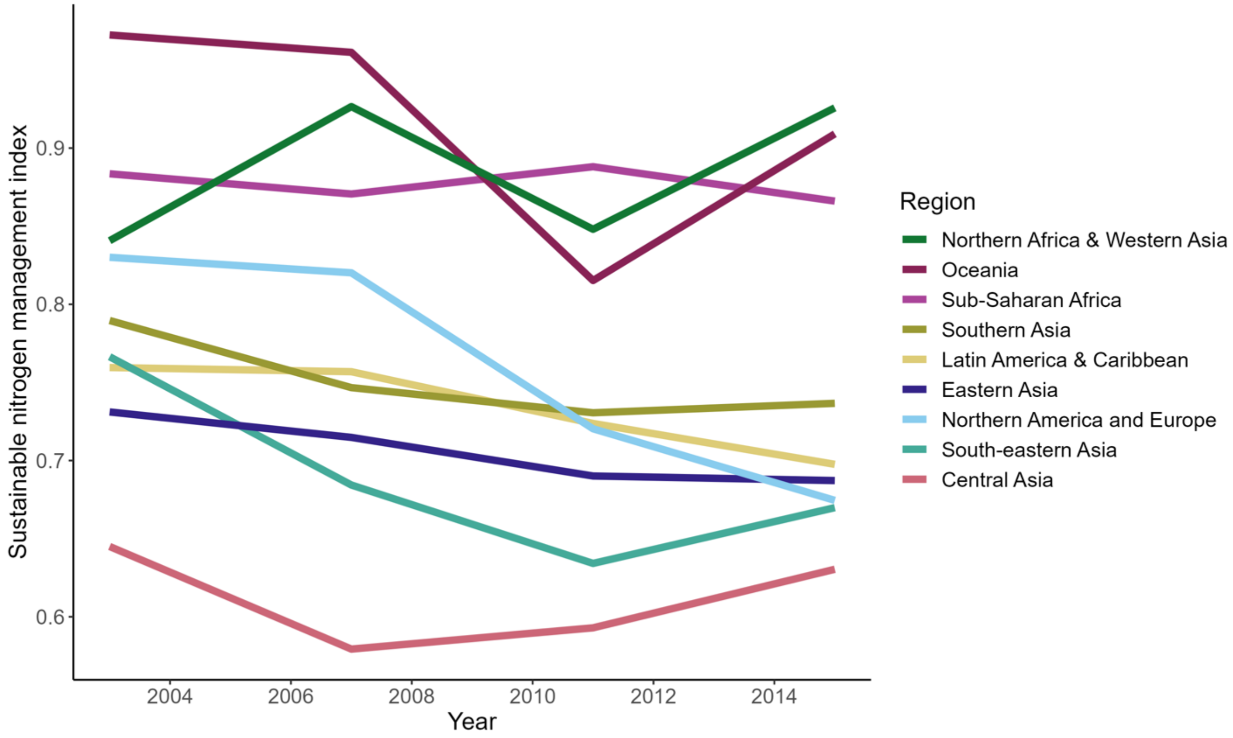

Cropland-weighted mean by region.

1.2.20 Sustainable nitrogen management index, 2018

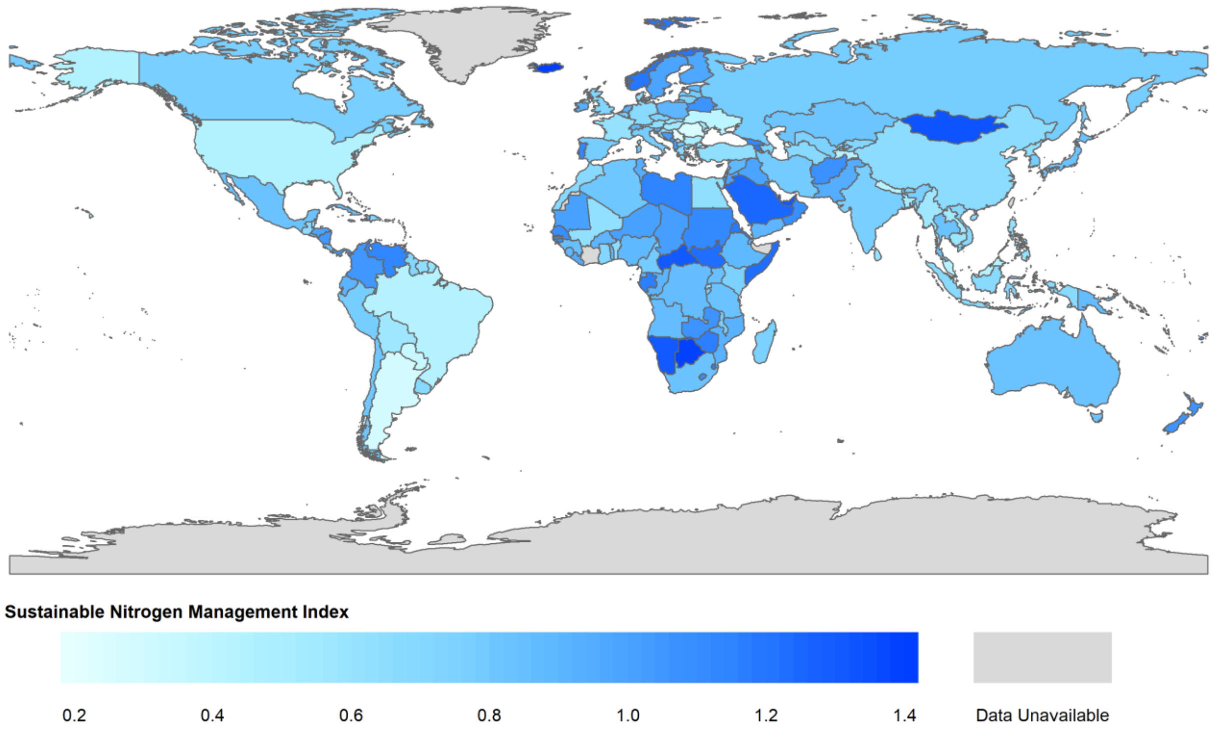

Basemap obtained using the R package ‘rnaturalearth’ which accesses Natural Earth v4.1.0 (March 2018) maps which are wholly in the public domain.

1.3.1 Share of agriculture in GDP, 2019

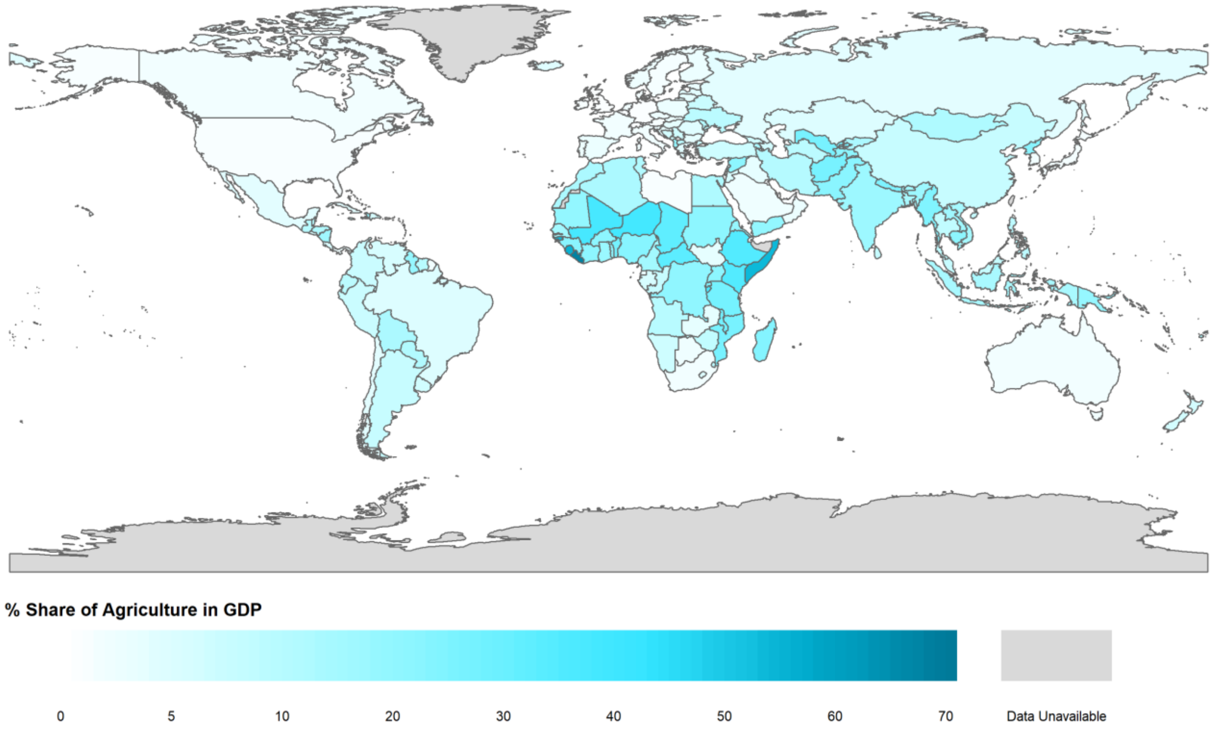

Basemap obtained using the R package 'rnaturalearth' which accesses Natural Earth v4.1.0 (March 2018) maps which are wholly in the public domain.

1.3.2 Share of agriculture in GDP, 2000-2019, by region

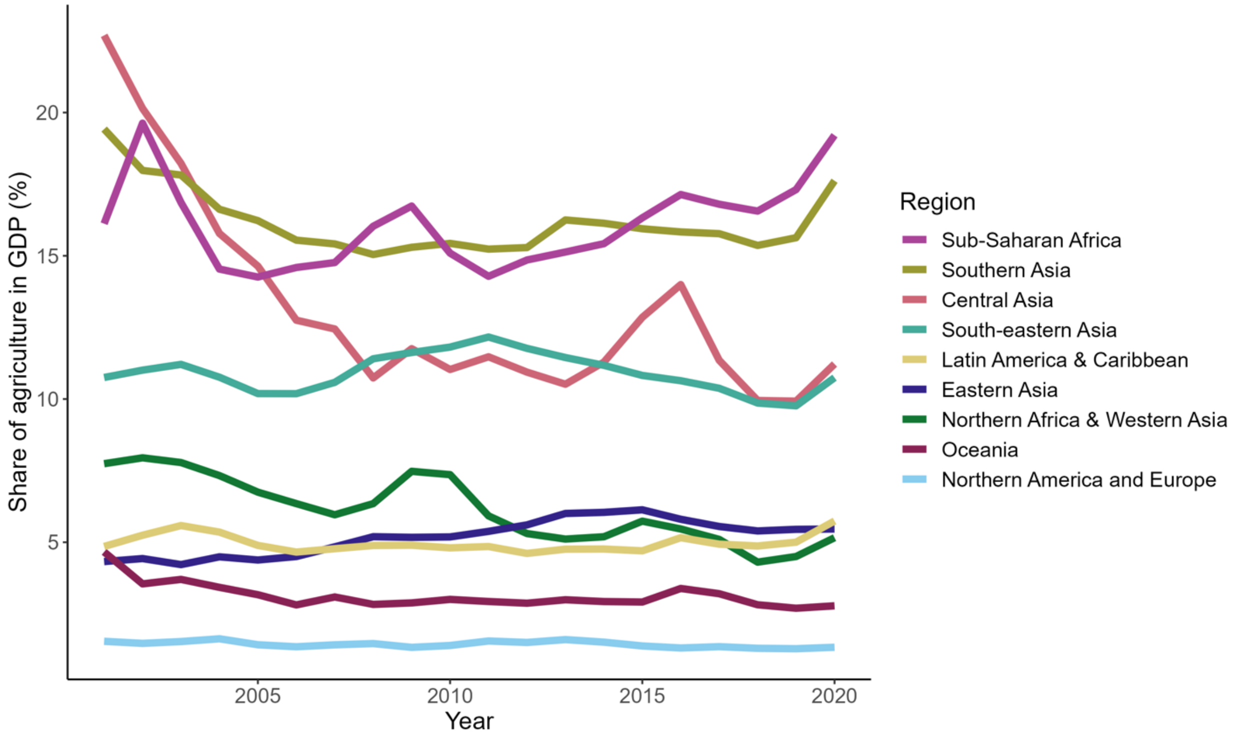

GDP-weighted regional means.

1.3.3 Unemployment and Underemployment rates by sex, age, and urban/rural, 2020

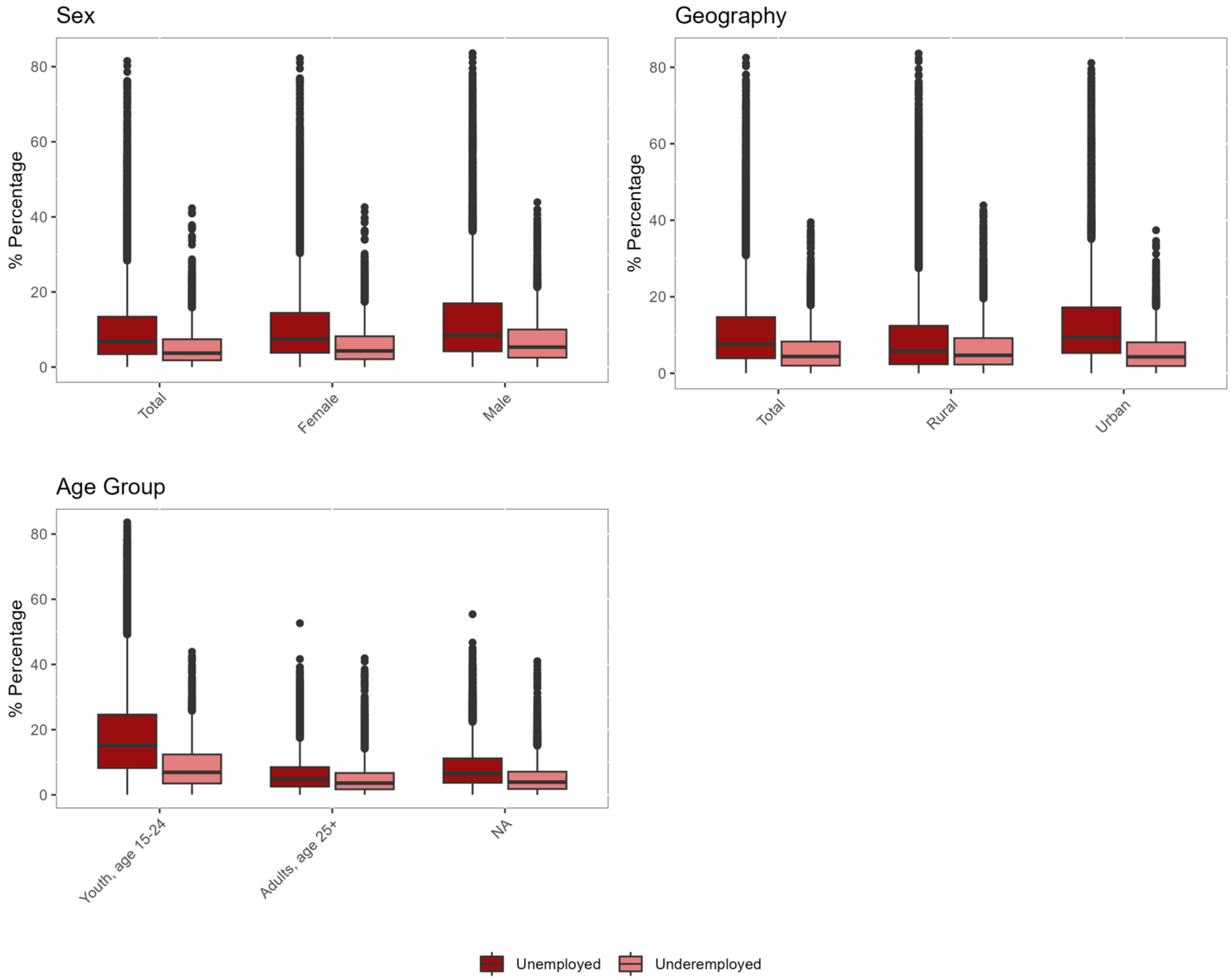

Sample size: 185 countries with disaggregated data, NA shown where disaggregation is not reported. Boxplot illustrates a box of the 25th to 75th percentiles with the horizontal line at the median, whiskers (vertical lines) extend the length of 1.5 times the interquartile range. All outliers beyond 1.5 times the interquartile range are shown individually.

1.3.4 Unemployment rate (total), 2010-2020, by region

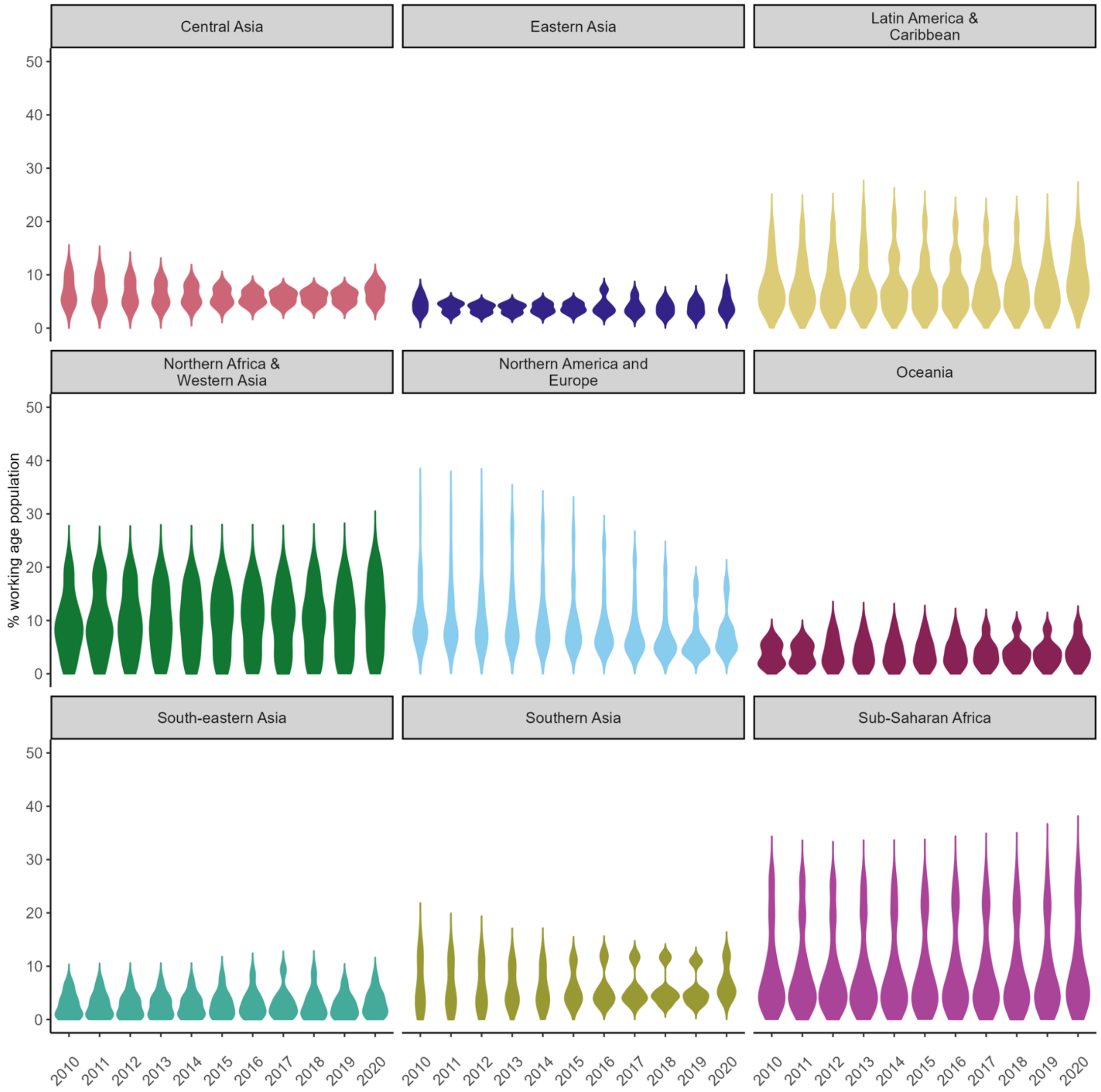

Sample size by region and year provided in the following table:

Region	2010	2011	2012	2013	2014	2015	2016	2017	2018	2019	2020
Central Asia	5	5	5	5	5	5	5	5	5	5	5
Eastern Asia	5	5	5	5	5	5	5	5	5	5	5
Latin America & Caribbean	29	29	29	29	29	29	29	29	29	29	29
Northern Africa & Western Asia	23	23	23	23	23	23	23	23	23	23	23

Region	2010	2011	2012	2013	2014	2015	2016	2017	2018	2019	2020
Northern America and Europe	41	41	41	41	41	41	41	41	41	41	41
Oceania	8	8	8	8	8	8	8	8	8	8	8
South-eastern Asia	10	10	10	10	10	10	10	10	10	10	10
Southern Asia	9	9	9	9	9	9	9	9	9	9	9
Sub-Saharan Africa	47	47	47	47	47	47	47	47	47	47	47

1.3.5 Underemployment rate (total), 2010-2020, by region

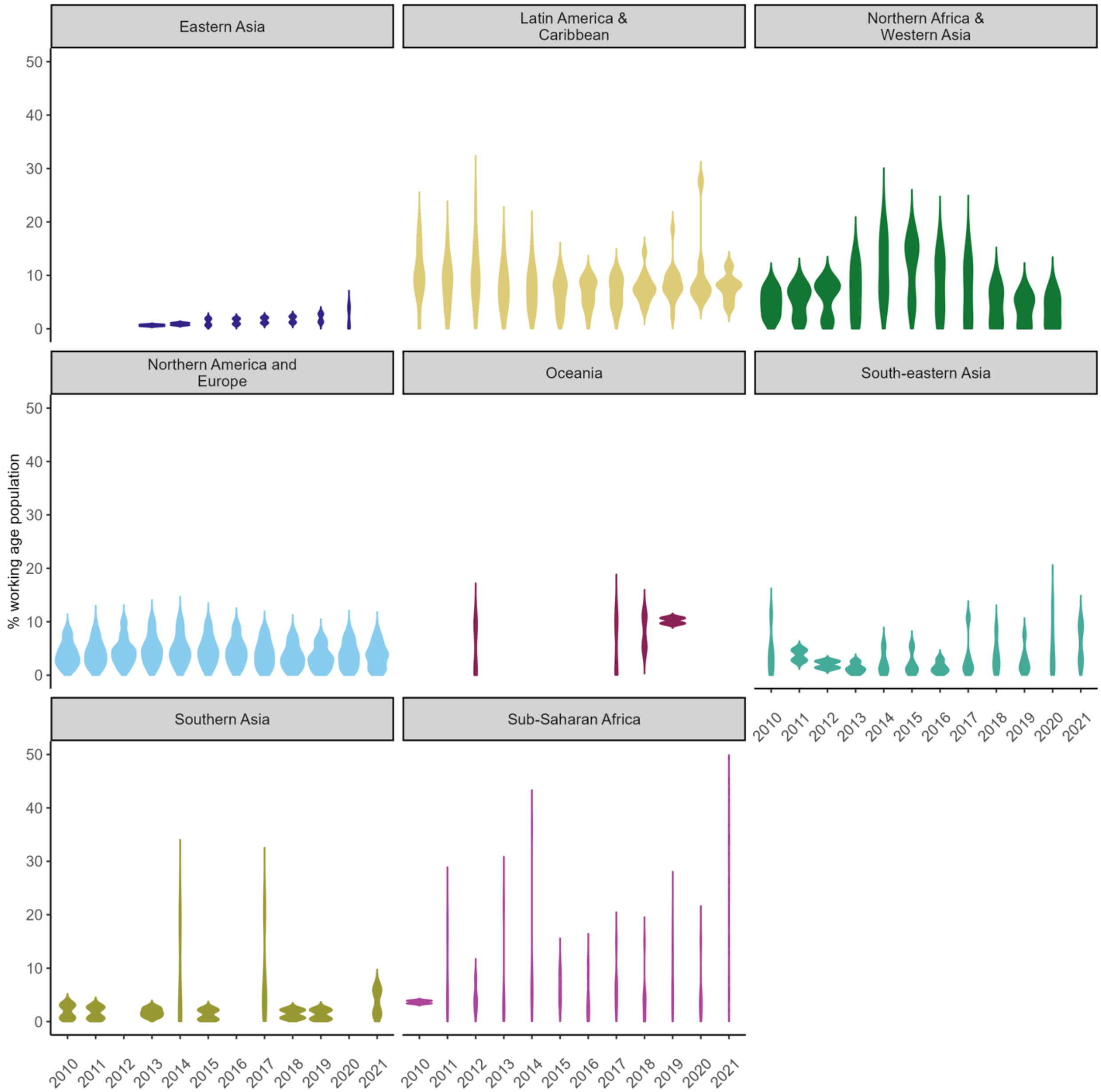

Sample size by region and year provided in the following table:

Region	2010	2011	2012	2013	2014	2015	2016	2017	2018	2019	2020	2021
Eastern Asia	1	1	1	2	2	2	2	2	2	2	2	1
Latin America & Caribbean	11	12	14	14	14	14	14	14	14	15	8	6
Northern Africa & Western Asia	3	3	3	3	4	3	4	4	4	5	5	1

Region	2010	2011	2012	2013	2014	2015	2016	2017	2018	2019	2020	2021
Northern America and Europe	32	32	32	32	33	33	33	34	35	35	33	32
Oceania	1	1	2	1	1	1	1	2	2	2	1	NA
South-eastern Asia	5	2	2	4	4	4	4	5	5	6	5	2
Southern Asia	2	2	NA	3	3	2	1	4	2	2	1	2
Sub-Saharan Africa	2	3	4	4	7	4	4	10	6	10	4	2

1.3.6 Unemployment rate (total), 2010-2020, by income group

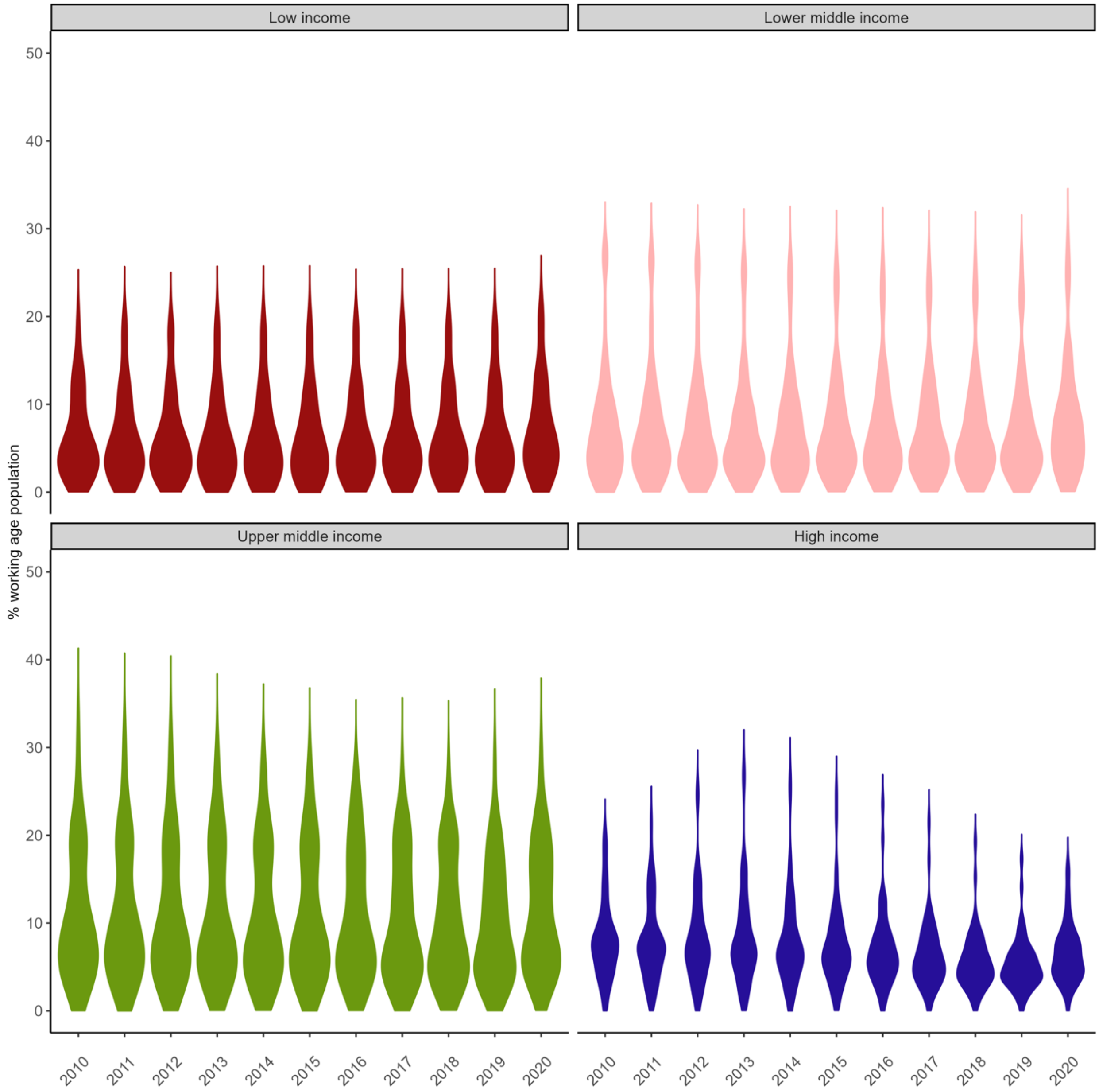

Sample size by income group and year provided in the following table:

Income group	2010	2011	2012	2013	2014	2015	2016	2017	2018	2019	2020
Low income	28	28	28	28	28	28	28	28	28	28	28
Lower middle income	51	51	51	51	51	51	51	51	51	51	51
Upper middle income	47	47	47	47	47	47	47	47	47	47	47

Income group	2010	2011	2012	2013	2014	2015	2016	2017	2018	2019	2020
High income	50	50	50	50	50	50	50	50	50	50	50

1.3.7 Underemployment rate (total), 2010-2020, by income group

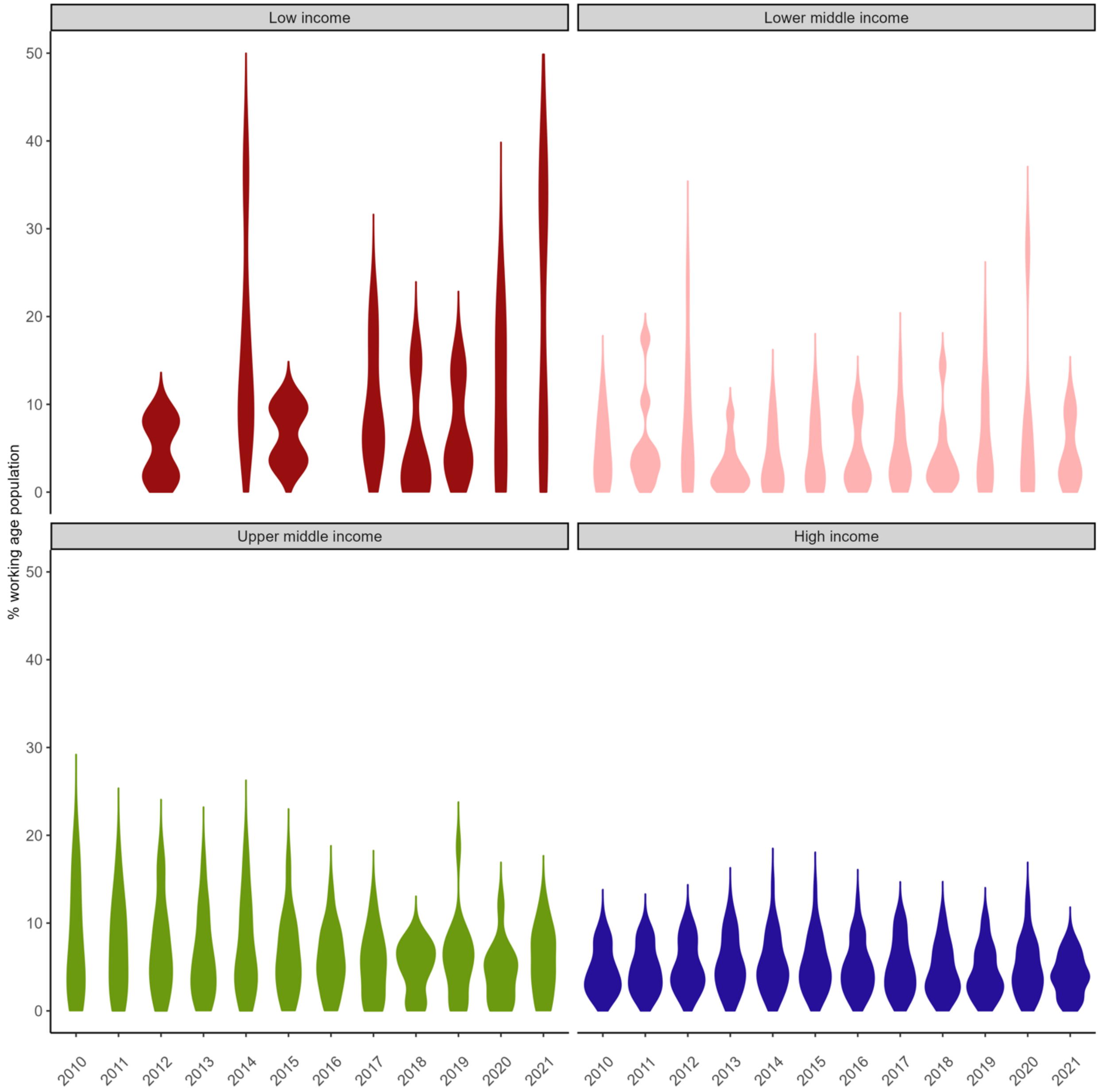

Sample size by income group and year provided in the following table:

Income group	2010	2011	2012	2013	2014	2015	2016	2017	2018	2019	2020	2021
Low income	1	1	2	1	5	2	NA	6	4	3	4	2
Lower middle income	9	9	9	11	10	10	12	16	10	18	7	3
Upper middle income	13	12	14	16	16	15	15	16	19	19	13	9
High income	34	34	33	35	37	36	36	37	37	37	35	32

1.3.8 Unemployment and underemployment (total), 2005-2020, by region

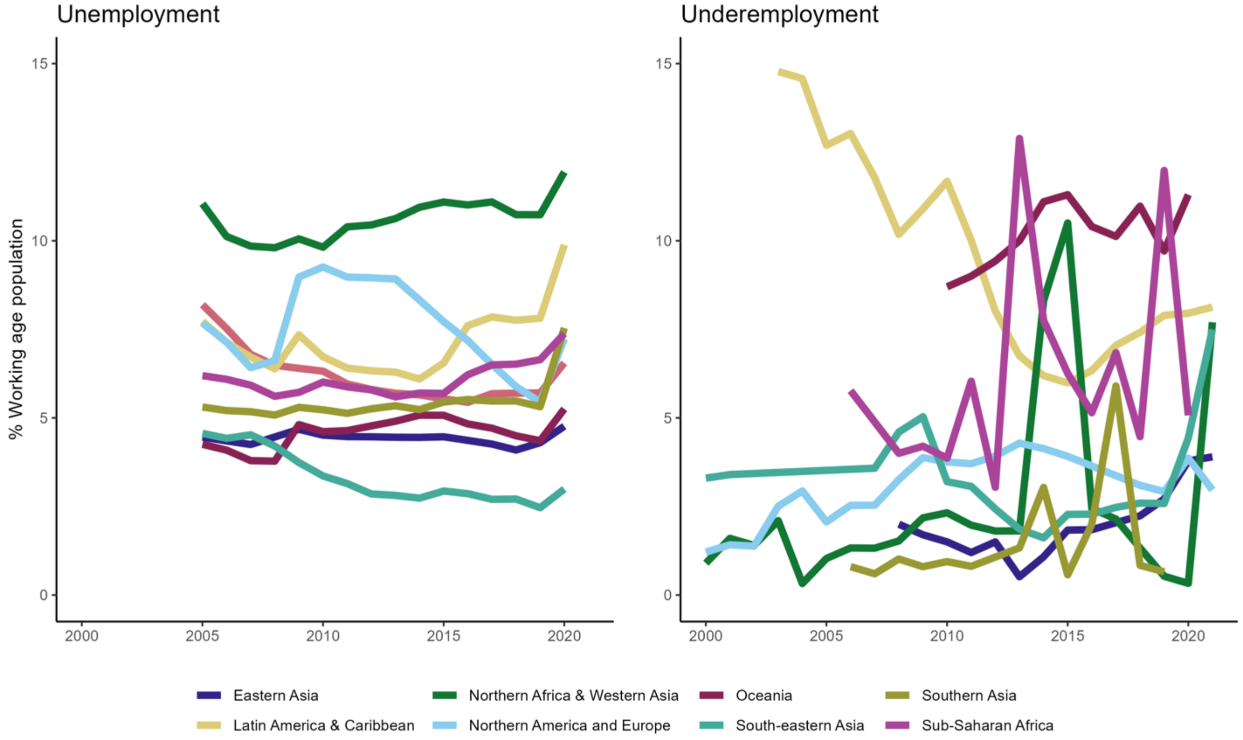

Population-weighted regional mean.

1.3.9 Unemployment and underemployment (total), 2005-2020, by income group

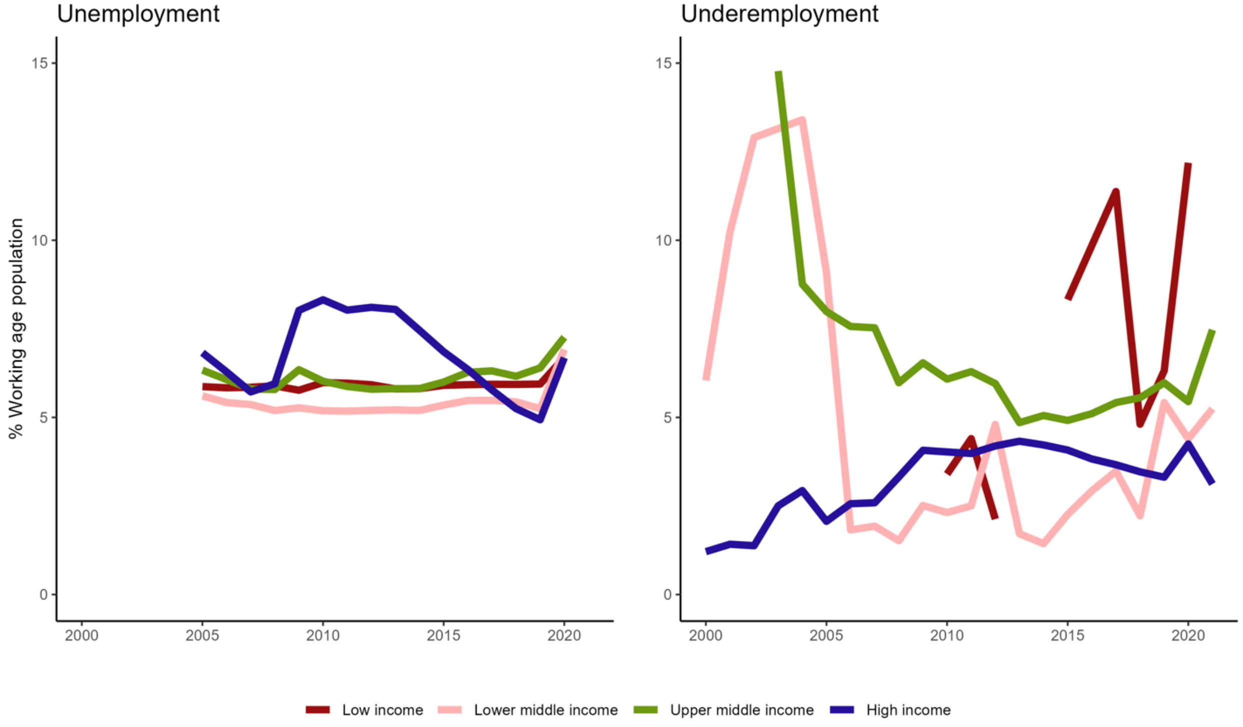

Population-weighted regional mean.

1.3.10 Social protection coverage and adequacy

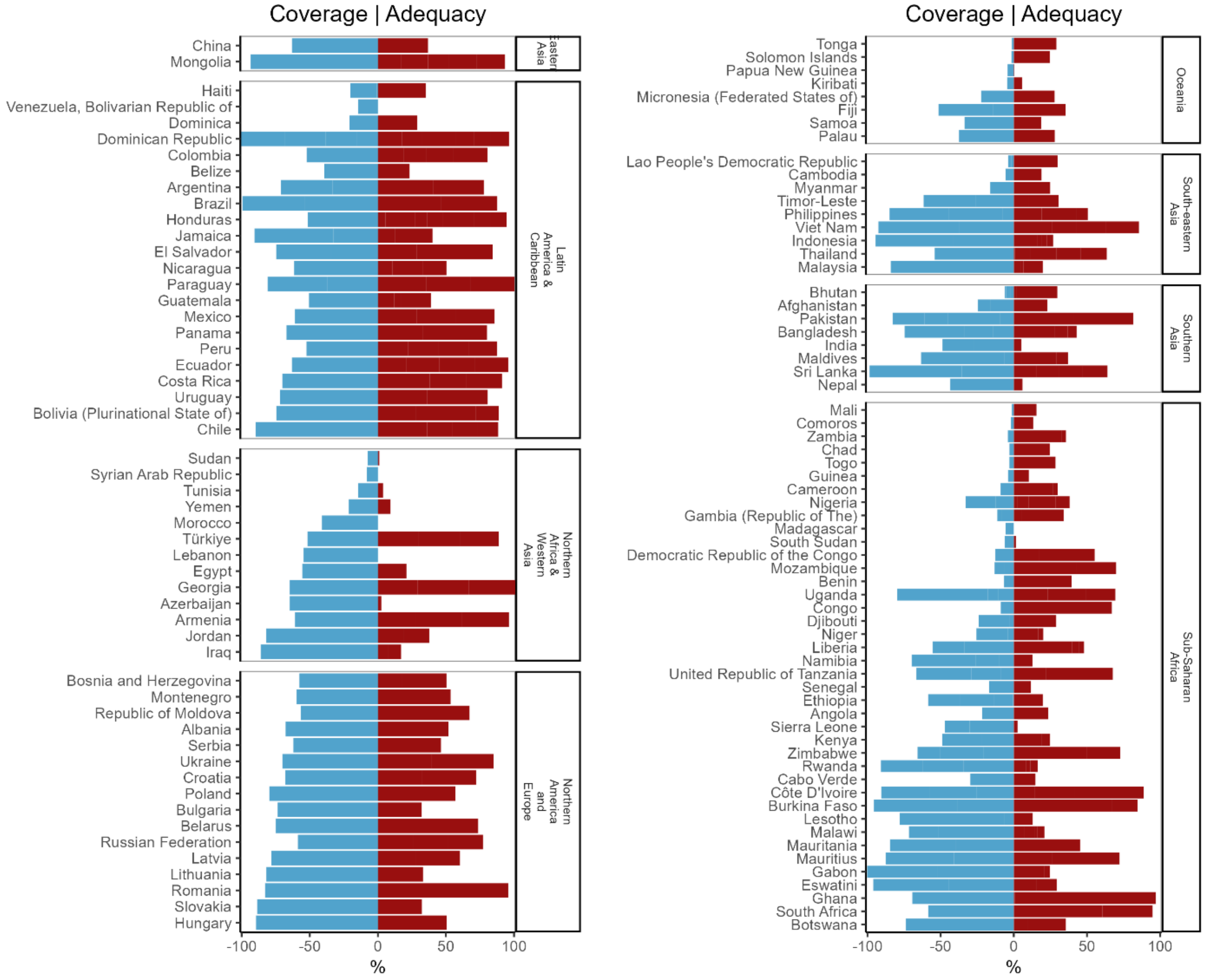

Data year differs by country. See table A1.2. The x-axis for coverage reflects the share of individuals in the total population from households where at least one member participates in a social protection and labor market program. The x-axis for adequacy reflects the total social protection benefit amount received by beneficiary households (direct and indirect beneficiaries) as a percentage of beneficiaries' post-transfer, household wealth.

1.3.11 Percent children 5-17 engaged in child labor, by sex, by region

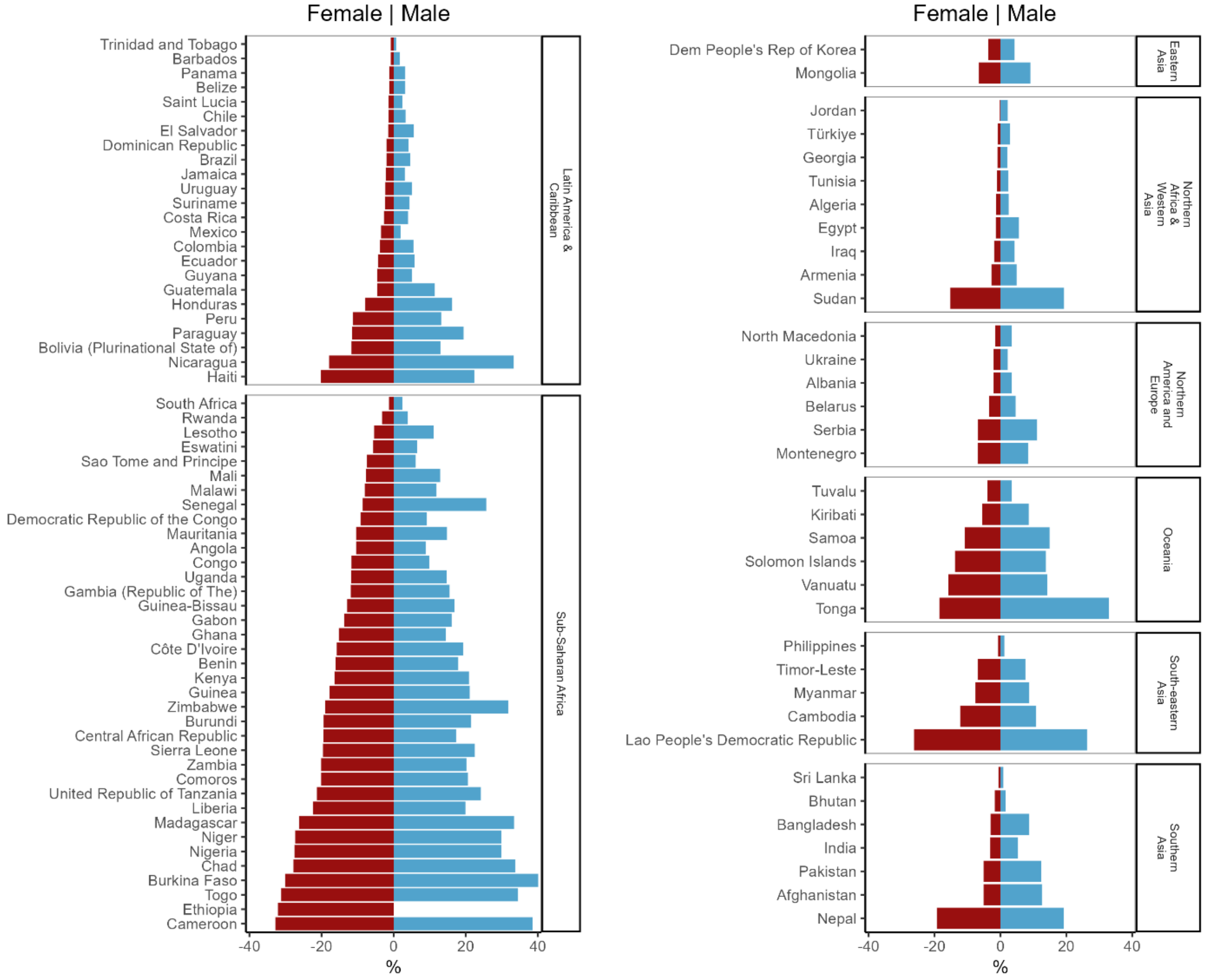

Unweighted regional mean. Data source year differs by country. See table A1.2.

1.3.12 Percent children 5-17 engaged in child labor, by sex, by income group

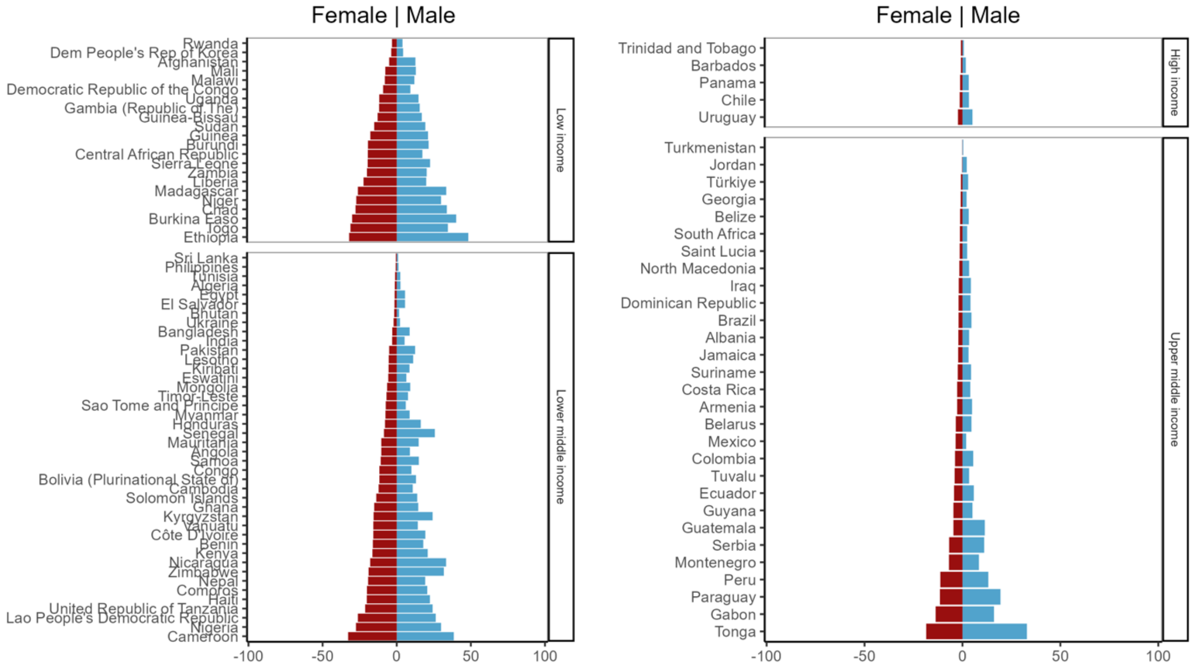

Unweighted income group mean. Data source year differs by country. See table A1.2.

1.3.13 Distribution of landholdings by sex

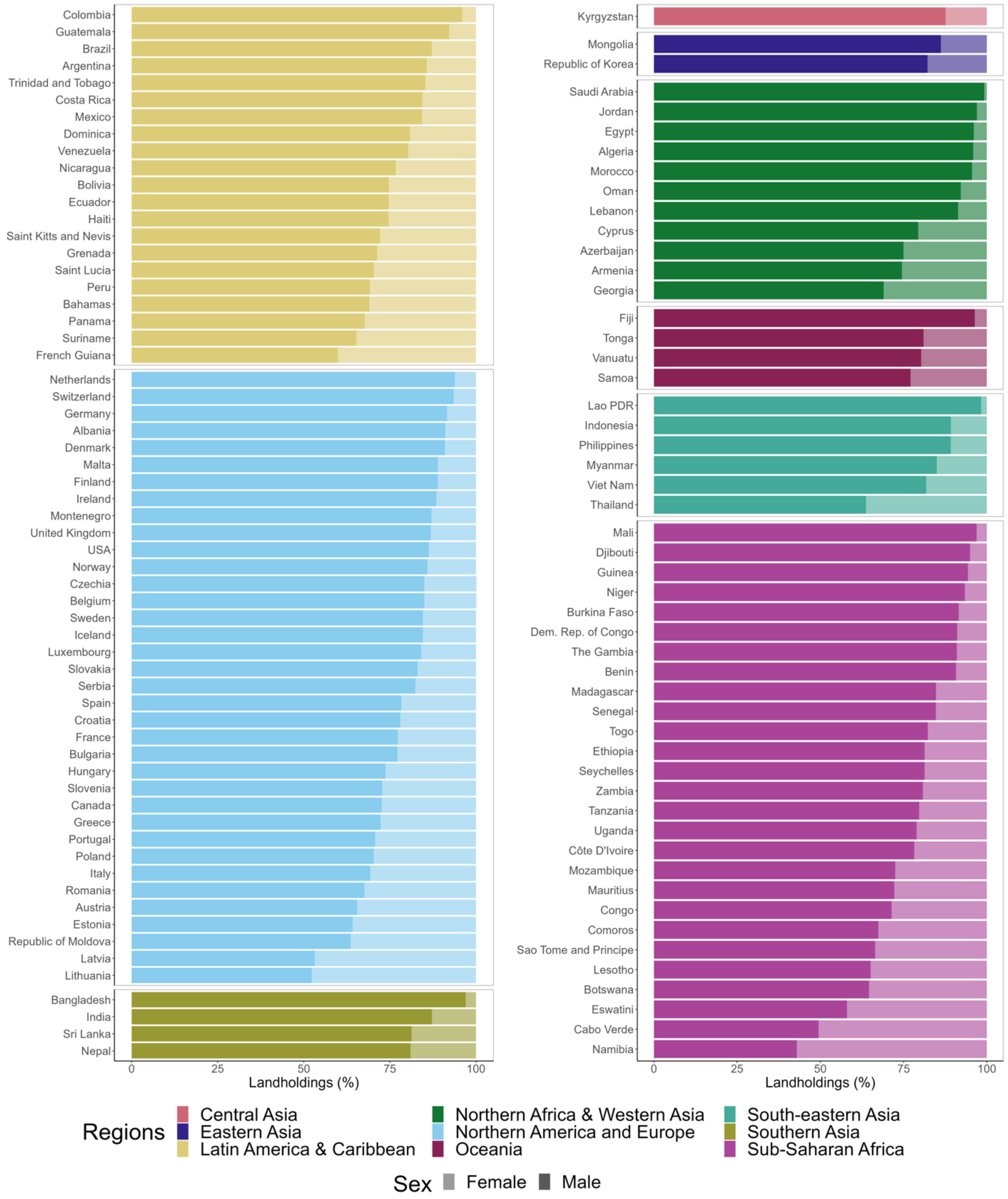

Data source year differs by country. See table A1.2.

1.4.1 Civil society participation index, 2021

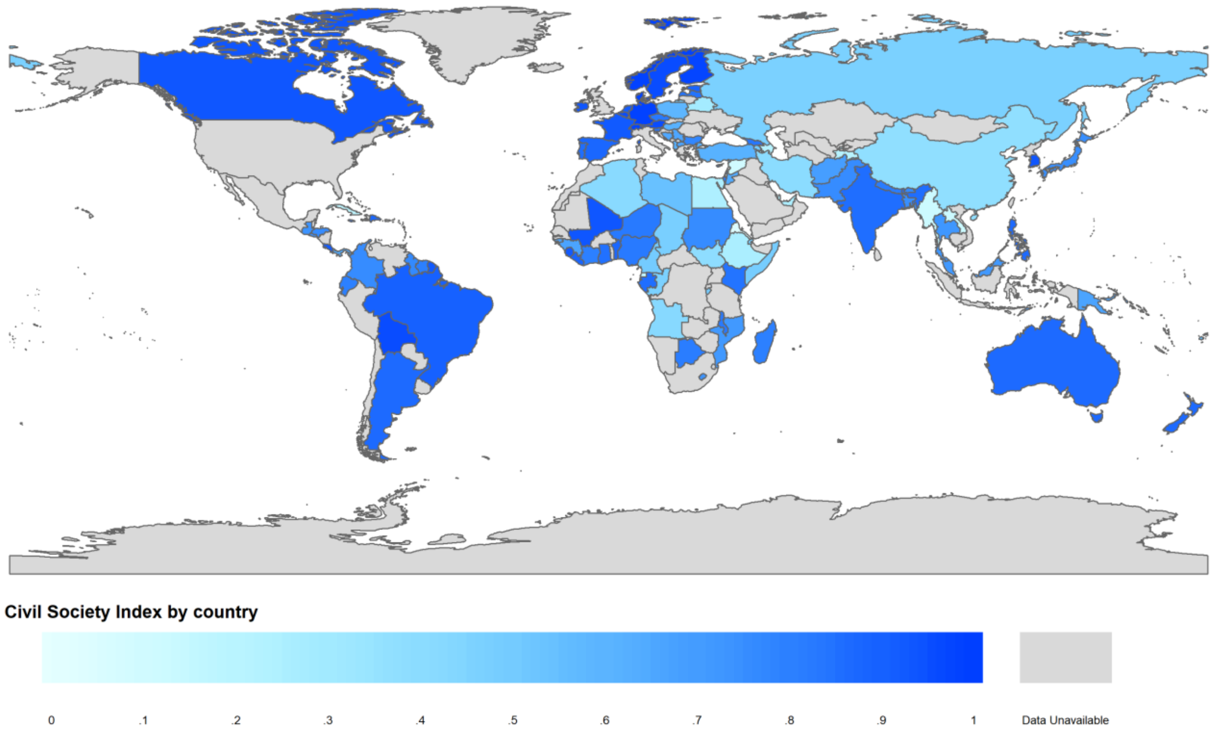

Basemap obtained using the R package 'rnaturalearth' which accesses Natural Earth v4.1.0 (March 2018) maps which are wholly in the public domain.

1.4.2 Civil society participation index, 2000-2021, by region

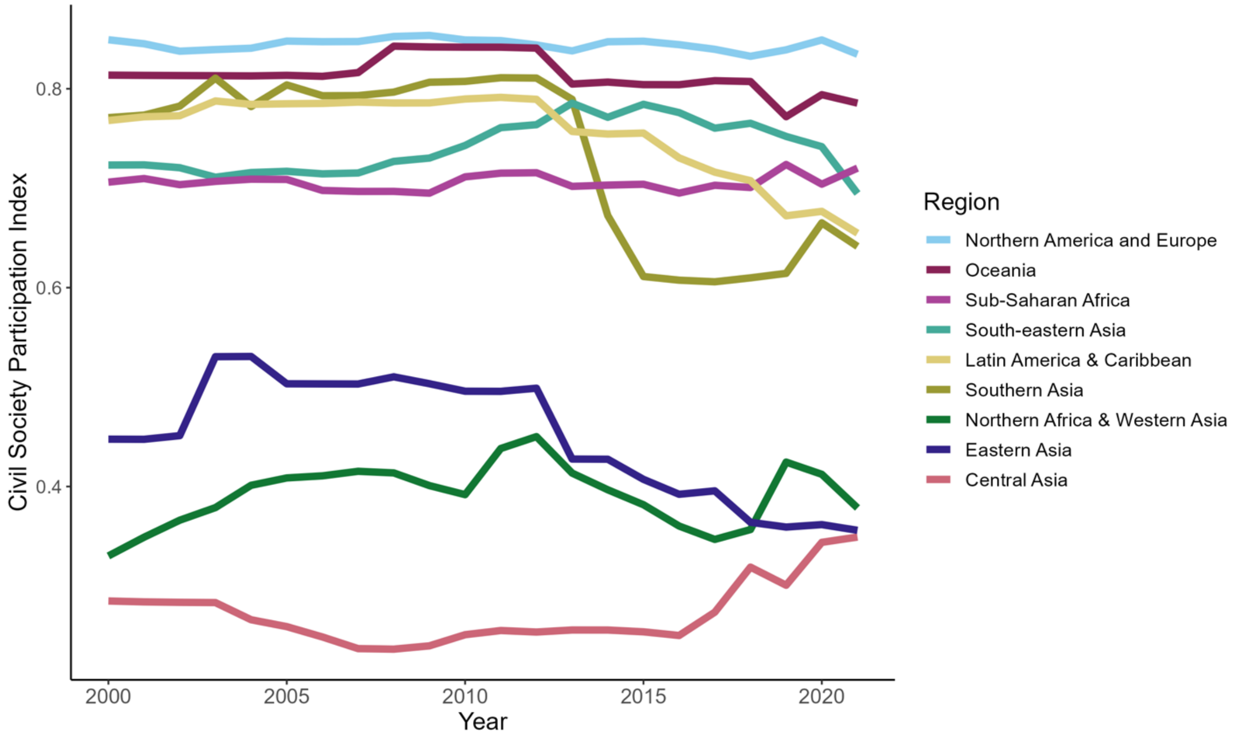

Population-weighted regional means.

1.4.3 Civil society participation index, 2000-2021, by income group

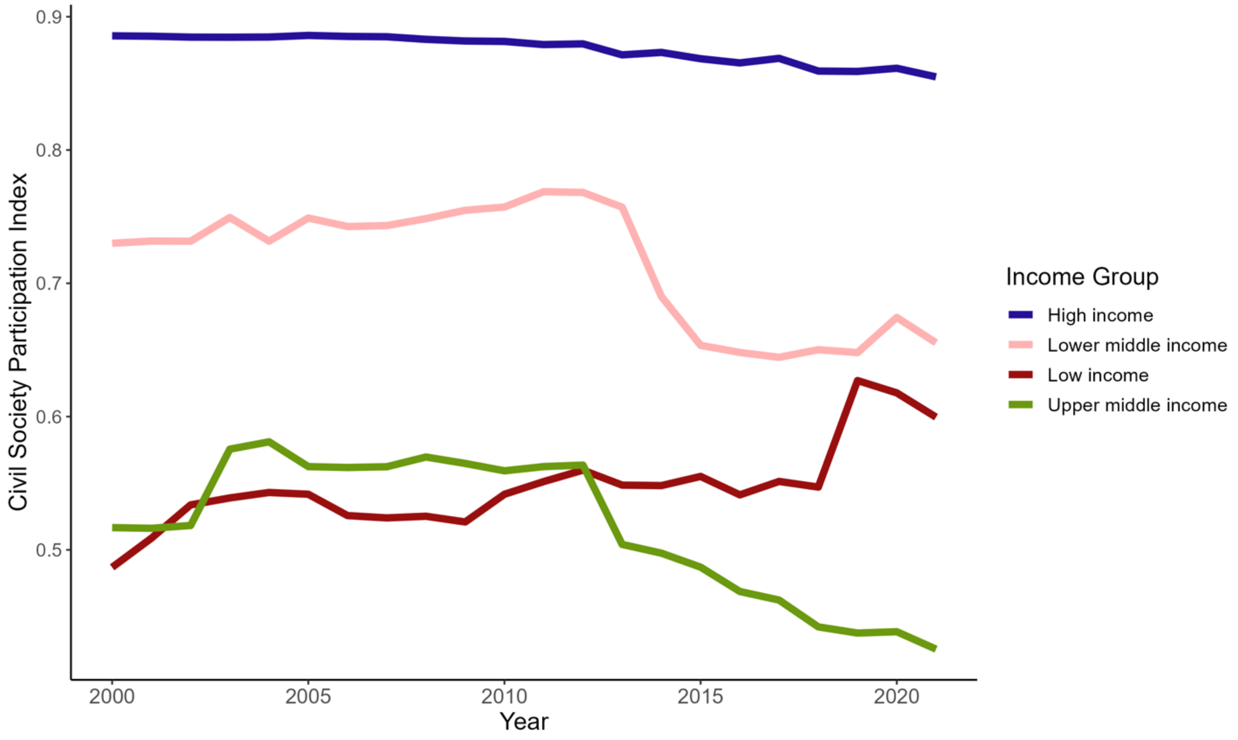

Population-weighted income group means.

1.4.4 Percent urban population living in cities signed onto the Milan Urban Food Policy Pact, 2020

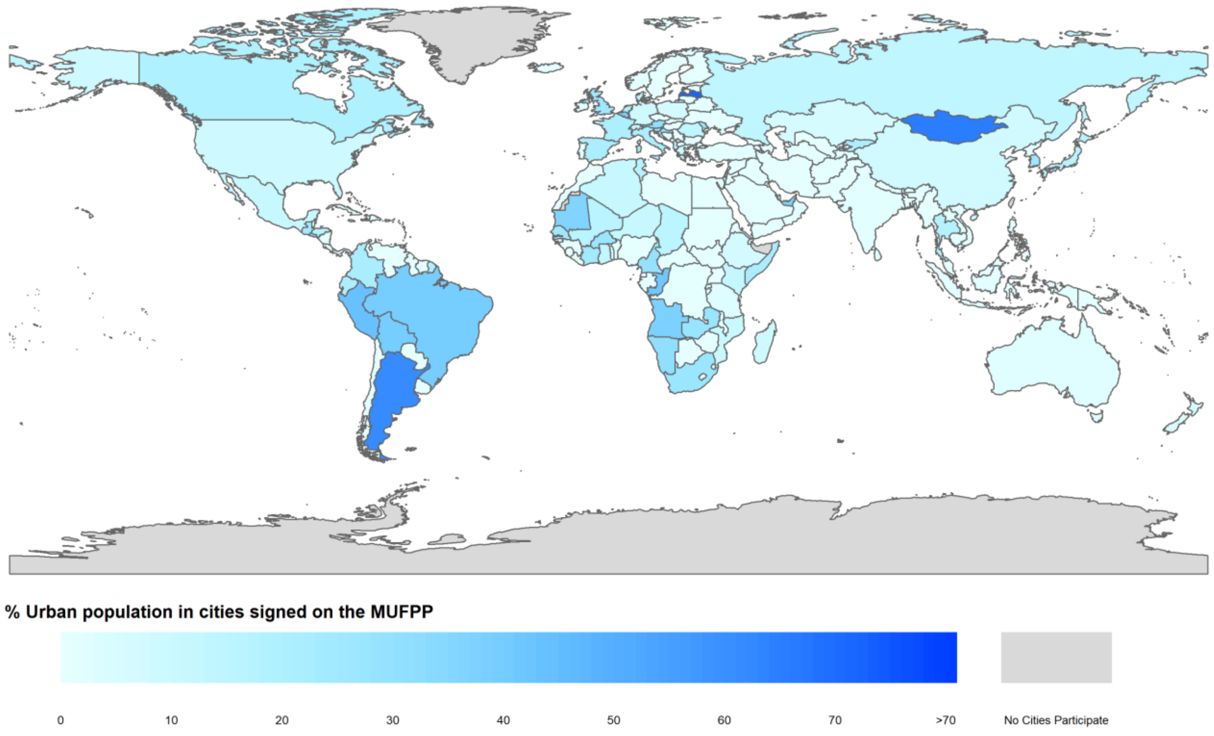

Basemap obtained using the R package 'rnaturalearth' which accesses Natural Earth v4.1.0 (March 2018) maps which are wholly in the public domain.

1.4.5 Percent urban population living in cities signed onto the Milan Urban Food Policy Pact, 2020, by country and region

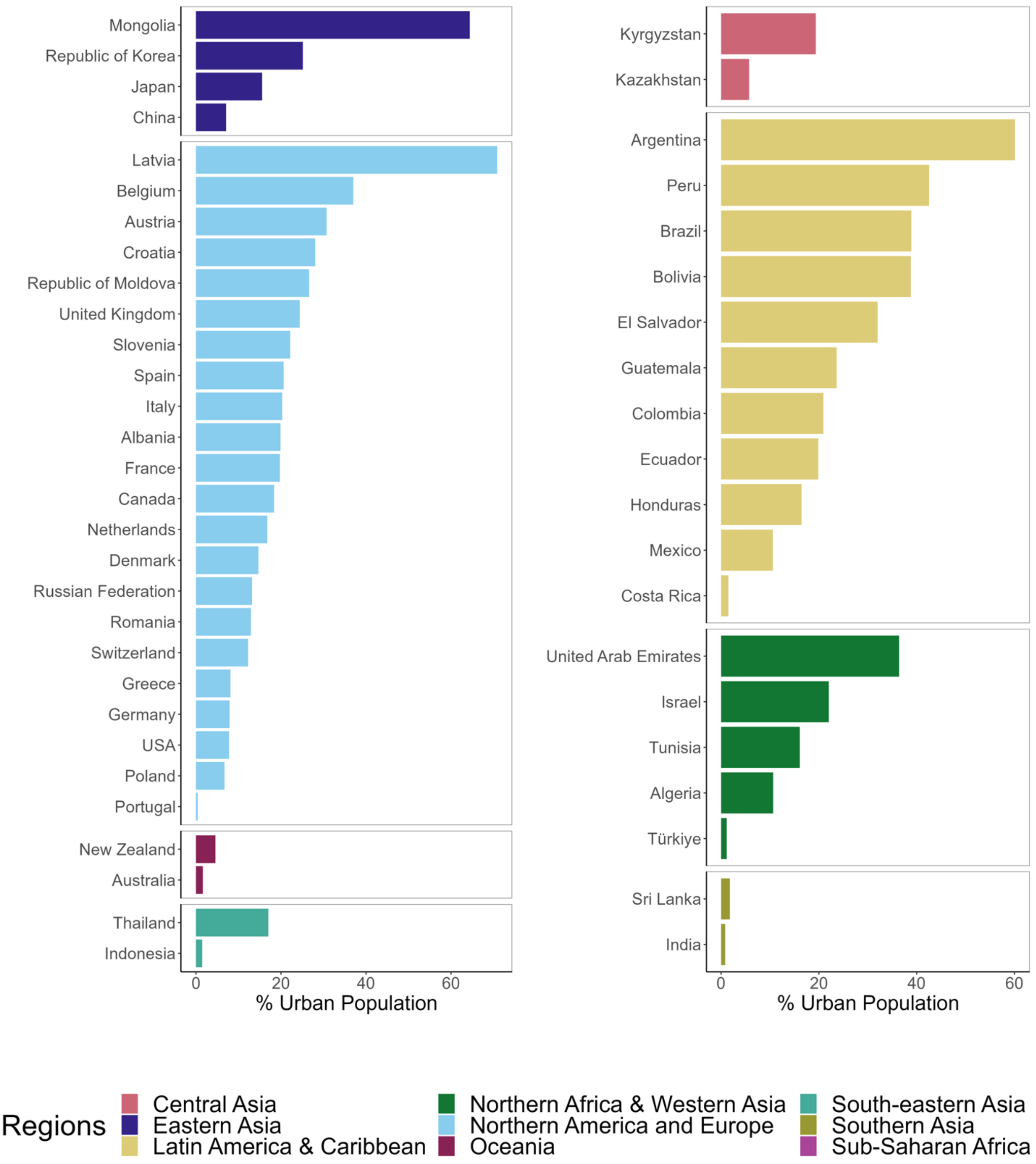

1.4.6 Degree of legal recognition of the Right to Food, 2021

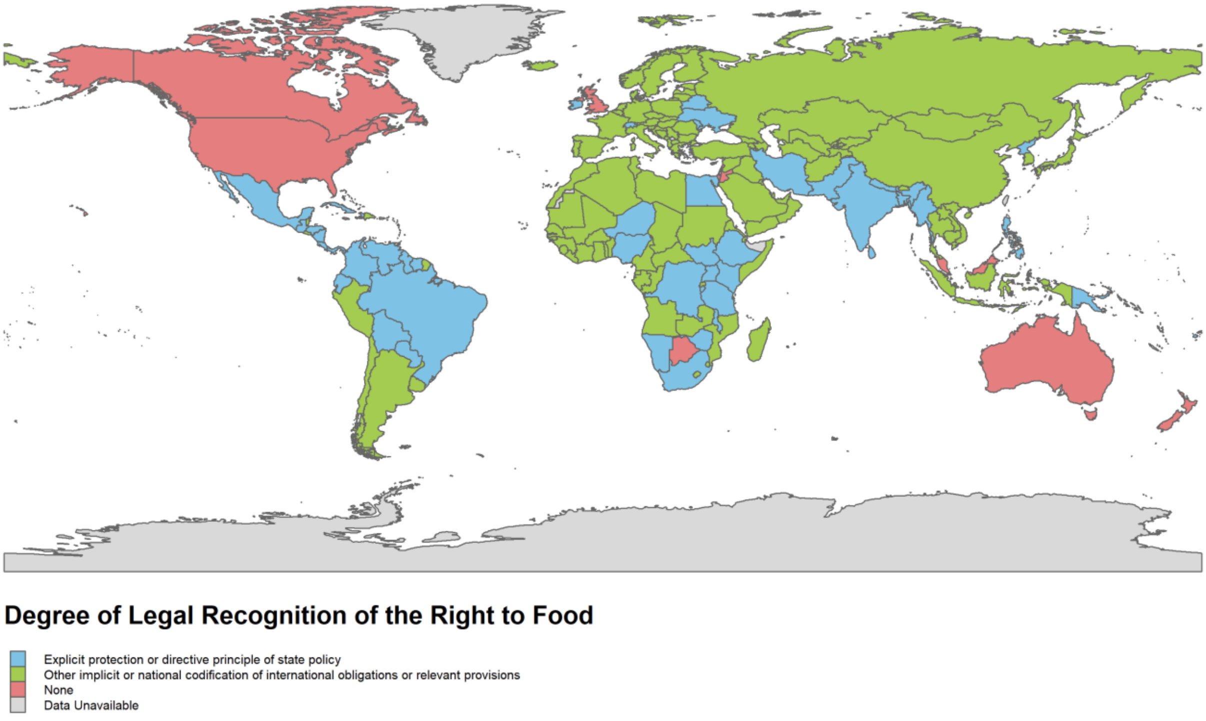

Basemap obtained using the R package 'rnaturalearth' which accesses Natural Earth v4.1.0 (March 2018) maps which are wholly in the public domain. Data as of 2021 update to the FAOLEX database.

1.4.7 Presence of a food policy transformation pathway (from the UN Food Systems Summit processes), 2022

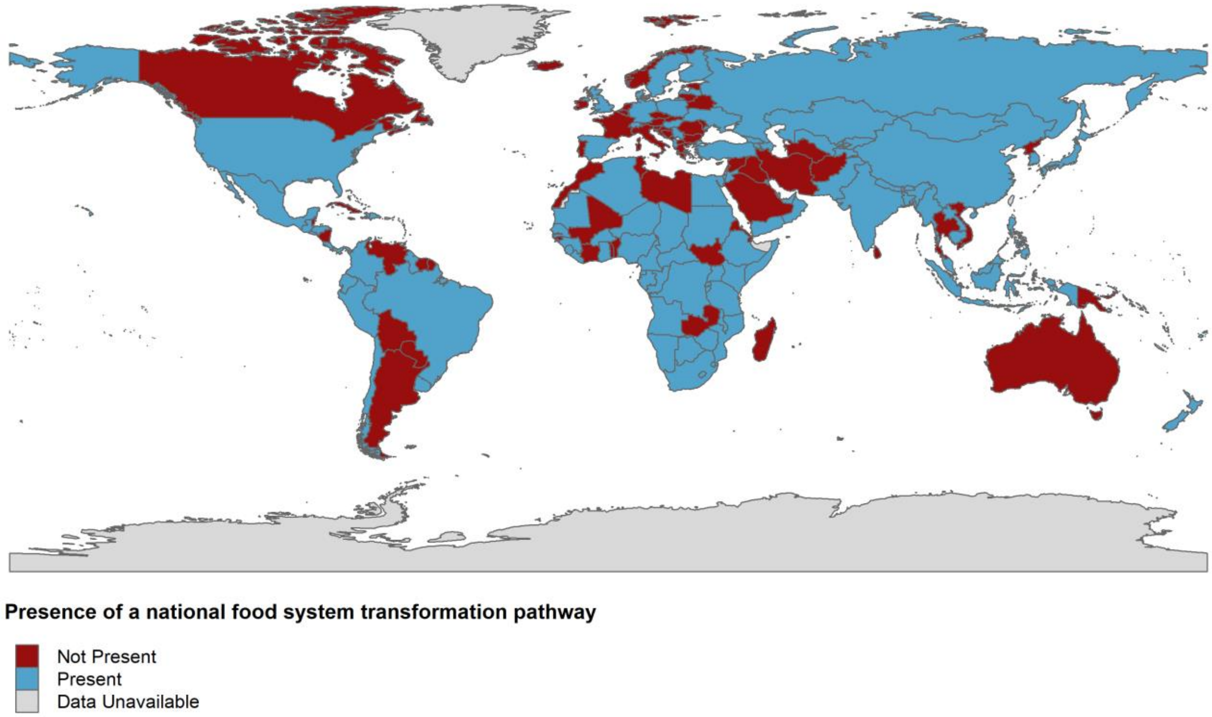

Basemap obtained using the R package ‘rnaturalearth’ which accesses Natural Earth v4.1.0 (March 2018) maps which are wholly in the public domain. Data current as of October 2022.

1.4.8 Government Effectiveness Index, 2000-2020, by region

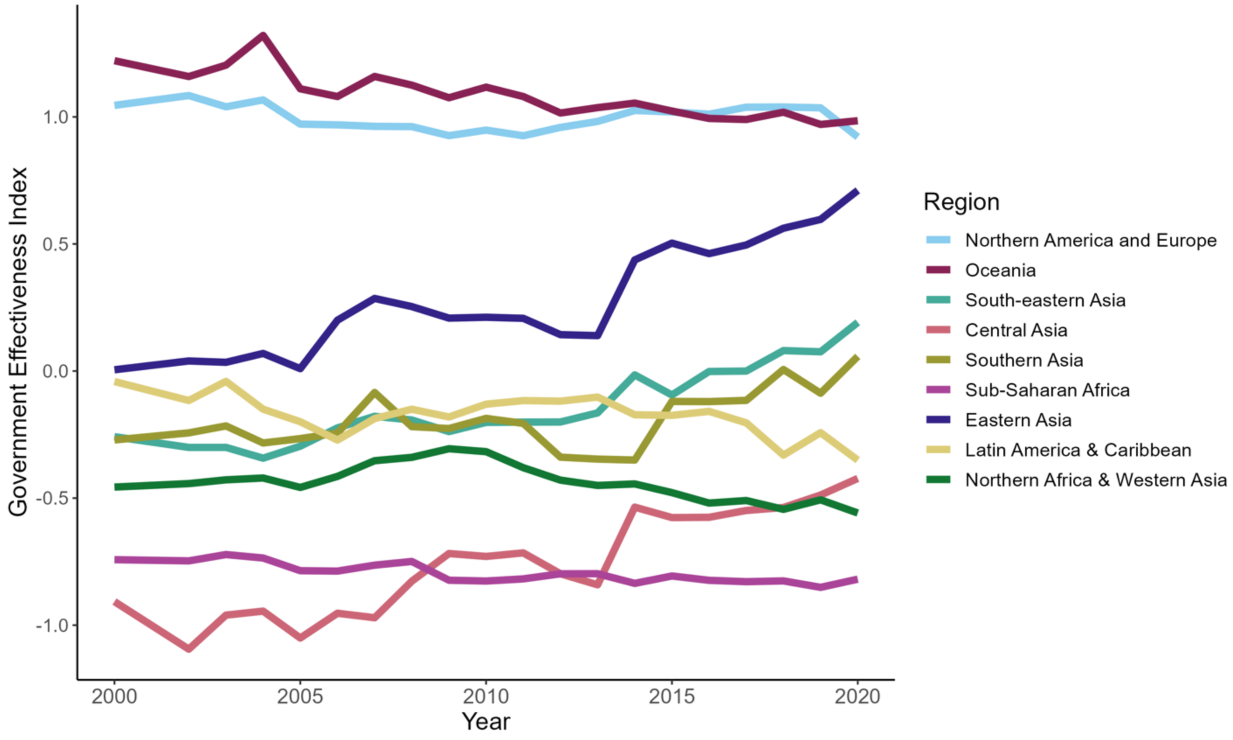

Population-weighted regional means.

1.4.9 Government Effectiveness Index, 2000-2020, by income group

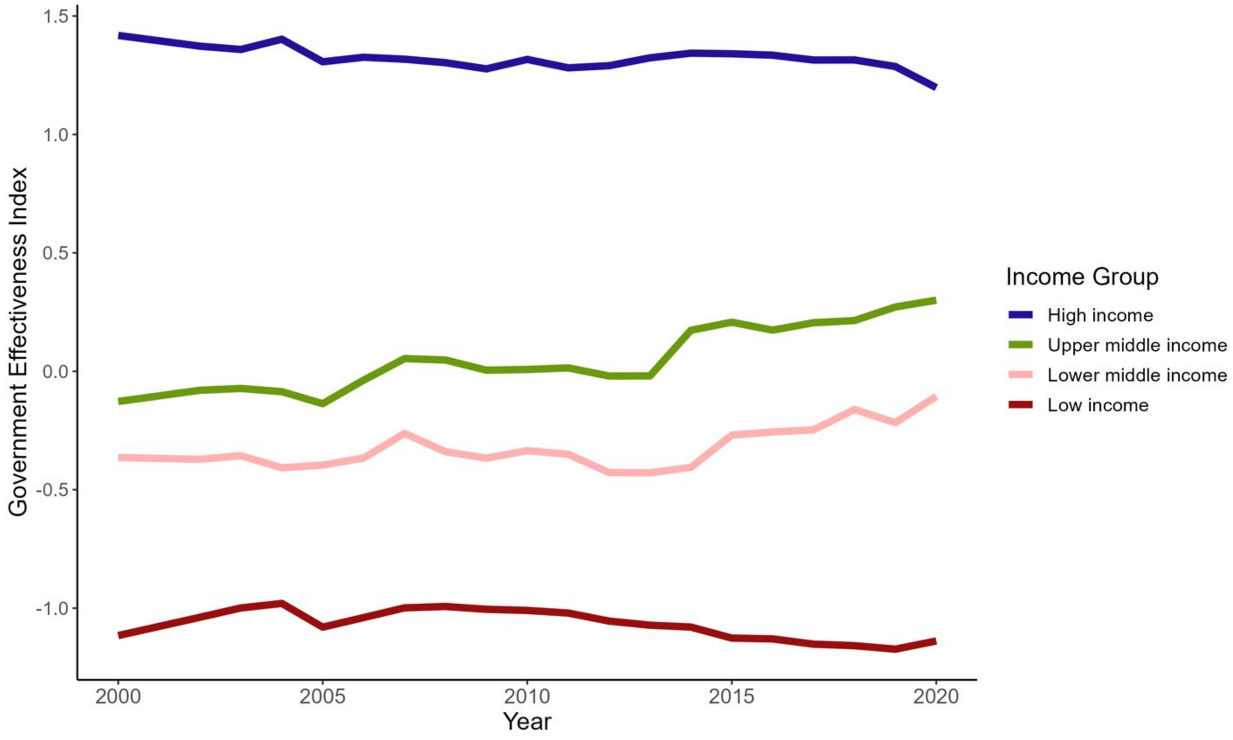

Population-weighted income group means.

1.4.10 International Health Regulations State Party Assessment report (IHR SPAR), Food safety capacity, 2020

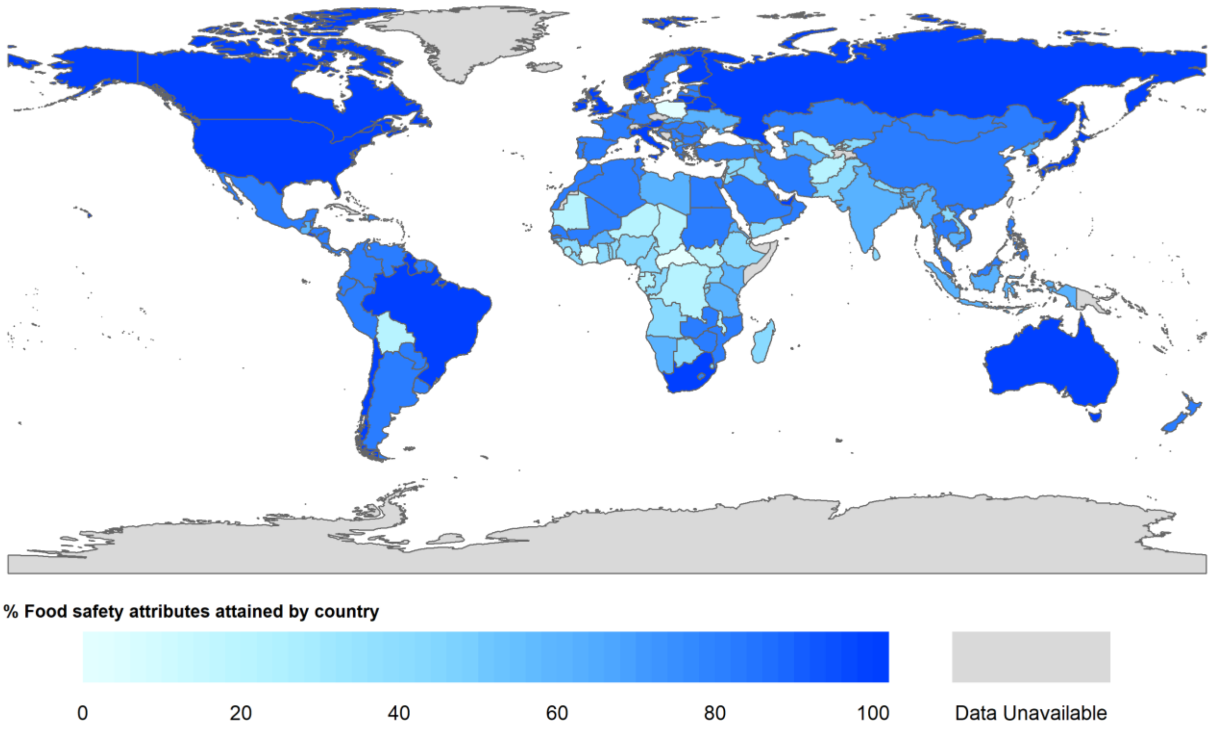

Basemap obtained using the R package ‘rnaturalearth’ which accesses Natural Earth v4.1.0 (March 2018) maps which are wholly in the public domain.

1.4.11 Presence of health-related food taxes, 2021

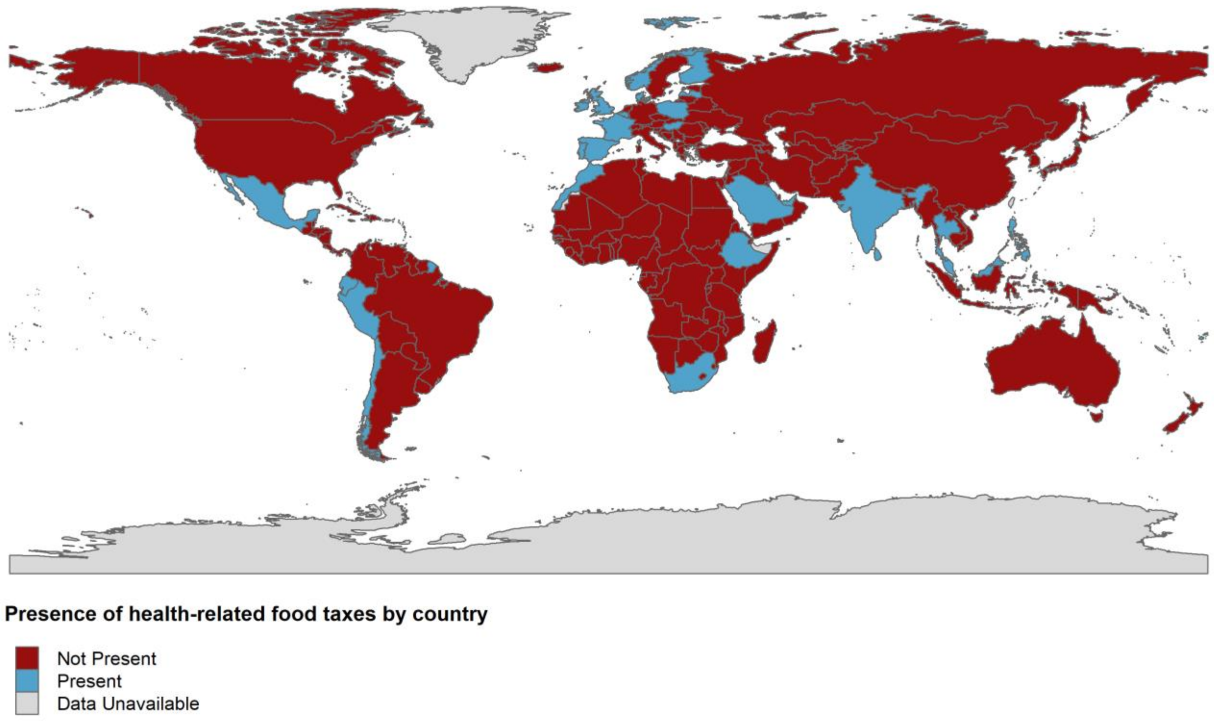

Basemap obtained using the R package 'rnaturalearth' which accesses Natural Earth v4.1.0 (March 2018) maps which are wholly in the public domain. Data as of 2021 update to the World Cancer Research Fund NOURISHING database.

1.4.12 V-Dem Accountability index, 2021

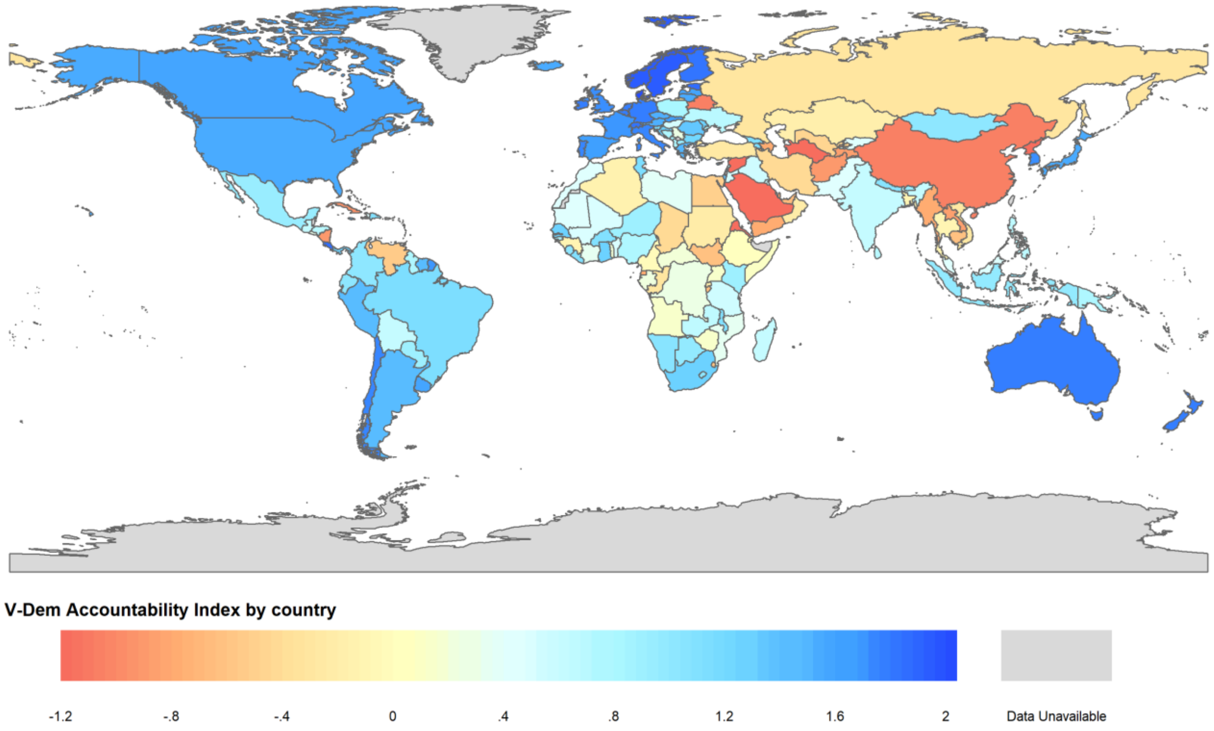

Basemap obtained using the R package 'rnaturalearth' which accesses Natural Earth v4.1.0 (March 2018) maps which are wholly in the public domain.

1.4.13 V-Dem Accountability index, 2000-2021, by region

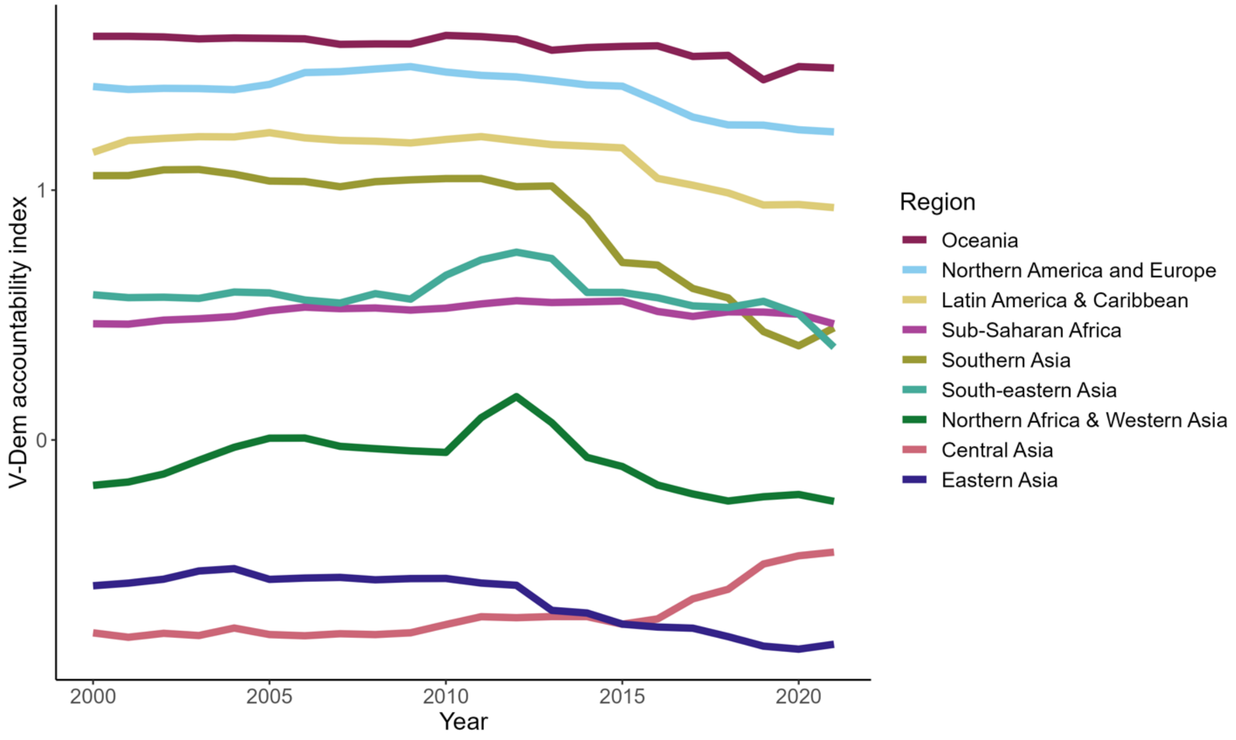

Population-weighted regional means.

1.4.14 V-Dem Accountability index, 2000-2021, by income group

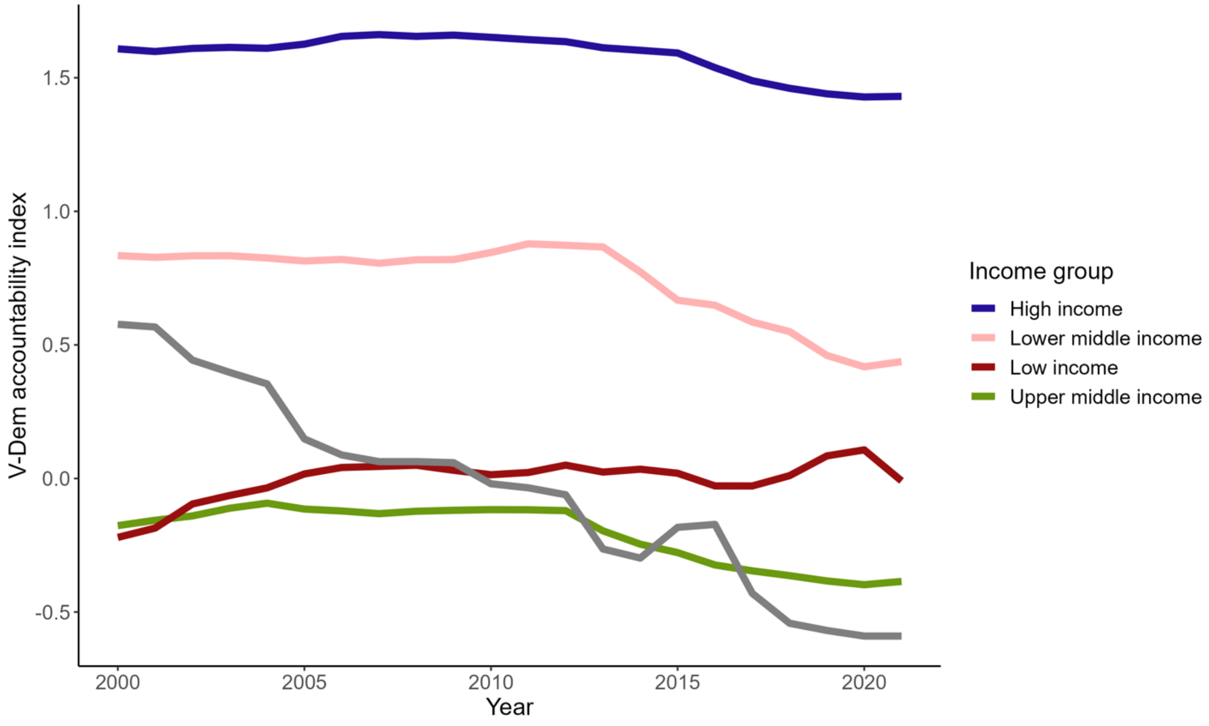

Population-weighted income group means.

1.4.15 Open Budget Index score, by income group

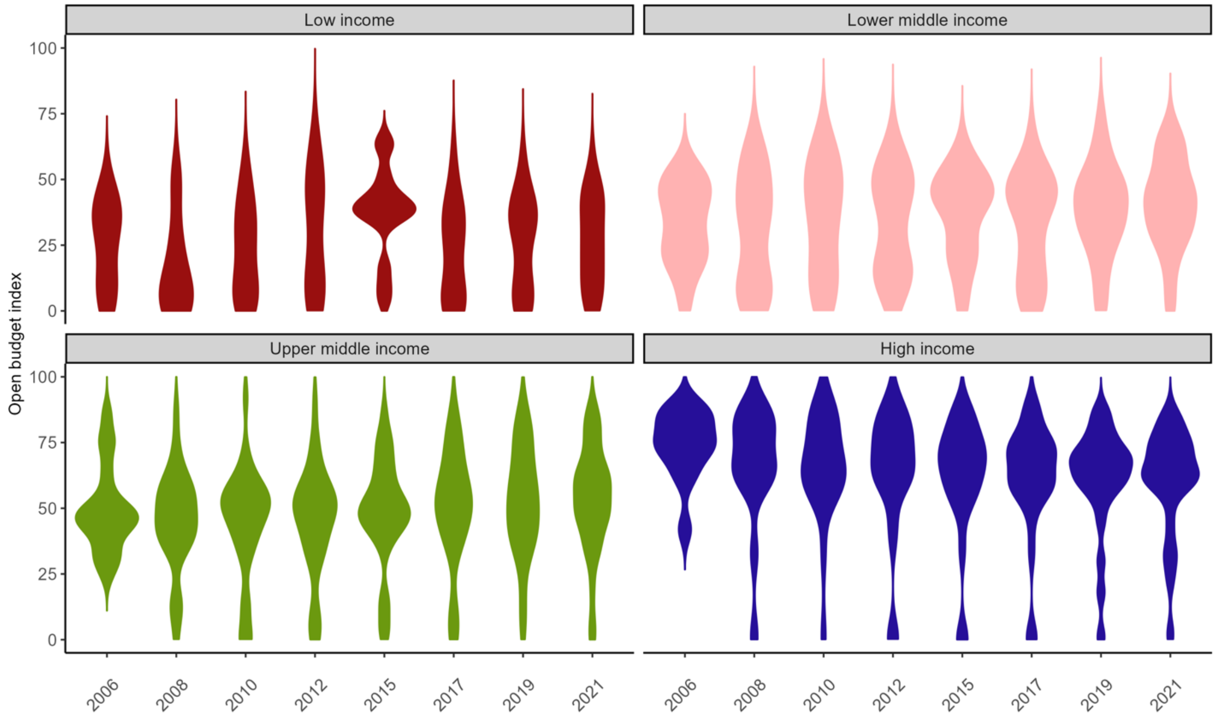

Sample size by income group and year provided in the following table:

Income group	2006	2008	2010	2012	2015	2017	2019	2021
Low income	5	12	14	14	15	19	20	22
Lower middle income	23	29	30	35	35	39	39	39
Upper middle income	19	28	29	29	29	31	32	33
High income	12	15	20	21	22	25	25	25

1.4.16 Guarantees for public access to information, by year of adoption

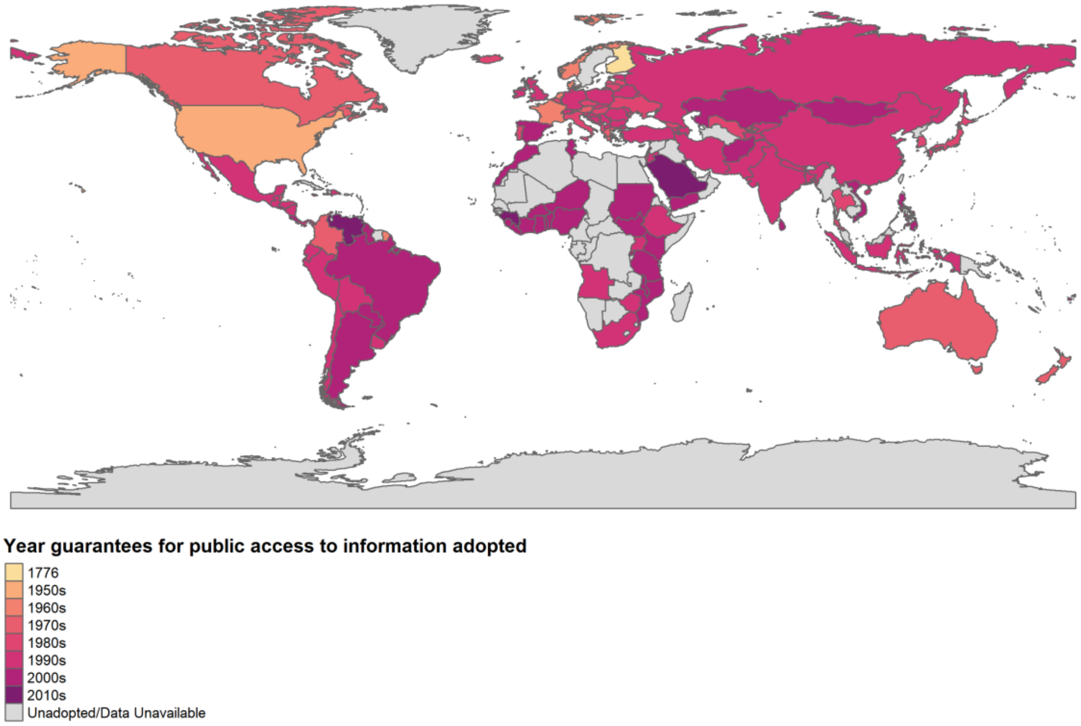

Basemap obtained using the R package ‘rnaturalearth’ which accesses Natural Earth v4.1.0 (March 2018) maps which are wholly in the public domain.

1.5.1 Exposure to shocks: Ratio of total damages of all disasters to GDP, 2012-2021

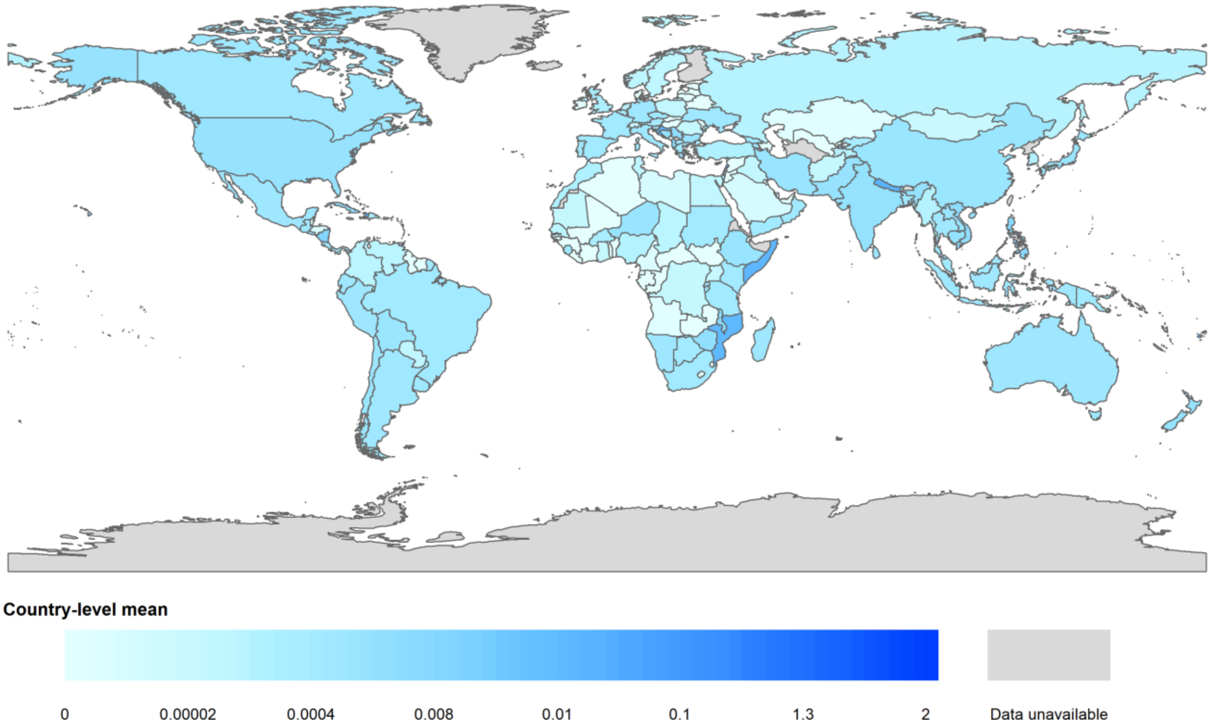

Basemap obtained using the R package ‘rnaturalearth’ which accesses Natural Earth v4.1.0 (March 2018) maps which are wholly in the public domain.

1.5.2 Dietary sourcing flexibility index, by nutrient / food group and country income level, 2018

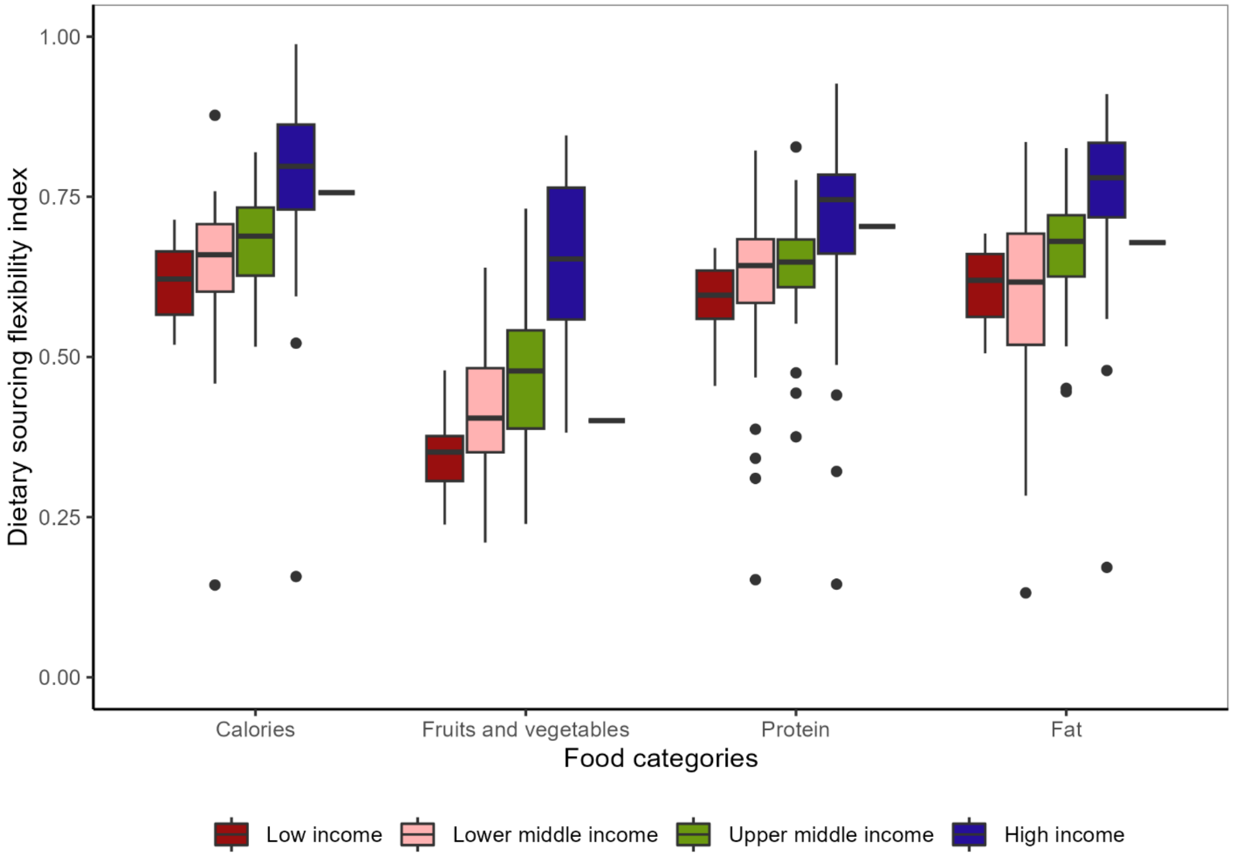

Calories, fruits and vegetables, protein, and fat are the total value from all sources. Sample size is 157 countries. Boxplot illustrates a box of the 25th to 75th percentiles with the horizontal line at the median, whiskers (vertical lines) extend the length of 1.5 times the interquartile range. All outliers beyond 1.5 times the interquartile range are shown individually.

1.5.3 Dietary sourcing flexibility index, Calories, 2018

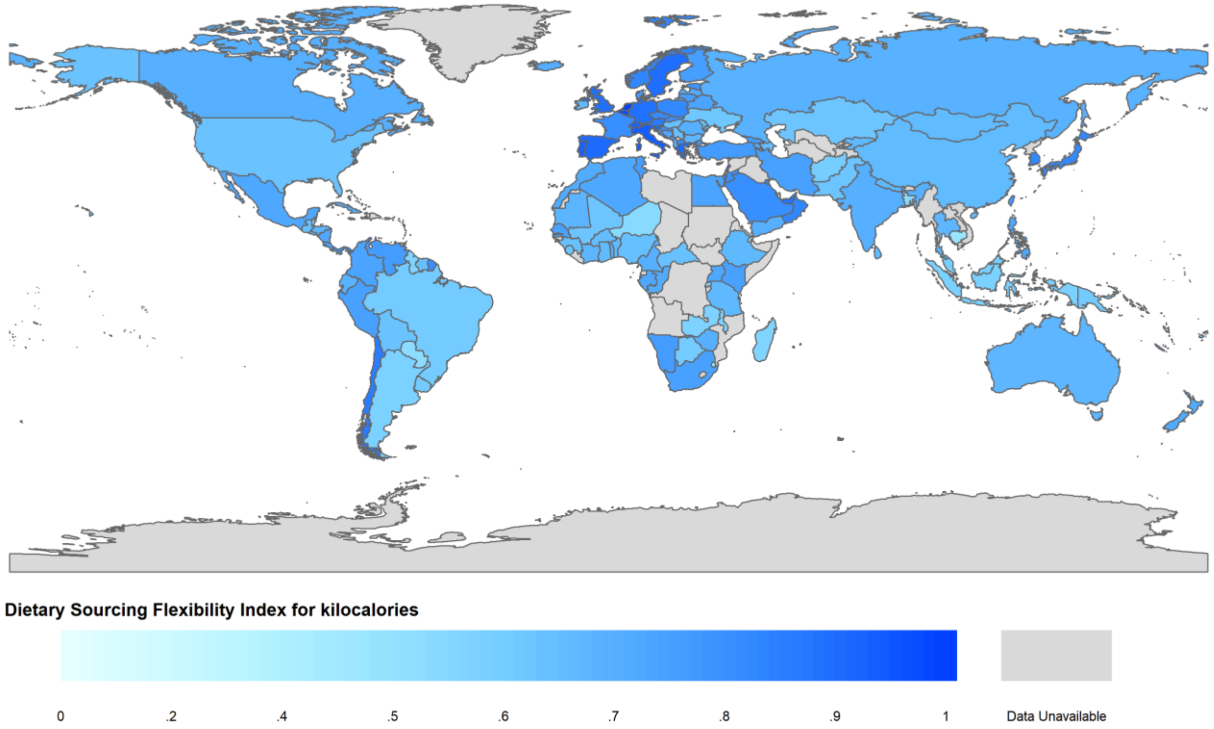

Basemap obtained using the R package ‘rnaturalearth’ which accesses Natural Earth v4.1.0 (March 2018) maps which are wholly in the public domain.

1.5.4 Mobile cellular subscriptions (per 100 people), 2000-2020, by region

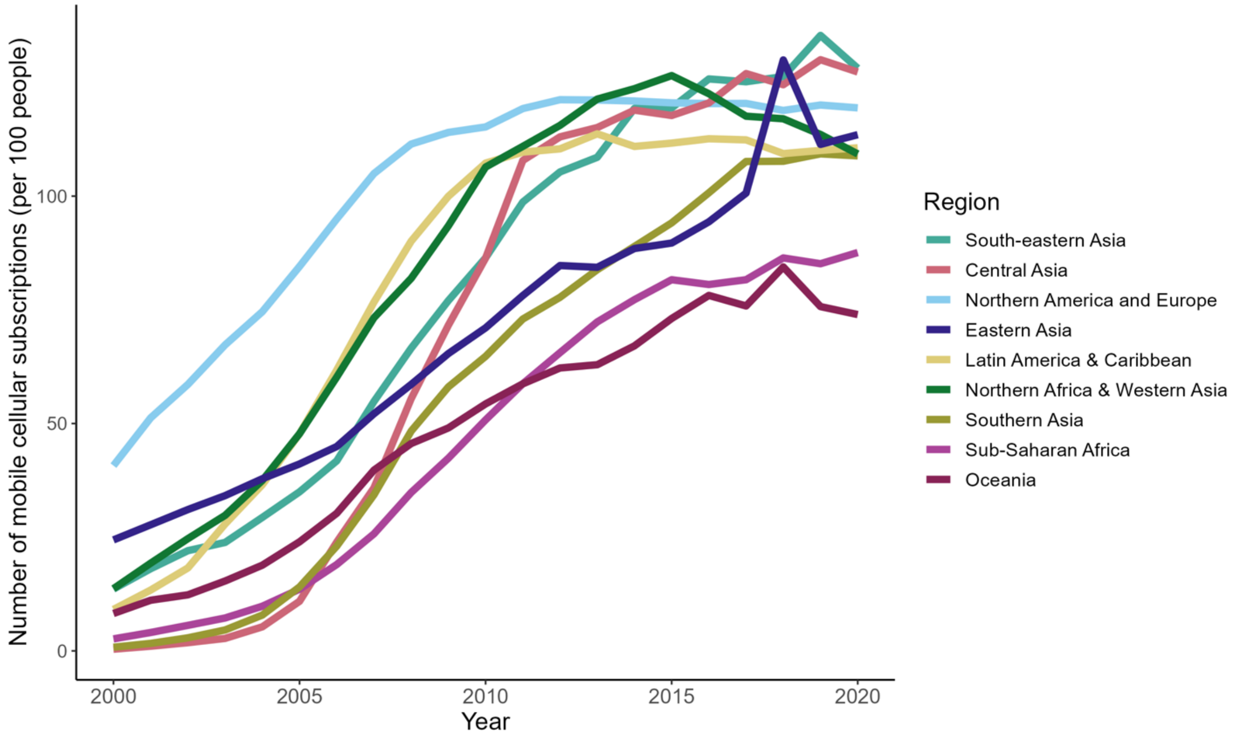

Unweighted mean by region.

1.5.5 Mobile cellular subscriptions (per 100 people), 2011-2020 average

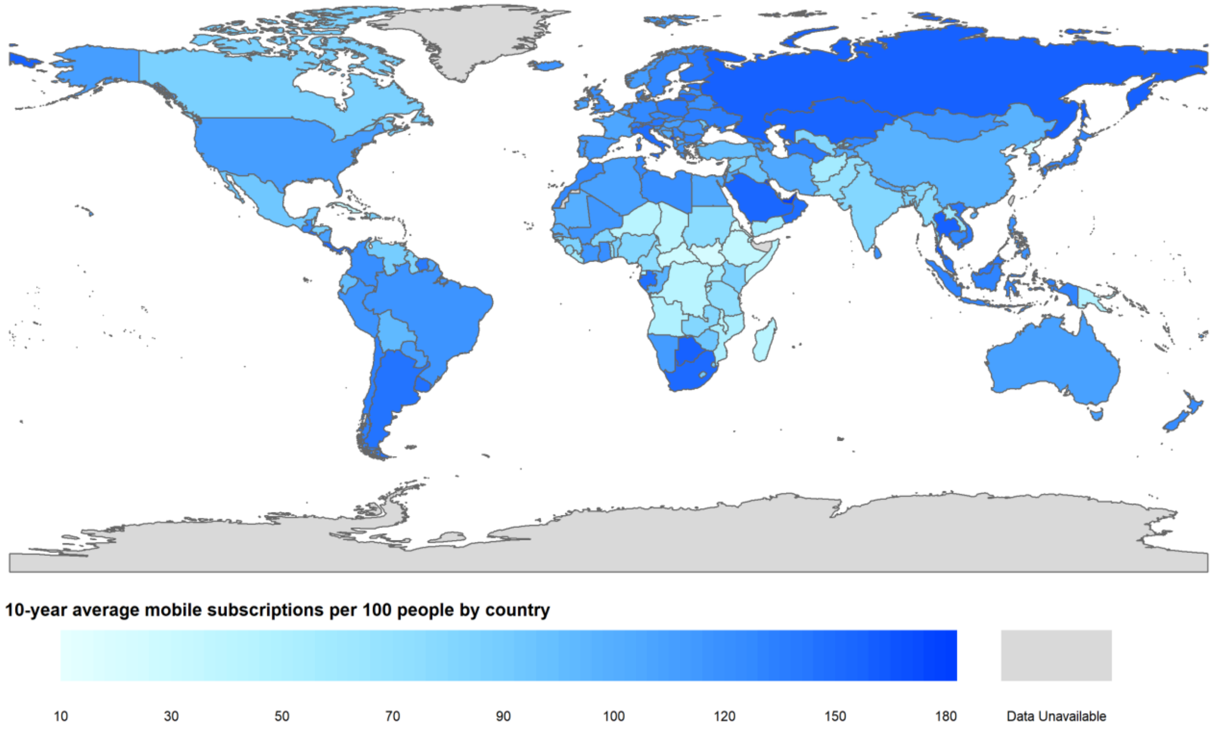

Basemap obtained using the R package ‘rnaturalearth’ which accesses Natural Earth v4.1.0 (March 2018) maps which are wholly in the public domain.

1.5.6 Social capital index, 2007-2021, by region

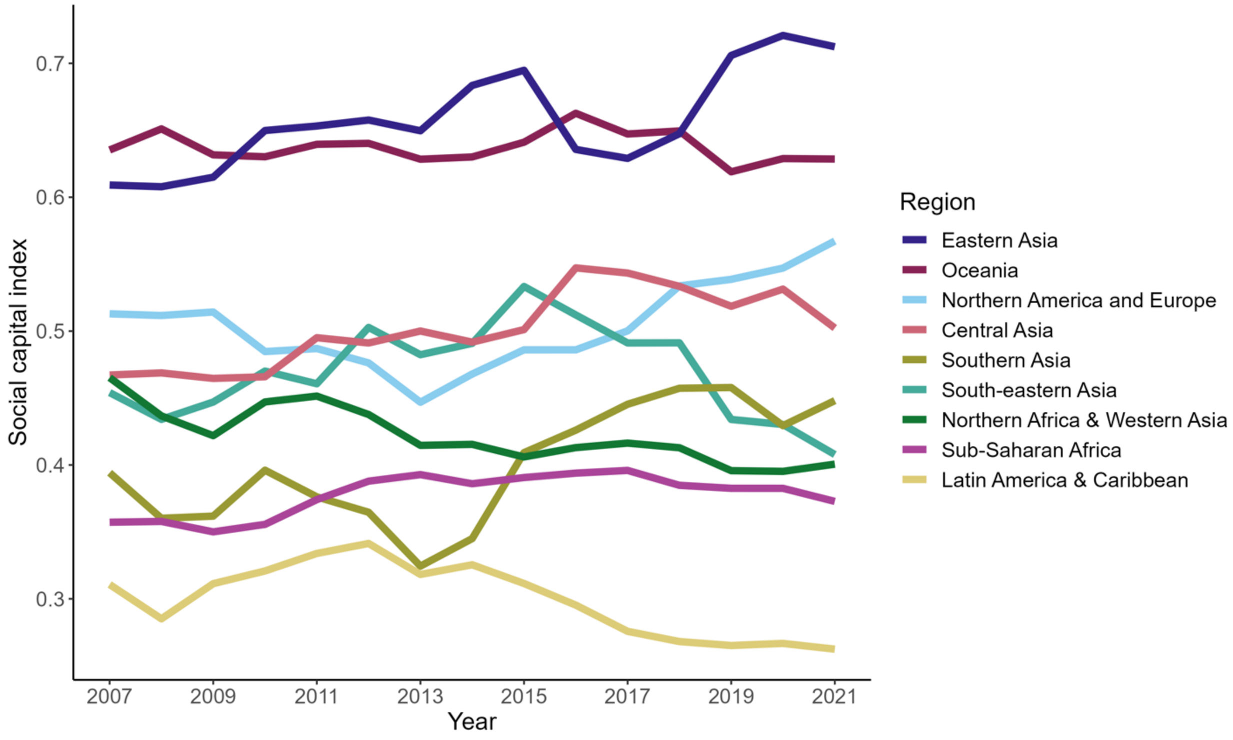

Population-weighted regional means.

1.5.7 Social capital index, 2007-2021, by income group

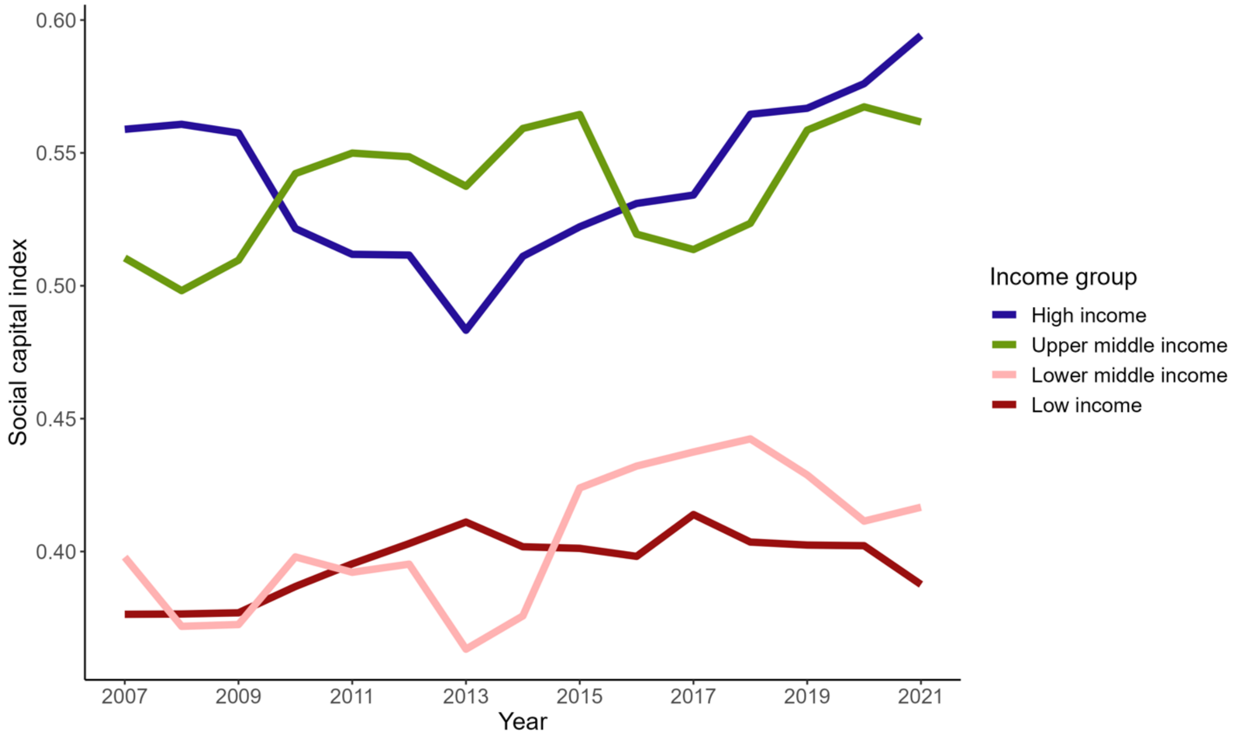

Population-weighted income group means

1.5.8 Social capital index, 2017-2021

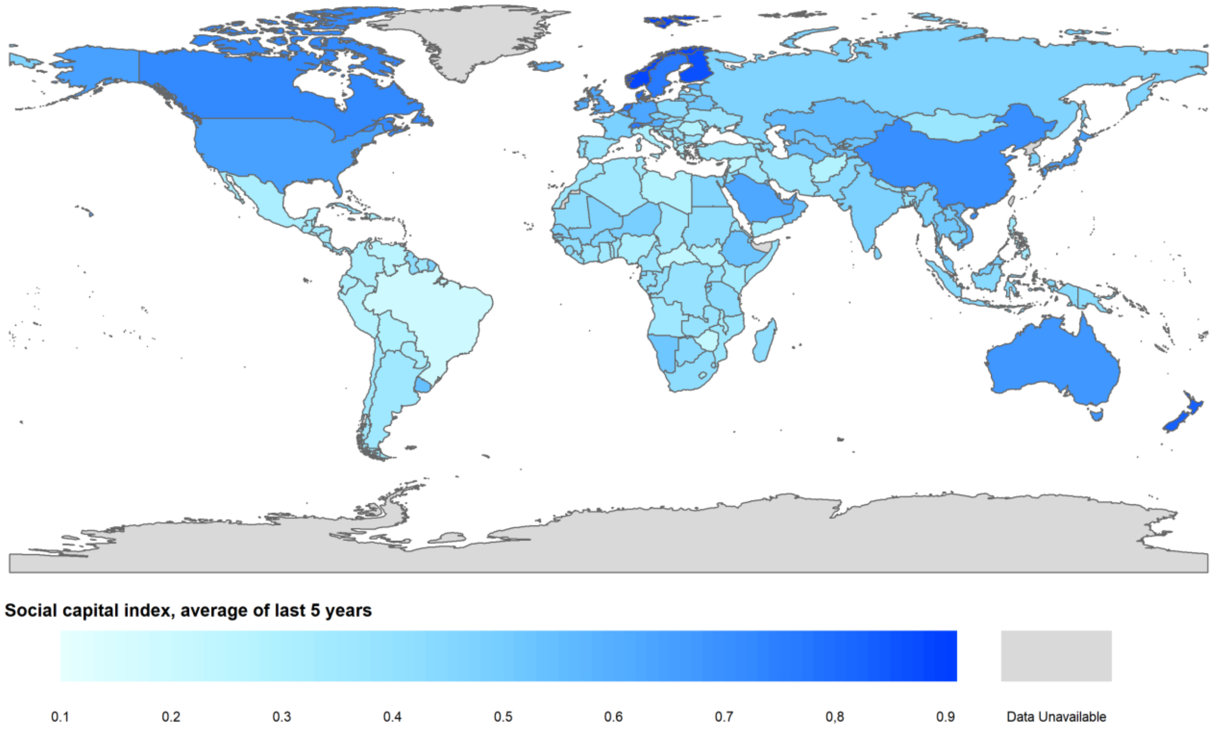

Basemap obtained using the R package ‘rnaturalearth’ which accesses Natural Earth v4.1.0 (March 2018) maps which are wholly in the public domain.

1.5.9 Percentage of agricultural land with minimum level of species diversity (crop and pasture), 2010

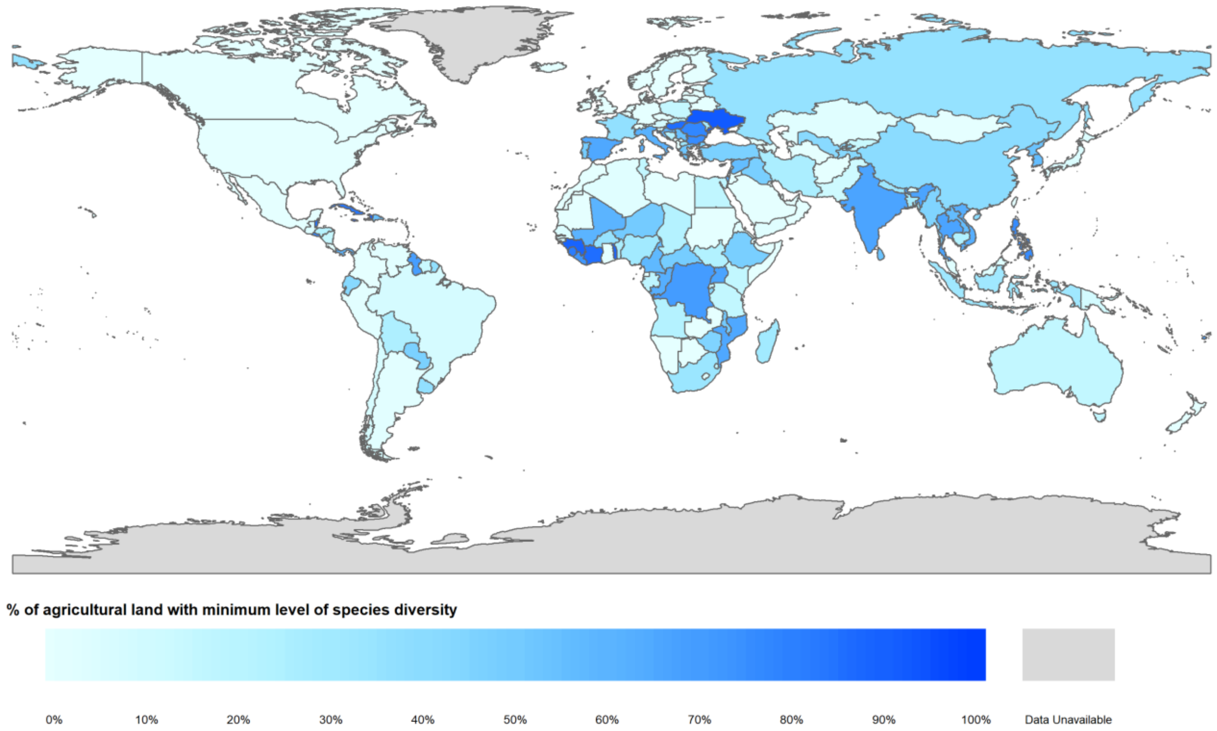

Basemap obtained using the R package ‘rnaturalearth’ which accesses Natural Earth v4.1.0 (March 2018) maps which are wholly in the public domain.

1.5.10 Number of wild useful plants for food and agriculture secured in conservation facilities (SDG 5.2.a), 2020

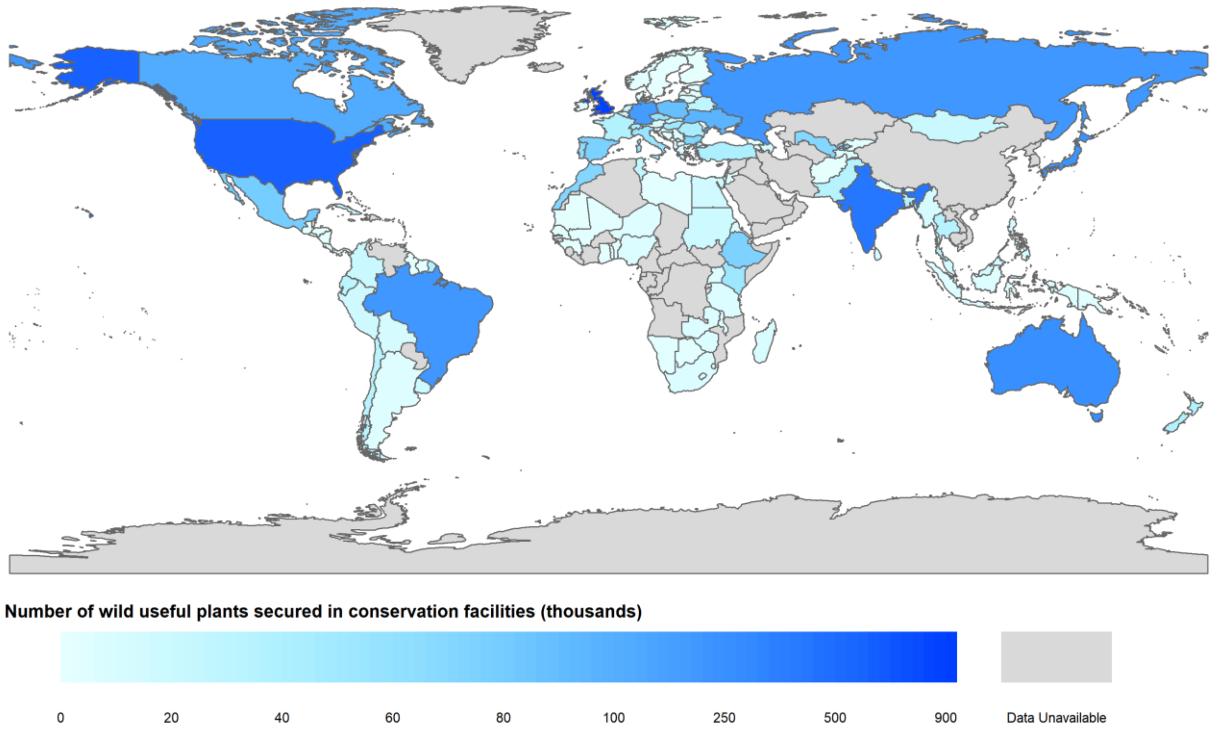

Basemap obtained using the R package ‘rnaturalearth’ which accesses Natural Earth v4.1.0 (March 2018) maps which are wholly in the public domain.

1.5.11 Number of animal genetic resources for food and agriculture secured in conservation facilities (SDG 5.2.b), 2021

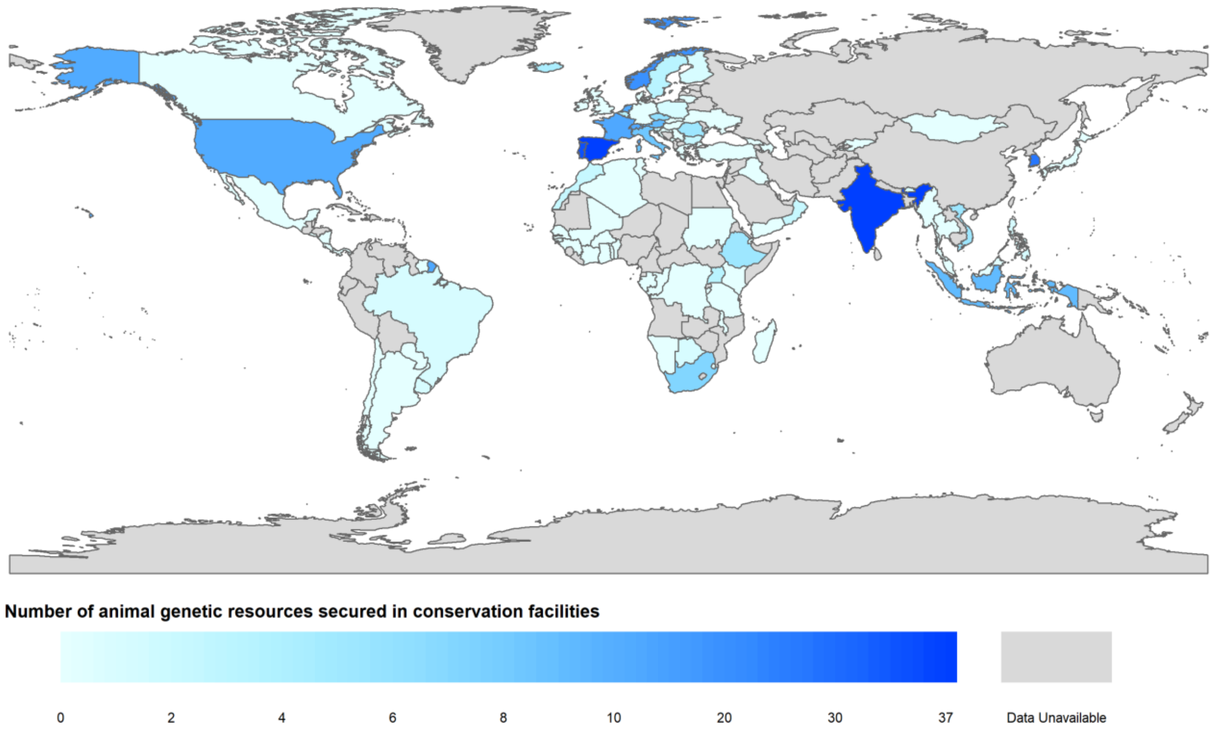

Basemap obtained using the R package 'rnaturalearth' which accesses Natural Earth v4.1.0 (March 2018) maps which are wholly in the public domain.

1.5.12 Prevalence of reduced coping strategies, 2021

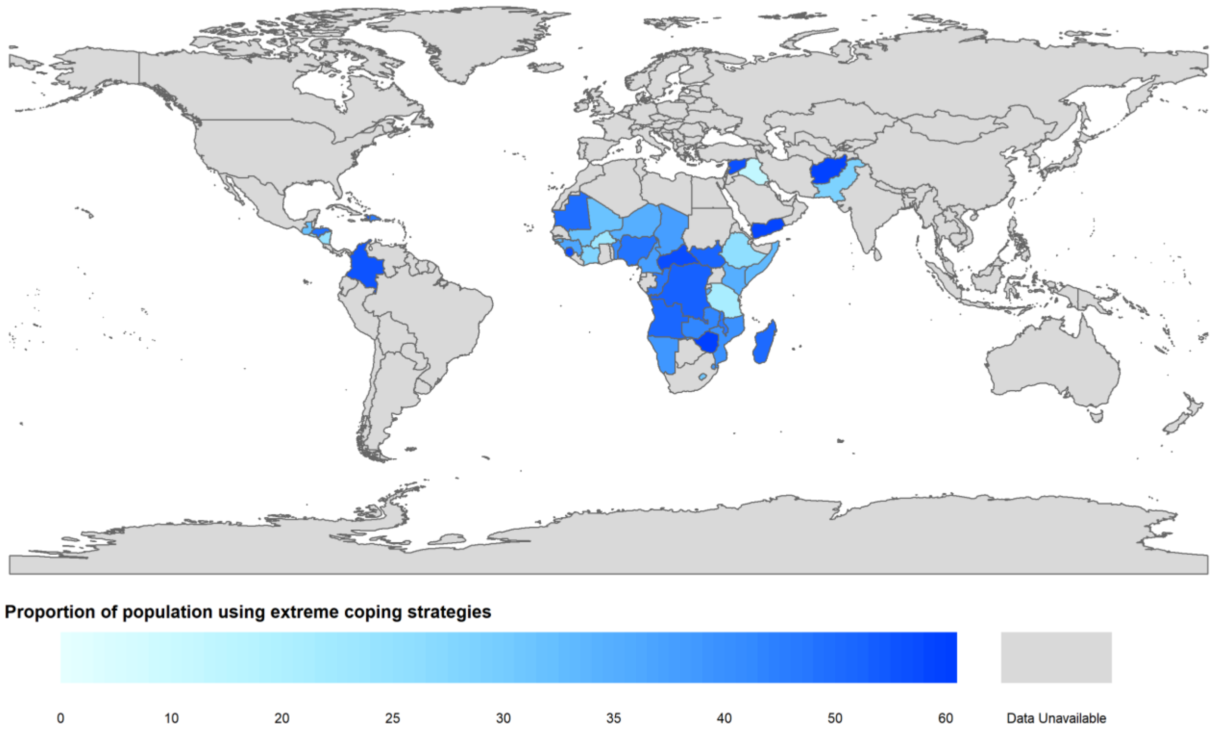

Basemap obtained using the R package 'rnaturalearth' which accesses Natural Earth v4.1.0 (March 2018) maps which are wholly in the public domain. Dichotomous classification of reduced coping strategies uses a threshold of an rCSI score >=19. Based on daily data, the country-year prevalence is defined as the highest daily observation at any point during the year.

1.5.13 Food price volatility, 2019-2021 (average)

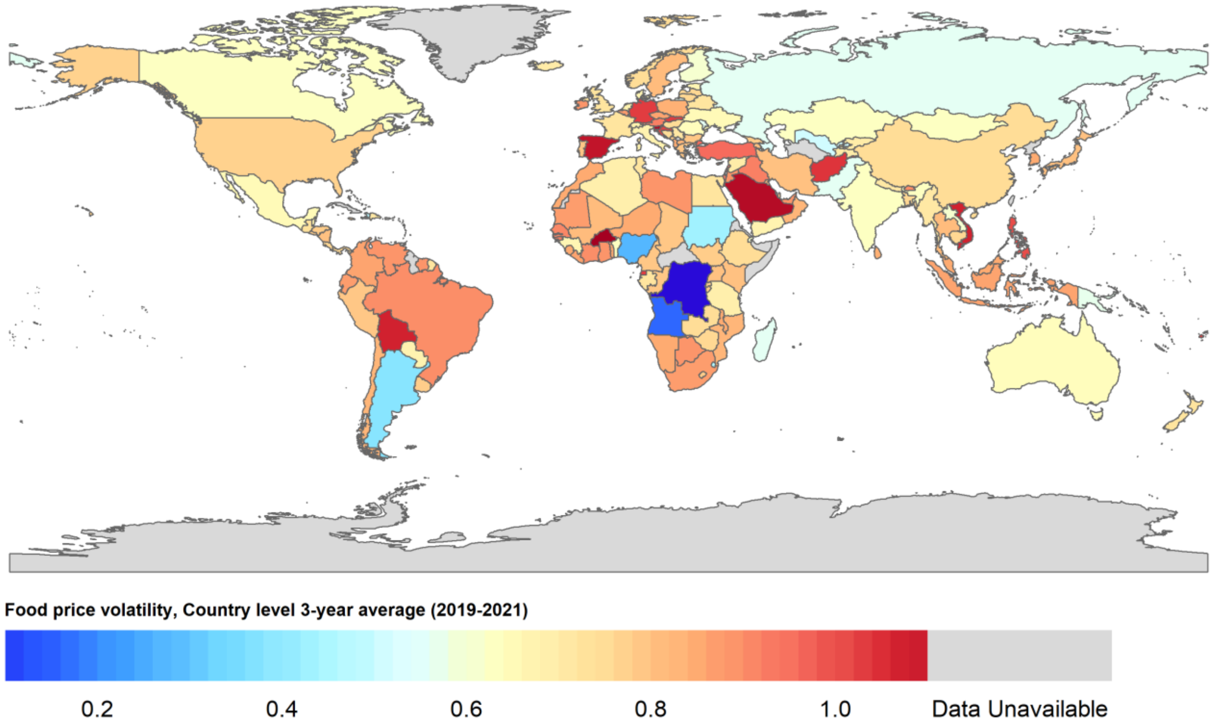

Basemap obtained using the R package ‘rnaturalearth’ which accesses Natural Earth v4.1.0 (March 2018) maps which are wholly in the public domain.

1.5.14 Food supply variability, 2000-2020, by region

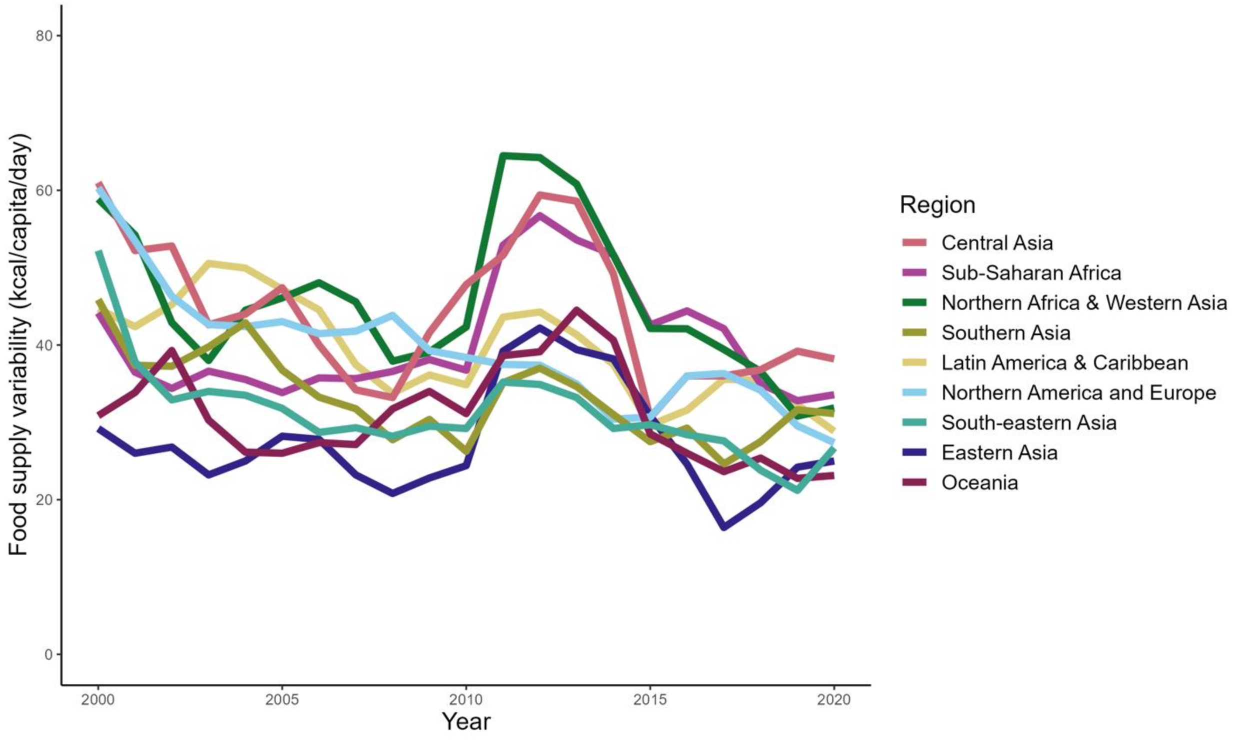

Unweighted regional means.

1.5.15 Food supply variability, 2000-2020, by income group

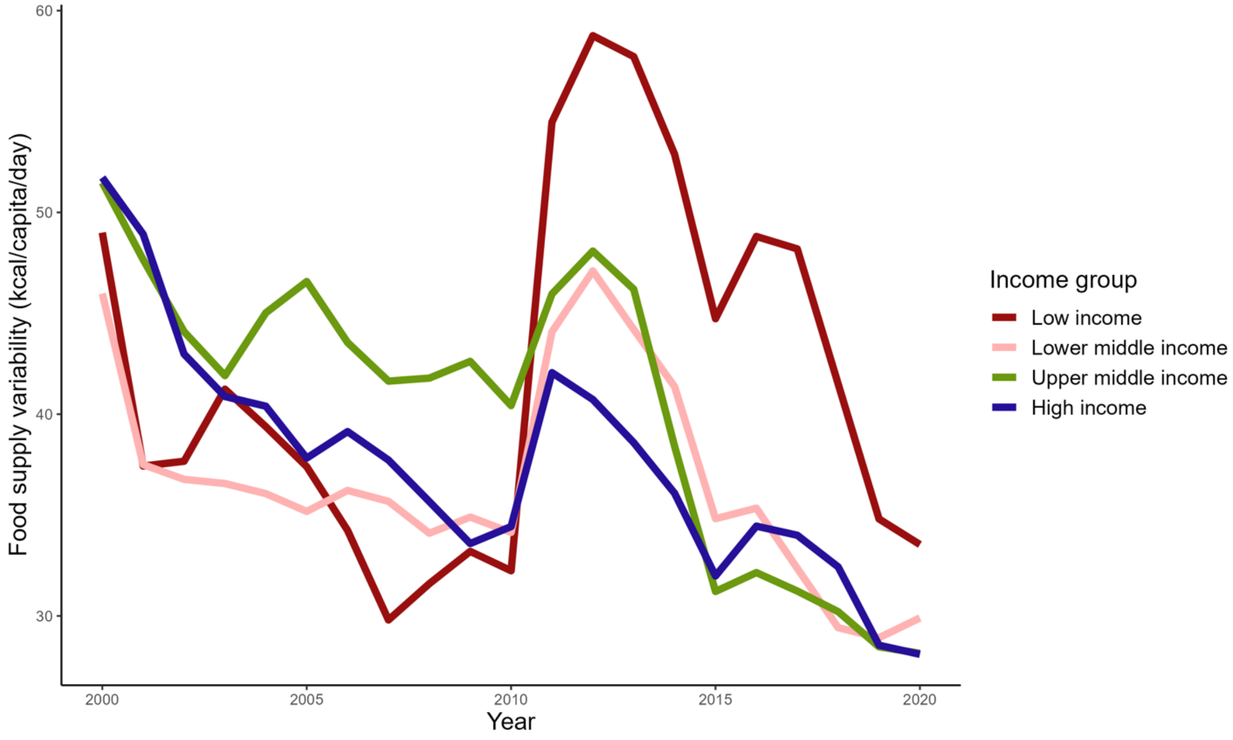

Unweighted income group means.

1.5.16 Food supply variability, 10-year average, (2011-2020)

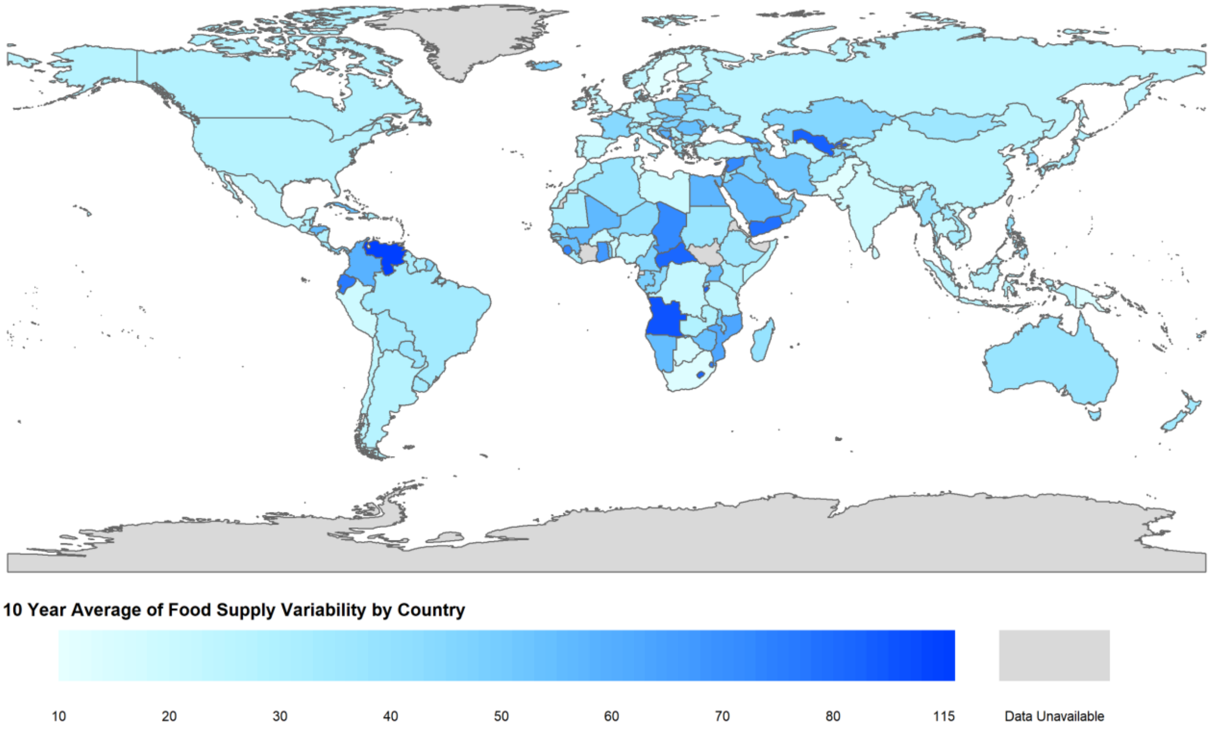

Basemap obtained using the R package ‘rnaturalearth’ which accesses Natural Earth v4.1.0 (March 2018) maps which are wholly in the public domain.

1.5.17 Food System Resilience

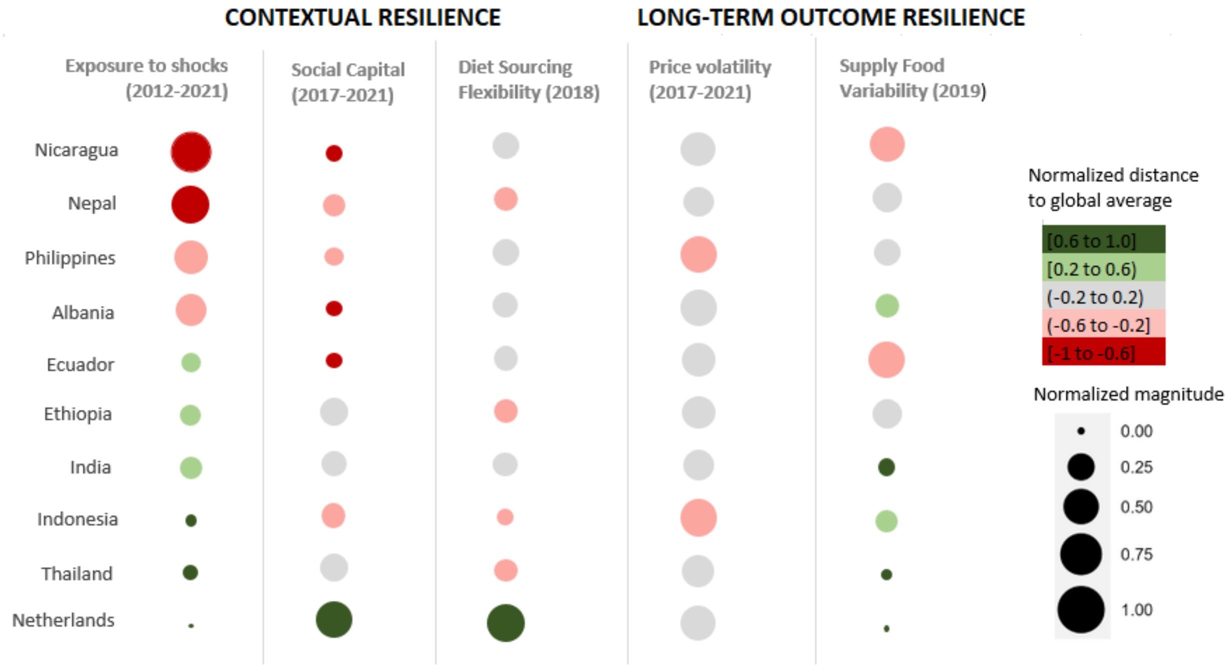

Food system resilience. Exploratory analysis based on five of the eight indicators initially proposed to measure food system resilience at country level. Three of the indicators capture the contextual dimension of resilience (exposure to shocks; social capital; and diet sourcing flexibility), while two indicators measure countries' food system resilience outcomes through the food price volatility and supply food variability. Ten countries were picked to illustrate the varied situation that characterizes the countries at present in relation to their food system resilience. To allow for comparison, the indicators have been normalized in two separate ways: their original value has been (i) normalized between 0 and +1 (thereafter represented by the size of the circle) and (ii) with respect to the distance to the world average (represented by the color of the circle, ranging from -1 (red) to +1 (green), while ensuring that the directionality of the gap with respect to the world average is appropriately accounted for.

Methodology note: The countries shown in this figure were selected by eliminating countries with fewer than 7 observations of exposure to shocks over the 10-year period (2012-2021) and where data were non-missing for all other indicators, resulting in a subset of 89 country-year observations. Exposure to shocks was calculated as the average of the highest three values per country over the 10-year period and the data were winsorized at the upper bound to the third-highest value for the purposes of visualization (Croatia and Nepal outliers, specifically). Normalization for the coloring was calculated using min-max scaling to the 89 observations in the subset dataset. Distance to the mean (mean of the 89 observations remaining in the subset dataset) was directionally adjusted to align with the desirable direction of change (see **Extended Data Table 1**) but the average is not centered at zero. The normalization for the size of the marker was conducted with min-max scaling based on the 89 observations in the subset dataset.

Supplementary Information, Appendix 2: Candidate indicators ultimately excluded

Contents

Table 2.1 Excluded indicators considered by expert and stakeholder consultations……………………2
Table 2.2 Indicators excluded prior to expert and stakeholder consultations, with rationale for exclusion…………………………………………………………………………………………..7
References………………………………………………………………………………………21

Table 2.1 Excluded indicators considered by expert and stakeholder consultations

** Denotes indicators added at the recommendation of respondents/participants of the survey and stakeholder consultation

Indicators considered for survey and stakeholder consultation	Data sources(s)	Reason Excluded
Diets, Nutrition & Health		
Egg and/or flesh food consumption among infants and young children(age 6-23 months)	UNICEF	This indicator is being tracked by UNICEF. Feedback from consultations did not rank this indicator as high as the others.
Sweet beverage consumption among infants and young children(age 6-23 months)	UNICEF	This indicator is now being collected in DHS-8 core module and MICS as of 2021. *Data are not yet available
Unhealthy food consumption among infants and young children(age 6-23 months)	UNICEF	This indicator is now being collected in DHS-8 core module and MICS as of 2021. *Data are not yet available
Consumption of all five recommended food groups among infants and young children(age 6-23 months)	UNICEF	This indicator is not currently reported by UNICEF but can be calculated from existing DHS and MICS source data (food group consumption). It mirrors the adult indicator.
Global Dietary Recommendations score (GDR score)	Gallup World Poll	It is the combined score of NCD-Protect and NCD-Risk, so duplicates information captured in those indicators.
Animal-source food consumption	Gallup World Poll	This indicator was considered because it is analogous to an IYCF indicator being tracked by UNICEF. Feedback from consultations did not rank this indicator as high as the others, in part because the directionality and policy relevance is less clear than some of the other diet indicators.
Pulses, nuts, and seeds consumption	Gallup World Poll	This indicator was considered because WHO recommends consumption of pulses, nuts and seeds as part of a healthy diet. Feedback from consultations did not rank this indicator as high as the others.
Whole grains consumption	Gallup World Poll	This indicator was considered because WHO recommends consumption of whole grains as part of a healthy diet. Feedback from consultations did not rank this indicator as high as the others.
Sweet beverage consumption	Gallup World Poll	This indicator includes soft drinks (sodas, energy drinks, and sports drinks), juice, and sweetened tea and coffee. Given that in the adult population, sweetened tea and coffee are highly prevalent traditional beverages, the indicator of sugar-sweetened soft drinks was selected because it is more policy relevant than this one.
Unhealthy food consumption (composed of sweet foods + salty or fried snacks)	Gallup World Poll	In the interest of having a smaller set of indicators, only one indicator for unhealthy food/beverage consumption is included.
Fast food or instant noodles consumption	Gallup World Poll	In the interest of having a smaller set of indicators, only one indicator for unhealthy food/beverage consumption is included.
Number of people who cannot afford a healthy diet	Food Prices for Nutrition Data Hub (soon FAOSTAT)	Duplicates information captured in the “proportion of people who cannot afford a healthy diet”

Cost of each food group (starchy staples, fats/oils, pulses/nuts/seeds, animal-source foods, fruits, vegetables) (per person per day)	Food Prices for Nutrition Data Hub (soon FAOSTAT)	Somewhat duplicative of the included Cost of a Healthy Diet indicator, so omitted in the interest of having a smaller set of indicators.
Cost of a Healthy Diet relative to average food expenditure	Food Prices for Nutrition DataHub	It may be duplicative of the other mainstreamed affordability indicators, the number and proportion of people who cannot afford a healthy diet.
Share of dietary energy from roots, cereals, tubers (%)	FAOSTAT (Food Systems Dashboard)	It may be difficult to interpret, because while a high share indicates low availability of diverse foods / imbalanced foods supply, a low share does not necessarily mean a nutritionally balanced food supply (could mean high sugars and fats).
Availability/supply (g/day/capita) - pulses	FAOSTAT	It could be compared to food group recommendations to determine adequacy of per capita supply, but it does not reflect disparities in access or consumption.
Availability/supply (kcal/day/capita) - animal-source foods	FAOSTAT	It could be compared to food group recommendations to determine adequacy of per capita supply, but it does not reflect disparities in access or consumption.
Availability (amount/day/capita) of macronutrients and micronutrients	FAOSTAT food and diet domain	It can be used to determine adequacy of per capita supply, but it does not reflect disparities in access or consumption.
Coverage of iodized salt (% of households)	UNICEF	The Working Group focused on the ends / outcomes, policies are not considered outcome, but rather a means to the end.
Fortification legislation (Any / Mandatory / None)	Food Systems Dashboard; Food Fortification Initiative, GAIN, Iodine Global Network, and the Micronutrient Forum, Global Fortification Data Exchange - legislation per food item	The Working Group focused on the ends / outcomes, policies are not considered outcome, but rather a means to the end.
Implementation of marketing of breast-milk substitutes restrictions (Fully achieved/ Partially achieved / Not achieved)	World Health Organization - Noncommunicable Diseases Progress Monitor	The Working Group focused on the ends / outcomes, policies are not considered outcome, but rather a means to the end.
Existence of policies on marketing junk foods to children (binary)	WHO (Food Systems Dashboard)	The Working Group focused on the ends / outcomes, policies are not considered outcome, but rather a means to the end.
Mandatory regulation of broadcast food advertising to children	World Cancer Research Fund International NOURISHING policy database	The Working Group focused on the ends / outcomes, policies are not considered outcome, but rather a means to the end.
Clearly visible "interpretative" labels and warning labels	World Cancer Research Fund International NOURISHING policy database	The Working Group focused on the ends / outcomes, policies are not considered outcome, but rather a means to the end.
Health-related food taxes	World Cancer Research Fund International NOURISHING policy database	The Working Group focused on the ends / outcomes, policies are not considered outcome, but rather a means to the end.
Best-practice policy implemented for industrially produced trans-fatty acids (TFA) (Y/N)	World Health Organization - Noncommunicable Diseases Progress Monitor Member State has adopted	The Working Group focused on the ends / outcomes, policies are not considered outcome, but rather a means to the end.

	national policies to reduce population salt/sodium consumption	
Greenhouse gas emissions from food systems (farm to fork)	Crippa et al. (2021)[1]	Trade is yet adequately not accounted for in these aggregations, therefore it provides a rather misleading aggregated number.
Greenhouse gas emissions from food systems per capita (production-based emissions)	Crippa et al. (2021)[1]	Total greenhouse gas emissions were prioritized as per capita seems to imply that this also accounts for trade issues.
% change in soil organic carbon	Global Soil Organic Carbon Map	It is conceptually very important but data is not adequately there yet.
Biodiversity intactness	F. DeClerck et al. (2021) [2]	Biodiversity intactness is very important, but it is a society broad outcome indicator rather than food system specific.
Households with significant income from agriculture	LMICs: Harmonized Household Budget Surveys (LSMS type) - harmonized by FAO via RULiS<br>EU: Eurostat harmonized Labor Force Surveys<br>ILO: harmonized labor force surveys	RULiS data for LMICs had insufficient coverage. In addition, this indicator is less relevant outside LMICs.
% of rural population living below the poverty line	World Bank	Insufficient coverage
% population earning low pay	International labor force statistics - ILO	No urban/rural disaggregation available, so not very informative regarding agricultural workforce.
Informal employment rate in agriculture	International labor force statistics - ILO	Overlap with the underemployment rate.
Monthly wages for agricultural workers compared to the country's median monthly wage	World Bank	Lack of wage PPP data for inter-country comparability.
Coverage of school feeding programs	World Bank (2022). The Atlas of Social Protection: Indicators of Resilience and Equity (ASPIRE) Database.	Unclear definition of the indicator denominator, which appeared to include the total population rather than a population relevant for school-based programs (e.g. school-aged children).
Governance		
Constitutional recognition of the right to adequate food	National policy documents (as collected and analyzed by FAOLEX and UNHCHR) and International Treaty documents	Constitutional recognition is not the only type of legal recognition that would facilitate the governance conditions to exercise the Right to Food.
Policy coordination	Bertelsmann Transformation Index	
SDG 16.7.2: Proportion of population who believe decision-making is inclusive and responsive, by sex, age, disability and population group **	UN Custodial Agency: UNDP	There is no data yet, perhaps forthcoming?
SDG 16.7.1: Proportions of positions (by sex, age, persons with disabilities and population groups) in public institutions (national and local legislatures, public	UN Custodial Agency: UNDP (Partnering Agencies: UN Women)	There is no data yet, perhaps forthcoming?

service, and judiciary) compared to national distributions**		
Policy implementation	Bertelsmann Transformation Index	
Implementation of marketing of breast-milk substitutes restrictions (Fully achieved/ Partially achieved / Not achieved)	World Health Organization - Noncommunicable Diseases Progress Monitor	Breastfeeding is a nutrition-relevant care practice and may not be considered directly tied to food systems.
Voice and Accountability, WGI	World Bank, Worldwide Governance Indicators	It incorporates data from V-Dem Index and Open Budget Index so it is less nuanced, and could lead to duplication of indicators.
Resilience		
EM-DAT human impact	The database is made up of information from various sources, including UN agencies, governmental and non-governmental organizations, insurance companies, research institutes, and press agencies.	The ability of countries to better protect human life against shocks (e.g. cyclone, typhoons) has improved tremendously in the last 20 years thanks to investments in shelters and early warning systems, introducing a negative trend in long-term time series. If this indicator were to be used to proxy the exposure to shocks it would underestimate the actual level of exposure faced by countries in recent years.
SDG 16.1.2: Conflict-related deaths per 100,000 population, by sex, age, and cause.	Office of the High Commissioner for Human Rights (OHCHR) https://www.ohchr.org/en/instruments-and-mechanisms/human-rights-indicators/sdg-indicators-under-ohchrs-custodianship	Data is unavailable (only a very small number of countries seem to be collecting and making public this indicator). Some technical issues caused the Working Group to drop indicators that may be preferred by expert groups.
Economic value of violence	Institute for Economics and Peace	This indicator does not cover all types of shocks such as for instance natural disasters. Database is also not readily available.
People affected indicator	GCSI Monthly Dataset is uploaded to the ACAPS website and shared with INFORM partners on the last day of every month unless a new significant crisis occurs, in which case it may be updated ad hoc. The final scores and data are available in the ACAPS and the INFORM websites, uploaded in HDX, as well as through API.	Two indicators -- the economic impact and the people affected -- do not cover the same part of the world. The former covers low- and middle-income countries, while the latter covers high income countries.
FAO Relative detour cost (local impact)	Data available from University of Twente - ITC	New dataset that is tailored to food systems; however, there was uncertainty on how often it would be updated.
Road density (km of road per 100 sq. km of land area)	OpenStreetMap OSM, since road density indicator of the International Road Federation is under the private license. Open Street Map is open source and delivers better coverage.	The two global datasets available are not comparable due to difference in the methodology.
Poverty headcount ratio at $1.90 a day	World Bank	While poverty headcount could be considered as a possible resilience capacity indicator at household level, it was not considered a relevant indicator for food system resilience.

Access to electricity (% of population)	World Bank	With the exception of Sub-Sahara Africa and few countries in South Asia, most countries in the world now have electricity coverage over 85%, making this indicator not very useful or relevant at differentiating countries at a global level.
Renewable electricity output (% of total electricity output)	IEA statistics, World Bank	Renewable energy is not yet an appropriate global indicator as many countries still depend exclusively on fossil fuel-based energy.
Primary completion rate, total (% of relevant age group)	UNESCO, World Bank	While education could be considered a possible resilience capacity indicator at the household level, it was not considered a relevant indicator for food system resilience.
Social capital	BTI Transformation Index	A large number of Sub-Saharan Africa and all high income countries are not included in the BTI, making it less attractive as an indicator to track regional and global analyses than the Legatum Prosperity Index.
Global Innovation Index	Global Innovation Index	Although initially considered as a potential indicator of adaptation (one important dimension of resilience) the GII was not ranked as one of the top candidates by the different experts surveyed during the expert consultations. Additionally, the latest data available are from 2016.
Agrobiodiversity indicator 1: Species diversity in food supply	FAOSTAT food balance sheets	It is already included in the Dietary Sourcing and Flexibility Index, thus excluded to avoid double counting/bias.
Agrobiodiversity indicator 2: Species diversity in food production	FAOSTAT food balance sheets;	It is already included in the Dietary Sourcing and Flexibility Index, thus excluded to avoid double counting/bias.
Agrobiodiversity index	Jones et al. (2021)[3]	Combination of 22 indicators. Preference for non-aggregated indicators.
Stability of Food Consumption Score (FCS)	WFP curated Hunger Map https://hungermap.wfp.org	This indicator was excluded from the 'diets, food security and nutrition' domain as an insufficient measure of diet quality and therefore the stability thereof would not be an indicator of stability of diet quality.
Stability of Food Insecurity Experience Scale (FIES) - based indicators	FAO, MICS, DHS, World Bank high frequency phone surveys, country's own data	Was removed to avoid duplication or high cross-correlation with stability of the Food Consumption Score, however, that indicator was also ultimately excluded. The FIES (but not the stability of it) is included in the diet domain.

Table 2.2 Indicators excluded prior to expert and stakeholder consultations, with rationale for exclusion

Domain	Indicator name	Definition or description	Reason for elimination from consideration
Diets, Nutrition, & Health			
Diet Quality	Exclusive breastfeeding rate (children <6 months)	percentage of infants 0–5 months of age who were fed exclusively with breast milk during the previous day.	This is primarily a care practices indicator rather than a food systems indicator; outside the scope of the WG. Was excluded from Food Systems Dashboard diagnosis for this reason.
Diet Quality	Continued breastfeeding at 12-23 months	percentage of children 12–23 months of age who were fed breast milk during the previous day.	This is primarily a care practices indicator rather than a food systems indicator; outside the scope of the WG. Was excluded from FSD diagnosis for this reason.
Diet Quality	Minimum Meal Frequency for IYC (age 6-23 months)	percentage of children 6–23 months of age who consumed solid, semi-solid or soft foods (but also including milk feeds for non-breastfed children) at least the minimum number of times during the previous day.	This is primarily a care practices indicator rather than a food systems indicator; outside the scope of the WG. Was excluded from FSD diagnosis for this reason.
Diet Quality	Minimum Acceptable Diet for IYC (age 6-23 months)	percentage of children 6–23 months of age who consumed a minimum acceptable diet during the previous day. The minimum acceptable diet is defined as:<br>• for breastfed children: receiving at least the minimum dietary diversity and minimum meal frequency for their age during the previous day;<br>• for non-breastfed children: receiving at least the minimum dietary diversity and minimum meal frequency for their age during the previous day as well as at least two milk feeds.	This is primarily a care practices indicator rather than a food systems indicator; outside the scope of the WG. Was excluded from FSD diagnosis for this reason.
Diet Quality	Adolescent diet indicators	Various	Lack of geographic coverage.
Diet Quality	Healthy Eating Index	The Healthy Eating Index (HEI) is a measure of diet quality used to assess how well a set of foods aligns with key recommendations of the Dietary Guidelines for Americans. The Dietary Guidelines for Americans is designed for nutrition and health professionals to help individuals (ages 2 years and older) and families to consume a healthful and nutritionally adequate diet.	Not feasible; requires quantitative dietary intake data.
Diet Quality	Global Dietary Quality Score	The GDQS is a measure of diet quality with respect to both nutrient adequacy and diet-related NCD risk for use at the population level, with a range of 0-49. GDQS scores ≥23 are	Not feasible; requires quantitative or semi-quantitative dietary intake data.

Domain	Indicator name	Definition or description	Reason for elimination from consideration
		associated with a low risk of both nutrient adequacy and NCD risk, scores ≥15 and <23 indicate moderate risk, and scores <15 indicate high risk. Points are assigned based on 3 or 4 categories of quantitative amounts (defined in g/d) specific to each group.	
Diet Quality	Percent of dietary energy from ultraprocessed foods	Percent of dietary energy from ultraprocessed foods, using the NOVA classification.	Not feasible; requires quantitative dietary intake data.
Diet Quality	Sweet foods consumption	The percent of the population age 15+ that consumed any sweet food in the previous day or night. This is a negative indicator.	Captured in the more aggregated indicator "Unhealthy food consumption".
Diet Quality	Salty or fried snacks consumption	The percent of the population age 15+ that consumed any salty or fried snack in the previous day or night. This is a negative indicator.	Captured in the more aggregated indicator "Unhealthy food consumption".
Diet Quality	Vegetable consumption	Prevalence of the population including vegetables as part of the diet, which is a global recommendation.	Captured in the more aggregated indicator "zero fruit and vegetable consumption".
Diet Quality	Fruit consumption	Prevalence of the population including fruits as part of the diet, which is a global recommendation.	Captured in the more aggregated indicator "zero fruit and vegetable consumption".
Diet Quality	WHO-FV	A score of 3 or more indicates likelihood of consuming at least 400g fruits and vegetables, which is a global dietary recommendation. Based on food group consumption data collected in the DQQ. *Provisional indicator; cutoff not yet globally validated.	Not yet globally validated. Possible to reconsider in future years; the indicator is constructed from the DQQ, which is collected in the Gallup World Poll.
Diet Quality	WHO-Sugar	A score of 2 or more indicates likelihood of exceeding 10% of dietary energy from free sugars, which is a negative indicator; limiting free sugar consumption to <10% of dietary energy is a global dietary recommendation. Based on food group consumption data collected in the DQQ. *Provisional indicator; cutoff not yet globally validated.	Not yet globally validated. Possible to reconsider in future years; the indicator is constructed from the DQQ, which is collected in the Gallup World Poll.
Diet Quality	WHO-Fiber	A score of 4 or more indicates likelihood of consuming at least 25g fiber, which is a global dietary recommendation. Based on food group consumption data collected in the DQQ. *Provisional indicator; cutoff not yet globally validated.	Not yet globally validated. Possible to reconsider in future years; the indicator is constructed from the DQQ, which is collected in the Gallup World Poll.

Domain	Indicator name	Definition or description	Reason for elimination from consideration
Diet Quality	WHO-SatFat	A score of 2 or more indicates likelihood of exceeding 10% of dietary energy from saturated fat, which is a negative indicator; limiting saturated fat consumption to <10% of dietary energy is a global dietary recommendation. Based on food group consumption data collected in the DQQ. *Provisional indicator; cutoff not yet globally validated.	Not yet globally validated. Possible to reconsider in future years; the indicator is constructed from the DQQ, which is collected in the Gallup World Poll.
Diet Quality	Global Burden of Disease - Estimated dietary intake for adults (g/day); available for: Fruit; Vegetables; Whole Grains; Legumes, Nuts, Seeds; Sugar-sweetened beverages; Milk; Red meat; Processed meat; Sodium; Calcium; Fiber; Poly-unsaturated fatty acids	Modeled estimate of food group intake per person, per day in each country.	Black box methodology. Beal et al. (2021)[4] showed that estimated intakes vary widely between estimation models, and as such may not be fit for advising policy.
Diet Quality	Global Dietary Database - Estimated dietary intake for adults (g/day); available for: Fruits, Non-starchy Vegetables, Potatoes, Other Starchy Vegetables, Beans and Legumes, Nuts and Seeds, Refined Grains, Whole Grains, Unprocessed Red Meats, Total Processed Meats, Total Seafoods, Eggs, Cheese, Yogurt (including fermented milk)	Modeled estimate of food group intake per person, per day in each country.	
Food security	Affordability of a healthy diet (as compared to Intl Poverty Line)	The ratio of the cost of an energy sufficient diet to the food poverty line (63% of the international poverty line of 1.90/day in 2017 USD).	Duplicative of other affordability indicators that are more informative; the indicator inherently critiques the denominator (i.e., highlights the limitations of food poverty line construction), suggesting that the denominator is not a solid standard for comparison.
Food security	Cost of nutrient adequacy (CoNA) relative to average food expenditure	The ratio of the cost of a nutrient adequate diet to observed per capita food expenditures from national accounts. CoNA is the cost of the least expensive locally available foods to meet average requirements for dietary energy and 23 essential macro- and micro-nutrients, per capita, per day (2017USD).	Duplicative/confusing alongside Cost and affordability of a Healthy Diet indicator.
Food environments	Relative cost of fruits and vegetables	The ratio of the cost of fruits and vegetables to the cost of starchy staples, each in the amount required to satisfy average daily recommendations from FBDG.	Overly disaggregated compared to the main indicators, cost and affordability of a healthy diet.
Food environments	Relative cost of legumes, nuts and seeds	The ratio of the cost of legumes, nuts and seeds to the cost of starchy staples, each in the amount required to satisfy average daily recommendations from FBDG.	Overly disaggregated compared to the main indicators, cost and affordability of a healthy diet.

Domain	Indicator name	Definition or description	Reason for elimination from consideration
Food environments	Relative cost of ASF	The ratio of the cost of animal-source foods to the cost of starchy staples, each in the amount required to satisfy average daily recommendations from FBDG.	Overly disaggregated compared to the main indicators, cost and affordability of a healthy diet.
Food environments	Relative cost of oils and fats	The ratio of the cost of oils and fats to the cost of starchy staples, each in the amount required to satisfy average daily recommendations from FBDG.	Overly disaggregated compared to the main indicators, cost and affordability of a healthy diet.
Food environments	Relative caloric price – available for the following food groups: Cereals, Fats and oils, Eggs, Nuts, Pulses, Milk, Fish, Meat, Dark green leafy vegetables, Vitamin A-rich fruits, Other vegetables, Other fruit, Salty snacks, Sugary snacks, SSBs	The ratio of a calorie of the specified food group to a calorie of cereals.	Not currently routinely calculated; feasibility may be limited.
Food environments	Per capita dietary energy supply (kcal/person/day)	kcal per person per day available in a country's foods supply.	Information is better captured in PoU; duplicative.
Food environments	Availability/supply (g/day/capita) – Available for: Meat, Fish, Eggs, and Milk	Grams per person per day of each meat, fish, eggs, and milk available in a country's food supply.	Information is better captured in aggregate indicator "Availability/supply of animal-source foods". There is no requirement or recommendations for this item specifically; rather recommendations are set for a more aggregated food group. Because of the absence of item-specific recommendations, this indicator is difficult to interpret.
Food environments	Food supply adequacy	This indicator estimates to what extent the national food supply is adequately meeting the daily requirements per person (g/capita/day) of the food groups recommended for a healthy diet (fruits, vegetables, pulses, animal-source foods).	Indicator not clearly defined / established. Possible to reconsider in future years.
Food environments	Household availability (unit/day/HH equivalent) of macro- and micronutrients		Duplicative of availability at individual level (this indicator is the same indicator estimated at household level, which requires additional assumptions).
Food environments	Global demand for instant noodles	Interesting indicator of UFP in the food environment. Many but not all countries in the database.	Black box methodology.
Food environments	Retail value of packaged food sales per capita (also available as growth rate)		Unclear interpretation. Packaged food may be healthy -- like canned beans -- or unhealthy. Unhealthy packaged food is better captured by retail value of UPF sales per capita.
Food environments	Modern grocery retailers per 100K people (also available as growth rate)		Unclear interpretation.
Food environments	Supermarkets per 100K people (also available as growth rate)		Unclear interpretation.

Domain	Indicator name	Definition or description	Reason for elimination from consideration
Food environments	Yields (tonnes/hectare) of cereals		Outside the scope for the diets, nutrition and health theme.
Food environments	Yields (tonnes/hectare) of vegetables		Outside the scope for the diets, nutrition and health theme.
Food environments	Losses of nutritious foods as pct of domestic supply; fruits		Outside the scope for the diets, nutrition and health theme. Does not indicate where along supply chain loss occurs.
Food environments	Losses of nutritious foods as pct of domestic supply; vegetables		Outside the scope for the diets, nutrition and health theme. Does not indicate where along supply chain loss occurs.
Food environments	Losses of nutritious foods as pct of domestic supply; pulses		Outside the scope for the diets, nutrition and health theme. Does not indicate where along supply chain loss occurs.
Food environments	Losses of nutritious foods as pct of domestic supply; cereals		Outside the scope for the diets, nutrition and health theme. Does not indicate where along supply chain loss occurs.
Policies affecting food environments	Food safety standards	Undefined	Indicators not defined. Possible to reconsider in future years.
Policies affecting food environments	Availability of food composition table (binary)	The country has a country-specific food composition table (1/0).	Unclear interpretation; unclear need for country specific food composition table for every country in the world.
Policies affecting food environments	Availability of food based dietary guidelines (binary)	The country has developed national food-based dietary guidelines (FBDG).	Unclear interpretation.
Policies affecting food environments	Fortification legislation, rice - Voluntary / Mandatory / None	The data may not exist in the form as disaggregated per food item (perhaps, salt is the most relevant one). May use the structure they exist in the database (fortification data site).	Overly disaggregated compared to the main indicator, presence of any fortification legislation.
Policies affecting food environments	Fortification legislation, maize flour - Voluntary / Mandatory / None	The data may not exist in the form as disaggregated per food item (perhaps, salt is the most relevant one). May use the structure they exist in the database (fortification data site).	Overly disaggregated compared to the main indicator, presence of any fortification legislation.
Policies affecting food environments	Fortification legislation, wheat flour - Voluntary / Mandatory / None	The data may not exist in the form as disaggregated per food item (perhaps, salt is the most relevant one). May use the structure they exist in the database (fortification data site).	Overly disaggregated compared to the main indicator, presence of any fortification legislation.
Policies affecting food environments	Fortification legislation, oil - Voluntary / Mandatory / None	The data may not exist in the form as disaggregated per food item (perhaps, salt is the most relevant one). May use the structure they exist in the database (fortification data site).	Overly disaggregated compared to the main indicator, presence of any fortification legislation.

Domain	Indicator name	Definition or description	Reason for elimination from consideration
Policies affecting food environments	Fortification legislation, salt- Voluntary / Mandatory / None	The data may not exist in the form as disaggregated per food item (perhaps, salt is the most relevant one). May use the structure they exist in the database (fortification data site).	Overly disaggregated compared to the main indicator, presence of any fortification legislation.
Policies affecting food environments	Biofortification policies and programs (binary: adopted/in process versus none)	Countries where biofortification is included in government legislation/programs/policies/strategies.	Not globally relevant; Lack of geographic coverage (additional note: conveys limited information and only 'yes' for a few countries in the Food Systems Dashboard: HarvestPlus made detailed policy (including information on which policies are in which country) and crops released databases available online. However, it does not meet the criteria to have at least 70 countries of coverage.)
Policies affecting food environments	Diet related (advertising, labelling, restrictions on trans fat, sodium etc.)	Unclear definition; topic rather than indicator.	Many indicators, but geographic coverage is unclear - also unclear if database is regularly updated.
Policies affecting food environments	Availability of legislation on nutrition labelling		Precise indicator definition and Feasibility needs to be verified.
Policies affecting food environments	Availability of legislation on ingredient labelling on packaged foods		Precise indicator definition and Feasibility needs to be verified.
Policies affecting food environments	Trans fat ban in place (binary? Voluntary / Mandatory / None?)		Duplicative of other indicator on implementation of trans fat legislation.
Policies affecting food environments	COMP 1: Food composition targets/standards have been established for processed foods by the government for the content of the nutrients of concern in certain foods or food groups if they are major contributors to population intakes of these nutrients of concern (trans fats and added sugars in processed foods, salt in bread salt in snacks etc.))		Not feasible; Lack of geographic coverage (additional note: not monitorable across the countries).
Policies affecting food environments	COMP 2: Food composition targets/standards have been established by the government for out-of-home meals in food service outlets (such as fast food joints, food kiosks, check-check joints, restaurants, and other local food vendors) for the content of the nutrients of concern in certain foods or food groups if they are major contributors to population intakes of these nutrients of concern (e.g. trans fats, added sugars, salt, saturated fat, saturated fat in commercial frying fats/oils)		Not feasible; Lack of geographic coverage (additional note: not monitorable across the countries).
Policies affecting food environments	LABEL1: Ingredient lists and nutrient declarations in line with Codex recommendations are present on the labels of packaged foods		Not feasible; Lack of geographic coverage (additional note: not monitorable across the countries).
Policies affecting food environments	LABEL2: Robust, evidence-based regulatory systems are in place for approving/reviewing claims on foods, so that consumers are protected against unsubstantiated and misleading nutrition and health claims		Not feasible; Lack of geographic coverage (additional note: not monitorable across the countries).
Policies affecting food environments	LABEL3: A single, consistent, interpretive, evidence-informed front-of-pack supplementary nutrition information system, which readily allows consumers to assess a product's healthiness, is applied to packaged foods		Not feasible; Lack of geographic coverage (additional note: not monitorable across the countries).

Domain	Indicator name	Definition or description	Reason for elimination from consideration
Policies affecting food environments	LABEL4: A consistent, single, simple, clearly-visible system of labelling the menu boards of quick service restaurants (i.e. fast food chains) is applied by the government, which allows consumers to interpret the nutrient quality and energy content of foods and meals on sale		Not feasible; Lack of geographic coverage (additional note: not monitorable across the countries).
Policies affecting food environments	PROMO1: Effective policies are implemented by the government to restrict exposure and power of promotion of unhealthy foods to or for children through broadcast media (TV, radio)		Not feasible; Lack of geographic coverage (additional note: not monitorable across the countries).
Policies affecting food environments	PROMO2: Effective policies are implemented by the government to restrict exposure and power of promotion of unhealthy foods to or for children through non-broadcast media (e.g. Internet, social media, food packaging, sponsorship, religious events, outdoor advertising including around schools)		Not feasible; Lack of geographic coverage (additional note: not monitorable across the countries).
Policies affecting food environments	PROMO3: Effective policies are implemented by the government to ensure that unhealthy foods are not commercially promoted to or for children in settings where children gather (e.g. preschools, schools, sport and cultural events)		Not feasible; Lack of geographic coverage (additional note: not monitorable across the countries).
Policies affecting food environments	PROMO4: Effective policies are implemented by the government to restrict the marketing of breastmilk substitutes		Not feasible; Lack of geographic coverage (additional note: not monitorable across the countries).
Policies affecting food environments	PRICES1: Taxes or levies on healthy foods are minimized to encourage healthy food choices where possible (e.g. low or no sales tax, excise, value-added or import duties on fruit and vegetables)		Not feasible; Lack of geographic coverage (additional note: not monitorable across the countries).
Policies affecting food environments	PRICES2: Taxes or levies on unhealthy foods (e.g. sugar-sweetened beverages, foods high in nutrients of concern) are in place and increase the retail prices of these foods by at least 10% to discourage unhealthy food choices where possible, and these taxes are reinvested to improve population health		Not feasible; Lack of geographic coverage (additional note: not monitorable across the countries).
Policies affecting food environments	PRICES3: The intent of existing subsidies on foods, including infrastructure funding support (e.g. research and development, supporting markets or transport systems), is to favour healthy rather than unhealthy foods		Not feasible; Lack of geographic coverage (additional note: not monitorable across the countries).
Policies affecting food environments	PRICES4: The government ensures that food-related income support programs are for healthy foods		Not feasible; Lack of geographic coverage (additional note: not monitorable across the countries).
Policies affecting food environments	PROV1: The government ensures that there are clear, consistent policies (including nutrition standards) implemented in schools and early childhood education services for food service activities (canteens, food at events, fundraising, promotions, vending machines etc.) to provide and promote healthy food choices		Not feasible; Lack of geographic coverage (additional note: not monitorable across the countries).
Policies affecting food environments	PROV2: The government ensures that there are clear, consistent policies in other public sector settings for food service activities (canteens, hospitals, clinics, food at events, fundraising, promotions, vending machines, public procurement standards etc.) to provide and promote healthy food choices.		Not feasible; Lack of geographic coverage (additional note: not monitorable across the countries).
Policies affecting food environments	PROV3: The Government ensures that there are good support and training systems to help schools and other public sector organisations and their caterers meet the healthy food service policies and guidelines		Not feasible; Lack of geographic coverage (additional note: not monitorable across the countries).

Domain	Indicator name	Definition or description	Reason for elimination from consideration
Policies affecting food environments	RETAIL1: Zoning laws and policies are robust enough and are being used, where needed, by local governments to place limits on the density or placement of quick serve restaurants or other outlets selling mainly unhealthy foods in communities, and to encourage the availability of outlets selling healthy options such as fresh fruit and vegetables		Not feasible; Lack of geographic coverage (additional note: not monitorable across the countries).
Policies affecting food environments	RETAIL2: The Government ensures existing support systems are in place to encourage food stores and food service outlets to promote the availability of healthy foods and to limit the promotion and availability of unhealthy foods		Not feasible; Lack of geographic coverage (additional note: not monitorable across the countries).
Policies affecting food environments	RETAIL 3: Food hygiene policies are robust enough and are being enforced, where needed, by national and local government to protect human health and consumers' interests in relation to food.		Not feasible; Lack of geographic coverage (additional note: not monitorable across the countries).
Policies affecting food environments	TRADE1: The Government undertakes risk impact assessments before and during the negotiation of trade and investment agreements, to identify, evaluate and minimize the direct and indirect negative impacts of such agreements on population nutrition and health		Not feasible; Lack of geographic coverage (additional note: not monitorable across the countries).
Policies affecting food environments	TRADE2: The government adopts measures to manage investment and protect their regulatory capacity with respect to public health nutrition		Not feasible; Lack of geographic coverage (additional note: not monitorable across the countries).
Policies affecting food environments	Implementation of salt/sodium policies (Fully achieved/ Partially achieved / Not achieved)	This indicator is about sodium restriction, and is not about salt iodization; that is covered by other indicators on fortification legislation and iodized salt coverage. Country has implemented policies restricting salt/sodium in sold food products.	Lack of coverage - Could not find this indicator in the WHO Global Health Observatory database https://www.who.int/data/gho/data/indicators even though a TF team member indicated "World Health Organization - Noncommunicable Diseases Progress Monitor Member State has adopted national policies to reduce population salt/sodium consumption".
Environment, Natural Resources, & Production			
GHG	Per capita biodiversity impact of food consumption (sp.yr*10^12)	The extinction rate expected under current food consumption patterns. These biodiversity losses result from the occupation of farmland and the effects of transformation of natural ecosystems into farmland. Food consumption is estimated using FAO Food Balance Sheet data as a proxy.	Too many parameter assumptions in calculating these per capita per country.
GHG	Per capita GHG emissions of food consumption (kg Co2Eq)	Greenhouse gas emissions (measured in carbon dioxide equivalent) related to current food consumption patterns. Food consumption is estimated using FAO Food Balance Sheet data as a proxy.	Too many parameter assumptions in calculating these per capita per country.
Water	Per capita eutrophication of food consumption (gPO43 eq)	The increase in phosphorous and nitrogen concentration (measured in phosphate equivalent) in water and soils as a result of current food consumption patterns. Food consumption is estimated using FAO Food Balance Sheet data as a proxy.	Too many parameter assumptions in calculating these per capita per country.

Domain	Indicator name	Definition or description	Reason for elimination from consideration
Water	Per capita water use linked to food consumption (L)	Freshwater withdrawals needed to produce food under current food consumption patterns. Includes irrigation water (for crops and livestock feed), animal drinking water, and water used during food processing. Withdrawals are weighted by local water scarcity. Food consumption is estimated using FAO Food Balance Sheets data as a proxy.	Too many parameter assumptions in calculating these per capita per country.
Water	Per capita water scarcity of food consumption (L eq)	Indicator undefined.	Too many parameter assumptions in calculating these per capita per country.
Biosphere integrity	Total Ecological Footprint of consumption [global ha]	The ecological footprint of production plus the ecological footprint of imports and minus the footprint of exports.	Too many parameter assumptions in calculating these per capita per country.
Biosphere integrity	Number of Earths required	Number of Earths required to support human's footprint if everyone lives like an average individual or country.	Too aggregated and too many assumptions.
Land & soils	Nitrogen fertilizer use per unit of land (tonnes ha-1)	Totals in nitrogen (N) for agriculture use of inorganic (chemical or mineral) fertilizers. Both straight and compound fertilizers are included.	Not normative - very context specific.
Land & soils	Phosphorous fertilizer use per unit of land (tonnes ha-1)	Totals in phosphorus (expressed as P2O5) for agriculture use of inorganic (chemical or mineral) fertilizers. Both straight and compound fertilizers are included.	Not normative - very context specific.
Land & soils	Agricultural land as % of arable land	Agricultural land includes arable land, land under permanent crops, and meadows or pasture. This indicator offers a quick snapshot of how much of the land area per country is occupied with agriculture.	Not normative - very context specific.
Land & soils	Percentage of intact area (% per country)	Intact land areas are those with low levels of human impact or disturbance, such that natural processes predominate.	Not necessarily normative and broader than food systems.
Land & soils	Soil Organic Content (tonnes ha-1)	Average soil organic carbon (SOC). Organic carbon is a major component of the organic material present in soils. It stabilizes soil structure and reduces erosion, improves soil fertility, and enhances its water-holding capacity.	Dependent on agroecological setting - replaced by change in SOC.
Biosphere integrity	% Natural vegetation in agricultural land	Cropland with a minimum level of proximate natural and seminatural vegetation to maintain ecosystem integrity.	Included a similar indicator "Functional Integrity (% agricultural land with minimum level of natural habitat)".
Biosphere integrity	Crop richness (average number of crops per unit of land)	Crop richness in production systems.	Replaced by production diversity metric as part of resilience theme.

Domain	Indicator name	Definition or description	Reason for elimination from consideration
Biosphere integrity	Average Shannon diversity crops (dimensionless)	The Shannon diversity index reflects how many different types of foods (crops and livestock) are produced in a certain country, and how evenly these different types are distributed. This indicator identifies the diversity of crops in a country without regard for the nutrient content of each crop.	See resilience group.
Biosphere integrity	Calories diversity measured by Shannon index	How many different types of food items there are in a certain country, and how evenly these different types are distributed.[5]	See resilience group.
Biosphere integrity	Tree cover on agricultural land (%)	Average percentage tree cover on agricultural land in 2010.	Not normative - very context and crop specific.
Biosphere integrity	Benefits of biodiversity index (0= no biodiversity potential to 100 maximum)		Too aggregated.
Biosphere integrity	Integrated plant nutrient management	Concept not an indicator.	Replaced by sustainable nitrogen management index.
Biosphere integrity	Total Ecological Footprint of production (global ha)	Combined land appropriation for producing agricultural, livestock, fishery and aquaculture, and timber products as well as the CO2 emissions and built-up surfaces (e.g. roads, factories, cities) linked to that production.	Too aggregated and too much a black box.
Biosphere integrity	Environmental Performance Index	Composite index of 37 indicators for measuring countries' environmental performance regarding environmental health (i.e. air quality, sanitation and drinking water, heavy metals and waste management) and ecosystem vitality (e.g. biodiversity and habitat, ecosystem services, fisheries, water resources, climate change, pollution emissions, agriculture).	Too aggregated.
GHG	Global Climate Risk Index	The Global Climate Risk Index assess the extent to which countries have been affected (in terms of fatalities and economic losses) by meteorological events such as tropical storms, winter storms, severe weather, hail, tornados, local storms; hydrological events such as storm surges, river floods, flash floods, mass movement (landslide); climatological events such as freezing, wildfires, droughts, and cold/heat waves.	Too aggregated and also a driver, not just an outcome.
GHG	Climate Risk fatalities per 100,000 (1999 – 2018)		More a vulnerability indicator than an environmental indicator.
GHG	Climate Risk $ loss in million USD (1999 – 2018) (or per unit of GDP)		More a vulnerability indicator than an environmental indicator.

Domain	Indicator name	Definition or description	Reason for elimination from consideration
Land & soils	Soil erosion	Concept not an indicator.	Not updated sufficiently and aggregated indicator.
Land & soils	Soil biodiversity threats index	Average levels of potential risk to soil biodiversity and life at a global scale. Considered threats include loss of above ground biodiversity, pollution and nutrient overloading, cropland percentage cover, cattle density, fire density between 1997-2010 , Water and Wind Erosion Vulnerability Indices, Desertification Vulnerability Index, and Global Aridity Index.	Not updated sufficiently and aggregated indicator.
Land & soils	Soil biodiversity potential index	Average Soil Biodiversity Potential Index describes the potential level of diversity (micro and macro fauna) living in soils on our planet.	Not updated sufficiently and aggregated indicator.
Pollution	(Change in) fertilizer use (tonnes per year)		Not sufficiently normative - context specific.
Land & soils	Tree cover loss (%)		Considered as part of agricultural land use change.
Land & soils	Wetland loss (%)		Considered as part of agricultural land use change.
Land & soils	Grassland loss (%)		Considered as part of agricultural land use change.
Land & Soil	% of agricultural land degraded		No frequent updates.
Water	Water stress (SDG 6.4.2)	Level of water stress: freshwater withdrawal as a proportion of available freshwater resources.	Redundant with % withdrawal by agriculture.
Pollution	Trend in Chemical Production	Does not currently exist and would have to be created.	Not directly related to impact.
Biosphere integrity	Human trophic level	Trophic levels are critical for synthesizing species' diets, depicting energy pathways, understanding food web dynamics and ecosystem functioning, and monitoring ecosystem health. Specifically, trophic levels describe the position of species in a food web, from primary producers to apex predators (range, 1–5). Small differences in trophic level can reflect large differences in diet. Developed by Bonhommeau et al (2013)[6].	Not validated.
Livelihoods, Poverty, & Equity			
Income and poverty	Share of agricultural income from fisheries	Agricultural income from fishery activities as a share of total income (%).	Disaggregated data by sub-sector to identify fishery-specific data are not available in harmonized global database. De novo calculation from microdata is not feasible at present.

Domain	Indicator name	Definition or description	Reason for elimination from consideration
Income and poverty	Agricultural income (livestock, crop, fishery, forestry, ag wage), share of total income (%)	Household income from all agricultural activities, as a share of total household income.	Data are only available for 64 low- and lower-middle income countries. There are no comparable data from countries at other income levels to combine with these to meet the prerequisite requirement for global coverage.
Income and poverty	Proportion of the rural population living below the international poverty line	The indicator "proportion of the population below the international poverty line" is defined as the percentage of the population living on less than $1.90 a day at 2011 international prices.	Data are not presently available. When the data for the SDG indicator 1.1.1 become available for urban/rural disaggregation, we can calculate the share of the rural population living below this line (by combining with population data).
Employment	Frequency rate of occupational injuries		Insufficient disaggregation to link to food systems.
Employment	Farmer age	Average age of farmers per nation.	Closest proxy indicators are "Average age of self-employed or unpaid workers, aged 15-64, rural" and "Average age of wage workers, aged 15-64, rural", however these data have insufficient coverage across countries and country income levels.
Employment	Farm labour force - family labour	Family workers are persons who help another member of the family to run an agricultural holding or other business, provided they are not considered as employees. Persons working in a family business or on a family farm without pay should be living in the same household as the owner of the business or farm, or in a slightly broader interpretation, in a house located on the same plot of land and with common household interests.	Insufficient data coverage across countries and country income levels.
Governance			
Effective Implementation	Representation and influence of rural organizations and rural people, IFAD Rural Sector Performance Assessments	Representation and influence of rural organizations and rural people. Scale from 1 (worst) to 6 (best); Annual until 2014 and thereafter only conducted every 3 years; Targeted at LMICs and based on subjective assessments of IFAD country economists and then centrally reviewed.	The assessment approach seems to be a "black box"; data collected only every 3 years.
Accountability	Healthy Food Environment Policy Index (Food-EPI) developed by International Network for Food and Obesity/Non-communicable Diseases Research, Monitoring and Action Support (INFORMAS), Swinburn et al (2013).[7]	Food-EPI is comprised of 7 policy domains related to food environments and 6 infrastructure domains: leadership, governance, funding and resources, monitoring and intelligence, platforms for interaction and health-in-all-policies. Each domain is specified	It only covers 49 countries.

Domain	Indicator name	Definition or description	Reason for elimination from consideration
		by several good practice indicators (47 in total) that encompass the directions necessary to improve the healthiness of food environments and to help prevent obesity and diet-related NCDs.	
Effective Implementation	World Benchmarking Alliance (WBA) Food and Agriculture Benchmark		Data is at the level of companies rather than countries.
Effective Implementation	CAADP's Agricultural Transformation Scorecards (Department of Rural Economy and Agriculture, African Union, 2020)		Only available for Africa.
Shared Vision	Hunger and Nutrition Commitment Index (HANCI)		No longer being collected.
Strategic planning	WHO Landscape Analysis (WHO, 2012)	It is a tools package for in-depth country assessment that consist of stakeholder mapping tool, interview questionnaires, and analytical framework to do analysis of a country's capacities and resources, and identifies promising actions that could be scaled up to improve nutrition.	Seems to have only been done once (in 2012) and only covers a handful of countries.
Effective Implementation	WHO Global database on the Implementation of Nutrition Action (GINA)	GINA provides a repository of policies, actions and mechanisms related to nutrition. It is an interactive platform for sharing standardized information on nutrition policies and action, i.e. what are the commitments made and who is doing what, where, when, why and how (including lessons learnt).	Great resources on policy actions but it would need to be consolidated into quantifiable indicators.
Accountability	Access To Nutrition Index (ATNI)	The Global Access to Nutrition Index focusses on the role that food and beverage manufacturers play in making healthy food affordable and accessible to all consumers globally.	Focuses on companies, not countries. It does not seem to have been updated since 2018.
Accountability	Monitoring, Evaluation, Accountability, and Learning (MEAL), Scaling Up Nutrition (SUN)	Tracks progress towards overall SUN movement objectives and enforces mutual accountability. Relies on review of multi-stakeholder joint assessments, national budget analysis, national nutrition action plans, subnational action mapping, etc.	Not clear that this is still collected. Last update was 2018/2019. Also does not meet coverage requirement.
Resilience			
Resilience capacities	Positive peace index	The Positive Peace Index measures the level of societal resilience of a nation or region.	Black box, composite indicator.
Resilience capacities	Social trust	Based on responses to questions that include trust in various group, trust in other people confidence in police, courts, govt, etc.	Data available for only 57 countries.

Domain	Indicator name	Definition or description	Reason for elimination from consideration
Resilience responses/strategies	Agricultural irrigated land (% of total agricultural land)	Agricultural irrigated land refers to agricultural areas purposely provided with water, including land irrigated by controlled flooding.	Using irrigation for some countries does not make sense because they don't need it for their agriculture as much as others (e.g. North Europe vs South Europe), so irrigation is not a globally valid indicator. In Bene et al 2019[8] the following exclusion criterion was used: "Global validity. Were excluded indicators that refer to processes that are specific to some specific regions of the world and not to others. For instance, “Percentage of agricultural land lost yearly to desertification” is excluded as desertification is a phenomenon that by definition can only occur in some specific regions of the world" - similarly the use of irrigation is not globally valid.

References

1. Crippa, M. *et al.* Food systems are responsible for a third of global anthropogenic GHG emissions. *Nature Food* **2**, 198–209 (2021).

2. DeClerck, F., Jones, S., Estrada-Carmona, N. & Fremier, A. Spare half, share the rest: A revised planetary boundary for biodiversity intactness and integrity. (2021) doi:10.21203/rs.3.rs-355772/v1.

3. Jones, S. K. *et al.* Agrobiodiversity Index scores show agrobiodiversity is underutilized in national food systems. *Nature Food* 1–12 (2021).

4. Beal, T., Herforth, A., Sundberg, S., Hess, S. Y. & Neufeld, L. M. Differences in modelled estimates of global dietary intake. *Lancet* **397**, 1708–1709 (2021).

5. Remans, R., Wood, S. A., Saha, N., Anderman, T. L. & DeFries, R. S. Measuring nutritional diversity of national food supplies. *Global Food Security* **3**, 174–182 (2014).

6. Bonhommeau, S. *et al.* Eating up the world's food web and the human trophic level. *Proc. Natl. Acad. Sci. U. S. A.* **110**, 20617–20620 (2013).

7. Swinburn, B. *et al.* Monitoring and benchmarking government policies and actions to improve the healthiness of food environments: a proposed Government Healthy Food Environment Policy Index. *Obes. Rev.* **14 Suppl 1**, 24–37 (2013).

8. Béné, C., Prager, S.D., Achicanoy, H.A.E. et al. Global map and indicators of food system sustainability. *Sci Data* 6, 279 (2019).

Supplementary Information, Appendix 3: Consultation Reports

This appendix contains the reports of the expert and stakeholder consultations used in the process of selecting indicators.

Contents

Expert consultation report…………………………………………………………………………2

Regional policy stakeholder consultation reports, by region…………………………………….15

Expert Consultation Report

The Food Systems Countdown Initiative (FSCI) 2030 aims to track food systems transformation until 2030 and beyond. This tracking is done by means of a set of indicators applicable across various themes and geographies.

In order to develop a list of potential candidate indicator, the initiative undertook a process of consultations with various stakeholders.

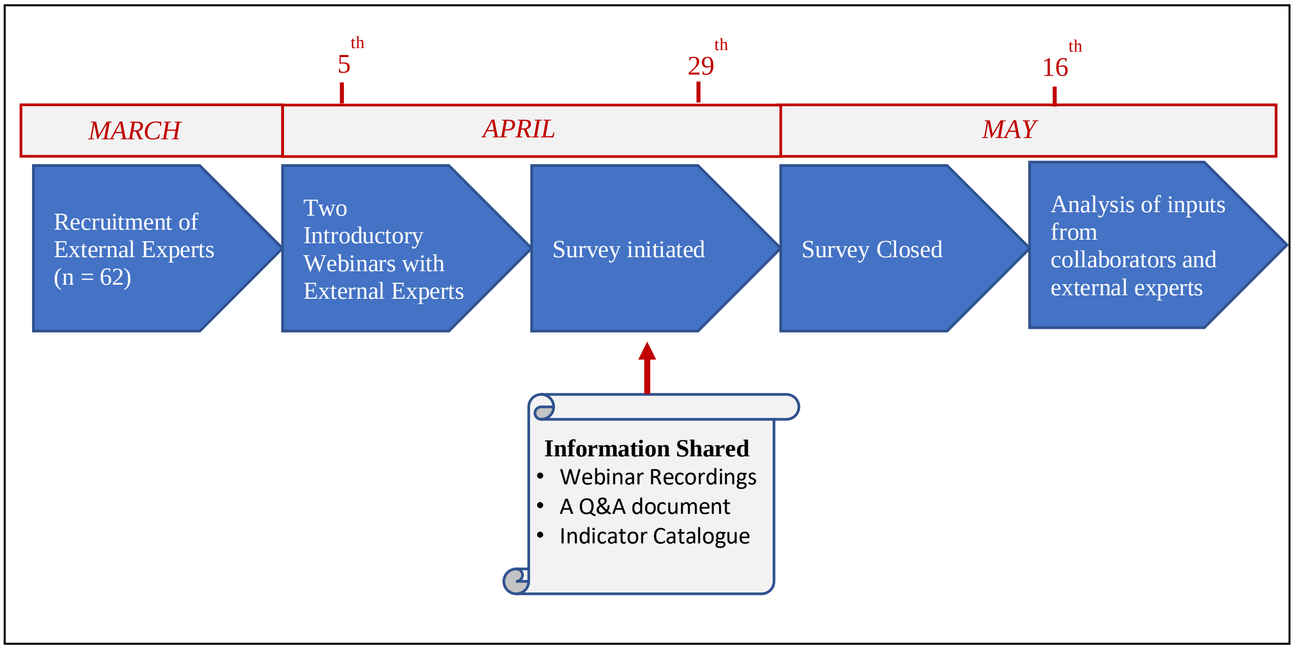

Timeline for the expert consultation process

The consultation process to finalize the list of indicators involved two groups of experts. One group comprised the collaborators—involved in the FSCI work from the beginning—who developed the long list of candidate indicators. The other group consisted of external experts who were recruited from a variety of networks and organizations. It was made sure that there was a diverse group of outside experts who represented various geographies, subject areas, and levels of experience.

During February and March 2022, 173 individuals were invited to join the external expert group; 62 of them replied and confirmed their attendance. They took part in the initial webinar and discussions on the FSCI indicator selection process. However, only 28 of the 62 external experts completed the indicator scoring survey. In addition, from the core collaborators group 39 completed the survey, thus a total of 67 individuals scored the FSCI indicators.

Two introductory webinars were held in the beginning of April 2022 to introduce the FSCI 2030 to external experts and describe the survey and indicator scoring criteria. After the webinar, the external experts received the scoring survey, webinar recordings, presentations, a document with questions and answers, and an indicator catalogue. The survey was closed Mid-May 2022, results were analyzed and presented to the FSCI core collaborator group.

Composition of External Experts who confirmed their participation in the consultation.

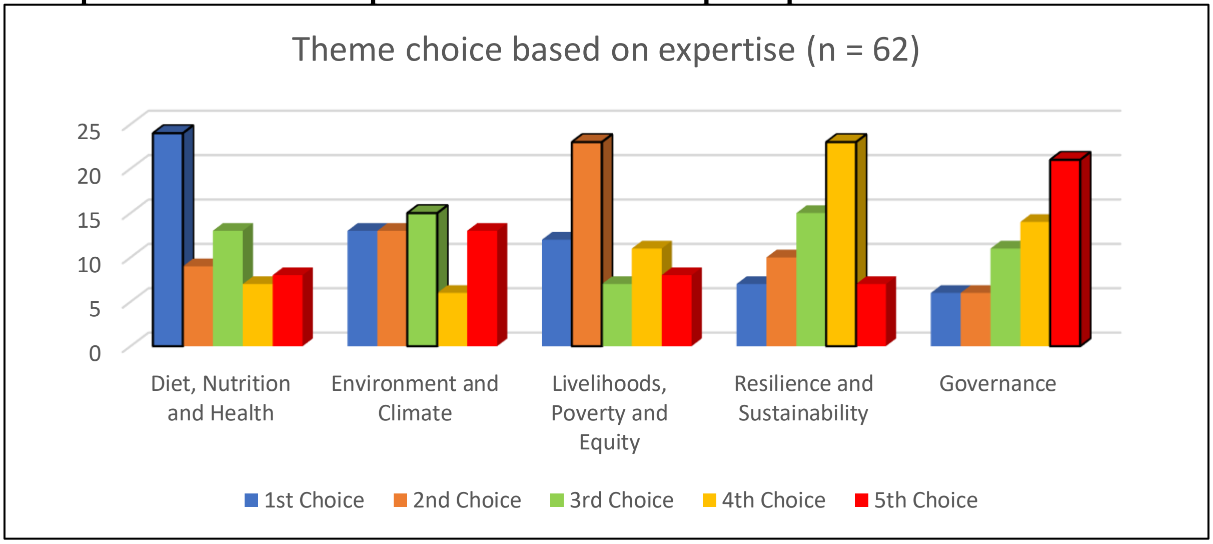

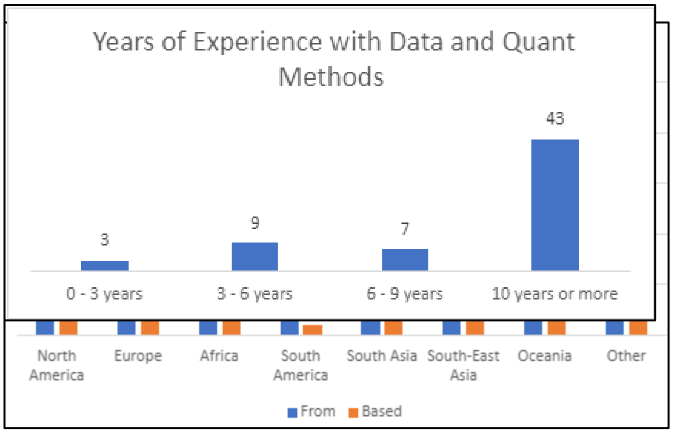

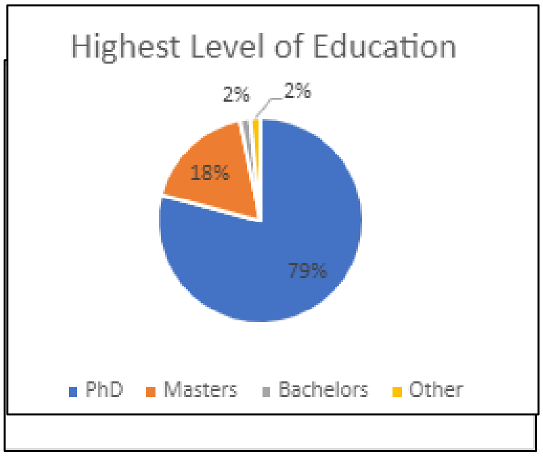

The Indicator Scoring Survey Instrument

The indicator scoring survey was created using the online platform KOBOToolbox. The survey included 98 indicators spread across five themes and 23 domains. The breakdown of the number of indicators in each domain under the five themes is shown in the table below.

Theme and Domains	Number of Indicators
**DIETS, NUTRITION, AND HEALTH**	
Diet Quality	19
Food Security	4
Food Environments	10
Policies Affecting Food Environments	8
**ENVIRONMENT AND CLIMATE**	
Greenhouse Gas Emissions	3
Land and Soil	2
Pollution	2
Biosphere Integrity	2
Biosphere Intactness	1
Water	1
**LIVELIHOODS, POVERTY, AND EQUITY**	
Income and Poverty	2
Employment	3
Social Protection	3
Rights	3
**GOVERNANCE**	
Shared Vision	2
Strategic Planning	1
Effective Implementation	3
Accountability	3
**RESILIENCE**	
Exposure to Shocks	5
Resilience Capacities	11
Agro- and Food Diversity	2
Resilience Responses/Strategies	2
Long-term Outcomes	6

For each indicator, the scorers were given a set of details that included the name of the indicator, its definition, relevant methodological details, data sources, links to the key data sources and methodology, and any additional information. Each indicator was evaluated using the following criteria:

» Please indicate the extent to which you agree this indicator meets the criteria. (1 = least agreement, 5 = most agreement)

» » RELEVANCE

RELEVANCE	1 (least)	2	3	4	5 (most)	Opt Out
Can be clearly mapped to the food systems framework	○	○	○	○	○	○
Observing change in the indicator is possible within a decade	○	○	○	○	○	○

» » HIGH QUALITY

HIGH QUALITY	1 (least)	2	3	4	5 (most)	Opt Out
Well-documented methodologies and metadata	○	○	○	○	○	○
Data are nationally representative	○	○	○	○	○	○

» » INTERPRETABLE

INTERPRETABLE	1 (least)	2	3	4	5 (most)	Opt Out
Change has a clear interpretation	○	○	○	○	○	○
Data are comparable across countries	○	○	○	○	○	○

» » IMPORTANCE

IMPORTANCE	1 (least)	2	3	4	5 (most)	Opt Out
Do you agree that this is an important indicator to assess desirable food system transformation?	○	○	○	○	○	○

Composition of Survey Respondents by Domains

There were more collaborators than external experts scoring indicators in general. The number of external experts exceeded the number of collaborators only in the Food Environments domain in the Diets, Nutrition and Health theme and were split 50/50 for Land and Soil domain in the Environment and Climate theme. The bar charts below illustrate the breakdown of survey respondents for each domain under the five themes.

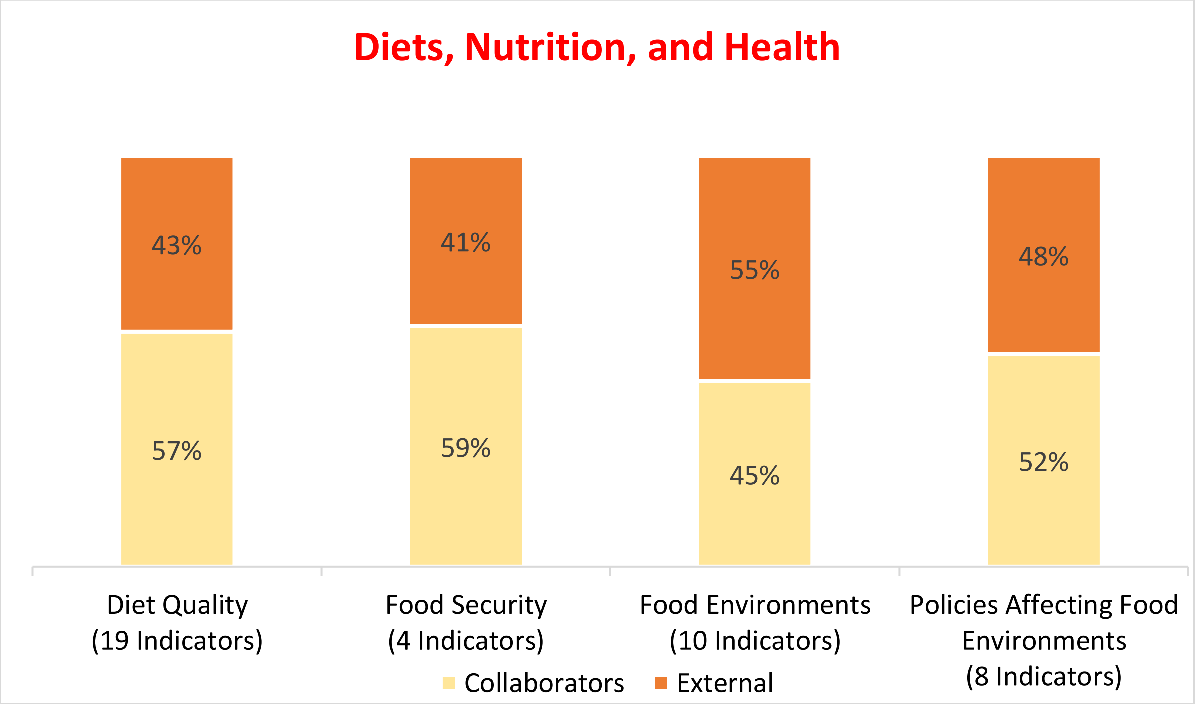

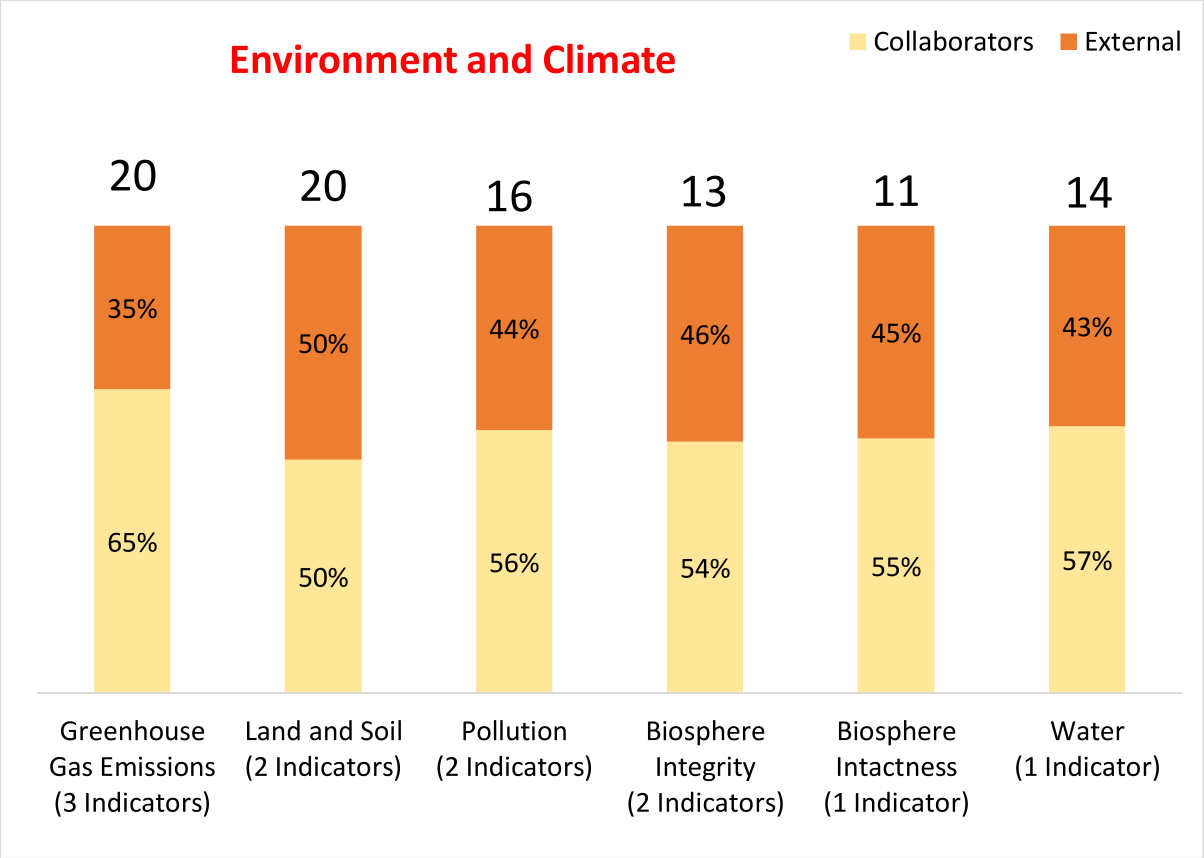
Environment and Climate
Collaborators
External
20
20
16
13
11
14
35%
65%
50%
50%
44%
56%
46%
54%
45%
55%
43%
57%
Greenhouse Gas Emissions (3 Indicators)
Land and Soil (2 Indicators)
Pollution (2 Indicators)
Biosphere Integrity (2 Indicators)
Biosphere Intactness (1 Indicator)
Water (1 Indicator)

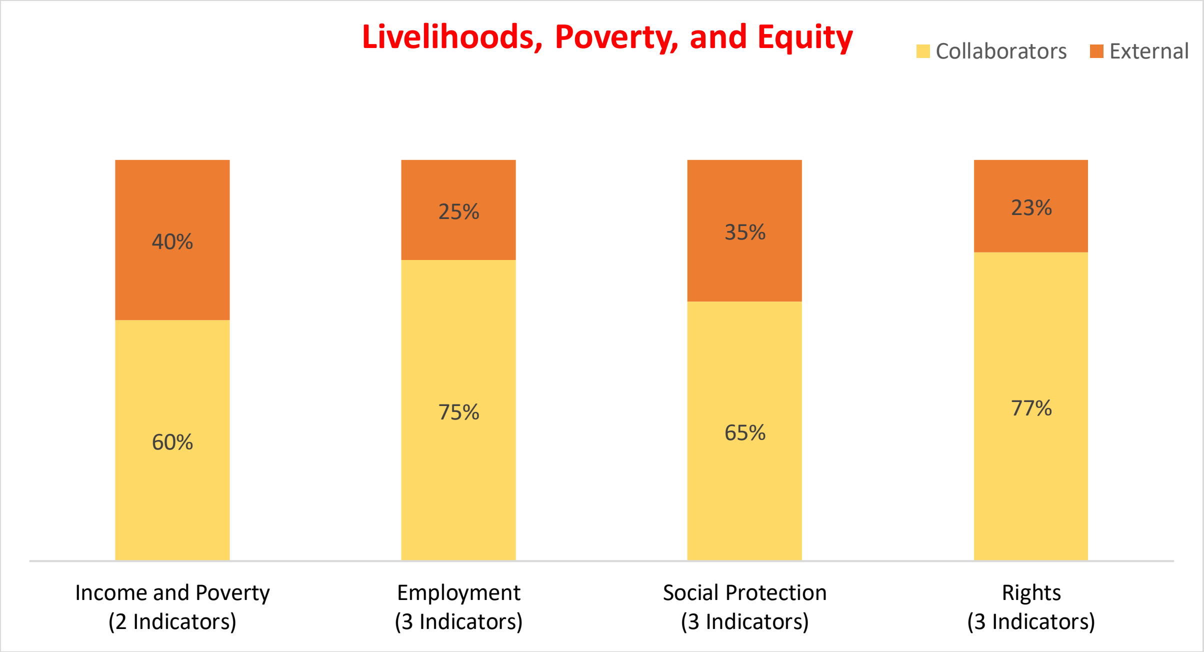
Livelihoods, Poverty, and Equity
Collaborators
External
40%
60%
25%
75%
35%
65%
23%
77%
Income and Poverty (2 Indicators)
Employment (3 Indicators)
Social Protection (3 Indicators)
Rights (3 Indicators)

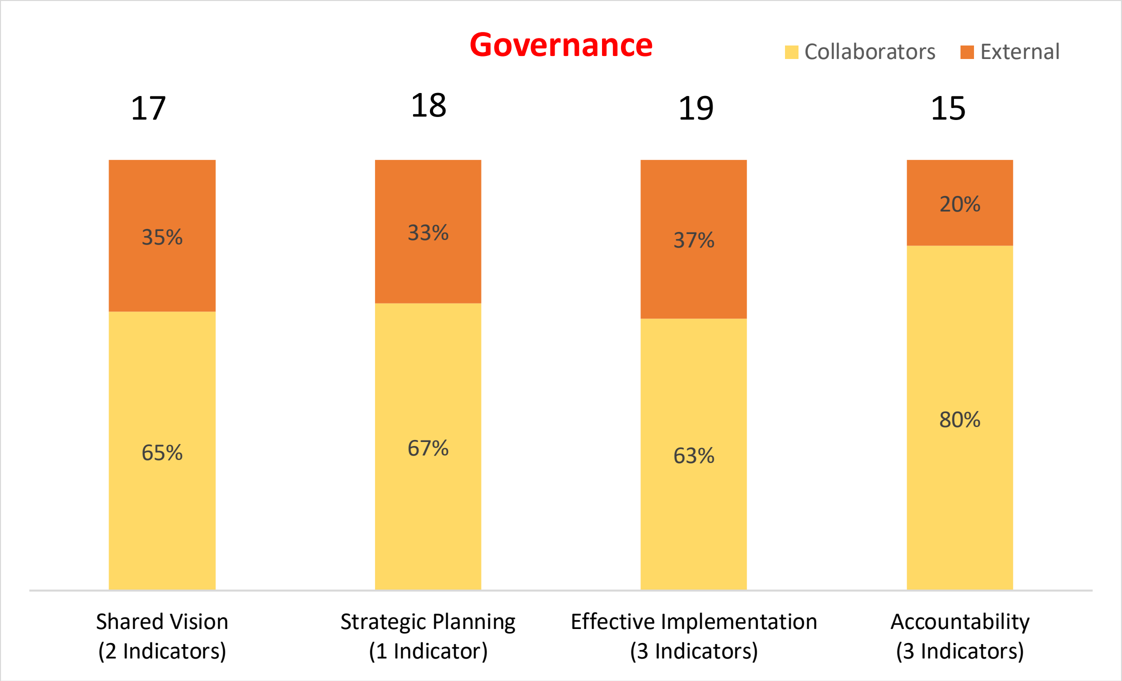

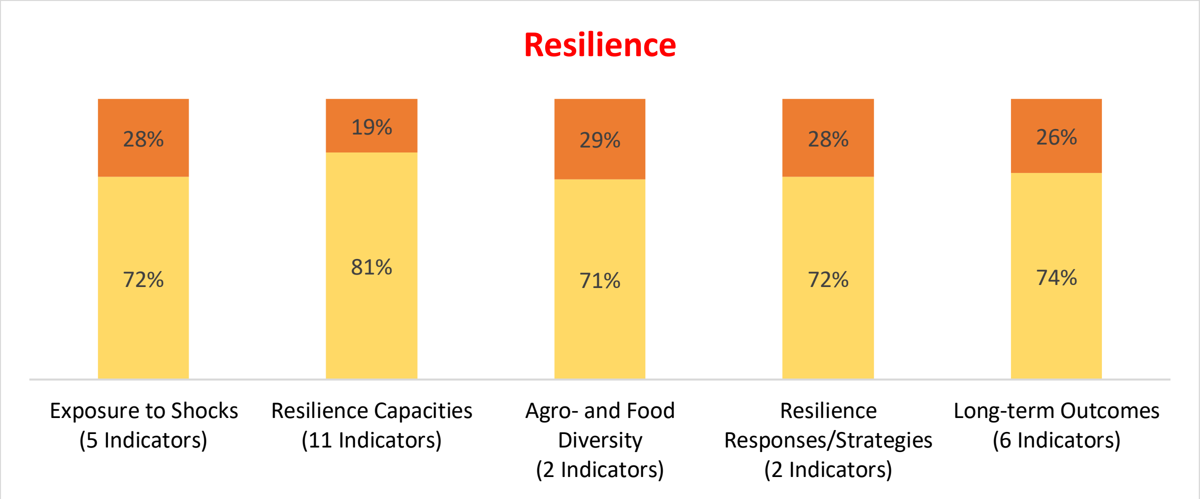

Indicator Scoring Results

Using the survey data, we calculated a summary score for each indicator. We began by calculating a weighted average score at the criterion level. Because different numbers of respondents scored each indicator, and there was an option to opt out of scoring any single criterion, the denominator of the weighted average is the number of respondents who selected a score (1 to 5) for that criterion. The criteria scores for each indicator were then added up to get an overall indicator score. The indicators within each domain were then ranked on the summary score to determine which were highest and lowest ranked. Scoring and ranking indicators helped the core collaborators determine which indicators to include for monitoring purposes.

The table below ranks the top two indicators for each domain. Some domains only have one or two indicators, so they are listed as is.

Domain	Top 2 Indicators	Weighted Score	Rank within Domain	Rank within Theme
**Diet Quality**	Minimum Dietary Diversity for Women of Reproductive Age (MDD-W)	**30.64**	**1**	**2**
	Minimum Dietary Diversity (MDD) among IYC (age 6-23 months)	**30.11**	**2**	**5**
**Food Security**	Food Insecurity Experience Scale (FIES) - based indicators	**30.72**	**1**	**1**
	Proportion of people who cannot afford a healthy diet	**30.30**	**2**	**3**
**Food Environments**	Cost of a Healthy Diet	**29.65**	**1**	**6**
	Availability/supply (g/day/capita) - vegetables	**28.54**	**2**	**12**
**Policies Affecting Food Environments**	Best-practice policy implemented for industrially produced trans-fatty acids (TFA) (Y/N)	**28.86**	**1**	**9**
	Implementation of marketing of breast-milk substitutes restrictions (Fully achieved/ Partially achieved / Not achieved)	**28.06**	**2**	**17**
**Greenhouse Gas Emission**	Greenhouse gas emissions from food systems (farm to fork)	**31.22**	**1**	**1**
	GHG Emissions intensity	**29.74**	**2**	**3**
**Land and Soil**	Cropland expansion	**27.99**	**1**	**4**
	% change in soil organic carbon	**24.26**	**2**	**11**
**Biosphere Intactness**	biodiversity intactness	**25.11**	**1**	**8**
**Biosphere Integrity**	Functional Integrity (% agricultural land with minimum level of natural habitat)	**25.01**	**1**	**9**
	Fishery health index - Progress score	**24.57**	**2**	**10**
**WATER**	Agricultural water withdrawal as % of total renewable water resources	**30.15**	**1**	**2**
**Pollution**	Total pesticides per unit of land	**27.27**	**1**	**6**

	Sustainable Nitrogen Management index	**26.48**	**2**	**7**
**Shared Vision**	Presence of a national food system transformation pathway	**25.01**	**1**	**5**
	Civil society index, Varieties of Democracy (V-Dem)	**24.31**	**2**	**9**
**Strategic Planning**	Policy coordination	**27.12**	**1**	**1**
**Effective Implementation**	Policy implementation	**27.05**	**1**	**2**
	Government Effectiveness	**25.61**	**2**	**3**
**Accountability**	Budget transparency score, Open Budget Initiative	**25.06**	**1**	**4**
	Voice and Accountability, WGI	**24.56**	**2**	**7**
**Income and Poverty**	Households with significant income from agriculture	**26.13**	**1**	**6**
	% population earning low pay	**25.68**	**2**	**8**
**Employment**	Unemployment, rural and urban	**28.14**	**1**	**2**
	Monthly wages for agricultural workers compared to the country's median monthly wage	**25.59**	**2**	**9**
**Social Protection**	Adequacy of benefits from social protection and labor programs	**27.94**	**1**	**3**
	Coverage of any social protection and labor program	**26.95**	**2**	**4**
**Rights**	Distribution of land holdings by sex (Female %)	**28.17**	**1**	**1**
	Constitutional recognition of the right to adequate food	**25.77**	**2**	**7**
**Exposure to Shocks**	SDG 16.1.2: Conflict-related deaths per 100,000 population, by sex, age, and cause.	**27.93**	**1**	**13**
	EM-DAT economic impact	**27.9**	**2**	**14**
**Resilience Capacities**	Poverty headcount ratio at $1.90 a day	**29.59**	**1**	**3**
	FAO Dietary Sourcing Flexibility Index	**29.43**	**2**	**6**
**Agro- and Food Diversity**	Agrobiodiversity indicator 2: Species diversity in food production	**29.13**	**1**	**10**

	Agrobiodiversity indicator 1: Species diversity in food supply	**29.08**	**2**	**11**
**Resilience Responses/Strategies**	Consumption-based Coping Strategy Index (reduced CSI)	**29.92**	**1**	**2**
	Nature-based solution for adaptation	**26.73**	**2**	**22**
**Long-term Outcomes**	Domestic food price volatility index	**30.71**	**1**	**1**
	Stability of Food Insecurity Experience Scale (FIES) - based indicators	**29.57**	**2**	**4**

Survey respondents were also asked to suggest alternative indicators that were not on the long list. These suggested indicators are as follows:

Theme: **Domain**	**Suggested Indicator**	**Suggested Indicator Data Source**
Diet, Nutrition and Health: **Diet Quality**	Water and Sanitation (component of the Social Progress Index)	https://www.socialprogress.org/index/global
Diet, Nutrition and Health: **Policies Affecting Food Environments**	Food expenditure	LSMS/household surveys; https://inddex.nutrition.tufts.edu/data4diets/data-source/household-consumption-and-expenditure-surveys-hces
Diet, Nutrition and Health: **Policies Affecting Food Environments**	# of dieticians/public health nutritionist in a country	https://www.who.int/data/nutrition/nlis/info/nutrition-professionals-density
Diet, Nutrition and Health: **Diet Quality**	Hypertension	National surveys and WHO step surveys
Environment and Climate: **Pollution**	Food Loss and waste, N and P surplus	https://www.cell.com/one-earth/fulltext/S2590-3322(21)00473-5; https://www.fao.org/platform-food-loss-waste/flw-data/en/#:~:text=The%20Food%20Loss%20and%20Waste,grey%20literature%2C%20countries%20among%20others.
Environment and Climate: **Land and Soil**	deforestation emissions; peatland degradation; fires	FAOSTAT ET domains

Environment and Climate: **Land and Soil**	organic agriculture; forest land	FAOSTAT RL and EL domains
Environment and Climate: **Water**	Water depletion	link
Governance: **Accountability**	I'd like to have seen indicators for discrimination under the "governance: accountability" aim. Additionally, and I appreciate such an off-the-shelf database doesn't exist, but indicators re: stigma for accessing food assistance would be important to know under governance and I would put safety net access and rights protections as governances issues (not under livelihoods)	
Governance: **Accountability**	Hanci - can we reinitiate it?	http://www.hancindex.org/the-index/
Governance: **Effective Implementation**	Nutrition spending?	Check GNR
Governance: **Effective Implementation**	Regulatory Enforcement (component of the WJP Rule of Law Index)	https://worldjusticeproject.org/our-work/wjp-rule-law-index
Resilience: **Long-term Outcomes**	SDG 12.3.1.A Food Loss Index	https://www.fao.org/sustainable-development-goals/indicators/1231/en/
Resilience: **Long-term Outcomes**	SDG 12.3.1.B Food Waste Index	https://www.unep.org/resources/report/unep-food-waste-index-report-2021
Resilience: **Long-term Outcomes**	Gender Equality (component of the SDG Gender Index)	https://www.equalmeasures2030.org/wp-content/uploads/2021/06/SDG-Gender-Index-1.png

Resilience: **Long-term Outcomes**	Political Empowerment (component of the Global Gender Gap Index)	https://www.weforum.org/reports/gender-gap-2020-report-100-years-pay-equality
Resilience: **Long-term Outcomes**	Food Losses Index	FAO
Resilience: **Long-term Outcomes**	Food waste index	UNEP

Participants external to the co-authors:

**Name**	**Affiliation**
Carl Lachat	Department of Food Technology, Safety and Health, Ghent University, Belgium
Inge D. Brouwer	Division of Human Nutrition and Health, Wageningen University and Research, the Netherlands
Anna Lartey	
William A. Masters	Tufts University, Friedman School of Nutrition
Shauna Downs	Rutgers School of Public Health
Jamie Morrison	
Xin Zhang	Appalachian Laboratory, University of Maryland Center for Environmental Science
Chris Vogliano	USAID Advancing Nutrition
Elizabeth L. Fox	Department of Public and Ecosystem Health, Cornell University
Natalia Strigin	International Rescue Committee
Jinfeng Chang	Zhejiang University
Jonathan R.B. Fisher	The Pew Charitable Trusts
Frank Eyhorn	Biovision Foundation
Michelle Jurkovich	Assistant Professor of Political Science, University of Massachusetts Boston
Min Jung Cho	Leiden University
Jing Zhu	
Michelle Holdsworth	IRD (French National Research Institute for Sustainable Development)
Lijun Zuo	Aerospace Information Research Institute, Chinese Academy of Sciences
Mariana Rufino	Lancaster University, UK
Olutayo Adeyemi	Department of Human Nutrition and Dietetics, University of Ibadan, Nigeria
Rachel Nugent	RTI International Center for Global Noncommunicable Diseases
Ojwang A A	The Technical University of Kenya
Ramya Ambikapathi*	Purdue University
Mark Lawrence	IPAN, Deakin University
Julia Compton	

Dr. Isabel Madzorera	Department of Global Health and Population, Harvard School of Public Health
Jamie L. Thomas	Bread for the World
Roberto O. Valdivia	Applied Economics, Oregon State University, USA

* Joined the FSCI collaborators after the execution of the survey.

Regional Stakeholder Consultation Reports

FAO *RAF* Regional Expert Consultation of the Food System Countdown Initiative's Indicator Framework

Virtual, May *19th* 2022
This report was produced by the FAO Regional Office for Africa to summarize the results of the FAO RAF regional expert consultation held on May 19 2022.

Introduction:
Accelerating action towards the achievement of SDGs is becoming more imperative that ever before. With only 8 years left to 2030, it is becoming imperative that concerted and collaborative actions be found to achieve the 2030 SDG Agenda. This is becoming especially urgent given the shocks in food systems that are taking the world off track in achieving the SDGs. For the Africa Region, recent shocks in the food systems are but not limited to Climate Change; Conflicts and terrorism; economic impact of COVID-19 and other pandemics; and the increased Cost of food (the 4C's). Climate shocks destroy lives, crops and livelihoods, undermine people's ability to feed themselves, and have displaced 30 million from their homes globally in 2020. Currently in the Horn of Africa region, Livestock are dying, crops are failing, and an estimated 13 million people wake up hungry every day across the region as it grapples with severe drought caused by the driest condition since 1981.[1]

Conflict are major threats to food security and nutrition and the leading cause of global food crises. Marked increases in the number and complexity of conflicts in the last ten years have eroded gains in food security and nutrition, leading several countries to the brink of famine. Of the more than 800 million chronically food insecure people in the world, 60 percent live in countries affected by conflict. In the Sahel, over 10.5 million people are facing Crisis (IPC 3) levels of hunger, including 1.1 million in Emergency (IPC 4), across five countries - Burkina Faso, Chad, Mali, Mauritania, and Niger. [2]
The COVID-19 pandemic has worsened the prevalence of multiple forms of malnutrition and could have lasting effects. More people slid into chronic hunger in 2020 than in the previous five years combined, and one in five children around the world are stunted. Children are paying the heaviest price, at the beginning of 2020, one in every two schoolchildren, or 388 million children, received school meals every day from national programmes in at least 161 countries from all income levels. The COVID-19 pandemic brought an end to this decade of global growth. At the height of the crisis in April 2020, 370 million children were suddenly deprived of what for many was their main meal of the day with the closure of schools.[3]

The rising food prices, resulting from the COVID-19 pandemic and other factors, including the recent conflict in Ukraine, risk of putting healthy diets out of reach of even more people, on top of the current 964.8 million people in Africa who cannot afford an adequate diet.[4] FAO has estimated that, globally, the number of undernourished people, which was around 817 million in 2021, could increase by 7.6 - 13.1 million people in 2022-2023 due to the conflict between Russia and Ukraine.[5]

---

[1] https://news.un.org/en/story/2022/02/1111472

[2] https://reliefweb.int/sites/reliefweb.int/files/resources/GHI%20February%202022%20Report%20-.pdf

[3] FAO, ECA and AUC. 2021. Africa – Regional Overview of Food Security and Nutrition 2021: Statistics and trends. Accra, FAO. https://doi.org/10.4060/cb7496en

[4] FAO, ECA and AUC. 2021. Africa – Regional Overview of Food Security and Nutrition 2021: Statistics and trends. Accra, FAO. https://doi.org/10.4060/cb7496en

[5] Technical Briefing to FAO Members on the impact of COVID-19 and the war in Ukraine on the outlook for food Security and nutrition, March 2022. https://www.fao.org/3/cb9241en/cb9241en.pdf

For the continent to be back on track to achieving the SDGs, the food systems must transform, in line with the commitments made during the UN Food Systems Summit.[6] Tracking can assess performance relative to established targets and goals and incentivize action. Doing so for food systems complements other global and regional monitoring and tracking initiatives focused on related outcomes, such as sustainable agriculture, nutrition and health. Tracking and assessment also offers food system actors and stakeholders (e.g. civil society, governments and international organizations) actionable evidence to hold governments, consumers (specifically, those with the privilege to choose), and the private sector accountable for food system transformation.

In this regard, The Food Systems Countdown Initiative has arrived at an opportune time for the continent. The Food Systems Countdown Initiative (the 'Initiative') is working to build a science-based observational system to track the performance and transformation of food systems globally. The 2021 UN Food Systems Summit presented a window of opportunity with food systems on the international political agenda, yet no rigorous mechanism currently exists to measure and track all aspects of food systems, their interactions and their changes over time. Deliberately changing complex systems that cut across sectors, jurisdictions and national borders calls for a comprehensive, ongoing programme of scientific measurement, tracking, and assessment of all aspects of the system to guide decision-makers and hold those in power to account for transformation. Led by John Hopkins University, GAIN and FAO, an unparalleled partnership has come together to implement the Initiative. Over 50 collaborating scientists have joined from nearly 30 organizations across academia, NGOs, and UN agencies, and from nearly all continents.

The principal goal of the Initiative is to provide independent tracking of a curated, parsimonious set of indicators that together cover all the important aspects of food systems. The architecture so far proposed covers the five thematic areas described below, each with three to five indicator domains (i.e. subtopics for which indicators must be found to capture that aspect of the thematic area). Fanzo et al. (2021) defined criteria for indicators to be included in the tracking system that will be used to guide the selection of indicators. The architecture covers five domains as follows: 1. Diets and nutrition; 2. Environment and climate domain 3. Livelihoods, poverty and equity Domain; 4. Governance Domain 5. Resilience and sustainability Domain

Objectives of the RAF Regional Expert Consultation

In line with The Initiative's commitment to an inclusive, consultative, and transparent process, the objective of the FAORAF consultation was to subject the set of propped indicators to a validation and peer review mechanism by carefully selected experts and scientist working with data and policy. The regional expert consultation is an opportunity to get inputs, comments, and suggestions on the monitoring framework proposed by the initiative. The framework is not mandatory. However, the consultations ensure that it has the capacity to be a useful tool for policy decision-making processes. The outcome of each regional consultation will be a public document that summarizes the inputs received in each thematic area.

For the Africa Region, the experts were drawn from those working at country level in policy development from the relevant Planning Units of the Ministries of Agriculture, National Statistical Offices and Ministries working on the thematic areas of the indicator framework; regional level experts, including non-state actors, academia and NGOs. The experts working and collaborating with the Comprehensive Africa Agriculture Development Programme (CAADP) were of particular value given that the CAADP initiative is an African led process sanctioned by Heads of Member States that has the objective of tracking collective performances in order to trigger continental, regional and national level action programmes to drive agricultural transformation in Africa.

[6] https://www.un.org/en/food-systems-summit

The CAADP process has strong political support that strengthens national and regional institutional capacity for agriculture data collection and knowledge management to inform actions that will support improved evidence-based planning, implementation, monitoring and evaluation, mutual learning and foster alignment, harmonization and coordination among multi-sectoral and multi-stakeholder efforts.

Opening:
Participants were welcomed by Mr Mphumuzi Sukati – Senior Nutrition and Food Systems Officer, who was stepping in for the Regional Programme Leader, Mr Ade Freeman. Following his welcoming of participant, Mr Jose Rosero Moncayo, Director of Statistics gave the opening remarks. He noted the importance of food systems transformation to deliver better on SDGs, and the need to track this transformation in line with the UN Food Systems Summit and the commitments made by partners and Member States to ensure sustainability of food systems. He then introduced briefly *The Food Systems Countdown Initiative* framework and what it entails in helping countries monitor their progress towards transforming food systems for the accelerated achievement of SDGs.

Presentation of Themes
Methodology
Following the opening remarks, the session started with keynote presentation that gave an overview of the role food systems play in meeting all 17 sustainable development goals. The overview highlighted the key role of The Food Systems Countdown Initiative (FSCI), which is to fill the gap on lack of rigorous mechanism to track food systems change, despite the need for food systems to transform. The overview highlighted the importance of actionable evidence to track progress, guide decision-makers, and inform transformation, and placed emphasis on complementing other monitoring and tracking of related goals at global and regional scales.

Following the overview, the FSCI was welcomed by the African experts, who felt that it has come at an opportune time. This was with the realization that the continent has its own framework to track progress towards transforming agriculture and food systems, which needs reinforcement. The greatest albatross of the CAADP initiative in particular, is the quality of data that is produced to track progress towards the Malabo targets of 2025 and challenges of aligning the Malabo Targets to the SDGs.

The consultation was conducted at a time when the African Union (AU) had convened a joint meeting of ministers in charge of agriculture, trade and finance from the AU Member States on the impacts of COVID-19 on African food systems, and the preparation and the presentation of the African common position to the UNFSS as a continent. In both instances, there was a recommitment to advance agricultural transformation of the continent through strengthening the implementation of CAADP and the Biennial Review process as the tool to assess progress on the implementation of the recommendations and the game changing solutions contained in the Africa common position to the UNFSS.

Following the plenary session, subsequent discussions took place in side events, under five sessions covering the themes of the indicator framework as follows: Session 1: Diets, Nutrition and Health; Session 2: Environment and climate Domain; Session 3: Livelihoods, poverty and equity Domain; Session 4: Governance Domain; and Session 5: Resilience and sustainability.

In each session, the discussions were motivated by a presentation on the proposed set of indicators. The discussions focused on the capacity of the proposed indicators to guide policy decisions and promote accountability mechanisms. In this sense, the focus was on the relevance, usefulness, and validity of the proposed set of indicators from a regional perspective.

The session on Diets, nutrition and Health need to monitor if:

- People have access to healthy diets? (*Food and nutrition security*)

- People consume healthy diets? (*Diet quality and adequate nutrients content*)
- Food environments support people to access and consume healthy diets? (*Access to Healthy Diets*)
- Policies contribute positively or negatively toward food availability, food access, product properties (e.g. food safety, labelling), and food messaging? (*Policies affecting food environments)*

The Session on Environment and Climate seeks to monitor:

- Food systems as a major source of environmental degradation
- Actions required achieving global environmental commitments
- Monitoring and accountability essential

The session on Livelihoods, poverty and equity aims to monitor;

- The number of people working as part of the food system, spanning rural and urban areas, high and low-income countries
- The diversity of livelihood supported by the diversity in food systems: farming, transport, processing, formal and informal retail, "gig economy", etc.
- The extent of poverty, vulnerability and exploitation across the food systems

The session on Governance seeks to monitor;

- Shared expectations for outcomes
- Relevant policy instruments to align efforts and bring about change
- Implementation capacity and resources
- Accountability for outcomes

The session on Resilience and sustainability seeks to monitor food systems based on the following issues:

- Food systems resilience being critical to food security and nutrition (e.g., fragile states = food insecurity, COVID-19).
- Food systems being critical for other functions (e.g., livelihoods, inclusion).
- Resilience being a pre-condition for sustainability.
- Sustainability having multi-dimensional interpretation
- Food system sustainability contributing to SDGs
- Normative element in the food systems transformation and resilience (transformation per se is not enough)
- Monitoring sustainability being necessary to capture food systems' holistic nature.

Guiding questions to animate the discussions were the following:
Do you consider the proposed indicators:
- Relevant, defined as their ability to measure something meaningful for food systems across a variety of settings, during relevant time periods?
- High quality, defined as using the best and most rigorous statistical methodologies and data available?
- Interpretable defined as having the ability to show a clear desirable direction of change, comparable across time and space, and easily communicated.
- Useful, defined as its ability to be used for policy and decision-making processes and by meeting actual information needs.

What are the data gaps that you can identify? Are these gaps structural? If not, are there some data or indicators that you know are available in your region and can be used for the purpose of this monitoring and assessment system?
What are the regional aspects we need to take into consideration now of selecting a set of indicators to monitor the state of food systems and its evolution?

Key observations from all the sessions were as follows;

- The Initiative was welcomed by participants who all appreciated the work done. It was noted that this Initiative was going to be an important addition to Africa's efforts t to strengthen tracking of

progress towards achieving the SDGs and the Malabo Targets. It was noted that food systems play a major role in sustainable development and reversing the hunger and malnutrition statistics that continue to be an albatross weighing down on the continent.

- Having high quality data will help improve the statistical significance and reliability of estimated relationships between policies, investments, and outcomes. Hence, policymakers and investors can be more confident in using results of strategic analysis to make policies and investments that are more likely to lead to desirable outcomes.
- Capacity shortfalls to collect, analyze and report reliable statistics that will inform policy was a major observation.
- The indicators for tracking food systems transformation were many and at times confusing.
- Strengthening institutional capacity to track progress and putting in place strong M&E frameworks was key.
- The need to focus on demand driven indicators that respond to specific challenges in countries and regions.
- Gap in advocacy and wide dissemination of the indicators for national and regional buy in and adoption.
- Importance of political support to the initiative was highlighted and financing the data collection, following results based financing approaches.
- The need to analyze the interaction between the indicators was also discussed, considering the mechanisms in which these interactions progress over time – The "Law of Motion" of the indicators for food systems transformation in general.
- The need to bring in indicators outside the food systems that have an impact on food systems or influence food systems transformation.
- Advocacy for domestication of the indicators – based on international frameworks and conventions was also raised
- Analysis of the commonality of the indicators across countries and their divergence given countries and regional heterogeneities.
- Considerations on how the indicators are related to continental and regional initiatives like the African Continental Free Trade Area and Food Safety.
- There was a general concert to translate the indicators to French and other working languages in the continent where possible for ease of adoption of indicators by countries

Discussion

Diets and nutrition

Do you consider the proposed indicators relevant, high quality, interpretable and useful?

On this question, participants noted that the indicators proposed are relevant high quality, interpretable and useful. However, there is a need to define clearly the *"zero fruits and vegetable meaning"* for the indicator measuring the diet's quality. The indicators were many and were diverse, meaning it was important to aggregate some of them. Countries did not have the indicators, meaning that a lot of advocacy and disseminating the indicators was still needed.

The participants said that it might be difficult to interpret the indicator, as it should take into consideration the quantity, the frequency, the seasonality and safety. This will be complicated by the capacity at country level to collect, analyze and report on the data and the indicators.
Furthermore, the selection of the indicators in the category should be justified and it is important and relevant to compare the data according to gender, age and economical status of the respondent.

What are the data gaps that you can identify? Are these gaps structural? If not, are there some data or indicators that you know are available in your region and can be used for the purpose of this monitoring and assessment system?

The participants agreed that there is a lack of data in their countries and a lot of efforts should be done to ensure accurate and quality data to be analyzed.

Indicators on the percentage of locally produced and locally processed food components in the diet consumed, especially by urban consumers can be considered. Another indicator is the percentage of locally produced foods and commodities that are successfully integrated in urban food supply chains. Regarding the food policies, there is a need to have indicator(s) that track the enforcement of laws/policies, integrated into food safety standards and regulations for example.

What are the regional aspects we need to take into consideration at the moment of selecting a set of indicators to monitor the state of food systems and its evolution?

The data collection should be coordinated and harmonized building on existing initiatives. The CAADP M&E framework is seen as a key entry point.
The following points should be taken into consideration:

Growing population as the African population will double by 2050
Poor industrialization; high import of food commodities
Food safety
Cultures and food habits should be considered
Promotion of locally produced foods should be part of the strategies
Quality of water , as water play a key role in the health it is important to ensure that we have clean water

Environment and climate domain
Do you consider the proposed indicators relevant, high quality, interpretable and useful?
It was generally agreed that the data and indicators were of high quality and relevant.
However, it was felt that climate change was a complex phenomenon that requires specialized and highly technical data set and expertise to track its effect on food systems and importantly, the role of food systems in driving climate change itself.

What are the data gaps that you can identify? Are these gaps structural? If not, are there some data or indicators that you know are available in your region and can be used for the purpose of this monitoring and assessment system?

The climate change indicators must respond to global climate change frameworks, conversion, legislation and agreements.

Data and indicators to respond to environment and climate change must consider areas linked to climate mitigation, climate adaptation, and the linkages between climate change and the broader 2030 Agenda.
It was noted that Africa has lower carbon footprints yet the continents bears the brunt of the effects of climate change. In this regard, it was suggested that climate change and environment indicators for Africa must place strong emphasis on climate adaptation rather than mitigation, and make use of the global carbon trading schemes where possible.

The importance of coming up with regional data hubs for supporting the vetting of the indicators, data collection and coordination, reporting and mutual accountability was also raised. This will help in domestication of the indicators and identifying common indicators that will be easily comparable adaptable across countries.

It was noted that because of the complexity of the climate change subject and its wide scope and interlinkages, coming up with a set of smaller number of reliable indicators was not easy but this was a way to go, especially for countries with limited capacity to collect data.

Participants noted that data and indicators are becoming complex in line with the rapidly transforming food systems and the sporadic shocks in the system. For a complex subject like climate change, there is weak capacity to provide the system-wide coordination that is needed to provide greater access to data, identify and address data gaps or deliver data synthesis and assessment.

What are the regional aspects we need to take into consideration at the moment of selecting a set of indicators to monitor the state of food systems and its evolution?

Again, the importance of embedding the environment and climate change indicators to the CAADP process was raised, and linking the suggested indicators to the resilience indicators that already exist. The complexity of climate change and environmental systems and the rapidity in which they are progressing need special expertise and support in tracking these changes.

<u>Livelihoods, poverty and equity Domain</u>
Do you consider the proposed indicators relevant, high quality, interpretable and useful?
Participants agreed that, the indicators mentioned were very relevant and useful but still need other areas to be considered if we are indeed aiming at transforming food systems in Africa.
However, more collaborators should be included to enrich the existing information and be able to fill the missing gaps.

Participants also felt that reviewing of documents should be done to avoid duplication of ideas. To achieve sustainable agriculture and food systems, there should be more research and data metrics to fill knowledge gaps in terms of economic efficiency, environmental and social sustainability, and links to food security and nutrition for the transformation of food systems. The following indicators were considered important to have in the list:

- Post-harvest Losses
- Agric-business
- Agric value chain
- Involvement of the youth and integrating gender aspects
- Agriculture should be made attractive for all especially the youth
- Introduction of more user-technology / tools
- National Investment Programme (NIP) should be looked at again
- Identify the link between NIP and CAADP

What are the data gaps that you can identify? Are these gaps structural? If not, are there some data or indicators that you know are available in your region and can be used for the purpose of this monitoring and assessment system?
Data collection is always an issue; a lot of work still need to be done in that area. Lack of data makes it very difficult to move on. Without data, it will be very difficult to improve and transform the food systems in Africa and the world as a whole, and to have reliable baselines.
Other innovative indicators should be considered to achieving sustainable agriculture and food systems especially given the rapidity of the transformation in food systems.
What are the regional aspects we need to take into consideration at the moment of selecting a set of indicators to monitor the state of food systems and its evolution?
It was observed that there should be direct youth engagement and involvement in policymaking at local and global levels when it comes to agriculture and the need to make agriculture more attractive so many people could join especially the youth.

<u>Governance Domain</u>
Do you consider the proposed indicators relevant, high quality, interpretable and useful?

The indicators on Governance were welcomed by participants and were found to be very relevant. These indicators were observed to play a pivotal role in holding governments accountable to their commitments to transform food systems to deliver better on the SDGs. They also noted that the indicators were important in institutionalizing the national, regional and global commitments to transform food systems, grounded at country level.

What are the data gaps that you can identify? Are these gaps structural? If not, are there some data or indicators that you know are available in your region and can be used for the purpose of this monitoring and assessment system?
Data gaps on the governance domain were to do with lack of indicators to track public sector engagement in governance of food systems transformation, something that the initiative can consider. Balancing data from existing official structures with those from emerging or unofficial structures was again noted. Ensuring that the needs of data users are met and integrating this with country-level data support mechanisms was highlighted. Facilitating access to high quality, usable data and strengthening transparent and accountable governance structures, M&E and mutual accountability frameworks was raised.

What are the regional aspects we need to take into consideration at the moment of selecting a set of indicators to monitor the state of food systems and its evolution?
Indicators of governance of land rights and access to land and factors of production by marginalized groups and women was mentioned for consideration
It was again suggested that for the Initiative to gain the political mandate to hold governments accountable, it must be embedded into the CAADP accountability framework that has been sanction and adopted by Member States.

Resilience and sustainability Domain
Do you consider the proposed indicators relevant, high quality, interpretable and useful?
The indicators proposed are relevant, high quality, interpretable and useful. Additionally, participants stressed the fact that the indicators should be adapted to national realities as well as a difference should be made between individual, household, and community indicators. In fact each level has it own way to cope and it is crucial to capture them well for a better analysis.

What are the data gaps that you can identify? Are these gaps structural? If not, are there some data or indicators that you know are available in your region and can be used for the purpose of this monitoring and assessment system?
When it comes to measure resilience and sustainability, there are data gaps. This is because there is no consensus on the indicators to use. It leads to confusion i.e the Resilience Index Measurement and Analysis (RIMA) and other indicators.

What are the regional aspects we need to take into consideration at the moment of selecting a set of indicators to monitor the state of food systems and its evolution?
The CAADP M&E framework could be useful, and capacity building should be part of the plan. Furthermore, the selection of indicators should be demand driven and sub-national aspects should be considered.
In fact, the subnational aspect might pilot the indicators in certain countries to test their uptake and domestication.

Overall recommendations

- Strengthening institutional capacity to track progress and strong M&E frameworks.
- Coming up with regional and sub-regional data hubs for supporting data collection, analysis, M&E and peer review and mutual accountability mechanisms

- Mainstreaming the indicators into the CAADP framework was seen as a low hanging fruit for their adaptability and the political buy in.
- Strengthening advocacy for domestication of the indicators – based on international frameworks and conventions.
- The indicators should also respond to tools that monitor food and nutrition security. In particular, the issue of Food Based Dietary Guidelines was given as an example.
- There was a general concert to translate the indicators to French and other working languages in the continent where possible for ease of adoption of indicators by countries
- Gender and youth considerations in the data was recommended.

Agenda of meeting

GMT or Accra Time

**Time**	**Subject**	**Speakers**
9:00 – 9:30	Plenary: Welcoming remarks and introduction to the Initiative	*FAO*
9:30 -10:30	Block 1:<br>Diet, Nutrition, and Health;<br>Environment and climate Domain;<br>Livelihoods, poverty and equity Domain	*FAO*<br>*Participants*<br>*Moderator*
10:30 - 10:45	Break	
10:45 -11:45	Block 2:<br>Governance Domain;<br>Resilience and sustainability Domain	*FAO*<br>*Participants*<br>*Moderator*
11:45-12:00	Break	
12:00-13:00	Wrap up and Conclusions	*Moderator*

Annex 2: Invited Participants List

Region	Country	Nom & Prénoms	Fonction	Organisation
**ECCAS**	Cameroon	**Ondoa Manga Tobie**	Point Focal PDDAA	Ministère de l'Agriculture et du Développement Rural
		**Ntouda Betsogo julien**	Chef de Service du Traitement des Données	Ministère de l'Agriculture et du Développement Rural
		**Amos Bassia Bassia**		Ministère de l'Agriculture et du Développement Rural
	Central African Republic	**Francis Doui**	Point Focal PDDAA	Ministère de l'Agriculture et du Développement Rural
		**Sylvie Solange NDODET BETIBANGUI**	Point Focal suivi de la Déclaration de Malabo	Ministère de l'Agriculture et du Développement Rural
	Chad	**AHOURDET DJAPANIA**	Point Focal PDDAA	Ministère de Production de l'Irrigation et des Equipements Agricoles
		**Djimadoum Djeramian**	RB Point focal	Ministère de Production de l'Irrigation et des Equipements Agricoles
		**EDMOND SOULEINGAR**	Statiscien agricole	CELLULE PERMANENTE
		**Abdel Kerim Mahamat Issakha**	Expert Revue Biennale	Ministère de l'Agriculture
		**Abakar Mahamat**	Expert Revue Biennale	Ministère de l'Agriculture
	Congo Republic	**Mobengue Cyprien**	Point Focal PDDAA	Ministère de l'Agriculture, de l'Evage et de la Pêche
		**Pierre MPANDOU**	Planificateur en développement	Ministère de l'Agriculture, de l'Evage et de la Pêche
		**HOMBESSA KIAKOUNDA Oriali Gaël Godlov**	Statisticien informatique et responsable de saisie des données RB	Ministère de l'Agriculture, de l'Evage et de la Pêche
	DR Congo	**Paul M'FINDA**	Chef de Division chargé des TIC et Point Focal RB	Ministère de l'Agriculture
		**Alain Etango**	Chargé de saisie des données RB, Expert à la Direction d'Etudes et Planification et en charge de Programmes et Suivi	Ministère de l'Agriculture
		**Jean LUFIMPU LUKOMBO**	Point Focal RB	Ministère de l'Agriculture
		**Albine BUNGA**	Gestion des données	Ministère de l'Agriculture
	Equatorial Guinea	**José - Juan NDONG TOM**	Point Focal PDDAA	Ministerio de Agricultura, Ganadería y Alimentation (MAGA)
		**Aquiles-Serafin CHONI BOLOPO**	Statiscien agricole	Ministerio de Agricultura, Ganadería y Alimentation (MAGA)

		**Mba NGUI ANGUESOMO**	Responsable Adjoint de saisie des données RB	Ministerio de Agricultura, Ganadería y Alimentation (MAGA)
		**Madame Estefania Isabel NGUEMA ANDEME**	Directeur Générale de vulgarisation, formation coopérative et de Mécanisation	Ministère de l'Agriculture, de l'Evage des Forêts et de l'Environnement
	Gabon	**Nguema Ndong Patrick**	Spécialiste en S&E et Revue Biennale	MAE et l'Alimentation chargé du Programme GRAINE
		**Mesmin Ndong Biyo'o**	Point Focal PDDAA	MAE et l'Alimentation chargé du Programme GRAINE
	São Tomé and Príncipe	**Raimundo Gonsalves JB**	Technicien au Ministère	Ministère de l'Agriculture
		**Idalécio Guadalupe Pereira Neto**	Technicien au Ministère	Ministère de l'Agriculture
**UMA**	Algeria	BENYAHIA-ZEGHLI	Point Focal PDDAA/Sous-directrice de la Coopération	
		selsabil chouihat	ingénieur agronome	ministère de l'agriculture
		Sabrina Ichou	Chargée d'études et de synthèse	Ministère de l'agriculture et du développement rural
		Amel YESREF		MADR
	Egypt	Ahmed Abdeen	Agriculture engineering/Animal production	Ministry of agreculture
		Entessar El sayed Emam Desouky	Director General Department General for Coordinating the projects and Agricultural Affairs of the COMESA Countries	Ministry of Agriculture and Land Reclamation
		hamdi swilam	Engineering of agriculture	Ministry of agriculture
		DR. HOSSAM SALLAM	Agricultural engineer	Ministry of agriculture and Land reclamation
	Libya	Mohamed Ashour Adham	Point Focal PDDAA	
	Mauritania	Abdellahi zeyad	Point Focal PDDAA	
	Morocco	Zakaria Aharrouille	Point Focal PDDAA	
		MUSTAPHA ABDERRAFIK	CAADP Biennial Review	
		YAHYA FARESS	CAADP Biennial Review	
	Tunisia	Souhir Belaid	Point Focal PDDAA	
		kamel zaidi		ministère agriculture

		Amina HICHRI		Ministère de l'Agriculture, des Ressources Hydrauliques et de la Peche
**EAC**	Burundi	Mr Elias Ngendabanyikwa	CAADP Focal Person	Ministere de l'Agriculture, Elevage
		Zénon Nsananikiye	CAADP Focal Person	Ministere de l'Agriculture, Elevage
	Rwanda	Bertrand Dushimayezu	CAADP Focal Person / Statistician	Ministry of Agriculture and Animal Resources
		Joas Tugizimana	CAADP Focal Person / M&E	Ministry of Agriculture and Animal Resources
	Kenya	Josephine Love	CAADP Focal Person	Ministry of Agriculture
		Isaiah Okeyo	CAADP Focal Person	Ministry of Agriculture
	Uganda	Stephen Kayongo	Commisioner/CAADP Focal Point	Ministry of Agriculture, Animal Industry and Fisheries
		Fred Mayanja	Commisioner/CAADP Focal Point	Ministry of Agriculture and Animal Industry and Fisheries
	Tanzania	Daines P. Mtei	CAADP Focal Person	Ministry of Agriculture
		DAINES MTEI	CAADP F.P	ministry of agriculture
		Irene S. Lucas	M&E Officer	Ministry of Agriculture
	South Sudan	Loro George Leju Lugő	CAADP Focal Point	Ministry of Agriculture, Fisheries and Livestock
		Stephen Wani Augustino	Statistician	Ministry of Agriculture and Forests
**IGAD, COMESA**	Djibouti	Ibrahim Djama Ismael		Ministry of Agriculture, water , Livestock and Fisheries
		Moussa Issak farah	Statistician	Ministre of Agriculture
	Ethiopia	Alemtsehay Hailegiorgis	food and nutrition coordination office head	Ministry of Agriculture
		Seble Tilahun	Monitoring and Evaluation Expert	Ministry of Agriculture
	Somalia	Mohamud Artan	Director of Planning and Policy	Ministry of Agriculture and Irrigation
		Mohamed Gure	Head of M & E	MOAI
		Suleikha Ahmed Ali	Head of statistics	Ministry of agriculture and irrigation
	Sudan	Hamza Abdalla Siror Osman	Information and Analysis Unit Coordinator Food Technical Secretariat (FSTS)	Ministry of Agriculture & Forests
		Mr. Babiker Hassan Adam Mohammed	CAADP M&E Officer	Ministry of Agriculture and Forests
		Zeina Abbas	M&E officer	Ministry of Agriculture
		Bahlbi Goita	BR	Ministry of Agriculture

	Eritre a	Bereket Tsehaye	BR	Ministry of Agriculture
**SADC**	Angol a	Beto pinto	BR	Ministry of Agriculture
		Gabriel Domingos	BR	minagrip
	Com oros	Issouf Miradji Ambadi	BR	Ministère de l'Agriculture, de la Pêche et de l'Environnement
		Mahdy Youssouf	BR	Ministère de l'Agriculture, de la Pêche et de l'Environnement
	Bots wana	GORATA BOIKANYO	AGRICULTURAL ECONOMIST	MINISTRY OF AGRICULTURE
		Sandra Modise	Statistician	Statistics Botswana
		Bueno Shanto Mokhutshwane	District Animal Production Officer	Ministry of Agricultural Development and Food Security; Department of Animal Production
		Sandra Modise	Statistician	Statistics Botswana
	Eswa tini	Howard mbuyisa	snr economist/ caadp focal point	ministry of agriculture
		Kunene Sebenele	Planning officer	Ministry of agriculture
	Lesot ho	Maoala Khesa	Senior Economic Planner and CAADP Focal Point	Ministry of Agriculture and Food Security
		Leeto Semethe	Economic Planner	Ministry of Agriculture, Marketing and Food Security
	Mala wi	Daudi Chinong'one	Principal Economist	Ministry of Agriculture
		Charles Chinkhuntha	Chief Economist	Ministry of Agriculture
		Readwell Musopole		Ministry of Agriculture
		Emmanuel Mwanaleza	Statistician	Ministry of Agriculture, Irrigation and Water Development
	Mada gascar	Mr. RAMBOLARIMANAN A Tahiana	Director of Planning and Monitoring and Evaluation	Ministry of Agriculture, Livestock and Fisheries (MAEP)
		Mrs SOLONITOMPOARIN ONY Jocelyne	Head of Agricultural Statistics	Ministry of Agriculture, Livestock and Fisheries (MAEP)
		Mr. RANDRIANAIVOMA NANA Andritiana Luc	Directeur de la Planification et du Suivi-Evaluation	
	Maur itius	Krishna Chikhuri	Sappdo	Ministry of Agro-Industry & Food Security/Trade
		Varuna Dreepaul		Moafs
		Maria Manussa	head of Department do Agriculture Policy	Ministry of Agriculture and Rural Development

	Mozambique	Duque Wilson	Hod	Ministry of Agriculture and Rural Development : Mozambique
	Namibia	Petrus Uushona	Policy Analyst, Policy Planning and Monitoring and Evaluation	Ministry of Agriculture,
		Stephanus SANDA	Chief Statistician	Ministry of Agriculture, Water and Land Reform
		Eric Kana Miaso	Acting Chief Development Planner-Monitoring & Evalutaion	Ministry of Agriculture, Water and Land Reform
	South Africa	Bongeka Mdleleni	Deputy Director	Department of Agriculture, Land Reform and Rural Development
		Kelefetswe Seleka	Monitoring and Evaluation Specialist	Department of Agriculture, Land Reform and Rural Development
		Heidi Phahlane	Agric Economist	DALRRD
		Nico De Kock	Senior agric economist	Dept of Agriculture
	Zambia	Christopher Mbewe	caadp focal/agricultural economist	moa
		Mweemba Chijoka	Senior Statistican	Ministry of Fisheries and Livestock
		Mary Chilala	Principal Economist/Planning, Monitoring and Evaluation	Ministry of Agriculture
		Yotam Nyirenda	Economists	Ministry of Agriculture
	Zimbabwe	Fredy Chinyavanhu	Deputy Director-Food Control	Ministry of Health and Child Care
		Grace Nicholas		
	Seychelles	Sylvie Larue	BR	Ministry of Agriculture
**ECOWAS**	Benin	Aguemon Dossa	Représentant du Point Focal PDDAA	Directeur de la Prospective et de la Programmation, Ministere de l/Agricultire, Elevage et peche
		ACAKPO Charles	Représentant du Point Focal S&E du PDDAA	Chef Division Évaluation des Performances du Secteur et Capitalisation (CSE/DPP/MAEP), Ministere de l/Agricultire, Elevage et peche
		BIAOU Samon Alexandre	Statisticien	Directeur de la Statistique Agricole, Ministere de l/Agricultire, Elevage et peche
		Odilon Steeven ZOHOUN		SAKSS Bénin

	Burkina Faso	SANA Souleimane	Spécialiste Suivi & Evaluation (S&E) PDDAA	Ministère de l’Agriculture et des Amengements Hydro-agricoles
		SOME Gustave	Directeur Suivi & Evaluation (S&E) PDDAA	Ministère de l’Elevage et des Ressources Animales
		NIKIEMA Adama	Statisticien/DGESS	Ministère de l’Agriculture et des Amengements Hydro-agricoles
	Cabo Verde	Clarimundo Pina Goncaives	Ponto Focal PNIA/PDDAA	Ministère de l’Agriculture
		TEIXEIRA Arilde Emilia	Responsável Seguimento & Avaliação	Ministère de l’Agriculture
		Emanuela.Santos		Instituto Nacional de estatística
		Emanuela Santos	Responsável pela componente Estatística (contas nacionais, dados macro - económicos, dados sectoriais etc.)	Ministère de l’Agriculture
	Côte d’Ivoire	Kouassi Adjoua Jeannine	Point Focal PDDAA	Ministère de l'Agriculture et du Développement Rural (MINADER)
		Mme KOMENAN née Kouassi Mathilde Akissi	Spécialiste Suivi & Evaluation (S&E) PDDAA	MINADER
		Gbahi Djoua Luc	Statisticien	MINADER
	Gambia	Momodou Sowe	CAADP Focal Person	Ministry of food and agriculture
		Amet Sallah	CAADP Monitoring & Evaluation (M&E) Officer	Ministry of food and agriculture
		Sabina K Mendy	Statistician	Ministry of food and agriculture
	Ghana	Mr. Faisal Munkaila	Senior Agricultural Economist (Policy, Planning Divsion) (Representing the CAADP Focal point)	Ministry of Food and Agriculture
		Mr. Patrick Ofori	Deputy Director (Monitoring &Evaluation Division) (Representing M&E)	Ministry of Food and Agriculture
		Mr. Foster Azasi	Statistician (Representing the Statistics department)	Ministry of Food and Agriculture
	Guinea	Jean Luc Faber	Représentant le Point Focal PDDAA	Ministère de l'Agriculture
		Atigou Balde	Spécialiste Suivi & Evaluation (S&E) PDDAA	Ministère de l'Agriculture

		Mohamed Moustapha Camara	Chargé du rapport, en rapport avec les statistiques	Ministère de l'Agriculture
	Guinea-Bissau	Rui Jorge Alves da Fonseca	Point Focal PDDAA	Ministère de l'Agriculture et du Développement Rural
		Ildo Afonso LOPES	Directeur des Statistiques Agricoles	Ministère de l'Agriculture et du Développement Rural
		Suanda INFONDA	Technicien de la Direction de Suivi et Evaluation des Politiques et Programmes	Ministère de l'Agriculture et du Développement Rural
	Liberia	D. Musu B. Flomo Bendah	Director for Planning & Policy	Ministry of Agriculture
		Jlopleh Dennis Wiagbe. Jr.	Director, Monitoring & Evaluation	Ministry of Agriculture
		Peter W. Kun	Principal Data Analyst (Agriculture)	
	Mali	Mr. Abdoulaye Baba Arby	Point Focal PDDAA	Ministère de l'Agriculture
		Mme Korotoumou TOURE	Spécialiste Suivi & Evaluation (S&E) PDDAA	Ministère de l'Agriculture
		Mr. Aly KONE	Statisticien (Expert en statistiques agricoles)	Ministère de l'Agriculture
	Niger	Adamou Danguioua	Point Focal PDDAA	Ministre de l'Agriculture et de l'élevage
		Arimi Mamadou Elh. Ousmane	Spécialiste Suivi & Evaluation (S&E) PDDAA	Ministre de l'Agriculture et de l'élevage
		Neino Gondah	Chef de Division Gestion Base de Données, Informatiques et Archives	Ministre de l'Agriculture et de l'élevage
	Nigeria	Ibrahim Mohammed	Assistant Director (CAADP/NAIP Focal point)	Federal Ministry of Agriculture
		Dare Olorunmola	Statistician	Fmard
		muhammed adamu mele	ASST DIR (M & E)	PROJECT COORDINATING UNIT (PCU)OF THE FEDERAL MINISTRY OF AGRICULTURE AND RURAL DEVELO[PMENT
	Senegal	M. Ousmane SYLLA	Point Focal PDDAA	Ministère de l'Agriculture et de l'Equipement Rural
		Mme Ndeye Dibor NGOM	Spécialiste Suivi & Evaluation (S&E) PDDAA	Ministère de l'Agriculture et de l'Equipement Rural

		M. Moustapha NIANG	Statisticien	Ministère de l'Agriculture et de l’Equipement Rural
	Sierra Leone	Bai Bai Sesay	CAADP Focal Person	Ministry of Agriculture and Forestry
		Umaru Sankoh	CAADP Monitoring & Evaluation (M&E) Officer	Ministry of Agriculture and Forestry
		Mohamed Ajuba Sheriff (Dr)	Director of planning	Ministry of Agriculture and Forestry
	Togo	Alassani Ennardja	Point Focal PDDAA Directeur des Politiques, de la Planification et du suivi-évaluation (DPPSE)	Ministère de l’Agriculture, de la Production animale et Halieutique
		TAWO Kodjovi Orénykuwua	Ingénieur Agronome à la Direction des Politiques, de la Planification et du suivi-évaluation (DPPSE),	Ministère de l’Agriculture, de la Production animale et Halieutique
		AGOUDA Affèyitom	Statisticien à la Direction des Statistiques agricole, de l’informatique et de la documentation (DSID)	Ministère de l’Agriculture, de la Production animale et Halieutique

FAO RAP Regional Expert Consultation of the Food System Countdown Initiative´s Indicator Framework
Virtual Meeting, May 24

This report was produced by the FAO Regional Office for Asia and the Pacific to summarize the results of the FAO RAP regional expert consultation held on May 24, 2022.

1. Introduction

Food systems play a role in meeting all 17 sustainable development goals (SDGs). With less than a decade to achieve the SDGs, the global community faces a critical juncture to transform food systems to be healthier, safer, more sustainable, more efficient, and more equitable. Lately, the UN Food Systems Summit has focused the global attention on food systems and set the stage of food system transformation. Country and independent dialogues catalyzed the development of shared visions for food systems that apply to different contexts and geographies.

It is widely recognized that to deliberately change food systems and, at the same time, cover all aspects of food systems and their interactions, a clear, rigorous and comprehensive set of metrics and indicators are required to guide decision-makers while, at the same time, to hold them accountable. However, no rigorous commonly agreed mechanism yet exists to track the state of food systems, their change and performance over time.

In much of 2021, most countries, including from Asia and the Pacific held multi-stakeholder national dialogues and prepared and submitted national papers expounding on their country's vision and plans to implement agri-food transformation pathways in line with national priorities, needs and capacity. In fact, the commitments reached at the UN Food Systems Summit, and the realization of each nation's food systems vision reflected in the national pathways need metrics to guide decisions and track progress. At the same time, food system actors and stakeholders (e.g., civil society, governments, and international organizations) require trustworthy, science-based metrics and assessment to ensure measurable progress and accountability.

Within this context and the articulated need to fill this gap, the Food Systems Countdown Initiative ("the Initiative") was formed in 2021 as a comprehensive, independent, inclusive, science-based mechanism to provide actionable evidence to track progress, guide decision-makers, and inform transformation. At the same time, it intends to complement other monitoring mechanisms and the tracking of related goals at global and regional scales (i.e., SDG agenda, CAADP). The main goal of the Initiative is to provide an independent tracking and assessment system based on a high-quality, curated, parsimonious set of indicators that cover all important aspects of food systems and measure food system performance. The Initiative expects to deliver an annual assessment of the state of global food systems and their transformation, published in a peer reviewed scientific paper. It is also envisioned that policy briefs will be delivered in parallel for a broader audience and to facilitate transformative action.

To implement the Initiative an unparalleled partnership and collaboration has been put together, led by FAO, GAIN and John Hopkins University and with the participation of more than 50 scientists from nearly 30 academic institutions, non-governmental organizations, and UN agencies from nearly all continents. The Initiative/FSCI has designed an architecture for such a system from a multidisciplinary point of view and is moving towards implementation.

The first milestone of the Initiative was the publication of the initial proposed architecture of the system and the description of an inclusive process to move from the concept to its execution[7]. Parallel to this, an initial framework inclusive of indicative set of indicators was produced and published in a paper to serve as a starting point. As a second step, the Initiative will aim to an agreed set of indicators in each of the five thematic areas identifies and to deliver a first assessment of the state of global food systems that will serve as a baseline for monitoring progress and performance.

2. Objective of the Regional Expert Consultation

The Initiative is committed to an inclusive, consultative, and transparent process that will allow for validation and peer review of the set of indicators that will be used for the assessments. As part of that process, a regional consultation to engage with national and regional stakeholders and receive their feedback and input on the FSCI was held in Asia and the Pacific region through a virtual meeting on May 24.

The objective of the regional consultation on FSCI was to receive inputs from policymakers and policy-adjacent users of data, on the **relevance, usefulness, and the validity** of the proposed set of indicators from a regional perspective. The consultation covered the proposed indicators in each of the five thematic areas that will be used for the first assessment of the state of global food systems and later for tracking progress and assessing performance.

The regional consultation for Asia and the Pacific brought together representatives from governments in Asia and the Pacific, as well as individual experts in food systems from development partners and FAO networks in the countries. Participants included senior staff engaged in policy development from the relevant Planning Units of the Ministry, as well as senior experts from across the five thematic areas. It is also extended to additional relevant staff from other Ministries working on the thematic areas of the indicator framework. Also invited were the individuals and institutions most directly involved in the national dialogue meetings in 2021 as part of the UN Food Systems Summit.

The meeting started with a short plenary introduction with welcoming remarks by Mr Takayuki Hagiwara, Regional Programme Leader, FAORAP followed by a short overview on FSCI by Jose Rosero Moncayo, Director, Statistics Division, FAO (see full agenda of the meeting in annex).

This was immediately followed by three parallel breakout sessions of 90 minutes each focusing on the three domains:
1. Diets, nutrition and health;
2. Environment and climate change; and
3. Livelihoods, poverty and social inclusion).

The participants are then reconvened in a plenary session for another 90 minutes to discuss the two cross cutting domains
Governance
Resilience and sustainability;

Followed by a wrap up and conclusion session.

The thematic sessions focused on the capacity of the indicators proposed to guide policy decisions and promote accountability mechanisms. Discussion also focused on the relevance, usefulness, and validity of

[7] Fanzo et al. Viewpoint: Rigorous monitoring is necessary to guide food system transformation in the countdown to the 2030 global goals. Food Policy 2021; 104.

the proposed set of indicators from a regional perspective. Each thematic discussion was guided by the following set of framing questions:

What are the regional aspects we need to take into consideration in selecting and prioritizing a set of indicators to monitor the state of food systems and its evolution?.

Evaluate the proposed indicators in terms of:

- Relevancy; defined as their ability to measure something meaningful for food systems across a variety of settings, during relevant time periods?
- High quality, defined as using the best and most rigorous statistical methodologies and data available?
- Interpretable defined as having the ability to show a clear desirable direction of change, comparable across time and space, and easily communicated.
- Useful, defined as its ability to be used for policy and decision-making processes and by meeting actual information needs

What are the data gaps that you can identify?. Are these gaps structural? If not, are there some data or indicators that you know are available in your region and can be used for the purpose of this monitoring and assessment system?

3. Key session outcomes

3.1 DOMAIN 1: Diet, Nutrition, and Health

What are the regional aspects that should be taken into consideration when selecting the set of indicators to monitor the state of food systems and its evolution?

The discussion did not emphasized specific regional aspects that should be considered, except for aspects that alluded to non-communicable diseases (NCD) indicators for the Pacific and food safety from Asia.

On the questions of proposed indicators relevancy, quality, ease of interpretation or usefulness, the group discussed yielded the following insights:

There were questions about these indicators including a question about the **decision scenarios** regarding the indicators at each level (global, national and even subnational) and about drivers of consumption that influence diets (e.g. fuel and water insecurity, prices, etc.).

Another concerned raised was the link between this initiative and **existing relevant frameworks** (complement/duplicate efforts).

While the set of indicators were acknowledged to be relevant, interpretable, etc., there was also a concern about government capacity and resources including budget availability for governments to be able to implement them.

Another question raised was the link between FSCI indicators and **SDGs** and whether the indicators can be used to benchmark the performance of countries in terms of Food Systems achievements.

Another issue raised is about the **usefulness/application of these indicators in emergency and humanitarian situations**. One issue raised about the type of indicators that could be useful to reflect or measure cross-countries collaboration and coaching to improve nutrition, food policies, etc. FSCI experts

replied that the FSCI indicators retained left out issues like education or issues related to emergency and humanitarian situations.

One inquiry asked whether this set of indicators would be **built into existing surveys** like DHS or a new food systems survey.

Also pointed out was the need to think about **consumption indicators** that may not change over time, unless something drastic happens like disasters, or changes in food policies that would drastically affect the food system pathway. These indicators are important especially for children it was pointed out. While participants acknowledged the importance of these indicators, they raised the question of how to make these indicators as priority indicators at the country level.

What are the data gaps that you can identify? Are these gaps structural? Which data or indicators listed are available in your region and can be used for the purpose of this monitoring and assessment system?

<u>The discussion raised a number of points and issues:</u>

There were inquiries about the **monitoring of nutrition status**, including wasting of children, obesity and so on. These however were expletively excluded from the set of FSCI indicator, as they are already well established.

Also, participants asked about indicators to access adequacy of energy intake, macronutrients and selected micronutrients intake. In addition, it was emphasized the need to consider some **policy measure indicators** that can act as a driver in the transformation of food systems and influence health outcomes such as availability of policies/ legislation on unhealthy food marketing and taxation, among others.
A government representative inquired about the inclusion of **cognitive development in young children 0 - 16 years** that relates to diet, nutrition and health.

Another question was related to whether it would be possible to include **indicators to map out social, cultural norms and personal factors that influence diets and food environments**.
A government official inquired about how the proposed indicators would take into consideration the challenges brought about by the **pandemic**. The importance of the topic was acknowledged by FSCI, but the difficulties were noted.

Another intervention the need to think about some **indicators on consumption** that may not change over time unless something drastic happens like disasters, or changes in food policies that will drastically affect the food system pathway. These indicators are important especially those for children, and can be used as markers on the quality of food intake of children.

A participant underlined the possibility of taking into account **production, processing and logistics (post-harvest) factors** critical to ensuring food security and nutrition. Factors in the environment, including the involvement of NGOs and other facilitators were also pointed out. The FSCI team acknowledged the pertinence of these issues, particularly infrastructure [along the agrifood value chain].
An attendee raised the issue of food safety and asked whether there is an indicator that measures import and export of prohibited chemical fertilizers. This tends to happen in countries with weak policy enforcement environment.

A Government official recommended including a **specific indicator on food safety** as a crosscutting indicator. She also echoed the interventions of some participants on monitoring education in food systems

including on safe food, better choice of food, etc. The difficulty with such indicator is capturing the data as these indicators are multiclausal and difficult to measure.

Another proposal from a Ministry official was to consider using the inclusion of an **indicator regarding to the percentage of dietary energy intake from ultra-processed food** using the NOVA classification, in light of the abundance of unhealthy ultra-processed food in the market.

A government official recommended including **NCD indicators**, noting that they are very common in the Pacific. Another recommendation was to consider the fact that different countries have different capacities to measure indicators and collect the data.

A participant recommended including under the food environment set of indicators, one related to the **volatility of food prices/food price regulation**, given their significant impact on food security and nutrition. She also mentioned the existence of data gaps in the Philippines regarding food losses and waste (FLW). In a later intervention, the same participant noted the importance of disaggregating the data on animal food consumption in terms of fish, seafood and others. FSCI team acknowledged the importance of taking into account food price regulations, understanding the trade-offs (farmers vs consumers), and taking account of their typical short notice implementation on food systems. Also FLW was acknowledged to be a major oversight in current FSCI indicators. However, data for the proposed indicators may not be available for all the countries.

Additional questions raised by the session participants include:

- The proposed indicators are mostly related to assess the practices related, what about the knowledge related indicators?
- Can we include cognitive development in young children 0 - 16 years that relates to diet, nutrition and health? Not the only reduction of malnutrition, stunting, and wasting but importantly on the total human development including the education and academic excellence in younger population?
- How will these indicators take into consideration the challenges brought about by the pandemic?
- How the FSCI indicators will be in line with SDGs (as some indicators also in SDGs)? Will the FSCI indicators be used to compare the country achievement on Food Systems?
- Would it be possible to include indicators to map out social, cultural norms and personal factors that influence diets and food environments.
- How about indicators to access adequacy of energy intake, macronutrients and selected micronutrients intake. Considering abundant of unhealthy ultra-processed food in the market, is recommended to access the % of dietary energy intake from ultra-processed food using the NOVA classification.
- Considering the effects of the pandemic on food supply and access (logistics particularly) it will really be helpful to know the extent and what can be done (decision scenarios) if this global situation persists
- Need to take into account countries different capacities to measure indicators and collect the data.
- Other possible indicator to access food environment - Percentage of monthly income spent on food.
- What kind of indicators can be useful to reflect or measure cross-countries collaboration and coaching to improve nutrition, food policies, and system?
- Monitoring education on food system including on safe food, better choice of food, etc.
- Will the FSCI made up of "parsimonious" set of indicators would be built into existing surveys like DHS? or a new food systems survey?
- Consider policies that measure indicators which can act as a driver in the transformation of food systems and influence health outcomes such as availability of policies/ legislation on unhealthy food marketing, taxation and etc.

3.2 Domain 2: Environment and Climate
This session covered a number of topics related to pollution, land and soil, water, biodiversity, climate and issues of implementation of indicators

Pollution
1. Pesticides – HHP
2. Risk-based approach and indicators – not just amount
3. Track hazardous pesticide use in terms of availability of such pesticides per unit. This unit can be crop/soil type/prevalent pests. Standard indicators:
a. Chronic Toxicity needs to be included
Acidification of land
The new indicator should be split into three different segments: X, Y, Z
Collapse of wild bees, and lady bird populations, would be good indicators of hazardous pesticides
Link with health - deaths from pesticide poisoning
4. Pollution and waste (no indicators defined for this, only pesticides) but it needs attention, otherwise it is not really a systemic indicators.
5. Crop residue management
6. Animal health - use of antibiotics is missing.

Land – Soil
Respondents indicated the importance to track not just crop land but also pasture land under different soil types
Overgrazing - temporary migration due to unavailability of grazing land for cattle - negatively affects the local ecosystem. It would be helpful to have an indicator that captures this interlinkage, perhaps in the cross cutting session
Climate induced migration – Look at WG3 and Cross cutting domain
Land productivity: Percentage of production or ag land under organic/sustainable cultivation (another difficult one but essential)
Soil losses and erosion - impact on land productivity (GLADA last updated in 2011 and may be out of date - what measures are regularly updated)

Water
Transboundary water dependence (there is some data on this, but may be difficult to separate the ag use from other)
Water use efficiency and water balance: extremes climatic events also include floods and sea-level rise that could impact on soil fertility and salinity wash-off surface soils.
How can we better measure irrigation performance for land productivity/food production per hectare

Biodiversity
Are the biodiversity intactness and fisheries health index indicators sufficient?
Consider ways to capture aquatic species loss
Monitoring of ecosystem diversity to better understand impacts on human health (Zoonoses)

Climate
GHG intensity indicators (if looking at intensity against Ag value added - needs careful consideration as when food prices go up dramatically, as they are, then intensities will go down)
"% change in soil organic carbon", is it anticipated to have synergies with the 4per1000 initiative?
Climate extremes and adaptation indicators – data and timescale for monitoring as climate extremes come up frequently – Look at Resilience domain
Overall - Implementation

It would be helpful to show clearer links between suggested indicators and relevant SDG target indicators and related commitments such under the UNFCCC Paris Agreement, UNCBD and others
Experts will share further ideas on possible indicators and data
Regional collaboration
Standardizing agreed measures of food system health to encourage learning and cooperation (but not government responses - no-one-size fits all approach)

Additional comments from participants in the environment and climate session:

- What would be the working modalities for this initiative be implemented at regional cooperation framework?
- A regionally useful set of indicators should look at 1. crop residue management 2. transboundary water dependence (there is some data on this, but may be difficult to separate the ag use from other) 3. pollution and waste (no indicators defined for this, only pesticides) but it needs attention, otherwise it is not really a systemic indicators. 4. a link with health - deaths from pesticide poisoning could be treated here, rather than in the diet and health rubric (?). 5. GHG intensity indicators (if looking at intensity against Ag value added - needs careful consideration as when food prices go up dramatically, as they are, then intensities will go down) 6. Animal health - use of antibiotics is missing. 7. percentage of production or ag land under organic/sustainable cultivation (another difficult one but essential) (ESCAP)
- On water use, extremes climatic events also include floods and sea-level rise that could impact on soil fertility salinity wash-off surface soils. Please consider also water use efficiency and water balance
- In regards to the indicator on "% change in soil organic carbon", is it anticipated to have synergies with the 4per1000 initiative for instance?

3.3 Domain 3: Livelihood, poverty and equity

What are the regional aspects that should be taken into consideration when selecting the set of indicators to monitor the state of food systems and its evolution?

It was generally agreed that the regional and country level aspects and differences need to be taken into account when considering the list of proposed indicators.

Countries are at different levels in terms of food systems (FS) monitoring, with some countries already with developed monitoring frameworks with a set indicators and others working towards the development of monitoring frameworks and collection of baselines.

The difference in country context was highlighted in the **Philippines** experience whereby it was noted that some of the indicators were already ones used in the country's already established monitoring system, but that there were some indicators that were not included in their current framework which could be looked into for possible inclusion.

Tonga on the other hand is keen to review the indicators as part of its formulation of a monitoring framework for its national FS transformation.

Therefore, countries may use the list depending on specific country situations, and indicators can be adapted to the country's own specific issues.

A participant pointed out that countries may find it challenging to produce reporting on these indicators, and that we need to assess the feasibility of reporting against these indicators.

Specific Recommendations

A participant asked whether the indicators covered '**off farm aspects of food systems'**. He noted that, across Asia, although the agriculture share of GDP is decreasing, the share of food enterprises (food processing, food selling and wholesale) is rising in GDP contribution. He noted that there were no **indicators covering employment and incomes of people in food enterprises**, and given this is an aspect of the food systems, indicators covering 'off farm' aspects should also be included. Data can probably be found in food enterprise surveys.

Also noted in areas such as South Asia, **gender aspects** are indeed important when looking at different indicators, not just in land ownership but we also need to consider **inequities such as the wage gap between men and women**. In addition to gender aspects, other areas of **inequalities** need to be captured such as **deprived communities**.

A participant proposed to consider an indicator to determine **youth participation** in Food Systems transformation.

A participant from FAO noted that indicators capturing **inclusiveness** are missing and that indicators on **access to finance and technology** should also be considered given the key role these two aspects have in food systems transformation.

A Government representative emphasized that **migration** plays a critical role in this process including on livelihoods. He mentioned that indicators should take into account the increasing number of migrant workers (e.g. seasonal, those in food insecurity), looking into rights and protection angles of these workers, as well as the laws and regulations concerning migration (immigration) and these workers (e.g. health, job security, etc). For specific indicators in identifying the baseline, there could be potential of migration in the relevant areas.

Related the questions of indicators relevancy, quality, ease of interpretation and usefulness, the following discussion points were captured:

- It was generally agreed that the list of indicators was relevant. However, working definition of each indicator is dependent on the country context.
- A government representative confirmed that these indicators are all relevant, but that, in terms of the definition of each indicator, the indicators need further elaboration. In particular:
- Income is relevant, as his country is doing the best on the national household savings as well as on household income expenditure survey (agricultural households).
- Population is not yet in the list, but this aspect can be looked at.
- In terms of other indicators related to income and poverty, they can consider other aspects and factors of poverty.
- Employment and social protection (such as school feeding programmes) are relevant, and can depend on the existing data (e.g. on agricultural workers).
- Indicators related to rights are currently not in the list of indicators of the country, but they can also look at this aspect. Rights to land tenure are included in the list and covered already.

In relation to questions on data gaps, and data or indicators available in the region, the discussion noted the following points:

It was confirmed that **data availability** is an important issue to be looked at. We also need to look at the feasibility and consistency in data availability across the countries. There is no one single solution to this issue.

A Ministry official, informed the meeting that right now the issue of data and monitoring is high on the agenda in his country. They are working to develop a baseline data to measure food systems transformation including measuring baseline for livelihood and employment in the food systems. He emphasized the need to improve the **capacity for collecting data**. It was noted that, with the indicators, there is a need to collect data and therefore capacity to collect data is important in considering the indicators to be adopted.

Additional feedback from session on livelihood, poverty and equity:
Would it be useful to highlight crucial aspects of food systems on which countries are not collecting data? The number of the proposed FSCI indicators for livelihoods poverty and equity which is 11. It would be a helpful menu from which countries can base their specific indicators and further apply these in their own ongoing monitoring and assessment and method/s of measuring and tracking. Certainly, there are some nuancing on country to country.

3.4 CROSS CUTTING DOMAINS: GOVERNANCE AND SUSTAINABILITY

Key feedback from plenary session Governance and Resilience Domains:

- For small countries, the set of monitoring data presented are quite complex and technically challenging requiring external technical assistance to collect and monitor relevant data.
- There are also institutional challenges in how to capture these data sets for some countries
- On resilience, consider integrating entire logistics system apart from road density when it comes to calamities/pandemic.
- Globalization is also posing a danger to decreasing resilience of villages
- The list of proposed indicators has their equivalencies in countries and the criteria used to list these down may also be applied differently across countries depending on those relevant to their situations.
- This FSCI Initiative is laudable but may find even more utility depending on what each country is already measuring and what else they could consider assessing and tracking, regarding their food systems, if not yet.
- The indicators for both resilience and governance should be directly linked to the component parts of the food system - these could be organized into two categories - those internal to the system (production, processing, supply chain, consumption...) and external to the system that affects the efficacy of food system.
- In the Pacific we need specific development to identify relevant data sets that we can use to monitor food systems. Similar issue to SDG where some of the indictors in the FSCI matrix are very complicated and are very hard for us (in the Pacific) to access and understand
- There is need for further discussion on datasets that we can use to monitor food system development in Pacific countries starting from baseline data where we are now in terms of food system development
- There is a need for closer collaboration between food system coordinators and National Statistics Office. Not all indices presented are applicable for all, each country can contextualize what is relevant. Not all indicators require quantitative datasets, some success stories and qualitative info may suffice.
- In South Asia, the most politically sensitive crops are Onion, potato and tomato and are important part of food system. All the three are prone to climate change and require dedicated analysis.
- Similarly the ratio between area, production, productivity should be a good indicator of which direction the fulcrum is moving

- There has to be a critical look at elasticity vs volatility of commodity prices under the influence of extreme events such as severe precipitation etc. This will be a measure of resilience vs threat analysis from Climate induced changes.

AGENDA AND TIMELINE

The event took place virtually according to the following agenda and timeline:

Timeline:

Session	Pakistan	India	Bangkok	China/Mongolia	Japan	Australia	Apia, Samoa
Opening	7:00 – 7:30	7:30 – 8:00	**9:00 – 9:30**	10:00 – 10:30	11:00 – 11:30	12:00 – 12:30	15:00 – 15:30
BOS 1,2,3	7:30 – 9:00	8:00 – 9:30	**9:30 – 11:00**	10:30 – 12:00	11:30 – 13:00	12:30 – 14:00	15:30 – 17:00
Break	9:00- 9:15	9:30- 9:45	**11:00- 11:15**	12:00- 12:15	13:00- 13:15	14:00- 14:15	17:00- 17:15
Plenary	9:15 – 10:30	9:45 – 11:00	**11:15 – 12:30**	12:15 – 13:30	13:15 – 14:30	14:15 – 15:30	17:15 – 18:30
Wrap up	10:30 – 11:30	11:00 – 12:00	**12:30 – 13:30**	13:30 – 14:30	14:30 – 15:30	15:30 – 16:30	18:30 – 19:30

AGENDA:

**Time**	**Subject**	**Speakers**
9:00 -9:30	Plenary: **Welcoming remarks and introduction to the Initiative**<br><br>Welcoming remarks, **Takayuki Hagiwara**, Regional Program leader, FAO RAP<br><br>Introduction to the Food Systems Countdown Initiative, **Jose Rosero Moncayo**, Director, Statistics Division, FAO<br><br>Opening	*FAO*
9:30-11:00	Break-out (BO) Sessions (parallel)<br><br>**<u>Break out Session 1</u>: Diet, Nutrition, and Health**<br>Moderator: **Joseph Nyemah**<br>Rapporteurs: **Eva GalvezNogales, Rosemary Kafa**<br><br>Discussion opener (**Kate Schneider**): Brief introduction to candidate indicators (10 minutes)<br><br>Open floor interventions- feedback from participants	*Moderator*<br><br>*Discussion starter*<br><br>*Participants*
	**<u>Break out Session 2</u>: Environment and climate Domain**<br>Moderator: **Hang Pham, Malia Talakai**<br>Rapporteur(s): **Beau Damen, Caroline Turner**<br><br>Discussion opener (**Mario Herrero**): Brief introduction to thematic indicators (10 minutes)<br><br>Open floor interventions- feedback from participants	*Moderator*<br><br>*Discussion starter*<br><br>*Participants*

	<u>**Break out Session 3:**</u> **Livelihoods, poverty and equity Domain**<br>Moderator: **Aziz Elbehri, Fiasili Lam**<br>Rapporteur: **Kae Mihara**<br><br>Discussion opener (**Jikun Huang**): Brief introduction to thematic indicators (10 minutes)<br><br>Open floor interventions- feedback from participants	*Moderator*<br><br>*Discussion starter*<br><br>*Participants*
11:00-11:15	Break	
11:15-12:45	Plenary: **Governance and Resilience and sustainability Domains**<br><br>Moderator: **Aziz Elbehri**<br>Discussant opener (Governance – **Danielle Resnick**) (10 Min)<br>Discussant starter (Resilience **- Yuta Masuda**) (10 min)<br><br>Open floor for participants interventions, feedback and discussion	*Moderator*<br><br>*Discussion starters*<br><br>*Participants*
12:45-13:30	Plenary: **Summary and wrap up**<br><br>Moderators of BO session 1, 2 and 3 to present highlights of their sessions<br><br>15 minutes overall wrap up on what was discussed, key findings and follow up<br>**Lawrence Haddad**, GAIN<br><br><br>Closing remarks from FAO RAP	*Moderators*<br><br>*FSCI members*<br><br>*RAP-RPL*

List of participants

Last Name	First Name	Organization	Country
Abbas	Touseef	UNICEF	PNG
Abella	Ellen Ruth	National Nutrition Council	Philippines
Agipar	Bakyei	Mongolian University of life Science	Mongolia
'Aholelei	'Isileli	Tonga Ministry of Agriculture, Food and Forest	Tonga
Ali	Pungkas	Bappenas	Indonesia
Arya	Aziz	FAO/UN	Thailand
Aye	Myint Myint	FAO	Myanmar
Azma	Nurul	Bappenas	Indonesia
Banura	Hitomi	Ministry of Agriculture, Forestry and Fisheries	Japan
Battulga	Altanshagai	UN FAO	Mongolia
Bicchieri	Marianna	FAO	Thailand
Bolima	Dean Marc	Department of Agriculture	Philippines
Boonchan	Surasak	FAO	Thailand
Chand	Ramesh	NITI Aayog	India
Chatrurvedi	Siddharth	Bill & Melinda Gates Foundation	India
Conda	Angel Mae	Department of Agriculture	Philippines
Da Cruz	Gil Rangel	Ministry of Agriculture and Fisheries	Timor-Leste
Damen	Beau	FAO	Thailand
Duran	Tamara	FAO	Philippines
Enkhtur	Anudari	FAO Mongolia	Mongolia
Estoesta	Alexander II	Department of Agriculture	Philippines
Fatmaningrum	Dewi	FAO	Indonesia
Felipe	Judi Anne	DA- Planning and Monitoring Service	Philippines
Ferrand	Pierre	FAO	Thailand
GalvezNogales	Eva	FAO	Thailand
Guterres	Maria Odete do Ce	Ministry of Agriculture and Fisheries	Timor-Leste
Haas	Linda	reu	Hungary
Haddad	Lawrence	GAIN	Switzerland
Hagiwara	Takayuki	FAO	Thailand
Huang	Jikun	Peking University	China
Hunter	David	Ministry of Agriculture and Fisheriess	Samoa
Iuvale	Soo Jr	MAF	Samoa
Jack	Randon	Ministry of Natural Resources and Commerce	Marshall Islanc
Jayanetti	Nandalal	Ministry of Agriculture	Sri Lanka
Kafa	Rosemary	United Nations FAO	Thailand
Kafle	Samjhana	Ministry of Agriculture and Livestock	Nepal
KAPUR	RAKESH	IFFCO	India
Khaine	Aye Aye	FAO	Myanmar
K-hasuwan	Prae-ravee	Ministry of Agriculture and Cooperatives	Thailand
kim	soojung	KOICA	Mongolia
Kishore	Avinash	IFPRI	India
Krishna	Gopal	ICAR CIFE	India
Kumwilai	Usakorn	The Queen Sirikit Department of Sericulture	Thailand
Lam	Fiasili	FAO	Samoa
Latifi	Muhebullah	FAO	Afghanistan
Lee	Nara	FAO	R.O. Korea
Lee Hang	Keyonce	Ministry of Agriculture and Fisheries	Samoa
Locke	Dan	FAO	Thailand

Magtibay	Jasmine	FAOPH	Philippines
Magyaya	Ariana	Department of Agriculture	Philippines
Mandal	Bir	FAO	PNG
Manu	Viliami	Ministry of Agriculture, Food and Forests	Tonga
Marothia	Dinesh	Indian Society of Agricultural Economics	India
Marte	Karen	Department of Agriculture	Philippines
Masuda	Yuta	The Nature Conservancy	United States
Matatumua	Taimalietane	Ministry of Agriculture and Fisheries	Samoa
Menon	Purnima	IFPRI	India
Mihara	Kae	FAO	Thailand
Mishra	Rajendra	Government of Nepal, MoALD	Nepal
Moon	Dooree	KOICA	Mongolia
Mumtaz	Amer	Pakistan Agricultural Research Council	Pakistan
Munir	Muhammad	FAO	Myanmar
Munkong	Tipaporn	The Queen Sirikit Department of Sericulture	Thailand
Munoz	Hernan	FAO	Italy
Myazoe	Walter	Ministry of Natural Resources and Commerce	Marshall Islanc
Nallur	Krishna Kumar	ICAR	India
Naz	Farrah	Global Alliance for Improved Nutrition (GAIN)	Pakistan
Neupane	Sumanta	International Food Policy Research Institute	United States
newman	scott	FAO	Thailand
Ngarmtab	Poranee	FAO	Thailand
Nkoroi	Alice	UNICEF	Philippines
Nyemah	Joseph	FAO	United States
On-ubol	Woranut	Ministry of Social Development and Human Sec	Thailand
Panda	Ranjan	Water Initiatives	India
Pastores	Maria Cecilia	FAO	Philippines
Pechcho	Pachirarat	Department of Agricultural Extension	Thailand
Peixoto de Lima	Camilla Hellen	FAO	Thailand
Pham	HangThiThanh	FAO	Thailand
Pioneta	Joyce Mae	Department of Agriculture	Philippines
Piyawatpaphad	Pongpakorn	Cooperative promotion department	Thailand
Plianphanich	Ms. Phatthicha	Department of Agriculture	Thailand
Prasula	Paulina	FAO	Italy
Puana	Ilagi	NAQIA	PNG
Rahmani	Shahriar	FAO	null
Rana	Jai	Alliance of BI nd CIAT	India
Rankine	Hitomi	ESCAP	Thailand
Rasool	Faiz	Global Alliance for Improved Nutrition (GAIN)	Pakistan
Resnick	Danielle	Brookings	United States
Rosero Moncay	Jose	FAO	Italy
Rufus	Lajikit	Ministry of Natural Resources and Commerce	Marshall Islanc
Sainzaya	Batbayar/Johny	the world vision in Mongolia	Mongolia
SAMSON	MARIVIC	NATIONAL NUTRITION COUNCIL	Philippines
Schneider	Kate	Johns Hopkins University	United States
Scott	Samuel	IFPRI	India
Shaheen	Aslam	Planning Commission	Pakistan
Sharav	Nandinzaya	KOICA	Mongolia
Sharma	Inoshi	Food Safety & Standards Authority of India	India
Shivakoti	Sabnam	Ministry of Agriculture and Livestock Developm	Nepal

Shrestha	Ram	Ministry of Agriculture and Livestock Developm	Nepal
Sinclair	Chris	Ministry of Agriculture & Fisheries	Samoa
Singh	Nishmeet	IFPRI	India
Singh	Sunil	NPC	India
Singh	Lt.Col.(Dr) Baljit	National Cooperative Development Corporatio	India
Sinha	Alok	VillageNama	India
SJ	Balaji	Indian Council of Agricultural Research	India
Smektala	Isabelle	French Embassy	Mongolia
Sui	Hluan	FAO Myanmar	Myanmar
Talakai	Malia	FAO	Samoa
Tienpati	Supajit	FAO	Thailand
Tonga	Semisi Latu	Department of Agriculture	Tuvalu
Tripathi	Bhupendra Nath	Indian Council of Agricultural Research	India
Tupou	Siutoni	MAFF	Tonga
Turner	Caroline	FAO	Thailand
Vainikolo	Leody Eleutilde	Ministry of Agriculture, Food and Forests	Tonga
Varma	Anupam	Indian Agricultural Research Institute	India
Voratira	Yowanat	FAO	Thailand
Wani	Suhas	Formerly with ICRISAT, Current with FAO	India
Win	Le Le	FAO	Myanmar
Wongkanit	Napaporn	Agricultural Land Reform Office	PNG
Yoovatana	Dr. MARGARET C.	Department of Agriculture	Thailand

FAO REU Regional Expert Consultation of the Food System Countdown Initiative's Indicator Framework
Virtual, May 25, 2022

This report was produced by the FAO Regional Office for Europe and Central Asia to summarize the results of the FAO REU regional expert consultation held on May 25, 2022.

Introduction:

Food Systems Countdown Initiative – Regional Expert Consultation
The Initiative is committed to an inclusive, consultative, and transparent process that will allow for validation and peer review of the set of indicators that will be used for the assessments. As part of that process, two efforts are envisioned. First, a consultation with expert scientists and, second, a series of regional expert consultations across the FAO regions.

Objective and outcomes
The objective of the FAO REU regional expert consultation was to bring an expert point of view from policymakers and policy-adjacent users of data, on the relevance, usefulness, and validity of the proposed set of indicators from a regional perspective and, finally, on the potential data gaps and resources. The consultation covered the proposed indicators in each of the five thematic areas. These will be used for the first assessment of the state of global food systems and later for tracking progress and assessing performance.

The regional expert consultation was an opportunity to provide inputs, comments, and suggestions on the proposed indicators both in terms of appropriateness as well as also feasibility related to the data availability. The Initiative proposes a monitoring framework. This framework is not mandatory, however, the consultations will ensure that it has the capacity to be a useful tool for policy decision-making processes.

The Initiative expects to deliver an annual assessment of the state of global food systems and their transformation, published in a peer-reviewed scientific paper (October 2021). It is also envisioned that policy briefs will be delivered in parallel for a broader audience and to facilitate transformative action.

The first milestone of the Initiative will be the publication of the initially proposed architecture of the system and the description of an inclusive process to move from the concept to its execution. The architecture covers the five thematic areas of diet, nutrition and health; environment and climate; livelihoods, poverty and equity; governance and resilience and sustainability (October 2022).
As a second step, the Initiative will aim to deliver the set of indicators in each of the five thematic areas above and to deliver a first assessment of the state of global food systems that will serve as a baseline for monitoring progress and performance. A report is expected to be published in October 2023 in conjunction with the UNFSS review mechanism to assess the status and performance of the food systems transformation path. In 2029, a Global Food Systems Conference will take place. "1 year left to achieve SDGs".

Participant's general profile
The regional experts' consultation for Europe and Central Asia brought together representatives from governments in the region, as well as individual food system experts.

Recognizing the complexity of the food systems challenges and actions, the participants were invited through a multidisciplinary, multi-stakeholder approach, while ensuring equitable geographical and gender-balanced representation. It enabled to capitalize on the expertise of regional and country representatives, who were nominated experts on the following aspects of food systems:

- Nutrition, food security, health;
- Environment and climate;
- Resilience and sustainability;
- Rural development, social protection and poverty alleviation.

Participants were mainly senior experts from the public sector, engaged in policy development, UNFSS National Convenors, statisticians, or data specialists.

The Regional Consultation also capitalized on the expertise of several UN agencies as an important element of the 2030 Agenda. In addition to FAO colleagues, some representatives of the Regional Issue-based Coalition on Sustainable Food Systems (IBC-SFS) - namely UNECE, UNICEF, WFP and WMO – as well as World Bank also joined the conversation.
In total, 83 participants joined the consultation from 18 countries. The number of participants in different breakout sessions was the following:
Nutrition and diets: 30
Environment: 22
Livelihoods: 26
Governance: 25
Resilience: 38
Regarding the geographical representation, most of the participants came from FAO program-countries in the region, but EU Member countries were also represented.

List of the countries participating in the FAO REU Regional Expert Consultation of the Food System Countdown Initiative's Indicator Framework:

Albania
Armenia
Belarus
Bosnia and Herzegovina
Finland
Georgia
Hungary
Iceland
Montenegro
North Macedonia
Republic of Moldova
Serbia
Tajikistan
Turkey
Ukraine

Working arrangements

The structure of the regional expert consultation was similar to other FAO regions.

The organization of the meeting was led by Raimund Jehle, Regional Programme Leader, Deputy Regional Representative – Programme, Regional Office for Europe and Central Asia, and Mary Kenny, Food Safety and Consumer Protection Officer and the Regional Initiative Coordinator on Transforming food systems and facilitating market access and integration. The FAO REU meeting Secretariat included Aniko Nemeth, Food Safety and Nutrition Expert, Valeria Rocca, Regional SDGs Advisor, Klaudia Krizsan, Food Safety and Nutrition Junior Technical Officer, Valentina Gasbarri, Knowledge Management and Communication Specialist, and Linda Haas, Office Assistant.

The rationale and objectives of the consultations were introduced by Hernan Munoz, Statistician, FAO Statistics Division, and Lawrence Haddad, Executive Director, GAIN, Co-chair of the Food Systems Countdown Initiative while the closing sessions of the meeting was supported by José Rosero Moncayo, Director of the Statistics Division, FAO and Jessica Fanzo, PhD, Bloomberg Distinguished Professor of Global Food & Agricultural Policy and Ethics, as Co-Chairs of the Initiative.
Methodology
A short video introducing all the panelists was broadcasted at the beginning of the event.

A general overview on the food systems transformation in the Europe and Central Asia region and the brief introduction of the Food Systems Countdown Initiative were provided in the plenary.

The five breakout thematic sessions covered the domains of the indicator framework, namely:

Session 1: Diets, Nutrition and Health Domain;
Session 2: Environment and climate Domain;
Session 3: Livelihoods, poverty and equity Domain;
Session 4: Governance Domain;
and Session 5: Resilience and sustainability Domain.

In each session, the discussion was stimulated by an opening presentation of the experts of the Food Systems Countdown Initiative on the proposed set of indicators. The discussion was facilitated by Moderators from the FAO Regional Office in all breakout sessions. The Moderators were all technical experts on the topic of the session. The reporting process was supported by Note Takers, Rapporteurs and the Moderators (See Table 1).

Table 1. List of Working Groups, FSCI Experts, Moderators/Facilitators and Note Takers

	**Topic**	**FSCI Expert**	**Moderator/Facilitator FAO REU**	**Note Takers, FAO REU**
**WG1**	Diet, nutrition and health	Musonda Mofu, Public Health Nutritionist and Director for the National Food and Nutrition Commission of Zambia	Keigo Obara, Food Security Officer	Klaudia Krizsan, Food Safety and Nutrition, Junior Technical Officer
**WG2**	Environment and climate	Fabrice DeClerck, Science Director of EAT and a Senior Scientist with One CGIAR	Anna Kanshieva, Biodiversity Expert	Virag Nagypal, Climate Change and Natural Resource Management Junior Technical Officer
**WG3**	Livelihoods, poverty and equity	Alejandro Guarin, Researcher at the International	Anna Jenderedjian, Gender and Social Protection Specialist	Anetta Szilagyi, Rural Development Consultant and Ildiko Buglyo, Assistant to the Forestry and Land Tenure Officers

		Institute for Environment and Development (IIED)		
**WG4**	Governance	Namukolo Covic, Nutritionist with the Health Professions Council of South Africa	Mary Kenny, Food Safety and Consumer Protection Officer and the Regional Initiative Coordinator	Gokce Akbalik, , Food Safety Consultant
**WG5**	Resilience and sustainability	Preet Lidder, Technical Adviser to the Chief Scientist, FAO	Pedro Arias, Economist	Fanni Zsilinszky, Agrifood Policy Junior Technical Officer

The breakout sessions' discussions focused on the regional specificities and aspects that should be taken into consideration when selecting the set of indicators to monitor the state of food systems and its evolution capacity of the proposed indicators to guide policy decisions and promote accountability mechanisms.

Opening:
Summary of Welcome Remarks

The opening remarks by Raimund Jehle, Regional Programme Leader, Deputy Regional Representative, FAO Regional Office for Europe and Central Asia, highlighted the importance of keeping the momentum around sustainable food systems created by the UN Food Systems Summit in 2021 and its follow up mechanism at the global, regional national level. He reiterated that food systems related issues have also been put on the spotlight by the COVID-19 pandemic and, more recently, by the ongoing war in Ukraine, as reaffirmed during the 33rd Session of the Regional Conference for Europe in Łódź, Poland, where 50-plus FAO Members reaffirmed its commitments to ensuring food-secure, sustainable and inclusive agrifood systems, while, most importantly, pursuing peace. This is why food systems and their interactions require a clear, rigorous, and comprehensive set of metrics and indicators, to guide decision-makers and to hold them accountable, such as the Food Systems Countdown Initiative.

He highlighted that food system assessment is not an easy task, because of the complexity of the system and that this informal consultation is part of a range of work ongoing – such as a number of global, regional and national mechanisms that have been established to support the UN Food Systems follow- up. Such mechanisms include the UNFSS Coordination Hub, the UNFSS Coalitions, the national teams working with the national convenors on the implementation of national pathways and the work of the Issue-based Coalition on Sustainable Food Systems, a regional UN mechanism supporting the UN Country Teams in the region on the work on sustainable food systems.

"Many decisions we could make, whether we are producers, transporters, retailers…could bring us toward the achievement of the SDGs outcomes or away from SDGs. Majority of decisions will bring us away from SDGs", said **Lawrence Haddad,** Executive Director, GAIN; co-chair of the Food Systems Countdown Initiative during his keynote speech at the opening of the FAO REU regional Consultations.

Food systems play a role in meeting all 17 sustainable development goals (SDGs). With less than a decade to achieve the SDGs, the global community faces a critical juncture to transform food systems to be healthier, safer, more sustainable, more efficient, and more equitable.

He added: *"Food systems must be on the policy agenda for the coming years. Countries are thinking about food systems now.*
We hope they will develop national monitoring mechanisms, in line with the Countdown Initiative".

It is widely recognized that to enhance all aspects of food systems and their interactions, a clear, rigorous, and comprehensive set of metrics and indicators are required to guide decision-makers and to hold them accountable. However, no rigorous mechanism currently exists to track the state of food systems, their change, and performance over time.

Presentation of Themes
The themes were introduced by members of the Initiative.
Session 1 on Diet, Nutrition and Health Domain was presented by Musonda Mofu. In this theme, several indicators related to diet, nutrition and health were discussed by looking at the various areas which measure quality of diet, including nutrient adequacy and dietary risk factors for NCDs. As well as measuring the other factors affecting diet quality, namely the accessibility to healthy diets (food security); and a focus on the food environment (availability, affordability, messaging, and food & vendor properties). Finally, examining whether policies contribute positively or negatively towards food availability, food access, and product properties (policies affecting food environments).

Session 2 on Environment and climate Domain was presented by Fabrice DeClerck. This theme focused on the relation between food systems and the environment. The indicators focused on the main environmental systems and processes which interact with food systems: land use, climate, water use, biosphere integrity, and pollution (e.g., biogeochemical flows/novel entities). The indicators cover components and processes providing essential environmental services for the environment and humanity. As well as focusing on the areas where food systems can achieve the necessary change.

Session 3 on Livelihoods, poverty and equity Domain was presented by Alejandro Guarin. The indicators for this theme monitor the transformation created by food systems for the numerous people who work as part of the food system, in rural and urban areas, and in high and low-income countries. It focused on the areas of poverty and income, employment, social protection and rights. Cross-cutting issues were also pointed out: governance and resilience and sustainability.

Session 4 on Governance was presented by Namukolo Covic. The Governance Theme looks at how governance of the food systems domain can foster alignment and coherence across different food system actors, their activities, and progress toward results. It aims to monitor the shared vision of the outcomes, the relevant policy instruments to align efforts, the implementation of resources, and accountability for the outcomes.

Session 5 on Resilience and sustainability was presented by Preet Lidder. The theme of Resilience and sustainability was presented as a transversal aspect of the transformation of agri-food systems with a multi-dimensional interpretation. It covered the domains of exposure to shocks, resilience capacities, agrobiodiversity, food security stability, food system sustainability index. Resilience and sustainability are critical for food and nutritional security. They are also critical for other functions such as being a precondition for sustainability, and critical to tackling poverty and livelihoods issues.

Discussion
This section synthesizes the regional perspectives and results of the thematic discussions among the participants and contributors to the informal consultation.

Key general recommendations common to all the breakout groups:
Participants welcomed the Initiative. It was noted that this Initiative was going to be an important addition to the global, regional and country-led efforts to strengthen tracking of progress towards achieving the SDGs and transforming food systems (such as the UN Food Systems follow- up. i.e. the UNFSS Coordination Hub, the UNFSS Coalitions, the national teams working with the national convenors on the implementation of national pathways and the work of the Issue-based Coalition on Sustainable Food Systems).

It was noted that tracking food systems require a clear, rigorous and comprehensive set of metrics and indicators, to guide decision-makers and to hold them accountable. Having high-quality, timely, accessible, comparable and reliable data, which monitor and evaluate the whole agrifood value chain, will help improve the statistical significance and reliability of relationships between policies, investment, and outcomes.

Suggested indicators were generally found useful and high quality, but some region-specific measures were suggested for each thematic areas.

The debate highlighted the need to solve the issue of the lack of data through a global commitment to raise the bar on the international political agenda. At the EU level, there are many frameworks and approaches that could guide the work of FSCI.

The discussion provided an opportunity to explore demand-driven indicators that respond to specific challenges in countries and regions. It was also recommended to use a food systems approach, not only focus on the primary production phase (due to the lack of data available) as the majority of indicators included so far.

Importance of political support to the initiative was highlighted and financing the data collection. Advocacy for domestication and country-led implementation of the indicators was raised. Disaggregation of data based on geography, gender, youth and age and consideration of cross-sectorial indicators were also suggested.

There was a general concert to translate the indicators to Russian and other working languages in the region, where possible, for ease of adoption of indicators by countries.

<u>1. Diets and nutrition</u>
<u>Summary</u>

The impact of socio-economic and health-related shocks on food security and nutrition is a growing concern for the region, especially in import-dependent countries. In order to monitor these impacts and support evidence-based decision-making, it was suggested to collect more data related to vulnerable populations.

The prevalence of different forms of malnutrition and diet-related NCDs were also highlighted as prominent food system related issues in the ECA region. The monitoring and improvement of the food environment was recognized as an effective solution for these problems.

Finally, it was agreed that the availability of data should be improved through more strategic and predictable data collection. More advocacy is needed to help countries recognizing the value of data in supporting decision-making processes.

Do you consider the proposed indicators relevant, high quality, interpretable and useful?

The proposed indicators were considered necessary and useful to reach national and regional targets for all food system actors such as governments, civil society organizations, UN agencies and experts.

Specific recommendations:

Indicators measuring *processed foods*, foods high in sugar, salt etc. are useful when considering their contributions to *non-communicable diseases (*NCDs).
In order to complement the indicator of the coverage of iodized salt (% of households), an indicator to measure the *prevalence of iodine deficiency* among the population should also be considered.
Ensuring food safety is crucial for food security, and it is an integral aspect of sustainability of food systems. More *food safety indicators* in the framework.
Food borne diseases and residues of pesticides and veterinary drugs were highlighted as potential food safety risks in the region. Monitoring these issues would help to understand their health impacts.
Indicators mainly cover 6-23 months of age and adults. *Indicators for adolescents* should be also considered, for example the prevalence of anemia and other micronutrient deficiencies among adolescent girls could be a useful information for the ECA region.
In order to monitor the impacts of socio-economic, health-related and humanitarian crises, it was suggested to collect more data related to *vulnerable populations (e.g. refugees, internally displaced or lower income groups*). The changes in dietary patterns of vulnerable populations during crises could be interesting indicator for food security/resilience.
As food security and resilience becomes increasingly important in the region, it is recommended that indicators related to *self-sufficiency, strategic food stocks and food independence* could be also measured.

What are the data gaps that you can identify? Are these gaps structural? If not, are there some data or indicators that you know are available in your region and can be used for the purpose of this monitoring and assessment system?

It was agreed that data gaps exist, and the availability of data should be improved through more strategic and predictable data collection. In the ECA region, sometimes the national policies and strategic plans do not ensure effective and frequent data collection. One of the main reasons is the need for financial resources.

It was also noted that in many cases, the data is collected by external partners, such as academia and independent organizations. In this regard, mapping data availability would be very important.

In addition, more advocacy is needed to help countries to recognize the value of data in decision-making, as well as encouraging data sharing, to ensure that all available data is used by policy makers. Transparency should be also improved since in some countries data is considered a sensitive area, with a reluctance to share it.

What are the regional aspects we need to take into consideration at the moment of selecting a set of indicators to monitor the state of food systems and its evolution?

The prevalence of different forms of malnutrition and diet-related NCDs were highlighted as prominent food system related issues in the region. The prevalence of overweight and obesity causes concerns for both children and adults. NCDs represent a significant burden and contribute to a high percentage of the mortality in the region, therefore they should be addressed.

Improving the food environment is key in this process. Even when policies affecting food environments such as marketing of breast-milk substitutes, or marketing junk foods to children do exist, they are not

well implemented. The implementation of large-scale nutrition related programmes targeting both children and adults are also necessary.

The impact of socio-economic and health-related shocks and the ongoing war in Ukraine on food security and nutrition is a growing concern for the region. In order to monitor these impacts and support evidence-based decision-making, it was suggested to collect more data related to vulnerable populations. For example, in Turkey, there are particular issues to be addressed among refugees' communities, including stunting, wasting and malnutrition under 5 years old, especially as the number of refugees are rising quickly.

It may be also needed to streamline the cultural aspect of diets in different countries, and analyze how food systems and technical groups can help countries and policies to adjust to this issue. There are certain countries in the region, where the traditional diet contains high proportion of animal-based products, while alternative protein sources, such as pulses are not commonly consumed. Raising consumer awareness on nutrition and sustainability are crucial in this context.

Disparity in terms of consumption patterns is significant in the region and it sometimes occurs within a country or within different population groups. Therefore, the indicators related to dietary patterns are particularly important for the region. However, the relevancy of the Indicator on "zero fruit and vegetable consumption in adults" was questioned.

2. Environment and climate domain
Summary

There was a shared, renewed understanding that food systems have an outstanding role regarding their impacts on nature and climate change.
Also, the ECA region was particularly affected by the negative impacts of COVID-19 and the ongoing war in Ukraine, resulting in rapidly rising food and energy prices.

In the region, a more conscious consumer behavior could have an impact on the climate-food nexus and the planetary boundaries. On one side, there is an increasing demand for animal food products, but, on the other side, consumers are becoming more aware of the challenges facing the food system, resulting in a growing number of them that are paying attention to how food is produced, processed, distributed and, finally, consumed and, sometimes, wasted, particularly in relation to the major challenges the ECA region is now facing.

There were additional indicators suggested particularly related to water scarcity and its sustainable management; soil degradation and land management; climate change adaptation and mitigation; pollution (air, water, land) and GHG emissions; food loss and food waste; energy efficiency at all stages of the food supply chain, and, finally, antimicrobial resistance (AMR).

Regarding the data sources and availability in the region, there were several suggestions at the international, regional, sub-regional and national level, with a common recommendations of all data sets to be gathered, collected and analysed in line with the EU environmental and climate framework of reference.

Do you consider the proposed indicators relevant, high quality, interpretable and useful?

A few participants pointed out that the *indicators are very much dedicated to the production*, whereas the other stages of the food supply chain are not well represented (processing, transformation, distribution, consumption and waste).

Water availability issues and food loss and waste were the topics that came out frequently in the discussion. Water systems decline due to improper construction of water reservoir, thus water vulnerability needs to be assessed in the region (Armenia). Drought was also highlighted as an increasing challenge, particularly in the Caucasus and some Central Asian countries that are downstream from the source of important natural water supplies, leading to significant decline in water systems.

Land and soil pollution, either by excessive input use and/or solid waste disposal:
land degradation and land management are very relevant to be considered at the regional level.
Discussion on the possibility to include indicators to monitor and measure crop rotations, not only to restore soil but also valuable for reversing biodiversity loss (i.e. the case of Albania)
The large extent of ploughed land and its growing trend was also considered a concern (with the consequent decrease in productivity and the increase in input use).

Reflection was made on the possibility to include within the *GHG domain, the reference to energy efficiency*, with particular attention to the renewable energy.

GHG domain:
Carbon capture is a relevant indicator, but there is a common understanding that it needs to be evaluated *throughout the whole food value chain* (raised by a participant from Ukraine)
There is a suggestion to incorporate evidence from the food loss and waste also at the GHG emissions level. There was also a specific suggestion that an indicator on food waste reduction could be included, and it was noted that countries have begun collecting this data in many instances (raised by a participant from Bosnia and Herzegovina).
Measurement, reporting and verification of greenhouse gas emissions and mitigation in agriculture are not common in the region and national emissions trade schemes (if exist) are not always in line with the European Union Emissions Trading Scheme - EU ETS.

Debate on the degree of localness of food value chains and GHG:
Suggestions were made to include an indicator of the "localness of food systems", although it would be difficult to define.
However, concerns were raised to avoid demonization of the food trade sector. While it might make sense intuitively, eating locally would only have a significant impact on reducing GHG if transport was responsible for a large share of food's final carbon footprint. For most foods, this is not the case, see the supporting paper (raised by UNECE).

Consumption patterns, particularly the change of diet and nutrition habits among the ECA population, and its positive environmental implications could represent a valuable evidence to connect the climate-related indicators. An example is the link between consumption of foods of animal origin, and the amount of land required for animal feed for livestock.

Linked to an earlier comment, there is a need to consider the different stages of the food supply chain and how they contribute to GHG emissions and environmental impacts. It was acknowledged that there is progress in the application of innovative solutions to reduce the environmental impacts of food processing through bioeconomy approaches. It was suggested that this is an area where progress will be made in the region in the next few years, and it can be a potential indicator for the future analysis.

Specific Recommendations:

- *Applying a food systems approach* to the selection of indicator, and not focusing only on the production stage of the food supply chain. There was a shared perception that the indicators suggested were only related to the primary production phase.[8]
- Use of *renewable energy* in the food system suggested as an indicator.
- The *percentage of countries with operational drought monitoring systems in place* is suggested as an additional indicator for the Land Use and Water use domains (this indicator could also be considered for the Resilience domain) (WMO); Drought and Flood Forecasting and Warning Systems and Services suggested as indicator (WMO).
- *Food loss and waste reduction* targets are suggested as additional indicators, from economic and environmental perspective at different levels, to identify potential causes and potential interventions. The indicator on food loss and waste is not only related to the environmental *Pollution domain* and also strictly related to the *GHG emissions* and natural inputs efficient management (water use)(raised by a participant from Ukraine). This requires assessing waste at the different stages of the food value chain, including prevention of food waste at the consumer stage.
- To expand indicators of Land Use domain and include *crops typology and crop rotation*.
- *Reduced use of pesticides and other relevant chemical inputs* in agrifood production can also be an indicator.
- *Air pollution and solid waste* should be addressed with specific indicators.
- *Meteorological data on climate change impact on different economic sectors* should be included
- The use of antibiotics in food – also associate with the One Health approach - is a missing aspect in the indicators and reveals much about the health of food system (in line with the EU Green Deal and in line with the national policies in the most countries in the region).
- *What are the data gaps that you can identify? Are these gaps structural? If not, are there some data or indicators that you know are available in your region and can be used for the purpose of this monitoring and assessment system?*

The European Green Deal, Farm to Fork Strategy, Biodiversity Strategy, Repower Strategy, EU Zero Pollution plan represent a good framework of reference for supporting the countries in the region to become more sustainable by turning climate and environmental challenges into opportunities, in line with international standards: UNFCCC; UN Convention on Biodiversity, United Nations Convention to Combat Desertification and SDGs.

Some data gaps exist in agricultural water withdrawal. The assessment becomes very difficult, when the irrigation largely relies on individual wells, for example in the case of Albania. Local estimation is possible, but it is dependent on local governance. It was advised to consider assessing underground aquifers with remote sensing. The majority of data indicators could be captured with remote sensing, including also burning residues.

Food loss and waste data are good quality and available in the region, and data collection is being invested in. FAO and UNEP are regularly publishing data on the food loss and food waste Indices.

On land use, statistics in the region are often available and reliable.

Bosnia and Herzegovina Council of Ministries recently approved a set of environmental indicators which might be useful for the initiative.

---

[8] According to latest data, the vast majority of the environmental footprint of food products (ca. 80%) is generated during the production stage (land use and farm-stage).
https://ourworldindata.org/food-choice-vs-eating-local

Serbia, Environmental Protection Agency, can provide data on GHG emissions, and on land use and land use changes. Whereas, common to all Western Balkan countries, it is more difficult to provide data on pollution indicators, there is the need to develop monitoring and data analysis systems on environmental pollution.

What are the regional aspects we need to take into consideration at the moment of selecting a set of indicators to monitor the state of food systems and its evolution?

The highly diverse region of Europe and Central Asia faces the same range of challenges due to the increasing vulnerability to climate change impacts, and environmental pollution.

One of the top policy priorities among the countries in the region is more rationale, sustainable and coordinated use of natural resources, namely water and land.
In addition to the threat posed by climate change to water availability in the region, wasteful and inefficient water use emerges as a common problem across the region and as one of the primary area of policy action, particularly for the Central Asia and Southern European countries, as well as for some countries in the North. But, cutting across discussions of the need for improved water resource management, there is an equally strong emphasis on the importance of strong water governance and cooperation (e.g. Azerbaijan and Turkey cooperation).
Land degradation, particularly the high-level mechanical degradation, and soil erosion are also common challenges across the region. Land use, and conservation approaches should be applied in a way that benefits both productivity and nature. Reference to heavy contamination of the soil from previous decades was made. Alongside the land management challenges, aspects of preventing environmental pollution through the responsible use of chemical inputs in food production, and importance to avoid the mismanagement of pesticides and fertilizers, in the region still remains a crucial issue, since these items generate soil and water pollution, contribute to the loss of biodiversity and diverse ecosystems in the region. The situation is further exacerbated in some countries due to the proliferation of obsolete pesticide stocks.

It was noted, that these actions are also important for human health. Furthermore, effective waste disposal, and biosecurity measures are also important to prevent environmental pollution, for example adequate waste disposal from *slaughterhouses and other livestock activities.* Also affected are the quality and cost of food, with impacts to the whole food system, food security, and human health. All of the components of food systems (production, processing, distribution and consumption) are generating huge amounts of externalities. These are primarily linked to damages to the environment throughout the whole food value chain and the costs of human health through consuming unhealthy food.

Plastic waste was also another major theme of concern in the region.

Antimicrobial resistance poses a major threat in the region and preventing the threat of AMR is an important priority. FAO is currently supporting data collection through surveys on the use of antimicrobials in the livestock sector in 14 programme countries.
In addition, relevant efforts are recorded in the EU countries to prevent food losses and food waste, and the topic is receiving increased attention in the Caucasus and Central Asian context.

3. Livelihoods, poverty and equity Domain

Summary

The importance of providing clear definitions for the indicators was noted, and they are suggested to be strengthened in some cases.
Concerns on the availability of data on agriculture and livelihoods at national level due to significant informal and unregistered information were raised several times during the discussion. Participants

further stressed the need for sex-disaggregation of data and per urban and rural areas where possible as some of the issues could be very context specific.
The speaker explained the challenge on how to identify populations involved in the food systems, as some countries are collecting disaggregated data, while others do not have even the overall data. He referred to the background paper where ILO data which includes primary employment complimented with EUROSTAT. However, there are some countries that are excluded from ILO data, and informal, self-employed, migrant data is just estimated.
Participants suggested that indicators on the impact of forced or voluntary migration, remittances, EU indicators, and seasonal work could be also considered.
Do you consider the proposed indicators relevant, high quality, interpretable and useful?

Specific recommendations:

Countries (Turkey, Albania, Georgia) commented that the *agricultural income* is one of the key indicators, but the definition needs to be clarified by considering factors such as rural-urban households, percentage of the income, sources that are considered as agricultural income, and the part of the system this indicator considers (full food chain or only farm level). Additionally, the indicator "percentage of the population earning low pay" is important, since it can relate to the migration from rural areas due to low income. But the definition includes hourly earnings for agriculture; which is very difficult to obtain in many countries.

On the *employment indicators*, the group noted the importance of including informal employment as it is very common in the agricultural sector but data is difficult to obtain. While the number of smallholders and their contribution to the agricultural production is very significant in the region, they often remain in informal employment as not all are registered. The land holdings indicator is relevant, but measuring access to land is problematic due to the lack of registration.

"Households with significant income from agriculture" was highlighted as key indicator, but the definition needs to be clear on the target population whether they include full food chain or only agriculture. The indicators under employment (earnings/wages) are also noted as highly relevant as people are leaving rural areas because of low compensation and non-farming and shifting/seasonal sources; but data on hourly earnings is very limited or non-existent in the region. The indicators on human rights were found very relevant, with a note on access to land as well as resources.

Access to resources was also suggested as a potential indicator. The FSCI Expert noted that the rights and access to inputs and resources should be cross-examined closer because of their implications on the livelihoods.

What are the data gaps that you can identify? Are these gaps structural? If not, are there some data or indicators that you know are available in your region and can be used for the purpose of this monitoring and assessment system?
Participants noted the crucial need for accountable data sources. International organizations, such as World Bank, are reliable and accurate, but challenges exist at the national level, particularly for farm-relevant data. In certain countries, data on small- and medium scale food enterprises is also absent. At country level, informal data sources might provide the information that are not available in the official sources, or complement it. These could be considered, depending on their reliability.
Disaggregation of data (rural/urban, gender, age) wherever possible is strongly advised by the participants and their quality should be improved. Concerns were raised on the limited availability, informality, non-informality and access to data.

Bosnia and Herzegovina informed on their Ministry's available data source, which should be considered amongst the resources, as they had a recent survey on agriculture and rural development.

What are the regional aspects we need to take into consideration at the moment of selecting a set of indicators to monitor the state of food systems and its evolution?

For the EU candidate countries in the region, it is important that indicators are harmonized or taken into account with those also set by the EU, when agreeing on the Food Systems Countdown indicators.

Suggestion was made to include also an indicator on migration's impact on the food systems.
In addition, seasonal work is a significant factor in the region particularly for the agriculture sector and suggested to be considered.

4. Governance Domain
Summary

It was noted that in the Europe and Central Asia region, the governments have well-established systems of governance in place, which provide a good foundation and experience on agriculture, education, health, nutrition and trade. But it is important to take a critical view and to ensure that weaknesses in governance are addressed and to consider as part of the continuing evolution specific aspects to be improved as part of transforming agrifood systems. It was acknowledged that it is important to encourage monitoring, evaluation and continuous development. Selecting and tracking the right set of indicators will help countries to ensure that the transformation of food systems proceeds in the agreed direction, and the holistic food systems approach evolves in a continual momentum.

It was highlighted that UNFSS national pathways have been important to formulate policy documents and commitments related to sustainable food systems. This process of policy integration and committing the actions in the national pathways to strategic plans is on-going in many countries of the region.

Do you consider the proposed indicators relevant, high quality, interpretable and useful?

Discussing the indicators related to the governance thematic was interesting, but also a relatively new discussion for the audience.
It was challenging to identify specific, quantifiable indicators.

Specific recommendations:

In case of *food systems governance, multisectoral coordination* was highlighted as a key area for improvement, and a number of examples were given to demonstrate efforts both at national, and at local levels.

Under *coordination*, it was mentioned that it is important to look at the trade-offs between different policy goals and ensure they are balanced. The indicators would need to allow to look not only horizontal but also vertical levels of coordination. In addition, it would be important to capture the trends both on the national level and subnational level, including recognizing that is there are moves towards different government sectors working together, e.g. nutrition and agriculture, agriculture and environment – this is an essential prior step to full policy coherence (end objective). This information would help to identify specific interventions on the sub-national level and support the more efficient use of resources in food systems transformation. Responsible investment in food systems might also be an indicator.

There are several examples, where the formulation and implementation of national food system pathways are supported by national coordination platforms. However, the need for capturing nuances while looking at policy coordination and coherence was highlighted. Different countries may have different policy instruments or tools in place that can lead to the same transformation. Governance also needs to take

place at many different levels and across many different aspects of the food system (value chain development, sustainable tourism, environmental protection, food safety, animal health, etc.) and the indicators need to ensure these are adequately measured. Or at least it would be helpful to understand at what level and how effective governance will be measured.

On *shared vision indicators*, it was suggested that it is important to look beyond what is reflected in the national food system transformation pathways. The candidate indicators could be expanded to capture the *cooperation and coordination level between the public and private sector; not only CSO level*.
For example, inter-branch organizations and similar public-private platforms contribute to the transformation process in terms of defining a national vision for specific sectors in Albania (fresh produce, dairy, meat etc.), similar to the EU model.

Under *implementation*, additional to the "marketing of breast-milk substitutes restrictions" indicator, it is suggested to look at breastfeeding in a more holistic manner and measure public health indicators, such as the *rate of exclusive breastfeeding*, as well as *the level of education provided to mothers related to breastfeeding*.

What are the data gaps that you can identify? Are these gaps structural? If not, are there some data or indicators that you know are available in your region and can be used for the purpose of this monitoring and assessment system?

The FSCI Expert explained that the expert team at the Initiative tried to identify the most relevant indicators and agreed that at least 70% of countries should have data on the selected indicator. However, in many cases, this may not be realistic. Due to this limitation, one recommendation is to choose potential indicators, even if data is not currently available and advocate for more data collection in the future.

Regarding data sources in the region, a recommendation is to make use of the regional platforms that are involved in food system analysis (e.g. Economic Coordination Organization - Regional Coordination Centre for Food Security (ECO-RCCFS), Black Sea Economic Cooperation - Regional Cooperation Centre for Sustainable Food Systems (BSEC-RCCSFS), etc.).

What are the regional aspects we need to take into consideration at the moment of selecting a set of indicators to monitor the state of food systems and its evolution?

As mentioned above, UNFSS national pathways are important tools to formulate holistic policy documents and commitments related to sustainable food systems in the region.

Some specific examples from Albania were shared that might be relevant for the whole region. These are the formulation and implementation of policies to promote healthy diets (school meals, nutrition education, marketing of food products, regulation of breastmilk substitutes) and food safety (e.g. on foodborne diseases and pesticide/veterinary residues). The One Health approach was also highlighted as an important example on policy coherence.

5. Resilience and sustainability Domain
Summary

- Overall, the participants found the candidate indicators important and relevant, although 26 indicators are quite a long list to be considered and evaluated.
- The importance of providing clear definitions for resilience considering the regional nuances for the indicators was noted.

- Resilience and sustainability indicators, as complex, cross-cutting themes, have to be identified avoiding overlaps with other indicators under the five domains.
- Resilience and sustainability are considered at all levels as a pre-condition for sustainability and its monitoring is necessary to capture food systems' holistic nature. Resilience is somehow a trade of short-term efficiency and longer-term survival.
- Identifying indicators in this area was very challenging, as it is very hard to find reliable, quantifiable indicators.
- It would be important to capture diversification as one of the potential paths to follow to transform food systems. If a country has diversified trade partners, food production, import and export, it will become more flexible to absorb shocks in the future.

Do you consider the proposed indicators relevant, high quality, interpretable and useful?
<u>Specific recommendations:</u>

- Indicators must capture *regional specificities*: sub-national and disaggregated.
- *Proposal of including renewable energy indicator*: already included into the following indicators: Mobile cellular subscriptions (per 100 people); Access to electricity (% of population); Renewable electricity output (% of total electricity output) (Ukraine).
- Suggestion to include *social protection indicators*: adequacy coverage, school feeding programs (also mentioned in the Livelihoods domain).
- Proposal of indicator to monitor and track the *exposure the dependency on international markets and trade, import dependency ratio* should be an aspect of resilience.
- *Proposal of inclusion of an indicator on the number of operational drought and early warming systems*.
- Indicators related to *higher levels of education (vocational education, and above)* would be interesting and should be included, too.
- *Corporate non-financial reporting – ESG & Corporate Social Responsibility* – are suggested as indicators.

What are the data gaps that you can identify? Are these gaps structural? If not, are there some data or indicators that you know are available in your region and can be used for the purpose of this monitoring and assessment system?

Gaps:
Environmental-related indicators: incidents related to adverse events – number of deaths (attributed only to agriculture) associated to the social impact of those events, considering increasing droughts.

Suggestions:
Global Innovation Index by WIPO and consortium of partners (also including private sector)
EM-DAT human impact
Combining also data from databases on Data conflict, armed conflict and IDPs.
Non financial reporting – CSR & ESG, in corporate reporting is increasingly gaining attention within EU countries and might represent a framework of reference for other countries in our region.

What are the regional aspects we need to take into consideration at the moment of selecting a set of indicators to monitor the state of food systems and its evolution?

Food systems resilience is critical to food security and nutrition (e.g., fragile states = food insecurity, COVID-19).
The theme is also critical for other functions (e.g., livelihoods, inclusion).

Within EU countries, we are witnessing an ever-lasting debate within the 2 different views on CAP: traditional family farming and market-oriented industrial agriculture efficiency view points, with key components including resilience.
Growing consensus on the diversification as one of the potential paths to follow to transform food systems. If a country has diversified trade partners, food production, import and export, it will become more flexible to absorb shocks in the future.
Diversification – to capture the social, economic, environmental dimensions and make food systems intelligible.
A relevant policy priority area for the region refers to migration, both forced and voluntary, (how migration reveals some aspects of the resilience ability of the food system of the region).
Green finance also is an indicator we may want to take into consideration and show the interest of governments in the region for investing responsibly in agrifood systems.

Conclusions
"The need for food system transformation is undeniable. We need to transform the way that we produce, we need to do something about the way that we consume, we have to try to transform the way that we conceive food, if we want to be able to offer diets that are healthy but at the same time produced in a sustainable, resilient and equitable way" said **José Rosero Moncayo,** Director of the Statistics Division, FAO, Co-Chair of the Initiative.

Food system transformation is urgent, requiring rigorous, science-based monitoring to guide public and private decisions and support those who hold decision-makers to account.

National and global governments are struggling to govern the increasingly complex crises and powerful forces in food systems, especially as the COVID-19 pandemic, climate change and the recent conflict in Ukraine have shown the fragility of food systems and the lack of sustainability to continue along the present course. These crises and challenges underscore the urgency to change the trajectory for food systems but also offer great potential to do so.

Monitoring food system transformation in this manner can aid governments in setting priorities and establishing incentives and regulations to align food systems in a transformative direction. High-quality evidence allows food system actors to undertake "course corrections" and make necessary changes.

This transformation is achievable. Rigorous monitoring is necessary to keep progress on track. It requires validation and to put indicators into reality of people that use this type of data to guide for the right policy.

Concerns about data availability:

- The initiative is aiming at solving a remarkable challenge: Using indicators based on the data available now.
- Lack of data could be solved through a global commitment to raise the bar on the international political agenda.
- Need to keep developing methods to compile indicators that are relevant to food systems and expand the list of indicators that we have now.
- The current set of proposed indicators – more bias towards production and agriculture in contrast to other aspects of the food system. The reason is the lack of data and methods.

Jessica Fanzo, PhD, Bloomberg Distinguished Professor of Global Food & Agricultural Policy and Ethics, Co-Chair of the Initiative highlighted the importance of the convening power of FAO: around 500 people joined to the discussions from all regions to provide their feedback.

Raimund Jehle, Regional Programme Leader, Deputy Regional Representative, FAO Regional Office for Europe and Central Asia confirmed that the there is an interest in measuring the progress to demonstrate the importance of the food system contributing to achieve the SDGs.

"National ownership of sustainable food systems concept is the key to be successful.
The challenge now is how to get these actions at country level and harmonize these indicators with different national sectorial strategies as in many countries a food systems strategy is not yet available. We need countries' input and support in order to work more coherently and to integrate into the national pathways developed as part of the UN Food Systems Summit", he added.

Agenda of meeting
AGENDA

08:30 -08:50 hours		*Welcoming remarks*
		**Raimund Jehle**<br>Regional Programme Leader, FAO Regional Office for Europe and Central Asia<br>**Hernán Daniel Muñoz**<br>Statistician, Statistics Division, FAO
8:50 -09:15 hours		*Introduction to the initiative*
		**Lawrence Haddad**<br>Executive Director, GAIN
09:15 -10.30 hours		*Breakout sessions, block 1:*<br>*• Diet, nutrition and health Domain;*<br>*• Environment and climate Domain;*<br>*• Livelihoods, poverty and equity Domain.*
		Moderators:<br>**Musonda Mofu**<br>Deputy Executive Director, National Food and Nutrition Commission, Zambia<br>**Fabrice DeClerck**<br>Science Director, EAT<br>Senior Scientist, One CGIAR<br>**Alejandro Guarin**<br>Researcher, International Institute for Environment and Development (IIED)
10:30 -10.45 hours		*Summary of the breakout sessions*
10:45 -12.00 hours		*Breakout sessions, block 2:*<br>*• Governance Domain;*<br>*• Resilience and sustainability Domain.*
		Moderators:<br>**Namukolo Covic**<br>Registered Nutritionist, Health Professions Council of South Africa<br>Senior Research Coordinator at the International Food Policy Research Institute<br>**Dr. Preet Lidder**<br>Technical Adviser to the Chief Scientist, FAO
12:00 -12.15 hours		*Summary of the breakout sessions*

12:15 -13.00 hours		*Wrap up and conclusions*

Participants List

**Country/ Organization**	**Name**	**Title**
FAO - REU - RI1	Ms Bianka Laskovics	Regional Initiative Coordination and Rural Development Associate
FAO - REU	Ms Valeria Rocca	Regional SDG Advisor
FAO - REU	Mr Giorgi Kvinikadze	Statisttician
FAO - REU	Anna Jenderedjian	Gender Mainstreaming and Social Protection Specialist
FAOTR	Ozcan Turkoglu	Senior Programme and Pipeline Coordinator
FAOTR	Yasemen Asli Karatas	Programme Associate
WMO	Natalia Berghi	UN Programme Officer
Regional DCO in Istanbul	Alper Almaz	
North Macedonia	Mr Aleksandar Musalevski	Head of sector for coordination of Minister’s Cabinet, Ministry of Agriculture, Forestry and Water Economy (also involved in Agriculture Policy Analysis Department)
North Macedonia	Ms Neda Gruevska	Rural Development Department, Ministry of Agriculture, Forestry and Water Economy
North Macedonia	Mr Jordan Chukaliev	Professor, University Ss. Cyril and Methodius, Faculty of Agricultural Sciences and Food – Skopje, Institute of natural resources, management and environment protection in agriculture
North Macedonia	Ms Aleksandra Martinovska Stojcheska	Professor, University Ss. Cyril and Methodius, Faculty of Agricultural Sciences and Food – Skopje, Institute of Agricultural Economics
North Macedonia	Mr Vladimir Petkovski	Institute for Economics
North Macedonia	Mr Igor Spirovski	Head of Department of Physiology and Monitoring of Nutrition, Public Health Institute
Finland National Convenor	Mr Jyri Ollila	Counsellor; World Food Systems Summit Ministry of Agriculture and Forestry, Finland

Albania	Ms Valbona Paluka	Advisor to minister of agriculture on food safety/security and Curator of National Food System Dialogues
Albania	Ms Renata Kongoli	Professor at Agriculture University of Tirana
Albania	Mr Drini Imami	Professor at Agriculture University of Tirana
Albania	Mr Engjell Skreli	Professor at Agriculture University of Tirana
Spain	Ms Patricia Villoch Carrión	Ministry of Agriculture, Fisheries and Food and the Spanish Representation to FAO
Republic of Moldova	Ms Maria Nagornii	Ministry of Environment, Head of the Division for Policies on Pollution Prevention
Republic of Moldova	Ms Galina Petrachi	Ministry of Agriculture and Food Industry, Department for Rural Development Policy and Programs
Republic of Moldova	Ms Marina Dintiu	National Food Safety Agency, Head of Department of Quality and Safety of food products of non-animal origin
Republic of Moldova	Mr Viaceslav Grigorita	Ministry of Agriculture and Food Industry, Head of Department Policies in Plant production and processing sector
Republic of Moldova (additional person)	Ms Liliana Martin	Ministry of Agriculture and Food Industry, Policy Analysis, Monitoring and Evaluation Department, Acting Head of Department
Republic of Moldova (additional person)	Ms Nicoleta Golovaci	Ministry of Agriculture and Food Industry, Policy Analysis, Monitoring and Evaluation Department, Senior Consultant
Kyrgyz Republic	Ms Meerim Esenkulova	Specialist, Food security Department Ministry of Agriculture
Kyrgyz Republic	Mr Abdymital Chyngojoev	Expert
Kyrgyz Republic	Mr Kanat Tilekeev	Research Fellow, UCA University
Kyrgyz Republic	Ms Miraida Naktaeva	Head of welfare department, Ministry of Labour, Social Protection and Migration
Georgia (Food System Summit Convenor)	Ms Ekaterine Zviadadze	Head of Primary Structural Unit of the Policy Coordination and Analysis Department at the Ministry of Environmental Protection and Agriculture of Georgia

Georgia (Thematic Expert)	Ms Maia Tskhvaradze	Head of the Climate Change Division at the Ministry of Environmental Protection and Agriculture of Georgia
Georgia	Mr Giorgi Machavariani (accompanies Ms Tskhvaradze)	Senior Specialist at the Climate Change Division at the Ministry of Environmental Protection and Agriculture of Georgia
Georgia (Data Expert)	Mr Giorgi Sanadze	Head of the Agricultural and Environment Statistics Department at Georgian National Statistics Office (GeoStat)
Georgia	Mr Irakli Tsikhelashvili (accomapies Mr Sanadze)	Head of Environment Statistics Division
Georgia	Mr Goga Talakhadze (accomapies Mr Sanadze)	Head of Agricultural Statistics Division
Hungary (Observer)	Ms Liliána Kaszás	Hungarian Ministry of Agriculture, FAO coordinator
Hungary (Observer)	Ms Dorka Mudrity	Hungarian Ministry of Agriculture, Project referee
Turkey	Pelin POLAT ÇAVUŞOĞLU	Engineer, General Directorate of Agricultural Reform (Ministry of Agriculture and Forestry)
Turkey	Dr. Aygül ÇAĞDAŞ	Engineer, Strategy Development Department (Ministry of Agriculture and Forestry)
Turkey	Arap Diri	Engineer, Turkish Statistical Institute
Turkey UNRC	Bulent Acikgoz	UNRC Office
Turkey	Mehmet Emre Uğur	Expert, Turkish Statistical Institute
Turkey UNRC	Bulent Acikgoz	Partnerships and Development Finance Officer, UNRC
Turkey FAO	Ozcan Turkoglu	FAO
Turkey UNICEF	Mehmet Ali Torunoglu	UNICEF
Serbia	Milos Stojanovic	FSS Curator, Ministry of Agriculture, Forestry and Water Management
Serbia	Dragana Vidojevic	Head of Section for Indicators and Reporting, Serbian Environmental Protection Agency, Ministry of Environmental Protection
Serbia	Milica Jevtic	Head of Unit for Analytics and Statistics, Ministry of Agriculture, Forestry and Water Management

Serbia	Jelena Perać	Group for agricultural statistics, Statistical Office of the Republic of Serbia
Tajikistan	Mr Sherali Rahmatulloev	Chief specialist of the Department of Maternal and Child Health and Family Planning of the Ministry of Health and Social Protection
Tajikistan	Mr. Nazarov Bahodur	Head of department on agrarian policy and food security monitoring of the Ministry of Agriculture of the Republic of Tajikistan
Tajikistan	Mr. Andamov Ismoil	Head of the Department on Veterinary and Breeding Supervision of the Committee for Food Security under the Government of the Republic of Tajikistan
Tajikistan	Mr. Murodjon Ergashev	Expert on Environment
Tajikistan	Mr. Firuz Saidov	National Consultant on Climate Change in Project Management Unit under Committee for Environmental Protection under the Government of the Republic of Tajikistan
Tajikistan	Mr. Jamshed Kamolov	Head of Main department on Population and Territory Protection of the Committee on Emergency Situations and Civil Defense under the Government of the Republic of Tajikistan
Tajikistan	Mr. Rustam Babadjanov	Professor, Institute of Economy and Demography of the Academy of Science of Tajikistan
Armenia World Bank	Artemis Ter Sargsyan	National Consultant to WB
Armenia World Bank	Irina Ghaplanyan	
Armenia World Bank	Arusyak Alaverdyan	
Armenia World Bank	Vahan Danielyan	
Armenia (recommendation from WFP)	Vardan Urutyan	Rector of Agrarian University
Armenia (recommendation from WFP)	Mr Gevorg V. Ghazaryan	
Armenia (recommendation from WFP)	Magda N. Hovhannesyan	
Ukraine	Olena Kovalova	Senior expert, IPRSA, Dr.Sc Econ., former DM of MAPF
Ukraine	Larysa Petriv	Head of Unit, National Scientific Center for the Climate-smart Agriculture, National Academy of Agrarian Science
Ukraine	Mykhailo Malkov	Environment Advisor, Office of the UN Resident Coordinator to Ukraine

Ukraine	Olena Safarova	Area Coordinator, Emergency Programme in Ukraine
Expert	Elisabeth Duban	
Expert	Valentina Bodrug-Lungu	
Expert	Eugenia Ganea	
WMO	Dr Elena Mateescu	
BiH	Mr Dalibor Vidacak	Expert Adviser, Food Safety Agency of BiH
BiH	Ms Vanda Medic	Senior Adviser, Ministry of Foreign Trade and Economic Relations BiH
BiH	Ms Sanida Sarajlić	Head of Department for Rural Development Policy Coordination, Ministry of Foreign trade and Economic relations of BiH
UNECE	Marit Nilses	
WMO	Milan Dacic	
WFP Cairo Regional Office	Khalid al-Qudsi	
WMO	Bob Stefanski	Chief, WMO Applied Climate Services Division
Georgia	Zaza Chelidze	Statistical Expert
Belgium	Louis Bout	Cabinet Member, Cabinet Hilde Crevits, Vice-Minister-President of the Flemish Government and Flemish Minister for Welfare, Public health and Family
Montenegro	Ms. Zorka Prljević	Deputy Director, Agency for Food Safety, Veterinary and Phitosanitary Affairs
Montenegro	Ms. Jelena Vračar Filipović	Advisor, Agency for Food Safety, Veterinary and Phitosanitary Affairs
Montenegro	Ms. Dubravka Radulović	Advisor in Directorate for Agriculture, MAFWM
Montenegro	Mr. Mirsad Spahić	Head of Department for Economy Analysis and Market, MAFWM
Montenegro	Ms. Kristina Radević	Advisor in Directorate for Rural Development, MAFWM
Belarus	Olga Lobanova	Consultant of the Agriculture and Environment Statistics Department, the National Statistical Committee of the Republic of Belarus
Belarus	Iryna Husakova	PhD, Head of the Food Market Sector, The Institute of System Research in Agro-Industrial Complex of National Academy of Sciences of Belarus

Belarus	Natallia Kireenko	DSc, Professor, Head of the Chair of Innovative Development in Agro-Industrial Complex, The Belarusian State Agrarian Technical University
Belarus	Irina Voitko	PhD, Associate professor of the Chair of Innovative Development in Agro-Industrial Complex, The Belarusian State Agrarian Technical University
Belarus FAO	Alesia Launikevich	FAO
JHU	Kate Schneider	JHU
Iceland	Dr. Matthías G. Pálsson	Permanent Representative of Iceland to FAO, WFP and IFAD

FAO RLC Regional Expert Consultation on the Food System Countdown Initiative's Indicator Framework
Virtual, May 17, 2022

This report was produced by RLC to summarize the results of the FAO RLC regional expert consultation held on May 17, 2022.

Introduction:
The session took place normally on the Zoom platform, with opening words from the then-ADG Sr. Julio Berdegué and an introduction from the Statistics Division (ESS) Director, José Rosero. Subsequently, the audience was divided into working groups, to review in detail the different domains of the FSS Indicators. To close, a plenary session was held in which the moderators summarized what was discussed in each group.

Through a formal letter from the FAORs and RLC leaders, 270 people were invited to participate in the consultation, from more than 20 countries in the region. 244 of them were partners (especially government), while 26 were people from FAO and speakers. On the side of the invited UN institutions, there was ECLAC (with 7 guests) and IFAD (with 4). Before the event, 64 people confirmed their participation.

On the day of the event, there were more than 100 participants, and the range varied between 70 and 100 people, remaining stable among all blocks.

IT technical support, simultaneous interpretation and recording arrangements were provided. Each working group had a moderator and a note-taker from RLC, as well as a speaker chosen by ESS.

Opening:
Mr. Berdegué spoke about the challenges in relation to the concept and ways of measuring the transformation of agri-food systems. He pointed out that it is a broad concept, involving different types of actors, and all types of domains.

For Mr. Berdegué, the transformation of agri-food systems is a phenomenon that affects several dimensions, from the individual behavior of consumers to relationships with the environment, health institutions, the adoption of technologies, etc.

A key question was raised: "How to know if the actions that are being carried out – by civil society, governments, etc. – are contributing to the transformation of agri-food systems in the right direction and in a consistent way?" He concluded by pointing out that it is essential for the transformation of agri-food systems to have good analytical frameworks, to move from the conceptualization to the operationalization of these concepts.

Presentation of Themes

<u>Group 1: Diet, Nutrition, and Health Domain</u>
People were in the center. The objective of the exercise was to discuss several indicators related to diet, nutrition and health. To do this, the analysis needed to focus on different areas to measure people consumption (diet quality).
Nutrient adequacy and dietary risk factors for NCDs had to be measured, as well as the accessibility to healthy diets (food security). Other important issues were: food environment (availability, affordability, messaging, and food & vendor properties); and to answer whether policies contribute positively or negatively towards food availability, food access, and product properties (policies affecting food environments).

Finally, a little disclaimer was raised: “Why don't we have measurements like weight? Because those indicators are also affected by other contexts and factors”.

Group 2: Environment and climate Domain
The “Environment and Climate Domain” group had 5 areas.
What is to be monitored? Food systems are a major source of environmental degradation, the actions required to achieve global environmental commitments, monitoring and accountability are essential.
The definition and the candidate indicators for each area of the “Environment and Climate” domain were presented: land use, greenhouse gas emissions, water use, pollution, and Integrity of the Biosphere.

Group 3: Livelihoods, poverty and equity Domain
The topics related to the group were introduced; these are: Poverty and income, employment, social protection and rights. Cross-cutting issues were also pointed out: governance, resilience and sustainability, emphasizing their importance.
The topics and problems that were sought to be measured were presented: the number of people who are part of the food system, in rural and urban areas, considering their diversity and also considering the poverty and vulnerability of those who work in these sectors. The definition of each one was given, and the questions were asked.

Group 4: Governance Domain
The objective of the meeting was to obtain feedback on the governance indicators’ component, focused on the context of our region. Governance had four components: joint vision, policies and strategic planning, effective implementation, and accountability.
What is to be monitored? First, the common vision of the outcomes, relevant policy instruments to align efforts, the implementation of resources, and accountability for the outcomes.
The joint definition of governance is: inclusive and participatory processes that allow to identify priorities and to generate a guide with desired results. For this, different actors, civil society, relevant actors from the academy and decision-makers are involved.
To do this, it is necessary to measure power imbalances (market, influence asymmetries, concentration and market power).
The possible indicators are the following: Index of civil society, variants of democracy, and presence of a national food transformation system (strategic planning).

Group 2: Resilience and sustainability Domain
Resilience and sustainability were presented as a transversal aspect of the transformation of agri-food systems.
The subdomains are exposure to shocks, resilience capacities, agrobiodiversity, food security stability, and food system sustainability index. This issue is critical for food and nutritional security (for example, as states become more fragile, there is more insecurity, or the context of covid 19). It is critical for other functions: resilience is a precondition for sustainability; it has a multidimensional interpretation. The definition and candidate indicators were presented.

Discussion

- Some aspects to consider in the region are: retail trade, formality and informality, dependence on international trade, frequent natural disasters in certain areas (droughts, hurricanes, tornadoes, etc.), family farming, gender, generational change, among others.
- Cluster analysis idea is repeated in several groups.
- Considering demand factors, such as consumption habits, is highly relevant. The use of technologies and innovation in food systems should be considered. The financial support and inequality of countries should also be included, as well as the biodiversity of the production area, the trend in increasing food imports, crop productivity levels, and food losses and waste.

- Are indicators that are already on the SDG list being considered? That thinking of facilitating the follow-up of the countries to the indicators, and not repeating it...
- The indicators represent a great advance in the construction of measurement, but there is not going to be much correlation in an indicator that measures something in a construct (environment, or diets), and that again in another construct returns to exist in a similar way.
- Lack of data and availability of information in the region. For example, in the Dominican Republic, agricultural censuses have not been carried out since 1982. The absence of updated data is a problem for the formulation of good policies. There are NGOs that have good data, and good use could be made of it.
- An immediate task for LAC should be to make an inventory of available variables and indicators based on official information; multilateral organizations must draw on official information. Some national statistical systems are stronger than others in LAC. Many times, the measurements that are made are not published, due to political implications. The information exists.

<u>1. Diets and nutrition</u>

Do you consider the proposed indicators relevant, high-quality, interpretable and useful?

There are three repeated discussions: 1) among the proposed indicators, there are indicators of different levels and hierarchies (of processes, which seem to be inputs from others, of results, and whether or not impact indicators should be considered). 2) The aggregation capacity of these indicators at the sub-regional, regional and global levels, but also at local, territorial levels, to groups of certain ages, from different areas. 3) The data and data update frequency, the quality of the information.
With regard to food environment, governance indicators such as food supply in schools are often included, which is directly related to the food environment at school level. Therefore, it is suggested to adapt the definition, in such a way that it is not confused with governance aspects that directly aim to change the food environment.
The indicators of accessibility to healthy diets and the cost of healthy diets are very interesting and necessary, but it is important to consider countries that do not have data associated with this. On the other hand, the indicators are highly concentrated in food consumption and availability, but there are not many biometric or anthropometric indicators, which are very important having sustainable food systems.
It is pointed out that there is information associated with malnutrition due to excess or deficit; and this can be interesting to include, to think about medium and long-term indicators.
The healthy cost indicator seems very important; however, again the data gap is a big issue (for example, there is an absolute data gap in the case of Venezuela). On the other hand, the inclusion of food in social programs is very important and the use of instruments such as vouchers only for fruits and vegetables can make a big difference. The zero fruits indicator seems very relevant.
Finally, it would be necessary to make an ordering through the three elements of the food system (political, sectorial and individual), and to recognize which the process indicators that are required to achieve certain health are (and those of impact in each of these areas).

What are the data gaps that you can identify? Are these gaps structural? If not, are there some data or indicators that you know are available in your region and can be used for the purpose of this monitoring and assessment system?

It is suggested to incorporate the issue of policies, laws, strategies, regulations, which may be affecting the results and possibilities of transforming food systems, and also indicators that capture food loss, which are absent.
There are gaps in terms of physical access to food, the availability of food at nearby points. This is relevant and there are no indicators of this.

On the other hand, the type of place where food is purchased, countries that have a greater penetration of supermarkets, versus more informal fairs, etc.; this generates a substantially different food system, and it is interesting.

The main concern is the availability of data for the indicators. An example of this is the data on the costs of the diet for the Caribbean, how can this information gap be managed in the different countries of the region? On the other hand, it is suggested to think about how to involve the countries more in the process of collecting this data.

In addition, there are limitations for updating impact indicators, and there is also the issue of being able to disaggregate by region. There are big gaps in data and the time taken to collect this data is varies between countries.

It is very important to consider the food environment. There are two very different educational systems: the private and the public. The public system follows a menu for the school diet, but they are low-income children. It would be very good to monitor the effects of the policy changes that are needed to generate more agile ways of obtaining these nutrition indicators.
It could be interesting to address the measurement of food spending. On the other hand, it is also interesting to consider what happens with the eating behavior indicators.
Regarding child nutrition, it would be good to consider not only the child of breastfeeding age, but also beyond 23 months. It would be necessary to see other spaces to obtain more information to add these indicators.

What are the regional aspects we need to take into consideration at the moment of selecting a set of indicators to monitor the state of food systems and its evolution?

It is very important to consider the availability of data for indicators. In operational terms, the information needs to be disaggregated, not all regions have the same information. In terms of policy, it is necessary to consider the differences between provinces, departments, vulnerable groups; therefore, disaggregation is something that must be included in this proposal.

The cycles and food supply are different among countries; this should be something to take into account. Regarding food security policies, there is no a Central American level policy, based on the restructuring or relevance in data collection.

Caribbean countries are complaining that, over the years, the food system hasn't been well-developed and that the import bills are getting more expensive every year. So, the questions are raised: "Is it possible to have indicators on this?", "Is it possible to have indicators related to imported food bills, so that countries can monitor this and take appropriate measures?"

<u>2. Environment and climate domain</u>

Do you consider the proposed indicators relevant, high-quality, interpretable and useful?

Specifically on the indicators, the following question arose: "what is the need or usefulness of differentiating an indicator of net emissions of greenhouse gases, and those of intensity?" It is considered that since there are different production systems throughout the region (with countries with high production intensity and others that are dispersed in small areas), the intensity indicator is one of the most relevant.
The indicators are not as rigorous as they would have liked.

Concern that selected indicators are not highly correlated.
Ease/pragmatism and follow-up skills are needed.

What are the data gaps that you can identify? Are these gaps structural? If not, are there some data or indicators that you know are available in your region and can be used for the purpose of this monitoring and assessment system?

- About the gaps: regarding land issues, there are two key indicators. One indicator is land degradation (which can be formulated as the % of land in relation to the total area), and this indicator can be divided into 3 indicators, which are those used in the convention to Combat Desertification and in SDG15: Vegetal Cover, productivity of the land, and soil organic carbon. That is essential, given the degradation of the land.
- In terms of gaps related to water: water stress (hydric stress), water availability (surface and groundwater), and water pollution. There is concern over soil contamination, but not about water.
- In the initial presentation, solid waste was discussed, but that was not being visualized in the dimension of contamination. On the issue of food waste, an indicator on the generation of this type of organic waste could be considered, which in any case is already contemplated in goal 12.3 of the SDGs, and some countries are already beginning to work on it. On the other hand, the entry of organic waste of this type to a sanitary landfill could be considered.
- In terms of pollution, as is the use of pesticides per unit of land, it is suggested to incorporate an indicator of the total bio-inputs on the land -natural, organic- (to think positively).
- It was asked why food loss and waste was not included in the indicators.
- There is concern regarding the Deforestation issue (It is not explicitly included in the proposed indicators, and it is one of the key elements of the expansion of the agricultural frontier in various countries, and especially in the Amazon region). Deforestation is reflected in 2 parts of the proposed indicators: 1) expansion of the cultivated area; 2) In relation to the issue of greenhouse gases, it is suggested to verify if once deforestation has been carried out in an area to expand crops, that remains in the proposal.
- Concern regarding the indicator "Nitrogen management index". It is much more interesting to include an index of sustainable carbon management in the soil, since nitrogen is part of organic matter.
- It is suggested to include an indicator related to agroecology, which is very important for production.
- On the subject of climate variability, it is suggested to include indicators regarding Climate Change and production: changes in rainfall patterns, changes in seasonality (there are areas where the dry season was short and now it has extended), intensity of drought, to name a few.
- Functional integrity within food systems can go further. Biologists and conservationist work on landscape fragmentation indexes, which are possible to calculate with secondary information, and give a much better estimate than just the % of cover.
- Diversity of ecosystems should also be included.
- There are no indicators of the increase in pests and diseases as a result of climate variability that leads to an increase in the use of pesticides.
- It would be good to include an indicator on Circular Bioproduction in relation to proposing alternatives for sustainable agriculture.

What are the regional aspects we need to take into consideration at the moment of selecting a set of indicators to monitor the state of food systems and its evolution?

- Regarding the data needs in the region, there are biodiversity characteristics that must be considered in the construction of the indicators. Climate variability and heterogeneity and their impact on agriculture were also mentioned.

- One participant points out that small farmers are not prepared for the weather effects that occur in the region. They do not have a contingency plan to prevent these effects. In the Dominican Republic, for example, a donation plan is beginning according to weather stations, to give them the appropriate support. The importance of considering the issue of vulnerability due to the CC effect within the indicators is highlighted.
- About carbon, a worry is that its behavior is very variable depending on where it is. In Costa Rica, for example it is very difficult to see increases because the organic matter materializes a lot due to the tropical soils.
- On agroecology, there is a recent proposal that emerged at the Food Systems Forum. It is suggested to take into account what experts on this subject recommended there.
- When there are extreme events, food security disappears. It is worrying that the proposed indicators focus on FF emissions, and not on the production systems of the countries that emit the most.
- On issues of biodiversity and ecosystem integrity, there is a comment that has to do with the % of natural area indicator: there are ways to measure the integrity of landscapes, measuring the patches, the distances between patches, and this can give a better vision of the areas that are involved in productive sectors. Another issue is the security of these areas. It is not enough for the areas to exist, but for there to be certainty about it, a management agreement with the communities, for example, is needed.

Others:

- The need to see if there are indicators of the total or partial use of agricultural inputs (fertilizers, pesticides) arises.
- The management of fruit trees stands out as a good indicator; that is directly related to peatlands, and these are directly related to CC mitigation.
- It is marked that FAO has a new methodology developed together on the Multidimensional Assessment of Agroecological Systems, based on 10 indicators. It is suggested to analyze and include them.
- There is a concern, which is the efficient use of water, or re-use of water. There are areas of our country with a lot of water deficiency. Using water in several productive units can be very good (for example, the experience with fish farming and vegetable production.)
- There is a voluntary code for the issue of food loss and waste, and it is pointed out that some elements of monitoring could be considered beyond strictly the FLI or FWI of table 12.3 of the SDGs. Some more qualitative elements may emerge, but they could add up in terms of initiatives or preferred actions as an indicator https://www.fao.org/3/nf393en/nf393en.pdf
- Some key indicators on water issues: water availability, rainwater changes, groundwater availability, water pollution, water stress.
- There is a new framework developed by the United Nations Statistics Division: link.

3. Livelihoods, poverty and equity Domain

From the nature and regional reality, some definitional issues are raised in relation to the role of family farming, the role of food systems. The contribution that agriculture makes to the economy is also mentioned; issues of inflation, GDP, expanded agricultural participation are discussed.

Do you consider the proposed indicators relevant, high-quality, interpretable and useful?

- The distinction between more or fewer indicators arises, with the risk that if there are more indicators, they may be contradictory to each other. The idea of a brief index or of forming clusters arises. On the other hand, assess whether there are indicators that are more relevant than others.

- The availability of information, censuses, statistical institutes, incorporate studies and data from NGOs, academic institutions, and other United Nations organizations were discussed.
- The dilemma about what can and cannot be done in terms of measurements and available information emerges.
- It is pointed out that it is better to use simple indicators than indices (indices become complex and feel unclear).
- It is stated that including many indicators at all levels (impact, results, outputs and inputs; according to theory of change), several can cancel each other out. The cluster exercise is key.
- In the same way, it is not recommended to mix so many different levels of indicators: there are final objectives, intermediate objectives, immediate results and inputs, and, in addition, policies (it could be very confusing, and cannot be distinguished where the growth, the production, the trade is).
- It is suggested to know what the expenses for agriculture and expenses for food systems are.
- It is recommended to reduce the number of indicators. A cluster analysis should be considered.
- The existence of external macroeconomic factors is mentioned. It would be a mistake to associate poverty with agriculture or with agri-food systems that are still developing.
- The question arises: "How is the issue of double causality going to be addressed?" (Lower levels of poverty contribute to further development of FSS, and more developed FSS also contribute to poverty alleviation).
- It is noted that the characterization of agri-food systems (those that are less developed, with less added value, etc.) was not contemplated.
- The indicator "Recognition in the Constitution of the Right to Food" could be included in the group of indicators for the topic of Governance.
- A dialogue was developed around including the indicator of participation of agricultural GDP in GDP. This could generate incorrect interpretations, because participation falls with the level of development of the countries and is sometimes associated with less importance of the agricultural sector, and higher participation is associated with higher levels of poverty.
- Based on the above, the question arises as to what indicator would be proposed and what it would include. "If it were included (with the problem that data is not available for all countries), the indicator should be the share of extended agriculture (agriculture + sectors linked to agribusiness, agribusiness and food). The traditional agricultural GDP only captures production at farm level."
- Interpretability criteria is not clear in terms of direction and context on the indicator "households of significant income from agriculture."
- Another issue is the distribution of land ownership by sects (or sectors?). Currently, the definition of the indicator does not represent all land holdings (only agricultural land). Therefore, it is noted that the title should be adjusted. In addition, for the data sources for this indicator, there is more than just the agricultural census because agricultural surveys also collect information on this, and in some cases, household surveys with agricultural modules collect data on this, too; there, data sources could be expanded. For the coverage of school feeding programs, there is no clarity on the coverage of this indicator in the region.

What are the data gaps that you can identify? Are these gaps structural? If not, are there some data or indicators that you know are available in your region and can be used for the purpose of this monitoring and assessment system?

- It is indicated that there is nothing about the contribution of food systems to economic growth, and that is the main "trade off" that can be carried out for transformation policies and processes. "We need to know if the proposed political trends are going to have an impact on economic growth. Employment is measured, but that is not direct enough."
- There should be indicators on the budget allocation that countries have, specifically for the issue of sustainable agri-food systems. "You have to look at the other groups of indicators, as well as

the government budget allocations. See how to link government development programs and public policies to country roadmaps.”

- In relation to the income indicator, the direction is not so clear; only agricultural ownership is considered. Agricultural censuses should be considered, and data sources expanded.
- Regarding data gaps, it was proposed to include the contribution of agriculture to GDP as one of the indicators. Also, to include some productivity indicator, to see if the agricultural sector is becoming more productive.
- The agricultural orientation index is available for all countries using national accounts’ data, and this would be a good indication for how much of government expenditures are going to agricultural programs.
- Lack of explicit indicators on access and appropriation of technology. This access must be generated in an inclusive manner, so that there is no greater gap between rural inhabitants.
- It is proposed to create a funnel system and to have sets to order and find out which the most common indicators in the countries are and which could have the desired comparability. Making a chain of results to organize and have the indicators that are most relevant is also mentioned.
- “It would be very important for the FAO to have economic, productive, and social indicators for all the countries. As an investment for agriculture, a budget for agricultural innovation, nutrition, per capita consumption, etc.”
- It is proposed to delve into the gender gap within the indicators: “especially how employment is defined within rural women; many statistics do not consider all the activities carried out by them as ‘employment’, and they are in the inactive category. In Colombia, rural women are the ones who work the most hours without any income and when it is paid, the gender gaps are larger than in urban areas.”
- It is suggested to incorporate ECLAC indicators of poverty and extreme poverty, which is calculated for rural and urban areas, and the gaps could be observed.
- It is suggested to build specific indicators for the group that account for the sensitivities of the different households.
- The issue of income has a lot to do with distribution within the agri-food chain; for example, rural and agricultural producers are being affected by the prices of production. One could try to see this problem within the indicators: how is value distributed within the food chain? Try to value, for example, the natural resources within each of the chains. How to deal with exports? Exporting ecosystem services; water is being exported, natural resources that in many cases are not going to be renewed.

What are the regional aspects we need to take into consideration at the moment of selecting a set of indicators to monitor the state of food systems and its evolution?

- The needs of the region must be taken into account, highlighting that family farming is not included in the indicators presented. "From the Dominican Republic it is mandatory that, when talking about food systems, family farming is considered."
- Family farming: the idea that there is nothing about the contribution of food systems to economic growth is reinforced, and this is the main challenge for many policies and transformation processes. “We need to be able to know if certain political trends are going to have a certain impact on economic growth. Employment is being measured, but that is not enough. It should include the economic consequences.”
- It is pointed out that within the measurement of the indicators that are being proposed, there is an invisibility of subsistence family farming in rural areas. They are part of the negative effect of this system, since they are the most malnourished and those with the least possibility of production and the most difficulty in obtaining accessibility. Difficulties arise when using indicators that are not linked to the issue of poverty. The importance of making visible what governments are doing to include families in these areas is highlighted.

- Food security for the migrant and refugee population is considered an immediate issue. “Many of these people are excluded from formal systems and are also often reluctant to participate in surveys because of fear of persecution/prosecution in places where they do not have adequate social protections or permissions to work legally. Given their lack of formal inclusion, is there a framework in place to ensure their conditions are considered? From our perspective, food security is of paramount concern for this community, especially facing rising inflation and recessionary economic conditions.”
- Information on urban population should be incorporated. There is a fairly strong bias towards agriculture, “food systems are beyond agriculture, and an important part of the economy is in the postproduction of food systems in many Latin American countries.”
- How to capture the income, jobs, employment conditions in the part of retail, wholesale, formal and informal trade, and exports? “As a region, a lot of food comes out, how will that be considered?”
- "In countries like Colombia, the countryside is becoming uninhabited and that puts food supply at high risk at the internal level." There is an issue of generational change and the legal occupation of the territory. There are people who are being displaced from their natural habitat.

Others:

- One participant suggests that in the specific case of poverty, equity and life systems, development indicators go beyond food. As an example, a classic indicator of development is "infant mortality rates, which is not directly related to food systems.” It is suggested to learn from UNDP. However, it is known that there is incomplete data for many countries (for example, multidimensional poverty, many countries do not report it). National statistical systems are very weak and incomplete, and that is the reason why non-governmental organizations are emerging. It is recommended to see to what extent there is a possibility, in some countries, of taking reliable indicators that are constantly measured by NGOs as an alternative.
- It is talked about the topic of crime in the rural sector: “sectors in rural development, stressed by climate, economic problems, etc. This ends up generating migration, which creates empty spaces and that's where crime comes in, which, in return, feeds back the problems of stress and migration. This does not only happen in LAC, but also in Africa, and if we are talking about livelihoods, one of the most serious problems they have is the lack of crime and violence control systems in rural sectors.”

4. Governance Domain

Do you consider the proposed indicators relevant, high-quality, interpretable and useful?

The indicators are not evidently clear to the group.
A participant points out a reflection: from a conceptual point of view, the indicators seem fine. The issue is that it is logically ordered, but it is not very operational; it does not refer specifically to the agri-food sector. In addition, the indicators are based on people’s opinion; there is no hard data. Therefore, there is no comparison. So, it is important to generate indicators that can be used as hard data. One solution to this can be to think about how to formulate the question; for example, a specific question can be: did you or someone you know have to pay a bribe to the police? It is a yes-or-no answer. This type of question could be hard data. What would be useful then? An institutional analysis of the entire system that is around the agri-food system, which does not exist right now, because they are all separated into different ministries. It is suggested to consider, when using these indicators, that this is a sensitive issue for governments, especially when there is no hard data, because all the indicators on the institutional level at the international level are perceptions. It is important to build the most objective data possible.

The importance of focusing on the institutional framework in general is highlighted (what the coordination mechanisms are, the plans and the budget). A vision of budget analysis with a food system perspective is lacking, and at the other level of effective implementation, it is necessary to check if what has to be done is being done. Perhaps it would be necessary to go a little further and review the institutional system, which is what is needed to give long-term sustainability to the desired changes in agri-food systems. It is suggested to review the intersectoral plan in the countries, clearly indicating what each institution contributes and does and how this is translated into an institutional plan.

Finally, there are doubts about the interpretability of the indicators, about whether it will be a faithful reflection of situations with better governance for the transformation of agri-food systems.

What are the data gaps that you can identify? Are these gaps structural? If not, are there some data or indicators that you know are available in your region and can be used for the purpose of this monitoring and assessment system?

- It is proposed to pay attention to consultation mechanisms for consumers, producers and family farmers, and also for vulnerable groups that go unnoticed (native peoples). Many countries have many mechanisms, but they are not effective, they do not translate into dialogue processes, or coordination between actions of public sectors themselves. This should be taken into account.
- It is recommended to accompany the indicators with an institutional analysis (graph what is the institutionality that exists in the countries for the government of agri-food systems).
- It is desirable to incorporate public spending variables and an analysis of effective policy implementation regulations.
- The lack of data for several countries in the region and the periodicity of this data collection (regarding the inclusion of indigenous peoples or consumers, there is not enough international data to have them as indicators) is pointed out as an important gap.
- It is pointed out that governance indicators are a challenge for many countries in the region, since the institutions and laws are not always applied, for example, Venezuela.
- It is indicated that the region has a problem at the level of dialogue in agri-food systems and chains, and there are no laws that stimulate dialogue between the actors of the system and the agri-food value chains.

What are the regional aspects we need to take into consideration at the moment of selecting a set of indicators to monitor the state of food systems and its evolution?

- Regional peculiarities were identified: corruption, informality, limited capacity of public sectors to implement decisions, and policies that are made.
- A participant indicates that in the region, the generation of dominant actors is usually sought (in all institutional relations and among the various actors of the society itself). In practice, these actors subordinate the true peasant and productive representation; this creates an environment where small producers are no longer heard. Therefore, it is suggested to plan actions so that this power does not end up submerging those small producers, who are the ones who generate the production that guarantees the food consumed in the country.
- It is suggested to consider that the existing indicators generate controversy.

Others:

- A suggestion is made: the indicators should be comparable across countries and over time, but that does not mean that discrete indicators are no longer important. Proxies must be used. For example, regarding the budget issue, the countries have defined agricultural spending for their countries; this expense is a % of the budget, of the GDP. This can be important information, hard

data. There is also information on agricultural financing and financing for small producers that can be measured annually -this is to mention some indicators that are measurable over time.

- It is suggested to design an indicator that measures the relevance and functionality of the institutional offer.
- It is also suggested to have data on the sustainability of projects implemented by governments ("when a project is installed, after the management is over, the project is lost; and this is worrying"). It is recommended that one of the indicators focus on government effectiveness. Related to this, it is also proposed to design an indicator that measures the relevance and functionality of the institutional offer.
- It is pointed out that one of the missing indicators for the region is the issue of corruption, and the voice of consumers and users when it comes to accessing quality food, because when implementing the laws, it is important to measure consumer satisfaction. Generally, international NGOs work to improve production, with different tools, but it is not seen until the end of the process (once the customer buys the product, knowing if there are problems, where to complain, to whom they can express their satisfaction or dissatisfaction).
- It is mentioned that, at this time, the countries compile the information based on the thematic classification of the monetary fund in which agriculture appears, but not what is being spent on agri-food systems in general. It is suggested that countries have satellite accounts based on this approach, which will allow for something to define what is important. It is also pointed out that the same thing happens with the regulations (for example, the USA has the code of federal regulations, where is possible to see all the federal regulations that exist, and this would be very useful for the agri-food sector).
- In another topic, it is pointed out that part of the problem of non-effectiveness is the non-continuity of operations and the fragmentation of operations. It is highlighted that there are many projects that cover very few people, leave large gaps or often compete for the same beneficiaries, but since no one does a global mapping, there is duplication and little coverage. In addition to that, there is discontinuity; projects are often completed because there are contracts with entities that are later rescinded. One proposal that is presented is that, in addition to the indicators mentioned, more institutional data and more qualitative data with identification recommendations be generated, with alternatives (satellite accounts, for example). With this there will be no comparative tables between countries, but it will be possible to have a vision of their governance schemes and the impacts on agri-food systems.
- A participant wonders if, taking into account that the article talks about systems theory, there is an interrelation between the components of the system.

<u>5. Resilience and sustainability Domain</u>

Do you consider the proposed indicators relevant, high-quality, interpretable and useful?

- It is pointed out that something important to begin with is to define what is meant by resilience within agri-food systems. That is a starting point to understand the proposed indicators.
- It is mentioned that the definition of food system resilience is the capacity of the different individual and institutional actors of the food system to maintain, protect or quickly recover the key functions of that system despite the impacts of disturbances. Link.
- One participant comment that resilience is easy to describe and pronounce, but difficult to measure; its interpretation is highly variable.
- A participant asks about the methodology: having indicators at different levels, how is this synthesis exercise going to be carried out? Are you thinking about cluster analysis? When there are many indicators, they can cancel each other out.
- There is concern regarding the indicators. In the case of Colombia, the armed conflict and migration determine the transformation of agri-food systems.

- The concern that the concepts do not overlap (resilience capacities and resilience strategies) is presented and repeated. Information is lost when it is not easily understood. It is necessary to avoid redundancy.
- It is recognized that the major limitation to this process is the availability of data for most countries.
- One participant note that he agrees with the text's definition: “resilience is the capacity to ensure that stressors and disturbances do not have lasting adverse consequences for development”.

What are the data gaps that you can identify? Are these gaps structural? If not, are there some data or indicators that you know are available in your region and can be used for the purpose of this monitoring and assessment system?

- It is recognized that indicators referring to food agrodiversity, produced and consumed, are taken into account, but the importance of talking about food biodiversity produced at the country level is recalled, because there are countries that depend a lot on imports, but little is said about efforts made by the country for biodiversity.
- Given that the Sustainable Food Systems Program exists, and that these consultations are taking place in other regions of the world, it is suggested that the co-leaders of the Program be included in this invitation.
- The conceptual proposal is recognized as good, but some gaps are identified: 1) despite how complicated it is to define resilience, whenever you want to measure something, it is essential to define and agree on what is meant by each concept, 2) there is no consideration of economic shocks (current context of Eastern Europe, which has involved export restrictions). It is indicated that measures are now being tested to interrupt the normal flow of trade, which has a severe impact on the agri-food system. The idea of diverse agri-food systems, which is related to local trade versus exports, is valued, but it is suggested to add indicators that measure a) diversification of consumption, b) diversification of production (diversifying more will help resilience), and c) trade diversification.
- Along with the volatility of domestic food prices, there could be another similar indicator for inputs (fertilizers, insecticides, labor, etc.).
- It is highlighted that the volatility of food prices is an aspect of affordability, but also of income, savings, etc.
- The importance of considering the climatic shocks that are often experienced in this particular region of the Central American Caribbean is highlighted.
- For purposes of resilience in the purchasing economic sphere, and for decision-making, the importance of considering indicators that exist in El Salvador is highlighted: Indicator of the purchasing power of wages (if purchasing power drops, wages must be adjusted, so that the poorest families have greater purchasing power). This indicator is not visible in the proposal.
- The indicator of the degree of dependence on imports of basic grains is not observed. It is emphasized that it is important to know which foods are depended on... Another indicator of foreign trade is the % of total exports dedicated to importing food for the basic basket, which was not on the list either.
- There are gaps in terms of contamination, it is important to define it much better. The question is: What are the practices that are based on nature that are going to be identified as useful for the sustainability and resilience of agri-food systems? How the implementation of topics such as agroecology could help the nitrogen concentration of certain cultivars? The idea would be to make a filter thinking about the ecosystem services that these solutions provide.
- About diversity: the importance of identifying which are non-native or non-local species is highlighted. Introduced species can help a territory economically, but their impact can be significant, killing some local species. It is key that this biodiversity indicator can be adjusted taking into account the non-promotion of the introduction of non-native species.

- It is important to have an indicator to know when an economy goes from one equilibrium to another, an indicator that shows: if "x" amount of food was produced, and a hurricane comes, "x" less is produced, showing statistical changes relative to an initial reference point. In this way, the concept of resilience could be better understood.
- It is commented that **resilience is not only individual, but collective,** and there is no indicator that shows this: the formation of new organizations, for example.
- The following question arises: what will be the weighting of each of the indicators, which together add up to resilience?
- A participant states that he does not see indicators of resilience. He understands that resilience is a product of native biodiversity. He works in Peru, where it is important to work with Amazonian fish. They do not depend on imports, but on the technology that has been developed in the area. He suggests incorporating an indicator that reflects the use of local species (fish, plants or animals).
- On the gaps: measure of dependency or integration with global markets. It may be affected if there is an international introduction.
- There is an issue that has to do with the volume of food that does not go through informal commercial relations. For some populations it is highly significant to have more informal exchanges: for example, the family that sends cheese or beans. The following question arises: “To what extent are we clear about how important this form of provisioning is? And if it is important, how can this be captured?”

What are the regional aspects we need to take into consideration at the moment of selecting a set of indicators to monitor the state of food systems and its evolution?

- From the regional point of view, there are issues that are inherent in the Caribbean zone, which have to do with vulnerability to dependency on food imports and inputs for agriculture and, at the same time, vulnerability to climatic shocks (hurricanes).
- Dependence on food and imports is an issue that concerns the entire region.
- A mention is made about the SICA area: it is a large staple food market that should be promoted. The resilience of the region lies here. It is recommended to consider all this. In addition, it was pointed out that agricultural variability and livestock activities, which sustain the countries' food, should also be considered.
- It is pointed out that it is important to consider the definition of food sustainability from the nutritional point of view. How, from the region, is it being produced healthily? (With fewer transgenics and reduction of agrochemicals).

Others:

- Discussion around the purchasing power of money: to what extent domestic and international shocks have a local impact through the economic part, purchasing power and the ability to ensure the basic food basket.
- It is commented that in Peru there is a great commitment to the indicators, and to the SDGs. However, the concern arises as to how to ground the theoretical framework to more specific tools. It is requested to illustrate with practical examples (Excel sheets, etc.), and exemplify the sources of information, in order to comply better.
- The INTA (Nicaragua), with the Agrarian University and the Movement of Agroecological Producers developed a resilience assessment methodology, based on the Stoplight method, which could be shared. The Methodology is called REDAGRES.
- It is recommended to have up-to-date and quality data to identify and address the problems faced by food systems, such as distortions at the level of production, the marketing chain and consumption.

- The question is raised: Are the policies or market and regulatory incentives that countries have to encourage the adoption of environmentally sustainable practices that are profitable at the same time contemplated?

Agenda of meeting

Agenda:

**10.00-10.05**	Opening	*SDG and RR of RLC Julio Berdegué.*
**10.05-11.15**	Introduction to the Initiative	*José Rosero, Statistics Division (ESS) Director.*
**11.15-12.15**	Work sessions in 3 breakout rooms:<br>1. Diet, Nutrition, and Health Domain.<br>2. Environment and climate Domain.<br>3. Livelihoods, poverty and equity Domain.	 *1. Nancy Aburto.*<br>*2. Mario Herrero.*<br>*3. Rashid Sumaila.*
**12.15-12.30**	***Break***	
**12.30-13.30**	Work sessions in 2 breakout rooms:<br>1. Governance Domain.<br>2. Resilience and sustainability Domain.	 *1. Simón Barquera.*<br>*2. Ty Beal.*
**13.30-13.45**	Closing and next steps.	*José Rosero.*

FAO RNE Regional Expert Consultation of the Food System Countdown Initiative's Indicator Framework
Virtual, May 18 2022

This report was produced by the FAO Regional Office for the Near East and North Africa to summarize the results of the FAO RNE regional expert consultation held on May *18* 2022.

Introduction

Food systems play a role in meeting all 17 sustainable development goals (SDGs). With less than a decade to achieve the SDGs, the global community faces a critical juncture to transform food systems to be healthier, safer, more sustainable, more efficient, and more equitable. Lately, the UN Food Systems Summit has focused global attention on food systems and set the stage for food system transformation. Country and independent dialogues catalyzed the development of shared visions for food systems that apply to different contexts and geographies.

It is widely recognized that to enhance all aspects of food systems and their interactions, a clear, rigorous, and comprehensive set of metrics and indicators are required to guide decision-makers and to hold them accountable. However, no rigorous mechanism currently exists to track the state of food systems, their change, and performance over time. In fact, the commitments reached at the UN Food Systems Summit, and the realization of the visions reflected in the national food systems pathways need metrics to guide decisions and track progress. At the same time, food system actors and stakeholders (e.g., civil society, governments, and international organizations) require trustworthy, science-based metrics and assessment.

Food Systems Countdown Initiative

With the ultimate objective to fill this gap, the Food Systems Countdown Initiative ("the Initiative") was formed in 2021 as a comprehensive, independent, inclusive, science-based mechanism to provide actionable evidence to track progress, guide decision-makers, and inform transformation. At the same time, it intends to complement other monitoring mechanisms and the tracking of related goals at global and regional scales (i.e., SDG agenda CAADP).

To implement the Initiative an unparalleled partnership and collaboration have been put together, led by FAO, GAIN, and John Hopkins University and with the participation of more than 50 scientists from nearly 30 academic institutions, non-governmental organizations, and UN agencies from nearly all continents.

Independent tracking and assessment system

The main goal of the Initiative is to provide an independent tracking and assessment system based on a high-quality, curated, parsimonious set of indicators that cover all important aspects of food systems and measure food system performance. The Initiative/FSCI has designed an architecture for such a system from a multidisciplinary point of view and is moving towards implementation.

The Initiative expects to deliver an annual assessment of the state of global food systems and their transformation, published in a peer-reviewed scientific paper. It is also envisioned that policy briefs will be delivered in parallel for a broader audience and to facilitate transformative action.
The first milestone of the Initiative was the publication of the initially proposed architecture of the system and the description of an inclusive process to move from the concept to its execution.[9] The architecture covers the five thematic areas of diet, nutrition and health; environment and climate; livelihoods, poverty and equity; governance and resilience and sustainability.

[9] Fanzo et al (2021).

As a second step, the Initiative will aim to deliver the set of indicators in each of the five thematic areas above and to deliver a first assessment of the state of global food systems that will serve as a baseline for monitoring progress and performance.

The proposed tracking and assessment system are an independent effort which do not represent any obligation or commitment for reporting from countries.

Objectives of the RNE Regional Expert Consultation
The Initiative is committed to an inclusive, consultative, and transparent process that will allow for validation and peer review of the set of indicators that will be used for the assessments. Two sets of consultations have taken place. The first was a consultation with expert scientists. The second, a series of regional expert consultations across the FAO regions, including this Regional Expert Consultation for the Near East and North Africa Region.

The Regional Expert Consultation for the Near East and North Africa Region brought an expert point of view from policymakers and policy-adjacent users of data, on the relevance, usefulness, and validity of the proposed set of indicators from a regional perspective. The consultation covered the proposed indicators in each of the five thematic areas.

It provided an opportunity to get inputs, comments, and suggestions on the monitoring framework proposed by the initiative. The framework is voluntary, however, the consultations will ensure that it has the capacity to be a useful tool for policy decision-making processes. The results will be used for the first assessment of the state of global food systems and later for tracking progress and assessing performance.

The FAO RNE Regional Expert Consultation of the Food Systems Countdown Initiative's Indicator Framework was held virtually on May 18 2022. It brought together representatives from governments in the Near East and North Africa region, as well as individual experts in food systems from the FAO network in countries. Participants were drawn from senior staff engaged in policy development from the relevant Planning Units of the Ministries of Agriculture, National Statistical Offices and Ministries working on the thematic areas of the indicator framework. As well as senior experts from international, regional and national agencies in the region from across the five thematic areas.

Opening
The opening remarks by FAO Regional Office Assistant Director General (ADG-RNE), Mr. Abdulhakim Elwaer, highlighted the importance placed on monitoring of food systems at the UN Food Systems Summit held in New York in September 2021. He highlighted the importance of food systems transformation in achieving sustainable development and the SDGs. It was underlined that transformation is needed across four key areas of food systems transformation highlighted from the Food Systems Summit for the which the Near East and North Africa Region. Namely, hunger, food security and nutrition; nature based solutions; advancing equitable livelihoods, decent work and empowered communities; and building resilience to vulnerabilities, shocks and stresses. He highlighted the work FAO is doing across the region linked to food systems transformation and reinforced that success requires enhanced national capacity building, investment, science based solutions; and strong governance along the food supply chain and regional cooperation and integration. The importance of the Food Systems Countdown Initiative framework and monitoring system in leading the way forward to track progress, guide decision-makers, and inform transformation was stressed and welcomed.

Presentation of Themes
The themes were introduced by members of the Initiative.

Session 1 on Diet, Nutrition and Health Domain was presented by Stella Nordhagen. In this theme people are at the center, and it discusses several indicators related to diet, nutrition and health by looking at the

various areas which measure quality of diet, including nutrient adequacy and dietary risk factors for NCDs. As well as measuring the other factors affecting diet quality, namely the accessibility to healthy diets (food security); and a focus on on the food environment (availability, affordability, messaging, and food & vendor properties). Finally, examining whether policies contribute positively or negatively towards food availability, food access, and product properties (policies affecting food environments).

Session 2 on Environment and climate Domain was presented by Rosaline Remans. This theme focused on the relation between food systems and the environment. The indicators focused on the main environmental systems and processes which interact with food systems: land use, climate, water use, biosphere integrity, and pollution (e.g., biogeochemical flows/novel entities). The indicators cover components and processes providing essential environmental services for the environment and humanity. As well as focusing on the areas where food systems can achieve the necessary change.

Session 3 on Livelihoods, poverty and equity Domain was presented by Alejandro Guarin. The indicators for this theme monitor the transformation created by food systems for the numerous people who work as part of the food system, in rural and urban areas, and in high and low-income countries. It focused on the areas of poverty and income, employment, social protection and rights. Cross-cutting issues were also pointed out: governance and resilience and sustainability.

Session 4 on Governance was presented by Sheryl L. Hendriks. The Governance Theme looks at how governance of the food systems domain can foster alignment and coherence across different food system actors, their activities, and progress toward results. It aims to monitor the shared vision of the outcomes, the relevant policy instruments to align efforts, the implementation of resources, and accountability for the outcomes.
Session 5 on Resilience and sustainability was presented by Paulina Bizzotto Molina. The theme of Resilience and sustainability was presented as a transversal aspect of the transformation of agri-food systems with a multi-dimensional interpretation. It covered the domains of exposure to shocks, resilience capacities, agrobiodiversity, food security stability, food system sustainability index. Resilience and sustainability are critical for food and nutritional security. They are also critical for other functions such as being a precondition for sustainability, and critical to tackling poverty and livelihoods issues.

Discussion
Methodology
Discussions took place in plenary, under five sessions covering the themes of the indicator framework, namely: Session 1: Diets, Nutrition and Health; Session 2: Environment and climate Domain; Session 3: Livelihoods, poverty and equity Domain; Session 4: Governance Domain; and Session 5: Resilience and sustainability

In each session, the discussion was motivated by a presentation on the proposed set of indicators. The discussions focused on the capacity of the proposed indicators to guide policy decisions and promote accountability mechanisms. In this sense, the focus is on the relevance, usefulness, and validity of the proposed set of indicators from a regional perspective.

This was followed by discussions based on the three guiding questions listed below.

Do you consider the proposed indicators **(this question examines the indicators suggested by the Initiative):**
Relevant, defined as their ability to measure something meaningful for food systems across a variety of settings, during relevant time periods?
High quality, defined as using the best and most rigorous statistical methodologies and data available?

Interpretable defined as having the ability to show a clear desirable direction of change, comparable across time and space, and easily communicated.
Useful, defined as its ability to be used for policy and decision-making processes and by meeting actual information needs.

What are the data gaps that you can identify? **(this question examines data issues).** Are these gaps structural? If not, are there some data or indicators that you know are available in your region and can be used for the purpose of this monitoring and assessment system?

What are the regional aspects we need to take into consideration at the moment of selecting a set of indicators to monitor the state of food systems and its evolution? **(this question identifies any new indicators needed to cover relevant issues)**

Discussion points relevant to all Domains
This section discusses points which are cross-cutting across all Domains.

The indicator sets are presented in silos. However, it was noted that there are links between Domains which are not reflected. Particularly for Diets, Nutrition and Health; Livelihoods, Poverty and Equity; and also for Environment. The details are discussed under each Domain.

It was also highlighted, that for several issues relevant to the region, disaggregation of indicators is needed: by gender, urban/rural location, and youth. In addition, sub-national reporting of internally displaced persons (IDPs) and refugees is needed as these groups are of particular importance to food systems monitoring in the region.

Participants emphasized the lack of data availability in the region. In particular, the region lacks recent data for many of the indicators included in the catalogue. Even where data is available, this is often out of date or lacks a complete time series. Which makes monitoring and examining trends problematic. Dependence on global data may not be very useful as they do not capture country/ regional specificities.

Participants raised concerns about the cost of such data collection as well as the local capacity to do data collection/ analysis/ reporting. Despite numerous capacity building initiatives held in the region, data collection remains an institutional as well as a political problem. These problems need to be addressed to facilitate availability of data from this region. Capacity building alone is not sufficient to ensure data availability.

Key issues which are important to the region, and cut across all domains were water, trade, food import dependency, conflict and refugees/IDPs. These were not well reflected in the set of indicators.
It was also noted that indicators of agricultural production and productivity were not explicitly mentioned in the indicator set, despite being a core element of food systems transformation. Such indicators should be included as it is important from the point of view of availability and affordability of nutritious food at national level in the context of high import dependency.

4.1 Diets and nutrition

Do you consider the proposed indicators relevant, high quality, interpretable and useful?
Overall, indicators selected for the theme are relevant and they are of high quality depending on the source they are coming from. However, in some cases data interpretation may be difficult. It will be important to think about the ways of communicating the indicators to non-experts on the theme, as some of these indicators are not easy to interpret. For example, the indicator on egg consumption is not very clear – e.g. consumption of how many eggs is considered to be good for (or detrimental to) health.

Participants have raised a number of other questions such as the number of indicators included in the catalogue; the ways of selecting a limited number of indicators to convey clear messages to policy makers; and how to construct a clear dashboard with fewer indicators which clearly indicates those which need improvement.

The issue of the baseline information for monitoring was mentioned. Participants suggested to use internationally sourced data for the baseline as national data might not always be available. The question was raised whether countries would be ready to share national data for reporting.

Participants have inquired whether the countries would need to collect these data. They have expressed their concerns about the cost of such data collection as well as the local capacity to do data collection/ analysis/ reporting.

There was a question regarding a geographic level of reporting on the indicators – whether this should be done at global, national and/or regional levels. The selection of indicators should ensure that they are useful for national and regional policy making. Sub-national data is of particular importance for the countries in conflict as national data may not cover the issues of internally displaced persons (IDPs) and refugees.

Participants pointed out that the proposed indicators were biased towards high-income countries, where more data was available compared to low-income countries. The data situation is also different across countries of the region.

Specific Recommendations

Indicators must capture regional specificities: sub-national and disaggregated indicators should be included to capture specific issues related to the region such as the situation of refugees and IDPs. Not only are these important for national policy making, but disaggregated data would also be useful for FAO-RNE programming.

Number of indicators should be limited: this will be helpful for better understanding and interpretation by policymakers and non-experts. It is also important to flag the indicators that need to be addressed urgently, which is crucial for policy guidance.

Developing a data manual is important: the manual will document the details of data requirements for the monitoring framework, definitions of proxy indicators used, methods of data computation, and a guide on the interpretation of the results and relevance to policies.

What are the data gaps that you can identify? Are these gaps structural? If not, are there some data or indicators that you know are available in your region and can be used for the purpose of this monitoring and assessment system?

Participants emphasized the data availability issue in the region. In particular, the region lacks up to date data for many of the indicators included in the catalogue (this applies to all themes discussed during the meeting). Due to existing gaps in the data series, monitoring and examining trends of different indicators will be problematic. Dependence on global data may not be very useful as they do not capture country/ regional specificities.

There are also issues with timeliness and completeness of the data series, which are important data quality characteristics and are required for interpreting the change. For example, UNICEF has flagged the issues about the availability, quality and timeliness of data related to children's diet quality (6-23 months). In most cases, countries may collect data for the Minimum Dietary Diversity (MDD) indicator, but less so

for other diet-related indicators. It also depends if you use national surveys or other surveys (e.g. SMART surveys) for this information. There are also challenges with the existence of relevant indicators around the food environment and related policies across countries.

Participants mentioned the issues related to data interpretability and comparability among different national data sources. For example, data on healthy diets is collected by different entities and organizations inside the same country and data comparability is a systemic issue. A consortium from all the concerned parties or any other form of cooperation is needed to collect data on the same theme.

Participants have pointed out that data availability problems were endemic and long term. Despite numerous capacity building initiatives held in the region, data collection remains an institutional as well as a political problem. The statistics institutions lack resources to collect data on a regular basis as well as the mandate to disseminate data. These problems need to be addressed to facilitate availability of data from this region. Capacity building alone is not sufficient to ensure data availability.

Specific Recommendations

When selecting indicators for monitoring, it is important to consider not only data availability, but also timeliness of data. Even if some data is available it is likely to be out of date. There are also many gaps in the data series so considering completeness of the time series is important.

What are the regional aspects we need to take into consideration at the moment of selecting a set of indicators to monitor the state of food systems and its evolution?
There is a need for indicators to capture the situation of countries affected by conflicts, considering that conflict is one of the major driving factors of food insecurity and malnutrition in the region. Namely, conflicts cause disruptions to livelihoods (loss of assets and incomes), food production (crop and livestock) and food systems, which affect affordability and accessibility of nutritious foods for healthy diets. Conflicts also affect access to clean water, which is key to ensure food safety and food utilization. It is also crucial to think about the rapid changes and implications caused by conflicts and their impact on the extent towards which data can depict the current situation.

The situation of IDPs and refugees from countries affected by conflicts, including the countries of the region that host refugees (e.g., Lebanon, Jordan) needs to be captured. National data do not always depict situation of these population groups.

Participants reemphasized throughout the discussion the region's dependence on food imports and the need to incorporate indicators of food trade into the catalogue. It is important to distinguish between imported and locally (country or region) produced food and agriculture products.

A binary indicator whether the country has any tax policy levied on foods for health reasons may not be very efficient since there might be several such policies for different food products. Currently, the catalogue of indicators does not make differentiation between having one policy or several policies.

The Economic Commission for Western Asia and Africa (ESCWA) has suggested to examine the Arab Food Security Monitoring Framework (developed jointly by ESCWA, FAO and AOAD[10]) for indicators that potentially could be included in the indicator catalogue for monitoring food systems transformation. https://www.unescwa.org/publications/tracking-food-security-arab-region.

Specific recommendations for additional indicators

[10] https://www.unescwa.org/sites/default/files/pubs/pdf/manual-monitoring-food-security-arab-region-english_1.pdf

Import dependency ratio was suggested to be included in the indicator catalogue. This indicator would reflect food systems transformation as well as development of the agriculture sector. This would help to understand their impacts on the costs and affordability of different food products. It will be important to highlight import dependency on strategic food products such as wheat, oils and sugar among others; monitoring of imports of unhealthy food items (highly processed food, sugar-sweetened beverages, etc.) is also needed.

Indicators of agricultural production and productivity related to specific or strategic crops and animal products relevant to food habits in the region should be included. This is important from the point of view of availability and affordability of nutritious food at national level in the context of high import dependency.

Indicators measuring the efficiency of marketing infrastructure in the countries and percentage of food loss of agriculture products (wheat, fruits and vegetables, animal products) need to be included.

A logistics indicator such as the food cooling indicator can be used to assess food quality and availability.

A number of enacted legislations/policies promoting/limiting the import of unhealthy food items would be more useful indicator rather than a binary indicator of having a policy to tax foods for health reasons.

4.2 Environment and climate domain

Do you consider the proposed indicators relevant, high quality, interpretable and useful?
Participants expressed concerns about the growing number of indicators being developed. It was suggested that there should be some consideration to reduce the number of indicators, and to select those which have a broader relevance and are applicable to multiple country and regional contexts. Some highlighted the need for indicators which are applicable across different countries. One proposal for a more compact set of indicators was for the creation of a matrix to indicate an indicator's relevance to multiple processes and states, including soil health, agricultural productivity, or consumption. Other participants highlighted the need to build capacity in countries, as well as to account for the cost of collecting data related to environment and climate.

What are the data gaps that you can identify? Are these gaps structural? If not, are there some data or indicators that you know are available in your region and can be used for the purpose of this monitoring and assessment system?
Concerns were raised about the complexity of measuring GHG emissions, biodiversity loss, land encroachment, and soil carbon. This challenge was noted during the preparation of the upcoming report on the *State of Land and Water Resources SOLAW in the Near East and North Africa.* Remote sensing is a good tool for monitoring and data collection, but there is a need to ensure that the results produced are triangulated with realities on the ground. In order to better support informed decision making, the methodologies used should be robust.

Participants highlighted that one aspect which is missing from the set of environment and climate indicators is the negative impacts of encroachment on agricultural land by the expansion of urban and industrial uses. The expansion of these uses are reducing the area available for crop production and livestock. The threat of encroachment was highlighted by representatives of at least three countries, including Egypt and Libya. In one case (Egypt), data on this indicator was available.

Regarding the indicator on pesticides, participants suggested adding an indicator on countries' uptake of biological fertilizers containing bacteria, fungi and microorganisms. Participants agreed there could be the potential also to capture the transition to sustainable agricultural practices. Furthermore, in terms of soil

biodiversity, there is an indicator called ‘soil biodiversity potential’ which is included in a global atlas, but there are currently no plans to update this. Instead, data on changes in soil organic carbon is available. Another participant noted that soil data collection is ongoing in several countries within the context of a FAO-Technical Cooperation Project, and maps of soil properties and soil organic carbon are expected to be produced by the end of the year.

Water is critical to the region and the production of cash crops has implications on water use. Currently there is only one indicator for water. One suggestion was for an indicator on virtual water, measuring the water use through exports and imports of agricultural and other products, that may be helpful in understanding the transition to a sustainable food system in water-scarce contexts.

What are the regional aspects we need to take into consideration at the moment of selecting a set of indicators to monitor the state of food systems and its evolution?
Participants highlighted the links between the environment/climate and food security themes for the region. For example, the ongoing conflict in Ukraine poses a threat to global and regional food security, highlighting the vulnerability of countries and food systems to such threats.

While participants were reminded that the indicators came from already-established secondary sources and datasets, several participants drew attention to relevant indicators that already exist in countries, and encouraged the narrative developed to include also these indicators. This could assist countries in identifying gaps and investment needs.

There was a need for an evaluation of the results and lessons learned from past programmes, as well as achievements so far, to better understand the regional context for food systems monitoring.

4.3 Livelihoods, poverty and equity Domain

Do you consider the proposed indicators relevant, high quality, interpretable and useful?
The indicators on livelihoods, poverty and equality the indicators are generally relevant but some points were raised regarding their quality and interpretability.

One drawback is that several of the proposed indicators cannot be measured for the specific population that is engaged in the agrifood system, but only for the entire national population. Without this specificity, the usefulness of indicators, such as national rates of unemployment, rate of informality or the percent of the population earning low pay is limited in terms of assessing progress in agrifood system transformation. As they reflect the general population, any increase or decrease in these indicators cannot be attributed to changes within the agrifood sector.

Another key issue is that disaggregation by gender and space, are key elements of equality in the region, and are thus needed to adequately assess the theme. Gender gaps and geospatial gaps can only be reflected if the data is disaggregated by sex and rural versus urban geography. Although this data is not readily available, ignoring disaggregation would be detrimental to the quality and usefulness of the proposed indicators. Age and migrant status are two additional disaggregations that could be important when assessing equality.

In terms of interpretability, the indicators of households with significant income from agriculture and coverage of any social protection programme presented difficulties. It is not immediately clear if an increase in the share of households with significant income from agriculture would be considered a positive or negative outcome. Such a conclusion would depend on the context and drivers of this change. Moreover, off-farm activities including post-harvest handling, aggregation, processing and distribution are often key additional sources of income in rural areas that should not be ignored.

Similarly, social protection coverage could mean different things in different contexts. For example, an increase in social protection coverage could be positive if the number of those in poverty has not changed and if the programmes are adequately targeting those in need. Indicators could be added regarding the availability, efficiency and coverage of social safety nets, and the availability of official social solidarity systems that encompass dimensions related to food security such as food subsidies.

Questions were raised on the usefulness of the indicator of population earning low pay, and whether this is an adequate measure of poverty. Poverty headcount is included in the indictors under resilience and may be better placed under livelihoods. Another indicator for poverty is the Multi-dimensional Poverty Index which is also disaggregated by rural and urban for select Arab countries.

What are the data gaps that you can identify? Are these gaps structural? If not, are there some data or indicators that you know are available in your region and can be used for the purpose of this monitoring and assessment system?

Data on the distribution of land holdings is collected through the agriculture census in Egypt as in many countries. This census is conducted every 10 years on average meaning the data would not allow for bi-annual monitoring. To address the issue of data availability more broadly, one suggestion is to also examine other sources of data such as those generated by the private sector.

Informal employment, especially in agriculture will be difficult to measure without a specific study in each country. Most of the agriculture in the region is family agriculture with family labour that is unpaid. This indicator may not fit the rural reality in the region.

In terms of methodology, the indicators need to have clear definitions of poverty, informal employment and significant income as well as indicate clearly how national discrepancies in these definitions are overcome.

What are the regional aspects we need to take into consideration at the moment of selecting a set of indicators to monitor the state of food systems and its evolution?

There are very high levels of gender inequality in the NENA region which affect agrifood system development. While the indicator on female ownership of landholdings attempts to reflect gender disparities, this issue is insufficiently captured by the proposed indicators. The share of women in agriculture through paid or unpaid work, inclusion in rural institutions, exposure to gender based violence, discriminatory laws and practices are critical elements that should be included to the extent possible.

The NENA region has the highest youth unemployment rates in the world. Unemployment for young women is significantly higher than young men, reaching 40%. This concern would only be reflected if the unemployment indicator was disaggregated by sex and age. The region is also witnessing an unprecedented level of youth in the population where, in several NENA countries, more than half the population is below the age of 25. At the same time young people are less engaged in agriculture production and are often migrating out of rural areas to escape poverty.

A closely related issue is that employment in rural areas is often higher than in urban areas but the quality and decency of work is lower due to its informal and physical nature. The low wages, unhealthy and unsafe working conditions, and irregularity are among the reasons that young people are reluctant to work in agriculture. Possible indicators which can reflect not only the quantity of jobs but their quality include: work related injury in agriculture sector, prevalence of unpaid work, applicability of labour laws to the agriculture sector (including minimum wage).

Another critical aspect to be taken into consideration is the refugee crisis faced by so many NENA countries. Forced migrants tend to settle in urban areas where services are more available, however a

significant share of refugees and IDPs (up to 40%) end up in rural areas where they work in food production and processing. These workers are especially vulnerable to abuse and exploitation. An indicator on Share of migrants in the agriculture workforce, could help indicate the presence of vulnerable labour. In addition, international and rural migration for economic reasons may also have an important bearing on agrifood systems through remittances and labour movement, although data looking at the intersection of migration and agrifood sectors is limited.

The discussion highlighted a number of assets and resources that influence livelihood, poverty and equality. Principle among these are access to financial services, access to land and water (of particular importance in the NENA) and access to digital technologies. The digital divide and asymmetry in the use of digital tools can in many cases exacerbate inequalities across the agrifood sector. However, digital technologies can also be leveraged to close gaps. For example mobile payments can be especially empowering for female producers who face restrictions on their mobility. Smartphone penetration is included under the indicators for resilience, but can fall under the livelihoods and poverty domain as well along with indicators like internet penetration and computer use. It is important to capture how digital technologies are transforming the sector and to whose benefit.

Finally, the indicators focus mainly on the agricultural producers, but for the region a focus on the consumers (which reflect the consumption and demand side) is important for poverty. To track livelihoods of consumers an indicator on price volatility could be useful as it affects consumer purchasing power and many trends can be covered with this one indicator. In addition, agricultural productivity impacts resilience, livelihoods and environmental sustainability yet is not included as an indicator in any of the domains.

4.4 Governance Domain

Do you consider the proposed indicators relevant, high quality, interpretable and useful?
It was noted that food systems are complex and delivers on a range of outcomes from nutrition, environmental to socio economic. Therefore, in order to capture governance it may be more useful to focus on one major outcome rather than looking at the food system as a whole, as that may not be manageable through broad indicators.

In terms of interpretability, it was noted that many of the indicators proposed are composite indicators. The point was made that these will need to be explained and unpacked to policy makers to make the governance aspect of the framework useful for them.

Regarding the proposed indicators, discussion reflected the view that these provide only a limited view of governance and indicators reflecting quality of food systems governance is also needed. For instance, Policy Coordination can be measured looking at existence of multi stakeholder platforms but the quality in terms of inclusivity and linkage to centres of decision making is also important. Some of the indicators were too specific while some others were too broad due to composite indices. The narrow or specific indicators may need to be reviewed as these may not be applicable universally, at least in the context of data and reporting.

The need to consider quality was also highlighted for the indicator titled "Presence of a national food systems pathways". The development of a pathway is just a start, and it should reflect the quality of the pathway, with complementary indicators which measure 1)whether there is an associated investment or resource mobilization plan (key to its success in implementation), 2) is it sufficiently gender and age responsive and 3) does it have clear and measurable targets. The power of the food systems pathways for decision makers could also be indicated by the presence of communication or dialogue mechanisms.

Discussion was raised around specific indicators, namely:

The point was made that many of the governance indicators are not specific to food systems, while the theme should focus more directly on food systems governance than trying to monitor governance in general. The point applies to the indicators: Civil Society Index, Varieties of Democracy; VDEM Accountability Index, and Voice and Accountability, WGI. The role of an international food systems framework is to measure food systems transformation rather than wider governance transformation, and in addition, the wording of the indicators in terms of accountability of governance does not reflect the ethos of international frameworks. A suggestion was made that for the Civil Society Index a more useful indicator would be a measure of public engagement and would allow measurement of the voices of the ordinary public in food systems transformations.

The indicator on Government Effectiveness describes it as including " the degree of independence of the civil service from political pressures". Clarification is needed on the interpretation of this indicator. The civil service develops and implement policies required by government. Its role is also to advise, not to decide policy which is the prerogative of the Minister, therefore by its nature, it is not independent from politics.

Participants noted that the indicator on implementation of marketing of breast-milk substitutes restrictions may be more relevant to Theme 1 and also qualifies as too specific/narrow for the governance theme. In addition, for the NENA region it may be more of social than governance issue, as was the case in countries which faced resistance from industry to legislative change.

What are the data gaps that you can identify? Are these gaps structural? If not, are there some data or indicators that you know are available in your region and can be used for the purpose of this monitoring and assessment system?
The indicators are drawn from non-national sources. However, the general issues of data gaps also applies to the governance theme. This theme has a particular requirement for timely and accurate data which may not be available, at least in many of the NENA regional countries

What are the regional aspects we need to take into consideration at the moment of selecting a set of indicators to monitor the state of food systems and its evolution?
Relevant governance indicators can be found under SDG Goal 16 "Promote peaceful and inclusive societies for sustainable development, provide access to justice for all and build effective, accountable and inclusive institutions at all levels". In particular, Target 16.6: Develop effective, accountable and transparent institutions at all levels with Indicator 16.6.1: Primary government expenditures as a proportion of original approved budget, by sector (or by budget codes or similar) and Indicator 16.6.2: Proportion of population satisfied with their last experience of public services

In the region conflict is an issue affecting governance of food systems as it weakens the structure and increases informality.

Another issue which is important for food systems governance is asymmetry of power and power dynamics. Some area of interest are the driving power of cooperate and retail actors and their level of influence on food systems; and the conflict of interest for the transformation of food systems. It was suggested that levels of private and public sector partnership might be useful to monitor

There are many indicators by city region networks, for example, the Milan Networks. It may be useful to examine city-region governance networks as urban food governance also provide suggestions for indicators at national level. An indicator is needed which covers the existence of urban food system policies/plans/strategies, and a possible source is the Milan Food Policy site and the City Region Networks

Examination of the transition from centralized to decentralized governance is a useful aspect of food systems transformation. For example, the presence of municipal food system policies/plans/strategies. However, it was noted that this would have to be adjusted for countries where there is no strong urban system.

Another suggested indicator is whether a country has implemented the Voluntary Guidelines on Food Security and Nutrition; it was suggested that relevant information is available from the Committee on Food Security.

4.5 Resilience and sustainability Domain

Do you consider the proposed indicators relevant, high quality, interpretable and useful?
A number of the indicators this domain are also relevant to the Diets, Nutrition and Health, and Livelihoods, Poverty and Equity, but also to the Environment domain. It was suggested that Resilience could focus on indicators which reduce the impact of shocks and help systems to better sustain or recover from those shocks.

It was also noted that composite indexes such as the Dietary Sourcing Flexibility Index and the Global Innovation Index can be difficult to interpret by policy makers, and can hide issues revealed by their individual components. It would also be useful to highlight the causal pathways between specific interventions and sustainability outcomes.

Discussion focused on specific indicators, namely:
Regarding the Stability of Food Insecurity Experience Scale (FIES) - based indicators and Stability of Prevalence of Undernourishment, stability is not the direction of interest. A stable POU and FIES indicator can be high, but to show improvement the trend should be decreasing – not stable.

The relevance of mobile cellular subscriptions (per 100 people) and renewable electricity output (% of total electricity output) for policy makers was not clear.

It was also questioned whether road density and distance to food are both needed as they provide a similar measure.

Specific Recommendations
Resilience should focus on resilience and capacities according to its definition, as well as presence of instruments and tools used to reduce the impact of or recover from shocks, such as the different insurance tools.
The number of resilience indicators can be more limited, with triage led by examination of use from the policy perspective, giving priority to those that are simple, easy to understand and can convey key messages to policymakers.
Comprehension of averaged composite indexes is difficult for policy makers; and an unpacking of the sub-indicators within the composite index would be useful.

What are the data gaps that you can identify? Are these gaps structural? If not, are there some data or indicators that you know are available in your region and can be used for the purpose of this monitoring and assessment system?
The issues discussed in Diets, Nutrition and Health are also relevant to this theme.

It was mentioned that the data on economic impact of disasters in the region is incomplete and could lead to underestimation of the impact.

What are the regional aspects we need to take into consideration at the moment of selecting a set of indicators to monitor the state of food systems and its evolution?
The discussion opened highlighted the high vulnerability of food systems in a region, particularly as there is a high dependence on imports for the majority of food staples. Vulnerability was seen as being determined by three generic characteristics: (1) the wealth available in the system, (2) how connected is the system, and (3) how much diversity exists in the system.

It was also suggested that consideration be given to food system vulnerability to future shocks (not only current shocks) is important and strongly related to food system resilience.

This is also linked to the need to balance efficiency and resilience (which is is one of the largest trade-offs in food systems). For example, vulnerability increases with the interconnectedness of food systems and trade patterns. Importing countries which rely on a single source have a lower diversity in terms of trade partners. This increases the vulnerability and exposure to future shocks.

A second issue relevant to the resilience of the region was that of economic migration and refugees as a coping strategy.

Specific Recommendations
It is important to include indicators that link issues of trade and migration as a coping mechanism to resilience of food systems.
Indicators on how to build resilience that consider both domestic production and transportation flows between countries within the region.
Diversity of the food systems in the region and the extent of concentration of imports of major food and agriculture products, such as wheat, should be considered in the indicators used.
Indicators must assess the vulnerability of the food systems in the region to withstand future shocks. For example, what are the commodities imported for major food staples, and the level of vulnerability. There has also been debate on future shocks around the FSTI dietary sources index.
Migration can be considered as a coping strategy in the región. For example, relevant indicators are remittances and how they are used in consumption and investment.

Annex 1: Agenda of meeting

Wednesday 18 May 2022
Cairo time

**Time**	**Subject**	**Speaker**
		Master of Ceremonies, Jean Marc Faurés, FAO
10:00-10:30	Welcoming remarks and introduction to the Initiative	*RNE ADG*<br>*José Rosero Moncayo, FAO*<br>*Kate Schneider, the Initiative*
10:30-11:45	Session 1: Diet, Nutrition and Health Domain<br>*Motivational presentation*<br>*Moderated discussion*<br>*Summary*	 *Stella Nordhagen, the Initiative*<br>*Moderator: Tamara Nanitashvili, FAO*
11:45-12:00	Break	
12:00-13:15	Session 2: Environment and climate Domain<br>*Motivational presentation*<br>*Moderated discussion*<br>*Summary*	 *Roseline Remans, the Initiative*<br>*Moderator: Theresa Wong, FAO*
13:15-14:30	Session 3: Livelihoods, poverty and equity Domain<br>*Motivational presentation*<br>*Moderated discussion*<br>*Summary*	 *Alejandro Guarin, the Initiative*<br>*Moderator: Dalia Abulfotuh, FAO*
14:30-14:45	Break	
14:45-16:00	Session 4: Governance Domain<br>*Motivational presentation*<br>*Moderated discussion*<br>*Summary*	 *Sheryl L. Hendriks, the Initiative*<br>*Moderator: Ahmad Mukhtar, FAO*
16:00-17:15	Session 5: Resilience and sustainability<br>*Motivational presentation*<br>*Moderated discussion*<br>*Summary*	 *Paulina Bizzotto Molina, the Initiative*<br>*Moderator: Ahmad Mukhtar, FAO*
17:15-17:30	Wrap up and Conclusions	*Jean Marc Faurés*<br>*Kate Schneider, the Initiative*

Participants List

<u>Invited Experts:</u>

AHL ZINE Brahim
Chef Division
Le Haut Commissariat au Plan HCP
Morocco

AITKADI Mohamed
President
General Council of Agricultural Development
Morocco

ALAJMI Asma
Senior Economic Analyst
Public Authority of Food & Nutrition
Kuwait

AL DOMOR Lamia
Manager
Ministry of Agriculture
Jordan

ALESTAD Jeehan
First Permanent Representation of Kuwait to Food and Agriculture Organization of the United Nations FAO & World Food Programme WFP Secretary
Kuwait

ALFRIHAT Nada
Head of Organizations Division
Ministry of Agriculture
Jordan

ALHAMMAD Mohammad
Food Quality Regulations Manager
The Saudi Grains Organization SAGO
Saudi Arabia

AL JAILANI Fatma
Head Section of Studies and Research
Ministry of Agriculture, Fisheries Wealth and Water Resources
Oman

ALKOSTABAN Khaled
Agriculture Value Chain Officer
Food and Agriculture Organization of the United Nations FAO
Yemen

ALLAN Mohamed
Deputy Minister
Ministry Agriculture Irrigation and Fishing Wealth
Yemen

AL-OTHAMN Wajd
Food Security Acting Director
Public Authority for Food and Nutrition
Kuwait

ALRAWAHIA Nargis
Head of Sectors Accounts Department
National Centre of Statistics and Information (NCSI)
Oman

AL SAADI Anwar
Director of Statistics and Planning
Ministry of Fish Wealth
Yemen

ALSABAH Manar
Attaché
Permanent Representation of Kuwait to Food and Agriculture Organization of the United Nations FAO & World Food Programme WFP
Kuwait

ALSULAIMAN Mohammed
Consultant
Ministry of Environment, Water and Agriculture MEWA
Saudi Arabia

ALWAN Abdulla
Chairman
Ministry of Agriculture, Irrigation and Fisheries
Yemen

BAKAR Lutfi
Expert
Libya

BALUSHI Sultan
Head of Consumer Price Statistics Section
National Centre for Statistical Information NCSI

Oman

BOUCHAMA Khalid
Conseiller
Conseil Général du Développement Agricole
Morocco

DAKHAKHNI Sumaiya
Engineer
Ministry of Agriculture
Libya

EID Mariam
Head of Agro Industries Department
Ministry of Agriculture
Lebanon

ELMEZDAWI Walid Khalid Omar
Representative of the International Cooperation Office
Ministry of Agriculture and Livestock
Libya

FADLAOUI Aziz
Chercheur
Institut National de la Recherche Agronomique
Morocco

FARESS Yahya
Chef de Service
Département de l'Agriculture
Morocco

HATTER Raed
Deputy General Manager
Arab Organization for Agricultural Development
Egypt AOAD
Sudan

HOBIESHAN Ahmed Saeed
Manager of Coffee Department
Ministry of Agriculture and Irrigation & Fish Wealth
Yemen

KAMOUKA Sadeg
Chairman
National Committee for Desert Locust Control
Libya

KHAMIS Shokry
General Manager
Ministry of Agriculture
Yemen

KORDASS Hassan
Chargé de Mission
Conseil Général du Développement Agricole (CGDA)
Morocco

MAAKOUL Siham
Département de l'Agriculture
Chargée de Dossiers de Coopération Multilatérale
Morocco

MANSOURI Amal
Economist
Le Haut Commissariat au Plan HCP
Morocco

MHANNA Maya
Head of Rural Engineering Service
Ministry of Agriculture
Lebanon

QTANANNI Iqbal
Head of Studies of Value Chain Department
Ministry of Agriculture MOA
Jordan

RASHID Hanaa
Director
General Authority for Marine and Aquatic Sciences Research
Yemen

RIZK Reda
PGR Expert
Biodiversity expert
Egypt

SALIBI Amal
Head of Economic Studies and Statistics Service
Lebanese Ministry of Agriculture
Lebanon

TALY Ahmed
Assistant of General Director
Ministry of Agriculture MOA
Iraq

YASSIN Dalia
Prof. Economist,
Director of Food Loss and Waste
Monitoring and Evaluation Unit
Agriculture Research Center,
Agriculture Economic Research Institute Egypt

ZAROUALI Said
Le Haut Commissariat au Plan HCP
Morocco

International and Regional Agencies and Other Institutions:

ABD-ALLAH Eman
Expert
Agricultural Economics
Arab Organization for Agricultural Development
AOAD
Egypt

ABDELGADIR Mohamed
Country Director
International Fund for Agricultural Development
IFAD
Egypt

ABOUARAB Julie
United Nations Economic and Social
Commission for Western Asia UN ESCWA
Associate Coordination Officer
Lebanon

AL-QUDSI Khalid
Regional Programme Advisor
World Food Programme WFP
Egypt

AL-SAMAWI Ahmed
Agricultural Expert
Arab Organization for Agricultural Development
AOAD
Sudan

BARIGOU Sabah
Regional Head
Nutrition, HIV and School Based Programmes
World Food Programme
Egypt

BERTINI Raffaele
Data and Statistics Officer
International Organization for Migration
IOM
Egypt

FAISAL Walaa
Rural Women Development Expert
Arab Organization for Agricultural Development
AOAD
Sudan

GARG Aashima
Senior Nutrition Specialist
United Nations Children's Fund UNICEF
Middle East and North Africa Regional Office
Jordan

GUARIN Alejandro
Researcher
International Institute for Environment and
Development IIED
United Kingdom of Great Britain and Northern
Ireland (the)

HENDRIKS Sheryl
Professor
University of Pretoria
South Africa

MADANI Wigdan
United Nations Children's Fund UNICEF
Nutrition specialist
Jordan

MOLINA Paulina Bizzotto
Policy Officer
European Centre for Development Policy
Management (ECDPM)
Netherlands

NEJDAWI Reem
Chief of Food and Environment Section
United Nations Economic and Social
Commission for Western Asia UN ESCWA
Lebanon

NORDHAGEN Stella
Senior Technical Specialist
Global Alliance for Improved Nutrition
Switzerland

OUMID Bedreddine
Directeur Régional
L'Organisation Arabe du Développement de l'Agriculture OADA
Algeria

RAWABDEH Fida'a Ali
Head of Eastern Region Office
Arab Organization for Agriculture Development AOAD
Jordan

REMANS Roseline
Visiting Researcher
Alliance of Biodiversity and International Centre for Tropical Agriculture CIAT, CGIAR
Switzerland

SCHNEIDER Kate
Researcher
Johns Hopkins University
United States of America (the)

<u>FAO ESS</u>

ROSERO MONCAYO José
Director
Food and Agriculture Organization of the United Nations, Statistics Division, FAO-ESS
Rome, Italy

MUNOZ Hernan Daniel
Statistician
Food and Agriculture Organization of the United Nations, Statistics Division, FAO-ESS
Rome, Italy

<u>FAO RNE</u>

ELWAER Abdulhakim
Assistant Director General
Regional Representative
Food and Agriculture Organization of the United Nations FAO Regional Office for the Near East and North Africa
Cairo, Egypt

FAURÈS JeanMarc
Regional Programme Leader
Food and Agriculture Organization of the United Nations FAO Regional Office for the Near East and North Africa
Cairo, Egypt

ABDELMAGEED Samar
Data Analyst
Food and Agriculture Organization of the United Nations FAO Regional Office for the Near East and North Africa
Cairo, Egypt

ABULFOTUH Dalia
Economist
Food and Agriculture Organization of the United Nations FAO Regional Office for the Near East and North Africa
Cairo, Egypt

AHMED Mohamed
Policy Officer
Food and Agriculture Organization of the United Nations FAO Regional Office for the Near East and North Africa
Cairo, Egypt

ALAOUI Hicham El Mhamdi El
Monitoring and Evaluation M&E expert
Food and Agriculture Organization of the United Nations FAO Regional Office for the Near East and North Africa
Cairo, Egypt

AMIN Karim
Innovation Consultant
Food and Agriculture Organization of the United Nations FAO Regional Office for the Near East and North Africa
Cairo, Egypt

CHIN Nancy
Regional Statistician
Food and Agriculture Organization of the United Nations FAO Regional Office for the Near East and North Africa
Cairo, Egypt

FETSI Theodora
Consultant

Food and Agriculture Organization of the United Nations FAO Regional Office for the Near East and North Africa
Cairo, Egypt

MUKHTAR Ahmad
Senior Economist
Food and Agriculture Organization of the United Nations FAO Regional Office for the Near East and North Africa
Cairo, Egypt

NANITASHVILI Tamara
Nutrition and Food Systems Officer
Food and Agriculture Organization of the United Nations FAO Regional Office for the Near East and North Africa
Cairo, Egypt

REZAEI Maryam
Agroindustry officer
Food and Agriculture Organization of the United Nations FAO Regional Office for the Near East and North Africa
Cairo, Egypt

WONG Theresa
Natural Resources Officer
Food and Agriculture Organization of the United Nations FAO Regional Office for the Near East and North Africa

FAO Iraq

ALI Hamid
Sr. Tech. Dialogues Officer
Food and Agriculture Organization of the United Nations FAO Iraq
Iraq

Fadhil Ismael
National Dialogue Supporter
Food and Agriculture Organization of the United Nations FAO Iraq
Iraq

SHLASH Khalid
Assistant FAO Representative AFAOR (Programme)
Food and Agriculture Organization of the United Nations FAO Iraq
Iraq

FAO Lebanon

SAADE Solange Matta-
Assistant FAO Representative AFAOR (Programme)
Food and Agriculture Organization of the United Nations FAO Lebanon
Lebanon

FAO

AQUILA Dell
Policy advisor for Palestine
Food and Agriculture Organization of the United Nations FAO

Supplementary Information, Appendix 4: Alignment between the Food Systems Countdown Initiative and Related Efforts

Contents

Table 4.1 2030 Agenda for Sustainable Development Global Indicator Alignment to FSCI…………..….2

Table 4.2 Related and other relevant initiatives……………………………………………………..29

Table 4.3 Food System Transformation Pathway characteristics mapped to FSCI architecture and themes……………………………………………………………………………………………44

Table 4.1 2030 Agenda for Sustainable Development Global Indicator Alignment to FSCI

**Goals and targets (from the 2030 Agenda for Sustainable Development)**	**Indicators**	**Related to food systems?**	**Specific to food systems?**	**(How) does it fit into FSCI?**
**Goal 1. End poverty in all its forms everywhere**				
1.1 By 2030, eradicate extreme poverty for all people everywhere, currently measured as people living on less than $1.25 a day	1.1.1 Proportion of the population living below the international poverty line by sex, age, employment status and geographic location (urban/rural)	Yes	No	Lacking data for the rural poverty indicator, we rely on the share of agriculture in GDP as a proxy until the better data become available.
1.2 By 2030, reduce at least by half the proportion of men, women and children of all ages living in poverty in all its dimensions according to national definitions	1.2.1 Proportion of population living below the national poverty line, by sex and age	Yes	No	Not specific to food systems, though poverty and food insecurity are closely related. In service of parsimony, we concentrate on rural poverty for the FSCI indicators.
	1.2.2 Proportion of men, women and children of all ages living in poverty in all its dimensions according to national definitions	Yes	No	

1.3 Implement nationally appropriate social protection systems and measures for all, including floors, and by 2030 achieve substantial coverage of the poor and the vulnerable	1.3.1 Proportion of population covered by social protection floors/systems, by sex, distinguishing children, unemployed persons, older persons, persons with disabilities, pregnant women, newborns, work-injury victims and the poor and the vulnerable	Yes	No	Social protection provides a base degree of social security to vulnerable populations, who are also the most likely to face food insecurity issues or challenges in local food systems. We use coverage and adequacy indicators from the ASPIRE database directly because they are disaggregated into urban and rural, which is more closely related to food systems than the sustainable development goals indicator that is disaggregated by population sub-groups but not geographically.
1.5 By 2030, build the resilience of the poor and those in vulnerable situations and reduce their exposure and vulnerability to climate-related extreme events and other economic, social and environmental shocks and disasters	1.5.2 Direct economic loss attributed to disasters in relation to global gross domestic product (GDP)	Yes	No	[illegible]rely on the EM-DAT dataset [illegible]h has better country and event [illegible]rage than the SDG data that [illegible]e through the Sendai [illegible]ework. However, the EM-DAT [illegible]omic losses dataset is [illegible]mplete relative to its recording [illegible]treme and medium scale [illegible]ster events and is not specific to [illegible] systems. We will replace our [illegible]nt indicator with a new [illegible]ator of agricultural losses due to [illegible]ages when it is released by FAO [illegible]ected late 2023).

**Goal 2. End hunger, achieve food security and improved nutrition and promote sustainable agriculture**				
2.1 By 2030, end hunger and ensure access by all people, in particular the poor and people in vulnerable situations, including infants, to safe, nutritious and sufficient food all year round	2.1.1 Prevalence of undernourishment	Yes	Yes	Included in FSCI
	2.1.2 Prevalence of moderate or severe food insecurity in the population, based on the Food Insecurity Experience Scale (FIES)	Yes	Yes	Included in FSCI
2.2 By 2030, end all forms of malnutrition, including achieving, by 2025, the internationally agreed targets on stunting and wasting in children under 5 years of age, and address the nutritional needs of adolescent girls, pregnant and lactating women and older persons	2.2.1 Prevalence of stunting (height for age <-2 standard deviation from the median of the World Health Organization (WHO) Child Growth Standards) among children under 5 years of age	Yes	No	Stunting is an effect of malnutrition, which is related to the presence of viable food systems. Ultimately not chosen because this has other non-food system causes as well, and this issue is monitored elsewhere.

	2.2.2 Prevalence of malnutrition (weight for height >+2 or <-2 standard deviation from the median of the WHO Child Growth Standards) among children under 5 years of age, by type (wasting and overweight)	Yes	No	Malnutrition is directly related to the presence of viable food systems. Ultimately not chosen because this has other non-food system causes as well, and this issue is monitored elsewhere.
	2.2.3 Prevalence of anaemia in women aged 15 to 49 years, by pregnancy status (percentage)	Yes	No	Anaemia is caused by iron deficiency, which often results from an inadequately nutritious diet. Ultimately not chosen because this has other non-food system causes as well, and this issue is monitored elsewhere.

2.3 By 2030, double the agricultural productivity and incomes of small-scale food producers, in particular women, indigenous peoples, family farmers, pastoralists and fishers, including through secure and equal access to land, other productive resources and inputs, knowledge, financial services, markets and opportunities for value addition and non-farm employment	2.3.1 Volume of production per labour unit by classes of farming/pastoral/forestry enterprise size	Yes	Yes	This indicator measures agricultural productivity, which directly influences the productivity and sustainability of national food systems. However, data beyond agriculture (the rest of food systems) do not presently exist. However, in related work we are developing a new dataset that extends throughout food systems and we expect to incorporate this indicator in the future.

	2.3.2 Average income of small-scale food producers, by sex and indigenous status	Yes	Yes	This indicator helps measure inequalities within key participants in the development and sustainability of national food systems, identifying just how equitable these systems really are. However, it is lacking in data and we acknowledge this as a data gap in the FSCI framework. Data only exist for 49 countries from FAO's RuLIS database. This indicator will be included in future publications if the data become available for a sufficient number of countries across regions and income groups. In the meantime, we use the share of agriculture in GDP as a proxy for rural poverty.
2.4 By 2030, ensure sustainable food production systems and implement resilient agricultural practices that increase productivity and production, that help maintain ecosystems, that strengthen capacity for adaptation to climate change, extreme weather, drought, flooding and other disasters and that progressively improve land and soil quality	2.4.1 Proportion of agricultural area under productive and sustainable agriculture	Yes	Yes	This indicator is relevant to the FSCI framework and will fill one of the identified data gaps. However, this indicator is only just coming into fruition and was not developed when indicators were selected and data remain unavailable. We intend to add this indicator in the future when the data are released.

2.5 By 2020, maintain the genetic diversity of seeds, cultivated plants and farmed and domesticated animals and their related wild species, including through soundly managed and diversified seed and plant banks at the national, regional and international levels, and promote access to and fair and equitable sharing of benefits arising from the utilization of genetic resources and associated traditional knowledge, as internationally agreed	2.5.1 Number of (*a*) plant and (*b*) animal genetic resources for food and agriculture secured in either medium- or long-term conservation facilities	Yes	No	Included in FSCI
	2.5.2 Proportion of local breeds classified as being at risk of extinction	Yes	No	Risk of extinction demonstrates the threats global food diversity faces, which can have a negative impact on the food sources available to regional populations. However, the concept of diversity for food system resilience is well-captured with 2.5.1 and therefore this indicator is not included in service of parsimony.

2.a Increase investment, including through enhanced international cooperation, in rural infrastructure, agricultural research and extension services, technology development and plant and livestock gene banks in order to enhance agricultural productive capacity in developing countries, in particular least developed countries	2.a.1 The agriculture orientation index for government expenditures	Yes	Yes	Budgetary allocation to the whole of food systems, not only agriculture, is an identified data gap of the FSCI that needs to be filled.
	2.a.2 Total official flows (official development assistance plus other official flows) to the agriculture sector	Yes	Yes	The FSCI focuses on indicators relevant to country governments and does not include metrics related to Official Development Assistance.
2.b Correct and prevent trade restrictions and distortions in world agricultural markets, including through the parallel elimination of all forms of agricultural export subsidies and all export measures with equivalent effect, in accordance with the mandate of the Doha Development Round	2.b.1 Agricultural export subsidies	Yes	Yes	Not considered for FSCI as it is not clear how to interpret a desirable direction of change.

2.c Adopt measures to ensure the proper functioning of food commodity markets and their derivatives and facilitate timely access to market information, including on food reserves, in order to help limit extreme food price volatility	2.c.1 Indicator of food price anomalies	Yes	Yes	We chose to use food price volatility because it provides more information than the anomalies indicator that only includes upside spikes.
**Goal 3. Ensure healthy lives and promote well-being for all at all ages**				
3.9 By 2030, substantially reduce the number of deaths and illnesses from hazardous chemicals and air, water and soil pollution and contamination	3.9.2 Mortality rate attributed to unsafe water, unsafe sanitation and lack of hygiene (exposure to unsafe Water, Sanitation and Hygiene for All (WASH) services)	Yes	No	There is a demonstrated relationship between water and nutrition, making understanding access to safe water important for understanding broader nutrition deficiencies in global food systems. In service of parsimony and considering data availability, the relationship between water and nutrition is captured by the access to safe water indicator.
**Goal 4. Ensure inclusive and equitable quality education and promote lifelong learning opportunities for all**				
**Goal 5. Achieve gender equality and empower all women and girls**				
5.4 Recognize and value unpaid care and domestic work through the provision of public services, infrastructure and social protection policies and the promotion of shared responsibility within the household and the family as nationally appropriate	5.4.1 Proportion of time spent on unpaid domestic and care work, by sex, age and location	Yes	No	This indicator is related but not specific to food systems and lacks data coverage. Gender issues in food systems are partly captured by the women's land ownership indicator.

5.a Undertake reforms to give women equal rights to economic resources, as well as access to ownership and control over land and other forms of property, financial services, inheritance and natural resources, in accordance with national laws	5.a.1 (*a*) Proportion of total agricultural population with ownership or secure rights over agricultural land, by sex; and (*b*) share of women among owners or rights-bearers of agricultural land, by type of tenure	Yes	Yes	Though the ideal indicator of women's access to land, data are currently limited. We currently use the data from the Gender and Land Rights database because it exceeds the country coverage of SDG 5.a.1. Currently there are only 46 countries with data for the SDG indicator, and as soon as this exceeds the coverage of the Gender and Land Rights database dataset (which is no longer being updated) we will replace the current indicator with the SDG indicator.
	5.a.2 Proportion of countries where the legal framework (including customary law) guarantees women's equal rights to land ownership and/or control	Yes	No	In service of parsimony, we include women's access to land as the outcome.

5.b Enhance the use of enabling technology, in particular information and communications technology, to promote the empowerment of women	5.b.1 Proportion of individuals who own a mobile telephone, by sex	Yes	No	We use the closely related World Development Indicator on mobile phone ownership per 100,000 people. Unfortunately, there is insufficient country and time coverage at present to use the SDG data, as the indicator is collected by the ITU with a novel survey that was first administered in 2015. The World Development Indicator is also based on data from the ITU. When there is sufficient coverage of the SDG indicator we will replace the indicator with SDG Indicator 5.b.1 Proportion of individuals who own a mobile telephone, by sex.
**Goal 6. Ensure availability and sustainable management of water and sanitation for all**				
6.1 By 2030, achieve universal and equitable access to safe and affordable drinking water for all	6.1.1 Proportion of population using safely managed drinking water services	Yes	No	Included in FSCI because water is an essential nutrient necessary for life. Unsafe water undermines the body's ability to absorb nutrients and increases risk of disease that also contributes to malnutrition.
6.3 By 2030, improve water quality by reducing pollution, eliminating dumping and minimizing release of hazardous chemicals and materials, halving the proportion of untreated wastewater and substantially increasing recycling and safe reuse globally	6.3.1 Proportion of domestic and industrial wastewater flows safely treated	Yes	No	Safe water captured by indicator 6.1.1 in service of parsimony.

6.4 By 2030, substantially increase water-use efficiency across all sectors and ensure sustainable withdrawals and supply of freshwater to address water scarcity and substantially reduce the number of people suffering from water scarcity	6.4.1 Change in water-use efficiency over time	Yes	No	Water use efficiency in agriculture is captured by the rate of water withdrawals indicator.
	6.4.2 Level of water stress: freshwater withdrawal as a proportion of available freshwater resources	Yes	No	Included in FSCI
6.5 By 2030, implement integrated water resources management at all levels, including through transboundary cooperation as appropriate	6.5.1 Degree of integrated water resources management	Yes	No	Water use sustainability in agriculture is captured by the rate of water withdrawals indicator. In service of parsimony, we focus on the outcome indicator.

	6.5.2 Proportion of transboundary basin area with an operational arrangement for water cooperation	Yes	No	
6.6 By 2020, protect and restore water-related ecosystems, including mountains, forests, wetlands, rivers, aquifers and lakes	6.6.1 Change in the extent of water-related ecosystems over time	Yes	No	Not specific to food systems and water availability for agriculture is captured by the sustainability of withdrawals.
6.a By 2030, expand international cooperation and capacity-building support to developing countries in water- and sanitation-related activities and programmes, including water harvesting, desalination, water efficiency, wastewater treatment, recycling and reuse technologies	6.a.1 Amount of water- and sanitation-related official development assistance that is part of a government-coordinated spending plan	Yes	No	The FSCI focuses on indicators relevant to country governments and does not include metrics related to Official Development Assistance. We acknowledge the data gap on budgetary allocations to food systems.
6.b Support and strengthen the participation of local communities in improving water and sanitation management	6.b.1 Proportion of local administrative units with established and operational policies and procedures for participation of local communities in water and sanitation management	Yes	No	Not specific to food systems and water availability for agriculture is captured by the sustainability of withdrawals. This is related but not specific to food systems and we acknowledge the data gap on food systems governance outside of urban areas (with urban areas covered by the MUFPP indicator).

**Goal 7. Ensure access to affordable, reliable, sustainable and modern energy for all**				
7.1 By 2030, ensure universal access to affordable, reliable and modern energy services	7.1.1 Proportion of population with access to electricity	Yes	No	Access to electricity is a broader indicator of development which has implications for the development and productivity of a population's food systems. It was considered in the indicator selection process but excluded because with the exception of Sub-Saharan Africa and few countries in South Asia, most countries in the world now have electricity coverage over 85%, making this indicator not very useful or relevant at differentiating countries at a global level. Because of this, we use mobile phone coverage as a proxy for infrastructure in service of parsimony.
**Goal 8. Promote sustained, inclusive and sustainable economic growth, full and productive employment and decent work for all**				
8.1 Sustain per capita economic growth in accordance with national circumstances and, in particular, at least 7 per cent gross domestic product growth per annum in the least developed countries	8.1.1 Annual growth rate of real GDP per capita	Yes	No	We focus on the share of agriculture in GDP as a proxy for rural poverty but also acknowledge the data gap of the economic value (value added) of food systems to GDP.
8.2 Achieve higher levels of economic productivity through diversification, technological upgrading and innovation, including through a focus on high-value added and labour-intensive sectors	8.2.1 Annual growth rate of real GDP per employed person	Yes	No	Not specific to food system and we acknowledge the data gap in labor productivity in food systems which we are expecting new indicators to fill based on work currently under development.

8.3 Promote development-oriented policies that support productive activities, decent job creation, entrepreneurship, creativity and innovation, and encourage the formalization and growth of micro-, small- and medium-sized enterprises, including through access to financial services	8.3.1 Proportion of informal employment in total employment, by sector and sex	Yes	No	Informal employment lacks many of the protections formal employment does, making this important for measuring the extent of vulnerabilities among food system workers, however this indicator is not specific to food systems. We acknowledge this data gap.
8.4 Improve progressively, through 2030, global resource efficiency in consumption and production and endeavour to decouple economic growth from environmental degradation, in accordance with the 10-Year Framework of Programmes on Sustainable Consumption and Production, with developed countries taking the lead	8.4.1 Material footprint, material footprint per capita, and material footprint per GDP	Yes	No	This is directly related to the sustainability of food systems as we seek to cut down on material footprints and reduce these systems' environmental impact, however this indicator is not specific to food ssytems. We acknowledge the data gap in material pollution attributable to food systems.
8.5 By 2030, achieve full and productive employment and decent work for all women and men, including for young people and persons with disabilities, and equal pay for work of equal value	8.5.1 Average hourly earnings of employees, by sex, age, occupation and persons with disabilities	Yes	No	While this would be a valuable indicator for poverty, and related to food systems if it was disaggregated by urban/rural location, there is not sufficient data coverage at present.

	8.5.2 Unemployment rate, by sex, age and persons with disabilities	Yes	No	This is also an indicator for poverty and its impact on different populations who may not be as likely to benefit from food systems due to their job insecurity. Only rural data is included in the framework, using the World Development Indicators as the rural/urban disaggregation is not accounted for in this SDG indicator.
8.6 By 2020, substantially reduce the proportion of youth not in employment, education or training	8.6.1 Proportion of youth (aged 15–24 years) not in education, employment or training	Yes	No	Because this is related but not specific to food systems, we currently include unemployment and underemployment in rural areas as something more closely related to food systems. We acknowledge the data gap in youth employment in food systems.
8.7 Take immediate and effective measures to eradicate forced labour, end modern slavery and human trafficking and secure the prohibition and elimination of the worst forms of child labour, including recruitment and use of child soldiers, and by 2025 end child labour in all its forms	8.7.1 Proportion and number of children aged 5–17 years engaged in child labour, by sex and age	Yes	No	Included in the FSCI because a large share of child labor is known to occur in agriculture.

8.b By 2020, develop and operationalize a global strategy for youth employment and implement the Global Jobs Pact of the International Labour Organization	8.b.1 Existence of a developed and operationalized national strategy for youth employment, as a distinct strategy or as part of a national employment strategy	Yes	No	This indicator is related to food systems due to its focus on the strategies countries are taking to tackle youth employment issues as key participants in food systems, especially in Africa where so many rural youth lack employment opportunities. However, data coverage is currently insufficient for consideration as an FSCI indicator.
**Goal 9. Build resilient infrastructure, promote inclusive and sustainable industrialization and foster innovation**				
9.1 Develop quality, reliable, sustainable and resilient infrastructure, including regional and transborder infrastructure, to support economic development and human well-being, with a focus on affordable and equitable access for all	9.1.1 Proportion of the rural population who live within 2 km of an all-season road	Yes	No	Access to markets is essential for food systems, and enabled by high quality transportation infrastructure. However, there is insufficient data coverage for this indicator to consider for FSCI and we use mobile phones as a proxy for infrastructure.
9.2 Promote inclusive and sustainable industrialization and, by 2030, significantly raise industry's share of employment and gross domestic product, in line with national circumstances, and double its share in least developed countries	9.2.1 Manufacturing value added as a proportion of GDP and per capita	Yes	No	Data specific to food systems do not presently exist. However, in related work we are developing a new dataset that extends throughout food systems and expect to incorporate this indicator in the future.

	9.2.2 Manufacturing employment as a proportion of total employment	Yes	No	Data specific to food systems do not presently exist. However, in related work we are developing a new dataset that extends throughout food systems and expect to incorporate this indicator in the future.
9.3 Increase the access of small-scale industrial and other enterprises, in particular in developing countries, to financial services, including affordable credit, and their integration into value chains and markets	9.3.1 Proportion of small-scale industries in total industry value added	Yes	No	Though many small-scale enterprises are in food systems because they present low barriers to entry, the ideal indicator would be specific to food systems. Furthermore, data coverage is insufficient at present for consideration as an FSCI indicator.

	9.3.2 Proportion of small-scale industries with a loan or line of credit	Yes	No	As above, this is relevant to food systems given that many small-scale enterprises are in food systems and their access to financial services is an important indicator of barriers to firm growth. However, the ideal indicator would be specific to food systems and data coverage is insufficient at present for consideration as an FSCI indicator.
9.4 By 2030, upgrade infrastructure and retrofit industries to make them sustainable, with increased resource-use efficiency and greater adoption of clean and environmentally sound technologies and industrial processes, with all countries taking action in accordance with their respective capabilities	9.4.1 CO_2 emission per unit of value added	Yes	No	While related to food systems, we use indicators that are more specific to food systems including total food systems emissions and emissions intensities for several major food products.

9.5 Enhance scientific research, upgrade the technological capabilities of industrial sectors in all countries, in particular developing countries, including, by 2030, encouraging innovation and substantially increasing the number of research and development workers per 1 million people and public and private research and development spending	9.5.1 Research and development expenditure as a proportion of GDP	Yes	No	This is not specific to food systems but we recognize the data gap of budgetary allocations to food systems, including to research.
	9.5.2 Researchers (in full-time equivalent) per million inhabitants	Yes	No	This is not specific to food systems, but we acknowledge the data gap of agriculture and food researchers at the national level.
9.a Facilitate sustainable and resilient infrastructure development in developing countries through enhanced financial, technological and technical support to African countries, least developed countries, landlocked developing countries and small island developing States	9.a.1 Total official international support (official development assistance plus other official flows) to infrastructure	Yes	No	This is not specific to food systems but we recognize the data gap of budgetary allocations to food systems, including to infrastructure. Furthermore, we focus on indicators relevant to country governments and therefore do not include metrics of Official Development Assistance.

**Goal 10. Reduce inequality within and among countries**				
**Goal 11. Make cities and human settlements inclusive, safe, resilient and sustainable**				
11.5 By 2030, significantly reduce the number of deaths and the number of people affected and substantially decrease the direct economic losses relative to global gross domestic product caused by disasters, including water-related disasters, with a focus on protecting the poor and people in vulnerable situations	11.5.2 Direct economic loss attributed to disasters in relation to global gross domestic product (GDP)	Yes	No	As noted above, we use the EM-DAT database because it has greater coverage of disaster events.
**Goal 12. Ensure sustainable consumption and production patterns**				
12.1 Implement the 10-Year Framework of Programmes on Sustainable Consumption and Production Patterns, all countries taking action, with developed countries taking the lead, taking into account the development and capabilities of developing countries	12.1.1 Number of countries developing, adopting or implementing policy instruments aimed at supporting the shift to sustainable consumption and production	Yes	No	This is not specific to food systems, so we use instead the presence of a national food system transformation pathway.
12.3 By 2030, halve per capita global food waste at the retail and consumer levels and reduce food losses along production and supply chains, including post-harvest losses	12.3.1 (*a*) Food loss index and (*b*) food waste index	Yes	Yes	Insufficient data coverage at present, but will be added to FSCI when suitable data are available.
12.4 By 2020, achieve the environmentally sound management of chemicals and all wastes throughout their life cycle, in accordance with agreed international frameworks, and significantly reduce their release to air, water and soil in order to minimize their adverse impacts on human health and the environment	12.4.2 (*a*) Hazardous waste generated per capita; and (*b*) proportion of hazardous waste treated, by type of treatment	Yes	No	This data is not specific to food systems but we note the data gap in hazardous waste attributable to food systems.

12.7 Promote public procurement practices that are sustainable, in accordance with national policies and priorities	12.7.1 Number of countries implementing sustainable public procurement policies and action plans	Yes	No	This is not specific to food systems, so we use instead the presence of a national food system transformation pathway.
12.a Support developing countries to strengthen their scientific and technological capacity to move towards more sustainable patterns of consumption and production	12.a.1 Installed renewable energy-generating capacity in developing countries (in watts per capita)	Yes	No	We have used mobile phone ownership as a proxy for infrastructure overall, as a parsimonious approach.
**Goal 13. Take urgent action to combat climate change and its impacts**[3]				
13.2 Integrate climate change measures into national policies, strategies and planning	13.2.1 Number of countries with nationally determined contributions, long-term strategies, national adaptation plans and adaptation communications, as reported to the secretariat of the United Nations Framework Convention on Climate Change	Yes	No	This is not specific to food systems, so we use instead the presence of a national food system transformation pathway. But we do use environmental indicators aligned to the agreed upon frameworks for UNFCCC accounting.

	13.2.2 Total greenhouse gas emissions per year	Yes	No	We use the total emissions from food systems as it is more specific to food systems.
13.b Promote mechanisms for raising capacity for effective climate change-related planning and management in least developed countries and small island developing States, including focusing on women, youth and local and marginalized communities	13.b.1 Number of least developed countries and small island developing States with nationally determined contributions, long-term strategies, national adaptation plans and adaptation communications, as reported to the secretariat of the United Nations Framework Convention on Climate Change	Yes	No	Not specific to food systems, so we instead use the indicators of food system resilience
**Goal 14. Conserve and sustainably use the oceans, seas and marine resources for sustainable development**				
14.2 By 2020, sustainably manage and protect marine and coastal ecosystems to avoid significant adverse impacts, including by strengthening their resilience, and take action for their restoration in order to achieve healthy and productive oceans	14.2.1 Number of countries using ecosystem-based approaches to managing marine areas	Yes	No	We focus on the outcome of sustainable fisheries management, which is specific to food systems.
14.4 By 2020, effectively regulate harvesting and end overfishing, illegal, unreported and unregulated fishing and destructive fishing practices and implement science-based management plans, in order to restore fish stocks in the shortest time feasible, at least to levels that can produce maximum sustainable yield as determined by their biological characteristics	14.4.1 Proportion of fish stocks within biologically sustainable levels	Yes	No	We use the Minderoo Foundation sustainable fisheries index because it includes both stocks and capture, providing an overview of the degree to which countries have been able to transition to sustainable marine food systems.

14.6 By 2020, prohibit certain forms of fisheries subsidies which contribute to overcapacity and overfishing, eliminate subsidies that contribute to illegal, unreported and unregulated fishing and refrain from introducing new such subsidies, recognizing that appropriate and effective special and differential treatment for developing and least developed countries should be an integral part of the World Trade Organization fisheries subsidies negotiation[4]	14.6.1 Degree of implementation of international instruments aiming to combat illegal, unreported and unregulated fishing	Yes	No	Data coverage is insufficient at present for consideration as an FSCI indicator. We acknowledge this data gap.
14.7 By 2030, increase the economic benefits to small island developing States and least developed countries from the sustainable use of marine resources, including through sustainable management of fisheries, aquaculture and tourism	14.7.1 Sustainable fisheries as a proportion of GDP in small island developing States, least developed countries and all countries	Yes	No	This indicator only includes capture (removal). We use the Minderoo Foundation sustainable fisheries index because it includes both stocks and capture, providing an overview of the degree to which countries have been able to transition to sustainable marine food systems.
14.a Increase scientific knowledge, develop research capacity and transfer marine technology, taking into account the Intergovernmental Oceanographic Commission Criteria and Guidelines on the Transfer of Marine Technology, in order to improve ocean health and to enhance the contribution of marine biodiversity to the development of developing countries, in particular small island developing States and least developed countries	14.a.1 Proportion of total research budget allocated to research in the field of marine technology	Yes	No	Research of marine technology Data coverage is insufficient at present for consideration as an FSCI indicator. We acknowledge the data gap of budgetary allocations to food systems, including to fisheries.

14.b Provide access for small-scale artisanal fishers to marine resources and markets	14.b.1 Degree of application of a legal/regulatory/ policy/institutional framework which recognizes and protects access rights for small-scale fisheries	Yes	Yes	Not considered for the FSCI since it is more relevant in countries with ocean fisheries. It may be considered in the future to fill the data gap on indicators relevant to fisheries and governance of food systems.
**Goal 15. Protect, restore and promote sustainable use of terrestrial ecosystems, sustainably manage forests, combat desertification, and halt and reverse land degradation and halt biodiversity loss**				
15.3 By 2030, combat desertification, restore degraded land and soil, including land affected by desertification, drought and floods, and strive to achieve a land degradation-neutral world	15.3.1 Proportion of land that is degraded over total land area	Yes	No	Not specific to food systems and to date the indicator has only been calculated for a single year. We acknowledge the interrelationship to food systems and may consider this indicator in the future.
15.7 Take urgent action to end poaching and trafficking of protected species of flora and fauna and address both demand and supply of illegal wildlife products	15.7.1 Proportion of traded wildlife that was poached or illicitly trafficked	Yes	No	Data are not currently available for this indicator, and it includes wildlife traded as food as well as other wildlife trade and is therefore not specific to food systems. However, if data become available, this and the illegal fishing indicators could be considered to fill data gaps under food systems governance.
15.9 By 2020, integrate ecosystem and biodiversity values into national and local planning, development processes, poverty reduction strategies and accounts	15.9.1 (*a*) Number of countries that have established national targets in accordance with or similar to Aichi Biodiversity Target 2 of the Strategic Plan for Biodiversity 2011–2020 in their national biodiversity strategy and action plans and the progress reported towards these targets; and (*b*) integration of biodiversity into national accounting and reporting systems, defined as implementation of the System of Environmental-Economic Accounting	Yes	No	We use the food system specific indicator of presence of a national food system transformation pathway, which incorporates goals of environmental sustainability including biodiversity.

15.c Enhance global support for efforts to combat poaching and trafficking of protected species, including by increasing the capacity of local communities to pursue sustainable livelihood opportunities	15.c.1 Proportion of traded wildlife that was poached or illicitly trafficked	Yes	No	See 15.17.1
**Goal 16. Promote peaceful and inclusive societies for sustainable development, provide access to justice for all and build effective, accountable and inclusive institutions at all levels**				
16.10 Ensure public access to information and protect fundamental freedoms, in accordance with national legislation and international agreements	16.10.2 Number of countries that adopt and implement constitutional, statutory and/or policy guarantees for public access to information	Yes	No	We use the national level data on public access to information instead of the SDG indicator.
**Goal 17. Strengthen the means of implementation and revitalize the Global Partnership for Sustainable Development**				
**Finance**				
17.11 Significantly increase the exports of developing countries, in particular with a view to doubling the least developed countries' share of global exports by 2020	17.11.1 Developing countries' and least developed countries' share of global exports	Yes	No	This indicator is not specific to trade in agrifood items.
17.12 Realize timely implementation of duty-free and quota-free market access on a lasting basis for all least developed countries, consistent with World Trade Organization decisions, including by ensuring that preferential rules of origin applicable to imports from least developed countries are transparent and simple, and contribute to facilitating market access	17.12.1 Weighted average tariffs faced by developing countries, least developed countries and small island developing States	Yes	No	This indicator is not specific to trade in agrifood items.
**Systemic issues**				

Policy and institutional coherence				
17.14 Enhance policy coherence for sustainable development	17.14.1 Number of countries with mechanisms in place to enhance policy coherence of sustainable development	Yes	No	This helps reflect the extent to which countries are taking action to ensure they follow sustainable development policies, which are key for the development of their food systems. However, indicators at the supra-national level were not considered for inclusion in FSCI and data only exist for a small proportion of all countries. Furthermore, this indicator is not specific to policy coherence for food systems transformation. We acknowledge this as a data gap and in related efforts we are working to develop an indicator for policy coherence for food systems transformation.
Multi-stakeholder partnerships				
Data, monitoring and accountability				
	17.18.3 Number of countries with a national statistical plan that is fully funded and under implementation, by source of funding	Yes	No	This helps show the degree to which countries are capable of measuring progress in their sustainable development goals, including the development of their food systems.

Table 4.2 Related and other relevant initiatives

Initiative Name	Description	Distinction from FSCI
50x2030	50x2030 is a 10-year, ~US$500 million initiative that aims to increase the capacity of 50 low and lower middle-income countries to produce, analyze, interpret, and apply data to decisions in the agricultural sector that support rural development and food security. It is Implemented through a unique partnership between the World Bank, Food and Agriculture Organization of the United Nations (FAO) and the International Fund for Agricultural Development (IFAD).	50x2030 focuses on improving country-level data systems, particularly for collecting new survey data. In contrast, FSCI will not collect new data or build country capacity to do so. The two are complementary in the FSCI can identify key indicators for 50x2030 to collect data on, helping with their priority setting, and flag gaps where more data is needed, which 50x2030 can then help fill.
Access to Nutrition Initiative (ATNI) - Global Index	ATNI is hosted by the Access to Nutrition Foundation, an independent not-for-profit organization based in the Netherlands that works internationally.<br>ATNI focuses on developing tools and initiatives that track and drive the contribution made by the food and beverage sector to addressing the world's global nutrition challenges.<br>ATNI actively seeks partnerships with other organizations taking a multi-stakeholder, holistic approach to everything it does.<br>Funding comes from foundations, governments and fees. ATNI does not take any funding from — nor undertake projects commissioned by — food and beverage companies or industry associations.	ATNI focuses primarily on tracking performance of major food and beverage firms vis-a-vis nutrition outcomes. It does not generally look beyond these firms, nor does it look beyond nutrition, and its unit of analysis is the firm. In contrast, FSCI considers country-level data and looks well beyond nutrition, with private sector companies being only one stakeholder of interest among many.

Accountability Pact	A statement to set out a collaborative agenda for measurement, monitoring, and accountability following the UNFSS	The Pact focuses on galvanizing action from researchers and others to hold leaders accountable for food systems transformation; it does not endorse any specific indicators or curate, analyse, or present any actual data, unlike the FSCI. The two are complementary in that FSCI will be useful source of curated data that can be used in accountability efforts and flags data gaps where particular work on measurement and monitoring are needed and that, if the Pact leads to better monitoring and measurement of food systems transformation by signatories, those data could feed into FSCI in later years, helping to fill data gaps.

Agrifood Systems Technologies and Innovations Outlook (ATIO)	Agrifood system transformation to achieve the Sustainable Development Goals requires increased attention to developing, adapting and diffusing impactful science, technology and innovation (STI). Current levels and patterns of STI uptake are inadequate to facilitate needed agrifood system transformations, especially in today's low- and middle-income countries. Moreover, the descriptive and evaluative evidence on current and emergent STI is also insufficiently well understood to permit intentional management of STI to meet the multiple objectives of future agrifood systems: efficient, inclusive, resilient and sustainable. This report introduces the vision, rationale, scope and methods for new knowledge products FAO will launch as part of a new Agrifood System Technologies and Innovations Outlook (ATIO). ATIO's objective is to curate existing information on the current, measurable state of STI and upcoming changes, as well as their transformative potential, to inform evidence-based policy dialogue and decisions, including on investments.	ATIO focuses specifically on science, technology and innovation within agrifood systems, tracking progress on discrete technologies and innovations that can be used within those systems. In contrast, FSCI is tracking outcomes and key characteristics of the agrifood systems themselves. The two are complementary in that science, technology and innovation will be key to achieving desires shifts in the outcomes that FSCI tracks, and that FSCI can be used to pinpoint outcomes or areas of the food system for which additional technology and innovation are particularly needed.

Ceres 2030	Ceres2030 has brought together economic modelling, machine learning, and evidence-based synthesis into one initiative, helping fill a major knowledge gap in the field of agricultural and food policy. Ceres2030 connects this knowledge back to the donor community, making sure decision makers have the cost figures and evidence they need when deciding where and how to make their investments. The partnership brought together Cornell University, the International Food Policy Research Institute (IFPRI) and the International Institute for Sustainable Development (IISD).<br>Funding support came from Germany's Federal Ministry of Economic Cooperation and Development (BMZ) and the Bill & Melinda Gates Foundation (BMGF).	Ceres2030 focused on identifying impactful interventions and investments that can support reductions in hunger and food insecurity, with its analysis focusing primarily in agricultural production and at the level of the intervention/investment. In contrast, FSCI is tracking outcomes and key characteristics at the level of the overall food system, looking beyond hunger as an outcome and agriculture as a sector and without a focus on specific interventions or investments. The two are complementary in that investments and interventions will be key to achieving desires shifts in the outcomes that FSCI tracks, and FSCI emphasizes continued undernourishment and food insecurity as a central challenge of food system transformation.
EDGAR-food	EDGAR is a multipurpose, independent, global database of anthropogenic emissions of greenhouse gases and air pollution on Earth. EDGAR provides independent emission estimates compared to what reported by European Member States or by Parties under the United Nations Framework Convention on Climate Change (UNFCCC), using international statistics and a consistent IPCC methodology.<br>EDGAR provides both emissions as national totals and gridmaps at 0.1 x 0.1 degree resolution at global level, with yearly, monthly and up to hourly data.	EDGAR's focus is narrower than the FSCI's, looking only at GHG emissions and air pollution; at the same time, its level of data resolution for this topic is much higher than that of the FSCI. The two are complementary in that FSCI highlights the importance of GHG emissions as one food system outcome among many, and EDGAR offers considerably more detail on how these vary across space and time.

FACT Alliance	The FACT Alliance embarked on a project with USAID, co-led by J-WAFS and D-Lab, that uses a combination of machine learning and traditional systematic review to identify evidence gaps in USAID's evaluation metrics in the areas of agriculture, nutrition, water, and resilience. The mission of the FACT Alliance is to transform the sustainability of food systems through collaborative, actionable research.<br>Central to the work of the FACT Alliance is the development of new methodologies for aligning data across scales and food systems components, improving data access, integrating research across the diverse disciplines that address aspects of food systems, making stakeholders partners in the research process, and assessing impact in the context of complex and interconnected food and climate systems.	FACT focuses primarily on climate impacts of the food system and on the development of new methodologies and conducting primary research. In contrast, the FSCI takes a broader perspective across food systems domains and focuses on indicator prioritisation and curation and analysis of existing data.
Food Action Alliance	The Food Action Alliance was catalysed by the World Economic Forum, International Fund for Agricultural Development (IFAD) and Rabobank, and today engages over 35 strategic and affiliate partners, in addition to a vast network of over 700 global and regional organisations including government, business, international organisations, civil society and farmer organisations. This allows the FAA to provide strong access to a wide range of implementing, commercial and funding partners across the food system with the ability to design and execute complex systemic solutions.	The FAA aims to focus on stakeholder coordination and collaboration in support of implementing food systems transformation pathways. Unlike FSCI, it does not have a focus on data or monitoring of food systems transformation. The two are complementary in that FSCI highlights the need for transformation and priorities for transformative policies, and tracks their ultimate outcomes, while the FAA could support cooperation around implementing those transformative policies.

Food Sustainability Index	The Food Sustainability Index (FSI), developed by the Economist Intelligence Unit (EIU) with the Barilla Center for Food & Nutrition (BCFN), measures the sustainability of food systems in 67 countries around three key issues outlined in the 2015 BCFN Milan Protocol and designed around the Sustainable Development Goals (SDGs): nutrition, sustainable agriculture and food loss and waste. The index looks at policies and outcomes around sustainable food systems and diets through a series of key performance indicators that consider environmental, social and economic sustainability.	The FSCI takes a broader scope, looking across a wider set of domains and indicators within those domains, as well as having global coverage.
Food system sustainability metric (Bene et. al.)	Compiles a metric of **12 key drivers** of food system from a globally-representative set of low, middle, and high-income countries and analyze the relationships between these drivers and a composite index that integrates the four key dimensions of food system sustainability, namely: food security & nutrition, environment, social, and economic dimensions	Bene et al is a one-off paper, not an ongoing tracking initiative and aims to analyse correlations and relationships between four dimensions of food system sustainability and specific drivers. While there is overlap between the domains and indicators examined by Bene et al and those of the FSCI, the FSCI includes some additional areas (e.g., governance) and aims to be an ongoing tracking initiative.

Food Systems Initiative on Shaping the Future of Food (WEF)	The mission of the World Economic Forum's System Initiative on Shaping the Future of Food is to build inclusive, sustainable, efficient, and nutritious food systems through leadership-driven, market-based action and collaboration, informed by insights and innovation, in alignment with the Sustainable Development Goals.<br>The System Initiative on Shaping the Future of Food aims to:<br>Strengthen global food systems by developing new insights; facilitating collaboration on priority action areas, including leveraging technology and innovation for food systems change; and mobilizing leadership and expertise at the global level.<br>Supporting regional and country platforms by achieving the New Vision for Agriculture through the Food Action Alliance - strengthening multistakeholder collaboration at the country and regional level, and by mobilizing new investments, partnerships, and best practices to achieve concrete results.<br>Harness the power of technological innovations to transform the food system through Innovation with a Purpose, as a large-scale partnership aggregator and project accelerator.	This Initiative aims to focus on stakeholder collaboration, mobilizing leadership and expertise, and one-off analysis on specific topics. Unlike FSCI, it does not have a focus on data curation, analysis, or monitoring of food systems transformation. The two are complementary in that FSCI highlights the need for transformation and priorities for transformative policies, and tracks their ultimate outcomes, while this Initiative could support cooperation around implementing those transformative policies and add new insights on specific topics related to them.

Food System Development Pathways (Gaupp et al.)	"Food system development pathways (FSDPs)… elicit the biophysical and technical feasibility of food systems transformation and potential trade-offs among multiple food systems objectives, notably between health, environmental and inclusion goals. These pathways are meant to provide decision-makers with possible combinations of policy options to achieve an inclusive food systems transformation."	Gaupp et al (2021) is similar to the FSCI in that they present a set of indicators that can be used to capture food systems transformation, spanning health, environment, and inclusion. However, their intention is not to track these indicators over time or to compare across countries but rather to use them to illustrate a framework and analytical approach to prioritising policies and interventions. They thus present limited quantitative data on the indicators. The FSCI also includes additional domains (resilience and governance) that have little overlap with those of Gaupp et al.
Global Strategy to Improve Agricultural and Rural Statistics	The purpose of the global strategy is to provide a framework for national and international statistical systems that enables them to produce and to apply the basic data and information needed to guide decision making in the twenty-first century. The Global Strategy is the result of an extensive consultation process with national and international statistical organizations as well as with agriculture ministries and other governmental institutions represented in Food and Agricultural Organization of the United Nations (FAO) governing bodies.	The Global Strategy is focused on guiding governments and national statistics agencies to collect more accurate and efficient data. It concentrates on data collection and the collection of many of the indicators from which the FSCI draws.

INFORMAS	INFORMAS (International Network for Food and Obesity / Non-communicable Diseases (NCDs) Research, Monitoring and Action Support) is a global network of public-interest organisations and researchers that aims to monitor, benchmark and support public and private sector actions to increase healthy food environments and reduce obesity and NCDs and their related inequalities.<br>INFORMAS supports the WHO's Global Action Plan for the Prevention and Control of Non-Communicable Diseases (2013-2020) and the World Cancer Research Fund International NOURISHING framework.	INFORMAS has a primary focus on NCD/obesity prevention (though expanding into other issues) and looks particularly at policy-related indicators. It offers very detailed analysis and indicators on these topics, but for only 42 countries and without the coverage of larger food system issues (e.g., undernutrition, livelihoods, resilience) that the FSCI provides.
CAADP Results Framework (Malabo Declaration)	The Twenty-Third ordinary session of the African Union Assembly held in Malabo, Equatorial Guinea recommitted to the Comprehensive African Agriculture Development Programme (CAADP) principles and goals and defined a set of targets and goals, referred to as the Accelerated Agricultural Growth and Transformation Goals 2025. In their Declaration in Malabo, the Heads of State recalled the progress made and noted the need for monitoring, tracking and reporting on the implementation of the Declaration using the CAADP Results Framework.<br>Progress tracked by the CAADP Biennial Review Report (latest available: 2015-2021)	The CAADP Results Framework primarily tracks indicators related to agricultural production and productivity, and is focused only on Africa. FSCI considers a broader set of food system domains and indicators, with a global focus.
Regional Strategic Analysis and Knowledge Support System (ReSAKSS)	The Regional Strategic Analysis and Knowledge Support System (ReSAKSS) compiles and analyzes national indicators to help monitor the progress of the CAADP, Africa's policy framework for agricultural transformation.	As ReSAKSS aims to support monitoring progress according to the CAADP Results Framework, it also primarily tracks indicators related to agricultural production and productivity, and is focused only on Africa. FSCI considers a broader set of food system domains and indicators, with a global focus.

Africa Trends and Outlooks Report (ATOR)	A product of ReSAKSS. Latest ATOR 2022 on Agrifood processing strategies for successful food systems transformation in Africa https://www.resakss.org/sites/default/files/ReSAKSS_AW_ATOR_2022.pdf	As a product of ReSAKSS aiming to support monitoring progress according to the CAADP Results Framework, ATOR's main focus is on agricultural production and productivity, and it is focused only on Africa. FSCI considers a broader set of food system domains and indicators, with a global focus.
Nutrition for Growth (N4G) (Global Nutrition Report) & Nutrition Accountability Framework	Nutrition for Growth (N4G) is a global pledging moment to drive greater action toward ending malnutrition and helping ensure everyone, everywhere can reach their full potential.<br><br>Over the past decade, the governments of the United Kingdom, Brazil, and Japan each stepped up to mobilize N4G nutrition commitments against the backdrop of the Olympics—a symbol of health, strength, and human potential. With the support of governments, donors, civil society, and the private sector, the result has been unprecedented, coordinated, and impactful commitments to improve global nutrition.<br><br>The Nutrition Accountability Framework (NAF) creates the world's first independent and comprehensive platform for registering SMART nutrition commitments and monitoring nutrition action. It has been endorsed by the government of Japan, the SUN Movement, the World Health Organization, UNICEF, USAID and many others, and will hold all data on commitments made for the Tokyo Nutrition for Growth (N4G) Summit 2021 and progress made against them over time.	The NAF focuses on tracking commitments (primarily from N4G) and progress on them, with a narrow focus on nutrition; its analysis is at the level of the commitment. In contrast, FSCI takes a holistic view across food system domains and considers data at the country level, with no focus on commitment tracking.
OmniAction	OmniAction takes existing metrics and methodologies from across the food system, supports the development of those that are less established, and harmonises the data into one global and unifying framework. We are a not-for-profit making this resource available for the public good	While this initiative has not yet published its framework or data, its intended focus appears to be on rating individual foods or food products, as opposed to tracking transformation at the level of the food system -- the goal of the FSCI.

Progress towards Sustainable Agriculture (PROSA) (FAO)	A methodological approach aimed at measuring progress towards sustainable agriculture in countries and across agri-food systems typologies, by measuring socio-economic and environmental dimensions with available national statistics, with sixteen indicators defined and constructed from FAOSTAT data	PROSA primarily considers indicators related to agricultural production and sustainability, drawing on FAO data. FSCI considers a broader set of food system domains and indicators as well as broader set of data sources.
Scaling Up Nutrition (SUN)	Since 2010, the SUN Movement has inspired a new way of working collaboratively to end malnutrition, in all its forms. With the governments of SUN Countries in the lead, it unites people—from civil society, the United Nations, donors, businesses and researchers—in a collective effort to improve nutrition.<br>The Scaling Up Nutrition Movement Strategy SUN 3.0 (2021–2025) continues to highlight the importance of nutrition as a universal agenda – and one which is integral to achieving the Sustainable Development Goals (SDGs).<br>To realize the vision of a world without hunger and malnutrition, the SUN Movement Principles of Engagement guide members as they work in a multi-sectoral and multi-stakeholder space to effectively work together to end malnutrition, in all its forms. These principles ensure that the SUN Movement is flexible while maintaining a common purpose and mutual accountability.	SUN is a global movement aimed primarily at increasing focus on malnutrition and supporting collaboration among actors to end it. It does not have a major focus on data curation, analysis, or monitoring and focuses only on nutrition, as opposed to the other food system domains the FSCI considers. The two are complementary in that FSCI flags areas where action on nutrition is particularly needed, and tracks progress on that at the level of the food system, whereas SUN can support ensuring that action happens within member countries.

Sustainable Agriculture Matrix (SAM)	SAM aims to serve as a platform to engage conversations among stakeholders involved in agriculture and to forge positive changes towards sustainability while avoiding unintended consequences. SAM reports indicators by country and year so that end-users can track a country's progress along time and make comparison across countries among different dimensions of sustainability. However, SAM does not only rely on available data reported on the national level but also synthesizes data in the literature and in other public domains on various spatial and temporal scales.<br>There are mainly three goals for the development and publication of SAM:<br>Provide a consistent and transparent measurement of countries' performance in sustainable agricultural production<br>Investigate the socioeconomic and ecological drivers for a country achieving sustainability.<br>Quantify and visualize the impacts of current agricultural production on its future sustainability.	SAM focuses on agriculture and the sustainability of agricultural production; FSCI considers a broader set of food system domains and indicators, without as much detail on the topic of sustainable agriculture.

Sustainable Nutrition Security (SNS) food system metrics (Gustafson et al.)	New methodology based on the concept of "sustainable nutrition security" (SNS). This novel assessment methodology is intended to remedy both kinds of deficiencies in the previous work by defining seven metrics, each based on a combination of multiple indicators, for use in characterizing sustainable nutrition outcomes of food systems: **(1) food nutrient adequacy; (2) ecosystem stability; (3) food affordability and availability; (4) sociocultural wellbeing; (5) food safety; (6) resilience; and (7) waste and loss reduction**. Each of the metrics comprises multiple indicators that are combined to derive an overall score (0–100).	Gustafson et al is a one-off paper, not an ongoing tracking initiative, and aimed to develop new composite metrics. While there is overlap between the domains and indicators examined by Gustafson et al and those of the FSCI, the FSCI: does not aim to create composite metrics; includes some additional areas (e.g., governance, more details on diets) and omits some of those included by Gustafson; and aims to be an ongoing tracking initiative.
The Paris Declaration on Aid Effectiveness and the Accra Agenda for Action	At the Second High Level Forum on Aid Effectiveness (2005) it was recognised that aid could - and should - be producing better impacts. The Paris Declaration was endorsed in order to base development efforts on first-hand experience of what works and does not work with aid. It is formulated around five central pillars: Ownership, Alignment, Harmonisation, Managing for Results and Mutual Accountability.<br><br>In 2008 at the Third High Level Forum on Aid Effectiveness all OECD donors, more than 80 developing countries and some 3 000 civil society organisations from around the world joined representatives of emerging economies, United Nations and multilateral institutions and global funds in the negotiations leading up to and taking place during the Accra meeting. The Accra Agenda for Action (AAA) was endorsed. The AAA both reaffirms commitment to the Paris Declaration and calls for greater partnership between different parties working on aid and development.	The Accra Agenda / Paris Declaration is process oriented: it focuses on the process of development and the provision of development aid. As such, its monitoring also takes this focus. Moreover, it is not specific to food systems, considering multiple areas of development. In contrast, FSCI looks at outcomes of food systems without a focus on the provision of development aid of the process of setting development agendas. The two are complementary in that effective use of development aid and appropraite setting of development agendas are critical for achieving the food system transformation advocated for, and tracked by, FSCI.

World Benchmarking Alliance	To achieve the SDGs by 2030, we need transformational change from farm to fork. Food systems transformation requires large-scale and fundamental action led by those who drive environmental, health and social pressures in the system.<br>The benchmark's aim is to stimulate the most influential food and agriculture companies to apply sustainable business practices throughout their operations as well as use their influence to encourage value chain partners to do the same.<br>Developed a framework that set out the critical areas and topics where private sector action is needed and where companies must step up their efforst to collectively transform the food system.	The benchmark focuses on tracking performance of major food and beverage companies. It does not look beyond these companies, and its unit of analysis is the company. In contrast, FSCI considers country-level data, with private sector companies being only one stakeholder of interest among many.
Global Nutrition Report and Country Nutrition Profiles	The Global Nutrition Report provides a concise data-focused update on the state of diets and nutrition in the world. Its Country Profiles enable users to explore the latest data on nutrition at global, regional and country level.	The GNR focuses just on diets and nutrition, without considering the other domains included in the FSCI (i.e., sustainability, resilience, livelihoods, and governance). The two are complementary in that FSCI highlights the importance of nutrition outcomes (among other food system outcomes), while GNR provides a more in-depth perspective on diet and nutrition indicators.
Food Systems Dashboard	The Food Systems Dashboard combines data from multiple sources to give users a complete view of food systems. Users can compare components of food systems across countries and regions. They can also identify and prioritize ways to sustainably improve diets and nutrition in their food systems.	The Dashboard aims at a complete or comprehensive view of food systems, including some that are not useful for tracking progress towards transformation but have other purposes, while FSCI focuses on identifying a smaller set of trackable key indicators. The Dashboard also does not currently include indicators for livelihoods or governance. The two are complementary in that many FSCI indicators are included in the Dashboard, which provides an easy platform for visualization and comparison.

The State of Food Security and Nutrition in the World	The State of Food Security and Nutrition in the World (SOFI) is an annual flagship report jointly prepared by FAO, IFAD, UNICEF, WFP and WHO to inform on progress towards ending hunger, achieving food security and improving nutrition and to provide in depth analysis on key challenges for achieving this goal in the context of the 2030 Agenda for Sustainable Development. The report targets a wide audience, including policy-makers, international organizations, academic institutions and the general public.	These annual reports focus on food security and nutrition, with in-depth analysis of a given topic (which varies by the year), without considering the other domains included in the FSCI (i.e., sustainability, resilience, livelihoods, and governance). The two are complementary in that FSCI highlights the importance of nutrition outcomes (among other food system outcomes), while SOFI provides a more in-depth perspective on diet and nutrition indicators, and the indicators developed and reported by SOFI are among those included in the FSCI
The State of Food and Agriculture	The State of Food and Agriculture, one of FAO's major annual flagship publications, aims at bringing to a wider audience balanced science-based assessments of important issues in the field of food and agriculture. Each edition of the report contains a comprehensive, yet easily accessible, overview of a selected topic of major relevance for rural and agriculture development and for global food security.	These annual reports focus on agriculture, with in-depth analysis of a given topic (which varies by the year). It does not aim to comprehensively track food systems indicators and its focus is on agriculture. The two are complementary in that indicators developed and reported by SOFA play a key role in plugging food system data gaps and could feed into those included in the FSCI.
Healthy Diets Monitoring Initiative (WHO, UNICEF, FAO) managed by TEAM	The overall objective of the Healthy Diets Monitoring Initiative is to enable national and global monitoring of the diet quality to inform policies and programmes across a wide range of sectors. Specifically, the Initiative will determine how best to measure healthy diets for different purposes, build consensus, and promote uptake and use of healthy diet measures and indicators among national and global stakeholders. It is currently in planning stages: The work of the Initiative will be phased over three to five years, with the initial planning and priority-setting occurring during summer 2022 and a first technical consultation in late 2022, with further work to follow in 2023-24.	This initiative is exclusively focused on healthy diets, whereas FSCI includes healthy diets as part of a broader agenda to monitor the whole of food systems. When this cross-UN initiative produces recommendations, and subsequently when data become available, the indicators will be considered for inclusion in the FSCI.

Table 4.3 Food System Transformation Pathway characteristics mapped to FSCI architecture and themes

Theme	Domains	Indicators	Pathways theme 1	Pathways theme 2
Diets, Nutrition, and Health	Food environments	Cost of a healthy diet	Achieving Zero Hunger	Healthy Diets from Sustainable Food Systems for Children & All
		Availability of fruits and vegetables	Different Forms of Malnutrition	Healthy Diets from Sustainable Food Systems for Children & All
		Retail value of ultra-processed foods per capita	Different Forms of Malnutrition	Food quality and safety
		% population using safely managed drinking water services (SDG 6.1.1)	Food quality and safety	
	Food security	% Population experiencing moderate or severe food insecurity (SDG 2.1.2)	Achieving Zero Hunger	
		% Population who cannot afford a healthy diet	Achieving Zero Hunger	Different Forms of Malnutrition
		PoU: Prevalence of Undernourishment (SDG 2.1.1)	Achieving Zero Hunger	
	Diet quality	All-5: consumption of all 5 food groups	Different Forms of Malnutrition	
		MDD (IYCF): minimum dietary diversity for infants and young children	Different Forms of Malnutrition	Healthy Diets from Sustainable Food Systems for Children & All
		MDD-W: minimum dietary diversity for women	Different Forms of Malnutrition	Food Systems for Women and Girls
		NCD-Protect	Different Forms of Malnutrition	
		NCD-Risk	Different Forms of Malnutrition	
		Sugar-sweetened soft drink consumption	Different Forms of Malnutrition	
		Zero fruit or vegetable consumption, adults	Different Forms of Malnutrition	
		Zero fruit or vegetable consumption, children 6-23 months	Different Forms of Malnutrition	Healthy Diets from Sustainable Food Systems for Children & All

Environment, natural resources, and production	Greenhouse gas emissions	Greenhouse gas emissions intensity	Sustainable Productivity Growth	
		Agri-food systems greenhouse gas emissions	Sustainable Livestock	Halting Deforestation & Conversion from Agricultural Commodities
	Production	Food product yield per food group	Healthy Diets from Sustainable Food Systems for Children & All	
	Land	Cropland expansion (% change)	Halting Deforestation & Conversion from Agricultural Commodities	
	Water	Agriculture water withdrawal as % of total renewable water resources	Water	Healthy Diets from Sustainable Food Systems for Children & All
	Biosphere integrity	Fishery health index progress score	Aquatic / Blue Foods	Agroecology
		Functional integrity: % agricultural land with minimum level of natural habitat	Agroecology	Nature-Positive Innovation
	Pollution	Total pesticides per unit of land (kg/ha)	Sustainable Productivity Growth	
		Sustainable nitrogen management index	Soil Health	Nature-Positive Innovation
Livelihoods, Poverty, and Equity	Poverty and income	Share of agriculture in GDP	Family Farming	Vulnerable Peoples Food Systems
	Employment	Underemployment rate, urban and rural	Decent Work and Living Incomes and Wages for All Food Systems Workers	Equitable Livelihoods
		Unemployment rate, urban and rural	Decent Work and Living Incomes and Wages for All Food Systems Workers	Equitable Livelihoods
	Social protection	Social protection adequacy	Social Protection for Food Systems Transformation	
		Social protection coverage	Social Protection for Food Systems Transformation	
	Rights	% of children 5-17 engaged in child labor	Decent Work and Living Incomes and Wages for All Food Systems Workers	
		Female share of landholdings	Family Farming	Food Systems for Women and Girls

Governance	Shared vision and strategic planning	Civil society participation index		
		Presence of a national food system transformation pathway	Healthy Diets from Sustainable Food Systems for Children & All	Climate and disasters Resilient Development Pathways (CRDP)
		% urban population living in cities signed onto the Milan Urban Food Policy Pact	Urban Food Systems	
		Degree of legal recognition of the Right to Food	Vulnerable Peoples Food Systems	
	Effective implementation	International Health Regulations State Party Assessment report (IHR SPAR), Food safety capacity	Food quality and safety	
		Government effectiveness index		
		Presence of health-related food taxes	The True Value of Food	
	Accountability	Guarantees for public access to information (SDG 16.10.2)		
		V-Dem Accountability index		
		Open Budget Index Score		
Resilience	Exposure to shocks	Ratio of total damages of all disasters to GDP	Climate and disasters Resilient Development Pathways (CRDP)	
	Agro- and Food Diversity	Number of (a) plant and (b) animal genetic resources for food and agriculture secured in either medium- or long-term conservation facilities (SDG 2.5.1)	Agrobiodiversity	
		Proportion of agricultural land with minimum level of species diversity (crop and pasture)	Agrobiodiversity	
	Resilience capacities	Dietary sourcing flexibility index	Resilient Food Supply Chains	Resilience to Stress and Vulnerabilities
		Mobile cellular subscriptions (per 100 people)	Climate and disasters Resilient Development Pathways (CRDP)	Resilience to Stress and Vulnerabilities
		Social capital index	Resilience to Stress and Vulnerabilities	Resilience to shocks and violent conflicts and food crisis
	Resilience responses/strategies	Coping strategies index	Resilience to Stress and Vulnerabilities	Resilience to shocks and violent conflicts and food crisis
	Long-term outcomes	Food supply variability	Resilient Food Supply Chains	Resilience to Stress and Vulnerabilities
		Food price volatility	Resilient Food Supply Chains	Resilience to Stress and Vulnerabilities

Notes: Pathways that are not covered in the FSCI are Food Loss and Waste (identified gap); School Meals: Nutrition and Health and Education for Every Child; Sustainable Consumption; Restoring grasslands and shrublands and savannahs; Indigenous Peoples Food Systems; Youth Food Systems; Resilience to Health Crises. FSCI indicators that do not align to themes in food system transformation pathways are all in the governance theme and include: Civil society participation index; Government effectiveness index; Guarantees for public access to information (SDG 16.10.2); V-Dem Accountability index; Open Budget Index Score.
